# Genomic epidemiology of emerging ESBL-producing *Salmonella* Kentucky *bla*_CTX-M-14b_ in Europe

**DOI:** 10.1080/22221751.2020.1821582

**Published:** 2020-09-30

**Authors:** Claudia E. Coipan, Therese Westrell, Angela H.A.M. van Hoek, Erik Alm, Saara Kotila, Bas Berbers, Sigrid C.J. de Keersmaecker, Pieter-Jan Ceyssens, Maria Louise Borg, Marie Chattaway, Jacquelyn McCormick, Timothy J. Dallman, Eelco Franz

**Affiliations:** a National Institute for Public Health and the Environment, Netherlands; b European Centre for Disease Prevention and Control, Sweden; c Sciensano, Belgium; d Health Promotion and Disease Prevention Directorate, Malta; e Public Health England, UK

**Keywords:** Chromosomal antibiotic resistance, ESBL, ESC, clonal expansion, *bla*_CTX-M-14b_, *Salmonella* Kentucky

## Abstract

Global dissemination of ciprofloxacin-resistant *Salmonella* Kentucky has been observed over the past decades. In recent years, there have been reports of extended-spectrum β-lactamase (ESBL) producing *S*. Kentucky. Routine surveillance at the European Centre for Disease Prevention and Control (ECDC) detected cases with a ciprofloxacin-resistant *S*. Kentucky with the ESBL-gene *bla*_CTX-M-14b_. Ensuing research identified 78 cases in 2013–2018 in eight European countries. Compared to other *S*. Kentucky and non-typhoidal *Salmonella* infections, reported to the European Surveillance System, these cases were more likely to be elderly and to present urinary-tract infections. Bayesian time-scaled phylogeny on whole genome sequences of isolates from these cases and supplementary isolates from public sequence databases was used to infer the origin and spread of this clone. We dated the origin of the *bla*_CTX-M-14b_ clone to approximately 2005 in Northern Africa, most likely in Egypt. The geographic origin predicted by the phylogenetic analysis is consistent with the patients’ travel history. Next to multiple introductions of the clone to Europe from Egypt, our analysis suggests that in some parts of Europe the clone might have formed a stable population, from which further spread has occurred. Comparative genomics indicated that the *bla*_CTX-M-14b_ gene is present on the bacterial chromosome, within the type VI secretion system region. The *bla*_CTX-M-14b_ gene is integrated downstream of the *hcp1* gene, on a 2854 bp plasmid fragment containing also IS*Ecp1*. This is the first report of a chromosomally integrated CTX-M gene in *Salmonella* spp. in Europe, previous studies having identified similar genes only on plasmids.

## Introduction

Encompassing more than 2500 serovars and a wide genetic diversity, *Salmonella* spp. is the second most prevalent cause of food-borne human infections in Europe, with an average of approximately 95,000 laboratory-confirmed cases reported per year [1] in the last decade. With various ecological and epidemiological patterns, the numerous serovars circulate in a broad range of animal reservoirs and environmental niches from where they can spread to humans [2,3]. The diverse environmental exposure patterns and genetic plasticity of the bacteria (including the possibility to host a variety of mobile genetic elements) has shaped an accumulation of antimicrobial resistance genes [4]. This is an emerging public health problem as some of the *Salmonella* isolates have become multidrug resistant, displaying resistance to several of the antibiotics commonly used for treatment of salmonellosis. One of the serovars that have acquired a large number of antibiotic resistance genes, is *S. enterica* subsp. *enterica* serovar Kentucky (henceforth named *S*. Kentucky). Showing a steady number of ∼600 cases reported per year EU-wide [1] and resistance to fluoroquinolones, extended-spectrum β-lactams, and in some cases even to carbapenems, *S*. Kentucky is considered a high priority resistant pathogen according to the WHO global priority list [5].

In recent years, various studies shed light on the emergence, drivers, and potential threats of *S*. Kentucky in general and of sequence type (ST) 198 in particular. *S*. Kentucky ST 198 seems to be a versatile microorganism that adapts easily to selection pressure exerted by the use of antibiotics in various environments. It has thus evolved from absence of resistance before 1990 to increasing prevalence of ciprofloxacin-resistant isolates beginning of the 21 century (from 55% in 2007 to 88% in 2017 [4,6]) and steady accumulation of genetic elements conferring resistance to other antibiotic classes in the early years of this decade [6–8]. A common explanation for the continuous selective pressure is the intensive use of antibiotics in livestock farming and human medicine. Constant globalization of trade and travel contribute further to the spread of resistant bacterial clones. In Europe, human infections with multidrug-resistant *S*. Kentucky ST198 used to be predominantly associated with travel to North Africa or South-East Asia, with especially Egypt being cited as the most probable source of these bacterial isolates [6,7,9].

The accumulation of fluoroquinolones resistance in *S*. Kentucky ST198 has been driven by mutations in the *gyrA* and *parC* genes [9], but also by lateral transfer of genetic elements, such as plasmids, and insertion of parts of these elements in the bacterial chromosome by recombination [10,11]. Previous studies indicate the circulation in Europe of *S*. Kentucky isolates with a configuration of three ciprofloxacin-resistance inducing mutations: Ser83Phe and Asp87Asn/Asp87Tyr in *gyrA*, and Ser80Ile in *parC* [6,7,9]. Additional chromosomal resistance genes were found inserted in the bacterial genome at various positions in the *Salmonella* Genomic Island 1 (SGI1) by mediation of insertion sequence 26 (IS26). Occasionally, genes encoding carbapenemases, e.g. *bla*_OXA-48_, *bla*_NDM-1_, and extended-spectrum β-lactamases (ESBLs) were found e.g. *bla*_CTX-M-1_, particularly on IncC and IncI1 plasmids [9].

Recent years have brought about a further increase in the incidence of cases with *S*. Kentucky ST198 acquired within Europe harbouring both multidrug-resistance (77% in 2018) and high-level ciprofloxacin resistance (89% in 2018) [12,13]. A few countries have reported cases of *S*. Kentucky with ESBL to ECDC in the routine surveillance data for 2016 [13]. This triggered further investigations which led to the discovery of an emergent *S*. Kentucky clone in Europe – one that next to ciprofloxacin resistance also harbours *bla*_CTX-M-14b_, a type of ESBL gene that is new for this serovar. This study aimed at investigating the epidemiology, phylogenomics, and comparative genomics of this new ESBL-producing clone of *S*. Kentucky ST198. By analysing a set of clinical isolates and putting them in the broader context of internationally collected isolates, we try to trace back the origins of this emerging clone and highlight the threat it might pose in the future for public health.

## Material and methods

### Epidemiological data

Epidemiological information on cases with *S*. Kentucky *bla*_CTX-M-14b_ was collected from EU/EEA countries via an “urgent inquiry” launched in the ECDC Epidemic Intelligence Information System for Food- and Waterborne Diseases and Zoonoses (EPIS-FWD) in March 2018. Nine EU/EEA countries had observed such cases in recent years, and of these, eight could participate in the study. Data were collected on age, gender, travel, specimen type and date of receipt of the isolate at the reference laboratory. The Fisher exact test was used to compare frequencies in gender, age groups and specimen types of cases with *S*. Kentucky *bla*_CTX-M-14b_ with those of other *S*. Kentucky and of other non-typhoidal *Salmonella* reported with AMR data to the European Surveillance System (TESSy) at ECDC for the years 2013–2018. A significance level of 0.05 was applied.

### Sequence dataset

Sequences from 78 clinical isolates and one food isolate of *S*. Kentucky *bla*_CTX-M-14b_ as well as from 35 other *S*. Kentucky ST198 circulating in eight participant countries, were collected at ECDC. These 114 sequences were supplemented with 166 other *S*. Kentucky ST198 from various public sequence databases to provide context. These were selected based on a maximum 50 cgMLST loci dissimilarity with the *bla*_CTX-M-14b_ isolates, using the Enterobase cgMLST schema and filtering options [14]. The resultant dataset contained a total of 280 sequences, spanning 17 years (Table S1).

### Bacterial isolates processing

The sequencing of the *Salmonella* isolates was performed on various Illumina platforms (Illumina, San Diego, CA, U.S.A.), i.e. HiSeq 2000, HiSeq 2500, and MiSeq PE300 with the appropriate Illumina library protocols. Paired-end FASTQ files were quality trimmed and *de novo* genome assemblies were made using CLC Genomics Workbench v 10.0 (Qiagen, Hilden, Germany). The parameters for trimming were as follows; ambiguous limit: 3, quality limit: 0.05. The parameters for the *de novo* assembly were as follows. For mapping settings; mapping mode: map reads back to contigs (slow), update contigs selected, mismatch cost: 2, insertion cost: 3, deletion cost: 3, length fraction: 0.5, similarity fraction: 0.8, match mode: random. For de novo settings; bubble size: 50, word size: 20, minimum contig length: 200 bp, perform scaffolding selected; auto-detect paired distances selected.

For identification of single nucleotide polymorphisms (SNPs) the short paired-end reads were mapped to a reference genome of *S*. Kentucky (GenBank accession number CP026327) using BWA-MEM [15]. SAM files from this aligner were sorted and filtered into BAM files using SAMtools [16]. The VCF files with high quality SNPs (>90% consensus, minimum depth 15x, GQ > = 20) were created using BCFtools [16]. The SNPs present in regions potentially resulting from recombination processes were further identified using Gubbins v1 [17] with default settings and subsequently removed.

For several isolates (S16BD08730, S18BD00684, S18BD03994, and S18BD05011), additional long-sequence reads were obtained at Sciensano with a MinION sequencer (Oxford Nanopore Technology), in view of a hybrid assembly. The respective isolates were considered to be good representatives of the diversity of *S*. Kentucky observed in our dataset. The long-read sequencing library was prepared with the 1D native barcoding genomic DNA (with EXP-NBD103 and SQK-LSK108 kits) protocol from Oxford Nanopore Technologies. The long-read sequencing library pool consisting of 12 barcodes (including other samples not relevant for this study) was loaded onto a 9.4 MinION flowcell (FLO-MIN106) and sequenced for 48 h. Local basecalling was performed with Guppy (v3.2.4) with the trimming option enabled that removes low quality bases before the adapter sequence. For the hybrid assembly the same software and settings as described previously [18] were used. The hybrid assemblies were used to verify the genomic context of the *bla*_CTX-M_ alleles.

### Annotation

The overall gene content and position of genes on the genomic contigs of the isolates was assessed using the annotation tool Prokka v1 [19], with CP026327 as reference genome. Alignment of genomic regions was performed using the BLAST feature of Easyfig [20].

### Genome mining

Genome assemblies were screened with BLAST+ v.2.9.0 [21,22] for the presence of plasmid replicons, antibiotic resistance genes, pathogenicity islands, and insertion sequences, using the PlasmidFinder [23], ResFinder [24], PAI [25,26], and ISfinder [27,28] databases (accessed at 30 November 2018), respectively. The thresholds for considering one of the genetic features present were 60% coverage and 90% identity with the target.

### Phylogenetics and phylodynamics

For the phylogenetic analysis, we used only the SNPs present in the homologous regions of the genome. BEAST v.2.5.0 [29] was used for the time calibrated Bayesian inference of phylogeny, where the calibrator was the sampling year of the isolates. The substitution model best fitting the data was selected to be TVM + Gamma, as assessed in jModelTest2 [30,31]. Due to a low temporal signal in the data (R2 of the heuristic residual mean squared = 0.1541), as inferred by the root-to-tip regression in TempEst v.1.5.1 [32], a log-normal uncorrelated relaxed molecular clock was used. The choice for a specific demographic model was based on the number of population changes from the Extended Bayesian Skyline Plot analysis, with 10^8^ steps and sampling every 5000 steps. Since the 95%HPD did not contain 0, indicating at least one change in the bacterial population size, we used a Bayesian Skyline demographic model, which was run three times, each with 1.5 × 10^8^ steps and sampling every 10,000 steps. The maximum clade credibility tree was based on 34,000 trees.

Estimates of the demographic changes in the bacterial population were calculated for both the entire dataset and the *bla*_CTX-M-14b_ clone using Tajima's D [28] as implemented in the “pegas” package v.0.11 [33] in R v.3.6.0 [34].

The most likely country of origin for the various clades in the final BEAST phylogenetic tree was inferred using maximum likelihood estimation of discrete ancestral states with an equal rates model for state transition in the “ape” package v.5.3 [35] in R.

## Results

We have analysed European clinical isolates belonging to *S*. Kentucky ST198 *bla*_CTX-M-14b_ for distribution, phylodynamics, and presence of molecular markers associated with antibiotic resistance. Next to the 78 CTX-M-14b human isolates, we have used one additional CTX-M-14b food isolate from peppermint imported from Egypt (provided by Public Health England), and 201 other *S*. Kentucky ST198 isolates collected from either countries participating in this inquiry (n = 35), or the public domain (n = 166) in order to put the findings into the broader context of internationally circulating bacteria, and to reliably reconstruct the phylogeny. Details on all isolates included in the study can be found in the supplementary material (Table S1). For clarity, through the rest of the manuscript, we will refer to the 78 CTX-M-14b isolates as the clinical dataset, and to the total number of isolates used in the study (clinical, food and public databases) as the extended dataset.

### Epidemiology

**Figure 1.**
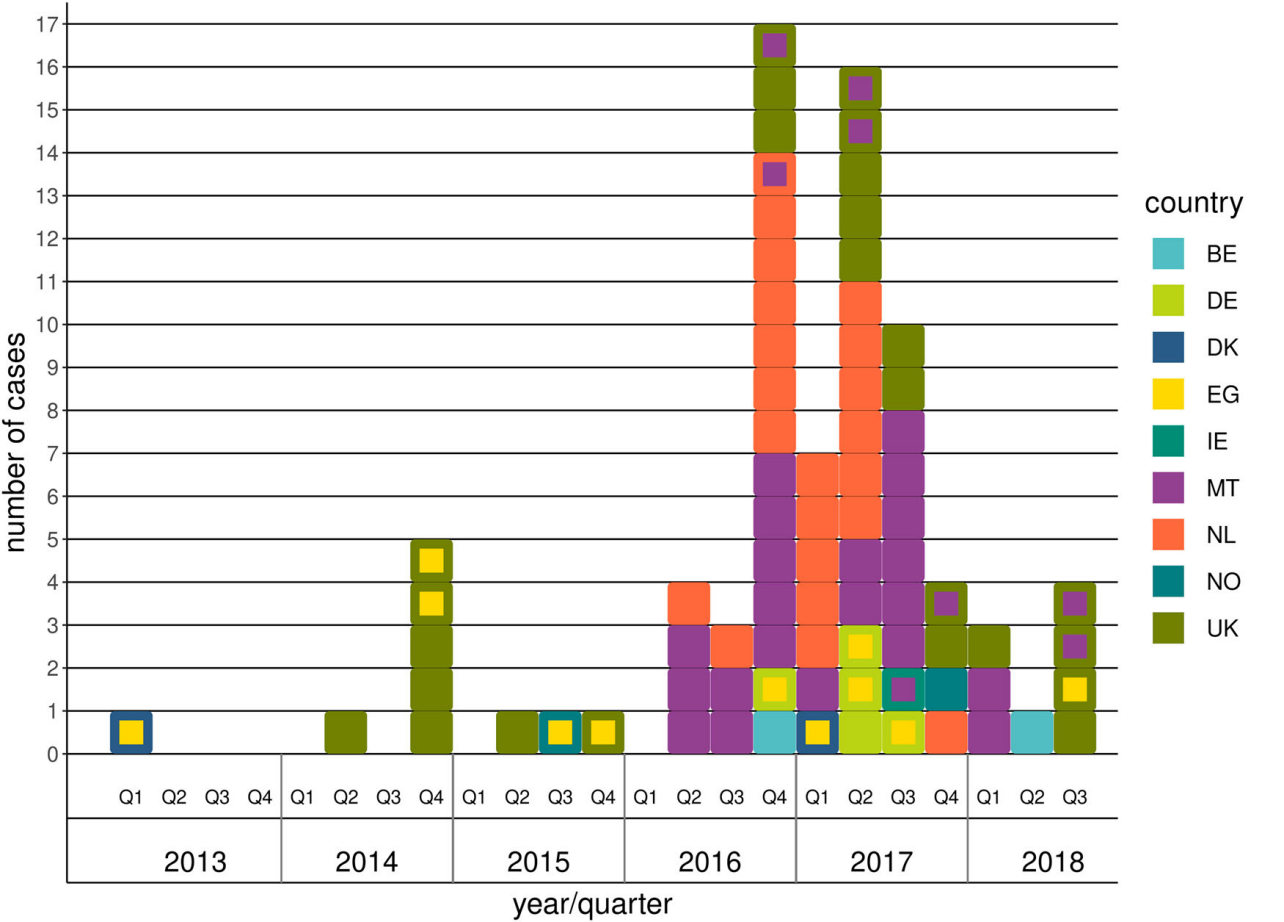
Distribution of *S*. Kentucky *bla*_CTX-M-14b_ cases by year and quarter. Each square in the figure represents one case; the outer colour indicates the country where the case was reported, while the inner colour indicates the most probable country of infection when the case had travelled during the incubation period.

Of the 78 *S*. Kentucky *bla*_CTX-M-14b_ clinical cases included in the study, 24 were from the United Kingdom, 21 from Malta, 21 from the Netherlands, five from Germany, two each from Belgium, Denmark, and Norway and one from Ireland. The first case was identified in February 2013 and the last case in August 2018 (Figure 1). Twenty cases had reported travel within the incubation period of which 11 to Egypt, eight to Malta and one with unknown destination. Forty-five cases (58%) were male and 33 (42%) female. The proportion of males among cases with *S*. Kentucky *bla*_CTX-M-14b_ was not significantly different than those of other non-typhoidal *Salmonella* serovars, though higher than in other *S*. Kentucky infections (Table 1). The age ranged from 0 to 95 years with most cases (42%) in persons 65 years and older (Table 1). The proportion elderly (65+ years) was significantly higher among cases with *S*. Kentucky *bla*_CTX-M-14b_ in comparison to other *S*. Kentucky and other non-typhoidal *Salmonella* serovars (Table 1). Isolation of the bacteria from urine (14.1%) was also significantly more common among these cases compared to other *S*. Kentucky and other non-typhoidal *Salmonella* serovars (Table 1).

**Table 1.** Comparison of epidemiological data of the *S*. Kentucky CTX-M-14b with other *S*. Kentucky and non-typhoidal *Salmonella.*

	*S*. Kentucky CTX-M-14b (*n*=78; 100%)		Other *S*. Kentucky (*n*=1901; 100%)		Other non-typhoidal *Salmonella* (*n*=108,162; 100%)		CTX-M-14 vs other *S*. Kentucky (*p-value*)	CTX-M-14 vs other non-typhoidal *Salmonella* (*p-value*)
*Gender*								
Male	45	(57.7%)	845	(44.5%)	51,328	(47.5%)	0.050*	0.213
Female	33	(42.3%)	980	(51.6%)	51,248	(47.4%)	-	-
Unknown	0	(0.0%)	76	(4.0%)	5586	(5.2%)	-	-
*Age groups (years)*								
0–4	4	(3.8%)	256	(13.5%)	23,124	(21.4%)	0.006*	<0.001*
5–14	0	(0.0%)	72	(3.8%)	16,475	(15.2%)	0.072	<0.001*
15–24	11	(14.1%)	188	(9.9%)	10,754	(9.9%)	0.343	0.266
25–44	15	(19.2%)	425	(22.4%)	17,784	(16.4%)	0.494	0.652
45–64	17	(20.5%)	473	(24.9%)	18,901	(17.5%)	0.294	0.561
65+	33	(42.3%)	397	(20.9%)	16,310	(15.1%)	<0.001*	<0.001*
Unknown	0	(0.0%)	90	(4.7%)	4814	(4.5%)	-	-
*Specimen type*								
Faeces	61	(76.9%)	1530	(80.5%)	91,333	(84.4%)	0.023*	<0.001*
Urine	11	(14.1%)	123	(6.5%)	1460	(1.3%)	0.024*	<0.001*
Blood	1	(1.3%)	22	(1.2%)	2511	(2.3%)	1.000	1.000
Other	2	(3.8%)	41	(2.2%)	2912	(2.7%)	0.428	0.160
Unknown	3	(3.8%)	185	(9.7%)	9946	(9.2%)	-	-

* Significant at *p*≤0.05.

### Antibiotic resistance genes content

Genome mining for genes encoding for antimicrobial resistance indicated the presence of a wide spectrum of predicted resistance. Ten classes of antibiotics were included in the search list: aminoglycosides, β-lactams, fosfomycin, macrolides/lincosamides/streptogramin (MLS), phenicol, quinolones, rifampicin, sulphonamides, tetracycline, and trimethoprim. Resistance genes from all ten classes were found in the extended dataset (280 isolates), while in the clinical CTX-M-14b isolates (78 isolates) resistance genes from seven classes were found: aminoglycosides, β-lactams, fosfomycin, phenicol, sulphonamides, tetracycline, and trimethoprim. The majority of these latter isolates (74/78, 94.8%) presented resistance genes from four classes of antibiotics – aminoglycosides, β-lactams, sulphonamides, and tetracycline. Resistance to fosfomycin, phenicol, and trimethoprim was sporadic (Table S1). All 280 isolates harboured one or more aminoglycoside resistance gene, with 56.8% (159/280) of all isolates and 97.4% (76/78) of the CTX-M-14b isolates harbouring four or more aminoglycoside resistance genes, and 30.4% (85/280) of all isolates and 89.7% (70/78) of the CTX-M-14b isolates harbouring six or seven of these determinants (Table S1). The majority of them were associated with SGI1, as described previously [9] i.e. *aac(3)-Id*, *aadA7*, *aph(3″)-Ib*, *aph(3’)-Ia*, and *aph(6)-Id*. The chromosomally located *aac(6’)-Iaa*, was present in all isolates.

The *sul1* and *tet(A)* genes were also present in most of the CTX-M-14b isolates (94.8% or 78/78 and 96.1% or 75/78, respectively). Among all isolates investigated, these two genes were slightly less prevalent (80.0% or 224/280 and 86.8% or 243/280, respectively). Three isolates carried also other β-lactamase encoding genes: non-ESBL *bla*_TEM_ variant (1.3% or 1/78), and carbapenemase *bla*_OXA-48_ (2.56% or 2/78; Table S1). In the extended dataset, the *bla*_TEM_ gene was present in 51.8% (145/280) of the isolates, with a majority of *bla*_TEM-1B_ (144/280), and one instance of *bla*_TEM-1C_ (1/280), while *bla*_OXA-48_ was only demonstrated in the CTX-M-14b isolates (Table S1). The peppermint isolate presented a similar antibiotic resistance pattern to the clinical *S*. Kentucky *bla*_CTX-M-14b_ isolates (Table S1).

Genes *qnrB4* and *qnr*B19, conferring resistance to quinolones were present in only four isolates from the extended dataset (1.43% or 4/280), but not among the CTX-M-14b isolates. Point mutations involved in the development of ciprofloxacin resistance were identified in the *gyrA* and *parC* genes. The CTX-M-14b clinical isolates presented the nonsynonymous mutations Ser80Ile in ParC, and Asp87Gly in GyrA. Other isolates in the extended dataset presented also Asp87Asn (84 isolates), and Asp87Tyr (53 isolates) in GyrA (Align S1, Align S2). All clinical CTX-M-14b isolates were resistant to ciprofloxacin. From the extended dataset, only five were susceptible to ciprofloxacin (5/280 or 1.78%); these were isolated between 2001 and 2005, and presented the wildtype aminoacids in positions 87 of GyrA (D) and 80 of ParC (S).

The *in silico* characterization of the antibiotic resistance profile identified the *S*. Kentucky CTX-M-14b as a set of MDR isolates with molecular markers encoding for resistance to aminoglycosides, fluoroquinolones, β-lactams, sulphonamides, and tetracycline.

### Plasmid replicon content

We have used the detection of plasmid replicons (short fragments of plasmid DNA responsible for the initiation and control of plasmid replication) to infer presence of certain plasmids in the bacterial isolates (as described in [36]) i.e. we assume that if the replicon specific for a certain plasmid class is detected, the plasmid will be present in its entirety. The plasmid replicon content analysis revealed a wide range of plasmids in the extended dataset, with several small plasmids and multiple different Inc families encountered, of which Col(pVC) and IncI1 were the most abundant (22/280 or 7.86% and 10/280 or 3.57% isolates, respectively). The distribution of the plasmids in the clinical CTX-M-14b isolates reflected also the distribution of the plasmids in the extended dataset (Table S1). Many of the isolates (31/280 or 11.07%) contained one plasmid only. Two and more plasmid types were found in seven isolates (7/280 or 2.5%; Table S1).

### Phylogenetics and phylodynamics

**Figure 2.**
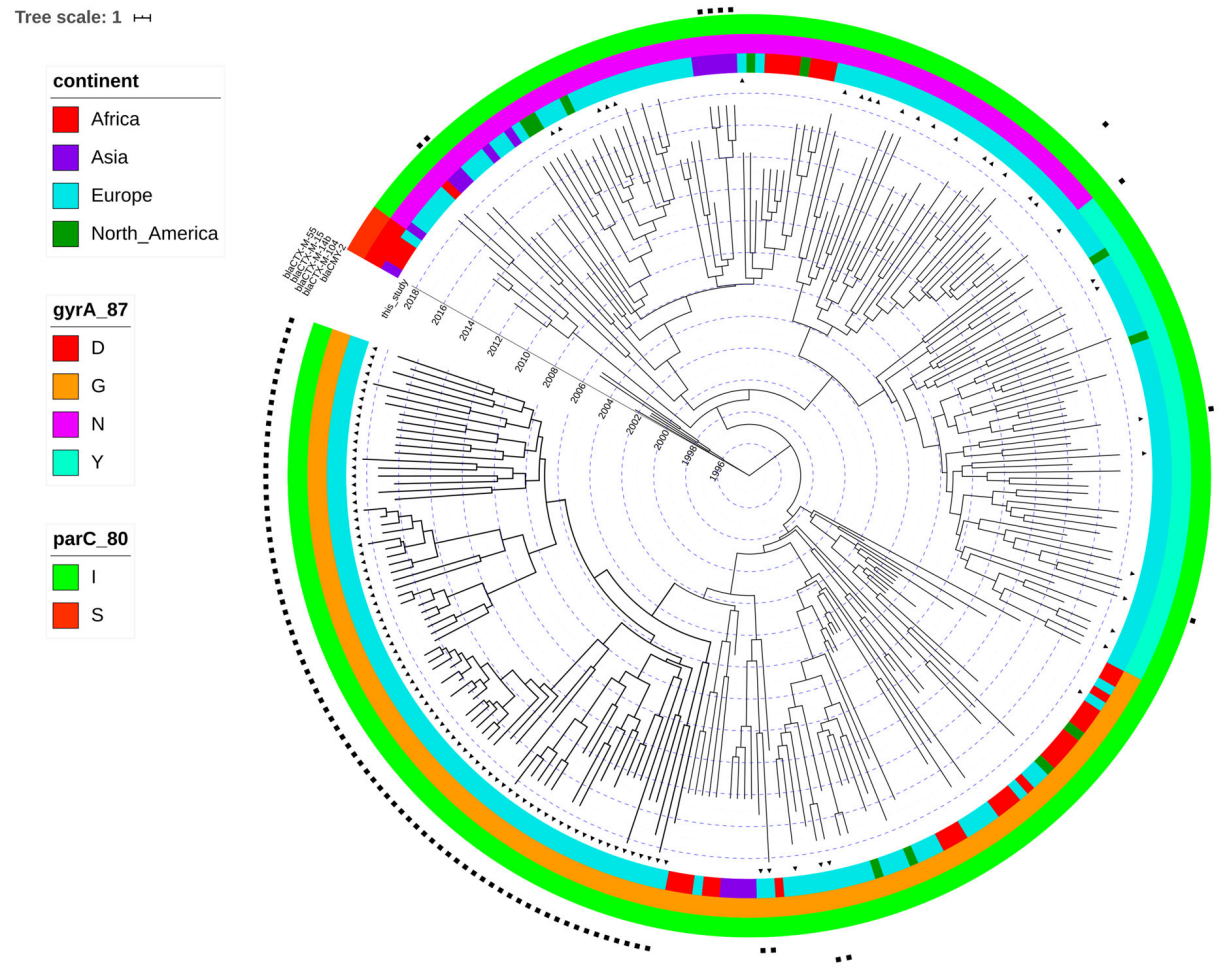
Time-scaled phylogeny of *S*. Kentucky ST198. The coloured concentric rings represent, from the center outwards, the continent where the isolate was collected, the amino acid at position 87 in GyrA, and the amino acid at position 80 in ParC. Ciprofloxacin sensitive isolates are characterized by presence of D at position 87 of GyrA and of S at position 80 of ParC. The presence of black triangles immediately next to the tips of the tree indicate isolates that were collected from European countries participating in this inquiry. The squares in multiple concentric rings indicate the presence of a specific ESBL gene/allele (from the center outwards): *bla*_CMY-2_, *bla*_CTX-M-104_, *bla*_CTX-M14b_, *bla*_CTX-M-15_, and *bla*_CTX-M-55_.

**Figure 3.**
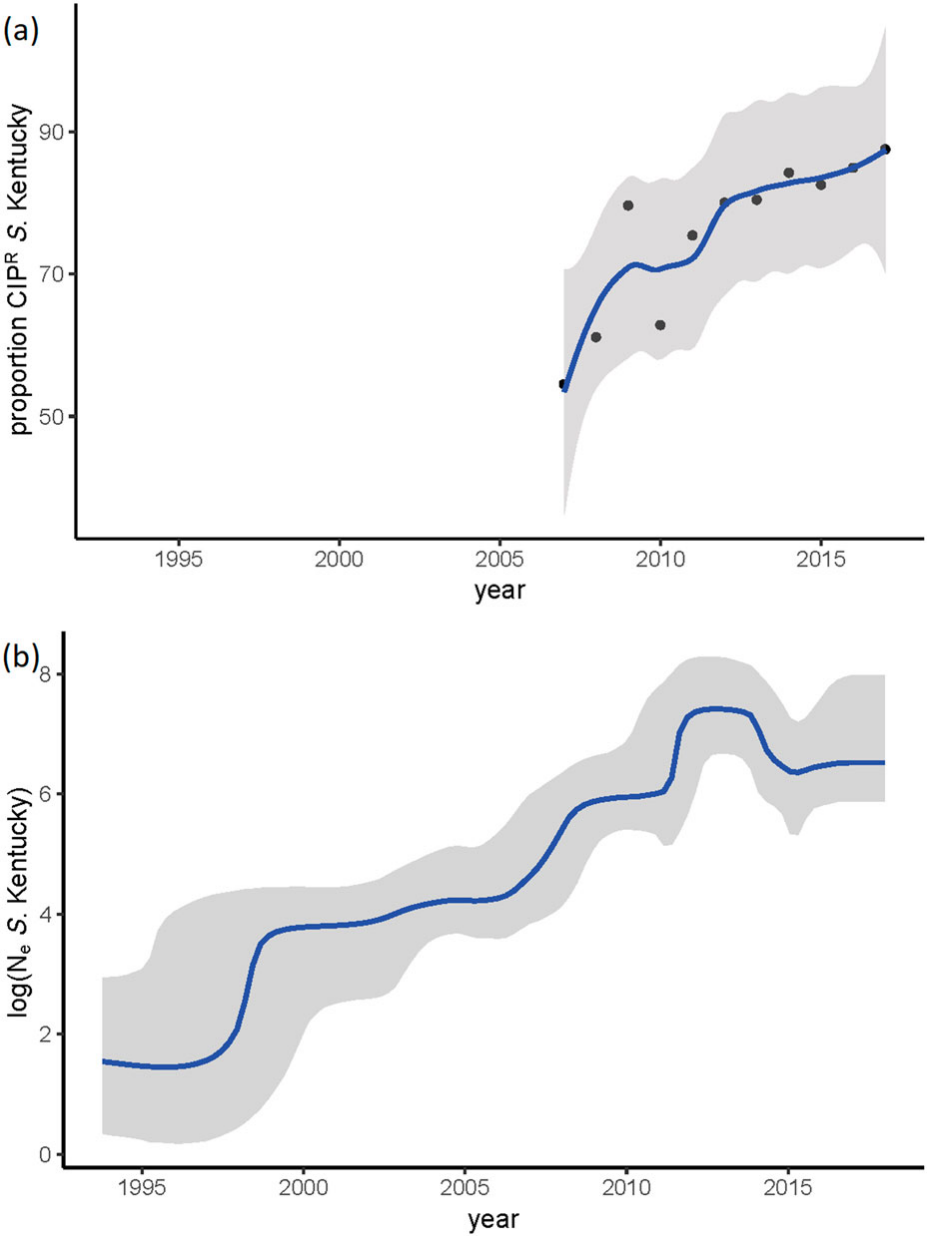
Temporal trends in *S*. Kentucky. (a) y-axis indicates the proportion of human cases with ciprofloxacin-resistant *S*. Kentucky among all *S*. Kentucky infections – data from ECDC; (b) Bayesian skyline plot of the extended dataset of *S*. Kentucky ST 198; y axis indicates the effective population size on a log10 scale.

After extraction of the core genome SNPs and filtering of the high frequency variants, potentially indicative of recombination, a total number of 2650 SNPs remained to be used in the phylogenetic inference. Two Dutch CTX-M-14b clinical isolates from this study were identical in their SNP profile and consequently, one of them was discarded, resulting in a phylogeny of 279 terminal nodes. Phylogenetic reconstruction of the extended dataset indicated the presence of three major clades, corresponding roughly to the amino acid substitutions in GyrA (Figure 2). The ciprofloxacin susceptible isolates clustered close to the root of the tree, which was estimated at ∼ 1993 (Figure 2). The origin of the ciprofloxacin resistance (CIP^R^) was dated to 1998 (95%HPD 1995-1999). The reconstruction of ancestral states of the nodes in the phylogeny indicates Egypt as the most probable origin of CIP^R^, followed by spread all over the world (Figure 2). Bayesian skyline analysis showed an increase in the effective population size of CIP^R^ *S*. Kentucky ST198 (Figure 3(b)), which is consistent with the statistics from TESSy on the increased prevalence of CIP^R^ in Europe (Figure 3(a)). Additionally, the negative Tajima's D (−2.72, *p* = 0.006) indicated a potential demographic expansion of CIP^R^ *S*. Kentucky ST198.

**Figure 4.**
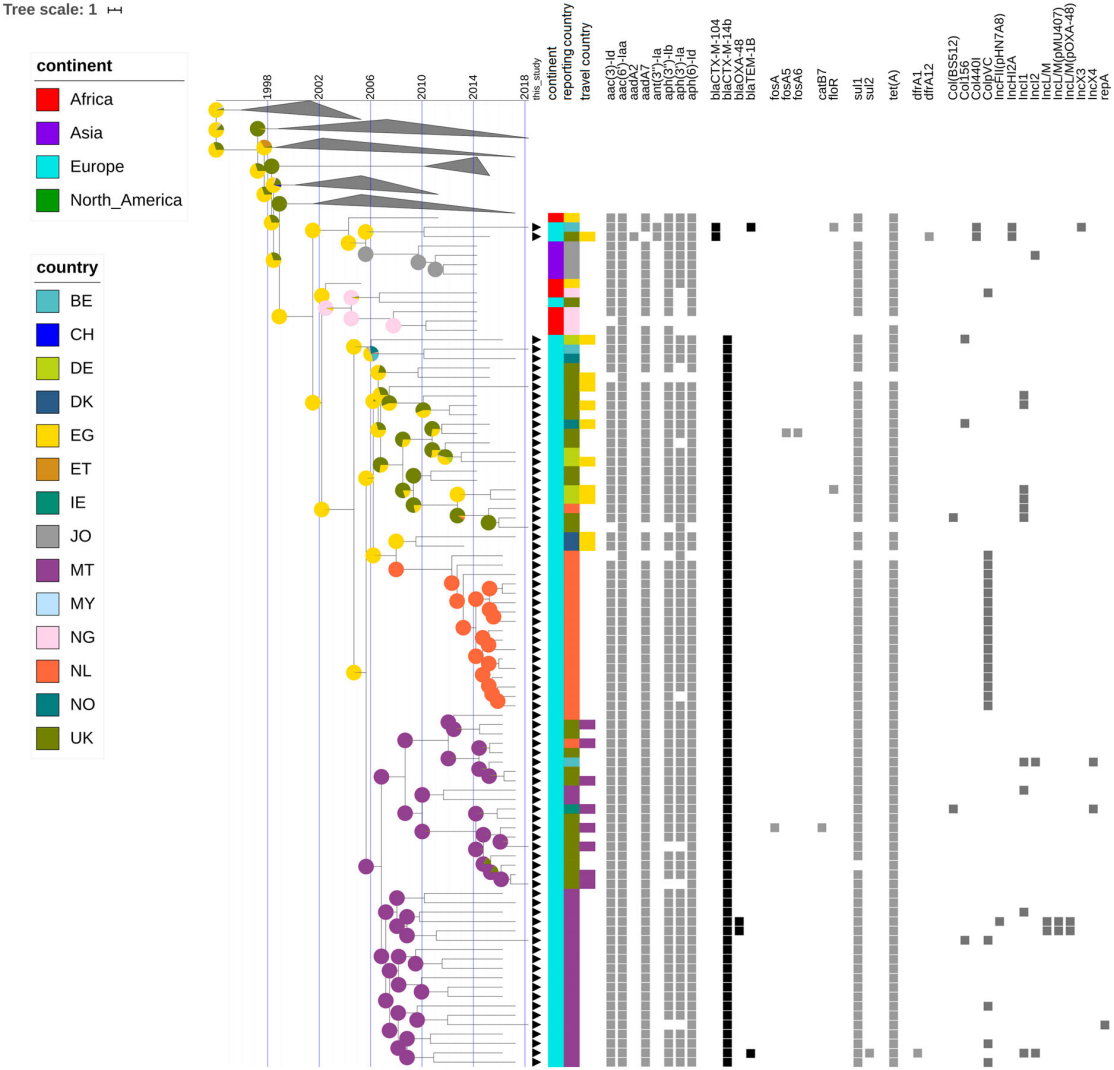
Time-scaled phylogeny with epidemiological and molecular features of the *S*. Kentucky *bla*_CTX-M-14b_ clade and closely related isolates. The presence of black triangles immediately next to the tips of the tree indicate isolates that were collected from European countries participating in this inquiry. The coloured strips indicate the continent, country of isolation, and country of infection for the respective isolates. The coloured squares indicate the presence of a specific antibiotic resistance gene or a plasmid. The pie charts associated with the internal nodes of the tree indicate the probability for the country of origin, with larger sectors of the circles corresponding to a higher probability for the country with the respective colour.

While resistance genes for cephalosporins are present in various branches of the phylogeny, the clone harbouring *bla*_CTX-M-14b_ was contained within the Asp87Gly (G) clade. The CTX-M-14b isolate from peppermint imported from Egypt clustered with the rest of the CTX-M14b isolates. The genetic distance between *bla*_CTX-M-14b_ and *bla*_CTX-M-104_, observed in a couple other isolates in an adjacent clade, is of only one point mutation – the adenine at position 824 is substituted by guanine (A824G), resulting in an amino acid change at codon 275 (Asn275Ser, Align S3, Align S4). Even with all additional isolates included in the analysis no human isolates carrying the *bla*_CTX-M-14b_ gene could be found elsewhere outside of Europe (Figure 2). However, isolates from ten *bla*_CTX-M-14b_ positive persons have been associated to travel to Egypt. Some of the first introductions to Europe were to Germany and Norway, followed by almost concomitant introductions to Malta, UK and other EU countries. No less than eight isolates seem to have been acquired during travel to a European country – Malta (Figure 4). The Bayesian reconstruction of phylogeny dated the most recent common ancestor (MRCA) of this clone to 2005 (95%HPD 2002-2006), and the reconstruction of the ancestral state indicates Egypt as its most likely country of origin (Figure 4). The *bla*_CTX-M-14b_ clade is structured in four clusters, rather homogeneous in terms of country of isolation. Remarkably, the isolates from the UK form two distinct clusters, heterogeneous in genetic diversity; one cluster is strongly associated with travel to Egypt, while the other one with travel to Malta (Figure 4). The Dutch isolates form a low diversity cluster, and they coalesce with two isolates from Germany with travel record to Egypt. The cluster of isolates from Malta shows a large genetic diversity, with long branches sharing a MRCA in about 2007. The Bayesian skyline plot did not indicate an increase in effective population size of the *bla*_CTX-M-14b_ clone. However, Tajima's D was < −2 (−2.38, *p* = 0.01), indicating a potential expansion of this clone.

## Genomic location of bla_CTX-M14b_

**Figure 5.**
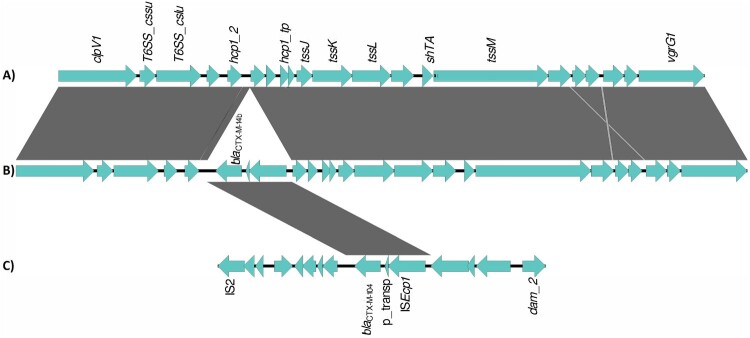
Alignment of genomic segments from the chromosome and plasmid of representative *S*. Kentucky ST198 isolates. (A) chromosome of *S*. Kentucky isolate lacking the chromosomal *bla*_CTX-M-14b_, (B) chromosome of *S*. Kentucky isolate carrying the chromosomal *bla*_CTX-M-14b_, (C) plasmid of *S*. Kentucky isolate carrying *bla*_CTX-M-104_. Genomic loci are depicted as blue arrows and homology regions are depicted as grey blocks. The comparison of the T6SS regions on the bacterial chromosome of a *bla*_CTX-M-14b_ negative isolate and a *bla*_CTX-M-14b_ positive isolate show the insertion of a fragment carrying the *bla*_CTX-M-14b_ gene, a putative transposase and the IS*ECp1* insertion sequence. This fragment is identical to a fragment located on the IncHIA2 plasmid of some *bla*_CTX-M-14b_ negative isolates.

The architecture of the antibiotic resistance complement was complex, with multiple locations in the bacterial genome harbouring antibiotic resistance genes. Most genes encoding for resistance to cephalosporins (*bla*_CTX-M_ and *bla*_CMY_) were located on plasmids, with the exception of *bla*_CTX-M-14b_ which was always located on the chromosome. Genome alignment of isolates carrying this gene, with isolates from neighbouring branches of the phylogeny, and with reference isolates, showed that *bla*_CTX-M-14b_ is present on a 2854 bp fragment inserted in the T6SS region of the bacterial genome, downstream of the *hcp1* gene (position 3701330 of reference genome CP126327). The fragment is flanked on the one side by an insertion sequence – IS*Ecp1* from the IS*1380* family of insertion sequences (Figure 5). The 2854 bp fragment was absent on the chromosome of the non-*bla*_CTX-M-14b_. A similar construct was, however, found on plasmids in isolates paraphyletic to the CTX-M-14b clade; the only difference was that the respective plasmids carried another *bla*_CTX-M_ allele - *bla*_CTX-M-104_, with one point mutation in comparison to *bla*_CTX-M-14b_ (Figure 5, Align S3, Align S4). Interestingly, one of the isolates carrying this ESBL gene on a plasmid had a travel record to Egypt.

## Discussion

At EU level, over the last years, the proportions of human *Salmonella* isolates resistant to the clinically important antimicrobials ciproﬂoxacin and cefotaxime were overall relatively low (12.5% and 1.5%, respectively in 2018)[12]. However, in *S*. Kentucky, the seventh most common serovar in human *Salmonella* infections in 2018, an extremely high proportion (88.6%) of the isolates were high-level ciproﬂoxacin resistant [12]. In addition, *S*. Kentucky shows relatively high levels of combined resistance to ciproﬂoxacin and cefotaxime. The highest proportion of *S*. Kentucky isolates with combined resistance was reported in Malta and related to ESBL-producing bacterial isolates [4]. This triggered the current study aiming at reconstructing the time and place of emergence of this strain, carrying *bla*_CTX-M-14b_, which seems to have evolved from the high-level ciproﬂoxacin resistant, multidrug-resistant *S*. Kentucky ST198. The latter has spread rapidly throughout Europe and elsewhere in the world, both in humans and in the food chain, and is considered to originally have been imported to Europe via travellers to North Africa [6]. To the date of completion of this analysis no ESBL-producing *S*. Kentucky carrying *bla*_CTX-M-14b_ have been reported in food or animals to the European Food Safety Agency or the EU Reference Laboratory for antimicrobial resistance [4].

The majority of European isolates of *S*. Kentucky ST198 used in this study contain multiple resistance genes to aminoglycosides. All of them contained mutations in the *gyrA* and *parC* genes, potentially involved in resistance to ciprofloxacin. Furthermore, ESBL-producing isolates in our dataset were not occasional, but amassed to more than 100 isolates, all collected in the past decade. Moreover, two of the CTX-M-14b isolates also contained a carbapenemase gene, i.e. *bla*_OXA-48_.

So far, *bla*_CTX-M_ genes in European *S*. Kentucky have always been described on plasmids. There have been several reports of sequential integration of antimicrobial resistance genes at specific locations in the chromosome, such as the SGI1 [7,9]. Here, we report the integration of *bla*_CTX-M-14b_ in the bacterial chromosome of *S*. Kentucky isolated from European patients. The similarity in structure of the inserted fragment with a corresponding fragment on the IncHI2 plasmid in isolates retrieved from other European countries, highlights the possibility that the integration has occurred as result of lateral gene transfer from this plasmid to the chromosome. One of the isolates carrying this fragment on the plasmid was isolated from a patient in the UK, but with a travel history to Egypt. Although the *bla*_CTX-M_ allele on the plasmid was a different one – *bla*_CTX-M-104_ – the difference between the two CTX-M alleles is one base substitution. This might suggest a two-step event that has led to the integration of *bla*_CTX-M-14b_ in the chromosome, with either order of the events possible: (a) point mutation leading to an amino acid substitution (Asn275Ser), and (b) integration in the chromosome, mediated by an IS element belonging to the IS*1380* family; IS elements are known to drive the expression of *bla*_CTX-M_ genes [37]. IS*Ecp1*-mediated integration events have also been described for *bla*_CMY_ genes in *Acinetobacter* spp. [38]. Alternatively, another source for the *bla*_CTX-M-14b_ might be lateral gene transfer with *Escherichia coli*. CTX-M-14b is highly prevalent in *E. coli* [39-41], and also present on IncHI2 plasmids [40], in the same genetic background (i.e. flanked by the same insertion sequence) as in our *S*. Kentucky isolates. Furthermore, the two bacterial species often share environmental and biological niches where the horizontal gene transfer could occur [42-44]. Recently, S. Kentucky with a chromosomally located *bla*_CTX-M-14b_ gene has been reported in China [45] in carcass washing water from a poultry slaughterhouse, and other events of integration of a *bla*_CTX-M_ gene in the bacterial chromosome have been reported in Chinese isolates of *S.* Chester*, S.* Indiana, and *S.* Typhimurium [46], converging to the scenario of *Salmonella* spp. evolving towards multidrug-resistant bacteria due to the increased use of antibiotics in intensive farming settings.

Travel history of the patients from which the *bla*_CTX-M-14b_ isolates have been retrieved indicate that this clone is prevalent in Egypt and that occasional infection of travellers occurs. The Dutch isolates forming a low diversity cluster were all isolated from an outbreak of salmonellosis in a retirement home, and they coalesced with isolates from people with travel history to Egypt. Additionally, the only non-human isolate containing *bla*_CTX-M-14b_ was retrieved from fresh peppermint imported from Egypt in 2015. Furthermore, the ancestral state reconstruction for the country of infection indicates Egypt as the most probable origin, not only for the *bla*_CTX-M-14b_ clone, but also for the entire CIP^R^ clone. What has once been a local problem has become of international concern, with the international travel of people and/or trade of food potentially playing a role in the dispersion of the MDR bacteria. Egypt and North Africa were also mentioned in previous research [7,47] as the origin of CIP^R^ *S*. Kentucky ST198 and commonplace for acquiring antibiotic resistance genes, and it is believed that intensive usage of antibiotics in poultry industry and animal husbandry is the cause for the development of multidrug-resistance in *Salmonella* spp. [6,46]. Furthermore, the high prevalence of the *bla*_CTX-M-14b_ clone in Malta, both indigenously and in people travelling to this country, coupled with the high genetic diversity within the Maltese isolates, indicates potential establishment of this clone in a European country. Contrary to previous studies, where the acquisition of various AMR plasmids remained an isolated event [9], here we report the clonal expansion of a bacterium carrying resistance determinants for aminoglycosides, ESBLs, quinolones, sulphonamides, and tetracycline. Classical population genetics metrics showed a significant bacterial population expansion of this clone. The failure to detect the increase of bacterial population size using a time-scaled Bayesian approach might be related to the lack of temporal signal, as the isolates were collected over a relatively short time (2013-2018).

We hypothesize that one of the factors leading to the success of the *bla*_CTX-M-14b_ clone is the integration of the ESBL resistance gene in the bacterial chromosome. Although plasmid-mediated antibiotic resistance is an undesired situation it is of less consequence than the chromosomal-mediated one. Plasmids can be acquired, but also lost in bacterial evolution; the reduction of antibiotic use that would reduce the selective pressure on bacteria might arguably lead to elimination of plasmids from the population [48,49]. The ecological implication of the chromosomal-mediated antibiotic resistance is much more severe, as it does not allow the reversibility of the phenomenon – once the genes are integrated in the chromosome they are most likely to be maintained indefinitely, especially if they incur no fitness cost for the bacteria.

Although the main transmission mode of *Salmonella* spp. is zoonotic, commonly via contaminated food, the scarcity of non-human *S*. Kentucky *bla*_CTX-M-14b_ isolates makes it difficult to assess what is its main source of transmission. While the main host of *S*. Kentucky is poultry, to the date when this analysis was performed, there had been no ESBL producing *S*. Kentucky isolated from poultry in European countries [4]. The one non-human *bla*_CTX-M-14b_ *S*. Kentucky isolate, identified in the UK, came from peppermint imported from Egypt, and it suggests a spill over of bacteria in the environment – from where they might end up on various greens via, e.g. irrigation water. A recent publication of ECDC and EFSA revealed that in 2018 there were six reports of cefotaxime resistant *S*. Kentucky isolates in poultry in three European countries: one from laying hens in Hungary, and five from broilers in the Netherlands and Malta (one and four respectively)[12]. While all five broiler isolates were presumptive ESBL-producers, the genotypes and the genomic sequences are not known; we can therefore not make any allegations on the circulation or persistence of CTX-M-14b in poultry in Europe.

In 2016 and 2017, the cases from the Netherlands and Malta accounted for 2/3 of all *S*. Kentucky CTX-M-14b human cases. The vast majority of Dutch cases were associated with an outbreak in a retirement home while the Maltese cases were sporadic. In Malta, most of the elderly cases and one young adult had severe underlying comorbidities. Two Maltese cases developed symptoms while in hospital. Most cases were treated with antimicrobials until symptoms stopped, though a few became carriers over several months with repeated antimicrobial treatment. The significance of the high proportion of elderly and of urinary-tract infections among cases with *S.* Kentucky *bla*_CTX-M-14b_ compared to other infections with *S*. Kentucky or other non-typhoidal *Salmonella* is unclear. It could indicate that the bacterium is more widespread in the community, with symptomatic cases being identified mainly among elderly and/or persons with immunosuppression/underlying disease. It could, however, indicate nosocomial transmission, i.e. transmission occurring in a hospital or health-care setting, where the antimicrobial pressure is high and where resistance genes could be shared between different bacterial species within the *Enterobacteriaceae* via horizontal gene transfer within and/or between patients [50,51]. For *Salmonella*, such transfer has been suggested for the finding of OXA-48 in MDR *S*. Kentucky in North Africa [52]. Recently, a source attribution study regarding community-acquired ESBL-producing *E. coli* revealed humans as the most important source [53]. Similarly, diarrhoea-causing infections with Shiga toxin-producing *E. coli* carrying the *stx_2f_* variant are hypothesized to have originated from a human reservoir [54].

In this line, of increased recognition of humans as reservoir for pathogens previously thought to be strictly zoonotic, it could be hypothesized that *S.* Kentucky CTX-M-14b spreads from humans to humans. Hygiene and responsible use of antibiotics in health care and veterinary medicine remain important pillars of prevention. Since new cases of human infections with S. Kentucky CTX-M-14b continued being reported to national health authorities across Europe after the compilation of the dataset for this analysis, it seems that this clone is persistent in at least some parts of Europe. Investigating whether the persistence is driven by long term carriage of the bacteria in humans, or maintenance in animal reservoirs, requires continuous monitoring in both human and non-human populations in a One Health fashion. Further monitoring of temporal and spatial trends in the incidence of this clone, might assist in identification of potential non-human reservoirs, and possibly prevent establishment of multidrug-resistant bacteria in new geographical regions.

## Supplementary Material

Suppelemental_files.zip

## Data Availability

The accession numbers of the WGS for the isolates included in this study can be found in Table S1.
