# Supplementary material for: Genomic epidemiology of emerging ESBL-producing Salmonella Kentucky blaCTX-M-14b in Europe
Source: Emerg Microbes Infect. 2020 Sep 30;9(1):2124–35. doi: 10.1080/22221751.2020.1821582 (PMC7580578; doi:10.1080/22221751.2020.1821582)
Supplement: Suppelemental_files.zip [file TEMI_A_1821582_SM9137.zip › AlignS1.docx]

| AlignS1 |  |
| --- | --- |
| CLUSTAL W (1.7) multiple sequence alignment | |
|  |  |
|  |  |
| S16BD08730 | MSDMAERLALHEFTENAYLNYSMYVIMDRALPFIGDGLKPVQRRIVYAMSELGLNASAKF |
| S18BD00684 | MSDMAERLALHEFTENAYLNYSMYVIMDRALPFIGDGLKPVQRRIVYAMSELGLNASAKF |
| S18BD03994 | MSDMAERLALHEFTENAYLNYSMYVIMDRALPFIGDGLKPVQRRIVYAMSELGLNASAKF |
| S18BD05011 | MSDMAERLALHEFTENAYLNYSMYVIMDRALPFIGDGLKPVQRRIVYAMSELGLNASAKF |
| RKI_16-03723 | MSDMAERLALHEFTENAYLNYSMYVIMDRALPFIGDGLKPVQRRIVYAMSELGLNASAKF |
| RKI_16-04315 | MSDMAERLALHEFTENAYLNYSMYVIMDRALPFIGDGLKPVQRRIVYAMSELGLNASAKF |
| RKI_17-02304 | MSDMAERLALHEFTENAYLNYSMYVIMDRALPFIGDGLKPVQRRIVYAMSELGLNASAKF |
| RKI_17-02411 | MSDMAERLALHEFTENAYLNYSMYVIMDRALPFIGDGLKPVQRRIVYAMSELGLNASAKF |
| RKI_17-02757 | MSDMAERLALHEFTENAYLNYSMYVIMDRALPFIGDGLKPVQRRIVYAMSELGLNASAKF |
| RKI_17-04797 | MSDMAERLALHEFTENAYLNYSMYVIMDRALPFIGDGLKPVQRRIVYAMSELGLNASAKF |
| RKI_17-06869 | MSDMAERLALHEFTENAYLNYSMYVIMDRALPFIGDGLKPVQRRIVYAMSELGLNASAKF |
| ERR2580277 | MSDMAERLALHEFTENAYLNYSMYVIMDRALPFIGDGLKPVQRRIVYAMSELGLNASAKF |
| ERR2580276 | MSDMAERLALHEFTENAYLNYSMYVIMDRALPFIGDGLKPVQRRIVYAMSELGLNASAKF |
| ERR2580273 | MSDMAERLALHEFTENAYLNYSMYVIMDRALPFIGDGLKPVQRRIVYAMSELGLNASAKF |
| ERR2580274 | MSDMAERLALHEFTENAYLNYSMYVIMDRALPFIGDGLKPVQRRIVYAMSELGLNASAKF |
| ERR2173656 | MSDMAERLALHEFTENAYLNYSMYVIMDRALPFIGDGLKPVQRRIVYAMSELGLNASAKF |
| 17041676 | MSDMAERLALHEFTENAYLNYSMYVIMDRALPFIGDGLKPVQRRIVYAMSELGLNASAKF |
| MT16-000061 | MSDMAERLALHEFTENAYLNYSMYVIMDRALPFIGDGLKPVQRRIVYAMSELGLNASAKF |
| MT16-019416 | MSDMAERLALHEFTENAYLNYSMYVIMDRALPFIGDGLKPVQRRIVYAMSELGLNASAKF |
| MT16-027865 | MSDMAERLALHEFTENAYLNYSMYVIMDRALPFIGDGLKPVQRRIVYAMSELGLNASAKF |
| MT16-031693 | MSDMAERLALHEFTENAYLNYSMYVIMDRALPFIGDGLKPVQRRIVYAMSELGLNASAKF |
| MT16-040253 | MSDMAERLALHEFTENAYLNYSMYVIMDRALPFIGDGLKPVQRRIVYAMSELGLNASAKF |
| MT16-045379 | MSDMAERLALHEFTENAYLNYSMYVIMDRALPFIGDGLKPVQRRIVYAMSELGLNASAKF |
| MT16-442728 | MSDMAERLALHEFTENAYLNYSMYVIMDRALPFIGDGLKPVQRRIVYAMSELGLNASAKF |
| MT16-462857 | MSDMAERLALHEFTENAYLNYSMYVIMDRALPFIGDGLKPVQRRIVYAMSELGLNASAKF |
| MT16-480196 | MSDMAERLALHEFTENAYLNYSMYVIMDRALPFIGDGLKPVQRRIVYAMSELGLNASAKF |
| MT16-861555 | MSDMAERLALHEFTENAYLNYSMYVIMDRALPFIGDGLKPVQRRIVYAMSELGLNASAKF |
| MT17-076833 | MSDMAERLALHEFTENAYLNYSMYVIMDRALPFIGDGLKPVQRRIVYAMSELGLNASAKF |
| MT17-110677 | MSDMAERLALHEFTENAYLNYSMYVIMDRALPFIGDGLKPVQRRIVYAMSELGLNASAKF |
| MT17-131730 | MSDMAERLALHEFTENAYLNYSMYVIMDRALPFIGDGLKPVQRRIVYAMSELGLNASAKF |
| MT17-140890 | MSDMAERLALHEFTENAYLNYSMYVIMDRALPFIGDGLKPVQRRIVYAMSELGLNASAKF |
| MT17-141840 | MSDMAERLALHEFTENAYLNYSMYVIMDRALPFIGDGLKPVQRRIVYAMSELGLNASAKF |
| MT17-152488 | MSDMAERLALHEFTENAYLNYSMYVIMDRALPFIGDGLKPVQRRIVYAMSELGLNASAKF |
| MT17-157311 | MSDMAERLALHEFTENAYLNYSMYVIMDRALPFIGDGLKPVQRRIVYAMSELGLNASAKF |
| MT17-161645 | MSDMAERLALHEFTENAYLNYSMYVIMDRALPFIGDGLKPVQRRIVYAMSELGLNASAKF |
| MT17-167951 | MSDMAERLALHEFTENAYLNYSMYVIMDRALPFIGDGLKPVQRRIVYAMSELGLNASAKF |
| MT18-217732 | MSDMAERLALHEFTENAYLNYSMYVIMDRALPFIGDGLKPVQRRIVYAMSELGLNASAKF |
| MT18-252580 | MSDMAERLALHEFTENAYLNYSMYVIMDRALPFIGDGLKPVQRRIVYAMSELGLNASAKF |
| RIVM_H_2009-01 | MSDMAERLALHEFTENAYLNYSMYVIMDRALPFIGDGLKPVQRRIVYAMSELGLNASAKF |
| RIVM_H_2010-01 | MSDMAERLALHEFTENAYLNYSMYVIMDRALPFIGDGLKPVQRRIVYAMSELGLNASAKF |
| RIVM_H_2010-02 | MSDMAERLALHEFTENAYLNYSMYVIMDRALPFIGDGLKPVQRRIVYAMSELGLNASAKF |
| RIVM_H_2011-01 | MSDMAERLALHEFTENAYLNYSMYVIMDRALPFIGDGLKPVQRRIVYAMSELGLNASAKF |
| RIVM_H_2011-02 | MSDMAERLALHEFTENAYLNYSMYVIMDRALPFIGDGLKPVQRRIVYAMSELGLNASAKF |
| RIVM_H_2011-03 | MSDMAERLALHEFTENAYLNYSMYVIMDRALPFIGDGLKPVQRRIVYAMSELGLNASAKF |
| RIVM_H_2013-01 | MSDMAERLALHEFTENAYLNYSMYVIMDRALPFIGDGLKPVQRRIVYAMSELGLNASAKF |
| RIVM_H_2013-02 | MSDMAERLALHEFTENAYLNYSMYVIMDRALPFIGDGLKPVQRRIVYAMSELGLNASAKF |
| RIVM_H_2014-01 | MSDMAERLALHEFTENAYLNYSMYVIMDRALPFIGDGLKPVQRRIVYAMSELGLNASAKF |
| RIVM_H_2014-02 | MSDMAERLALHEFTENAYLNYSMYVIMDRALPFIGDGLKPVQRRIVYAMSELGLNASAKF |
| RIVM_H_2016-01 | MSDMAERLALHEFTENAYLNYSMYVIMDRALPFIGDGLKPVQRRIVYAMSELGLNASAKF |
| RIVM_H_2016-02 | MSDMAERLALHEFTENAYLNYSMYVIMDRALPFIGDGLKPVQRRIVYAMSELGLNASAKF |
| RIVM_H_2016-03 | MSDMAERLALHEFTENAYLNYSMYVIMDRALPFIGDGLKPVQRRIVYAMSELGLNASAKF |
| RIVM_H_2016-04 | MSDMAERLALHEFTENAYLNYSMYVIMDRALPFIGDGLKPVQRRIVYAMSELGLNASAKF |
| RIVM_H_2016-05 | MSDMAERLALHEFTENAYLNYSMYVIMDRALPFIGDGLKPVQRRIVYAMSELGLNASAKF |
| RIVM_H_2016-06 | MSDMAERLALHEFTENAYLNYSMYVIMDRALPFIGDGLKPVQRRIVYAMSELGLNASAKF |
| RIVM_H_2016-07 | MSDMAERLALHEFTENAYLNYSMYVIMDRALPFIGDGLKPVQRRIVYAMSELGLNASAKF |
| RIVM_H_2016-08 | MSDMAERLALHEFTENAYLNYSMYVIMDRALPFIGDGLKPVQRRIVYAMSELGLNASAKF |
| RIVM_H_2016-09 | MSDMAERLALHEFTENAYLNYSMYVIMDRALPFIGDGLKPVQRRIVYAMSELGLNASAKF |
| RIVM_H_2016-10 | MSDMAERLALHEFTENAYLNYSMYVIMDRALPFIGDGLKPVQRRIVYAMSELGLNASAKF |
| RIVM_H_2016-11 | MSDMAERLALHEFTENAYLNYSMYVIMDRALPFIGDGLKPVQRRIVYAMSELGLNASAKF |
| RIVM_H_2016-12 | MSDMAERLALHEFTENAYLNYSMYVIMDRALPFIGDGLKPVQRRIVYAMSELGLNASAKF |
| RIVM_H_2016-13 | MSDMAERLALHEFTENAYLNYSMYVIMDRALPFIGDGLKPVQRRIVYAMSELGLNASAKF |
| RIVM_H_2016-14 | MSDMAERLALHEFTENAYLNYSMYVIMDRALPFIGDGLKPVQRRIVYAMSELGLNASAKF |
| RIVM_H_2016-15 | MSDMAERLALHEFTENAYLNYSMYVIMDRALPFIGDGLKPVQRRIVYAMSELGLNASAKF |
| RIVM_H_2017-01 | MSDMAERLALHEFTENAYLNYSMYVIMDRALPFIGDGLKPVQRRIVYAMSELGLNASAKF |
| RIVM_H_2017-02 | MSDMAERLALHEFTENAYLNYSMYVIMDRALPFIGDGLKPVQRRIVYAMSELGLNASAKF |
| RIVM_H_2017-03 | MSDMAERLALHEFTENAYLNYSMYVIMDRALPFIGDGLKPVQRRIVYAMSELGLNASAKF |
| RIVM_H_2017-04 | MSDMAERLALHEFTENAYLNYSMYVIMDRALPFIGDGLKPVQRRIVYAMSELGLNASAKF |
| RIVM_H_2017-05 | MSDMAERLALHEFTENAYLNYSMYVIMDRALPFIGDGLKPVQRRIVYAMSELGLNASAKF |
| RIVM_H_2017-06 | MSDMAERLALHEFTENAYLNYSMYVIMDRALPFIGDGLKPVQRRIVYAMSELGLNASAKF |
| RIVM_H_2017-07 | MSDMAERLALHEFTENAYLNYSMYVIMDRALPFIGDGLKPVQRRIVYAMSELGLNASAKF |
| RIVM_H_2017-08 | MSDMAERLALHEFTENAYLNYSMYVIMDRALPFIGDGLKPVQRRIVYAMSELGLNASAKF |
| RIVM_H_2017-09 | MSDMAERLALHEFTENAYLNYSMYVIMDRALPFIGDGLKPVQRRIVYAMSELGLNASAKF |
| RIVM_H_2017-10 | MSDMAERLALHEFTENAYLNYSMYVIMDRALPFIGDGLKPVQRRIVYAMSELGLNASAKF |
| RIVM_H_2017-11 | MSDMAERLALHEFTENAYLNYSMYVIMDRALPFIGDGLKPVQRRIVYAMSELGLNASAKF |
| RIVM_H_2017-12 | MSDMAERLALHEFTENAYLNYSMYVIMDRALPFIGDGLKPVQRRIVYAMSELGLNASAKF |
| RIVM_H_2017-13 | MSDMAERLALHEFTENAYLNYSMYVIMDRALPFIGDGLKPVQRRIVYAMSELGLNASAKF |
| RIVM_H_2017-14 | MSDMAERLALHEFTENAYLNYSMYVIMDRALPFIGDGLKPVQRRIVYAMSELGLNASAKF |
| RIVM_H_2017-15 | MSDMAERLALHEFTENAYLNYSMYVIMDRALPFIGDGLKPVQRRIVYAMSELGLNASAKF |
| RIVM_H_2017-16 | MSDMAERLALHEFTENAYLNYSMYVIMDRALPFIGDGLKPVQRRIVYAMSELGLNASAKF |
| RIVM_H_2017-17 | MSDMAERLALHEFTENAYLNYSMYVIMDRALPFIGDGLKPVQRRIVYAMSELGLNASAKF |
| RIVM_H_2017-18 | MSDMAERLALHEFTENAYLNYSMYVIMDRALPFIGDGLKPVQRRIVYAMSELGLNASAKF |
| RIVM_H_2017-19 | MSDMAERLALHEFTENAYLNYSMYVIMDRALPFIGDGLKPVQRRIVYAMSELGLNASAKF |
| 15EP001483 | MSDMAERLALHEFTENAYLNYSMYVIMDRALPFIGDGLKPVQRRIVYAMSELGLNASAKF |
| 17EP002363 | MSDMAERLALHEFTENAYLNYSMYVIMDRALPFIGDGLKPVQRRIVYAMSELGLNASAKF |
| S_0812_17 | MSDMAERLALHEFTENAYLNYSMYVIMDRALPFIGDGLKPVQRRIVYAMSELGLNASAKF |
| SRR1957844 | MSDMAERLALHEFTENAYLNYSMYVIMDRALPFIGDGLKPVQRRIVYAMSELGLNASAKF |
| SRR1958654 | MSDMAERLALHEFTENAYLNYSMYVIMDRALPFIGDGLKPVQRRIVYAMSELGLNASAKF |
| SRR1965077 | MSDMAERLALHEFTENAYLNYSMYVIMDRALPFIGDGLKPVQRRIVYAMSELGLNASAKF |
| SRR1966369 | MSDMAERLALHEFTENAYLNYSMYVIMDRALPFIGDGLKPVQRRIVYAMSELGLNASAKF |
| SRR1967117 | MSDMAERLALHEFTENAYLNYSMYVIMDRALPFIGDGLKPVQRRIVYAMSELGLNASAKF |
| SRR1967922 | MSDMAERLALHEFTENAYLNYSMYVIMDRALPFIGDGLKPVQRRIVYAMSELGLNASAKF |
| SRR8704720 | MSDMAERLALHEFTENAYLNYSMYVIMDRALPFIGDGLKPVQRRIVYAMSELGLNASAKF |
| SRR7216071 | MSDMAERLALHEFTENAYLNYSMYVIMDRALPFIGDGLKPVQRRIVYAMSELGLNASAKF |
| SRR7349175 | MSDMAERLALHEFTENAYLNYSMYVIMDRALPFIGDGLKPVQRRIVYAMSELGLNASAKF |
| SRR7523148 | MSDMAERLALHEFTENAYLNYSMYVIMDRALPFIGDGLKPVQRRIVYAMSELGLNASAKF |
| SRR7523854 | MSDMAERLALHEFTENAYLNYSMYVIMDRALPFIGDGLKPVQRRIVYAMSELGLNASAKF |
| 313865 | MSDMAERLALHEFTENAYLNYSMYVIMDRALPFIGDGLKPVQRRIVYAMSELGLNASAKF |
| SRR7277793 | MSDMAERLALHEFTENAYLNYSMYVIMDRALPFIGDGLKPVQRRIVYAMSELGLNASAKF |
| SRR7343877 | MSDMAERLALHEFTENAYLNYSMYVIMDRALPFIGDGLKPVQRRIVYAMSELGLNASAKF |
| SRR7351477 | MSDMAERLALHEFTENAYLNYSMYVIMDRALPFIGDGLKPVQRRIVYAMSELGLNASAKF |
| SRR5583183 | MSDMAERLALHEFTENAYLNYSMYVIMDRALPFIGDGLKPVQRRIVYAMSELGLNASAKF |
| SRR5585240 | MSDMAERLALHEFTENAYLNYSMYVIMDRALPFIGDGLKPVQRRIVYAMSELGLNASAKF |
| SRR7284317 | MSDMAERLALHEFTENAYLNYSMYVIMDRALPFIGDGLKPVQRRIVYAMSELGLNASAKF |
| SRR7299161 | MSDMAERLALHEFTENAYLNYSMYVIMDRALPFIGDGLKPVQRRIVYAMSELGLNASAKF |
| SRR7401730 | MSDMAERLALHEFTENAYLNYSMYVIMDRALPFIGDGLKPVQRRIVYAMSELGLNASAKF |
| SRR7469092 | MSDMAERLALHEFTENAYLNYSMYVIMDRALPFIGDGLKPVQRRIVYAMSELGLNASAKF |
| SRR7879556 | MSDMAERLALHEFTENAYLNYSMYVIMDRALPFIGDGLKPVQRRIVYAMSELGLNASAKF |
| SRR8526100 | MSDMAERLALHEFTENAYLNYSMYVIMDRALPFIGDGLKPVQRRIVYAMSELGLNASAKF |
| SRR8553991 | MSDMAERLALHEFTENAYLNYSMYVIMDRALPFIGDGLKPVQRRIVYAMSELGLNASAKF |
| SRR7842487 | MSDMAERLALHEFTENAYLNYSMYVIMDRALPFIGDGLKPVQRRIVYAMSELGLNASAKF |
| SRR8054524 | MSDMAERLALHEFTENAYLNYSMYVIMDRALPFIGDGLKPVQRRIVYAMSELGLNASAKF |
| SRR8054525 | MSDMAERLALHEFTENAYLNYSMYVIMDRALPFIGDGLKPVQRRIVYAMSELGLNASAKF |
| SRR8524733 | MSDMAERLALHEFTENAYLNYSMYVIMDRALPFIGDGLKPVQRRIVYAMSELGLNASAKF |
| SRR4093291 | MSDMAERLALHEFTENAYLNYSMYVIMDRALPFIGDGLKPVQRRIVYAMSELGLNASAKF |
| SRR4245549 | MSDMAERLALHEFTENAYLNYSMYVIMDRALPFIGDGLKPVQRRIVYAMSELGLNASAKF |
| SRR3057154 | MSDMAERLALHEFTENAYLNYSMYVIMDRALPFIGDGLKPVQRRIVYAMSELGLNASAKF |
| SRR1726150 | MSDMAERLALHEFTENAYLNYSMYVIMDRALPFIGDGLKPVQRRIVYAMSELGLNASAKF |
| SRR1996141 | MSDMAERLALHEFTENAYLNYSMYVIMDRALPFIGDGLKPVQRRIVYAMSELGLNASAKF |
| SRR1107842 | MSDMAERLALHEFTENAYLNYSMYVIMDRALPFIGDGLKPVQRRIVYAMSELGLNASAKF |
| SRR1157587 | MSDMAERLALHEFTENAYLNYSMYVIMDRALPFIGDGLKPVQRRIVYAMSELGLNASAKF |
| SRR3027706 | MSDMAERLALHEFTENAYLNYSMYVIMDRALPFIGDGLKPVQRRIVYAMSELGLNASAKF |
| SRR3027707 | MSDMAERLALHEFTENAYLNYSMYVIMDRALPFIGDGLKPVQRRIVYAMSELGLNASAKF |
| SRR3027708 | MSDMAERLALHEFTENAYLNYSMYVIMDRALPFIGDGLKPVQRRIVYAMSELGLNASAKF |
| SRR3027710 | MSDMAERLALHEFTENAYLNYSMYVIMDRALPFIGDGLKPVQRRIVYAMSELGLNASAKF |
| SRR3027711 | MSDMAERLALHEFTENAYLNYSMYVIMDRALPFIGDGLKPVQRRIVYAMSELGLNASAKF |
| SRR3027716 | MSDMAERLALHEFTENAYLNYSMYVIMDRALPFIGDGLKPVQRRIVYAMSELGLNASAKF |
| SRR3027717 | MSDMAERLALHEFTENAYLNYSMYVIMDRALPFIGDGLKPVQRRIVYAMSELGLNASAKF |
| SRR3027719 | MSDMAERLALHEFTENAYLNYSMYVIMDRALPFIGDGLKPVQRRIVYAMSELGLNASAKF |
| SRR3027721 | MSDMAERLALHEFTENAYLNYSMYVIMDRALPFIGDGLKPVQRRIVYAMSELGLNASAKF |
| SRR3027723 | MSDMAERLALHEFTENAYLNYSMYVIMDRALPFIGDGLKPVQRRIVYAMSELGLNASAKF |
| SRR3115978 | MSDMAERLALHEFTENAYLNYSMYVIMDRALPFIGDGLKPVQRRIVYAMSELGLNASAKF |
| SRR2534093 | MSDMAERLALHEFTENAYLNYSMYVIMDRALPFIGDGLKPVQRRIVYAMSELGLNASAKF |
| SRR2534094 | MSDMAERLALHEFTENAYLNYSMYVIMDRALPFIGDGLKPVQRRIVYAMSELGLNASAKF |
| SRR2534095 | MSDMAERLALHEFTENAYLNYSMYVIMDRALPFIGDGLKPVQRRIVYAMSELGLNASAKF |
| SRR2534108 | MSDMAERLALHEFTENAYLNYSMYVIMDRALPFIGDGLKPVQRRIVYAMSELGLNASAKF |
| SRR1106464 | MSDMAERLALHEFTENAYLNYSMYVIMDRALPFIGDGLKPVQRRIVYAMSELGLNASAKF |
| SRR1106463 | MSDMAERLALHEFTENAYLNYSMYVIMDRALPFIGDGLKPVQRRIVYAMSELGLNASAKF |
| SRR6949610 | MSDMAERLALHEFTENAYLNYSMYVIMDRALPFIGDGLKPVQRRIVYAMSELGLNASAKF |
| SRR6950452 | MSDMAERLALHEFTENAYLNYSMYVIMDRALPFIGDGLKPVQRRIVYAMSELGLNASAKF |
| ERR2019831 | MSDMAERLALHEFTENAYLNYSMYVIMDRALPFIGDGLKPVQRRIVYAMSELGLNASAKF |
| SRR2085693 | MSDMAERLALHEFTENAYLNYSMYVIMDRALPFIGDGLKPVQRRIVYAMSELGLNASAKF |
| SRR2086898 | MSDMAERLALHEFTENAYLNYSMYVIMDRALPFIGDGLKPVQRRIVYAMSELGLNASAKF |
| SRR2175312 | MSDMAERLALHEFTENAYLNYSMYVIMDRALPFIGDGLKPVQRRIVYAMSELGLNASAKF |
| SRR2175360 | MSDMAERLALHEFTENAYLNYSMYVIMDRALPFIGDGLKPVQRRIVYAMSELGLNASAKF |
| SRR5231997 | MSDMAERLALHEFTENAYLNYSMYVIMDRALPFIGDGLKPVQRRIVYAMSELGLNASAKF |
| SRR5232003 | MSDMAERLALHEFTENAYLNYSMYVIMDRALPFIGDGLKPVQRRIVYAMSELGLNASAKF |
| SRR5232015 | MSDMAERLALHEFTENAYLNYSMYVIMDRALPFIGDGLKPVQRRIVYAMSELGLNASAKF |
| SRR949434 | MSDMAERLALHEFTENAYLNYSMYVIMDRALPFIGDGLKPVQRRIVYAMSELGLNASAKF |
| SRR3216575 | MSDMAERLALHEFTENAYLNYSMYVIMDRALPFIGDGLKPVQRRIVYAMSELGLNASAKF |
| SRR5205342 | MSDMAERLALHEFTENAYLNYSMYVIMDRALPFIGDGLKPVQRRIVYAMSELGLNASAKF |
| SRR1501669 | MSDMAERLALHEFTENAYLNYSMYVIMDRALPFIGDGLKPVQRRIVYAMSELGLNASAKF |
| SRR5209740 | MSDMAERLALHEFTENAYLNYSMYVIMDRALPFIGDGLKPVQRRIVYAMSELGLNASAKF |
| SRR3240355 | MSDMAERLALHEFTENAYLNYSMYVIMDRALPFIGDGLKPVQRRIVYAMSELGLNASAKF |
| SRR3392777 | MSDMAERLALHEFTENAYLNYSMYVIMDRALPFIGDGLKPVQRRIVYAMSELGLNASAKF |
| SRR3593671 | MSDMAERLALHEFTENAYLNYSMYVIMDRALPFIGDGLKPVQRRIVYAMSELGLNASAKF |
| SRR5413290 | MSDMAERLALHEFTENAYLNYSMYVIMDRALPFIGDGLKPVQRRIVYAMSELGLNASAKF |
| SRR5590269 | MSDMAERLALHEFTENAYLNYSMYVIMDRALPFIGDGLKPVQRRIVYAMSELGLNASAKF |
| SRR5812103 | MSDMAERLALHEFTENAYLNYSMYVIMDRALPFIGDGLKPVQRRIVYAMSELGLNASAKF |
| SRR2830941 | MSDMAERLALHEFTENAYLNYSMYVIMDRALPFIGDGLKPVQRRIVYAMSELGLNASAKF |
| SRR2830966 | MSDMAERLALHEFTENAYLNYSMYVIMDRALPFIGDGLKPVQRRIVYAMSELGLNASAKF |
| SRR3137270 | MSDMAERLALHEFTENAYLNYSMYVIMDRALPFIGDGLKPVQRRIVYAMSELGLNASAKF |
| SRR3137271 | MSDMAERLALHEFTENAYLNYSMYVIMDRALPFIGDGLKPVQRRIVYAMSELGLNASAKF |
| ERR526807 | MSDMAERLALHEFTENAYLNYSMYVIMDRALPFIGDGLKPVQRRIVYAMSELGLNASAKF |
| ERR2197922 | MSDMAERLALHEFTENAYLNYSMYVIMDRALPFIGDGLKPVQRRIVYAMSELGLNASAKF |
| ERR2197923 | MSDMAERLALHEFTENAYLNYSMYVIMDRALPFIGDGLKPVQRRIVYAMSELGLNASAKF |
| ERR2197924 | MSDMAERLALHEFTENAYLNYSMYVIMDRALPFIGDGLKPVQRRIVYAMSELGLNASAKF |
| ERR2197925 | MSDMAERLALHEFTENAYLNYSMYVIMDRALPFIGDGLKPVQRRIVYAMSELGLNASAKF |
| ERR2197927 | MSDMAERLALHEFTENAYLNYSMYVIMDRALPFIGDGLKPVQRRIVYAMSELGLNASAKF |
| ERR2197929 | MSDMAERLALHEFTENAYLNYSMYVIMDRALPFIGDGLKPVQRRIVYAMSELGLNASAKF |
| SRR1648149 | MSDMAERLALHEFTENAYLNYSMYVIMDRALPFIGDGLKPVQRRIVYAMSELGLNASAKF |
| SRR1048299 | MSDMAERLALHEFTENAYLNYSMYVIMDRALPFIGDGLKPVQRRIVYAMSELGLNASAKF |
| SRR1300677 | MSDMAERLALHEFTENAYLNYSMYVIMDRALPFIGDGLKPVQRRIVYAMSELGLNASAKF |
| SRR1288356 | MSDMAERLALHEFTENAYLNYSMYVIMDRALPFIGDGLKPVQRRIVYAMSELGLNASAKF |
| SRR7426190 | MSDMAERLALHEFTENAYLNYSMYVIMDRALPFIGDGLKPVQRRIVYAMSELGLNASAKF |
| SRR7426192 | MSDMAERLALHEFTENAYLNYSMYVIMDRALPFIGDGLKPVQRRIVYAMSELGLNASAKF |
| SRR7426193 | MSDMAERLALHEFTENAYLNYSMYVIMDRALPFIGDGLKPVQRRIVYAMSELGLNASAKF |
| SRR7441832 | MSDMAERLALHEFTENAYLNYSMYVIMDRALPFIGDGLKPVQRRIVYAMSELGLNASAKF |
| SRR7426179 | MSDMAERLALHEFTENAYLNYSMYVIMDRALPFIGDGLKPVQRRIVYAMSELGLNASAKF |
| SRR7439238 | MSDMAERLALHEFTENAYLNYSMYVIMDRALPFIGDGLKPVQRRIVYAMSELGLNASAKF |
| SRR7439244 | MSDMAERLALHEFTENAYLNYSMYVIMDRALPFIGDGLKPVQRRIVYAMSELGLNASAKF |
| SRR7439259 | MSDMAERLALHEFTENAYLNYSMYVIMDRALPFIGDGLKPVQRRIVYAMSELGLNASAKF |
| SRR7439260 | MSDMAERLALHEFTENAYLNYSMYVIMDRALPFIGDGLKPVQRRIVYAMSELGLNASAKF |
| SRR7441786 | MSDMAERLALHEFTENAYLNYSMYVIMDRALPFIGDGLKPVQRRIVYAMSELGLNASAKF |
| SRR7441797 | MSDMAERLALHEFTENAYLNYSMYVIMDRALPFIGDGLKPVQRRIVYAMSELGLNASAKF |
| ERR1759093 | MSDMAERLALHEFTENAYLNYSMYVIMDRALPFIGDGLKPVQRRIVYAMSELGLNASAKF |
| ERR2580275 | MSDMAERLALHEFTENAYLNYSMYVIMDRALPFIGDGLKPVQRRIVYAMSELGLNASAKF |
| ERR1759204 | MSDMAERLALHEFTENAYLNYSMYVIMDRALPFIGDGLKPVQRRIVYAMSELGLNASAKF |
| SRR1300699 | MSDMAERLALHEFTENAYLNYSMYVIMDRALPFIGDGLKPVQRRIVYAMSELGLNASAKF |
| S_0825_17 | MSDMAERLALHEFTENAYLNYSMYVIMDRALPFIGDGLKPVQRRIVYAMSELGLNASAKF |
| SRR1958215 | MSDMAERLALHEFTENAYLNYSMYVIMDRALPFIGDGLKPVQRRIVYAMSELGLNASAKF |
| SRR1958540 | MSDMAERLALHEFTENAYLNYSMYVIMDRALPFIGDGLKPVQRRIVYAMSELGLNASAKF |
| SRR1958636 | MSDMAERLALHEFTENAYLNYSMYVIMDRALPFIGDGLKPVQRRIVYAMSELGLNASAKF |
| SRR1959422 | MSDMAERLALHEFTENAYLNYSMYVIMDRALPFIGDGLKPVQRRIVYAMSELGLNASAKF |
| SRR1959427 | MSDMAERLALHEFTENAYLNYSMYVIMDRALPFIGDGLKPVQRRIVYAMSELGLNASAKF |
| SRR1960226 | MSDMAERLALHEFTENAYLNYSMYVIMDRALPFIGDGLKPVQRRIVYAMSELGLNASAKF |
| SRR1963498 | MSDMAERLALHEFTENAYLNYSMYVIMDRALPFIGDGLKPVQRRIVYAMSELGLNASAKF |
| SRR1965947 | MSDMAERLALHEFTENAYLNYSMYVIMDRALPFIGDGLKPVQRRIVYAMSELGLNASAKF |
| SRR1966125 | MSDMAERLALHEFTENAYLNYSMYVIMDRALPFIGDGLKPVQRRIVYAMSELGLNASAKF |
| SRR1966330 | MSDMAERLALHEFTENAYLNYSMYVIMDRALPFIGDGLKPVQRRIVYAMSELGLNASAKF |
| SRR1966565 | MSDMAERLALHEFTENAYLNYSMYVIMDRALPFIGDGLKPVQRRIVYAMSELGLNASAKF |
| SRR1966864 | MSDMAERLALHEFTENAYLNYSMYVIMDRALPFIGDGLKPVQRRIVYAMSELGLNASAKF |
| SRR1966989 | MSDMAERLALHEFTENAYLNYSMYVIMDRALPFIGDGLKPVQRRIVYAMSELGLNASAKF |
| SRR1967688 | MSDMAERLALHEFTENAYLNYSMYVIMDRALPFIGDGLKPVQRRIVYAMSELGLNASAKF |
| SRR1967733 | MSDMAERLALHEFTENAYLNYSMYVIMDRALPFIGDGLKPVQRRIVYAMSELGLNASAKF |
| SRR1967746 | MSDMAERLALHEFTENAYLNYSMYVIMDRALPFIGDGLKPVQRRIVYAMSELGLNASAKF |
| SRR1968341 | MSDMAERLALHEFTENAYLNYSMYVIMDRALPFIGDGLKPVQRRIVYAMSELGLNASAKF |
| SRR1968456 | MSDMAERLALHEFTENAYLNYSMYVIMDRALPFIGDGLKPVQRRIVYAMSELGLNASAKF |
| SRR1968465 | MSDMAERLALHEFTENAYLNYSMYVIMDRALPFIGDGLKPVQRRIVYAMSELGLNASAKF |
| SRR1968761 | MSDMAERLALHEFTENAYLNYSMYVIMDRALPFIGDGLKPVQRRIVYAMSELGLNASAKF |
| SRR1969047 | MSDMAERLALHEFTENAYLNYSMYVIMDRALPFIGDGLKPVQRRIVYAMSELGLNASAKF |
| SRR1969255 | MSDMAERLALHEFTENAYLNYSMYVIMDRALPFIGDGLKPVQRRIVYAMSELGLNASAKF |
| SRR1969412 | MSDMAERLALHEFTENAYLNYSMYVIMDRALPFIGDGLKPVQRRIVYAMSELGLNASAKF |
| SRR1969524 | MSDMAERLALHEFTENAYLNYSMYVIMDRALPFIGDGLKPVQRRIVYAMSELGLNASAKF |
| SRR1969584 | MSDMAERLALHEFTENAYLNYSMYVIMDRALPFIGDGLKPVQRRIVYAMSELGLNASAKF |
| SRR1969648 | MSDMAERLALHEFTENAYLNYSMYVIMDRALPFIGDGLKPVQRRIVYAMSELGLNASAKF |
| SRR1969804 | MSDMAERLALHEFTENAYLNYSMYVIMDRALPFIGDGLKPVQRRIVYAMSELGLNASAKF |
| SRR1970221 | MSDMAERLALHEFTENAYLNYSMYVIMDRALPFIGDGLKPVQRRIVYAMSELGLNASAKF |
| SRR1970268 | MSDMAERLALHEFTENAYLNYSMYVIMDRALPFIGDGLKPVQRRIVYAMSELGLNASAKF |
| SRR1965862 | MSDMAERLALHEFTENAYLNYSMYVIMDRALPFIGDGLKPVQRRIVYAMSELGLNASAKF |
| SRR1967363 | MSDMAERLALHEFTENAYLNYSMYVIMDRALPFIGDGLKPVQRRIVYAMSELGLNASAKF |
| SRR1968276 | MSDMAERLALHEFTENAYLNYSMYVIMDRALPFIGDGLKPVQRRIVYAMSELGLNASAKF |
| SRR1968967 | MSDMAERLALHEFTENAYLNYSMYVIMDRALPFIGDGLKPVQRRIVYAMSELGLNASAKF |
| SRR3321531 | MSDMAERLALHEFTENAYLNYSMYVIMDRALPFIGDGLKPVQRRIVYAMSELGLNASAKF |
| SRR3321883 | MSDMAERLALHEFTENAYLNYSMYVIMDRALPFIGDGLKPVQRRIVYAMSELGLNASAKF |
| SRR3322413 | MSDMAERLALHEFTENAYLNYSMYVIMDRALPFIGDGLKPVQRRIVYAMSELGLNASAKF |
| SRR3323012 | MSDMAERLALHEFTENAYLNYSMYVIMDRALPFIGDGLKPVQRRIVYAMSELGLNASAKF |
| SRR5194289 | MSDMAERLALHEFTENAYLNYSMYVIMDRALPFIGDGLKPVQRRIVYAMSELGLNASAKF |
| SRR7163798 | MSDMAERLALHEFTENAYLNYSMYVIMDRALPFIGDGLKPVQRRIVYAMSELGLNASAKF |
| SRR7172610 | MSDMAERLALHEFTENAYLNYSMYVIMDRALPFIGDGLKPVQRRIVYAMSELGLNASAKF |
| SRR7204568 | MSDMAERLALHEFTENAYLNYSMYVIMDRALPFIGDGLKPVQRRIVYAMSELGLNASAKF |
| SRR7223230 | MSDMAERLALHEFTENAYLNYSMYVIMDRALPFIGDGLKPVQRRIVYAMSELGLNASAKF |
| SRR7230675 | MSDMAERLALHEFTENAYLNYSMYVIMDRALPFIGDGLKPVQRRIVYAMSELGLNASAKF |
| SRR7278056 | MSDMAERLALHEFTENAYLNYSMYVIMDRALPFIGDGLKPVQRRIVYAMSELGLNASAKF |
| SRR7278086 | MSDMAERLALHEFTENAYLNYSMYVIMDRALPFIGDGLKPVQRRIVYAMSELGLNASAKF |
| SRR7285841 | MSDMAERLALHEFTENAYLNYSMYVIMDRALPFIGDGLKPVQRRIVYAMSELGLNASAKF |
| SRR7292625 | MSDMAERLALHEFTENAYLNYSMYVIMDRALPFIGDGLKPVQRRIVYAMSELGLNASAKF |
| SRR7292665 | MSDMAERLALHEFTENAYLNYSMYVIMDRALPFIGDGLKPVQRRIVYAMSELGLNASAKF |
| SRR7297965 | MSDMAERLALHEFTENAYLNYSMYVIMDRALPFIGDGLKPVQRRIVYAMSELGLNASAKF |
| SRR7350726 | MSDMAERLALHEFTENAYLNYSMYVIMDRALPFIGDGLKPVQRRIVYAMSELGLNASAKF |
| SRR7410328 | MSDMAERLALHEFTENAYLNYSMYVIMDRALPFIGDGLKPVQRRIVYAMSELGLNASAKF |
| SRR7474665 | MSDMAERLALHEFTENAYLNYSMYVIMDRALPFIGDGLKPVQRRIVYAMSELGLNASAKF |
| SRR7523184 | MSDMAERLALHEFTENAYLNYSMYVIMDRALPFIGDGLKPVQRRIVYAMSELGLNASAKF |
| SRR7187264 | MSDMAERLALHEFTENAYLNYSMYVIMDRALPFIGDGLKPVQRRIVYAMSELGLNASAKF |
| SRR7204445 | MSDMAERLALHEFTENAYLNYSMYVIMDRALPFIGDGLKPVQRRIVYAMSELGLNASAKF |
| SRR7285641 | MSDMAERLALHEFTENAYLNYSMYVIMDRALPFIGDGLKPVQRRIVYAMSELGLNASAKF |
| SRR7286695 | MSDMAERLALHEFTENAYLNYSMYVIMDRALPFIGDGLKPVQRRIVYAMSELGLNASAKF |
| SRR7286705 | MSDMAERLALHEFTENAYLNYSMYVIMDRALPFIGDGLKPVQRRIVYAMSELGLNASAKF |
| SRR7292931 | MSDMAERLALHEFTENAYLNYSMYVIMDRALPFIGDGLKPVQRRIVYAMSELGLNASAKF |
| SRR7310349 | MSDMAERLALHEFTENAYLNYSMYVIMDRALPFIGDGLKPVQRRIVYAMSELGLNASAKF |
| SRR7351616 | MSDMAERLALHEFTENAYLNYSMYVIMDRALPFIGDGLKPVQRRIVYAMSELGLNASAKF |
| SRR7414818 | MSDMAERLALHEFTENAYLNYSMYVIMDRALPFIGDGLKPVQRRIVYAMSELGLNASAKF |
| SRR7426480 | MSDMAERLALHEFTENAYLNYSMYVIMDRALPFIGDGLKPVQRRIVYAMSELGLNASAKF |
| SRR5584105 | MSDMAERLALHEFTENAYLNYSMYVIMDRALPFIGDGLKPVQRRIVYAMSELGLNASAKF |
| SRR5584565 | MSDMAERLALHEFTENAYLNYSMYVIMDRALPFIGDGLKPVQRRIVYAMSELGLNASAKF |
| SRR5584614 | MSDMAERLALHEFTENAYLNYSMYVIMDRALPFIGDGLKPVQRRIVYAMSELGLNASAKF |
| SRR5631543 | MSDMAERLALHEFTENAYLNYSMYVIMDRALPFIGDGLKPVQRRIVYAMSELGLNASAKF |
| SRR5631553 | MSDMAERLALHEFTENAYLNYSMYVIMDRALPFIGDGLKPVQRRIVYAMSELGLNASAKF |
| SRR7123196 | MSDMAERLALHEFTENAYLNYSMYVIMDRALPFIGDGLKPVQRRIVYAMSELGLNASAKF |
| SRR7163819 | MSDMAERLALHEFTENAYLNYSMYVIMDRALPFIGDGLKPVQRRIVYAMSELGLNASAKF |
| SRR7163920 | MSDMAERLALHEFTENAYLNYSMYVIMDRALPFIGDGLKPVQRRIVYAMSELGLNASAKF |
| SRR7209528 | MSDMAERLALHEFTENAYLNYSMYVIMDRALPFIGDGLKPVQRRIVYAMSELGLNASAKF |
| SRR7249868 | MSDMAERLALHEFTENAYLNYSMYVIMDRALPFIGDGLKPVQRRIVYAMSELGLNASAKF |
| SRR7278088 | MSDMAERLALHEFTENAYLNYSMYVIMDRALPFIGDGLKPVQRRIVYAMSELGLNASAKF |
| SRR7285788 | MSDMAERLALHEFTENAYLNYSMYVIMDRALPFIGDGLKPVQRRIVYAMSELGLNASAKF |
| SRR7286789 | MSDMAERLALHEFTENAYLNYSMYVIMDRALPFIGDGLKPVQRRIVYAMSELGLNASAKF |
| SRR7286886 | MSDMAERLALHEFTENAYLNYSMYVIMDRALPFIGDGLKPVQRRIVYAMSELGLNASAKF |
| SRR7310632 | MSDMAERLALHEFTENAYLNYSMYVIMDRALPFIGDGLKPVQRRIVYAMSELGLNASAKF |
| SRR7350631 | MSDMAERLALHEFTENAYLNYSMYVIMDRALPFIGDGLKPVQRRIVYAMSELGLNASAKF |
| SRR7458741 | MSDMAERLALHEFTENAYLNYSMYVIMDRALPFIGDGLKPVQRRIVYAMSELGLNASAKF |
| SRR7480280 | MSDMAERLALHEFTENAYLNYSMYVIMDRALPFIGDGLKPVQRRIVYAMSELGLNASAKF |
| SRR7523660 | MSDMAERLALHEFTENAYLNYSMYVIMDRALPFIGDGLKPVQRRIVYAMSELGLNASAKF |
| SRR7523775 | MSDMAERLALHEFTENAYLNYSMYVIMDRALPFIGDGLKPVQRRIVYAMSELGLNASAKF |
| SRR7251101 | MSDMAERLALHEFTENAYLNYSMYVIMDRALPFIGDGLKPVQRRIVYAMSELGLNASAKF |
| SRR7284299 | MSDMAERLALHEFTENAYLNYSMYVIMDRALPFIGDGLKPVQRRIVYAMSELGLNASAKF |
| SRR7285738 | MSDMAERLALHEFTENAYLNYSMYVIMDRALPFIGDGLKPVQRRIVYAMSELGLNASAKF |
| SRR7310640 | MSDMAERLALHEFTENAYLNYSMYVIMDRALPFIGDGLKPVQRRIVYAMSELGLNASAKF |
| SRR7349159 | MSDMAERLALHEFTENAYLNYSMYVIMDRALPFIGDGLKPVQRRIVYAMSELGLNASAKF |
| SRR7474873 | MSDMAERLALHEFTENAYLNYSMYVIMDRALPFIGDGLKPVQRRIVYAMSELGLNASAKF |
| SRR7495689 | MSDMAERLALHEFTENAYLNYSMYVIMDRALPFIGDGLKPVQRRIVYAMSELGLNASAKF |
| SRR7495752 | MSDMAERLALHEFTENAYLNYSMYVIMDRALPFIGDGLKPVQRRIVYAMSELGLNASAKF |
| ------------------------------------------------------------------------------ | |
| S16BD08730 | KKSARTVGDVLGKYHPHGDIACYEAMVLMAQPFSYRYPLVDGQGNWGAPDDPKSFAAMRY |
| S18BD00684 | KKSARTVGDVLGKYHPHGDIACYEAMVLMAQPFSYRYPLVDGQGNWGAPDDPKSFAAMRY |
| S18BD03994 | KKSARTVGDVLGKYHPHGDIACYEAMVLMAQPFSYRYPLVDGQGNWGAPDDPKSFAAMRY |
| S18BD05011 | KKSARTVGDVLGKYHPHGDIACYEAMVLMAQPFSYRYPLVDGQGNWGAPDDPKSFAAMRY |
| RKI_16-03723 | KKSARTVGDVLGKYHPHGDIACYEAMVLMAQPFSYRYPLVDGQGNWGAPDDPKSFAAMRY |
| RKI_16-04315 | KKSARTVGDVLGKYHPHGDIACYEAMVLMAQPFSYRYPLVDGQGNWGAPDDPKSFAAMRY |
| RKI_17-02304 | KKSARTVGDVLGKYHPHGDIACYEAMVLMAQPFSYRYPLVDGQGNWGAPDDPKSFAAMRY |
| RKI_17-02411 | KKSARTVGDVLGKYHPHGDIACYEAMVLMAQPFSYRYPLVDGQGNWGAPDDPKSFAAMRY |
| RKI_17-02757 | KKSARTVGDVLGKYHPHGDIACYEAMVLMAQPFSYRYPLVDGQGNWGAPDDPKSFAAMRY |
| RKI_17-04797 | KKSARTVGDVLGKYHPHGDIACYEAMVLMAQPFSYRYPLVDGQGNWGAPDDPKSFAAMRY |
| RKI_17-06869 | KKSARTVGDVLGKYHPHGDIACYEAMVLMAQPFSYRYPLVDGQGNWGAPDDPKSFAAMRY |
| ERR2580277 | KKSARTVGDVLGKYHPHGDIACYEAMVLMAQPFSYRYPLVDGQGNWGAPDDPKSFAAMRY |
| ERR2580276 | KKSARTVGDVLGKYHPHGDIACYEAMVLMAQPFSYRYPLVDGQGNWGAPDDPKSFAAMRY |
| ERR2580273 | KKSARTVGDVLGKYHPHGDIACYEAMVLMAQPFSYRYPLVDGQGNWGAPDDPKSFAAMRY |
| ERR2580274 | KKSARTVGDVLGKYHPHGDIACYEAMVLMAQPFSYRYPLVDGQGNWGAPDDPKSFAAMRY |
| ERR2173656 | KKSARTVGDVLGKYHPHGDIACYEAMVLMAQPFSYRYPLVDGQGNWGAPDDPKSFAAMRY |
| 17041676 | KKSARTVGDVLGKYHPHGDIACYEAMVLMAQPFSYRYPLVDGQGNWGAPDDPKSFAAMRY |
| MT16-000061 | KKSARTVGDVLGKYHPHGDIACYEAMVLMAQPFSYRYPLVDGQGNWGAPDDPKSFAAMRY |
| MT16-019416 | KKSARTVGDVLGKYHPHGDIACYEAMVLMAQPFSYRYPLVDGQGNWGAPDDPKSFAAMRY |
| MT16-027865 | KKSARTVGDVLGKYHPHGDIACYEAMVLMAQPFSYRYPLVDGQGNWGAPDDPKSFAAMRY |
| MT16-031693 | KKSARTVGDVLGKYHPHGDIACYEAMVLMAQPFSYRYPLVDGQGNWGAPDDPKSFAAMRY |
| MT16-040253 | KKSARTVGDVLGKYHPHGDIACYEAMVLMAQPFSYRYPLVDGQGNWGAPDDPKSFAAMRY |
| MT16-045379 | KKSARTVGDVLGKYHPHGDIACYEAMVLMAQPFSYRYPLVDGQGNWGAPDDPKSFAAMRY |
| MT16-442728 | KKSARTVGDVLGKYHPHGDIACYEAMVLMAQPFSYRYPLVDGQGNWGAPDDPKSFAAMRY |
| MT16-462857 | KKSARTVGDVLGKYHPHGDIACYEAMVLMAQPFSYRYPLVDGQGNWGAPDDPKSFAAMRY |
| MT16-480196 | KKSARTVGDVLGKYHPHGDIACYEAMVLMAQPFSYRYPLVDGQGNWGAPDDPKSFAAMRY |
| MT16-861555 | KKSARTVGDVLGKYHPHGDIACYEAMVLMAQPFSYRYPLVDGQGNWGAPDDPKSFAAMRY |
| MT17-076833 | KKSARTVGDVLGKYHPHGDIACYEAMVLMAQPFSYRYPLVDGQGNWGAPDDPKSFAAMRY |
| MT17-110677 | KKSARTVGDVLGKYHPHGDIACYEAMVLMAQPFSYRYPLVDGQGNWGAPDDPKSFAAMRY |
| MT17-131730 | KKSARTVGDVLGKYHPHGDIACYEAMVLMAQPFSYRYPLVDGQGNWGAPDDPKSFAAMRY |
| MT17-140890 | KKSARTVGDVLGKYHPHGDIACYEAMVLMAQPFSYRYPLVDGQGNWGAPDDPKSFAAMRY |
| MT17-141840 | KKSARTVGDVLGKYHPHGDIACYEAMVLMAQPFSYRYPLVDGQGNWGAPDDPKSFAAMRY |
| MT17-152488 | KKSARTVGDVLGKYHPHGDIACYEAMVLMAQPFSYRYPLVDGQGNWGAPDDPKSFAAMRY |
| MT17-157311 | KKSARTVGDVLGKYHPHGDIACYEAMVLMAQPFSYRYPLVDGQGNWGAPDDPKSFAAMRY |
| MT17-161645 | KKSARTVGDVLGKYHPHGDIACYEAMVLMAQPFSYRYPLVDGQGNWGAPDDPKSFAAMRY |
| MT17-167951 | KKSARTVGDVLGKYHPHGDIACYEAMVLMAQPFSYRYPLVDGQGNWGAPDDPKSFAAMRY |
| MT18-217732 | KKSARTVGDVLGKYHPHGDIACYEAMVLMAQPFSYRYPLVDGQGNWGAPDDPKSFAAMRY |
| MT18-252580 | KKSARTVGDVLGKYHPHGDIACYEAMVLMAQPFSYRYPLVDGQGNWGAPDDPKSFAAMRY |
| RIVM_H_2009-01 | KKSARTVGDVLGKYHPHGDIACYEAMVLMAQPFSYRYPLVDGQGNWGAPDDPKSFAAMRY |
| RIVM_H_2010-01 | KKSARTVGDVLGKYHPHGDIACYEAMVLMAQPFSYRYPLVDGQGNWGAPDDPKSFAAMRY |
| RIVM_H_2010-02 | KKSARTVGDVLGKYHPHGDIACYEAMVLMAQPFSYRYPLVDGQGNWGAPDDPKSFAAMRY |
| RIVM_H_2011-01 | KKSARTVGDVLGKYHPHGDIACYEAMVLMAQPFSYRYPLVDGQGNWGAPDDPKSFAAMRY |
| RIVM_H_2011-02 | KKSARTVGDVLGKYHPHGDIACYEAMVLMAQPFSYRYPLVDGQGNWGAPDDPKSFAAMRY |
| RIVM_H_2011-03 | KKSARTVGDVLGKYHPHGDIACYEAMVLMAQPFSYRYPLVDGQGNWGAPDDPKSFAAMRY |
| RIVM_H_2013-01 | KKSARTVGDVLGKYHPHGDIACYEAMVLMAQPFSYRYPLVDGQGNWGAPDDPKSFAAMRY |
| RIVM_H_2013-02 | KKSARTVGDVLGKYHPHGDIACYEAMVLMAQPFSYRYPLVDGQGNWGAPDDPKSFAAMRY |
| RIVM_H_2014-01 | KKSARTVGDVLGKYHPHGDIACYEAMVLMAQPFSYRYPLVDGQGNWGAPDDPKSFAAMRY |
| RIVM_H_2014-02 | KKSARTVGDVLGKYHPHGDIACYEAMVLMAQPFSYRYPLVDGQGNWGAPDDPKSFAAMRY |
| RIVM_H_2016-01 | KKSARTVGDVLGKYHPHGDIACYEAMVLMAQPFSYRYPLVDGQGNWGAPDDPKSFAAMRY |
| RIVM_H_2016-02 | KKSARTVGDVLGKYHPHGDIACYEAMVLMAQPFSYRYPLVDGQGNWGAPDDPKSFAAMRY |
| RIVM_H_2016-03 | KKSARTVGDVLGKYHPHGDIACYEAMVLMAQPFSYRYPLVDGQGNWGAPDDPKSFAAMRY |
| RIVM_H_2016-04 | KKSARTVGDVLGKYHPHGDIACYEAMVLMAQPFSYRYPLVDGQGNWGAPDDPKSFAAMRY |
| RIVM_H_2016-05 | KKSARTVGDVLGKYHPHGDIACYEAMVLMAQPFSYRYPLVDGQGNWGAPDDPKSFAAMRY |
| RIVM_H_2016-06 | KKSARTVGDVLGKYHPHGDIACYEAMVLMAQPFSYRYPLVDGQGNWGAPDDPKSFAAMRY |
| RIVM_H_2016-07 | KKSARTVGDVLGKYHPHGDIACYEAMVLMAQPFSYRYPLVDGQGNWGAPDDPKSFAAMRY |
| RIVM_H_2016-08 | KKSARTVGDVLGKYHPHGDIACYEAMVLMAQPFSYRYPLVDGQGNWGAPDDPKSFAAMRY |
| RIVM_H_2016-09 | KKSARTVGDVLGKYHPHGDIACYEAMVLMAQPFSYRYPLVDGQGNWGAPDDPKSFAAMRY |
| RIVM_H_2016-10 | KKSARTVGDVLGKYHPHGDIACYEAMVLMAQPFSYRYPLVDGQGNWGAPDDPKSFAAMRY |
| RIVM_H_2016-11 | KKSARTVGDVLGKYHPHGDIACYEAMVLMAQPFSYRYPLVDGQGNWGAPDDPKSFAAMRY |
| RIVM_H_2016-12 | KKSARTVGDVLGKYHPHGDIACYEAMVLMAQPFSYRYPLVDGQGNWGAPDDPKSFAAMRY |
| RIVM_H_2016-13 | KKSARTVGDVLGKYHPHGDIACYEAMVLMAQPFSYRYPLVDGQGNWGAPDDPKSFAAMRY |
| RIVM_H_2016-14 | KKSARTVGDVLGKYHPHGDIACYEAMVLMAQPFSYRYPLVDGQGNWGAPDDPKSFAAMRY |
| RIVM_H_2016-15 | KKSARTVGDVLGKYHPHGDIACYEAMVLMAQPFSYRYPLVDGQGNWGAPDDPKSFAAMRY |
| RIVM_H_2017-01 | KKSARTVGDVLGKYHPHGDIACYEAMVLMAQPFSYRYPLVDGQGNWGAPDDPKSFAAMRY |
| RIVM_H_2017-02 | KKSARTVGDVLGKYHPHGDIACYEAMVLMAQPFSYRYPLVDGQGNWGAPDDPKSFAAMRY |
| RIVM_H_2017-03 | KKSARTVGDVLGKYHPHGDIACYEAMVLMAQPFSYRYPLVDGQGNWGAPDDPKSFAAMRY |
| RIVM_H_2017-04 | KKSARTVGDVLGKYHPHGDIACYEAMVLMAQPFSYRYPLVDGQGNWGAPDDPKSFAAMRY |
| RIVM_H_2017-05 | KKSARTVGDVLGKYHPHGDIACYEAMVLMAQPFSYRYPLVDGQGNWGAPDDPKSFAAMRY |
| RIVM_H_2017-06 | KKSARTVGDVLGKYHPHGDIACYEAMVLMAQPFSYRYPLVDGQGNWGAPDDPKSFAAMRY |
| RIVM_H_2017-07 | KKSARTVGDVLGKYHPHGDIACYEAMVLMAQPFSYRYPLVDGQGNWGAPDDPKSFAAMRY |
| RIVM_H_2017-08 | KKSARTVGDVLGKYHPHGDIACYEAMVLMAQPFSYRYPLVDGQGNWGAPDDPKSFAAMRY |
| RIVM_H_2017-09 | KKSARTVGDVLGKYHPHGDIACYEAMVLMAQPFSYRYPLVDGQGNWGAPDDPKSFAAMRY |
| RIVM_H_2017-10 | KKSARTVGDVLGKYHPHGDIACYEAMVLMAQPFSYRYPLVDGQGNWGAPDDPKSFAAMRY |
| RIVM_H_2017-11 | KKSARTVGDVLGKYHPHGDIACYEAMVLMAQPFSYRYPLVDGQGNWGAPDDPKSFAAMRY |
| RIVM_H_2017-12 | KKSARTVGDVLGKYHPHGDIACYEAMVLMAQPFSYRYPLVDGQGNWGAPDDPKSFAAMRY |
| RIVM_H_2017-13 | KKSARTVGDVLGKYHPHGDIACYEAMVLMAQPFSYRYPLVDGQGNWGAPDDPKSFAAMRY |
| RIVM_H_2017-14 | KKSARTVGDVLGKYHPHGDIACYEAMVLMAQPFSYRYPLVDGQGNWGAPDDPKSFAAMRY |
| RIVM_H_2017-15 | KKSARTVGDVLGKYHPHGDIACYEAMVLMAQPFSYRYPLVDGQGNWGAPDDPKSFAAMRY |
| RIVM_H_2017-16 | KKSARTVGDVLGKYHPHGDIACYEAMVLMAQPFSYRYPLVDGQGNWGAPDDPKSFAAMRY |
| RIVM_H_2017-17 | KKSARTVGDVLGKYHPHGDIACYEAMVLMAQPFSYRYPLVDGQGNWGAPDDPKSFAAMRY |
| RIVM_H_2017-18 | KKSARTVGDVLGKYHPHGDIACYEAMVLMAQPFSYRYPLVDGQGNWGAPDDPKSFAAMRY |
| RIVM_H_2017-19 | KKSARTVGDVLGKYHPHGDIACYEAMVLMAQPFSYRYPLVDGQGNWGAPDDPKSFAAMRY |
| 15EP001483 | KKSARTVGDVLGKYHPHGDIACYEAMVLMAQPFSYRYPLVDGQGNWGAPDDPKSFAAMRY |
| 17EP002363 | KKSARTVGDVLGKYHPHGDIACYEAMVLMAQPFSYRYPLVDGQGNWGAPDDPKSFAAMRY |
| S_0812_17 | KKSARTVGDVLGKYHPHGDIACYEAMVLMAQPFSYRYPLVDGQGNWGAPDDPKSFAAMRY |
| SRR1957844 | KKSARTVGDVLGKYHPHGDIACYEAMVLMAQPFSYRYPLVDGQGNWGAPDDPKSFAAMRY |
| SRR1958654 | KKSARTVGDVLGKYHPHGDIACYEAMVLMAQPFSYRYPLVDGQGNWGAPDDPKSFAAMRY |
| SRR1965077 | KKSARTVGDVLGKYHPHGDIACYEAMVLMAQPFSYRYPLVDGQGNWGAPDDPKSFAAMRY |
| SRR1966369 | KKSARTVGDVLGKYHPHGDIACYEAMVLMAQPFSYRYPLVDGQGNWGAPDDPKSFAAMRY |
| SRR1967117 | KKSARTVGDVLGKYHPHGDIACYEAMVLMAQPFSYRYPLVDGQGNWGAPDDPKSFAAMRY |
| SRR1967922 | KKSARTVGDVLGKYHPHGDIACYEAMVLMAQPFSYRYPLVDGQGNWGAPDDPKSFAAMRY |
| SRR8704720 | KKSARTVGDVLGKYHPHGDIACYEAMVLMAQPFSYRYPLVDGQGNWGAPDDPKSFAAMRY |
| SRR7216071 | KKSARTVGDVLGKYHPHGDIACYEAMVLMAQPFSYRYPLVDGQGNWGAPDDPKSFAAMRY |
| SRR7349175 | KKSARTVGDVLGKYHPHGDIACYEAMVLMAQPFSYRYPLVDGQGNWGAPDDPKSFAAMRY |
| SRR7523148 | KKSARTVGDVLGKYHPHGDIACYEAMVLMAQPFSYRYPLVDGQGNWGAPDDPKSFAAMRY |
| SRR7523854 | KKSARTVGDVLGKYHPHGDIACYEAMVLMAQPFSYRYPLVDGQGNWGAPDDPKSFAAMRY |
| 313865 | KKSARTVGDVLGKYHPHGDIACYEAMVLMAQPFSYRYPLVDGQGNWGAPDDPKSFAAMRY |
| SRR7277793 | KKSARTVGDVLGKYHPHGDIACYEAMVLMAQPFSYRYPLVDGQGNWGAPDDPKSFAAMRY |
| SRR7343877 | KKSARTVGDVLGKYHPHGDIACYEAMVLMAQPFSYRYPLVDGQGNWGAPDDPKSFAAMRY |
| SRR7351477 | KKSARTVGDVLGKYHPHGDIACYEAMVLMAQPFSYRYPLVDGQGNWGAPDDPKSFAAMRY |
| SRR5583183 | KKSARTVGDVLGKYHPHGDIACYEAMVLMAQPFSYRYPLVDGQGNWGAPDDPKSFAAMRY |
| SRR5585240 | KKSARTVGDVLGKYHPHGDIACYEAMVLMAQPFSYRYPLVDGQGNWGAPDDPKSFAAMRY |
| SRR7284317 | KKSARTVGDVLGKYHPHGDIACYEAMVLMAQPFSYRYPLVDGQGNWGAPDDPKSFAAMRY |
| SRR7299161 | KKSARTVGDVLGKYHPHGDIACYEAMVLMAQPFSYRYPLVDGQGNWGAPDDPKSFAAMRY |
| SRR7401730 | KKSARTVGDVLGKYHPHGDIACYEAMVLMAQPFSYRYPLVDGQGNWGAPDDPKSFAAMRY |
| SRR7469092 | KKSARTVGDVLGKYHPHGDIACYEAMVLMAQPFSYRYPLVDGQGNWGAPDDPKSFAAMRY |
| SRR7879556 | KKSARTVGDVLGKYHPHGDIACYEAMVLMAQPFSYRYPLVDGQGNWGAPDDPKSFAAMRY |
| SRR8526100 | KKSARTVGDVLGKYHPHGDIACYEAMVLMAQPFSYRYPLVDGQGNWGAPDDPKSFAAMRY |
| SRR8553991 | KKSARTVGDVLGKYHPHGDIACYEAMVLMAQPFSYRYPLVDGQGNWGAPDDPKSFAAMRY |
| SRR7842487 | KKSARTVGDVLGKYHPHGDIACYEAMVLMAQPFSYRYPLVDGQGNWGAPDDPKSFAAMRY |
| SRR8054524 | KKSARTVGDVLGKYHPHGDIACYEAMVLMAQPFSYRYPLVDGQGNWGAPDDPKSFAAMRY |
| SRR8054525 | KKSARTVGDVLGKYHPHGDIACYEAMVLMAQPFSYRYPLVDGQGNWGAPDDPKSFAAMRY |
| SRR8524733 | KKSARTVGDVLGKYHPHGDIACYEAMVLMAQPFSYRYPLVDGQGNWGAPDDPKSFAAMRY |
| SRR4093291 | KKSARTVGDVLGKYHPHGDSACYEAMVLMAQPFSYRYPLVDGQGNWGAPDDPKSFAAMRY |
| SRR4245549 | KKSARTVGDVLGKYHPHGDSACYEAMVLMAQPFSYRYPLVDGQGNWGAPDDPKSFAAMRY |
| SRR3057154 | KKSARTVGDVLGKYHPHGDIACYEAMVLMAQPFSYRYPLVDGQGNWGAPDDPKSFAAMRY |
| SRR1726150 | KKSARTVGDVLGKYHPHGDIACYEAMVLMAQPFSYRYPLVDGQGNWGAPDDPKSFAAMRY |
| SRR1996141 | KKSARTVGDVLGKYHPHGDIACYEAMVLMAQPFSYRYPLVDGQGNWGAPDDPKSFAAMRY |
| SRR1107842 | KKSARTVGDVLGKYHPHGDIACYEAMVLMAQPFSYRYPLVDGQGNWGAPDDPKSFAAMRY |
| SRR1157587 | KKSARTVGDVLGKYHPHGDIACYEAMVLMAQPFSYRYPLVDGQGNWGAPDDPKSFAAMRY |
| SRR3027706 | KKSARTVGDVLGKYHPHGDIACYEAMVLMAQPFSYRYPLVDGQGNWGAPDDPKSFAAMRY |
| SRR3027707 | KKSARTVGDVLGKYHPHGDIACYEAMVLMAQPFSYRYPLVDGQGNWGAPDDPKSFAAMRY |
| SRR3027708 | KKSARTVGDVLGKYHPHGDIACYEAMVLMAQPFSYRYPLVDGQGNWGAPDDPKSFAAMRY |
| SRR3027710 | KKSARTVGDVLGKYHPHGDIACYEAMVLMAQPFSYRYPLVDGQGNWGAPDDPKSFAAMRY |
| SRR3027711 | KKSARTVGDVLGKYHPHGDIACYEAMVLMAQPFSYRYPLVDGQGNWGAPDDPKSFAAMRY |
| SRR3027716 | KKSARTVGDVLGKYHPHGDSACYEAMVLMAQPFSYRYPLVDGQGNWGAPDDPKSFAAMRY |
| SRR3027717 | KKSARTVGDVLGKYHPHGDIACYEAMVLMAQPFSYRYPLVDGQGNWGAPDDPKSFAAMRY |
| SRR3027719 | KKSARTVGDVLGKYHPHGDIACYEAMVLMAQPFSYRYPLVDGQGNWGAPDDPKSFAAMRY |
| SRR3027721 | KKSARTVGDVLGKYHPHGDIACYEAMVLMAQPFSYRYPLVDGQGNWGAPDDPKSFAAMRY |
| SRR3027723 | KKSARTVGDVLGKYHPHGDIACYEAMVLMAQPFSYRYPLVDGQGNWGAPDDPKSFAAMRY |
| SRR3115978 | KKSARTVGDVLGKYHPHGDIACYEAMVLMAQPFSYRYPLVDGQGNWGAPDDPKSFAAMRY |
| SRR2534093 | KKSARTVGDVLGKYHPHGDIACYEAMVLMAQPFSYRYPLVDGQGNWGAPDDPKSFAAMRY |
| SRR2534094 | KKSARTVGDVLGKYHPHGDIACYEAMVLMAQPFSYRYPLVDGQGNWGAPDDPKSFAAMRY |
| SRR2534095 | KKSARTVGDVLGKYHPHGDIACYEAMVLMAQPFSYRYPLVDGQGNWGAPDDPKSFAAMRY |
| SRR2534108 | KKSARTVGDVLGKYHPHGDIACYEAMVLMAQPFSYRYPLVDGQGNWGAPDDPKSFAAMRY |
| SRR1106464 | KKSARTVGDVLGKYHPHGDIACYEAMVLMAQPFSYRYPLVDGQGNWGAPDDPKSFAAMRY |
| SRR1106463 | KKSARTVGDVLGKYHPHGDIACYEAMVLMAQPFSYRYPLVDGQGNWGAPDDPKSFAAMRY |
| SRR6949610 | KKSARTVGDVLGKYHPHGDIACYEAMVLMAQPFSYRYPLVDGQGNWGAPDDPKSFAAMRY |
| SRR6950452 | KKSARTVGDVLGKYHPHGDIACYEAMVLMAQPFSYRYPLVDGQGNWGAPDDPKSFAAMRY |
| ERR2019831 | KKSARTVGDVLGKYHPHGDIACYEAMVLMAQPFSYRYPLVDGQGNWGAPDDPKSFAAMRY |
| SRR2085693 | KKSARTVGDVLGKYHPHGDIACYEAMVLMAQPFSYRYPLVDGQGNWGAPDDPKSFAAMRY |
| SRR2086898 | KKSARTVGDVLGKYHPHGDIACYEAMVLMAQPFSYRYPLVDGQGNWGAPDDPKSFAAMRY |
| SRR2175312 | KKSARTVGDVLGKYHPHGDIACYEAMVLMAQPFSYRYPLVDGQGNWGAPDDPKSFAAMRY |
| SRR2175360 | KKSARTVGDVLGKYHPHGDIACYEAMVLMAQPFSYRYPLVDGQGNWGAPDDPKSFAAMRY |
| SRR5231997 | KKSARTVGDVLGKYHPHGDIACYEAMVLMAQPFSYRYPLVDGQGNWGAPDDPKSFAAMRY |
| SRR5232003 | KKSARTVGDVLGKYHPHGDIACYEAMVLMAQPFSYRYPLVDGQGNWGAPDDPKSFAAMRY |
| SRR5232015 | KKSARTVGDVLGKYHPHGDIACYEAMVLMAQPFSYRYPLVDGQGNWGAPDDPKSFAAMRY |
| SRR949434 | KKSARTVGDVLGKYHPHGDIACYEAMVLMAQPFSYRYPLVDGQGNWGAPDDPKSFAAMRY |
| SRR3216575 | KKSARTVGDVLGKYHPHGDIACYEAMVLMAQPFSYRYPLVDGQGNWGAPDDPKSFAAMRY |
| SRR5205342 | KKSARTVGDVLGKYHPHGDIACYEAMVLMAQPFSYRYPLVDGQGNWGAPDDPKSFAAMRY |
| SRR1501669 | KKSARTVGDVLGKYHPHGDIACYEAMVLMAQPFSYRYPLVDGQGNWGAPDDPKSFAAMRY |
| SRR5209740 | KKSARTVGDVLGKYHPHGDIACYEAMVLMAQPFSYRYPLVDGQGNWGAPDDPKSFAAMRY |
| SRR3240355 | KKSARTVGDVLGKYHPHGDIACYEAMVLMAQPFSYRYPLVDGQGNWGAPDDPKSFAAMRY |
| SRR3392777 | KKSARTVGDVLGKYHPHGDIACYEAMVLMAQPFSYRYPLVDGQGNWGAPDDPKSFAAMRY |
| SRR3593671 | KKSARTVGDVLGKYHPHGDIACYEAMVLMAQPFSYRYPLVDGQGNWGAPDDPKSFAAMRY |
| SRR5413290 | KKSARTVGDVLGKYHPHGDIACYEAMVLMAQPFSYRYPLVDGQGNWGAPDDPKSFAAMRY |
| SRR5590269 | KKSARTVGDVLGKYHPHGDIACYEAMVLMAQPFSYRYPLVDGQGNWGAPDDPKSFAAMRY |
| SRR5812103 | KKSARTVGDVLGKYHPHGDIACYEAMVLMAQPFSYRYPLVDGQGNWGAPDDPKSFAAMRY |
| SRR2830941 | KKSARTVGDVLGKYHPHGDIACYEAMVLMAQPFSYRYPLVDGQGNWGAPDDPKSFAAMRY |
| SRR2830966 | KKSARTVGDVLGKYHPHGDIACYEAMVLMAQPFSYRYPLVDGQGNWGAPDDPKSFAAMRY |
| SRR3137270 | KKSARTVGDVLGKYHPHGDIACYEAMVLMAQPFSYRYPLVDGQGNWGAPDDPKSFAAMRY |
| SRR3137271 | KKSARTVGDVLGKYHPHGDIACYEAMVLMAQPFSYRYPLVDGQGNWGAPDDPKSFAAMRY |
| ERR526807 | KKSARTVGDVLGKYHPHGDIACYEAMVLMAQPFSYRYPLVDGQGNWGAPDDPKSFAAMRY |
| ERR2197922 | KKSARTVGDVLGKYHPHGDIACYEAMVLMAQPFSYRYPLVDGQGNWGAPDDPKSFAAMRY |
| ERR2197923 | KKSARTVGDVLGKYHPHGDIACYEAMVLMAQPFSYRYPLVDGQGNWGAPDDPKSFAAMRY |
| ERR2197924 | KKSARTVGDVLGKYHPHGDIACYEAMVLMAQPFSYRYPLVDGQGNWGAPDDPKSFAAMRY |
| ERR2197925 | KKSARTVGDVLGKYHPHGDIACYEAMVLMAQPFSYRYPLVDGQGNWGAPDDPKSFAAMRY |
| ERR2197927 | KKSARTVGDVLGKYHPHGDIACYEAMVLMAQPFSYRYPLVDGQGNWGAPDDPKSFAAMRY |
| ERR2197929 | KKSARTVGDVLGKYHPHGDIACYEAMVLMAQPFSYRYPLVDGQGNWGAPDDPKSFAAMRY |
| SRR1648149 | KKSARTVGDVLGKYHPHGDSACYEAMVLMAQPFSYRYPLVDGQGNWGAPDDPKSFAAMRY |
| SRR1048299 | KKSARTVGDVLGKYHPHGDIACYEAMVLMAQPFSYRYPLVDGQGNWGAPDDPKSFAAMRY |
| SRR1300677 | KKSARTVGDVLGKYHPHGDIACYEAMVLMAQPFSYRYPLVDGQGNWGAPDDPKSFAAMRY |
| SRR1288356 | KKSARTVGDVLGKYHPHGDIACYEAMVLMAQPFSYRYPLVDGQGNWGAPDDPKSFAAMRY |
| SRR7426190 | KKSARTVGDVLGKYHPHGDIACYEAMVLMAQPFSYRYPLVDGQGNWGAPDDPKSFAAMRY |
| SRR7426192 | KKSARTVGDVLGKYHPHGDSACYEAMVLMAQPFSYRYPLVDGQGNWGAPDDPKSFAAMRY |
| SRR7426193 | KKSARTVGDVLGKYHPHGDIACYEAMVLMAQPFSYRYPLVDGQGNWGAPDDPKSFAAMRY |
| SRR7441832 | KKSARTVGDVLGKYHPHGDIACYEAMVLMAQPFSYRYPLVDGQGNWGAPDDPKSFAAMRY |
| SRR7426179 | KKSARTVGDVLGKYHPHGDIACYEAMVLMAQPFSYRYPLVDGQGNWGAPDDPKSFAAMRY |
| SRR7439238 | KKSARTVGDVLGKYHPHGDIACYEAMVLMAQPFSYRYPLVDGQGNWGAPDDPKSFAAMRY |
| SRR7439244 | KKSARTVGDVLGKYHPHGDIACYEAMVLMAQPFSYRYPLVDGQGNWGAPDDPKSFAAMRY |
| SRR7439259 | KKSARTVGDVLGKYHPHGDIACYEAMVLMAQPFSYRYPLVDGQGNWGAPDDPKSFAAMRY |
| SRR7439260 | KKSARTVGDVLGKYHPHGDIACYEAMVLMAQPFSYRYPLVDGQGNWGAPDDPKSFAAMRY |
| SRR7441786 | KKSARTVGDVLGKYHPHGDIACYEAMVLMAQPFSYRYPLVDGQGNWGAPDDPKSFAAMRY |
| SRR7441797 | KKSARTVGDVLGKYHPHGDIACYEAMVLMAQPFSYRYPLVDGQGNWGAPDDPKSFAAMRY |
| ERR1759093 | KKSARTVGDVLGKYHPHGDIACYEAMVLMAQPFSYRYPLVDGQGNWGAPDDPKSFAAMRY |
| ERR2580275 | KKSARTVGDVLGKYHPHGDIACYEAMVLMAQPFSYRYPLVDGQGNWGAPDDPKSFAAMRY |
| ERR1759204 | KKSARTVGDVLGKYHPHGDIACYEAMVLMAQPFSYRYPLVDGQGNWGAPDDPKSFAAMRY |
| SRR1300699 | KKSARTVGDVLGKYHPHGDIACYEAMVLMAQPFSYRYPLVDGQGNWGAPDDPKSFAAMRY |
| S_0825_17 | KKSARTVGDVLGKYHPHGDIACYEAMVLMAQPFSYRYPLVDGQGNWGAPDDPKSFAAMRY |
| SRR1958215 | KKSARTVGDVLGKYHPHGDIACYEAMVLMAQPFSYRYPLVDGQGNWGAPDDPKSFAAMRY |
| SRR1958540 | KKSARTVGDVLGKYHPHGDIACYEAMVLMAQPFSYRYPLVDGQGNWGAPDDPKSFAAMRY |
| SRR1958636 | KKSARTVGDVLGKYHPHGDIACYEAMVLMAQPFSYRYPLVDGQGNWGAPDDPKSFAAMRY |
| SRR1959422 | KKSARTVGDVLGKYHPHGDIACYEAMVLMAQPFSYRYPLVDGQGNWGAPDDPKSFAAMRY |
| SRR1959427 | KKSARTVGDVLGKYHPHGDIACYEAMVLMAQPFSYRYPLVDGQGNWGAPDDPKSFAAMRY |
| SRR1960226 | KKSARTVGDVLGKYHPHGDIACYEAMVLMAQPFSYRYPLVDGQGNWGAPDDPKSFAAMRY |
| SRR1963498 | KKSARTVGDVLGKYHPHGDIACYEAMVLMAQPFSYRYPLVDGQGNWGAPDDPKSFAAMRY |
| SRR1965947 | KKSARTVGDVLGKYHPHGDIACYEAMVLMAQPFSYRYPLVDGQGNWGAPDDPKSFAAMRY |
| SRR1966125 | KKSARTVGDVLGKYHPHGDIACYEAMVLMAQPFSYRYPLVDGQGNWGAPDDPKSFAAMRY |
| SRR1966330 | KKSARTVGDVLGKYHPHGDIACYEAMVLMAQPFSYRYPLVDGQGNWGAPDDPKSFAAMRY |
| SRR1966565 | KKSARTVGDVLGKYHPHGDIACYEAMVLMAQPFSYRYPLVDGQGNWGAPDDPKSFAAMRY |
| SRR1966864 | KKSARTVGDVLGKYHPHGDIACYEAMVLMAQPFSYRYPLVDGQGNWGAPDDPKSFAAMRY |
| SRR1966989 | KKSARTVGDVLGKYHPHGDIACYEAMVLMAQPFSYRYPLVDGQGNWGAPDDPKSFAAMRY |
| SRR1967688 | KKSARTVGDVLGKYHPHGDIACYEAMVLMAQPFSYRYPLVDGQGNWGAPDDPKSFAAMRY |
| SRR1967733 | KKSARTVGDVLGKYHPHGDIACYEAMVLMAQPFSYRYPLVDGQGNWGAPDDPKSFAAMRY |
| SRR1967746 | KKSARTVGDVLGKYHPHGDIACYEAMVLMAQPFSYRYPLVDGQGNWGAPDDPKSFAAMRY |
| SRR1968341 | KKSARTVGDVLGKYHPHGDIACYEAMVLMAQPFSYRYPLVDGQGNWGAPDDPKSFAAMRY |
| SRR1968456 | KKSARTVGDVLGKYHPHGDIACYEAMVLMAQPFSYRYPLVDGQGNWGAPDDPKSFAAMRY |
| SRR1968465 | KKSARTVGDVLGKYHPHGDIACYEAMVLMAQPFSYRYPLVDGQGNWGAPDDPKSFAAMRY |
| SRR1968761 | KKSARTVGDVLGKYHPHGDIACYEAMVLMAQPFSYRYPLVDGQGNWGAPDDPKSFAAMRY |
| SRR1969047 | KKSARTVGDVLGKYHPHGDIACYEAMVLMAQPFSYRYPLVDGQGNWGAPDDPKSFAAMRY |
| SRR1969255 | KKSARTVGDVLGKYHPHGDIACYEAMVLMAQPFSYRYPLVDGQGNWGAPDDPKSFAAMRY |
| SRR1969412 | KKSARTVGDVLGKYHPHGDIACYEAMVLMAQPFSYRYPLVDGQGNWGAPDDPKSFAAMRY |
| SRR1969524 | KKSARTVGDVLGKYHPHGDIACYEAMVLMAQPFSYRYPLVDGQGNWGAPDDPKSFAAMRY |
| SRR1969584 | KKSARTVGDVLGKYHPHGDIACYEAMVLMAQPFSYRYPLVDGQGNWGAPDDPKSFAAMRY |
| SRR1969648 | KKSARTVGDVLGKYHPHGDIACYEAMVLMAQPFSYRYPLVDGQGNWGAPDDPKSFAAMRY |
| SRR1969804 | KKSARTVGDVLGKYHPHGDIACYEAMVLMAQPFSYRYPLVDGQGNWGAPDDPKSFAAMRY |
| SRR1970221 | KKSARTVGDVLGKYHPHGDIACYEAMVLMAQPFSYRYPLVDGQGNWGAPDDPKSFAAMRY |
| SRR1970268 | KKSARTVGDVLGKYHPHGDIACYEAMVLMAQPFSYRYPLVDGQGNWGAPDDPKSFAAMRY |
| SRR1965862 | KKSARTVGDVLGKYHPHGDIACYEAMVLMAQPFSYRYPLVDGQGNWGAPDDPKSFAAMRY |
| SRR1967363 | KKSARTVGDVLGKYHPHGDIACYEAMVLMAQPFSYRYPLVDGQGNWGAPDDPKSFAAMRY |
| SRR1968276 | KKSARTVGDVLGKYHPHGDIACYEAMVLMAQPFSYRYPLVDGQGNWGAPDDPKSFAAMRY |
| SRR1968967 | KKSARTVGDVLGKYHPHGDIACYEAMVLMAQPFSYRYPLVDGQGNWGAPDDPKSFAAMRY |
| SRR3321531 | KKSARTVGDVLGKYHPHGDIACYEAMVLMAQPFSYRYPLVDGQGNWGAPDDPKSFAAMRY |
| SRR3321883 | KKSARTVGDVLGKYHPHGDIACYEAMVLMAQPFSYRYPLVDGQGNWGAPDDPKSFAAMRY |
| SRR3322413 | KKSARTVGDVLGKYHPHGDIACYEAMVLMAQPFSYRYPLVDGQGNWGAPDDPKSFAAMRY |
| SRR3323012 | KKSARTVGDVLGKYHPHGDIACYEAMVLMAQPFSYRYPLVDGQGNWGAPDDPKSFAAMRY |
| SRR5194289 | KKSARTVGDVLGKYHPHGDIACYEAMVLMAQPFSYRYPLVDGQGNWGAPDDPKSFAAMRY |
| SRR7163798 | KKSARTVGDVLGKYHPHGDIACYEAMVLMAQPFSYRYPLVDGQGNWGAPDDPKSFAAMRY |
| SRR7172610 | KKSARTVGDVLGKYHPHGDIACYEAMVLMAQPFSYRYPLVDGQGNWGAPDDPKSFAAMRY |
| SRR7204568 | KKSARTVGDVLGKYHPHGDIACYEAMVLMAQPFSYRYPLVDGQGNWGAPDDPKSFAAMRY |
| SRR7223230 | KKSARTVGDVLGKYHPHGDIACYEAMVLMAQPFSYRYPLVDGQGNWGAPDDPKSFAAMRY |
| SRR7230675 | KKSARTVGDVLGKYHPHGDIACYEAMVLMAQPFSYRYPLVDGQGNWGAPDDPKSFAAMRY |
| SRR7278056 | KKSARTVGDVLGKYHPHGDIACYEAMVLMAQPFSYRYPLVDGQGNWGAPDDPKSFAAMRY |
| SRR7278086 | KKSARTVGDVLGKYHPHGDIACYEAMVLMAQPFSYRYPLVDGQGNWGAPDDPKSFAAMRY |
| SRR7285841 | KKSARTVGDVLGKYHPHGDIACYEAMVLMAQPFSYRYPLVDGQGNWGAPDDPKSFAAMRY |
| SRR7292625 | KKSARTVGDVLGKYHPHGDIACYEAMVLMAQPFSYRYPLVDGQGNWGAPDDPKSFAAMRY |
| SRR7292665 | KKSARTVGDVLGKYHPHGDIACYEAMVLMAQPFSYRYPLVDGQGNWGAPDDPKSFAAMRY |
| SRR7297965 | KKSARTVGDVLGKYHPHGDIACYEAMVLMAQPFSYRYPLVDGQGNWGAPDDPKSFAAMRY |
| SRR7350726 | KKSARTVGDVLGKYHPHGDIACYEAMVLMAQPFSYRYPLVDGQGNWGAPDDPKSFAAMRY |
| SRR7410328 | KKSARTVGDVLGKYHPHGDIACYEAMVLMAQPFSYRYPLVDGQGNWGAPDDPKSFAAMRY |
| SRR7474665 | KKSARTVGDVLGKYHPHGDIACYEAMVLMAQPFSYRYPLVDGQGNWGAPDDPKSFAAMRY |
| SRR7523184 | KKSARTVGDVLGKYHPHGDIACYEAMVLMAQPFSYRYPLVDGQGNWGAPDDPKSFAAMRY |
| SRR7187264 | KKSARTVGDVLGKYHPHGDIACYEAMVLMAQPFSYRYPLVDGQGNWGAPDDPKSFAAMRY |
| SRR7204445 | KKSARTVGDVLGKYHPHGDIACYEAMVLMAQPFSYRYPLVDGQGNWGAPDDPKSFAAMRY |
| SRR7285641 | KKSARTVGDVLGKYHPHGDIACYEAMVLMAQPFSYRYPLVDGQGNWGAPDDPKSFAAMRY |
| SRR7286695 | KKSARTVGDVLGKYHPHGDIACYEAMVLMAQPFSYRYPLVDGQGNWGAPDDPKSFAAMRY |
| SRR7286705 | KKSARTVGDVLGKYHPHGDIACYEAMVLMAQPFSYRYPLVDGQGNWGAPDDPKSFAAMRY |
| SRR7292931 | KKSARTVGDVLGKYHPHGDIACYEAMVLMAQPFSYRYPLVDGQGNWGAPDDPKSFAAMRY |
| SRR7310349 | KKSARTVGDVLGKYHPHGDIACYEAMVLMAQPFSYRYPLVDGQGNWGAPDDPKSFAAMRY |
| SRR7351616 | KKSARTVGDVLGKYHPHGDIACYEAMVLMAQPFSYRYPLVDGQGNWGAPDDPKSFAAMRY |
| SRR7414818 | KKSARTVGDVLGKYHPHGDIACYEAMVLMAQPFSYRYPLVDGQGNWGAPDDPKSFAAMRY |
| SRR7426480 | KKSARTVGDVLGKYHPHGDIACYEAMVLMAQPFSYRYPLVDGQGNWGAPDDPKSFAAMRY |
| SRR5584105 | KKSARTVGDVLGKYHPHGDIACYEAMVLMAQPFSYRYPLVDGQGNWGAPDDPKSFAAMRY |
| SRR5584565 | KKSARTVGDVLGKYHPHGDIACYEAMVLMAQPFSYRYPLVDGQGNWGAPDDPKSFAAMRY |
| SRR5584614 | KKSARTVGDVLGKYHPHGDIACYEAMVLMAQPFSYRYPLVDGQGNWGAPDDPKSFAAMRY |
| SRR5631543 | KKSARTVGDVLGKYHPHGDIACYEAMVLMAQPFSYRYPLVDGQGNWGAPDDPKSFAAMRY |
| SRR5631553 | KKSARTVGDVLGKYHPHGDIACYEAMVLMAQPFSYRYPLVDGQGNWGAPDDPKSFAAMRY |
| SRR7123196 | KKSARTVGDVLGKYHPHGDIACYEAMVLMAQPFSYRYPLVDGQGNWGAPDDPKSFAAMRY |
| SRR7163819 | KKSARTVGDVLGKYHPHGDIACYEAMVLMAQPFSYRYPLVDGQGNWGAPDDPKSFAAMRY |
| SRR7163920 | KKSARTVGDVLGKYHPHGDIACYEAMVLMAQPFSYRYPLVDGQGNWGAPDDPKSFAAMRY |
| SRR7209528 | KKSARTVGDVLGKYHPHGDIACYEAMVLMAQPFSYRYPLVDGQGNWGAPDDPKSFAAMRY |
| SRR7249868 | KKSARTVGDVLGKYHPHGDIACYEAMVLMAQPFSYRYPLVDGQGNWGAPDDPKSFAAMRY |
| SRR7278088 | KKSARTVGDVLGKYHPHGDIACYEAMVLMAQPFSYRYPLVDGQGNWGAPDDPKSFAAMRY |
| SRR7285788 | KKSARTVGDVLGKYHPHGDIACYEAMVLMAQPFSYRYPLVDGQGNWGAPDDPKSFAAMRY |
| SRR7286789 | KKSARTVGDVLGKYHPHGDIACYEAMVLMAQPFSYRYPLVDGQGNWGAPDDPKSFAAMRY |
| SRR7286886 | KKSARTVGDVLGKYHPHGDIACYEAMVLMAQPFSYRYPLVDGQGNWGAPDDPKSFAAMRY |
| SRR7310632 | KKSARTVGDVLGKYHPHGDIACYEAMVLMAQPFSYRYPLVDGQGNWGAPDDPKSFAAMRY |
| SRR7350631 | KKSARTVGDVLGKYHPHGDIACYEAMVLMAQPFSYRYPLVDGQGNWGAPDDPKSFAAMRY |
| SRR7458741 | KKSARTVGDVLGKYHPHGDIACYEAMVLMAQPFSYRYPLVDGQGNWGAPDDPKSFAAMRY |
| SRR7480280 | KKSARTVGDVLGKYHPHGDIACYEAMVLMAQPFSYRYPLVDGQGNWGAPDDPKSFAAMRY |
| SRR7523660 | KKSARTVGDVLGKYHPHGDIACYEAMVLMAQPFSYRYPLVDGQGNWGAPDDPKSFAAMRY |
| SRR7523775 | KKSARTVGDVLGKYHPHGDIACYEAMVLMAQPFSYRYPLVDGQGNWGAPDDPKSFAAMRY |
| SRR7251101 | KKSARTVGDVLGKYHPHGDIACYEAMVLMAQPFSYRYPLVDGQGNWGAPDDPKSFAAMRY |
| SRR7284299 | KKSARTVGDVLGKYHPHGDIACYEAMVLMAQPFSYRYPLVDGQGNWGAPDDPKSFAAMRY |
| SRR7285738 | KKSARTVGDVLGKYHPHGDIACYEAMVLMAQPFSYRYPLVDGQGNWGAPDDPKSFAAMRY |
| SRR7310640 | KKSARTVGDVLGKYHPHGDIACYEAMVLMAQPFSYRYPLVDGQGNWGAPDDPKSFAAMRY |
| SRR7349159 | KKSARTVGDVLGKYHPHGDIACYEAMVLMAQPFSYRYPLVDGQGNWGAPDDPKSFAAMRY |
| SRR7474873 | KKSARTVGDVLGKYHPHGDIACYEAMVLMAQPFSYRYPLVDGQGNWGAPDDPKSFAAMRY |
| SRR7495689 | KKSARTVGDVLGKYHPHGDIACYEAMVLMAQPFSYRYPLVDGQGNWGAPDDPKSFAAMRY |
| SRR7495752 | KKSARTVGDVLGKYHPHGDIACYEAMVLMAQPFSYRYPLVDGQGNWGAPDDPKSFAAMRY |
| ------------------------------------------------------------------------------ | |
| S16BD08730 | TESRLSKYAELLLSELGQGTADWVPNFDGTMQEPKMLPARLPNILLNGTTGIAVGMATDI |
| S18BD00684 | TESRLSKYAELLLSELGQGTADWVPNFDGTMQEPKMLPARLPNILLNGTTGIAVGMATDI |
| S18BD03994 | TESRLSKYAELLLSELGQGTADWVPNFDGTMQEPKMLPARLPNILLNGTTGIAVGMATDI |
| S18BD05011 | TESRLSKYAELLLSELGQGTADWVPNFDGTMQEPKMLPARLPNILLNGTTGIAVGMATDI |
| RKI_16-03723 | TESRLSKYAELLLSELGQGTADWVPNFDGTMQEPKMLPARLPNILLNGTTGIAVGMATDI |
| RKI_16-04315 | TESRLSKYAELLLSELGQGTADWVPNFDGTMQEPKMLPARLPNILLNGTTGIAVGMATDI |
| RKI_17-02304 | TESRLSKYAELLLSELGQGTADWVPNFDGTMQEPKMLPARLPNILLNGTTGIAVGMATDI |
| RKI_17-02411 | TESRLSKYAELLLSELGQGTADWVPNFDGTMQEPKMLPARLPNILLNGTTGIAVGMATDI |
| RKI_17-02757 | TESRLSKYAELLLSELGQGTADWVPNFDGTMQEPKMLPARLPNILLNGTTGIAVGMATDI |
| RKI_17-04797 | TESRLSKYAELLLSELGQGTADWVPNFDGTMQEPKMLPARLPNILLNGTTGIAVGMATDI |
| RKI_17-06869 | TESRLSKYAELLLSELGQGTADWVPNFDGTMQEPKMLPARLPNILLNGTTGIAVGMATDI |
| ERR2580277 | TESRLSKYAELLLSELGQGTADWVPNFDGTMQEPKMLPARLPNILLNGTTGIAVGMATDI |
| ERR2580276 | TESRLSKYAELLLSELGQGTADWVPNFDGTMQEPKMLPARLPNILLNGTTGIAVGMATDI |
| ERR2580273 | TESRLSKYAELLLSELGQGTADWVPNFDGTMQEPKMLPARLPNILLNGTTGIAVGMATDI |
| ERR2580274 | TESRLSKYAELLLSELGQGTADWVPNFDGTMQEPKMLPARLPNILLNGTTGIAVGMATDI |
| ERR2173656 | TESRLSKYAELLLSELGQGTADWVPNFDGTMQEPKMLPARLPNILLNGTTGIAVGMATDI |
| 17041676 | TESRLSKYAELLLSELGQGTADWVPNFDGTMQEPKMLPARLPNILLNGTTGIAVGMATDI |
| MT16-000061 | TESRLSKYAELLLSELGQGTADWVPNFDGTMQEPKMLPARLPNILLNGTTGIAVGMATDI |
| MT16-019416 | TESRLSKYAELLLSELGQGTADWVPNFDGTMQEPKMLPARLPNILLNGTTGIAVGMATDI |
| MT16-027865 | TESRLSKYAELLLSELGQGTADWVPNFDGTMQEPKMLPARLPNILLNGTTGIAVGMATDI |
| MT16-031693 | TESRLSKYAELLLSELGQGTADWVPNFDGTMQEPKMLPARLPNILLNGTTGIAVGMATDI |
| MT16-040253 | TESRLSKYAELLLSELGQGTADWVPNFDGTMQEPKMLPARLPNILLNGTTGIAVGMATDI |
| MT16-045379 | TESRLSKYAELLLSELGQGTADWVPNFDGTMQEPKMLPARLPNILLNGTTGIAVGMATDI |
| MT16-442728 | TESRLSKYAELLLSELGQGTADWVPNFDGTMQEPKMLPARLPNILLNGTTGIAVGMATDI |
| MT16-462857 | TESRLSKYAELLLSELGQGTADWVPNFDGTMQEPKMLPARLPNILLNGTTGIAVGMATDI |
| MT16-480196 | TESRLSKYAELLLSELGQGTADWVPNFDGTMQEPKMLPARLPNILLNGTTGIAVGMATDI |
| MT16-861555 | TESRLSKYAELLLSELGQGTADWVPNFDGTMQEPKMLPARLPNILLNGTTGIAVGMATDI |
| MT17-076833 | TESRLSKYAELLLSELGQGTADWVPNFDGTMQEPKMLPARLPNILLNGTTGIAVGMATDI |
| MT17-110677 | TESRLSKYAELLLSELGQGTADWVPNFDGTMQEPKMLPARLPNILLNGTTGIAVGMATDI |
| MT17-131730 | TESRLSKYAELLLSELGQGTADWVPNFDGTMQEPKMLPARLPNILLNGTTGIAVGMATDI |
| MT17-140890 | TESRLSKYAELLLSELGQGTADWVPNFDGTMQEPKMLPARLPNILLNGTTGIAVGMATDI |
| MT17-141840 | TESRLSKYAELLLSELGQGTADWVPNFDGTMQEPKMLPARLPNILLNGTTGIAVGMATDI |
| MT17-152488 | TESRLSKYAELLLSELGQGTADWVPNFDGTMQEPKMLPARLPNILLNGTTGIAVGMATDI |
| MT17-157311 | TESRLSKYAELLLSELGQGTADWVPNFDGTMQEPKMLPARLPNILLNGTTGIAVGMATDI |
| MT17-161645 | TESRLSKYAELLLSELGQGTADWVPNFDGTMQEPKMLPARLPNILLNGTTGIAVGMATDI |
| MT17-167951 | TESRLSKYAELLLSELGQGTADWVPNFDGTMQEPKMLPARLPNILLNGTTGIAVGMATDI |
| MT18-217732 | TESRLSKYAELLLSELGQGTADWVPNFDGTMQEPKMLPARLPNILLNGTTGIAVGMATDI |
| MT18-252580 | TESRLSKYAELLLSELGQGTADWVPNFDGTMQEPKMLPARLPNILLNGTTGIAVGMATDI |
| RIVM_H_2009-01 | TESRLSKYAELLLSELGQGTADWVPNFDGTMQEPKMLPARLPNILLNGTTGIAVGMATDI |
| RIVM_H_2010-01 | TESRLSKYAELLLSELGQGTADWVPNFDGTMQEPKMLPARLPNILLNGTTGIAVGMATDI |
| RIVM_H_2010-02 | TESRLSKYAELLLSELGQGTADWVPNFDGTMQEPKMLPARLPNILLNGTTGIAVGMATDI |
| RIVM_H_2011-01 | TESRLSKYAELLLSELGQGTADWVPNFDGTMQEPKMLPARLPNILLNGTTGIAVGMATDI |
| RIVM_H_2011-02 | TESRLSKYAELLLSELGQGTADWVPNFDGTMQEPKMLPARLPNILLNGTTGIAVGMATDI |
| RIVM_H_2011-03 | TESRLSKYAELLLSELGQGTADWVPNFDGTMQEPKMLPARLPNILLNGTTGIAVGMATDI |
| RIVM_H_2013-01 | TESRLSKYAELLLSELGQGTADWVPNFDGTMQEPKMLPARLPNILLNGTTGIAVGMATDI |
| RIVM_H_2013-02 | TESRLSKYAELLLSELGQGTADWVPNFDGTMQEPKMLPARLPNILLNGTTGIAVGMATDI |
| RIVM_H_2014-01 | TESRLSKYAELLLSELGQGTADWVPNFDGTMQEPKMLPARLPNILLNGTTGIAVGMATDI |
| RIVM_H_2014-02 | TESRLSKYAELLLSELGQGTADWVPNFDGTMQEPKMLPARLPNILLNGTTGIAVGMATDI |
| RIVM_H_2016-01 | TESRLSKYAELLLSELGQGTADWVPNFDGTMQEPKMLPARLPNILLNGTTGIAVGMATDI |
| RIVM_H_2016-02 | TESRLSKYAELLLSELGQGTADWVPNFDGTMQEPKMLPARLPNILLNGTTGIAVGMATDI |
| RIVM_H_2016-03 | TESRLSKYAELLLSELGQGTADWVPNFDGTMQEPKMLPARLPNILLNGTTGIAVGMATDI |
| RIVM_H_2016-04 | TESRLSKYAELLLSELGQGTADWVPNFDGTMQEPKMLPARLPNILLNGTTGIAVGMATDI |
| RIVM_H_2016-05 | TESRLSKYAELLLSELGQGTADWVPNFDGTMQEPKMLPARLPNILLNGTTGIAVGMATDI |
| RIVM_H_2016-06 | TESRLSKYAELLLSELGQGTADWVPNFDGTMQEPKMLPARLPNILLNGTTGIAVGMATDI |
| RIVM_H_2016-07 | TESRLSKYAELLLSELGQGTADWVPNFDGTMQEPKMLPARLPNILLNGTTGIAVGMATDI |
| RIVM_H_2016-08 | TESRLSKYAELLLSELGQGTADWVPNFDGTMQEPKMLPARLPNILLNGTTGIAVGMATDI |
| RIVM_H_2016-09 | TESRLSKYAELLLSELGQGTADWVPNFDGTMQEPKMLPARLPNILLNGTTGIAVGMATDI |
| RIVM_H_2016-10 | TESRLSKYAELLLSELGQGTADWVPNFDGTMQEPKMLPARLPNILLNGTTGIAVGMATDI |
| RIVM_H_2016-11 | TESRLSKYAELLLSELGQGTADWVPNFDGTMQEPKMLPARLPNILLNGTTGIAVGMATDI |
| RIVM_H_2016-12 | TESRLSKYAELLLSELGQGTADWVPNFDGTMQEPKMLPARLPNILLNGTTGIAVGMATDI |
| RIVM_H_2016-13 | TESRLSKYAELLLSELGQGTADWVPNFDGTMQEPKMLPARLPNILLNGTTGIAVGMATDI |
| RIVM_H_2016-14 | TESRLSKYAELLLSELGQGTADWVPNFDGTMQEPKMLPARLPNILLNGTTGIAVGMATDI |
| RIVM_H_2016-15 | TESRLSKYAELLLSELGQGTADWVPNFDGTMQEPKMLPARLPNILLNGTTGIAVGMATDI |
| RIVM_H_2017-01 | TESRLSKYAELLLSELGQGTADWVPNFDGTMQEPKMLPARLPNILLNGTTGIAVGMATDI |
| RIVM_H_2017-02 | TESRLSKYAELLLSELGQGTADWVPNFDGTMQEPKMLPARLPNILLNGTTGIAVGMATDI |
| RIVM_H_2017-03 | TESRLSKYAELLLSELGQGTADWVPNFDGTMQEPKMLPARLPNILLNGTTGIAVGMATDI |
| RIVM_H_2017-04 | TESRLSKYAELLLSELGQGTADWVPNFDGTMQEPKMLPARLPNILLNGTTGIAVGMATDI |
| RIVM_H_2017-05 | TESRLSKYAELLLSELGQGTADWVPNFDGTMQEPKMLPARLPNILLNGTTGIAVGMATDI |
| RIVM_H_2017-06 | TESRLSKYAELLLSELGQGTADWVPNFDGTMQEPKMLPARLPNILLNGTTGIAVGMATDI |
| RIVM_H_2017-07 | TESRLSKYAELLLSELGQGTADWVPNFDGTMQEPKMLPARLPNILLNGTTGIAVGMATDI |
| RIVM_H_2017-08 | TESRLSKYAELLLSELGQGTADWVPNFDGTMQEPKMLPARLPNILLNGTTGIAVGMATDI |
| RIVM_H_2017-09 | TESRLSKYAELLLSELGQGTADWVPNFDGTMQEPKMLPARLPNILLNGTTGIAVGMATDI |
| RIVM_H_2017-10 | TESRLSKYAELLLSELGQGTADWVPNFDGTMQEPKMLPARLPNILLNGTTGIAVGMATDI |
| RIVM_H_2017-11 | TESRLSKYAELLLSELGQGTADWVPNFDGTMQEPKMLPARLPNILLNGTTGIAVGMATDI |
| RIVM_H_2017-12 | TESRLSKYAELLLSELGQGTADWVPNFDGTMQEPKMLPARLPNILLNGTTGIAVGMATDI |
| RIVM_H_2017-13 | TESRLSKYAELLLSELGQGTADWVPNFDGTMQEPKMLPARLPNILLNGTTGIAVGMATDI |
| RIVM_H_2017-14 | TESRLSKYAELLLSELGQGTADWVPNFDGTMQEPKMLPARLPNILLNGTTGIAVGMATDI |
| RIVM_H_2017-15 | TESRLSKYAELLLSELGQGTADWVPNFDGTMQEPKMLPARLPNILLNGTTGIAVGMATDI |
| RIVM_H_2017-16 | TESRLSKYAELLLSELGQGTADWVPNFDGTMQEPKMLPARLPNILLNGTTGIAVGMATDI |
| RIVM_H_2017-17 | TESRLSKYAELLLSELGQGTADWVPNFDGTMQEPKMLPARLPNILLNGTTGIAVGMATDI |
| RIVM_H_2017-18 | TESRLSKYAELLLSELGQGTADWVPNFDGTMQEPKMLPARLPNILLNGTTGIAVGMATDI |
| RIVM_H_2017-19 | TESRLSKYAELLLSELGQGTADWVPNFDGTMQEPKMLPARLPNILLNGTTGIAVGMATDI |
| 15EP001483 | TESRLSKYAELLLSELGQGTADWVPNFDGTMQEPKMLPARLPNILLNGTTGIAVGMATDI |
| 17EP002363 | TESRLSKYAELLLSELGQGTADWVPNFDGTMQEPKMLPARLPNILLNGTTGIAVGMATDI |
| S_0812_17 | TESRLSKYAELLLSELGQGTADWVPNFDGTMQEPKMLPARLPNILLNGTTGIAVGMATDI |
| SRR1957844 | TESRLSKYAELLLSELGQGTADWVPNFDGTMQEPKMLPARLPNILLNGTTGIAVGMATDI |
| SRR1958654 | TESRLSKYAELLLSELGQGTADWVPNFDGTMQEPKMLPARLPNILLNGTTGIAVGMATDI |
| SRR1965077 | TESRLSKYAELLLSELGQGTADWVPNFDGTMQEPKMLPARLPNILLNGTTGIAVGMATDI |
| SRR1966369 | TESRLSKYAELLLSELGQGTADWVPNFDGTMQEPKMLPARLPNILLNGTTGIAVGMATDI |
| SRR1967117 | TESRLSKYAELLLSELGQGTADWVPNFDGTMQEPKMLPARLPNILLNGTTGIAVGMATDI |
| SRR1967922 | TESRLSKYAELLLSELGQGTADWVPNFDGTMQEPKMLPARLPNILLNGTTGIAVGMATDI |
| SRR8704720 | TESRLSKYAELLLSELGQGTADWVPNFDGTMQEPKMLPARLPNILLNGTTGIAVGMATDI |
| SRR7216071 | TESRLSKYAELLLSELGQGTADWVPNFDGTMQEPKMLPARLPNILLNGTTGIAVGMATDI |
| SRR7349175 | TESRLSKYAELLLSELGQGTADWVPNFDGTMQEPKMLPARLPNILLNGTTGIAVGMATDI |
| SRR7523148 | TESRLSKYAELLLSELGQGTADWVPNFDGTMQEPKMLPARLPNILLNGTTGIAVGMATDI |
| SRR7523854 | TESRLSKYAELLLSELGQGTADWVPNFDGTMQEPKMLPARLPNILLNGTTGIAVGMATDI |
| 313865 | TESRLSKYAELLLSELGQGTADWVPNFDGTMQEPKMLPARLPNILLNGTTGIAVGMATDI |
| SRR7277793 | TESRLSKYAELLLSELGQGTADWVPNFDGTMQEPKMLPARLPNILLNGTTGIAVGMATDI |
| SRR7343877 | TESRLSKYAELLLSELGQGTADWVPNFDGTMQEPKMLPARLPNILLNGTTGIAVGMATDI |
| SRR7351477 | TESRLSKYAELLLSELGQGTADWVPNFDGTMQEPKMLPARLPNILLNGTTGIAVGMATDI |
| SRR5583183 | TESRLSKYAELLLSELGQGTADWVPNFDGTMQEPKMLPARLPNILLNGTTGIAVGMATDI |
| SRR5585240 | TESRLSKYAELLLSELGQGTADWVPNFDGTMQEPKMLPARLPNILLNGTTGIAVGMATDI |
| SRR7284317 | TESRLSKYAELLLSELGQGTADWVPNFDGTMQEPKMLPARLPNILLNGTTGIAVGMATDI |
| SRR7299161 | TESRLSKYAELLLSELGQGTADWVPNFDGTMQEPKMLPARLPNILLNGTTGIAVGMATDI |
| SRR7401730 | TESRLSKYAELLLSELGQGTADWVPNFDGTMQEPKMLPARLPNILLNGTTGIAVGMATDI |
| SRR7469092 | TESRLSKYAELLLSELGQGTADWVPNFDGTMQEPKMLPARLPNILLNGTTGIAVGMATDI |
| SRR7879556 | TESRLSKYAELLLSELGQGTADWVPNFDGTMQEPKMLPARLPNILLNGTTGIAVGMATDI |
| SRR8526100 | TESRLSKYAELLLSELGQGTADWVPNFDGTMQEPKMLPARLPNILLNGTTGIAVGMATDI |
| SRR8553991 | TESRLSKYAELLLSELGQGTADWVPNFDGTMQEPKMLPARLPNILLNGTTGIAVGMATDI |
| SRR7842487 | TESRLSKYAELLLSELGQGTADWVPNFDGTMQEPKMLPARLPNILLNGTTGIAVGMATDI |
| SRR8054524 | TESRLSKYAELLLSELGQGTADWVPNFDGTMQEPKMLPARLPNILLNGTTGIAVGMATDI |
| SRR8054525 | TESRLSKYAELLLSELGQGTADWVPNFDGTMQEPKMLPARLPNILLNGTTGIAVGMATDI |
| SRR8524733 | TESRLSKYAELLLSELGQGTADWVPNFDGTMQEPKMLPARLPNILLNGTTGIAVGMATDI |
| SRR4093291 | TESRLSKYAELLLSELGQGTADWVPNFDGTMQEPKMLPARLPNILLNGTTGIAVGMATDI |
| SRR4245549 | TESRLSKYAELLLSELGQGTADWVPNFDGTMQEPKMLPARLPNILLNGTTGIAVGMATDI |
| SRR3057154 | TESRLSKYAELLLSELGQGTADWVPNFDGTMQEPKMLPARLPNILLNGTTGIAVGMATDI |
| SRR1726150 | TESRLSKYAELLLSELGQGTADWVPNFDGTMQEPKMLPARLPNILLNGTTGIAVGMATDI |
| SRR1996141 | TESRLSKYAELLLSELGQGTADWVPNFDGTMQEPKMLPARLPNILLNGTTGIAVGMATDI |
| SRR1107842 | TESRLSKYAELLLSELGQGTADWVPNFDGTMQEPKMLPARLPNILLNGTTGIAVGMATDI |
| SRR1157587 | TESRLSKYAELLLSELGQGTADWVPNFDGTMQEPKMLPARLPNILLNGTTGIAVGMATDI |
| SRR3027706 | TESRLSKYAELLLSELGQGTADWVPNFDGTMQEPKMLPARLPNILLNGTTGIAVGMATDI |
| SRR3027707 | TESRLSKYAELLLSELGQGTADWVPNFDGTMQEPKMLPARLPNILLNGTTGIAVGMATDI |
| SRR3027708 | TESRLSKYAELLLSELGQGTADWVPNFDGTMQEPKMLPARLPNILLNGTTGIAVGMATDI |
| SRR3027710 | TESRLSKYAELLLSELGQGTADWVPNFDGTMQEPKMLPARLPNILLNGTTGIAVGMATDI |
| SRR3027711 | TESRLSKYAELLLSELGQGTADWVPNFDGTMQEPKMLPARLPNILLNGTTGIAVGMATDI |
| SRR3027716 | TESRLSKYAELLLSELGQGTADWVPNFDGTMQEPKMLPARLPNILLNGTTGIAVGMATDI |
| SRR3027717 | TESRLSKYAELLLSELGQGTADWVPNFDGTMQEPKMLPARLPNILLNGTTGIAVGMATDI |
| SRR3027719 | TESRLSKYAELLLSELGQGTADWVPNFDGTMQEPKMLPARLPNILLNGTTGIAVGMATDI |
| SRR3027721 | TESRLSKYAELLLSELGQGTADWVPNFDGTMQEPKMLPARLPNILLNGTTGIAVGMATDI |
| SRR3027723 | TESRLSKYAELLLSELGQGTADWVPNFDGTMQEPKMLPARLPNILLNGTTGIAVGMATDI |
| SRR3115978 | TESRLSKYAELLLSELGQGTADWVPNFDGTMQEPKMLPARLPNILLNGTTGIAVGMATDI |
| SRR2534093 | TESRLSKYAELLLSELGQGTADWVPNFDGTMQEPKMLPARLPNILLNGTTGIAVGMATDI |
| SRR2534094 | TESRLSKYAELLLSELGQGTADWVPNFDGTMQEPKMLPARLPNILLNGTTGIAVGMATDI |
| SRR2534095 | TESRLSKYAELLLSELGQGTADWVPNFDGTMQEPKMLPARLPNILLNGTTGIAVGMATDI |
| SRR2534108 | TESRLSKYAELLLSELGQGTADWVPNFDGTMQEPKMLPARLPNILLNGTTGIAVGMATDI |
| SRR1106464 | TESRLSKYAELLLSELGQGTADWVPNFDGTMQEPKMLPARLPNILLNGTTGIAVGMATDI |
| SRR1106463 | TESRLSKYAELLLSELGQGTADWVPNFDGTMQEPKMLPARLPNILLNGTTGIAVGMATDI |
| SRR6949610 | TESRLSKYAELLLSELGQGTADWVPNFDGTMQEPKMLPARLPNILLNGTTGIAVGMATDI |
| SRR6950452 | TESRLSKYAELLLSELGQGTADWVPNFDGTMQEPKMLPARLPNILLNGTTGIAVGMATDI |
| ERR2019831 | TESRLSKYAELLLSELGQGTADWVPNFDGTMQEPKMLPARLPNILLNGTTGIAVGMATDI |
| SRR2085693 | TESRLSKYAELLLSELGQGTADWVPNFDGTMQEPKMLPARLPNILLNGTTGIAVGMATDI |
| SRR2086898 | TESRLSKYAELLLSELGQGTADWVPNFDGTMQEPKMLPARLPNILLNGTTGIAVGMATDI |
| SRR2175312 | TESRLSKYAELLLSELGQGTADWVPNFDGTMQEPKMLPARLPNILLNGTTGIAVGMATDI |
| SRR2175360 | TESRLSKYAELLLSELGQGTADWVPNFDGTMQEPKMLPARLPNILLNGTTGIAVGMATDI |
| SRR5231997 | TESRLSKYAELLLSELGQGTADWVPNFDGTMQEPKMLPARLPNILLNGTTGIAVGMATDI |
| SRR5232003 | TESRLSKYAELLLSELGQGTADWVPNFDGTMQEPKMLPARLPNILLNGTTGIAVGMATDI |
| SRR5232015 | TESRLSKYAELLLSELGQGTADWVPNFDGTMQEPKMLPARLPNILLNGTTGIAVGMATDI |
| SRR949434 | TESRLSKYAELLLSELGQGTADWVPNFDGTMQEPKMLPARLPNILLNGTTGIAVGMATDI |
| SRR3216575 | TESRLSKYAELLLSELGQGTADWVPNFDGTMQEPKMLPARLPNILLNGTTGIAVGMATDI |
| SRR5205342 | TESRLSKYAELLLSELGQGTADWVPNFDGTMQEPKMLPARLPNILLNGTTGIAVGMATDI |
| SRR1501669 | TESRLSKYAELLLSELGQGTADWVPNFDGTMQEPKMLPARLPNILLNGTTGIAVGMATDI |
| SRR5209740 | TESRLSKYAELLLSELGQGTADWVPNFDGTMQEPKMLPARLPNILLNGTTGIAVGMATDI |
| SRR3240355 | TESRLSKYAELLLSELGQGTADWVPNFDGTMQEPKMLPARLPNILLNGTTGIAVGMATDI |
| SRR3392777 | TESRLSKYAELLLSELGQGTADWVPNFDGTMQEPKMLPARLPNILLNGTTGIAVGMATDI |
| SRR3593671 | TESRLSKYAELLLSELGQGTADWVPNFDGTMQEPKMLPARLPNILLNGTTGIAVGMATDI |
| SRR5413290 | TESRLSKYAELLLSELGQGTADWVPNFDGTMQEPKMLPARLPNILLNGTTGIAVGMATDI |
| SRR5590269 | TESRLSKYAELLLSELGQGTADWVPNFDGTMQEPKMLPARLPNILLNGTTGIAVGMATDI |
| SRR5812103 | TESRLSKYAELLLSELGQGTADWVPNFDGTMQEPKMLPARLPNILLNGTTGIAVGMATDI |
| SRR2830941 | TESRLSKYAELLLSELGQGTADWVPNFDGTMQEPKMLPARLPNILLNGTTGIAVGMATDI |
| SRR2830966 | TESRLSKYAELLLSELGQGTADWVPNFDGTMQEPKMLPARLPNILLNGTTGIAVGMATDI |
| SRR3137270 | TESRLSKYAELLLSELGQGTADWVPNFDGTMQEPKMLPARLPNILLNGTTGIAVGMATDI |
| SRR3137271 | TESRLSKYAELLLSELGQGTADWVPNFDGTMQEPKMLPARLPNILLNGTTGIAVGMATDI |
| ERR526807 | TESRLSKYAELLLSELGQGTADWVPNFDGTMQEPKMLPARLPNILLNGTTGIAVGMATDI |
| ERR2197922 | TESRLSKYAELLLSELGQGTADWVPNFDGTMQEPKMLPARLPNILLNGTTGIAVGMATDI |
| ERR2197923 | TESRLSKYAELLLSELGQGTADWVPNFDGTMQEPKMLPARLPNILLNGTTGIAVGMATDI |
| ERR2197924 | TESRLSKYAELLLSELGQGTADWVPNFDGTMQEPKMLPARLPNILLNGTTGIAVGMATDI |
| ERR2197925 | TESRLSKYAELLLSELGQGTADWVPNFDGTMQEPKMLPARLPNILLNGTTGIAVGMATDI |
| ERR2197927 | TESRLSKYAELLLSELGQGTADWVPNFDGTMQEPKMLPARLPNILLNGTTGIAVGMATDI |
| ERR2197929 | TESRLSKYAELLLSELGQGTADWVPNFDGTMQEPKMLPARLPNILLNGTTGIAVGMATDI |
| SRR1648149 | TESRLSKYAELLLSELGQGTADWVPNFDGTMQEPKMLPARLPNILLNGTTGIAVGMATDI |
| SRR1048299 | TESRLSKYAELLLSELGQGTADWVPNFDGTMQEPKMLPARLPNILLNGTTGIAVGMATDI |
| SRR1300677 | TESRLSKYAELLLSELGQGTADWVPNFDGTMQEPKMLPARLPNILLNGTTGIAVGMATDI |
| SRR1288356 | TESRLSKYAELLLSELGQGTADWVPNFDGTMQEPKMLPARLPNILLNGTTGIAVGMATDI |
| SRR7426190 | TESRLSKYAELLLSELGQGTADWVPNFDGTMQEPKMLPARLPNILLNGTTGIAVGMATDI |
| SRR7426192 | TESRLSKYAELLLSELGQGTADWVPNFDGTMQEPKMLPARLPNILLNGTTGIAVGMATDI |
| SRR7426193 | TESRLSKYAELLLSELGQGTADWVPNFDGTMQEPKMLPARLPNILLNGTTGIAVGMATDI |
| SRR7441832 | TESRLSKYAELLLSELGQGTADWVPNFDGTMQEPKMLPARLPNILLNGTTGIAVGMATDI |
| SRR7426179 | TESRLSKYAELLLSELGQGTADWVPNFDGTMQEPKMLPARLPNILLNGTTGIAVGMATDI |
| SRR7439238 | TESRLSKYAELLLSELGQGTADWVPNFDGTMQEPKMLPARLPNILLNGTTGIAVGMATDI |
| SRR7439244 | TESRLSKYAELLLSELGQGTADWVPNFDGTMQEPKMLPARLPNILLNGTTGIAVGMATDI |
| SRR7439259 | TESRLSKYAELLLSELGQGTADWVPNFDGTMQEPKMLPARLPNILLNGTTGIAVGMATDI |
| SRR7439260 | TESRLSKYAELLLSELGQGTADWVPNFDGTMQEPKMLPARLPNILLNGTTGIAVGMATDI |
| SRR7441786 | TESRLSKYAELLLSELGQGTADWVPNFDGTMQEPKMLPARLPNILLNGTTGIAVGMATDI |
| SRR7441797 | TESRLSKYAELLLSELGQGTADWVPNFDGTMQEPKMLPARLPNILLNGTTGIAVGMATDI |
| ERR1759093 | TESRLSKYAELLLSELGQGTADWVPNFDGTMQEPKMLPARLPNILLNGTTGIAVGMATDI |
| ERR2580275 | TESRLSKYAELLLSELGQGTADWVPNFDGTMQEPKMLPARLPNILLNGTTGIAVGMATDI |
| ERR1759204 | TESRLSKYAELLLSELGQGTADWVPNFDGTMQEPKMLPARLPNILLNGTTGIAVGMATDI |
| SRR1300699 | TESRLSKYAELLLSELGQGTADWVPNFDGTMQEPKMLPARLPNILLNGTTGIAVGMATDI |
| S_0825_17 | TESRLSKYAELLLSELGQGTADWVPNFDGTMQEPKMLPARLPNILLNGTTGIAVGMATDI |
| SRR1958215 | TESRLSKYAELLLSELGQGTADWVPNFDGTMQEPKMLPARLPNILLNGTTGIAVGMATDI |
| SRR1958540 | TESRLSKYAELLLSELGQGTADWVPNFDGTMQEPKMLPARLPNILLNGTTGIAVGMATDI |
| SRR1958636 | TESRLSKYAELLLSELGQGTADWVPNFDGTMQEPKMLPARLPNILLNGTTGIAVGMATDI |
| SRR1959422 | TESRLSKYAELLLSELGQGTADWVPNFDGTMQEPKMLPARLPNILLNGTTGIAVGMATDI |
| SRR1959427 | TESRLSKYAELLLSELGQGTADWVPNFDGTMQEPKMLPARLPNILLNGTTGIAVGMATDI |
| SRR1960226 | TESRLSKYAELLLSELGQGTADWVPNFDGTMQEPKMLPARLPNILLNGTTGIAVGMATDI |
| SRR1963498 | TESRLSKYAELLLSELGQGTADWVPNFDGTMQEPKMLPARLPNILLNGTTGIAVGMATDI |
| SRR1965947 | TESRLSKYAELLLSELGQGTADWVPNFDGTMQEPKMLPARLPNILLNGTTGIAVGMATDI |
| SRR1966125 | TESRLSKYAELLLSELGQGTADWVPNFDGTMQEPKMLPARLPNILLNGTTGIAVGMATDI |
| SRR1966330 | TESRLSKYAELLLSELGQGTADWVPNFDGTMQEPKMLPARLPNILLNGTTGIAVGMATDI |
| SRR1966565 | TESRLSKYAELLLSELGQGTADWVPNFDGTMQEPKMLPARLPNILLNGTTGIAVGMATDI |
| SRR1966864 | TESRLSKYAELLLSELGQGTADWVPNFDGTMQEPKMLPARLPNILLNGTTGIAVGMATDI |
| SRR1966989 | TESRLSKYAELLLSELGQGTADWVPNFDGTMQEPKMLPARLPNILLNGTTGIAVGMATDI |
| SRR1967688 | TESRLSKYAELLLSELGQGTADWVPNFDGTMQEPKMLPARLPNILLNGTTGIAVGMATDI |
| SRR1967733 | TESRLSKYAELLLSELGQGTADWVPNFDGTMQEPKMLPARLPNILLNGTTGIAVGMATDI |
| SRR1967746 | TESRLSKYAELLLSELGQGTADWVPNFDGTMQEPKMLPARLPNILLNGTTGIAVGMATDI |
| SRR1968341 | TESRLSKYAELLLSELGQGTADWVPNFDGTMQEPKMLPARLPNILLNGTTGIAVGMATDI |
| SRR1968456 | TESRLSKYAELLLSELGQGTADWVPNFDGTMQEPKMLPARLPNILLNGTTGIAVGMATDI |
| SRR1968465 | TESRLSKYAELLLSELGQGTADWVPNFDGTMQEPKMLPARLPNILLNGTTGIAVGMATDI |
| SRR1968761 | TESRLSKYAELLLSELGQGTADWVPNFDGTMQEPKMLPARLPNILLNGTTGIAVGMATDI |
| SRR1969047 | TESRLSKYAELLLSELGQGTADWVPNFDGTMQEPKMLPARLPNILLNGTTGIAVGMATDI |
| SRR1969255 | TESRLSKYAELLLSELGQGTADWVPNFDGTMQEPKMLPARLPNILLNGTTGIAVGMATDI |
| SRR1969412 | TESRLSKYAELLLSELGQGTADWVPNFDGTMQEPKMLPARLPNILLNGTTGIAVGMATDI |
| SRR1969524 | TESRLSKYAELLLSELGQGTADWVPNFDGTMQEPKMLPARLPNILLNGTTGIAVGMATDI |
| SRR1969584 | TESRLSKYAELLLSELGQGTADWVPNFDGTMQEPKMLPARLPNILLNGTTGIAVGMATDI |
| SRR1969648 | TESRLSKYAELLLSELGQGTADWVPNFDGTMQEPKMLPARLPNILLNGTTGIAVGMATDI |
| SRR1969804 | TESRLSKYAELLLSELGQGTADWVPNFDGTMQEPKMLPARLPNILLNGTTGIAVGMATDI |
| SRR1970221 | TESRLSKYAELLLSELGQGTADWVPNFDGTMQEPKMLPARLPNILLNGTTGIAVGMATDI |
| SRR1970268 | TESRLSKYAELLLSELGQGTADWVPNFDGTMQEPKMLPARLPNILLNGTTGIAVGMATDI |
| SRR1965862 | TESRLSKYAELLLSELGQGTADWVPNFDGTMQEPKMLPARLPNILLNGTTGIAVGMATDI |
| SRR1967363 | TESRLSKYAELLLSELGQGTADWVPNFDGTMQEPKMLPARLPNILLNGTTGIAVGMATDI |
| SRR1968276 | TESRLSKYAELLLSELGQGTADWVPNFDGTMQEPKMLPARLPNILLNGTTGIAVGMATDI |
| SRR1968967 | TESRLSKYAELLLSELGQGTADWVPNFDGTMQEPKMLPARLPNILLNGTTGIAVGMATDI |
| SRR3321531 | TESRLSKYAELLLSELGQGTADWVPNFDGTMQEPKMLPARLPNILLNGTTGIAVGMATDI |
| SRR3321883 | TESRLSKYAELLLSELGQGTADWVPNFDGTMQEPKMLPARLPNILLNGTTGIAVGMATDI |
| SRR3322413 | TESRLSKYAELLLSELGQGTADWVPNFDGTMQEPKMLPARLPNILLNGTTGIAVGMATDI |
| SRR3323012 | TESRLSKYAELLLSELGQGTADWVPNFDGTMQEPKMLPARLPNILLNGTTGIAVGMATDI |
| SRR5194289 | TESRLSKYAELLLSELGQGTADWVPNFDGTMQEPKMLPARLPNILLNGTTGIAVGMATDI |
| SRR7163798 | TESRLSKYAELLLSELGQGTADWVPNFDGTMQEPKMLPARLPNILLNGTTGIAVGMATDI |
| SRR7172610 | TESRLSKYAELLLSELGQGTADWVPNFDGTMQEPKMLPARLPNILLNGTTGIAVGMATDI |
| SRR7204568 | TESRLSKYAELLLSELGQGTADWVPNFDGTMQEPKMLPARLPNILLNGTTGIAVGMATDI |
| SRR7223230 | TESRLSKYAELLLSELGQGTADWVPNFDGTMQEPKMLPARLPNILLNGTTGIAVGMATDI |
| SRR7230675 | TESRLSKYAELLLSELGQGTADWVPNFDGTMQEPKMLPARLPNILLNGTTGIAVGMATDI |
| SRR7278056 | TESRLSKYAELLLSELGQGTADWVPNFDGTMQEPKMLPARLPNILLNGTTGIAVGMATDI |
| SRR7278086 | TESRLSKYAELLLSELGQGTADWVPNFDGTMQEPKMLPARLPNILLNGTTGIAVGMATDI |
| SRR7285841 | TESRLSKYAELLLSELGQGTADWVPNFDGTMQEPKMLPARLPNILLNGTTGIAVGMATDI |
| SRR7292625 | TESRLSKYAELLLSELGQGTADWVPNFDGTMQEPKMLPARLPNILLNGTTGIAVGMATDI |
| SRR7292665 | TESRLSKYAELLLSELGQGTADWVPNFDGTMQEPKMLPARLPNILLNGTTGIAVGMATDI |
| SRR7297965 | TESRLSKYAELLLSELGQGTADWVPNFDGTMQEPKMLPARLPNILLNGTTGIAVGMATDI |
| SRR7350726 | TESRLSKYAELLLSELGQGTADWVPNFDGTMQEPKMLPARLPNILLNGTTGIAVGMATDI |
| SRR7410328 | TESRLSKYAELLLSELGQGTADWVPNFDGTMQEPKMLPARLPNILLNGTTGIAVGMATDI |
| SRR7474665 | TESRLSKYAELLLSELGQGTADWVPNFDGTMQEPKMLPARLPNILLNGTTGIAVGMATDI |
| SRR7523184 | TESRLSKYAELLLSELGQGTADWVPNFDGTMQEPKMLPARLPNILLNGTTGIAVGMATDI |
| SRR7187264 | TESRLSKYAELLLSELGQGTADWVPNFDGTMQEPKMLPARLPNILLNGTTGIAVGMATDI |
| SRR7204445 | TESRLSKYAELLLSELGQGTADWVPNFDGTMQEPKMLPARLPNILLNGTTGIAVGMATDI |
| SRR7285641 | TESRLSKYAELLLSELGQGTADWVPNFDGTMQEPKMLPARLPNILLNGTTGIAVGMATDI |
| SRR7286695 | TESRLSKYAELLLSELGQGTADWVPNFDGTMQEPKMLPARLPNILLNGTTGIAVGMATDI |
| SRR7286705 | TESRLSKYAELLLSELGQGTADWVPNFDGTMQEPKMLPARLPNILLNGTTGIAVGMATDI |
| SRR7292931 | TESRLSKYAELLLSELGQGTADWVPNFDGTMQEPKMLPARLPNILLNGTTGIAVGMATDI |
| SRR7310349 | TESRLSKYAELLLSELGQGTADWVPNFDGTMQEPKMLPARLPNILLNGTTGIAVGMATDI |
| SRR7351616 | TESRLSKYAELLLSELGQGTADWVPNFDGTMQEPKMLPARLPNILLNGTTGIAVGMATDI |
| SRR7414818 | TESRLSKYAELLLSELGQGTADWVPNFDGTMQEPKMLPARLPNILLNGTTGIAVGMATDI |
| SRR7426480 | TESRLSKYAELLLSELGQGTADWVPNFDGTMQEPKMLPARLPNILLNGTTGIAVGMATDI |
| SRR5584105 | TESRLSKYAELLLSELGQGTADWVPNFDGTMQEPKMLPARLPNILLNGTTGIAVGMATDI |
| SRR5584565 | TESRLSKYAELLLSELGQGTADWVPNFDGTMQEPKMLPARLPNILLNGTTGIAVGMATDI |
| SRR5584614 | TESRLSKYAELLLSELGQGTADWVPNFDGTMQEPKMLPARLPNILLNGTTGIAVGMATDI |
| SRR5631543 | TESRLSKYAELLLSELGQGTADWVPNFDGTMQEPKMLPARLPNILLNGTTGIAVGMATDI |
| SRR5631553 | TESRLSKYAELLLSELGQGTADWVPNFDGTMQEPKMLPARLPNILLNGTTGIAVGMATDI |
| SRR7123196 | TESRLSKYAELLLSELGQGTADWVPNFDGTMQEPKMLPARLPNILLNGTTGIAVGMATDI |
| SRR7163819 | TESRLSKYAELLLSELGQGTADWVPNFDGTMQEPKMLPARLPNILLNGTTGIAVGMATDI |
| SRR7163920 | TESRLSKYAELLLSELGQGTADWVPNFDGTMQEPKMLPARLPNILLNGTTGIAVGMATDI |
| SRR7209528 | TESRLSKYAELLLSELGQGTADWVPNFDGTMQEPKMLPARLPNILLNGTTGIAVGMATDI |
| SRR7249868 | TESRLSKYAELLLSELGQGTADWVPNFDGTMQEPKMLPARLPNILLNGTTGIAVGMATDI |
| SRR7278088 | TESRLSKYAELLLSELGQGTADWVPNFDGTMQEPKMLPARLPNILLNGTTGIAVGMATDI |
| SRR7285788 | TESRLSKYAELLLSELGQGTADWVPNFDGTMQEPKMLPARLPNILLNGTTGIAVGMATDI |
| SRR7286789 | TESRLSKYAELLLSELGQGTADWVPNFDGTMQEPKMLPARLPNILLNGTTGIAVGMATDI |
| SRR7286886 | TESRLSKYAELLLSELGQGTADWVPNFDGTMQEPKMLPARLPNILLNGTTGIAVGMATDI |
| SRR7310632 | TESRLSKYAELLLSELGQGTADWVPNFDGTMQEPKMLPARLPNILLNGTTGIAVGMATDI |
| SRR7350631 | TESRLSKYAELLLSELGQGTADWVPNFDGTMQEPKMLPARLPNILLNGTTGIAVGMATDI |
| SRR7458741 | TESRLSKYAELLLSELGQGTADWVPNFDGTMQEPKMLPARLPNILLNGTTGIAVGMATDI |
| SRR7480280 | TESRLSKYAELLLSELGQGTADWVPNFDGTMQEPKMLPARLPNILLNGTTGIAVGMATDI |
| SRR7523660 | TESRLSKYAELLLSELGQGTADWVPNFDGTMQEPKMLPARLPNILLNGTTGIAVGMATDI |
| SRR7523775 | TESRLSKYAELLLSELGQGTADWVPNFDGTMQEPKMLPARLPNILLNGTTGIAVGMATDI |
| SRR7251101 | TESRLSKYAELLLSELGQGTADWVPNFDGTMQEPKMLPARLPNILLNGTTGIAVGMATDI |
| SRR7284299 | TESRLSKYAELLLSELGQGTADWVPNFDGTMQEPKMLPARLPNILLNGTTGIAVGMATDI |
| SRR7285738 | TESRLSKYAELLLSELGQGTADWVPNFDGTMQEPKMLPARLPNILLNGTTGIAVGMATDI |
| SRR7310640 | TESRLSKYAELLLSELGQGTADWVPNFDGTMQEPKMLPARLPNILLNGTTGIAVGMATDI |
| SRR7349159 | TESRLSKYAELLLSELGQGTADWVPNFDGTMQEPKMLPARLPNILLNGTTGIAVGMATDI |
| SRR7474873 | TESRLSKYAELLLSELGQGTADWVPNFDGTMQEPKMLPARLPNILLNGTTGIAVGMATDI |
| SRR7495689 | TESRLSKYAELLLSELGQGTADWVPNFDGTMQEPKMLPARLPNILLNGTTGIAVGMATDI |
| SRR7495752 | TESRLSKYAELLLSELGQGTADWVPNFDGTMQEPKMLPARLPNILLNGTTGIAVGMATDI |
| ------------------------------------------------------------------------------ | |
| S16BD08730 | PPHNLREVAKAAITLIEQPKTTLDQLLDIVQGPDYPTEAEIITPRAEIRKIYENGRGSVR |
| S18BD00684 | PPHNLREVAKAAITLIEQPKTTLDQLLDIVQGPDYPTEAEIITPRAEIRKIYENGRGSVR |
| S18BD03994 | PPHNLREVAKAAITLIEQPKTTLDQLLDIVQGPDYPTEAEIITPRAEIRKIYENGRGSVR |
| S18BD05011 | PPHNLREVAKAAITLIEQPKTTLDQLLDIVQGPDYPTEAEIITPRAEIRKIYENGRGSVR |
| RKI_16-03723 | PPHNLREVAKAAITLIEQPKTTLDQLLDIVQGPDYPTEAEIITPRAEIRKIYENGRGSVR |
| RKI_16-04315 | PPHNLREVAKAAITLIEQPKTTLDQLLDIVQGPDYPTEAEIITPRAEIRKIYENGRGSVR |
| RKI_17-02304 | PPHNLREVAKAAITLIEQPKTTLDQLLDIVQGPDYPTEAEIITPRAEIRKIYENGRGSVR |
| RKI_17-02411 | PPHNLREVAKAAITLIEQPKTTLDQLLDIVQGPDYPTEAEIITPRAEIRKIYENGRGSVR |
| RKI_17-02757 | PPHNLREVAKAAITLIEQPKTTLDQLLDIVQGPDYPTEAEIITPRAEIRKIYENGRGSVR |
| RKI_17-04797 | PPHNLREVAKAAITLIEQPKTTLDQLLDIVQGPDYPTEAEIITPRAEIRKIYENGRGSVR |
| RKI_17-06869 | PPHNLREVAKAAITLIEQPKTTLDQLLDIVQGPDYPTEAEIITPRAEIRKIYENGRGSVR |
| ERR2580277 | PPHNLREVAKAAITLIEQPKTTLDQLLDIVQGPDYPTEAEIITPRAEIRKIYENGRGSVR |
| ERR2580276 | PPHNLREVAKAAITLIEQPKTTLDQLLDIVQGPDYPTEAEIITPRAEIRKIYENGRGSVR |
| ERR2580273 | PPHNLREVAKAAITLIEQPKTTLDQLLDIVQGPDYPTEAEIITPRAEIRKIYENGRGSVR |
| ERR2580274 | PPHNLREVAKAAITLIEQPKTTLDQLLDIVQGPDYPTEAEIITPRAEIRKIYENGRGSVR |
| ERR2173656 | PPHNLREVAKAAITLIEQPKTTLDQLLDIVQGPDYPTEAEIITPRAEIRKIYENGRGSVR |
| 17041676 | PPHNLREVAKAAITLIEQPKTTLDQLLDIVQGPDYPTEAEIITPRAEIRKIYENGRGSVR |
| MT16-000061 | PPHNLREVAKAAITLIEQPKTTLDQLLDIVQGPDYPTEAEIITPRAEIRKIYENGRGSVR |
| MT16-019416 | PPHNLREVAKAAITLIEQPKTTLDQLLDIVQGPDYPTEAEIITPRAEIRKIYENGRGSVR |
| MT16-027865 | PPHNLREVAKAAITLIEQPKTTLDQLLDIVQGPDYPTEAEIITPRAEIRKIYENGRGSVR |
| MT16-031693 | PPHNLREVAKAAITLIEQPKTTLDQLLDIVQGPDYPTEAEIITPRAEIRKIYENGRGSVR |
| MT16-040253 | PPHNLREVAKAAITLIEQPKTTLDQLLDIVQGPDYPTEAEIITPRAEIRKIYENGRGSVR |
| MT16-045379 | PPHNLREVAKAAITLIEQPKTTLDQLLDIVQGPDYPTEAEIITPRAEIRKIYENGRGSVR |
| MT16-442728 | PPHNLREVAKAAITLIEQPKTTLDQLLDIVQGPDYPTEAEIITPRAEIRKIYENGRGSVR |
| MT16-462857 | PPHNLREVAKAAITLIEQPKTTLDQLLDIVQGPDYPTEAEIITPRAEIRKIYENGRGSVR |
| MT16-480196 | PPHNLREVAKAAITLIEQPKTTLDQLLDIVQGPDYPTEAEIITPRAEIRKIYENGRGSVR |
| MT16-861555 | PPHNLREVAKAAITLIEQPKTTLDQLLDIVQGPDYPTEAEIITPRAEIRKIYENGRGSVR |
| MT17-076833 | PPHNLREVAKAAITLIEQPKTTLDQLLDIVQGPDYPTEAEIITPRAEIRKIYENGRGSVR |
| MT17-110677 | PPHNLREVAKAAITLIEQPKTTLDQLLDIVQGPDYPTEAEIITPRAEIRKIYENGRGSVR |
| MT17-131730 | PPHNLREVAKAAITLIEQPKTTLDQLLDIVQGPDYPTEAEIITPRAEIRKIYENGRGSVR |
| MT17-140890 | PPHNLREVAKAAITLIEQPKTTLDQLLDIVQGPDYPTEAEIITPRAEIRKIYENGRGSVR |
| MT17-141840 | PPHNLREVAKAAITLIEQPKTTLDQLLDIVQGPDYPTEAEIITPRAEIRKIYENGRGSVR |
| MT17-152488 | PPHNLREVAKAAITLIEQPKTTLDQLLDIVQGPDYPTEAEIITPRAEIRKIYENGRGSVR |
| MT17-157311 | PPHNLREVAKAAITLIEQPKTTLDQLLDIVQGPDYPTEAEIITPRAEIRKIYENGRGSVR |
| MT17-161645 | PPHNLREVAKAAITLIEQPKTTLDQLLDIVQGPDYPTEAEIITPRAEIRKIYENGRGSVR |
| MT17-167951 | PPHNLREVAKAAITLIEQPKTTLDQLLDIVQGPDYPTEAEIITPRAEIRKIYENGRGSVR |
| MT18-217732 | PPHNLREVAKAAITLIEQPKTTLDQLLDIVQGPDYPTEAEIITPRAEIRKIYENGRGSVR |
| MT18-252580 | PPHNLREVAKAAITLIEQPKTTLDQLLDIVQGPDYPTEAEIITPRAEIRKIYENGRGSVR |
| RIVM_H_2009-01 | PPHNLREVAKAAITLIEQPKTTLDQLLDIVQGPDYPTEAEIITPRAEIRKIYENGRGSVR |
| RIVM_H_2010-01 | PPHNLREVAKAAITLIEQPKTTLDQLLDIVQGPDYPTEAEIITPRAEIRKIYENGRGSVR |
| RIVM_H_2010-02 | PPHNLREVAKAAITLIEQPKTTLDQLLDIVQGPDYPTEAEIITPRAEIRKIYENGRGSVR |
| RIVM_H_2011-01 | PPHNLREVAKAAITLIEQPKTTLDQLLDIVQGPDYPTEAEIITPRAEIRKIYENGRGSVR |
| RIVM_H_2011-02 | PPHNLREVAKAAITLIEQPKTTLDQLLDIVQGPDYPTEAEIITPRAEIRKIYENGRGSVR |
| RIVM_H_2011-03 | PPHNLREVAKAAITLIEQPKTTLDQLLDIVQGPDYPTEAEIITPRAEIRKIYENGRGSVR |
| RIVM_H_2013-01 | PPHNLREVAKAAITLIEQPKTTLDQLLDIVQGPDYPTEAEIITPRAEIRKIYENGRGSVR |
| RIVM_H_2013-02 | PPHNLREVAKAAITLIEQPKTTLDQLLDIVQGPDYPTEAEIITPRAEIRKIYENGRGSVR |
| RIVM_H_2014-01 | PPHNLREVAKAAITLIEQPKTTLDQLLDIVQGPDYPTEAEIITPRAEIRKIYENGRGSVR |
| RIVM_H_2014-02 | PPHNLREVAKAAITLIEQPKTTLDQLLDIVQGPDYPTEAEIITPRAEIRKIYENGRGSVR |
| RIVM_H_2016-01 | PPHNLREVAKAAITLIEQPKTTLDQLLDIVQGPDYPTEAEIITPRAEIRKIYENGRGSVR |
| RIVM_H_2016-02 | PPHNLREVAKAAITLIEQPKTTLDQLLDIVQGPDYPTEAEIITPRAEIRKIYENGRGSVR |
| RIVM_H_2016-03 | PPHNLREVAKAAITLIEQPKTTLDQLLDIVQGPDYPTEAEIITPRAEIRKIYENGRGSVR |
| RIVM_H_2016-04 | PPHNLREVAKAAITLIEQPKTTLDQLLDIVQGPDYPTEAEIITPRAEIRKIYENGRGSVR |
| RIVM_H_2016-05 | PPHNLREVAKAAITLIEQPKTTLDQLLDIVQGPDYPTEAEIITPRAEIRKIYENGRGSVR |
| RIVM_H_2016-06 | PPHNLREVAKAAITLIEQPKTTLDQLLDIVQGPDYPTEAEIITPRAEIRKIYENGRGSVR |
| RIVM_H_2016-07 | PPHNLREVAKAAITLIEQPKTTLDQLLDIVQGPDYPTEAEIITPRAEIRKIYENGRGSVR |
| RIVM_H_2016-08 | PPHNLREVAKAAITLIEQPKTTLDQLLDIVQGPDYPTEAEIITPRAEIRKIYENGRGSVR |
| RIVM_H_2016-09 | PPHNLREVAKAAITLIEQPKTTLDQLLDIVQGPDYPTEAEIITPRAEIRKIYENGRGSVR |
| RIVM_H_2016-10 | PPHNLREVAKAAITLIEQPKTTLDQLLDIVQGPDYPTEAEIITPRAEIRKIYENGRGSVR |
| RIVM_H_2016-11 | PPHNLREVAKAAITLIEQPKTTLDQLLDIVQGPDYPTEAEIITPRAEIRKIYENGRGSVR |
| RIVM_H_2016-12 | PPHNLREVAKAAITLIEQPKTTLDQLLDIVQGPDYPTEAEIITPRAEIRKIYENGRGSVR |
| RIVM_H_2016-13 | PPHNLREVAKAAITLIEQPKTTLDQLLDIVQGPDYPTEAEIITPRAEIRKIYENGRGSVR |
| RIVM_H_2016-14 | PPHNLREVAKAAITLIEQPKTTLDQLLDIVQGPDYPTEAEIITPRAEIRKIYENGRGSVR |
| RIVM_H_2016-15 | PPHNLREVAKAAITLIEQPKTTLDQLLDIVQGPDYPTEAEIITPRAEIRKIYENGRGSVR |
| RIVM_H_2017-01 | PPHNLREVAKAAITLIEQPKTTLDQLLDIVQGPDYPTEAEIITPRAEIRKIYENGRGSVR |
| RIVM_H_2017-02 | PPHNLREVAKAAITLIEQPKTTLDQLLDIVQGPDYPTEAEIITPRAEIRKIYENGRGSVR |
| RIVM_H_2017-03 | PPHNLREVAKAAITLIEQPKTTLDQLLDIVQGPDYPTEAEIITPRAEIRKIYENGRGSVR |
| RIVM_H_2017-04 | PPHNLREVAKAAITLIEQPKTTLDQLLDIVQGPDYPTEAEIITPRAEIRKIYENGRGSVR |
| RIVM_H_2017-05 | PPHNLREVAKAAITLIEQPKTTLDQLLDIVQGPDYPTEAEIITPRAEIRKIYENGRGSVR |
| RIVM_H_2017-06 | PPHNLREVAKAAITLIEQPKTTLDQLLDIVQGPDYPTEAEIITPRAEIRKIYENGRGSVR |
| RIVM_H_2017-07 | PPHNLREVAKAAITLIEQPKTTLDQLLDIVQGPDYPTEAEIITPRAEIRKIYENGRGSVR |
| RIVM_H_2017-08 | PPHNLREVAKAAITLIEQPKTTLDQLLDIVQGPDYPTEAEIITPRAEIRKIYENGRGSVR |
| RIVM_H_2017-09 | PPHNLREVAKAAITLIEQPKTTLDQLLDIVQGPDYPTEAEIITPRAEIRKIYENGRGSVR |
| RIVM_H_2017-10 | PPHNLREVAKAAITLIEQPKTTLDQLLDIVQGPDYPTEAEIITPRAEIRKIYENGRGSVR |
| RIVM_H_2017-11 | PPHNLREVAKAAITLIEQPKTTLDQLLDIVQGPDYPTEAEIITPRAEIRKIYENGRGSVR |
| RIVM_H_2017-12 | PPHNLREVAKAAITLIEQPKTTLDQLLDIVQGPDYPTEAEIITPRAEIRKIYENGRGSVR |
| RIVM_H_2017-13 | PPHNLREVAKAAITLIEQPKTTLDQLLDIVQGPDYPTEAEIITPRAEIRKIYENGRGSVR |
| RIVM_H_2017-14 | PPHNLREVAKAAITLIEQPKTTLDQLLDIVQGPDYPTEAEIITPRAEIRKIYENGRGSVR |
| RIVM_H_2017-15 | PPHNLREVAKAAITLIEQPKTTLDQLLDIVQGPDYPTEAEIITPRAEIRKIYENGRGSVR |
| RIVM_H_2017-16 | PPHNLREVAKAAITLIEQPKTTLDQLLDIVQGPDYPTEAEIITPRAEIRKIYENGRGSVR |
| RIVM_H_2017-17 | PPHNLREVAKAAITLIEQPKTTLDQLLDIVQGPDYPTEAEIITPRAEIRKIYENGRGSVR |
| RIVM_H_2017-18 | PPHNLREVAKAAITLIEQPKTTLDQLLDIVQGPDYPTEAEIITPRAEIRKIYENGRGSVR |
| RIVM_H_2017-19 | PPHNLREVAKAAITLIEQPKTTLDQLLDIVQGPDYPTEAEIITPRAEIRKIYENGRGSVR |
| 15EP001483 | PPHNLREVAKAAITLIEQPKTTLDQLLDIVQGPDYPTEAEIITPRAEIRKIYENGRGSVR |
| 17EP002363 | PPHNLREVAKAAITLIEQPKTTLDQLLDIVQGPDYPTEAEIITPRAEIRKIYENGRGSVR |
| S_0812_17 | PPHNLREVAKAAITLIEQPKTTLDQLLDIVQGPDYPTEAEIITPRAEIRKIYENGRGSVR |
| SRR1957844 | PPHNLREVAKAAITLIEQPKTTLDQLLDIVQGPDYPTEAEIITPRAEIRKIYENGRGSVR |
| SRR1958654 | PPHNLREVAKAAITLIEQPKTTLDQLLDIVQGPDYPTEAEIITPRAEIRKIYENGRGSVR |
| SRR1965077 | PPHNLREVAKAAITLIEQPKTTLDQLLDIVQGPDYPTEAEIITPRAEIRKIYENGRGSVR |
| SRR1966369 | PPHNLREVAKAAITLIEQPKTTLDQLLDIVQGPDYPTEAEIITPRAEIRKIYENGRGSVR |
| SRR1967117 | PPHNLREVAKAAITLIEQPKTTLDQLLDIVQGPDYPTEAEIITPRAEIRKIYENGRGSVR |
| SRR1967922 | PPHNLREVAKAAITLIEQPKTTLDQLLDIVQGPDYPTEAEIITPRAEIRKIYENGRGSVR |
| SRR8704720 | PPHNLREVAKAAITLIEQPKTTLDQLLDIVQGPDYPTEAEIITPRAEIRKIYENGRGSVR |
| SRR7216071 | PPHNLREVAKAAITLIEQPKTTLDQLLDIVQGPDYPTEAEIITPRAEIRKIYENGRGSVR |
| SRR7349175 | PPHNLREVAKAAITLIEQPKTTLDQLLDIVQGPDYPTEAEIITPRAEIRKIYENGRGSVR |
| SRR7523148 | PPHNLREVAKAAITLIEQPKTTLDQLLDIVQGPDYPTEAEIITPRAEIRKIYENGRGSVR |
| SRR7523854 | PPHNLREVAKAAITLIEQPKTTLDQLLDIVQGPDYPTEAEIITPRAEIRKIYENGRGSVR |
| 313865 | PPHNLREVAKAAITLIEQPKTTLDQLLDIVQGPDYPTEAEIITPRAEIRKIYENGRGSVR |
| SRR7277793 | PPHNLREVAKAAITLIEQPKTTLDQLLDIVQGPDYPTEAEIITPRAEIRKIYENGRGSVR |
| SRR7343877 | PPHNLREVAKAAITLIEQPKTTLDQLLDIVQGPDYPTEAEIITPRAEIRKIYENGRGSVR |
| SRR7351477 | PPHNLREVAKAAITLIEQPKTTLDQLLDIVQGPDYPTEAEIITPRAEIRKIYENGRGSVR |
| SRR5583183 | PPHNLREVAKAAITLIEQPKTTLDQLLDIVQGPDYPTEAEIITPRAEIRKIYENGRGSVR |
| SRR5585240 | PPHNLREVAKAAITLIEQPKTTLDQLLDIVQGPDYPTEAEIITPRAEIRKIYENGRGSVR |
| SRR7284317 | PPHNLREVAKAAITLIEQPKTTLDQLLDIVQGPDYPTEAEIITPRAEIRKIYENGRGSVR |
| SRR7299161 | PPHNLREVAKAAITLIEQPKTTLDQLLDIVQGPDYPTEAEIITPRAEIRKIYENGRGSVR |
| SRR7401730 | PPHNLREVAKAAITLIEQPKTTLDQLLDIVQGPDYPTEAEIITPRAEIRKIYENGRGSVR |
| SRR7469092 | PPHNLREVAKAAITLIEQPKTTLDQLLDIVQGPDYPTEAEIITPRAEIRKIYENGRGSVR |
| SRR7879556 | PPHNLREVAKAAITLIEQPKTTLDQLLDIVQGPDYPTEAEIITPRAEIRKIYENGRGSVR |
| SRR8526100 | PPHNLREVAKAAITLIEQPKTTLDQLLDIVQGPDYPTEAEIITPRAEIRKIYENGRGSVR |
| SRR8553991 | PPHNLREVAKAAITLIEQPKTTLDQLLDIVQGPDYPTEAEIITPRAEIRKIYENGRGSVR |
| SRR7842487 | PPHNLREVAKAAITLIEQPKTTLDQLLDIVQGPDYPTEAEIITPRAEIRKIYENGRGSVR |
| SRR8054524 | PPHNLREVAKAAITLIEQPKTTLDQLLDIVQGPDYPTEAEIITPRAEIRKIYENGRGSVR |
| SRR8054525 | PPHNLREVAKAAITLIEQPKTTLDQLLDIVQGPDYPTEAEIITPRAEIRKIYENGRGSVR |
| SRR8524733 | PPHNLREVAKAAITLIEQPKTTLDQLLDIVQGPDYPTEAEIITPRAEIRKIYENGRGSVR |
| SRR4093291 | PPHNLREVAKAAITLIEQPKTTLDQLLDIVQGPDYPTEAEIITPRAEIRKIYENGRGSVR |
| SRR4245549 | PPHNLREVAKAAITLIEQPKTTLDQLLDIVQGPDYPTEAEIITPRAEIRKIYENGRGSVR |
| SRR3057154 | PPHNLREVAKAAITLIEQPKTTLDQLLDIVQGPDYPTEAEIITPRAEIRKIYENGRGSVR |
| SRR1726150 | PPHNLREVAKAAITLIEQPKTTLDQLLDIVQGPDYPTEAEIITPRAEIRKIYENGRGSVR |
| SRR1996141 | PPHNLREVAKAAITLIEQPKTTLDQLLDIVQGPDYPTEAEIITPRAEIRKIYENGRGSVR |
| SRR1107842 | PPHNLREVAKAAITLIEQPKTTLDQLLDIVQGPDYPTEAEIITPRAEIRKIYENGRGSVR |
| SRR1157587 | PPHNLREVAKAAITLIEQPKTTLDQLLDIVQGPDYPTEAEIITPRAEIRKIYENGRGSVR |
| SRR3027706 | PPHNLREVAKAAITLIEQPKTTLDQLLDIVQGPDYPTEAEIITPRAEIRKIYENGRGSVR |
| SRR3027707 | PPHNLREVAKAAITLIEQPKTTLDQLLDIVQGPDYPTEAEIITPRAEIRKIYENGRGSVR |
| SRR3027708 | PPHNLREVAKAAITLIEQPKTTLDQLLDIVQGPDYPTEAEIITPRAEIRKIYENGRGSVR |
| SRR3027710 | PPHNLREVAKAAITLIEQPKTTLDQLLDIVQGPDYPTEAEIITPRAEIRKIYENGRGSVR |
| SRR3027711 | PPHNLREVAKAAITLIEQPKTTLDQLLDIVQGPDYPTEAEIITPRAEIRKIYENGRGSVR |
| SRR3027716 | PPHNLREVAKAAITLIEQPKTTLDQLLDIVQGPDYPTEAEIITPRAEIRKIYENGRGSVR |
| SRR3027717 | PPHNLREVAKAAITLIEQPKTTLDQLLDIVQGPDYPTEAEIITPRAEIRKIYENGRGSVR |
| SRR3027719 | PPHNLREVAKAAITLIEQPKTTLDQLLDIVQGPDYPTEAEIITPRAEIRKIYENGRGSVR |
| SRR3027721 | PPHNLREVAKAAITLIEQPKTTLDQLLDIVQGPDYPTEAEIITPRAEIRKIYENGRGSVR |
| SRR3027723 | PPHNLREVAKAAITLIEQPKTTLDQLLDIVQGPDYPTEAEIITPRAEIRKIYENGRGSVR |
| SRR3115978 | PPHNLREVAKAAITLIEQPKTTLDQLLDIVQGPDYPTEAEIITPRAEIRKIYENGRGSVR |
| SRR2534093 | PPHNLREVAKAAITLIEQPKTTLDQLLDIVQGPDYPTEAEIITPRAEIRKIYENGRGSVR |
| SRR2534094 | PPHNLREVAKAAITLIEQPKTTLDQLLDIVQGPDYPTEAEIITPRAEIRKIYENGRGSVR |
| SRR2534095 | PPHNLREVAKAAITLIEQPKTTLDQLLDIVQGPDYPTEAEIITPRAEIRKIYENGRGSVR |
| SRR2534108 | PPHNLREVAKAAITLIEQPKTTLDQLLDIVQGPDYPTEAEIITPRAEIRKIYENGRGSVR |
| SRR1106464 | PPHNLREVAKAAITLIEQPKTTLDQLLDIVQGPDYPTEAEIITPRAEIRKIYENGRGSVR |
| SRR1106463 | PPHNLREVAKAAITLIEQPKTTLDQLLDIVQGPDYPTEAEIITPRAEIRKIYENGRGSVR |
| SRR6949610 | PPHNLREVAKAAITLIEQPKTTLDQLLDIVQGPDYPTEAEIITPRAEIRKIYENGRGSVR |
| SRR6950452 | PPHNLREVAKAAITLIEQPKTTLDQLLDIVQGPDYPTEAEIITPRAEIRKIYENGRGSVR |
| ERR2019831 | PPHNLREVAKAAITLIEQPKTTLDQLLDIVQGPDYPTEAEIITPRAEIRKIYENGRGSVR |
| SRR2085693 | PPHNLREVAKAAITLIEQPKTTLDQLLDIVQGPDYPTEAEIITPRAEIRKIYENGRGSVR |
| SRR2086898 | PPHNLREVAKAAITLIEQPKTTLDQLLDIVQGPDYPTEAEIITPRAEIRKIYENGRGSVR |
| SRR2175312 | PPHNLREVAKAAITLIEQPKTTLDQLLDIVQGPDYPTEAEIITPRAEIRKIYENGRGSVR |
| SRR2175360 | PPHNLREVAKAAITLIEQPKTTLDQLLDIVQGPDYPTEAEIITPRAEIRKIYENGRGSVR |
| SRR5231997 | PPHNLREVAKAAITLIEQPKTTLDQLLDIVQGPDYPTEAEIITPRAEIRKIYENGRGSVR |
| SRR5232003 | PPHNLREVAKAAITLIEQPKTTLDQLLDIVQGPDYPTEAEIITPRAEIRKIYENGRGSVR |
| SRR5232015 | PPHNLREVAKAAITLIEQPKTTLDQLLDIVQGPDYPTEAEIITPRAEIRKIYENGRGSVR |
| SRR949434 | PPHNLREVAKAAITLIEQPKTTLDQLLDIVQGPDYPTEAEIITPRAEIRKIYENGRGSVR |
| SRR3216575 | PPHNLREVAKAAITLIEQPKTTLDQLLDIVQGPDYPTEAEIITPRAEIRKIYENGRGSVR |
| SRR5205342 | PPHNLREVAKAAITLIEQPKTTLDQLLDIVQGPDYPTEAEIITPRAEIRKIYENGRGSVR |
| SRR1501669 | PPHNLREVAKAAITLIEQPKTTLDQLLDIVQGPDYPTEAEIITPRAEIRKIYENGRGSVR |
| SRR5209740 | PPHNLREVAKAAITLIEQPKTTLDQLLDIVQGPDYPTEAEIITPRAEIRKIYENGRGSVR |
| SRR3240355 | PPHNLREVAKAAITLIEQPKTTLDQLLDIVQGPDYPTEAEIITPRAEIRKIYENGRGSVR |
| SRR3392777 | PPHNLREVAKAAITLIEQPKTTLDQLLDIVQGPDYPTEAEIITPRAEIRKIYENGRGSVR |
| SRR3593671 | PPHNLREVAKAAITLIEQPKTTLDQLLDIVQGPDYPTEAEIITPRAEIRKIYENGRGSVR |
| SRR5413290 | PPHNLREVAKAAITLIEQPKTTLDQLLDIVQGPDYPTEAEIITPRAEIRKIYENGRGSVR |
| SRR5590269 | PPHNLREVAKAAITLIEQPKTTLDQLLDIVQGPDYPTEAEIITPRAEIRKIYENGRGSVR |
| SRR5812103 | PPHNLREVAKAAITLIEQPKTTLDQLLDIVQGPDYPTEAEIITPRAEIRKIYENGRGSVR |
| SRR2830941 | PPHNLREVAKAAITLIEQPKTTLDQLLDIVQGPDYPTEAEIITPRAEIRKIYENGRGSVR |
| SRR2830966 | PPHNLREVAKAAITLIEQPKTTLDQLLDIVQGPDYPTEAEIITPRAEIRKIYENGRGSVR |
| SRR3137270 | PPHNLREVAKAAITLIEQPKTTLDQLLDIVQGPDYPTEAEIITPRAEIRKIYENGRGSVR |
| SRR3137271 | PPHNLREVAKAAITLIEQPKTTLDQLLDIVQGPDYPTEAEIITPRAEIRKIYENGRGSVR |
| ERR526807 | PPHNLREVAKAAITLIEQPKTTLDQLLDIVQGPDYPTEAEIITPRAEIRKIYENGRGSVR |
| ERR2197922 | PPHNLREVAKAAITLIEQPKTTLDQLLDIVQGPDYPTEAEIITPRAEIRKIYENGRGSVR |
| ERR2197923 | PPHNLREVAKAAITLIEQPKTTLDQLLDIVQGPDYPTEAEIITPRAEIRKIYENGRGSVR |
| ERR2197924 | PPHNLREVAKAAITLIEQPKTTLDQLLDIVQGPDYPTEAEIITPRAEIRKIYENGRGSVR |
| ERR2197925 | PPHNLREVAKAAITLIEQPKTTLDQLLDIVQGPDYPTEAEIITPRAEIRKIYENGRGSVR |
| ERR2197927 | PPHNLREVAKAAITLIEQPKTTLDQLLDIVQGPDYPTEAEIITPRAEIRKIYENGRGSVR |
| ERR2197929 | PPHNLREVAKAAITLIEQPKTTLDQLLDIVQGPDYPTEAEIITPRAEIRKIYENGRGSVR |
| SRR1648149 | PPHNLREVAKAAITLIEQPKTTLDQLLDIVQGPDYPTEAEIITPRAEIRKIYENGRGSVR |
| SRR1048299 | PPHNLREVAKAAITLIEQPKTTLDQLLDIVQGPDYPTEAEIITPRAEIRKIYENGRGSVR |
| SRR1300677 | PPHNLREVAKAAITLIEQPKTTLDQLLDIVQGPDYPTEAEIITPRAEIRKIYENGRGSVR |
| SRR1288356 | PPHNLREVAKAAITLIEQPKTTLDQLLDIVQGPDYPTEAEIITPRAEIRKIYENGRGSVR |
| SRR7426190 | PPHNLREVAKAAITLIEQPKTTLDQLLDIVQGPDYPTEAEIITPRAEIRKIYENGRGSVR |
| SRR7426192 | PPHNLREVAKAAITLIEQPKTTLDQLLDIVQGPDYPTEAEIITPRAEIRKIYENGRGSVR |
| SRR7426193 | PPHNLREVAKAAITLIEQPKTTLDQLLDIVQGPDYPTEAEIITPRAEIRKIYENGRGSVR |
| SRR7441832 | PPHNLREVAKAAITLIEQPKTTLDQLLDIVQGPDYPTEAEIITPRAEIRKIYENGRGSVR |
| SRR7426179 | PPHNLREVAKAAITLIEQPKTTLDQLLDIVQGPDYPTEAEIITPRAEIRKIYENGRGSVR |
| SRR7439238 | PPHNLREVAKAAITLIEQPKTTLDQLLDIVQGPDYPTEAEIITPRAEIRKIYENGRGSVR |
| SRR7439244 | PPHNLREVAKAAITLIEQPKTTLDQLLDIVQGPDYPTEAEIITPRAEIRKIYENGRGSVR |
| SRR7439259 | PPHNLREVAKAAITLIEQPKTTLDQLLDIVQGPDYPTEAEIITPRAEIRKIYENGRGSVR |
| SRR7439260 | PPHNLREVAKAAITLIEQPKTTLDQLLDIVQGPDYPTEAEIITPRAEIRKIYENGRGSVR |
| SRR7441786 | PPHNLREVAKAAITLIEQPKTTLDQLLDIVQGPDYPTEAEIITPRAEIRKIYENGRGSVR |
| SRR7441797 | PPHNLREVAKAAITLIEQPKTTLDQLLDIVQGPDYPTEAEIITPRAEIRKIYENGRGSVR |
| ERR1759093 | PPHNLREVAKAAITLIEQPKTTLDQLLDIVQGPDYPTEAEIITPRAEIRKIYENGRGSVR |
| ERR2580275 | PPHNLREVAKAAITLIEQPKTTLDQLLDIVQGPDYPTEAEIITPRAEIRKIYENGRGSVR |
| ERR1759204 | PPHNLREVAKAAITLIEQPKTTLDQLLDIVQGPDYPTEAEIITPRAEIRKIYENGRGSVR |
| SRR1300699 | PPHNLREVAKAAITLIEQPKTTLDQLLDIVQGPDYPTEAEIITPRAEIRKIYENGRGSVR |
| S_0825_17 | PPHNLREVAKAAITLIEQPKTTLDQLLDIVQGPDYPTEAEIITPRAEIRKIYENGRGSVR |
| SRR1958215 | PPHNLREVAKAAITLIEQPKTTLDQLLDIVQGPDYPTEAEIITPRAEIRKIYENGRGSVR |
| SRR1958540 | PPHNLREVAKAAITLIEQPKTTLDQLLDIVQGPDYPTEAEIITPRAEIRKIYENGRGSVR |
| SRR1958636 | PPHNLREVAKAAITLIEQPKTTLDQLLDIVQGPDYPTEAEIITPRAEIRKIYENGRGSVR |
| SRR1959422 | PPHNLREVAKAAITLIEQPKTTLDQLLDIVQGPDYPTEAEIITPRAEIRKIYENGRGSVR |
| SRR1959427 | PPHNLREVAKAAITLIEQPKTTLDQLLDIVQGPDYPTEAEIITPRAEIRKIYENGRGSVR |
| SRR1960226 | PPHNLREVAKAAITLIEQPKTTLDQLLDIVQGPDYPTEAEIITPRAEIRKIYENGRGSVR |
| SRR1963498 | PPHNLREVAKAAITLIEQPKTTLDQLLDIVQGPDYPTEAEIITPRAEIRKIYENGRGSVR |
| SRR1965947 | PPHNLREVAKAAITLIEQPKTTLDQLLDIVQGPDYPTEAEIITPRAEIRKIYENGRGSVR |
| SRR1966125 | PPHNLREVAKAAITLIEQPKTTLDQLLDIVQGPDYPTEAEIITPRAEIRKIYENGRGSVR |
| SRR1966330 | PPHNLREVAKAAITLIEQPKTTLDQLLDIVQGPDYPTEAEIITPRAEIRKIYENGRGSVR |
| SRR1966565 | PPHNLREVAKAAITLIEQPKTTLDQLLDIVQGPDYPTEAEIITPRAEIRKIYENGRGSVR |
| SRR1966864 | PPHNLREVAKAAITLIEQPKTTLDQLLDIVQGPDYPTEAEIITPRAEIRKIYENGRGSVR |
| SRR1966989 | PPHNLREVAKAAITLIEQPKTTLDQLLDIVQGPDYPTEAEIITPRAEIRKIYENGRGSVR |
| SRR1967688 | PPHNLREVAKAAITLIEQPKTTLDQLLDIVQGPDYPTEAEIITPRAEIRKIYENGRGSVR |
| SRR1967733 | PPHNLREVAKAAITLIEQPKTTLDQLLDIVQGPDYPTEAEIITPRAEIRKIYENGRGSVR |
| SRR1967746 | PPHNLREVAKAAITLIEQPKTTLDQLLDIVQGPDYPTEAEIITPRAEIRKIYENGRGSVR |
| SRR1968341 | PPHNLREVAKAAITLIEQPKTTLDQLLDIVQGPDYPTEAEIITPRAEIRKIYENGRGSVR |
| SRR1968456 | PPHNLREVAKAAITLIEQPKTTLDQLLDIVQGPDYPTEAEIITPRAEIRKIYENGRGSVR |
| SRR1968465 | PPHNLREVAKAAITLIEQPKTTLDQLLDIVQGPDYPTEAEIITPRAEIRKIYENGRGSVR |
| SRR1968761 | PPHNLREVAKAAITLIEQPKTTLDQLLDIVQGPDYPTEAEIITPRAEIRKIYENGRGSVR |
| SRR1969047 | PPHNLREVAKAAITLIEQPKTTLDQLLDIVQGPDYPTEAEIITPRAEIRKIYENGRGSVR |
| SRR1969255 | PPHNLREVAKAAITLIEQPKTTLDQLLDIVQGPDYPTEAEIITPRAEIRKIYENGRGSVR |
| SRR1969412 | PPHNLREVAKAAITLIEQPKTTLDQLLDIVQGPDYPTEAEIITPRAEIRKIYENGRGSVR |
| SRR1969524 | PPHNLREVAKAAITLIEQPKTTLDQLLDIVQGPDYPTEAEIITPRAEIRKIYENGRGSVR |
| SRR1969584 | PPHNLREVAKAAITLIEQPKTTLDQLLDIVQGPDYPTEAEIITPRAEIRKIYENGRGSVR |
| SRR1969648 | PPHNLREVAKAAITLIEQPKTTLDQLLDIVQGPDYPTEAEIITPRAEIRKIYENGRGSVR |
| SRR1969804 | PPHNLREVAKAAITLIEQPKTTLDQLLDIVQGPDYPTEAEIITPRAEIRKIYENGRGSVR |
| SRR1970221 | PPHNLREVAKAAITLIEQPKTTLDQLLDIVQGPDYPTEAEIITPRAEIRKIYENGRGSVR |
| SRR1970268 | PPHNLREVAKAAITLIEQPKTTLDQLLDIVQGPDYPTEAEIITPRAEIRKIYENGRGSVR |
| SRR1965862 | PPHNLREVAKAAITLIEQPKTTLDQLLDIVQGPDYPTEAEIITPRAEIRKIYENGRGSVR |
| SRR1967363 | PPHNLREVAKAAITLIEQPKTTLDQLLDIVQGPDYPTEAEIITPRAEIRKIYENGRGSVR |
| SRR1968276 | PPHNLREVAKAAITLIEQPKTTLDQLLDIVQGPDYPTEAEIITPRAEIRKIYENGRGSVR |
| SRR1968967 | PPHNLREVAKAAITLIEQPKTTLDQLLDIVQGPDYPTEAEIITPRAEIRKIYENGRGSVR |
| SRR3321531 | PPHNLREVAKAAITLIEQPKTTLDQLLDIVQGPDYPTEAEIITPRAEIRKIYENGRGSVR |
| SRR3321883 | PPHNLREVAKAAITLIEQPKTTLDQLLDIVQGPDYPTEAEIITPRAEIRKIYENGRGSVR |
| SRR3322413 | PPHNLREVAKAAITLIEQPKTTLDQLLDIVQGPDYPTEAEIITPRAEIRKIYENGRGSVR |
| SRR3323012 | PPHNLREVAKAAITLIEQPKTTLDQLLDIVQGPDYPTEAEIITPRAEIRKIYENGRGSVR |
| SRR5194289 | PPHNLREVAKAAITLIEQPKTTLDQLLDIVQGPDYPTEAEIITPRAEIRKIYENGRGSVR |
| SRR7163798 | PPHNLREVAKAAITLIEQPKTTLDQLLDIVQGPDYPTEAEIITPRAEIRKIYENGRGSVR |
| SRR7172610 | PPHNLREVAKAAITLIEQPKTTLDQLLDIVQGPDYPTEAEIITPRAEIRKIYENGRGSVR |
| SRR7204568 | PPHNLREVAKAAITLIEQPKTTLDQLLDIVQGPDYPTEAEIITPRAEIRKIYENGRGSVR |
| SRR7223230 | PPHNLREVAKAAITLIEQPKTTLDQLLDIVQGPDYPTEAEIITPRAEIRKIYENGRGSVR |
| SRR7230675 | PPHNLREVAKAAITLIEQPKTTLDQLLDIVQGPDYPTEAEIITPRAEIRKIYENGRGSVR |
| SRR7278056 | PPHNLREVAKAAITLIEQPKTTLDQLLDIVQGPDYPTEAEIITPRAEIRKIYENGRGSVR |
| SRR7278086 | PPHNLREVAKAAITLIEQPKTTLDQLLDIVQGPDYPTEAEIITPRAEIRKIYENGRGSVR |
| SRR7285841 | PPHNLREVAKAAITLIEQPKTTLDQLLDIVQGPDYPTEAEIITPRAEIRKIYENGRGSVR |
| SRR7292625 | PPHNLREVAKAAITLIEQPKTTLDQLLDIVQGPDYPTEAEIITPRAEIRKIYENGRGSVR |
| SRR7292665 | PPHNLREVAKAAITLIEQPKTTLDQLLDIVQGPDYPTEAEIITPRAEIRKIYENGRGSVR |
| SRR7297965 | PPHNLREVAKAAITLIEQPKTTLDQLLDIVQGPDYPTEAEIITPRAEIRKIYENGRGSVR |
| SRR7350726 | PPHNLREVAKAAITLIEQPKTTLDQLLDIVQGPDYPTEAEIITPRAEIRKIYENGRGSVR |
| SRR7410328 | PPHNLREVAKAAITLIEQPKTTLDQLLDIVQGPDYPTEAEIITPRAEIRKIYENGRGSVR |
| SRR7474665 | PPHNLREVAKAAITLIEQPKTTLDQLLDIVQGPDYPTEAEIITPRAEIRKIYENGRGSVR |
| SRR7523184 | PPHNLREVAKAAITLIEQPKTTLDQLLDIVQGPDYPTEAEIITPRAEIRKIYENGRGSVR |
| SRR7187264 | PPHNLREVAKAAITLIEQPKTTLDQLLDIVQGPDYPTEAEIITPRAEIRKIYENGRGSVR |
| SRR7204445 | PPHNLREVAKAAITLIEQPKTTLDQLLDIVQGPDYPTEAEIITPRAEIRKIYENGRGSVR |
| SRR7285641 | PPHNLREVAKAAITLIEQPKTTLDQLLDIVQGPDYPTEAEIITPRAEIRKIYENGRGSVR |
| SRR7286695 | PPHNLREVAKAAITLIEQPKTTLDQLLDIVQGPDYPTEAEIITPRAEIRKIYENGRGSVR |
| SRR7286705 | PPHNLREVAKAAITLIEQPKTTLDQLLDIVQGPDYPTEAEIITPRAEIRKIYENGRGSVR |
| SRR7292931 | PPHNLREVAKAAITLIEQPKTTLDQLLDIVQGPDYPTEAEIITPRAEIRKIYENGRGSVR |
| SRR7310349 | PPHNLREVAKAAITLIEQPKTTLDQLLDIVQGPDYPTEAEIITPRAEIRKIYENGRGSVR |
| SRR7351616 | PPHNLREVAKAAITLIEQPKTTLDQLLDIVQGPDYPTEAEIITPRAEIRKIYENGRGSVR |
| SRR7414818 | PPHNLREVAKAAITLIEQPKTTLDQLLDIVQGPDYPTEAEIITPRAEIRKIYENGRGSVR |
| SRR7426480 | PPHNLREVAKAAITLIEQPKTTLDQLLDIVQGPDYPTEAEIITPRAEIRKIYENGRGSVR |
| SRR5584105 | PPHNLREVAKAAITLIEQPKTTLDQLLDIVQGPDYPTEAEIITPRAEIRKIYENGRGSVR |
| SRR5584565 | PPHNLREVAKAAITLIEQPKTTLDQLLDIVQGPDYPTEAEIITPRAEIRKIYENGRGSVR |
| SRR5584614 | PPHNLREVAKAAITLIEQPKTTLDQLLDIVQGPDYPTEAEIITPRAEIRKIYENGRGSVR |
| SRR5631543 | PPHNLREVAKAAITLIEQPKTTLDQLLDIVQGPDYPTEAEIITPRAEIRKIYENGRGSVR |
| SRR5631553 | PPHNLREVAKAAITLIEQPKTTLDQLLDIVQGPDYPTEAEIITPRAEIRKIYENGRGSVR |
| SRR7123196 | PPHNLREVAKAAITLIEQPKTTLDQLLDIVQGPDYPTEAEIITPRAEIRKIYENGRGSVR |
| SRR7163819 | PPHNLREVAKAAITLIEQPKTTLDQLLDIVQGPDYPTEAEIITPRAEIRKIYENGRGSVR |
| SRR7163920 | PPHNLREVAKAAITLIEQPKTTLDQLLDIVQGPDYPTEAEIITPRAEIRKIYENGRGSVR |
| SRR7209528 | PPHNLREVAKAAITLIEQPKTTLDQLLDIVQGPDYPTEAEIITPRAEIRKIYENGRGSVR |
| SRR7249868 | PPHNLREVAKAAITLIEQPKTTLDQLLDIVQGPDYPTEAEIITPRAEIRKIYENGRGSVR |
| SRR7278088 | PPHNLREVAKAAITLIEQPKTTLDQLLDIVQGPDYPTEAEIITPRAEIRKIYENGRGSVR |
| SRR7285788 | PPHNLREVAKAAITLIEQPKTTLDQLLDIVQGPDYPTEAEIITPRAEIRKIYENGRGSVR |
| SRR7286789 | PPHNLREVAKAAITLIEQPKTTLDQLLDIVQGPDYPTEAEIITPRAEIRKIYENGRGSVR |
| SRR7286886 | PPHNLREVAKAAITLIEQPKTTLDQLLDIVQGPDYPTEAEIITPRAEIRKIYENGRGSVR |
| SRR7310632 | PPHNLREVAKAAITLIEQPKTTLDQLLDIVQGPDYPTEAEIITPRAEIRKIYENGRGSVR |
| SRR7350631 | PPHNLREVAKAAITLIEQPKTTLDQLLDIVQGPDYPTEAEIITPRAEIRKIYENGRGSVR |
| SRR7458741 | PPHNLREVAKAAITLIEQPKTTLDQLLDIVQGPDYPTEAEIITPRAEIRKIYENGRGSVR |
| SRR7480280 | PPHNLREVAKAAITLIEQPKTTLDQLLDIVQGPDYPTEAEIITPRAEIRKIYENGRGSVR |
| SRR7523660 | PPHNLREVAKAAITLIEQPKTTLDQLLDIVQGPDYPTEAEIITPRAEIRKIYENGRGSVR |
| SRR7523775 | PPHNLREVAKAAITLIEQPKTTLDQLLDIVQGPDYPTEAEIITPRAEIRKIYENGRGSVR |
| SRR7251101 | PPHNLREVAKAAITLIEQPKTTLDQLLDIVQGPDYPTEAEIITPRAEIRKIYENGRGSVR |
| SRR7284299 | PPHNLREVAKAAITLIEQPKTTLDQLLDIVQGPDYPTEAEIITPRAEIRKIYENGRGSVR |
| SRR7285738 | PPHNLREVAKAAITLIEQPKTTLDQLLDIVQGPDYPTEAEIITPRAEIRKIYENGRGSVR |
| SRR7310640 | PPHNLREVAKAAITLIEQPKTTLDQLLDIVQGPDYPTEAEIITPRAEIRKIYENGRGSVR |
| SRR7349159 | PPHNLREVAKAAITLIEQPKTTLDQLLDIVQGPDYPTEAEIITPRAEIRKIYENGRGSVR |
| SRR7474873 | PPHNLREVAKAAITLIEQPKTTLDQLLDIVQGPDYPTEAEIITPRAEIRKIYENGRGSVR |
| SRR7495689 | PPHNLREVAKAAITLIEQPKTTLDQLLDIVQGPDYPTEAEIITPRAEIRKIYENGRGSVR |
| SRR7495752 | PPHNLREVAKAAITLIEQPKTTLDQLLDIVQGPDYPTEAEIITPRAEIRKIYENGRGSVR |
| ------------------------------------------------------------------------------ | |
| S16BD08730 | MRAVWTKEDGAVVISALPHQVSGAKVLEQIAAQMRNKKLPMVDDLRDESDHENPTRLVIV |
| S18BD00684 | MRAVWTKEDGAVVISALPHQVSGAKVLEQIAAQMRNKKLPMVDDLRDESDHENPTRLVIV |
| S18BD03994 | MRAVWTKEDGAVVISALPHQVSGAKVLEQIAAQMRNKKLPMVDDLRDESDHENPTRLVIV |
| S18BD05011 | MRAVWTKEDGAVVISALPHQVSGAKVLEQIAAQMRNKKLPMVDDLRDESDHENPTRLVIV |
| RKI_16-03723 | MRAVWTKEDGAVVISALPHQVSGAKVLEQIAAQMRNKKLPMVDDLRDESDHENPTRLVIV |
| RKI_16-04315 | MRAVWTKEDGAVVISALPHQVSGAKVLEQIAAQMRNKKLPMVDDLRDESDHENPTRLVIV |
| RKI_17-02304 | MRAVWTKEDGAVVISALPHQVSGAKVLEQIAAQMRNKKLPMVDDLRDESDHENPTRLVIV |
| RKI_17-02411 | MRAVWTKEDGAVVISALPHQVSGAKVLEQIAAQMRNKKLPMVDDLRDESDHENPTRLVIV |
| RKI_17-02757 | MRAVWTKEDGAVVISALPHQVSGAKVLEQIAAQMRNKKLPMVDDLRDESDHENPTRLVIV |
| RKI_17-04797 | MRAVWTKEDGAVVISALPHQVSGAKVLEQIAAQMRNKKLPMVDDLRDESDHENPTRLVIV |
| RKI_17-06869 | MRAVWTKEDGAVVISALPHQVSGAKVLEQIAAQMRNKKLPMVDDLRDESDHENPTRLVIV |
| ERR2580277 | MRAVWTKEDGAVVISALPHQVSGAKVLEQIAAQMRNKKLPMVDDLRDESDHENPTRLVIV |
| ERR2580276 | MRAVWTKEDGAVVISALPHQVSGAKVLEQIAAQMRNKKLPMVDDLRDESDHENPTRLVIV |
| ERR2580273 | MRAVWTKEDGAVVISALPHQVSGAKVLEQIAAQMRNKKLPMVDDLRDESDHENPTRLVIV |
| ERR2580274 | MRAVWTKEDGAVVISALPHQVSGAKVLEQIAAQMRNKKLPMVDDLRDESDHENPTRLVIV |
| ERR2173656 | MRAVWTKEDGAVVISALPHQVSGAKVLEQIAAQMRNKKLPMVDDLRDESDHENPTRLVIV |
| 17041676 | MRAVWTKEDGAVVISALPHQVSGAKVLEQIAAQMRNKKLPMVDDLRDESDHENPTRLVIV |
| MT16-000061 | MRAVWTKEDGAVVISALPHQVSGAKVLEQIAAQMRNKKLPMVDDLRDESDHENPTRLVIV |
| MT16-019416 | MRAVWTKEDGAVVISALPHQVSGAKVLEQIAAQMRNKKLPMVDDLRDESDHENPTRLVIV |
| MT16-027865 | MRAVWTKEDGAVVISALPHQVSGAKVLEQIAAQMRNKKLPMVDDLRDESDHENPTRLVIV |
| MT16-031693 | MRAVWTKEDGAVVISALPHQVSGAKVLEQIAAQMRNKKLPMVDDLRDESDHENPTRLVIV |
| MT16-040253 | MRAVWTKEDGAVVISALPHQVSGAKVLEQIAAQMRNKKLPMVDDLRDESDHENPTRLVIV |
| MT16-045379 | MRAVWTKEDGAVVISALPHQVSGAKVLEQIAAQMRNKKLPMVDDLRDESDHENPTRLVIV |
| MT16-442728 | MRAVWTKEDGAVVISALPHQVSGAKVLEQIAAQMRNKKLPMVDDLRDESDHENPTRLVIV |
| MT16-462857 | MRAVWTKEDGAVVISALPHQVSGAKVLEQIAAQMRNKKLPMVDDLRDESDHENPTRLVIV |
| MT16-480196 | MRAVWTKEDGAVVISALPHQVSGAKVLEQIAAQMRNKKLPMVDDLRDESDHENPTRLVIV |
| MT16-861555 | MRAVWTKEDGAVVISALPHQVSGAKVLEQIAAQMRNKKLPMVDDLRDESDHENPTRLVIV |
| MT17-076833 | MRAVWTKEDGAVVISALPHQVSGAKVLEQIAAQMRNKKLPMVDDLRDESDHENPTRLVIV |
| MT17-110677 | MRAVWTKEDGAVVISALPHQVSGAKVLEQIAAQMRNKKLPMVDDLRDESDHENPTRLVIV |
| MT17-131730 | MRAVWTKEDGAVVISALPHQVSGAKVLEQIAAQMRNKKLPMVDDLRDESDHENPTRLVIV |
| MT17-140890 | MRAVWTKEDGAVVISALPHQVSGAKVLEQIAAQMRNKKLPMVDDLRDESDHENPTRLVIV |
| MT17-141840 | MRAVWTKEDGAVVISALPHQVSGAKVLEQIAAQMRNKKLPMVDDLRDESDHENPTRLVIV |
| MT17-152488 | MRAVWTKEDGAVVISALPHQVSGAKVLEQIAAQMRNKKLPMVDDLRDESDHENPTRLVIV |
| MT17-157311 | MRAVWTKEDGAVVISALPHQVSGAKVLEQIAAQMRNKKLPMVDDLRDESDHENPTRLVIV |
| MT17-161645 | MRAVWTKEDGAVVISALPHQVSGAKVLEQIAAQMRNKKLPMVDDLRDESDHENPTRLVIV |
| MT17-167951 | MRAVWTKEDGAVVISALPHQVSGAKVLEQIAAQMRNKKLPMVDDLRDESDHENPTRLVIV |
| MT18-217732 | MRAVWTKEDGAVVISALPHQVSGAKVLEQIAAQMRNKKLPMVDDLRDESDHENPTRLVIV |
| MT18-252580 | MRAVWTKEDGAVVISALPHQVSGAKVLEQIAAQMRNKKLPMVDDLRDESDHENPTRLVIV |
| RIVM_H_2009-01 | MRAVWTKEDGAVVISALPHQVSGAKVLEQIAAQMRNKKLPMVDDLRDESDHENPTRLVIV |
| RIVM_H_2010-01 | MRAVWTKEDGAVVISALPHQVSGAKVLEQIAAQMRNKKLPMVDDLRDESDHENPTRLVIV |
| RIVM_H_2010-02 | MRAVWTKEDGAVVISALPHQVSGAKVLEQIAAQMRNKKLPMVDDLRDESDHENPTRLVIV |
| RIVM_H_2011-01 | MRAVWTKEDGAVVISALPHQVSGAKVLEQIAAQMRNKKLPMVDDLRDESDHENPTRLVIV |
| RIVM_H_2011-02 | MRAVWTKEDGAVVISALPHQVSGAKVLEQIAAQMRNKKLPMVDDLRDESDHENPTRLVIV |
| RIVM_H_2011-03 | MRAVWTKEDGAVVISALPHQVSGAKVLEQIAAQMRNKKLPMVDDLRDESDHENPTRLVIV |
| RIVM_H_2013-01 | MRAVWTKEDGAVVISALPHQVSGAKVLEQIAAQMRNKKLPMVDDLRDESDHENPTRLVIV |
| RIVM_H_2013-02 | MRAVWTKEDGAVVISALPHQVSGAKVLEQIAAQMRNKKLPMVDDLRDESDHENPTRLVIV |
| RIVM_H_2014-01 | MRAVWTKEDGAVVISALPHQVSGAKVLEQIAAQMRNKKLPMVDDLRDESDHENPTRLVIV |
| RIVM_H_2014-02 | MRAVWTKEDGAVVISALPHQVSGAKVLEQIAAQMRNKKLPMVDDLRDESDHENPTRLVIV |
| RIVM_H_2016-01 | MRAVWTKEDGAVVISALPHQVSGAKVLEQIAAQMRNKKLPMVDDLRDESDHENPTRLVIV |
| RIVM_H_2016-02 | MRAVWTKEDGAVVISALPHQVSGAKVLEQIAAQMRNKKLPMVDDLRDESDHENPTRLVIV |
| RIVM_H_2016-03 | MRAVWTKEDGAVVISALPHQVSGAKVLEQIAAQMRNKKLPMVDDLRDESDHENPTRLVIV |
| RIVM_H_2016-04 | MRAVWTKEDGAVVISALPHQVSGAKVLEQIAAQMRNKKLPMVDDLRDESDHENPTRLVIV |
| RIVM_H_2016-05 | MRAVWTKEDGAVVISALPHQVSGAKVLEQIAAQMRNKKLPMVDDLRDESDHENPTRLVIV |
| RIVM_H_2016-06 | MRAVWTKEDGAVVISALPHQVSGAKVLEQIAAQMRNKKLPMVDDLRDESDHENPTRLVIV |
| RIVM_H_2016-07 | MRAVWTKEDGAVVISALPHQVSGAKVLEQIAAQMRNKKLPMVDDLRDESDHENPTRLVIV |
| RIVM_H_2016-08 | MRAVWTKEDGAVVISALPHQVSGAKVLEQIAAQMRNKKLPMVDDLRDESDHENPTRLVIV |
| RIVM_H_2016-09 | MRAVWTKEDGAVVISALPHQVSGAKVLEQIAAQMRNKKLPMVDDLRDESDHENPTRLVIV |
| RIVM_H_2016-10 | MRAVWTKEDGAVVISALPHQVSGAKVLEQIAAQMRNKKLPMVDDLRDESDHENPTRLVIV |
| RIVM_H_2016-11 | MRAVWTKEDGAVVISALPHQVSGAKVLEQIAAQMRNKKLPMVDDLRDESDHENPTRLVIV |
| RIVM_H_2016-12 | MRAVWTKEDGAVVISALPHQVSGAKVLEQIAAQMRNKKLPMVDDLRDESDHENPTRLVIV |
| RIVM_H_2016-13 | MRAVWTKEDGAVVISALPHQVSGAKVLEQIAAQMRNKKLPMVDDLRDESDHENPTRLVIV |
| RIVM_H_2016-14 | MRAVWTKEDGAVVISALPHQVSGAKVLEQIAAQMRNKKLPMVDDLRDESDHENPTRLVIV |
| RIVM_H_2016-15 | MRAVWTKEDGAVVISALPHQVSGAKVLEQIAAQMRNKKLPMVDDLRDESDHENPTRLVIV |
| RIVM_H_2017-01 | MRAVWTKEDGAVVISALPHQVSGAKVLEQIAAQMRNKKLPMVDDLRDESDHENPTRLVIV |
| RIVM_H_2017-02 | MRAVWTKEDGAVVISALPHQVSGAKVLEQIAAQMRNKKLPMVDDLRDESDHENPTRLVIV |
| RIVM_H_2017-03 | MRAVWTKEDGAVVISALPHQVSGAKVLEQIAAQMRNKKLPMVDDLRDESDHENPTRLVIV |
| RIVM_H_2017-04 | MRAVWTKEDGAVVISALPHQVSGAKVLEQIAAQMRNKKLPMVDDLRDESDHENPTRLVIV |
| RIVM_H_2017-05 | MRAVWTKEDGAVVISALPHQVSGAKVLEQIAAQMRNKKLPMVDDLRDESDHENPTRLVIV |
| RIVM_H_2017-06 | MRAVWTKEDGAVVISALPHQVSGAKVLEQIAAQMRNKKLPMVDDLRDESDHENPTRLVIV |
| RIVM_H_2017-07 | MRAVWTKEDGAVVISALPHQVSGAKVLEQIAAQMRNKKLPMVDDLRDESDHENPTRLVIV |
| RIVM_H_2017-08 | MRAVWTKEDGAVVISALPHQVSGAKVLEQIAAQMRNKKLPMVDDLRDESDHENPTRLVIV |
| RIVM_H_2017-09 | MRAVWTKEDGAVVISALPHQVSGAKVLEQIAAQMRNKKLPMVDDLRDESDHENPTRLVIV |
| RIVM_H_2017-10 | MRAVWTKEDGAVVISALPHQVSGAKVLEQIAAQMRNKKLPMVDDLRDESDHENPTRLVIV |
| RIVM_H_2017-11 | MRAVWTKEDGAVVISALPHQVSGAKVLEQIAAQMRNKKLPMVDDLRDESDHENPTRLVIV |
| RIVM_H_2017-12 | MRAVWTKEDGAVVISALPHQVSGAKVLEQIAAQMRNKKLPMVDDLRDESDHENPTRLVIV |
| RIVM_H_2017-13 | MRAVWTKEDGAVVISALPHQVSGAKVLEQIAAQMRNKKLPMVDDLRDESDHENPTRLVIV |
| RIVM_H_2017-14 | MRAVWTKEDGAVVISALPHQVSGAKVLEQIAAQMRNKKLPMVDDLRDESDHENPTRLVIV |
| RIVM_H_2017-15 | MRAVWTKEDGAVVISALPHQVSGAKVLEQIAAQMRNKKLPMVDDLRDESDHENPTRLVIV |
| RIVM_H_2017-16 | MRAVWTKEDGAVVISALPHQVSGAKVLEQIAAQMRNKKLPMVDDLRDESDHENPTRLVIV |
| RIVM_H_2017-17 | MRAVWTKEDGAVVISALPHQVSGAKVLEQIAAQMRNKKLPMVDDLRDESDHENPTRLVIV |
| RIVM_H_2017-18 | MRAVWTKEDGAVVISALPHQVSGAKVLEQIAAQMRNKKLPMVDDLRDESDHENPTRLVIV |
| RIVM_H_2017-19 | MRAVWTKEDGAVVISALPHQVSGAKVLEQIAAQMRNKKLPMVDDLRDESDHENPTRLVIV |
| 15EP001483 | MRAVWTKEDGAVVISALPHQVSGAKVLEQIAAQMRNKKLPMVDDLRDESDHENPTRLVIV |
| 17EP002363 | MRAVWTKEDGAVVISALPHQVSGAKVLEQIAAQMRNKKLPMVDDLRDESDHENPTRLVIV |
| S_0812_17 | MRAVWTKEDGAVVISALPHQVSGAKVLEQIAAQMRNKKLPMVDDLRDESDHENPTRLVIV |
| SRR1957844 | MRAVWTKEDGAVVISALPHQVSGAKVLEQIAAQMRNKKLPMVDDLRDESDHENPTRLVIV |
| SRR1958654 | MRAVWTKEDGAVVISALPHQVSGAKVLEQIAAQMRNKKLPMVDDLRDESDHENPTRLVIV |
| SRR1965077 | MRAVWTKEDGAVVISALPHQVSGAKVLEQIAAQMRNKKLPMVDDLRDESDHENPTRLVIV |
| SRR1966369 | MRAVWTKEDGAVVISALPHQVSGAKVLEQIAAQMRNKKLPMVDDLRDESDHENPTRLVIV |
| SRR1967117 | MRAVWTKEDGAVVISALPHQVSGAKVLEQIAAQMRNKKLPMVDDLRDESDHENPTRLVIV |
| SRR1967922 | MRAVWTKEDGAVVISALPHQVSGAKVLEQIAAQMRNKKLPMVDDLRDESDHENPTRLVIV |
| SRR8704720 | MRAVWTKEDGAVVISALPHQVSGAKVLEQIAAQMRNKKLPMVDDLRDESDHENPTRLVIV |
| SRR7216071 | MRAVWTKEDGAVVISALPHQVSGAKVLEQIAAQMRNKKLPMVDDLRDESDHENPTRLVIV |
| SRR7349175 | MRAVWTKEDGAVVISALPHQVSGAKVLEQIAAQMRNKKLPMVDDLRDESDHENPTRLVIV |
| SRR7523148 | MRAVWTKEDGAVVISALPHQVSGAKVLEQIAAQMRNKKLPMVDDLRDESDHENPTRLVIV |
| SRR7523854 | MRAVWTKEDGAVVISALPHQVSGAKVLEQIAAQMRNKKLPMVDDLRDESDHENPTRLVIV |
| 313865 | MRAVWTKEDGAVVISALPHQVSGAKVLEQIAAQMRNKKLPMVDDLRDESDHENPTRLVIV |
| SRR7277793 | MRAVWTKEDGAVVISALPHQVSGAKVLEQIAAQMRNKKLPMVDDLRDESDHENPTRLVIV |
| SRR7343877 | MRAVWTKEDGAVVISALPHQVSGAKVLEQIAAQMRNKKLPMVDDLRDESDHENPTRLVIV |
| SRR7351477 | MRAVWTKEDGAVVISALPHQVSGAKVLEQIAAQMRNKKLPMVDDLRDESDHENPTRLVIV |
| SRR5583183 | MRAVWTKEDGAVVISALPHQVSGAKVLEQIAAQMRNKKLPMVDDLRDESDHENPTRLVIV |
| SRR5585240 | MRAVWTKEDGAVVISALPHQVSGAKVLEQIAAQMRNKKLPMVDDLRDESDHENPTRLVIV |
| SRR7284317 | MRAVWTKEDGAVVISALPHQVSGAKVLEQIAAQMRNKKLPMVDDLRDESDHENPTRLVIV |
| SRR7299161 | MRAVWTKEDGAVVISALPHQVSGAKVLEQIAAQMRNKKLPMVDDLRDESDHENPTRLVIV |
| SRR7401730 | MRAVWTKEDGAVVISALPHQVSGAKVLEQIAAQMRNKKLPMVDDLRDESDHENPTRLVIV |
| SRR7469092 | MRAVWTKEDGAVVISALPHQVSGAKVLEQIAAQMRNKKLPMVDDLRDESDHENPTRLVIV |
| SRR7879556 | MRAVWTKEDGAVVISALPHQVSGAKVLEQIAAQMRNKKLPMVDDLRDESDHENPTRLVIV |
| SRR8526100 | MRAVWTKEDGAVVISALPHQVSGAKVLEQIAAQMRNKKLPMVDDLRDESDHENPTRLVIV |
| SRR8553991 | MRAVWTKEDGAVVISALPHQVSGAKVLEQIAAQMRNKKLPMVDDLRDESDHENPTRLVIV |
| SRR7842487 | MRAVWTKEDGAVVISALPHQVSGAKVLEQIAAQMRNKKLPMVDDLRDESDHENPTRLVIV |
| SRR8054524 | MRAVWTKEDGAVVISALPHQVSGAKVLEQIAAQMRNKKLPMVDDLRDESDHENPTRLVIV |
| SRR8054525 | MRAVWTKEDGAVVISALPHQVSGAKVLEQIAAQMRNKKLPMVDDLRDESDHENPTRLVIV |
| SRR8524733 | MRAVWTKEDGAVVISALPHQVSGAKVLEQIAAQMRNKKLPMVDDLRDESDHENPTRLVIV |
| SRR4093291 | MRAVWTKEDGAVVISALPHQVSGAKVLEQIAAQMRNKKLPMVDDLRDESDHENPTRLVIV |
| SRR4245549 | MRAVWTKEDGAVVISALPHQVSGAKVLEQIAAQMRNKKLPMVDDLRDESDHENPTRLVIV |
| SRR3057154 | MRAVWTKEDGAVVISALPHQVSGAKVLEQIAAQMRNKKLPMVDDLRDESDHENPTRLVIV |
| SRR1726150 | MRAVWTKEDGAVVISALPHQVSGAKVLEQIAAQMRNKKLPMVDDLRDESDHENPTRLVIV |
| SRR1996141 | MRAVWTKEDGAVVISALPHQVSGAKVLEQIAAQMRNKKLPMVDDLRDESDHENPTRLVIV |
| SRR1107842 | MRAVWTKEDGAVVISALPHQVSGAKVLEQIAAQMRNKKLPMVDDLRDESDHENPTRLVIV |
| SRR1157587 | MRAVWTKEDGAVVISALPHQVSGAKVLEQIAAQMRNKKLPMVDDLRDESDHENPTRLVIV |
| SRR3027706 | MRAVWTKEDGAVVISALPHQVSGAKVLEQIAAQMRNKKLPMVDDLRDESDHENPTRLVIV |
| SRR3027707 | MRAVWTKEDGAVVISALPHQVSGAKVLEQIAAQMRNKKLPMVDDLRDESDHENPTRLVIV |
| SRR3027708 | MRAVWTKEDGAVVISALPHQVSGAKVLEQIAAQMRNKKLPMVDDLRDESDHENPTRLVIV |
| SRR3027710 | MRAVWTKEDGAVVISALPHQVSGAKVLEQIAAQMRNKKLPMVDDLRDESDHENPTRLVIV |
| SRR3027711 | MRAVWTKEDGAVVISALPHQVSGAKVLEQIAAQMRNKKLPMVDDLRDESDHENPTRLVIV |
| SRR3027716 | MRAVWTKEDGAVVISALPHQVSGAKVLEQIAAQMRNKKLPMVDDLRDESDHENPTRLVIV |
| SRR3027717 | MRAVWTKEDGAVVISALPHQVSGAKVLEQIAAQMRNKKLPMVDDLRDESDHENPTRLVIV |
| SRR3027719 | MRAVWTKEDGAVVISALPHQVSGAKVLEQIAAQMRNKKLPMVDDLRDESDHENPTRLVIV |
| SRR3027721 | MRAVWTKEDGAVVISALPHQVSGAKVLEQIAAQMRNKKLPMVDDLRDESDHENPTRLVIV |
| SRR3027723 | MRAVWTKEDGAVVISALPHQVSGAKVLEQIAAQMRNKKLPMVDDLRDESDHENPTRLVIV |
| SRR3115978 | MRAVWTKEDGAVVISALPHQVSGAKVLEQIAAQMRNKKLPMVDDLRDESDHENPTRLVIV |
| SRR2534093 | MRAVWTKEDGAVVISALPHQVSGAKVLEQIAAQMRNKKLPMVDDLRDESDHENPTRLVIV |
| SRR2534094 | MRAVWTKEDGAVVISALPHQVSGAKVLEQIAAQMRNKKLPMVDDLRDESDHENPTRLVIV |
| SRR2534095 | MRAVWTKEDGAVVISALPHQVSGAKVLEQIAAQMRNKKLPMVDDLRDESDHENPTRLVIV |
| SRR2534108 | MRAVWTKEDGAVVISALPHQVSGAKVLEQIAAQMRNKKLPMVDDLRDESDHENPTRLVIV |
| SRR1106464 | MRAVWTKEDGAVVISALPHQVSGAKVLEQIAAQMRNKKLPMVDDLRDESDHENPTRLVIV |
| SRR1106463 | MRAVWTKEDGAVVISALPHQVSGAKVLEQIAAQMRNKKLPMVDDLRDESDHENPTRLVIV |
| SRR6949610 | MRAVWTKEDGAVVISALPHQVSGAKVLEQIAAQMRNKKLPMVDDLRDESDHENPTRLVIV |
| SRR6950452 | MRAVWTKEDGAVVISALPHQVSGAKVLEQIAAQMRNKKLPMVDDLRDESDHENPTRLVIV |
| ERR2019831 | MRAVWTKEDGAVVISALPHQVSGAKVLEQIAAQMRNKKLPMVDDLRDESDHENPTRLVIV |
| SRR2085693 | MRAVWTKEDGAVVISALPHQVSGAKVLEQIAAQMRNKKLPMVDDLRDESDHENPTRLVIV |
| SRR2086898 | MRAVWTKEDGAVVISALPHQVSGAKVLEQIAAQMRNKKLPMVDDLRDESDHENPTRLVIV |
| SRR2175312 | MRAVWTKEDGAVVISALPHQVSGAKVLEQIAAQMRNKKLPMVDDLRDESDHENPTRLVIV |
| SRR2175360 | MRAVWTKEDGAVVISALPHQVSGAKVLEQIAAQMRNKKLPMVDDLRDESDHENPTRLVIV |
| SRR5231997 | MRAVWTKEDGAVVISALPHQVSGAKVLEQIAAQMRNKKLPMVDDLRDESDHENPTRLVIV |
| SRR5232003 | MRAVWTKEDGAVVISALPHQVSGAKVLEQIAAQMRNKKLPMVDDLRDESDHENPTRLVIV |
| SRR5232015 | MRAVWTKEDGAVVISALPHQVSGAKVLEQIAAQMRNKKLPMVDDLRDESDHENPTRLVIV |
| SRR949434 | MRAVWTKEDGAVVISALPHQVSGAKVLEQIAAQMRNKKLPMVDDLRDESDHENPTRLVIV |
| SRR3216575 | MRAVWTKEDGAVVISALPHQVSGAKVLEQIAAQMRNKKLPMVDDLRDESDHENPTRLVIV |
| SRR5205342 | MRAVWTKEDGAVVISALPHQVSGAKVLEQIAAQMRNKKLPMVDDLRDESDHENPTRLVIV |
| SRR1501669 | MRAVWTKEDGAVVISALPHQVSGAKVLEQIAAQMRNKKLPMVDDLRDESDHENPTRLVIV |
| SRR5209740 | MRAVWTKEDGAVVISALPHQVSGAKVLEQIAAQMRNKKLPMVDDLRDESDHENPTRLVIV |
| SRR3240355 | MRAVWTKEDGAVVISALPHQVSGAKVLEQIAAQMRNKKLPMVDDLRDESDHENPTRLVIV |
| SRR3392777 | MRAVWTKEDGAVVISALPHQVSGAKVLEQIAAQMRNKKLPMVDDLRDESDHENPTRLVIV |
| SRR3593671 | MRAVWTKEDGAVVISALPHQVSGAKVLEQIAAQMRNKKLPMVDDLRDESDHENPTRLVIV |
| SRR5413290 | MRAVWTKEDGAVVISALPHQVSGAKVLEQIAAQMRNKKLPMVDDLRDESDHENPTRLVIV |
| SRR5590269 | MRAVWTKEDGAVVISALPHQVSGAKVLEQIAAQMRNKKLPMVDDLRDESDHENPTRLVIV |
| SRR5812103 | MRAVWTKEDGAVVISALPHQVSGAKVLEQIAAQMRNKKLPMVDDLRDESDHENPTRLVIV |
| SRR2830941 | MRAVWTKEDGAVVISALPHQVSGAKVLEQIAAQMRNKKLPMVDDLRDESDHENPTRLVIV |
| SRR2830966 | MRAVWTKEDGAVVISALPHQVSGAKVLEQIAAQMRNKKLPMVDDLRDESDHENPTRLVIV |
| SRR3137270 | MRAVWTKEDGAVVISALPHQVSGAKVLEQIAAQMRNKKLPMVDDLRDESDHENPTRLVIV |
| SRR3137271 | MRAVWTKEDGAVVISALPHQVSGAKVLEQIAAQMRNKKLPMVDDLRDESDHENPTRLVIV |
| ERR526807 | MRAVWTKEDGAVVISALPHQVSGAKVLEQIAAQMRNKKLPMVDDLRDESDHENPTRLVIV |
| ERR2197922 | MRAVWTKEDGAVVISALPHQVSGAKVLEQIAAQMRNKKLPMVDDLRDESDHENPTRLVIV |
| ERR2197923 | MRAVWTKEDGAVVISALPHQVSGAKVLEQIAAQMRNKKLPMVDDLRDESDHENPTRLVIV |
| ERR2197924 | MRAVWTKEDGAVVISALPHQVSGAKVLEQIAAQMRNKKLPMVDDLRDESDHENPTRLVIV |
| ERR2197925 | MRAVWTKEDGAVVISALPHQVSGAKVLEQIAAQMRNKKLPMVDDLRDESDHENPTRLVIV |
| ERR2197927 | MRAVWTKEDGAVVISALPHQVSGAKVLEQIAAQMRNKKLPMVDDLRDESDHENPTRLVIV |
| ERR2197929 | MRAVWTKEDGAVVISALPHQVSGAKVLEQIAAQMRNKKLPMVDDLRDESDHENPTRLVIV |
| SRR1648149 | MRAVWTKEDGAVVISALPHQVSGAKVLEQIAAQMRNKKLPMVDDLRDESDHENPTRLVIV |
| SRR1048299 | MRAVWTKEDGAVVISALPHQVSGAKVLEQIAAQMRNKKLPMVDDLRDESDHENPTRLVIV |
| SRR1300677 | MRAVWTKEDGAVVISALPHQVSGAKVLEQIAAQMRNKKLPMVDDLRDESDHENPTRLVIV |
| SRR1288356 | MRAVWTKEDGAVVISALPHQVSGAKVLEQIAAQMRNKKLPMVDDLRDESDHENPTRLVIV |
| SRR7426190 | MRAVWTKEDGAVVISALPHQVSGAKVLEQIAAQMRNKKLPMVDDLRDESDHENPTRLVIV |
| SRR7426192 | MRAVWTKEDGAVVISALPHQVSGAKVLEQIAAQMRNKKLPMVDDLRDESDHENPTRLVIV |
| SRR7426193 | MRAVWTKEDGAVVISALPHQVSGAKVLEQIAAQMRNKKLPMVDDLRDESDHENPTRLVIV |
| SRR7441832 | MRAVWTKEDGAVVISALPHQVSGAKVLEQIAAQMRNKKLPMVDDLRDESDHENPTRLVIV |
| SRR7426179 | MRAVWTKEDGAVVISALPHQVSGAKVLEQIAAQMRNKKLPMVDDLRDESDHENPTRLVIV |
| SRR7439238 | MRAVWTKEDGAVVISALPHQVSGAKVLEQIAAQMRNKKLPMVDDLRDESDHENPTRLVIV |
| SRR7439244 | MRAVWTKEDGAVVISALPHQVSGAKVLEQIAAQMRNKKLPMVDDLRDESDHENPTRLVIV |
| SRR7439259 | MRAVWTKEDGAVVISALPHQVSGAKVLEQIAAQMRNKKLPMVDDLRDESDHENPTRLVIV |
| SRR7439260 | MRAVWTKEDGAVVISALPHQVSGAKVLEQIAAQMRNKKLPMVDDLRDESDHENPTRLVIV |
| SRR7441786 | MRAVWTKEDGAVVISALPHQVSGAKVLEQIAAQMRNKKLPMVDDLRDESDHENPTRLVIV |
| SRR7441797 | MRAVWTKEDGAVVISALPHQVSGAKVLEQIAAQMRNKKLPMVDDLRDESDHENPTRLVIV |
| ERR1759093 | MRAVWTKEDGAVVISALPHQVSGAKVLEQIAAQMRNKKLPMVDDLRDESDHENPTRLVIV |
| ERR2580275 | MRAVWTKEDGAVVISALPHQVSGAKVLEQIAAQMRNKKLPMVDDLRDESDHENPTRLVIV |
| ERR1759204 | MRAVWTKEDGAVVISALPHQVSGAKVLEQIAAQMRNKKLPMVDDLRDESDHENPTRLVIV |
| SRR1300699 | MRAVWTKEDGAVVISALPHQVSGAKVLEQIAAQMRNKKLPMVDDLRDESDHENPTRLVIV |
| S_0825_17 | MRAVWTKEDGAVVISALPHQVSGAKVLEQIAAQMRNKKLPMVDDLRDESDHENPTRLVIV |
| SRR1958215 | MRAVWTKEDGAVVISALPHQVSGAKVLEQIAAQMRNKKLPMVDDLRDESDHENPTRLVIV |
| SRR1958540 | MRAVWTKEDGAVVISALPHQVSGAKVLEQIAAQMRNKKLPMVDDLRDESDHENPTRLVIV |
| SRR1958636 | MRAVWTKEDGAVVISALPHQVSGAKVLEQIAAQMRNKKLPMVDDLRDESDHENPTRLVIV |
| SRR1959422 | MRAVWTKEDGAVVISALPHQVSGAKVLEQIAAQMRNKKLPMVDDLRDESDHENPTRLVIV |
| SRR1959427 | MRAVWTKEDGAVVISALPHQVSGAKVLEQIAAQMRNKKLPMVDDLRDESDHENPTRLVIV |
| SRR1960226 | MRAVWTKEDGAVVISALPHQVSGAKVLEQIAAQMRNKKLPMVDDLRDESDHENPTRLVIV |
| SRR1963498 | MRAVWTKEDGAVVISALPHQVSGAKVLEQIAAQMRNKKLPMVDDLRDESDHENPTRLVIV |
| SRR1965947 | MRAVWTKEDGAVVISALPHQVSGAKVLEQIAAQMRNKKLPMVDDLRDESDHENPTRLVIV |
| SRR1966125 | MRAVWTKEDGAVVISALPHQVSGAKVLEQIAAQMRNKKLPMVDDLRDESDHENPTRLVIV |
| SRR1966330 | MRAVWTKEDGAVVISALPHQVSGAKVLEQIAAQMRNKKLPMVDDLRDESDHENPTRLVIV |
| SRR1966565 | MRAVWTKEDGAVVISALPHQVSGAKVLEQIAAQMRNKKLPMVDDLRDESDHENPTRLVIV |
| SRR1966864 | MRAVWTKEDGAVVISALPHQVSGAKVLEQIAAQMRNKKLPMVDDLRDESDHENPTRLVIV |
| SRR1966989 | MRAVWTKEDGAVVISALPHQVSGAKVLEQIAAQMRNKKLPMVDDLRDESDHENPTRLVIV |
| SRR1967688 | MRAVWTKEDGAVVISALPHQVSGAKVLEQIAAQMRNKKLPMVDDLRDESDHENPTRLVIV |
| SRR1967733 | MRAVWTKEDGAVVISALPHQVSGAKVLEQIAAQMRNKKLPMVDDLRDESDHENPTRLVIV |
| SRR1967746 | MRAVWTKEDGAVVISALPHQVSGAKVLEQIAAQMRNKKLPMVDDLRDESDHENPTRLVIV |
| SRR1968341 | MRAVWTKEDGAVVISALPHQVSGAKVLEQIAAQMRNKKLPMVDDLRDESDHENPTRLVIV |
| SRR1968456 | MRAVWTKEDGAVVISALPHQVSGAKVLEQIAAQMRNKKLPMVDDLRDESDHENPTRLVIV |
| SRR1968465 | MRAVWTKEDGAVVISALPHQVSGAKVLEQIAAQMRNKKLPMVDDLRDESDHENPTRLVIV |
| SRR1968761 | MRAVWTKEDGAVVISALPHQVSGAKVLEQIAAQMRNKKLPMVDDLRDESDHENPTRLVIV |
| SRR1969047 | MRAVWTKEDGAVVISALPHQVSGAKVLEQIAAQMRNKKLPMVDDLRDESDHENPTRLVIV |
| SRR1969255 | MRAVWTKEDGAVVISALPHQVSGAKVLEQIAAQMRNKKLPMVDDLRDESDHENPTRLVIV |
| SRR1969412 | MRAVWTKEDGAVVISALPHQVSGAKVLEQIAAQMRNKKLPMVDDLRDESDHENPTRLVIV |
| SRR1969524 | MRAVWTKEDGAVVISALPHQVSGAKVLEQIAAQMRNKKLPMVDDLRDESDHENPTRLVIV |
| SRR1969584 | MRAVWTKEDGAVVISALPHQVSGAKVLEQIAAQMRNKKLPMVDDLRDESDHENPTRLVIV |
| SRR1969648 | MRAVWTKEDGAVVISALPHQVSGAKVLEQIAAQMRNKKLPMVDDLRDESDHENPTRLVIV |
| SRR1969804 | MRAVWTKEDGAVVISALPHQVSGAKVLEQIAAQMRNKKLPMVDDLRDESDHENPTRLVIV |
| SRR1970221 | MRAVWTKEDGAVVISALPHQVSGAKVLEQIAAQMRNKKLPMVDDLRDESDHENPTRLVIV |
| SRR1970268 | MRAVWTKEDGAVVISALPHQVSGAKVLEQIAAQMRNKKLPMVDDLRDESDHENPTRLVIV |
| SRR1965862 | MRAVWTKEDGAVVISALPHQVSGAKVLEQIAAQMRNKKLPMVDDLRDESDHENPTRLVIV |
| SRR1967363 | MRAVWTKEDGAVVISALPHQVSGAKVLEQIAAQMRNKKLPMVDDLRDESDHENPTRLVIV |
| SRR1968276 | MRAVWTKEDGAVVISALPHQVSGAKVLEQIAAQMRNKKLPMVDDLRDESDHENPTRLVIV |
| SRR1968967 | MRAVWTKEDGAVVISALPHQVSGAKVLEQIAAQMRNKKLPMVDDLRDESDHENPTRLVIV |
| SRR3321531 | MRAVWTKEDGAVVISALPHQVSGAKVLEQIAAQMRNKKLPMVDDLRDESDHENPTRLVIV |
| SRR3321883 | MRAVWTKEDGAVVISALPHQVSGAKVLEQIAAQMRNKKLPMVDDLRDESDHENPTRLVIV |
| SRR3322413 | MRAVWTKEDGAVVISALPHQVSGAKVLEQIAAQMRNKKLPMVDDLRDESDHENPTRLVIV |
| SRR3323012 | MRAVWTKEDGAVVISALPHQVSGAKVLEQIAAQMRNKKLPMVDDLRDESDHENPTRLVIV |
| SRR5194289 | MRAVWTKEDGAVVISALPHQVSGAKVLEQIAAQMRNKKLPMVDDLRDESDHENPTRLVIV |
| SRR7163798 | MRAVWTKEDGAVVISALPHQVSGAKVLEQIAAQMRNKKLPMVDDLRDESDHENPTRLVIV |
| SRR7172610 | MRAVWTKEDGAVVISALPHQVSGAKVLEQIAAQMRNKKLPMVDDLRDESDHENPTRLVIV |
| SRR7204568 | MRAVWTKEDGAVVISALPHQVSGAKVLEQIAAQMRNKKLPMVDDLRDESDHENPTRLVIV |
| SRR7223230 | MRAVWTKEDGAVVISALPHQVSGAKVLEQIAAQMRNKKLPMVDDLRDESDHENPTRLVIV |
| SRR7230675 | MRAVWTKEDGAVVISALPHQVSGAKVLEQIAAQMRNKKLPMVDDLRDESDHENPTRLVIV |
| SRR7278056 | MRAVWTKEDGAVVISALPHQVSGAKVLEQIAAQMRNKKLPMVDDLRDESDHENPTRLVIV |
| SRR7278086 | MRAVWTKEDGAVVISALPHQVSGAKVLEQIAAQMRNKKLPMVDDLRDESDHENPTRLVIV |
| SRR7285841 | MRAVWTKEDGAVVISALPHQVSGAKVLEQIAAQMRNKKLPMVDDLRDESDHENPTRLVIV |
| SRR7292625 | MRAVWTKEDGAVVISALPHQVSGAKVLEQIAAQMRNKKLPMVDDLRDESDHENPTRLVIV |
| SRR7292665 | MRAVWTKEDGAVVISALPHQVSGAKVLEQIAAQMRNKKLPMVDDLRDESDHENPTRLVIV |
| SRR7297965 | MRAVWTKEDGAVVISALPHQVSGAKVLEQIAAQMRNKKLPMVDDLRDESDHENPTRLVIV |
| SRR7350726 | MRAVWTKEDGAVVISALPHQVSGAKVLEQIAAQMRNKKLPMVDDLRDESDHENPTRLVIV |
| SRR7410328 | MRAVWTKEDGAVVISALPHQVSGAKVLEQIAAQMRNKKLPMVDDLRDESDHENPTRLVIV |
| SRR7474665 | MRAVWTKEDGAVVISALPHQVSGAKVLEQIAAQMRNKKLPMVDDLRDESDHENPTRLVIV |
| SRR7523184 | MRAVWTKEDGAVVISALPHQVSGAKVLEQIAAQMRNKKLPMVDDLRDESDHENPTRLVIV |
| SRR7187264 | MRAVWTKEDGAVVISALPHQVSGAKVLEQIAAQMRNKKLPMVDDLRDESDHENPTRLVIV |
| SRR7204445 | MRAVWTKEDGAVVISALPHQVSGAKVLEQIAAQMRNKKLPMVDDLRDESDHENPTRLVIV |
| SRR7285641 | MRAVWTKEDGAVVISALPHQVSGAKVLEQIAAQMRNKKLPMVDDLRDESDHENPTRLVIV |
| SRR7286695 | MRAVWTKEDGAVVISALPHQVSGAKVLEQIAAQMRNKKLPMVDDLRDESDHENPTRLVIV |
| SRR7286705 | MRAVWTKEDGAVVISALPHQVSGAKVLEQIAAQMRNKKLPMVDDLRDESDHENPTRLVIV |
| SRR7292931 | MRAVWTKEDGAVVISALPHQVSGAKVLEQIAAQMRNKKLPMVDDLRDESDHENPTRLVIV |
| SRR7310349 | MRAVWTKEDGAVVISALPHQVSGAKVLEQIAAQMRNKKLPMVDDLRDESDHENPTRLVIV |
| SRR7351616 | MRAVWTKEDGAVVISALPHQVSGAKVLEQIAAQMRNKKLPMVDDLRDESDHENPTRLVIV |
| SRR7414818 | MRAVWTKEDGAVVISALPHQVSGAKVLEQIAAQMRNKKLPMVDDLRDESDHENPTRLVIV |
| SRR7426480 | MRAVWTKEDGAVVISALPHQVSGAKVLEQIAAQMRNKKLPMVDDLRDESDHENPTRLVIV |
| SRR5584105 | MRAVWTKEDGAVVISALPHQVSGAKVLEQIAAQMRNKKLPMVDDLRDESDHENPTRLVIV |
| SRR5584565 | MRAVWTKEDGAVVISALPHQVSGAKVLEQIAAQMRNKKLPMVDDLRDESDHENPTRLVIV |
| SRR5584614 | MRAVWTKEDGAVVISALPHQVSGAKVLEQIAAQMRNKKLPMVDDLRDESDHENPTRLVIV |
| SRR5631543 | MRAVWTKEDGAVVISALPHQVSGAKVLEQIAAQMRNKKLPMVDDLRDESDHENPTRLVIV |
| SRR5631553 | MRAVWTKEDGAVVISALPHQVSGAKVLEQIAAQMRNKKLPMVDDLRDESDHENPTRLVIV |
| SRR7123196 | MRAVWTKEDGAVVISALPHQVSGAKVLEQIAAQMRNKKLPMVDDLRDESDHENPTRLVIV |
| SRR7163819 | MRAVWTKEDGAVVISALPHQVSGAKVLEQIAAQMRNKKLPMVDDLRDESDHENPTRLVIV |
| SRR7163920 | MRAVWTKEDGAVVISALPHQVSGAKVLEQIAAQMRNKKLPMVDDLRDESDHENPTRLVIV |
| SRR7209528 | MRAVWTKEDGAVVISALPHQVSGAKVLEQIAAQMRNKKLPMVDDLRDESDHENPTRLVIV |
| SRR7249868 | MRAVWTKEDGAVVISALPHQVSGAKVLEQIAAQMRNKKLPMVDDLRDESDHENPTRLVIV |
| SRR7278088 | MRAVWTKEDGAVVISALPHQVSGAKVLEQIAAQMRNKKLPMVDDLRDESDHENPTRLVIV |
| SRR7285788 | MRAVWTKEDGAVVISALPHQVSGAKVLEQIAAQMRNKKLPMVDDLRDESDHENPTRLVIV |
| SRR7286789 | MRAVWTKEDGAVVISALPHQVSGAKVLEQIAAQMRNKKLPMVDDLRDESDHENPTRLVIV |
| SRR7286886 | MRAVWTKEDGAVVISALPHQVSGAKVLEQIAAQMRNKKLPMVDDLRDESDHENPTRLVIV |
| SRR7310632 | MRAVWTKEDGAVVISALPHQVSGAKVLEQIAAQMRNKKLPMVDDLRDESDHENPTRLVIV |
| SRR7350631 | MRAVWTKEDGAVVISALPHQVSGAKVLEQIAAQMRNKKLPMVDDLRDESDHENPTRLVIV |
| SRR7458741 | MRAVWTKEDGAVVISALPHQVSGAKVLEQIAAQMRNKKLPMVDDLRDESDHENPTRLVIV |
| SRR7480280 | MRAVWTKEDGAVVISALPHQVSGAKVLEQIAAQMRNKKLPMVDDLRDESDHENPTRLVIV |
| SRR7523660 | MRAVWTKEDGAVVISALPHQVSGAKVLEQIAAQMRNKKLPMVDDLRDESDHENPTRLVIV |
| SRR7523775 | MRAVWTKEDGAVVISALPHQVSGAKVLEQIAAQMRNKKLPMVDDLRDESDHENPTRLVIV |
| SRR7251101 | MRAVWTKEDGAVVISALPHQVSGAKVLEQIAAQMRNKKLPMVDDLRDESDHENPTRLVIV |
| SRR7284299 | MRAVWTKEDGAVVISALPHQVSGAKVLEQIAAQMRNKKLPMVDDLRDESDHENPTRLVIV |
| SRR7285738 | MRAVWTKEDGAVVISALPHQVSGAKVLEQIAAQMRNKKLPMVDDLRDESDHENPTRLVIV |
| SRR7310640 | MRAVWTKEDGAVVISALPHQVSGAKVLEQIAAQMRNKKLPMVDDLRDESDHENPTRLVIV |
| SRR7349159 | MRAVWTKEDGAVVISALPHQVSGAKVLEQIAAQMRNKKLPMVDDLRDESDHENPTRLVIV |
| SRR7474873 | MRAVWTKEDGAVVISALPHQVSGAKVLEQIAAQMRNKKLPMVDDLRDESDHENPTRLVIV |
| SRR7495689 | MRAVWTKEDGAVVISALPHQVSGAKVLEQIAAQMRNKKLPMVDDLRDESDHENPTRLVIV |
| SRR7495752 | MRAVWTKEDGAVVISALPHQVSGAKVLEQIAAQMRNKKLPMVDDLRDESDHENPTRLVIV |
| ------------------------------------------------------------------------------ | |
| S16BD08730 | PRSNRVDMEQVMNHLFATTDLEKSYRINLNMIGLDGRPAVKNLLEILTEWLAFRRDTVRR |
| S18BD00684 | PRSNRVDMEQVMNHLFATTDLEKSYRINLNMIGLDGRPAVKNLLEILTEWLAFRRDTVRR |
| S18BD03994 | PRSNRVDMEQVMNHLFATTDLEKSYRINLNMIGLDGRPAVKNLLEILTEWLAFRRDTVRR |
| S18BD05011 | PRSNRVDMEQVMNHLFATTDLEKSYRINLNMIGLDGRPAVKNLLEILTEWLAFRRDTVRR |
| RKI_16-03723 | PRSNRVDMEQVMNHLFATTDLEKSYRINLNMIGLDGRPAVKNLLEILTEWLAFRRDTVRR |
| RKI_16-04315 | PRSNRVDMEQVMNHLFATTDLEKSYRINLNMIGLDGRPAVKNLLEILTEWLAFRRDTVRR |
| RKI_17-02304 | PRSNRVDMEQVMNHLFATTDLEKSYRINLNMIGLDGRPAVKNLLEILTEWLAFRRDTVRR |
| RKI_17-02411 | PRSNRVDMEQVMNHLFATTDLEKSYRINLNMIGLDGRPAVKNLLEILTEWLAFRRDTVRR |
| RKI_17-02757 | PRSNRVDMEQVMNHLFATTDLEKSYRINLNMIGLDGRPAVKNLLEILTEWLAFRRDTVRR |
| RKI_17-04797 | PRSNRVDMEQVMNHLFATTDLEKSYRINLNMIGLDGRPAVKNLLEILTEWLAFRRDTVRR |
| RKI_17-06869 | PRSNRVDMEQVMNHLFATTDLEKSYRINLNMIGLDGRPAVKNLLEILTEWLAFRRDTVRR |
| ERR2580277 | PRSNRVDMEQVMNHLFATTDLEKSYRINLNMIGLDGRPAVKNLLEILTEWLAFRRDTVRR |
| ERR2580276 | PRSNRVDMEQVMNHLFATTDLEKSYRINLNMIGLDGRPAVKNLLEILTEWLAFRRDTVRR |
| ERR2580273 | PRSNRVDMEQVMNHLFATTDLEKSYRINLNMIGLDGRPAVKNLLEILTEWLAFRRDTVRR |
| ERR2580274 | PRSNRVDMEQVMNHLFATTDLEKSYRINLNMIGLDGRPAVKNLLEILTEWLAFRRDTVRR |
| ERR2173656 | PRSNRVDMEQVMNHLFATTDLEKSYRINLNMIGLDGRPAVKNLLEILTEWLAFRRDTVRR |
| 17041676 | PRSNRVDMEQVMNHLFATTDLEKSYRINLNMIGLDGRPAVKNLLEILTEWLAFRRDTVRR |
| MT16-000061 | PRSNRVDMEQVMNHLFATTDLEKSYRINLNMIGLDGRPAVKNLLEILTEWLAFRRDTVRR |
| MT16-019416 | PRSNRVDMEQVMNHLFATTDLEKSYRINLNMIGLDGRPAVKNLLEILTEWLAFRRDTVRR |
| MT16-027865 | PRSNRVDMEQVMNHLFATTDLEKSYRINLNMIGLDGRPAVKNLLEILTEWLAFRRDTVRR |
| MT16-031693 | PRSNRVDMEQVMNHLFATTDLEKSYRINLNMIGLDGRPAVKNLLEILTEWLAFRRDTVRR |
| MT16-040253 | PRSNRVDMEQVMNHLFATTDLEKSYRINLNMIGLDGRPAVKNLLEILTEWLAFRRDTVRR |
| MT16-045379 | PRSNRVDMEQVMNHLFATTDLEKSYRINLNMIGLDGRPAVKNLLEILTEWLAFRRDTVRR |
| MT16-442728 | PRSNRVDMEQVMNHLFATTDLEKSYRINLNMIGLDGRPAVKNLLEILTEWLAFRRDTVRR |
| MT16-462857 | PRSNRVDMEQVMNHLFATTDLEKSYRINLNMIGLDGRPAVKNLLEILTEWLAFRRDTVRR |
| MT16-480196 | PRSNRVDMEQVMNHLFATTDLEKSYRINLNMIGLDGRPAVKNLLEILTEWLAFRRDTVRR |
| MT16-861555 | PRSNRVDMEQVMNHLFATTDLEKSYRINLNMIGLDGRPAVKNLLEILTEWLAFRRDTVRR |
| MT17-076833 | PRSNRVDMEQVMNHLFATTDLEKSYRINLNMIGLDGRPAVKNLLEILTEWLAFRRDTVRR |
| MT17-110677 | PRSNRVDMEQVMNHLFATTDLEKSYRINLNMIGLDGRPAVKNLLEILTEWLAFRRDTVRR |
| MT17-131730 | PRSNRVDMEQVMNHLFATTDLEKSYRINLNMIGLDGRPAVKNLLEILTEWLAFRRDTVRR |
| MT17-140890 | PRSNRVDMEQVMNHLFATTDLEKSYRINLNMIGLDGRPAVKNLLEILTEWLAFRRDTVRR |
| MT17-141840 | PRSNRVDMEQVMNHLFATTDLEKSYRINLNMIGLDGRPAVKNLLEILTEWLAFRRDTVRR |
| MT17-152488 | PRSNRVDMEQVMNHLFATTDLEKSYRINLNMIGLDGRPAVKNLLEILTEWLAFRRDTVRR |
| MT17-157311 | PRSNRVDMEQVMNHLFATTDLEKSYRINLNMIGLDGRPAVKNLLEILTEWLAFRRDTVRR |
| MT17-161645 | PRSNRVDMEQVMNHLFATTDLEKSYRINLNMIGLDGRPAVKNLLEILTEWLAFRRDTVRR |
| MT17-167951 | PRSNRVDMEQVMNHLFATTDLEKSYRINLNMIGLDGRPAVKNLLEILTEWLAFRRDTVRR |
| MT18-217732 | PRSNRVDMEQVMNHLFATTDLEKSYRINLNMIGLDGRPAVKNLLEILTEWLAFRRDTVRR |
| MT18-252580 | PRSNRVDMEQVMNHLFATTDLEKSYRINLNMIGLDGRPAVKNLLEILTEWLAFRRDTVRR |
| RIVM_H_2009-01 | PRSNRVDMEQVMNHLFATTDLEKSYRINLNMIGLDGRPAVKNLLEILTEWLAFRRDTVRR |
| RIVM_H_2010-01 | PRSNRVDMEQVMNHLFATTDLEKSYRINLNMIGLDGRPAVKNLLEILTEWLAFRRDTVRR |
| RIVM_H_2010-02 | PRSNRVDMEQVMNHLFATTDLEKSYRINLNMIGLDGRPAVKNLLEILTEWLAFRRDTVRR |
| RIVM_H_2011-01 | PRSNRVDMEQVMNHLFATTDLEKSYRINLNMIGLDGRPAVKNLLEILTEWLAFRRDTVRR |
| RIVM_H_2011-02 | PRSNRVDMEQVMNHLFATTDLEKSYRINLNMIGLDGRPAVKNLLEILTEWLAFRRDTVRR |
| RIVM_H_2011-03 | PRSNRVDMEQVMNHLFATTDLEKSYRINLNMIGLDGRPAVKNLLEILTEWLAFRRDTVRR |
| RIVM_H_2013-01 | PRSNRVDMEQVMNHLFATTDLEKSYRINLNMIGLDGRPAVKNLLEILTEWLAFRRDTVRR |
| RIVM_H_2013-02 | PRSNRVDMEQVMNHLFATTDLEKSYRINLNMIGLDGRPAVKNLLEILTEWLAFRRDTVRR |
| RIVM_H_2014-01 | PRSNRVDMEQVMNHLFATTDLEKSYRINLNMIGLDGRPAVKNLLEILTEWLAFRRDTVRR |
| RIVM_H_2014-02 | PRSNRVDMEQVMNHLFATTDLEKSYRINLNMIGLDGRPAVKNLLEILTEWLAFRRDTVRR |
| RIVM_H_2016-01 | PRSNRVDMEQVMNHLFATTDLEKSYRINLNMIGLDGRPAVKNLLEILTEWLAFRRDTVRR |
| RIVM_H_2016-02 | PRSNRVDMEQVMNHLFATTDLEKSYRINLNMIGLDGRPAVKNLLEILTEWLAFRRDTVRR |
| RIVM_H_2016-03 | PRSNRVDMEQVMNHLFATTDLEKSYRINLNMIGLDGRPAVKNLLEILTEWLAFRRDTVRR |
| RIVM_H_2016-04 | PRSNRVDMEQVMNHLFATTDLEKSYRINLNMIGLDGRPAVKNLLEILTEWLAFRRDTVRR |
| RIVM_H_2016-05 | PRSNRVDMEQVMNHLFATTDLEKSYRINLNMIGLDGRPAVKNLLEILTEWLAFRRDTVRR |
| RIVM_H_2016-06 | PRSNRVDMEQVMNHLFATTDLEKSYRINLNMIGLDGRPAVKNLLEILTEWLAFRRDTVRR |
| RIVM_H_2016-07 | PRSNRVDMEQVMNHLFATTDLEKSYRINLNMIGLDGRPAVKNLLEILTEWLAFRRDTVRR |
| RIVM_H_2016-08 | PRSNRVDMEQVMNHLFATTDLEKSYRINLNMIGLDGRPAVKNLLEILTEWLAFRRDTVRR |
| RIVM_H_2016-09 | PRSNRVDMEQVMNHLFATTDLEKSYRINLNMIGLDGRPAVKNLLEILTEWLAFRRDTVRR |
| RIVM_H_2016-10 | PRSNRVDMEQVMNHLFATTDLEKSYRINLNMIGLDGRPAVKNLLEILTEWLAFRRDTVRR |
| RIVM_H_2016-11 | PRSNRVDMEQVMNHLFATTDLEKSYRINLNMIGLDGRPAVKNLLEILTEWLAFRRDTVRR |
| RIVM_H_2016-12 | PRSNRVDMEQVMNHLFATTDLEKSYRINLNMIGLDGRPAVKNLLEILTEWLAFRRDTVRR |
| RIVM_H_2016-13 | PRSNRVDMEQVMNHLFATTDLEKSYRINLNMIGLDGRPAVKNLLEILTEWLAFRRDTVRR |
| RIVM_H_2016-14 | PRSNRVDMEQVMNHLFATTDLEKSYRINLNMIGLDGRPAVKNLLEILTEWLAFRRDTVRR |
| RIVM_H_2016-15 | PRSNRVDMEQVMNHLFATTDLEKSYRINLNMIGLDGRPAVKNLLEILTEWLAFRRDTVRR |
| RIVM_H_2017-01 | PRSNRVDMEQVMNHLFATTDLEKSYRINLNMIGLDGRPAVKNLLEILTEWLAFRRDTVRR |
| RIVM_H_2017-02 | PRSNRVDMEQVMNHLFATTDLEKSYRINLNMIGLDGRPAVKNLLEILTEWLAFRRDTVRR |
| RIVM_H_2017-03 | PRSNRVDMEQVMNHLFATTDLEKSYRINLNMIGLDGRPAVKNLLEILTEWLAFRRDTVRR |
| RIVM_H_2017-04 | PRSNRVDMEQVMNHLFATTDLEKSYRINLNMIGLDGRPAVKNLLEILTEWLAFRRDTVRR |
| RIVM_H_2017-05 | PRSNRVDMEQVMNHLFATTDLEKSYRINLNMIGLDGRPAVKNLLEILTEWLAFRRDTVRR |
| RIVM_H_2017-06 | PRSNRVDMEQVMNHLFATTDLEKSYRINLNMIGLDGRPAVKNLLEILTEWLAFRRDTVRR |
| RIVM_H_2017-07 | PRSNRVDMEQVMNHLFATTDLEKSYRINLNMIGLDGRPAVKNLLEILTEWLAFRRDTVRR |
| RIVM_H_2017-08 | PRSNRVDMEQVMNHLFATTDLEKSYRINLNMIGLDGRPAVKNLLEILTEWLAFRRDTVRR |
| RIVM_H_2017-09 | PRSNRVDMEQVMNHLFATTDLEKSYRINLNMIGLDGRPAVKNLLEILTEWLAFRRDTVRR |
| RIVM_H_2017-10 | PRSNRVDMEQVMNHLFATTDLEKSYRINLNMIGLDGRPAVKNLLEILTEWLAFRRDTVRR |
| RIVM_H_2017-11 | PRSNRVDMEQVMNHLFATTDLEKSYRINLNMIGLDGRPAVKNLLEILTEWLAFRRDTVRR |
| RIVM_H_2017-12 | PRSNRVDMEQVMNHLFATTDLEKSYRINLNMIGLDGRPAVKNLLEILTEWLAFRRDTVRR |
| RIVM_H_2017-13 | PRSNRVDMEQVMNHLFATTDLEKSYRINLNMIGLDGRPAVKNLLEILTEWLAFRRDTVRR |
| RIVM_H_2017-14 | PRSNRVDMEQVMNHLFATTDLEKSYRINLNMIGLDGRPAVKNLLEILTEWLAFRRDTVRR |
| RIVM_H_2017-15 | PRSNRVDMEQVMNHLFATTDLEKSYRINLNMIGLDGRPAVKNLLEILTEWLAFRRDTVRR |
| RIVM_H_2017-16 | PRSNRVDMEQVMNHLFATTDLEKSYRINLNMIGLDGRPAVKNLLEILTEWLAFRRDTVRR |
| RIVM_H_2017-17 | PRSNRVDMEQVMNHLFATTDLEKSYRINLNMIGLDGRPAVKNLLEILTEWLAFRRDTVRR |
| RIVM_H_2017-18 | PRSNRVDMEQVMNHLFATTDLEKSYRINLNMIGLDGRPAVKNLLEILTEWLAFRRDTVRR |
| RIVM_H_2017-19 | PRSNRVDMEQVMNHLFATTDLEKSYRINLNMIGLDGRPAVKNLLEILTEWLAFRRDTVRR |
| 15EP001483 | PRSNRVDMEQVMNHLFATTDLEKSYRINLNMIGLDGRPAVKNLLEILTEWLAFRRDTVRR |
| 17EP002363 | PRSNRVDMEQVMNHLFATTDLEKSYRINLNMIGLDGRPAVKNLLEILTEWLAFRRDTVRR |
| S_0812_17 | PRSNRVDMEQVMNHLFATTDLEKSYRINLNMIGLDGRPAVKNLLEILTEWLAFRRDTVRR |
| SRR1957844 | PRSNRVDMEQVMNHLFATTDLEKSYRINLNMIGLDGRPAVKNLLEILTEWLAFRRDTVRR |
| SRR1958654 | PRSNRVDMEQVMNHLFATTDLEKSYRINLNMIGLDGRPAVKNLLEILTEWLAFRRDTVRR |
| SRR1965077 | PRSNRVDMEQVMNHLFATTDLEKSYRINLNMIGLDGRPAVKNLLEILTEWLAFRRDTVRR |
| SRR1966369 | PRSNRVDMEQVMNHLFATTDLEKSYRINLNMIGLDGRPAVKNLLEILTEWLAFRRDTVRR |
| SRR1967117 | PRSNRVDMEQVMNHLFATTDLEKSYRINLNMIGLDGRPAVKNLLEILTEWLAFRRDTVRR |
| SRR1967922 | PRSNRVDMEQVMNHLFATTDLEKSYRINLNMIGLDGRPAVKNLLEILTEWLAFRRDTVRR |
| SRR8704720 | PRSNRVDMEQVMNHLFATTDLEKSYRINLNMIGLDGRPAVKNLLEILTEWLAFRRDTVRR |
| SRR7216071 | PRSNRVDMEQVMNHLFATTDLEKSYRINLNMIGLDGRPAVKNLLEILTEWLAFRRDTVRR |
| SRR7349175 | PRSNRVDMEQVMNHLFATTDLEKSYRINLNMIGLDGRPAVKNLLEILTEWLAFRRDTVRR |
| SRR7523148 | PRSNRVDMEQVMNHLFATTDLEKSYRINLNMIGLDGRPAVKNLLEILTEWLAFRRDTVRR |
| SRR7523854 | PRSNRVDMEQVMNHLFATTDLEKSYRINLNMIGLDGRPAVKNLLEILTEWLAFRRDTVRR |
| 313865 | PRSNRVDMEQVMNHLFATTDLEKSYRINLNMIGLDGRPAVKNLLEILTEWLAFRRDTVRR |
| SRR7277793 | PRSNRVDMEQVMNHLFATTDLEKSYRINLNMIGLDGRPAVKNLLEILTEWLAFRRDTVRR |
| SRR7343877 | PRSNRVDMEQVMNHLFATTDLEKSYRINLNMIGLDGRPAVKNLLEILTEWLAFRRDTVRR |
| SRR7351477 | PRSNRVDMEQVMNHLFATTDLEKSYRINLNMIGLDGRPAVKNLLEILTEWLAFRRDTVRR |
| SRR5583183 | PRSNRVDMEQVMNHLFATTDLEKSYRINLNMIGLDGRPAVKNLLEILTEWLAFRRDTVRR |
| SRR5585240 | PRSNRVDMEQVMNHLFATTDLEKSYRINLNMIGLDGRPAVKNLLEILTEWLAFRRDTVRR |
| SRR7284317 | PRSNRVDMEQVMNHLFATTDLEKSYRINLNMIGLDGRPAVKNLLEILTEWLAFRRDTVRR |
| SRR7299161 | PRSNRVDMEQVMNHLFATTDLEKSYRINLNMIGLDGRPAVKNLLEILTEWLAFRRDTVRR |
| SRR7401730 | PRSNRVDMEQVMNHLFATTDLEKSYRINLNMIGLDGRPAVKNLLEILTEWLAFRRDTVRR |
| SRR7469092 | PRSNRVDMEQVMNHLFATTDLEKSYRINLNMIGLDGRPAVKNLLEILTEWLAFRRDTVRR |
| SRR7879556 | PRSNRVDMEQVMNHLFATTDLEKSYRINLNMIGLDGRPAVKNLLEILTEWLAFRRDTVRR |
| SRR8526100 | PRSNRVDMEQVMNHLFATTDLEKSYRINLNMIGLDGRPAVKNLLEILTEWLAFRRDTVRR |
| SRR8553991 | PRSNRVDMEQVMNHLFATTDLEKSYRINLNMIGLDGRPAVKNLLEILTEWLAFRRDTVRR |
| SRR7842487 | PRSNRVDMEQVMNHLFATTDLEKSYRINLNMIGLDGRPAVKNLLEILTEWLAFRRDTVRR |
| SRR8054524 | PRSNRVDMEQVMNHLFATTDLEKSYRINLNMIGLDGRPAVKNLLEILTEWLAFRRDTVRR |
| SRR8054525 | PRSNRVDMEQVMNHLFATTDLEKSYRINLNMIGLDGRPAVKNLLEILTEWLAFRRDTVRR |
| SRR8524733 | PRSNRVDMEQVMNHLFATTDLEKSYRINLNMIGLDGRPAVKNLLEILTEWLAFRRDTVRR |
| SRR4093291 | PRSNRVDMEQVMNHLFATTDLEKSYRINLNMIGLDGRPAVKNLLEILTEWLAFRRDTVRR |
| SRR4245549 | PRSNRVDMEQVMNHLFATTDLEKSYRINLNMIGLDGRPAVKNLLEILTEWLAFRRDTVRR |
| SRR3057154 | PRSNRVDMEQVMNHLFATTDLEKSYRINLNMIGLDGRPAVKNLLEILTEWLAFRRDTVRR |
| SRR1726150 | PRSNRVDMEQVMNHLFATTDLEKSYRINLNMIGLDGRPAVKNLLEILTEWLAFRRDTVRR |
| SRR1996141 | PRSNRVDMEQVMNHLFATTDLEKSYRINLNMIGLDGRPAVKNLLEILTEWLAFRRDTVRR |
| SRR1107842 | PRSNRVDMEQVMNHLFATTDLEKSYRINLNMIGLDGRPAVKNLLEILTEWLAFRRDTVRR |
| SRR1157587 | PRSNRVDMEQVMNHLFATTDLEKSYRINLNMIGLDGRPAVKNLLEILTEWLAFRRDTVRR |
| SRR3027706 | PRSNRVDMEQVMNHLFATTDLEKSYRINLNMIGLDGRPAVKNLLEILTEWLAFRRDTVRR |
| SRR3027707 | PRSNRVDMEQVMNHLFATTDLEKSYRINLNMIGLDGRPAVKNLLEILTEWLAFRRDTVRR |
| SRR3027708 | PRSNRVDMEQVMNHLFATTDLEKSYRINLNMIGLDGRPAVKNLLEILTEWLAFRRDTVRR |
| SRR3027710 | PRSNRVDMEQVMNHLFATTDLEKSYRINLNMIGLDGRPAVKNLLEILTEWLAFRRDTVRR |
| SRR3027711 | PRSNRVDMEQVMNHLFATTDLEKSYRINLNMIGLDGRPAVKNLLEILTEWLAFRRDTVRR |
| SRR3027716 | PRSNRVDMEQVMNHLFATTDLEKSYRINLNMIGLDGRPAVKNLLEILTEWLAFRRDTVRR |
| SRR3027717 | PRSNRVDMEQVMNHLFATTDLEKSYRINLNMIGLDGRPAVKNLLEILTEWLAFRRDTVRR |
| SRR3027719 | PRSNRVDMEQVMNHLFATTDLEKSYRINLNMIGLDGRPAVKNLLEILTEWLAFRRDTVRR |
| SRR3027721 | PRSNRVDMEQVMNHLFATTDLEKSYRINLNMIGLDGRPAVKNLLEILTEWLAFRRDTVRR |
| SRR3027723 | PRSNRVDMEQVMNHLFATTDLEKSYRINLNMIGLDGRPAVKNLLEILTEWLAFRRDTVRR |
| SRR3115978 | PRSNRVDMEQVMNHLFATTDLEKSYRINLNMIGLDGRPAVKNLLEILTEWLAFRRDTVRR |
| SRR2534093 | PRSNRVDMEQVMNHLFATTDLEKSYRINLNMIGLDGRPAVKNLLEILTEWLAFRRDTVRR |
| SRR2534094 | PRSNRVDMEQVMNHLFATTDLEKSYRINLNMIGLDGRPAVKNLLEILTEWLAFRRDTVRR |
| SRR2534095 | PRSNRVDMEQVMNHLFATTDLEKSYRINLNMIGLDGRPAVKNLLEILTEWLAFRRDTVRR |
| SRR2534108 | PRSNRVDMEQVMNHLFATTDLEKSYRINLNMIGLDGRPAVKNLLEILTEWLAFRRDTVRR |
| SRR1106464 | PRSNRVDMEQVMNHLFATTDLEKSYRINLNMIGLDGRPAVKNLLEILTEWLAFRRDTVRR |
| SRR1106463 | PRSNRVDMEQVMNHLFATTDLEKSYRINLNMIGLDGRPAVKNLLEILTEWLAFRRDTVRR |
| SRR6949610 | PRSNRVDMEQVMNHLFATTDLEKSYRINLNMIGLDGRPAVKNLLEILTEWLAFRRDTVRR |
| SRR6950452 | PRSNRVDMEQVMNHLFATTDLEKSYRINLNMIGLDGRPAVKNLLEILTEWLAFRRDTVRR |
| ERR2019831 | PRSNRVDMEQVMNHLFATTDLEKSYRINLNMIGLDGRPAVKNLLEILTEWLAFRRDTVRR |
| SRR2085693 | PRSNRVDMEQVMNHLFATTDLEKSYRINLNMIGLDGRPAVKNLLEILTEWLAFRRDTVRR |
| SRR2086898 | PRSNRVDMEQVMNHLFATTDLEKSYRINLNMIGLDGRPAVKNLLEILTEWLAFRRDTVRR |
| SRR2175312 | PRSNRVDMEQVMNHLFATTDLEKSYRINLNMIGLDGRPAVKNLLEILTEWLAFRRDTVRR |
| SRR2175360 | PRSNRVDMEQVMNHLFATTDLEKSYRINLNMIGLDGRPAVKNLLEILTEWLAFRRDTVRR |
| SRR5231997 | PRSNRVDMEQVMNHLFATTDLEKSYRINLNMIGLDGRPAVKNLLEILTEWLAFRRDTVRR |
| SRR5232003 | PRSNRVDMEQVMNHLFATTDLEKSYRINLNMIGLDGRPAVKNLLEILTEWLAFRRDTVRR |
| SRR5232015 | PRSNRVDMEQVMNHLFATTDLEKSYRINLNMIGLDGRPAVKNLLEILTEWLAFRRDTVRR |
| SRR949434 | PRSNRVDMEQVMNHLFATTDLEKSYRINLNMIGLDGRPAVKNLLEILTEWLAFRRDTVRR |
| SRR3216575 | PRSNRVDMEQVMNHLFATTDLEKSYRINLNMIGLDGRPAVKNLLEILTEWLAFRRDTVRR |
| SRR5205342 | PRSNRVDMEQVMNHLFATTDLEKSYRINLNMIGLDGRPAVKNLLEILTEWLAFRRDTVRR |
| SRR1501669 | PRSNRVDMEQVMNHLFATTDLEKSYRINLNMIGLDGRPAVKNLLEILTEWLAFRRDTVRR |
| SRR5209740 | PRSNRVDMEQVMNHLFATTDLEKSYRINLNMIGLDGRPAVKNLLEILTEWLAFRRDTVRR |
| SRR3240355 | PRSNRVDMEQVMNHLFATTDLEKSYRINLNMIGLDGRPAVKNLLEILTEWLAFRRDTVRR |
| SRR3392777 | PRSNRVDMEQVMNHLFATTDLEKSYRINLNMIGLDGRPAVKNLLEILTEWLAFRRDTVRR |
| SRR3593671 | PRSNRVDMEQVMNHLFATTDLEKSYRINLNMIGLDGRPAVKNLLEILTEWLAFRRDTVRR |
| SRR5413290 | PRSNRVDMEQVMNHLFATTDLEKSYRINLNMIGLDGRPAVKNLLEILTEWLAFRRDTVRR |
| SRR5590269 | PRSNRVDMEQVMNHLFATTDLEKSYRINLNMIGLDGRPAVKNLLEILTEWLAFRRDTVRR |
| SRR5812103 | PRSNRVDMEQVMNHLFATTDLEKSYRINLNMIGLDGRPAVKNLLEILTEWLAFRRDTVRR |
| SRR2830941 | PRSNRVDMEQVMNHLFATTDLEKSYRINLNMIGLDGRPAVKNLLEILTEWLAFRRDTVRR |
| SRR2830966 | PRSNRVDMEQVMNHLFATTDLEKSYRINLNMIGLDGRPAVKNLLEILTEWLAFRRDTVRR |
| SRR3137270 | PRSNRVDMEQVMNHLFATTDLEKSYRINLNMIGLDGRPAVKNLLEILTEWLAFRRDTVRR |
| SRR3137271 | PRSNRVDMEQVMNHLFATTDLEKSYRINLNMIGLDGRPAVKNLLEILTEWLAFRRDTVRR |
| ERR526807 | PRSNRVDMEQVMNHLFATTDLEKSYRINLNMIGLDGRPAVKNLLEILTEWLAFRRDTVRR |
| ERR2197922 | PRSNRVDMEQVMNHLFATTDLEKSYRINLNMIGLDGRPAVKNLLEILTEWLAFRRDTVRR |
| ERR2197923 | PRSNRVDMEQVMNHLFATTDLEKSYRINLNMIGLDGRPAVKNLLEILTEWLAFRRDTVRR |
| ERR2197924 | PRSNRVDMEQVMNHLFATTDLEKSYRINLNMIGLDGRPAVKNLLEILTEWLAFRRDTVRR |
| ERR2197925 | PRSNRVDMEQVMNHLFATTDLEKSYRINLNMIGLDGRPAVKNLLEILTEWLAFRRDTVRR |
| ERR2197927 | PRSNRVDMEQVMNHLFATTDLEKSYRINLNMIGLDGRPAVKNLLEILTEWLAFRRDTVRR |
| ERR2197929 | PRSNRVDMEQVMNHLFATTDLEKSYRINLNMIGLDGRPAVKNLLEILTEWLAFRRDTVRR |
| SRR1648149 | PRSNRVDMEQVMNHLFATTDLEKSYRINLNMIGLDGRPAVKNLLEILTEWLAFRRDTVRR |
| SRR1048299 | PRSNRVDMEQVMNHLFATTDLEKSYRINLNMIGLDGRPAVKNLLEILTEWLAFRRDTVRR |
| SRR1300677 | PRSNRVDMEQVMNHLFATTDLEKSYRINLNMIGLDGRPAVKNLLEILTEWLAFRRDTVRR |
| SRR1288356 | PRSNRVDMEQVMNHLFATTDLEKSYRINLNMIGLDGRPAVKNLLEILTEWLAFRRDTVRR |
| SRR7426190 | PRSNRVDMEQVMNHLFATTDLEKSYRINLNMIGLDGRPAVKNLLEILTEWLAFRRDTVRR |
| SRR7426192 | PRSNRVDMEQVMNHLFATTDLEKSYRINLNMIGLDGRPAVKNLLEILTEWLAFRRDTVRR |
| SRR7426193 | PRSNRVDMEQVMNHLFATTDLEKSYRINLNMIGLDGRPAVKNLLEILTEWLAFRRDTVRR |
| SRR7441832 | PRSNRVDMEQVMNHLFATTDLEKSYRINLNMIGLDGRPAVKNLLEILTEWLAFRRDTVRR |
| SRR7426179 | PRSNRVDMEQVMNHLFATTDLEKSYRINLNMIGLDGRPAVKNLLEILTEWLAFRRDTVRR |
| SRR7439238 | PRSNRVDMEQVMNHLFATTDLEKSYRINLNMIGLDGRPAVKNLLEILTEWLAFRRDTVRR |
| SRR7439244 | PRSNRVDMEQVMNHLFATTDLEKSYRINLNMIGLDGRPAVKNLLEILTEWLAFRRDTVRR |
| SRR7439259 | PRSNRVDMEQVMNHLFATTDLEKSYRINLNMIGLDGRPAVKNLLEILTEWLAFRRDTVRR |
| SRR7439260 | PRSNRVDMEQVMNHLFATTDLEKSYRINLNMIGLDGRPAVKNLLEILTEWLAFRRDTVRR |
| SRR7441786 | PRSNRVDMEQVMNHLFATTDLEKSYRINLNMIGLDGRPAVKNLLEILTEWLAFRRDTVRR |
| SRR7441797 | PRSNRVDMEQVMNHLFATTDLEKSYRINLNMIGLDGRPAVKNLLEILTEWLAFRRDTVRR |
| ERR1759093 | PRSNRVDMEQVMNHLFATTDLEKSYRINLNMIGLDGRPAVKNLLEILTEWLAFRRDTVRR |
| ERR2580275 | PRSNRVDMEQVMNHLFATTDLEKSYRINLNMIGLDGRPAVKNLLEILTEWLAFRRDTVRR |
| ERR1759204 | PRSNRVDMEQVMNHLFATTDLEKSYRINLNMIGLDGRPAVKNLLEILTEWLAFRRDTVRR |
| SRR1300699 | PRSNRVDMEQVMNHLFATTDLEKSYRINLNMIGLDGRPAVKNLLEILTEWLAFRRDTVRR |
| S_0825_17 | PRSNRVDMEQVMNHLFATTDLEKSYRINLNMIGLDGRPAVKNLLEILTEWLAFRRDTVRR |
| SRR1958215 | PRSNRVDMEQVMNHLFATTDLEKSYRINLNMIGLDGRPAVKNLLEILTEWLAFRRDTVRR |
| SRR1958540 | PRSNRVDMEQVMNHLFATTDLEKSYRINLNMIGLDGRPAVKNLLEILTEWLAFRRDTVRR |
| SRR1958636 | PRSNRVDMEQVMNHLFATTDLEKSYRINLNMIGLDGRPAVKNLLEILTEWLAFRRDTVRR |
| SRR1959422 | PRSNRVDMEQVMNHLFATTDLEKSYRINLNMIGLDGRPAVKNLLEILTEWLAFRRDTVRR |
| SRR1959427 | PRSNRVDMEQVMNHLFATTDLEKSYRINLNMIGLDGRPAVKNLLEILTEWLAFRRDTVRR |
| SRR1960226 | PRSNRVDMEQVMNHLFATTDLEKSYRINLNMIGLDGRPAVKNLLEILTEWLAFRRDTVRR |
| SRR1963498 | PRSNRVDMEQVMNHLFATTDLEKSYRINLNMIGLDGRPAVKNLLEILTEWLAFRRDTVRR |
| SRR1965947 | PRSNRVDMEQVMNHLFATTDLEKSYRINLNMIGLDGRPAVKNLLEILTEWLAFRRDTVRR |
| SRR1966125 | PRSNRVDMEQVMNHLFATTDLEKSYRINLNMIGLDGRPAVKNLLEILTEWLAFRRDTVRR |
| SRR1966330 | PRSNRVDMEQVMNHLFATTDLEKSYRINLNMIGLDGRPAVKNLLEILTEWLAFRRDTVRR |
| SRR1966565 | PRSNRVDMEQVMNHLFATTDLEKSYRINLNMIGLDGRPAVKNLLEILTEWLAFRRDTVRR |
| SRR1966864 | PRSNRVDMEQVMNHLFATTDLEKSYRINLNMIGLDGRPAVKNLLEILTEWLAFRRDTVRR |
| SRR1966989 | PRSNRVDMEQVMNHLFATTDLEKSYRINLNMIGLDGRPAVKNLLEILTEWLAFRRDTVRR |
| SRR1967688 | PRSNRVDMEQVMNHLFATTDLEKSYRINLNMIGLDGRPAVKNLLEILTEWLAFRRDTVRR |
| SRR1967733 | PRSNRVDMEQVMNHLFATTDLEKSYRINLNMIGLDGRPAVKNLLEILTEWLAFRRDTVRR |
| SRR1967746 | PRSNRVDMEQVMNHLFATTDLEKSYRINLNMIGLDGRPAVKNLLEILTEWLAFRRDTVRR |
| SRR1968341 | PRSNRVDMEQVMNHLFATTDLEKSYRINLNMIGLDGRPAVKNLLEILTEWLAFRRDTVRR |
| SRR1968456 | PRSNRVDMEQVMNHLFATTDLEKSYRINLNMIGLDGRPAVKNLLEILTEWLAFRRDTVRR |
| SRR1968465 | PRSNRVDMEQVMNHLFATTDLEKSYRINLNMIGLDGRPAVKNLLEILTEWLAFRRDTVRR |
| SRR1968761 | PRSNRVDMEQVMNHLFATTDLEKSYRINLNMIGLDGRPAVKNLLEILTEWLAFRRDTVRR |
| SRR1969047 | PRSNRVDMEQVMNHLFATTDLEKSYRINLNMIGLDGRPAVKNLLEILTEWLAFRRDTVRR |
| SRR1969255 | PRSNRVDMEQVMNHLFATTDLEKSYRINLNMIGLDGRPAVKNLLEILTEWLAFRRDTVRR |
| SRR1969412 | PRSNRVDMEQVMNHLFATTDLEKSYRINLNMIGLDGRPAVKNLLEILTEWLAFRRDTVRR |
| SRR1969524 | PRSNRVDMEQVMNHLFATTDLEKSYRINLNMIGLDGRPAVKNLLEILTEWLAFRRDTVRR |
| SRR1969584 | PRSNRVDMEQVMNHLFATTDLEKSYRINLNMIGLDGRPAVKNLLEILTEWLAFRRDTVRR |
| SRR1969648 | PRSNRVDMEQVMNHLFATTDLEKSYRINLNMIGLDGRPAVKNLLEILTEWLAFRRDTVRR |
| SRR1969804 | PRSNRVDMEQVMNHLFATTDLEKSYRINLNMIGLDGRPAVKNLLEILTEWLAFRRDTVRR |
| SRR1970221 | PRSNRVDMEQVMNHLFATTDLEKSYRINLNMIGLDGRPAVKNLLEILTEWLAFRRDTVRR |
| SRR1970268 | PRSNRVDMEQVMNHLFATTDLEKSYRINLNMIGLDGRPAVKNLLEILTEWLAFRRDTVRR |
| SRR1965862 | PRSNRVDMEQVMNHLFATTDLEKSYRINLNMIGLDGRPAVKNLLEILTEWLAFRRDTVRR |
| SRR1967363 | PRSNRVDMEQVMNHLFATTDLEKSYRINLNMIGLDGRPAVKNLLEILTEWLAFRRDTVRR |
| SRR1968276 | PRSNRVDMEQVMNHLFATTDLEKSYRINLNMIGLDGRPAVKNLLEILTEWLAFRRDTVRR |
| SRR1968967 | PRSNRVDMEQVMNHLFATTDLEKSYRINLNMIGLDGRPAVKNLLEILTEWLAFRRDTVRR |
| SRR3321531 | PRSNRVDMEQVMNHLFATTDLEKSYRINLNMIGLDGRPAVKNLLEILTEWLAFRRDTVRR |
| SRR3321883 | PRSNRVDMEQVMNHLFATTDLEKSYRINLNMIGLDGRPAVKNLLEILTEWLAFRRDTVRR |
| SRR3322413 | PRSNRVDMEQVMNHLFATTDLEKSYRINLNMIGLDGRPAVKNLLEILTEWLAFRRDTVRR |
| SRR3323012 | PRSNRVDMEQVMNHLFATTDLEKSYRINLNMIGLDGRPAVKNLLEILTEWLAFRRDTVRR |
| SRR5194289 | PRSNRVDMEQVMNHLFATTDLEKSYRINLNMIGLDGRPAVKNLLEILTEWLAFRRDTVRR |
| SRR7163798 | PRSNRVDMEQVMNHLFATTDLEKSYRINLNMIGLDGRPAVKNLLEILTEWLAFRRDTVRR |
| SRR7172610 | PRSNRVDMEQVMNHLFATTDLEKSYRINLNMIGLDGRPAVKNLLEILTEWLAFRRDTVRR |
| SRR7204568 | PRSNRVDMEQVMNHLFATTDLEKSYRINLNMIGLDGRPAVKNLLEILTEWLAFRRDTVRR |
| SRR7223230 | PRSNRVDMEQVMNHLFATTDLEKSYRINLNMIGLDGRPAVKNLLEILTEWLAFRRDTVRR |
| SRR7230675 | PRSNRVDMEQVMNHLFATTDLEKSYRINLNMIGLDGRPAVKNLLEILTEWLAFRRDTVRR |
| SRR7278056 | PRSNRVDMEQVMNHLFATTDLEKSYRINLNMIGLDGRPAVKNLLEILTEWLAFRRDTVRR |
| SRR7278086 | PRSNRVDMEQVMNHLFATTDLEKSYRINLNMIGLDGRPAVKNLLEILTEWLAFRRDTVRR |
| SRR7285841 | PRSNRVDMEQVMNHLFATTDLEKSYRINLNMIGLDGRPAVKNLLEILTEWLAFRRDTVRR |
| SRR7292625 | PRSNRVDMEQVMNHLFATTDLEKSYRINLNMIGLDGRPAVKNLLEILTEWLAFRRDTVRR |
| SRR7292665 | PRSNRVDMEQVMNHLFATTDLEKSYRINLNMIGLDGRPAVKNLLEILTEWLAFRRDTVRR |
| SRR7297965 | PRSNRVDMEQVMNHLFATTDLEKSYRINLNMIGLDGRPAVKNLLEILTEWLAFRRDTVRR |
| SRR7350726 | PRSNRVDMEQVMNHLFATTDLEKSYRINLNMIGLDGRPAVKNLLEILTEWLAFRRDTVRR |
| SRR7410328 | PRSNRVDMEQVMNHLFATTDLEKSYRINLNMIGLDGRPAVKNLLEILTEWLAFRRDTVRR |
| SRR7474665 | PRSNRVDMEQVMNHLFATTDLEKSYRINLNMIGLDGRPAVKNLLEILTEWLAFRRDTVRR |
| SRR7523184 | PRSNRVDMEQVMNHLFATTDLEKSYRINLNMIGLDGRPAVKNLLEILTEWLAFRRDTVRR |
| SRR7187264 | PRSNRVDMEQVMNHLFATTDLEKSYRINLNMIGLDGRPAVKNLLEILTEWLAFRRDTVRR |
| SRR7204445 | PRSNRVDMEQVMNHLFATTDLEKSYRINLNMIGLDGRPAVKNLLEILTEWLAFRRDTVRR |
| SRR7285641 | PRSNRVDMEQVMNHLFATTDLEKSYRINLNMIGLDGRPAVKNLLEILTEWLAFRRDTVRR |
| SRR7286695 | PRSNRVDMEQVMNHLFATTDLEKSYRINLNMIGLDGRPAVKNLLEILTEWLAFRRDTVRR |
| SRR7286705 | PRSNRVDMEQVMNHLFATTDLEKSYRINLNMIGLDGRPAVKNLLEILTEWLAFRRDTVRR |
| SRR7292931 | PRSNRVDMEQVMNHLFATTDLEKSYRINLNMIGLDGRPAVKNLLEILTEWLAFRRDTVRR |
| SRR7310349 | PRSNRVDMEQVMNHLFATTDLEKSYRINLNMIGLDGRPAVKNLLEILTEWLAFRRDTVRR |
| SRR7351616 | PRSNRVDMEQVMNHLFATTDLEKSYRINLNMIGLDGRPAVKNLLEILTEWLAFRRDTVRR |
| SRR7414818 | PRSNRVDMEQVMNHLFATTDLEKSYRINLNMIGLDGRPAVKNLLEILTEWLAFRRDTVRR |
| SRR7426480 | PRSNRVDMEQVMNHLFATTDLEKSYRINLNMIGLDGRPAVKNLLEILTEWLAFRRDTVRR |
| SRR5584105 | PRSNRVDMEQVMNHLFATTDLEKSYRINLNMIGLDGRPAVKNLLEILTEWLAFRRDTVRR |
| SRR5584565 | PRSNRVDMEQVMNHLFATTDLEKSYRINLNMIGLDGRPAVKNLLEILTEWLAFRRDTVRR |
| SRR5584614 | PRSNRVDMEQVMNHLFATTDLEKSYRINLNMIGLDGRPAVKNLLEILTEWLAFRRDTVRR |
| SRR5631543 | PRSNRVDMEQVMNHLFATTDLEKSYRINLNMIGLDGRPAVKNLLEILTEWLAFRRDTVRR |
| SRR5631553 | PRSNRVDMEQVMNHLFATTDLEKSYRINLNMIGLDGRPAVKNLLEILTEWLAFRRDTVRR |
| SRR7123196 | PRSNRVDMEQVMNHLFATTDLEKSYRINLNMIGLDGRPAVKNLLEILTEWLAFRRDTVRR |
| SRR7163819 | PRSNRVDMEQVMNHLFATTDLEKSYRINLNMIGLDGRPAVKNLLEILTEWLAFRRDTVRR |
| SRR7163920 | PRSNRVDMEQVMNHLFATTDLEKSYRINLNMIGLDGRPAVKNLLEILTEWLAFRRDTVRR |
| SRR7209528 | PRSNRVDMEQVMNHLFATTDLEKSYRINLNMIGLDGRPAVKNLLEILTEWLAFRRDTVRR |
| SRR7249868 | PRSNRVDMEQVMNHLFATTDLEKSYRINLNMIGLDGRPAVKNLLEILTEWLAFRRDTVRR |
| SRR7278088 | PRSNRVDMEQVMNHLFATTDLEKSYRINLNMIGLDGRPAVKNLLEILTEWLAFRRDTVRR |
| SRR7285788 | PRSNRVDMEQVMNHLFATTDLEKSYRINLNMIGLDGRPAVKNLLEILTEWLAFRRDTVRR |
| SRR7286789 | PRSNRVDMEQVMNHLFATTDLEKSYRINLNMIGLDGRPAVKNLLEILTEWLAFRRDTVRR |
| SRR7286886 | PRSNRVDMEQVMNHLFATTDLEKSYRINLNMIGLDGRPAVKNLLEILTEWLAFRRDTVRR |
| SRR7310632 | PRSNRVDMEQVMNHLFATTDLEKSYRINLNMIGLDGRPAVKNLLEILTEWLAFRRDTVRR |
| SRR7350631 | PRSNRVDMEQVMNHLFATTDLEKSYRINLNMIGLDGRPAVKNLLEILTEWLAFRRDTVRR |
| SRR7458741 | PRSNRVDMEQVMNHLFATTDLEKSYRINLNMIGLDGRPAVKNLLEILTEWLAFRRDTVRR |
| SRR7480280 | PRSNRVDMEQVMNHLFATTDLEKSYRINLNMIGLDGRPAVKNLLEILTEWLAFRRDTVRR |
| SRR7523660 | PRSNRVDMEQVMNHLFATTDLEKSYRINLNMIGLDGRPAVKNLLEILTEWLAFRRDTVRR |
| SRR7523775 | PRSNRVDMEQVMNHLFATTDLEKSYRINLNMIGLDGRPAVKNLLEILTEWLAFRRDTVRR |
| SRR7251101 | PRSNRVDMEQVMNHLFATTDLEKSYRINLNMIGLDGRPAVKNLLEILTEWLAFRRDTVRR |
| SRR7284299 | PRSNRVDMEQVMNHLFATTDLEKSYRINLNMIGLDGRPAVKNLLEILTEWLAFRRDTVRR |
| SRR7285738 | PRSNRVDMEQVMNHLFATTDLEKSYRINLNMIGLDGRPAVKNLLEILTEWLAFRRDTVRR |
| SRR7310640 | PRSNRVDMEQVMNHLFATTDLEKSYRINLNMIGLDGRPAVKNLLEILTEWLAFRRDTVRR |
| SRR7349159 | PRSNRVDMEQVMNHLFATTDLEKSYRINLNMIGLDGRPAVKNLLEILTEWLAFRRDTVRR |
| SRR7474873 | PRSNRVDMEQVMNHLFATTDLEKSYRINLNMIGLDGRPAVKNLLEILTEWLAFRRDTVRR |
| SRR7495689 | PRSNRVDMEQVMNHLFATTDLEKSYRINLNMIGLDGRPAVKNLLEILTEWLAFRRDTVRR |
| SRR7495752 | PRSNRVDMEQVMNHLFATTDLEKSYRINLNMIGLDGRPAVKNLLEILTEWLAFRRDTVRR |
| ------------------------------------------------------------------------------ | |
| S16BD08730 | RLNYRLEKVLKRLHILEGLLVAFLNIDEVIEIIRNEDEPKPALMSRFGISETQAEAILEL |
| S18BD00684 | RLNYRLEKVLKRLHILEGLLVAFLNIDEVIEIIRNEDEPKPALMSRFGISETQAEAILEL |
| S18BD03994 | RLNYRLEKVLKRLHILEGLLVAFLNIDEVIEIIRNEDEPKPALMSRFGISETQAEAILEL |
| S18BD05011 | RLNYRLEKVLKRLHILEGLLVAFLNIDEVIEIIRNEDEPKPALMSRFGISETQAEAILEL |
| RKI_16-03723 | RLNYRLEKVLKRLHILEGLLVAFLNIDEVIEIIRNEDEPKPALMSRFGISETQAEAILEL |
| RKI_16-04315 | RLNYRLEKVLKRLHILEGLLVAFLNIDEVIEIIRNEDEPKPALMSRFGISETQAEAILEL |
| RKI_17-02304 | RLNYRLEKVLKRLHILEGLLVAFLNIDEVIEIIRNEDEPKPALMSRFGISETQAEAILEL |
| RKI_17-02411 | RLNYRLEKVLKRLHILEGLLVAFLNIDEVIEIIRNEDEPKPALMSRFGISETQAEAILEL |
| RKI_17-02757 | RLNYRLEKVLKRLHILEGLLVAFLNIDEVIEIIRNEDEPKPALMSRFGISETQAEAILEL |
| RKI_17-04797 | RLNYRLEKVLKRLHILEGLLVAFLNIDEVIEIIRNEDEPKPALMSRFGISETQAEAILEL |
| RKI_17-06869 | RLNYRLEKVLKRLHILEGLLVAFLNIDEVIEIIRNEDEPKPALMSRFGISETQAEAILEL |
| ERR2580277 | RLNYRLEKVLKRLHILEGLLVAFLNIDEVIEIIRNEDEPKPALMSRFGISETQAEAILEL |
| ERR2580276 | RLNYRLEKVLKRLHILEGLLVAFLNIDEVIEIIRNEDEPKPALMSRFGISETQAEAILEL |
| ERR2580273 | RLNYRLEKVLKRLHILEGLLVAFLNIDEVIEIIRNEDEPKPALMSRFGISETQAEAILEL |
| ERR2580274 | RLNYRLEKVLKRLHILEGLLVAFLNIDEVIEIIRNEDEPKPALMSRFGISETQAEAILEL |
| ERR2173656 | RLNYRLEKVLKRLHILEGLLVAFLNIDEVIEIIRNEDEPKPALMSRFGISETQAEAILEL |
| 17041676 | RLNYRLEKVLKRLHILEGLLVAFLNIDEVIEIIRNEDEPKPALMSRFGISETQAEAILEL |
| MT16-000061 | RLNYRLEKVLKRLHILEGLLVAFLNIDEVIEIIRNEDEPKPALMSRFGISETQAEAILEL |
| MT16-019416 | RLNYRLEKVLKRLHILEGLLVAFLNIDEVIEIIRNEDEPKPALMSRFGISETQAEAILEL |
| MT16-027865 | RLNYRLEKVLKRLHILEGLLVAFLNIDEVIEIIRNEDEPKPALMSRFGISETQAEAILEL |
| MT16-031693 | RLNYRLEKVLKRLHILEGLLVAFLNIDEVIEIIRNEDEPKPALMSRFGISETQAEAILEL |
| MT16-040253 | RLNYRLEKVLKRLHILEGLLVAFLNIDEVIEIIRNEDEPKPALMSRFGISETQAEAILEL |
| MT16-045379 | RLNYRLEKVLKRLHILEGLLVAFLNIDEVIEIIRNEDEPKPALMSRFGISETQAEAILEL |
| MT16-442728 | RLNYRLEKVLKRLHILEGLLVAFLNIDEVIEIIRNEDEPKPALMSRFGISETQAEAILEL |
| MT16-462857 | RLNYRLEKVLKRLHILEGLLVAFLNIDEVIEIIRNEDEPKPALMSRFGISETQAEAILEL |
| MT16-480196 | RLNYRLEKVLKRLHILEGLLVAFLNIDEVIEIIRNEDEPKPALMSRFGISETQAEAILEL |
| MT16-861555 | RLNYRLEKVLKRLHILEGLLVAFLNIDEVIEIIRNEDEPKPALMSRFGISETQAEAILEL |
| MT17-076833 | RLNYRLEKVLKRLHILEGLLVAFLNIDEVIEIIRNEDEPKPALMSRFGISETQAEAILEL |
| MT17-110677 | RLNYRLEKVLKRLHILEGLLVAFLNIDEVIEIIRNEDEPKPALMSRFGISETQAEAILEL |
| MT17-131730 | RLNYRLEKVLKRLHILEGLLVAFLNIDEVIEIIRNEDEPKPALMSRFGISETQAEAILEL |
| MT17-140890 | RLNYRLEKVLKRLHILEGLLVAFLNIDEVIEIIRNEDEPKPALMSRFGISETQAEAILEL |
| MT17-141840 | RLNYRLEKVLKRLHILEGLLVAFLNIDEVIEIIRNEDEPKPALMSRFGISETQAEAILEL |
| MT17-152488 | RLNYRLEKVLKRLHILEGLLVAFLNIDEVIEIIRNEDEPKPALMSRFGISETQAEAILEL |
| MT17-157311 | RLNYRLEKVLKRLHILEGLLVAFLNIDEVIEIIRNEDEPKPALMSRFGISETQAEAILEL |
| MT17-161645 | RLNYRLEKVLKRLHILEGLLVAFLNIDEVIEIIRNEDEPKPALMSRFGISETQAEAILEL |
| MT17-167951 | RLNYRLEKVLKRLHILEGLLVAFLNIDEVIEIIRNEDEPKPALMSRFGISETQAEAILEL |
| MT18-217732 | RLNYRLEKVLKRLHILEGLLVAFLNIDEVIEIIRNEDEPKPALMSRFGISETQAEAILEL |
| MT18-252580 | RLNYRLEKVLKRLHILEGLLVAFLNIDEVIEIIRNEDEPKPALMSRFGISETQAEAILEL |
| RIVM_H_2009-01 | RLNYRLEKVLKRLHILEGLLVAFLNIDEVIEIIRNEDEPKPALMSRFGISETQAEAILEL |
| RIVM_H_2010-01 | RLNYRLEKVLKRLHILEGLLVAFLNIDEVIEIIRNEDEPKPALMSRFGISETQAEAILEL |
| RIVM_H_2010-02 | RLNYRLEKVLKRLHILEGLLVAFLNIDEVIEIIRNEDEPKPALMSRFGISETQAEAILEL |
| RIVM_H_2011-01 | RLNYRLEKVLKRLHILEGLLVAFLNIDEVIEIIRNEDEPKPALMSRFGISETQAEAILEL |
| RIVM_H_2011-02 | RLNYRLEKVLKRLHILEGLLVAFLNIDEVIEIIRNEDEPKPALMSRFGISETQAEAILEL |
| RIVM_H_2011-03 | RLNYRLEKVLKRLHILEGLLVAFLNIDEVIEIIRNEDEPKPALMSRFGISETQAEAILEL |
| RIVM_H_2013-01 | RLNYRLEKVLKRLHILEGLLVAFLNIDEVIEIIRNEDEPKPALMSRFGISETQAEAILEL |
| RIVM_H_2013-02 | RLNYRLEKVLKRLHILEGLLVAFLNIDEVIEIIRNEDEPKPALMSRFGISETQAEAILEL |
| RIVM_H_2014-01 | RLNYRLEKVLKRLHILEGLLVAFLNIDEVIEIIRNEDEPKPALMSRFGISETQAEAILEL |
| RIVM_H_2014-02 | RLNYRLEKVLKRLHILEGLLVAFLNIDEVIEIIRNEDEPKPALMSRFGISETQAEAILEL |
| RIVM_H_2016-01 | RLNYRLEKVLKRLHILEGLLVAFLNIDEVIEIIRNEDEPKPALMSRFGISETQAEAILEL |
| RIVM_H_2016-02 | RLNYRLEKVLKRLHILEGLLVAFLNIDEVIEIIRNEDEPKPALMSRFGISETQAEAILEL |
| RIVM_H_2016-03 | RLNYRLEKVLKRLHILEGLLVAFLNIDEVIEIIRNEDEPKPALMSRFGISETQAEAILEL |
| RIVM_H_2016-04 | RLNYRLEKVLKRLHILEGLLVAFLNIDEVIEIIRNEDEPKPALMSRFGISETQAEAILEL |
| RIVM_H_2016-05 | RLNYRLEKVLKRLHILEGLLVAFLNIDEVIEIIRNEDEPKPALMSRFGISETQAEAILEL |
| RIVM_H_2016-06 | RLNYRLEKVLKRLHILEGLLVAFLNIDEVIEIIRNEDEPKPALMSRFGISETQAEAILEL |
| RIVM_H_2016-07 | RLNYRLEKVLKRLHILEGLLVAFLNIDEVIEIIRNEDEPKPALMSRFGISETQAEAILEL |
| RIVM_H_2016-08 | RLNYRLEKVLKRLHILEGLLVAFLNIDEVIEIIRNEDEPKPALMSRFGISETQAEAILEL |
| RIVM_H_2016-09 | RLNYRLEKVLKRLHILEGLLVAFLNIDEVIEIIRNEDEPKPALMSRFGISETQAEAILEL |
| RIVM_H_2016-10 | RLNYRLEKVLKRLHILEGLLVAFLNIDEVIEIIRNEDEPKPALMSRFGISETQAEAILEL |
| RIVM_H_2016-11 | RLNYRLEKVLKRLHILEGLLVAFLNIDEVIEIIRNEDEPKPALMSRFGISETQAEAILEL |
| RIVM_H_2016-12 | RLNYRLEKVLKRLHILEGLLVAFLNIDEVIEIIRNEDEPKPALMSRFGISETQAEAILEL |
| RIVM_H_2016-13 | RLNYRLEKVLKRLHILEGLLVAFLNIDEVIEIIRNEDEPKPALMSRFGISETQAEAILEL |
| RIVM_H_2016-14 | RLNYRLEKVLKRLHILEGLLVAFLNIDEVIEIIRNEDEPKPALMSRFGISETQAEAILEL |
| RIVM_H_2016-15 | RLNYRLEKVLKRLHILEGLLVAFLNIDEVIEIIRNEDEPKPALMSRFGISETQAEAILEL |
| RIVM_H_2017-01 | RLNYRLEKVLKRLHILEGLLVAFLNIDEVIEIIRNEDEPKPALMSRFGISETQAEAILEL |
| RIVM_H_2017-02 | RLNYRLEKVLKRLHILEGLLVAFLNIDEVIEIIRNEDEPKPALMSRFGISETQAEAILEL |
| RIVM_H_2017-03 | RLNYRLEKVLKRLHILEGLLVAFLNIDEVIEIIRNEDEPKPALMSRFGISETQAEAILEL |
| RIVM_H_2017-04 | RLNYRLEKVLKRLHILEGLLVAFLNIDEVIEIIRNEDEPKPALMSRFGISETQAEAILEL |
| RIVM_H_2017-05 | RLNYRLEKVLKRLHILEGLLVAFLNIDEVIEIIRNEDEPKPALMSRFGISETQAEAILEL |
| RIVM_H_2017-06 | RLNYRLEKVLKRLHILEGLLVAFLNIDEVIEIIRNEDEPKPALMSRFGISETQAEAILEL |
| RIVM_H_2017-07 | RLNYRLEKVLKRLHILEGLLVAFLNIDEVIEIIRNEDEPKPALMSRFGISETQAEAILEL |
| RIVM_H_2017-08 | RLNYRLEKVLKRLHILEGLLVAFLNIDEVIEIIRNEDEPKPALMSRFGISETQAEAILEL |
| RIVM_H_2017-09 | RLNYRLEKVLKRLHILEGLLVAFLNIDEVIEIIRNEDEPKPALMSRFGISETQAEAILEL |
| RIVM_H_2017-10 | RLNYRLEKVLKRLHILEGLLVAFLNIDEVIEIIRNEDEPKPALMSRFGISETQAEAILEL |
| RIVM_H_2017-11 | RLNYRLEKVLKRLHILEGLLVAFLNIDEVIEIIRNEDEPKPALMSRFGISETQAEAILEL |
| RIVM_H_2017-12 | RLNYRLEKVLKRLHILEGLLVAFLNIDEVIEIIRNEDEPKPALMSRFGISETQAEAILEL |
| RIVM_H_2017-13 | RLNYRLEKVLKRLHILEGLLVAFLNIDEVIEIIRNEDEPKPALMSRFGISETQAEAILEL |
| RIVM_H_2017-14 | RLNYRLEKVLKRLHILEGLLVAFLNIDEVIEIIRNEDEPKPALMSRFGISETQAEAILEL |
| RIVM_H_2017-15 | RLNYRLEKVLKRLHILEGLLVAFLNIDEVIEIIRNEDEPKPALMSRFGISETQAEAILEL |
| RIVM_H_2017-16 | RLNYRLEKVLKRLHILEGLLVAFLNIDEVIEIIRNEDEPKPALMSRFGISETQAEAILEL |
| RIVM_H_2017-17 | RLNYRLEKVLKRLHILEGLLVAFLNIDEVIEIIRNEDEPKPALMSRFGISETQAEAILEL |
| RIVM_H_2017-18 | RLNYRLEKVLKRLHILEGLLVAFLNIDEVIEIIRNEDEPKPALMSRFGISETQAEAILEL |
| RIVM_H_2017-19 | RLNYRLEKVLKRLHILEGLLVAFLNIDEVIEIIRNEDEPKPALMSRFGISETQAEAILEL |
| 15EP001483 | RLNYRLEKVLKRLHILEGLLVAFLNIDEVIEIIRNEDEPKPALMSRFGISETQAEAILEL |
| 17EP002363 | RLNYRLEKVLKRLHILEGLLVAFLNIDEVIEIIRNEDEPKPALMSRFGISETQAEAILEL |
| S_0812_17 | RLNYRLEKVLKRLHILEGLLVAFLNIDEVIEIIRNEDEPKPALMSRFGISETQAEAILEL |
| SRR1957844 | RLNYRLEKVLKRLHILEGLLVAFLNIDEVIEIIRNEDEPKPALMSRFGISETQAEAILEL |
| SRR1958654 | RLNYRLEKVLKRLHILEGLLVAFLNIDEVIEIIRNEDEPKPALMSRFGISETQAEAILEL |
| SRR1965077 | RLNYRLEKVLKRLHILEGLLVAFLNIDEVIEIIRNEDEPKPALMSRFGISETQAEAILEL |
| SRR1966369 | RLNYRLEKVLKRLHILEGLLVAFLNIDEVIEIIRNEDEPKPALMSRFGISETQAEAILEL |
| SRR1967117 | RLNYRLEKVLKRLHILEGLLVAFLNIDEVIEIIRNEDEPKPALMSRFGISETQAEAILEL |
| SRR1967922 | RLNYRLEKVLKRLHILEGLLVAFLNIDEVIEIIRNEDEPKPALMSRFGISETQAEAILEL |
| SRR8704720 | RLNYRLEKVLKRLHILEGLLVAFLNIDEVIEIIRNEDEPKPALMSRFGISETQAEAILEL |
| SRR7216071 | RLNYRLEKVLKRLHILEGLLVAFLNIDEVIEIIRNEDEPKPALMSRFGISETQAEAILEL |
| SRR7349175 | RLNYRLEKVLKRLHILEGLLVAFLNIDEVIEIIRNEDEPKPALMSRFGISETQAEAILEL |
| SRR7523148 | RLNYRLEKVLKRLHILEGLLVAFLNIDEVIEIIRNEDEPKPALMSRFGISETQAEAILEL |
| SRR7523854 | RLNYRLEKVLKRLHILEGLLVAFLNIDEVIEIIRNEDEPKPALMSRFGISETQAEAILEL |
| 313865 | RLNYRLEKVLKRLHILEGLLVAFLNIDEVIEIIRNEDEPKPALMSRFGISETQAEAILEL |
| SRR7277793 | RLNYRLEKVLKRLHILEGLLVAFLNIDEVIEIIRNEDEPKPALMSRFGISETQAEAILEL |
| SRR7343877 | RLNYRLEKVLKRLHILEGLLVAFLNIDEVIEIIRNEDEPKPALMSRFGISETQAEAILEL |
| SRR7351477 | RLNYRLEKVLKRLHILEGLLVAFLNIDEVIEIIRNEDEPKPALMSRFGISETQAEAILEL |
| SRR5583183 | RLNYRLEKVLKRLHILEGLLVAFLNIDEVIEIIRNEDEPKPALMSRFGISETQAEAILEL |
| SRR5585240 | RLNYRLEKVLKRLHILEGLLVAFLNIDEVIEIIRNEDEPKPALMSRFGISETQAEAILEL |
| SRR7284317 | RLNYRLEKVLKRLHILEGLLVAFLNIDEVIEIIRNEDEPKPALMSRFGISETQAEAILEL |
| SRR7299161 | RLNYRLEKVLKRLHILEGLLVAFLNIDEVIEIIRNEDEPKPALMSRFGISETQAEAILEL |
| SRR7401730 | RLNYRLEKVLKRLHILEGLLVAFLNIDEVIEIIRNEDEPKPALMSRFGISETQAEAILEL |
| SRR7469092 | RLNYRLEKVLKRLHILEGLLVAFLNIDEVIEIIRNEDEPKPALMSRFGISETQAEAILEL |
| SRR7879556 | RLNYRLEKVLKRLHILEGLLVAFLNIDEVIEIIRNEDEPKPALMSRFGISETQAEAILEL |
| SRR8526100 | RLNYRLEKVLKRLHILEGLLVAFLNIDEVIEIIRNEDEPKPALMSRFGISETQAEAILEL |
| SRR8553991 | RLNYRLEKVLKRLHILEGLLVAFLNIDEVIEIIRNEDEPKPALMSRFGISETQAEAILEL |
| SRR7842487 | RLNYRLEKVLKRLHILEGLLVAFLNIDEVIEIIRNEDEPKPALMSRFGISETQAEAILEL |
| SRR8054524 | RLNYRLEKVLKRLHILEGLLVAFLNIDEVIEIIRNEDEPKPALMSRFGISETQAEAILEL |
| SRR8054525 | RLNYRLEKVLKRLHILEGLLVAFLNIDEVIEIIRNEDEPKPALMSRFGISETQAEAILEL |
| SRR8524733 | RLNYRLEKVLKRLHILEGLLVAFLNIDEVIEIIRNEDEPKPALMSRFGISETQAEAILEL |
| SRR4093291 | RLNYRLEKVLKRLHILEGLLVAFLNIDEVIEIIRNEDEPKPALMSRFGISETQAEAILEL |
| SRR4245549 | RLNYRLEKVLKRLHILEGLLVAFLNIDEVIEIIRNEDEPKPALMSRFGISETQAEAILEL |
| SRR3057154 | RLNYRLEKVLKRLHILEGLLVAFLNIDEVIEIIRNEDEPKPALMSRFGISETQAEAILEL |
| SRR1726150 | RLNYRLEKVLKRLHILEGLLVAFLNIDEVIEIIRNEDEPKPALMSRFGISETQAEAILEL |
| SRR1996141 | RLNYRLEKVLKRLHILEGLLVAFLNIDEVIEIIRNEDEPKPALMSRFGISETQAEAILEL |
| SRR1107842 | RLNYRLEKVLKRLHILEGLLVAFLNIDEVIEIIRNEDEPKPALMSRFGISETQAEAILEL |
| SRR1157587 | RLNYRLEKVLKRLHILEGLLVAFLNIDEVIEIIRNEDEPKPALMSRFGISETQAEAILEL |
| SRR3027706 | RLNYRLEKVLKRLHILEGLLVAFLNIDEVIEIIRNEDEPKPALMSRFGISETQAEAILEL |
| SRR3027707 | RLNYRLEKVLKRLHILEGLLVAFLNIDEVIEIIRNEDEPKPALMSRFGISETQAEAILEL |
| SRR3027708 | RLNYRLEKVLKRLHILEGLLVAFLNIDEVIEIIRNEDEPKPALMSRFGISETQAEAILEL |
| SRR3027710 | RLNYRLEKVLKRLHILEGLLVAFLNIDEVIEIIRNEDEPKPALMSRFGISETQAEAILEL |
| SRR3027711 | RLNYRLEKVLKRLHILEGLLVAFLNIDEVIEIIRNEDEPKPALMSRFGISETQAEAILEL |
| SRR3027716 | RLNYRLEKVLKRLHILEGLLVAFLNIDEVIEIIRNEDEPKPALMSRFGISETQAEAILEL |
| SRR3027717 | RLNYRLEKVLKRLHILEGLLVAFLNIDEVIEIIRNEDEPKPALMSRFGISETQAEAILEL |
| SRR3027719 | RLNYRLEKVLKRLHILEGLLVAFLNIDEVIEIIRNEDEPKPALMSRFGISETQAEAILEL |
| SRR3027721 | RLNYRLEKVLKRLHILEGLLVAFLNIDEVIEIIRNEDEPKPALMSRFGISETQAEAILEL |
| SRR3027723 | RLNYRLEKVLKRLHILEGLLVAFLNIDEVIEIIRNEDEPKPALMSRFGISETQAEAILEL |
| SRR3115978 | RLNYRLEKVLKRLHILEGLLVAFLNIDEVIEIIRNEDEPKPALMSRFGISETQAEAILEL |
| SRR2534093 | RLNYRLEKVLKRLHILEGLLVAFLNIDEVIEIIRNEDEPKPALMSRFGISETQAEAILEL |
| SRR2534094 | RLNYRLEKVLKRLHILEGLLVAFLNIDEVIEIIRNEDEPKPALMSRFGISETQAEAILEL |
| SRR2534095 | RLNYRLEKVLKRLHILEGLLVAFLNIDEVIEIIRNEDEPKPALMSRFGISETQAEAILEL |
| SRR2534108 | RLNYRLEKVLKRLHILEGLLVAFLNIDEVIEIIRNEDEPKPALMSRFGISETQAEAILEL |
| SRR1106464 | RLNYRLEKVLKRLHILEGLLVAFLNIDEVIEIIRNEDEPKPALMSRFGISETQAEAILEL |
| SRR1106463 | RLNYRLEKVLKRLHILEGLLVAFLNIDEVIEIIRNEDEPKPALMSRFGISETQAEAILEL |
| SRR6949610 | RLNYRLEKVLKRLHILEGLLVAFLNIDEVIEIIRNEDEPKPALMSRFGISETQAEAILEL |
| SRR6950452 | RLNYRLEKVLKRLHILEGLLVAFLNIDEVIEIIRNEDEPKPALMSRFGISETQAEAILEL |
| ERR2019831 | RLNYRLEKVLKRLHILEGLLVAFLNIDEVIEIIRNEDEPKPALMSRFGISETQAEAILEL |
| SRR2085693 | RLNYRLEKVLKRLHILEGLLVAFLNIDEVIEIIRNEDEPKPALMSRFGISETQAEAILEL |
| SRR2086898 | RLNYRLEKVLKRLHILEGLLVAFLNIDEVIEIIRNEDEPKPALMSRFGISETQAEAILEL |
| SRR2175312 | RLNYRLEKVLKRLHILEGLLVAFLNIDEVIEIIRNEDEPKPALMSRFGISETQAEAILEL |
| SRR2175360 | RLNYRLEKVLKRLHILEGLLVAFLNIDEVIEIIRNEDEPKPALMSRFGISETQAEAILEL |
| SRR5231997 | RLNYRLEKVLKRLHILEGLLVAFLNIDEVIEIIRNEDEPKPALMSRFGISETQAEAILEL |
| SRR5232003 | RLNYRLEKVLKRLHILEGLLVAFLNIDEVIEIIRNEDEPKPALMSRFGISETQAEAILEL |
| SRR5232015 | RLNYRLEKVLKRLHILEGLLVAFLNIDEVIEIIRNEDEPKPALMSRFGISETQAEAILEL |
| SRR949434 | RLNYRLEKVLKRLHILEGLLVAFLNIDEVIEIIRNEDEPKPALMSRFGISETQAEAILEL |
| SRR3216575 | RLNYRLEKVLKRLHILEGLLVAFLNIDEVIEIIRNEDEPKPALMSRFGISETQAEAILEL |
| SRR5205342 | RLNYRLEKVLKRLHILEGLLVAFLNIDEVIEIIRNEDEPKPALMSRFGISETQAEAILEL |
| SRR1501669 | RLNYRLEKVLKRLHILEGLLVAFLNIDEVIEIIRNEDEPKPALMSRFGISETQAEAILEL |
| SRR5209740 | RLNYRLEKVLKRLHILEGLLVAFLNIDEVIEIIRNEDEPKPALMSRFGISETQAEAILEL |
| SRR3240355 | RLNYRLEKVLKRLHILEGLLVAFLNIDEVIEIIRNEDEPKPALMSRFGISETQAEAILEL |
| SRR3392777 | RLNYRLEKVLKRLHILEGLLVAFLNIDEVIEIIRNEDEPKPALMSRFGISETQAEAILEL |
| SRR3593671 | RLNYRLEKVLKRLHILEGLLVAFLNIDEVIEIIRNEDEPKPALMSRFGISETQAEAILEL |
| SRR5413290 | RLNYRLEKVLKRLHILEGLLVAFLNIDEVIEIIRNEDEPKPALMSRFGISETQAEAILEL |
| SRR5590269 | RLNYRLEKVLKRLHILEGLLVAFLNIDEVIEIIRNEDEPKPALMSRFGISETQAEAILEL |
| SRR5812103 | RLNYRLEKVLKRLHILEGLLVAFLNIDEVIEIIRNEDEPKPALMSRFGISETQAEAILEL |
| SRR2830941 | RLNYRLEKVLKRLHILEGLLVAFLNIDEVIEIIRNEDEPKPALMSRFGISETQAEAILEL |
| SRR2830966 | RLNYRLEKVLKRLHILEGLLVAFLNIDEVIEIIRNEDEPKPALMSRFGISETQAEAILEL |
| SRR3137270 | RLNYRLEKVLKRLHILEGLLVAFLNIDEVIEIIRNEDEPKPALMSRFGISETQAEAILEL |
| SRR3137271 | RLNYRLEKVLKRLHILEGLLVAFLNIDEVIEIIRNEDEPKPALMSRFGISETQAEAILEL |
| ERR526807 | RLNYRLEKVLKRLHILEGLLVAFLNIDEVIEIIRNEDEPKPALMSRFGISETQAEAILEL |
| ERR2197922 | RLNYRLEKVLKRLHILEGLLVAFLNIDEVIEIIRNEDEPKPALMSRFGISETQAEAILEL |
| ERR2197923 | RLNYRLEKVLKRLHILEGLLVAFLNIDEVIEIIRNEDEPKPALMSRFGISETQAEAILEL |
| ERR2197924 | RLNYRLEKVLKRLHILEGLLVAFLNIDEVIEIIRNEDEPKPALMSRFGISETQAEAILEL |
| ERR2197925 | RLNYRLEKVLKRLHILEGLLVAFLNIDEVIEIIRNEDEPKPALMSRFGISETQAEAILEL |
| ERR2197927 | RLNYRLEKVLKRLHILEGLLVAFLNIDEVIEIIRNEDEPKPALMSRFGISETQAEAILEL |
| ERR2197929 | RLNYRLEKVLKRLHILEGLLVAFLNIDEVIEIIRNEDEPKPALMSRFGISETQAEAILEL |
| SRR1648149 | RLNYRLEKVLKRLHILEGLLVAFLNIDEVIEIIRNEDEPKPALMSRFGISETQAEAILEL |
| SRR1048299 | RLNYRLEKVLKRLHILEGLLVAFLNIDEVIEIIRNEDEPKPALMSRFGISETQAEAILEL |
| SRR1300677 | RLNYRLEKVLKRLHILEGLLVAFLNIDEVIEIIRNEDEPKPALMSRFGISETQAEAILEL |
| SRR1288356 | RLNYRLEKVLKRLHILEGLLVAFLNIDEVIEIIRNEDEPKPALMSRFGISETQAEAILEL |
| SRR7426190 | RLNYRLEKVLKRLHILEGLLVAFLNIDEVIEIIRNEDEPKPALMSRFGISETQAEAILEL |
| SRR7426192 | RLNYRLEKVLKRLHILEGLLVAFLNIDEVIEIIRNEDEPKPALMSRFGISETQAEAILEL |
| SRR7426193 | RLNYRLEKVLKRLHILEGLLVAFLNIDEVIEIIRNEDEPKPALMSRFGISETQAEAILEL |
| SRR7441832 | RLNYRLEKVLKRLHILEGLLVAFLNIDEVIEIIRNEDEPKPALMSRFGISETQAEAILEL |
| SRR7426179 | RLNYRLEKVLKRLHILEGLLVAFLNIDEVIEIIRNEDEPKPALMSRFGISETQAEAILEL |
| SRR7439238 | RLNYRLEKVLKRLHILEGLLVAFLNIDEVIEIIRNEDEPKPALMSRFGISETQAEAILEL |
| SRR7439244 | RLNYRLEKVLKRLHILEGLLVAFLNIDEVIEIIRNEDEPKPALMSRFGISETQAEAILEL |
| SRR7439259 | RLNYRLEKVLKRLHILEGLLVAFLNIDEVIEIIRNEDEPKPALMSRFGISETQAEAILEL |
| SRR7439260 | RLNYRLEKVLKRLHILEGLLVAFLNIDEVIEIIRNEDEPKPALMSRFGISETQAEAILEL |
| SRR7441786 | RLNYRLEKVLKRLHILEGLLVAFLNIDEVIEIIRNEDEPKPALMSRFGISETQAEAILEL |
| SRR7441797 | RLNYRLEKVLKRLHILEGLLVAFLNIDEVIEIIRNEDEPKPALMSRFGISETQAEAILEL |
| ERR1759093 | RLNYRLEKVLKRLHILEGLLVAFLNIDEVIEIIRNEDEPKPALMSRFGISETQAEAILEL |
| ERR2580275 | RLNYRLEKVLKRLHILEGLLVAFLNIDEVIEIIRNEDEPKPALMSRFGISETQAEAILEL |
| ERR1759204 | RLNYRLEKVLKRLHILEGLLVAFLNIDEVIEIIRNEDEPKPALMSRFGISETQAEAILEL |
| SRR1300699 | RLNYRLEKVLKRLHILEGLLVAFLNIDEVIEIIRNEDEPKPALMSRFGISETQAEAILEL |
| S_0825_17 | RLNYRLEKVLKRLHILEGLLVAFLNIDEVIEIIRNEDEPKPALMSRFGISETQAEAILEL |
| SRR1958215 | RLNYRLEKVLKRLHILEGLLVAFLNIDEVIEIIRNEDEPKPALMSRFGISETQAEAILEL |
| SRR1958540 | RLNYRLEKVLKRLHILEGLLVAFLNIDEVIEIIRNEDEPKPALMSRFGISETQAEAILEL |
| SRR1958636 | RLNYRLEKVLKRLHILEGLLVAFLNIDEVIEIIRNEDEPKPALMSRFGISETQAEAILEL |
| SRR1959422 | RLNYRLEKVLKRLHILEGLLVAFLNIDEVIEIIRNEDEPKPALMSRFGISETQAEAILEL |
| SRR1959427 | RLNYRLEKVLKRLHILEGLLVAFLNIDEVIEIIRNEDEPKPALMSRFGISETQAEAILEL |
| SRR1960226 | RLNYRLEKVLKRLHILEGLLVAFLNIDEVIEIIRNEDEPKPALMSRFGISETQAEAILEL |
| SRR1963498 | RLNYRLEKVLKRLHILEGLLVAFLNIDEVIEIIRNEDEPKPALMSRFGISETQAEAILEL |
| SRR1965947 | RLNYRLEKVLKRLHILEGLLVAFLNIDEVIEIIRNEDEPKPALMSRFGISETQAEAILEL |
| SRR1966125 | RLNYRLEKVLKRLHILEGLLVAFLNIDEVIEIIRNEDEPKPALMSRFGISETQAEAILEL |
| SRR1966330 | RLNYRLEKVLKRLHILEGLLVAFLNIDEVIEIIRNEDEPKPALMSRFGISETQAEAILEL |
| SRR1966565 | RLNYRLEKVLKRLHILEGLLVAFLNIDEVIEIIRNEDEPKPALMSRFGISETQAEAILEL |
| SRR1966864 | RLNYRLEKVLKRLHILEGLLVAFLNIDEVIEIIRNEDEPKPALMSRFGISETQAEAILEL |
| SRR1966989 | RLNYRLEKVLKRLHILEGLLVAFLNIDEVIEIIRNEDEPKPALMSRFGISETQAEAILEL |
| SRR1967688 | RLNYRLEKVLKRLHILEGLLVAFLNIDEVIEIIRNEDEPKPALMSRFGISETQAEAILEL |
| SRR1967733 | RLNYRLEKVLKRLHILEGLLVAFLNIDEVIEIIRNEDEPKPALMSRFGISETQAEAILEL |
| SRR1967746 | RLNYRLEKVLKRLHILEGLLVAFLNIDEVIEIIRNEDEPKPALMSRFGISETQAEAILEL |
| SRR1968341 | RLNYRLEKVLKRLHILEGLLVAFLNIDEVIEIIRNEDEPKPALMSRFGISETQAEAILEL |
| SRR1968456 | RLNYRLEKVLKRLHILEGLLVAFLNIDEVIEIIRNEDEPKPALMSRFGISETQAEAILEL |
| SRR1968465 | RLNYRLEKVLKRLHILEGLLVAFLNIDEVIEIIRNEDEPKPALMSRFGISETQAEAILEL |
| SRR1968761 | RLNYRLEKVLKRLHILEGLLVAFLNIDEVIEIIRNEDEPKPALMSRFGISETQAEAILEL |
| SRR1969047 | RLNYRLEKVLKRLHILEGLLVAFLNIDEVIEIIRNEDEPKPALMSRFGISETQAEAILEL |
| SRR1969255 | RLNYRLEKVLKRLHILEGLLVAFLNIDEVIEIIRNEDEPKPALMSRFGISETQAEAILEL |
| SRR1969412 | RLNYRLEKVLKRLHILEGLLVAFLNIDEVIEIIRNEDEPKPALMSRFGISETQAEAILEL |
| SRR1969524 | RLNYRLEKVLKRLHILEGLLVAFLNIDEVIEIIRNEDEPKPALMSRFGISETQAEAILEL |
| SRR1969584 | RLNYRLEKVLKRLHILEGLLVAFLNIDEVIEIIRNEDEPKPALMSRFGISETQAEAILEL |
| SRR1969648 | RLNYRLEKVLKRLHILEGLLVAFLNIDEVIEIIRNEDEPKPALMSRFGISETQAEAILEL |
| SRR1969804 | RLNYRLEKVLKRLHILEGLLVAFLNIDEVIEIIRNEDEPKPALMSRFGISETQAEAILEL |
| SRR1970221 | RLNYRLEKVLKRLHILEGLLVAFLNIDEVIEIIRNEDEPKPALMSRFGISETQAEAILEL |
| SRR1970268 | RLNYRLEKVLKRLHILEGLLVAFLNIDEVIEIIRNEDEPKPALMSRFGISETQAEAILEL |
| SRR1965862 | RLNYRLEKVLKRLHILEGLLVAFLNIDEVIEIIRNEDEPKPALMSRFGISETQAEAILEL |
| SRR1967363 | RLNYRLEKVLKRLHILEGLLVAFLNIDEVIEIIRNEDEPKPALMSRFGISETQAEAILEL |
| SRR1968276 | RLNYRLEKVLKRLHILEGLLVAFLNIDEVIEIIRNEDEPKPALMSRFGISETQAEAILEL |
| SRR1968967 | RLNYRLEKVLKRLHILEGLLVAFLNIDEVIEIIRNEDEPKPALMSRFGISETQAEAILEL |
| SRR3321531 | RLNYRLEKVLKRLHILEGLLVAFLNIDEVIEIIRNEDEPKPALMSRFGISETQAEAILEL |
| SRR3321883 | RLNYRLEKVLKRLHILEGLLVAFLNIDEVIEIIRNEDEPKPALMSRFGISETQAEAILEL |
| SRR3322413 | RLNYRLEKVLKRLHILEGLLVAFLNIDEVIEIIRNEDEPKPALMSRFGISETQAEAILEL |
| SRR3323012 | RLNYRLEKVLKRLHILEGLLVAFLNIDEVIEIIRNEDEPKPALMSRFGISETQAEAILEL |
| SRR5194289 | RLNYRLEKVLKRLHILEGLLVAFLNIDEVIEIIRNEDEPKPALMSRFGISETQAEAILEL |
| SRR7163798 | RLNYRLEKVLKRLHILEGLLVAFLNIDEVIEIIRNEDEPKPALMSRFGISETQAEAILEL |
| SRR7172610 | RLNYRLEKVLKRLHILEGLLVAFLNIDEVIEIIRNEDEPKPALMSRFGISETQAEAILEL |
| SRR7204568 | RLNYRLEKVLKRLHILEGLLVAFLNIDEVIEIIRNEDEPKPALMSRFGISETQAEAILEL |
| SRR7223230 | RLNYRLEKVLKRLHILEGLLVAFLNIDEVIEIIRNEDEPKPALMSRFGISETQAEAILEL |
| SRR7230675 | RLNYRLEKVLKRLHILEGLLVAFLNIDEVIEIIRNEDEPKPALMSRFGISETQAEAILEL |
| SRR7278056 | RLNYRLEKVLKRLHILEGLLVAFLNIDEVIEIIRNEDEPKPALMSRFGISETQAEAILEL |
| SRR7278086 | RLNYRLEKVLKRLHILEGLLVAFLNIDEVIEIIRNEDEPKPALMSRFGISETQAEAILEL |
| SRR7285841 | RLNYRLEKVLKRLHILEGLLVAFLNIDEVIEIIRNEDEPKPALMSRFGISETQAEAILEL |
| SRR7292625 | RLNYRLEKVLKRLHILEGLLVAFLNIDEVIEIIRNEDEPKPALMSRFGISETQAEAILEL |
| SRR7292665 | RLNYRLEKVLKRLHILEGLLVAFLNIDEVIEIIRNEDEPKPALMSRFGISETQAEAILEL |
| SRR7297965 | RLNYRLEKVLKRLHILEGLLVAFLNIDEVIEIIRNEDEPKPALMSRFGISETQAEAILEL |
| SRR7350726 | RLNYRLEKVLKRLHILEGLLVAFLNIDEVIEIIRNEDEPKPALMSRFGISETQAEAILEL |
| SRR7410328 | RLNYRLEKVLKRLHILEGLLVAFLNIDEVIEIIRNEDEPKPALMSRFGISETQAEAILEL |
| SRR7474665 | RLNYRLEKVLKRLHILEGLLVAFLNIDEVIEIIRNEDEPKPALMSRFGISETQAEAILEL |
| SRR7523184 | RLNYRLEKVLKRLHILEGLLVAFLNIDEVIEIIRNEDEPKPALMSRFGISETQAEAILEL |
| SRR7187264 | RLNYRLEKVLKRLHILEGLLVAFLNIDEVIEIIRNEDEPKPALMSRFGISETQAEAILEL |
| SRR7204445 | RLNYRLEKVLKRLHILEGLLVAFLNIDEVIEIIRNEDEPKPALMSRFGISETQAEAILEL |
| SRR7285641 | RLNYRLEKVLKRLHILEGLLVAFLNIDEVIEIIRNEDEPKPALMSRFGISETQAEAILEL |
| SRR7286695 | RLNYRLEKVLKRLHILEGLLVAFLNIDEVIEIIRNEDEPKPALMSRFGISETQAEAILEL |
| SRR7286705 | RLNYRLEKVLKRLHILEGLLVAFLNIDEVIEIIRNEDEPKPALMSRFGISETQAEAILEL |
| SRR7292931 | RLNYRLEKVLKRLHILEGLLVAFLNIDEVIEIIRNEDEPKPALMSRFGISETQAEAILEL |
| SRR7310349 | RLNYRLEKVLKRLHILEGLLVAFLNIDEVIEIIRNEDEPKPALMSRFGISETQAEAILEL |
| SRR7351616 | RLNYRLEKVLKRLHILEGLLVAFLNIDEVIEIIRNEDEPKPALMSRFGISETQAEAILEL |
| SRR7414818 | RLNYRLEKVLKRLHILEGLLVAFLNIDEVIEIIRNEDEPKPALMSRFGISETQAEAILEL |
| SRR7426480 | RLNYRLEKVLKRLHILEGLLVAFLNIDEVIEIIRNEDEPKPALMSRFGISETQAEAILEL |
| SRR5584105 | RLNYRLEKVLKRLHILEGLLVAFLNIDEVIEIIRNEDEPKPALMSRFGISETQAEAILEL |
| SRR5584565 | RLNYRLEKVLKRLHILEGLLVAFLNIDEVIEIIRNEDEPKPALMSRFGISETQAEAILEL |
| SRR5584614 | RLNYRLEKVLKRLHILEGLLVAFLNIDEVIEIIRNEDEPKPALMSRFGISETQAEAILEL |
| SRR5631543 | RLNYRLEKVLKRLHILEGLLVAFLNIDEVIEIIRNEDEPKPALMSRFGISETQAEAILEL |
| SRR5631553 | RLNYRLEKVLKRLHILEGLLVAFLNIDEVIEIIRNEDEPKPALMSRFGISETQAEAILEL |
| SRR7123196 | RLNYRLEKVLKRLHILEGLLVAFLNIDEVIEIIRNEDEPKPALMSRFGISETQAEAILEL |
| SRR7163819 | RLNYRLEKVLKRLHILEGLLVAFLNIDEVIEIIRNEDEPKPALMSRFGISETQAEAILEL |
| SRR7163920 | RLNYRLEKVLKRLHILEGLLVAFLNIDEVIEIIRNEDEPKPALMSRFGISETQAEAILEL |
| SRR7209528 | RLNYRLEKVLKRLHILEGLLVAFLNIDEVIEIIRNEDEPKPALMSRFGISETQAEAILEL |
| SRR7249868 | RLNYRLEKVLKRLHILEGLLVAFLNIDEVIEIIRNEDEPKPALMSRFGISETQAEAILEL |
| SRR7278088 | RLNYRLEKVLKRLHILEGLLVAFLNIDEVIEIIRNEDEPKPALMSRFGISETQAEAILEL |
| SRR7285788 | RLNYRLEKVLKRLHILEGLLVAFLNIDEVIEIIRNEDEPKPALMSRFGISETQAEAILEL |
| SRR7286789 | RLNYRLEKVLKRLHILEGLLVAFLNIDEVIEIIRNEDEPKPALMSRFGISETQAEAILEL |
| SRR7286886 | RLNYRLEKVLKRLHILEGLLVAFLNIDEVIEIIRNEDEPKPALMSRFGISETQAEAILEL |
| SRR7310632 | RLNYRLEKVLKRLHILEGLLVAFLNIDEVIEIIRNEDEPKPALMSRFGISETQAEAILEL |
| SRR7350631 | RLNYRLEKVLKRLHILEGLLVAFLNIDEVIEIIRNEDEPKPALMSRFGISETQAEAILEL |
| SRR7458741 | RLNYRLEKVLKRLHILEGLLVAFLNIDEVIEIIRNEDEPKPALMSRFGISETQAEAILEL |
| SRR7480280 | RLNYRLEKVLKRLHILEGLLVAFLNIDEVIEIIRNEDEPKPALMSRFGISETQAEAILEL |
| SRR7523660 | RLNYRLEKVLKRLHILEGLLVAFLNIDEVIEIIRNEDEPKPALMSRFGISETQAEAILEL |
| SRR7523775 | RLNYRLEKVLKRLHILEGLLVAFLNIDEVIEIIRNEDEPKPALMSRFGISETQAEAILEL |
| SRR7251101 | RLNYRLEKVLKRLHILEGLLVAFLNIDEVIEIIRNEDEPKPALMSRFGISETQAEAILEL |
| SRR7284299 | RLNYRLEKVLKRLHILEGLLVAFLNIDEVIEIIRNEDEPKPALMSRFGISETQAEAILEL |
| SRR7285738 | RLNYRLEKVLKRLHILEGLLVAFLNIDEVIEIIRNEDEPKPALMSRFGISETQAEAILEL |
| SRR7310640 | RLNYRLEKVLKRLHILEGLLVAFLNIDEVIEIIRNEDEPKPALMSRFGISETQAEAILEL |
| SRR7349159 | RLNYRLEKVLKRLHILEGLLVAFLNIDEVIEIIRNEDEPKPALMSRFGISETQAEAILEL |
| SRR7474873 | RLNYRLEKVLKRLHILEGLLVAFLNIDEVIEIIRNEDEPKPALMSRFGISETQAEAILEL |
| SRR7495689 | RLNYRLEKVLKRLHILEGLLVAFLNIDEVIEIIRNEDEPKPALMSRFGISETQAEAILEL |
| SRR7495752 | RLNYRLEKVLKRLHILEGLLVAFLNIDEVIEIIRNEDEPKPALMSRFGISETQAEAILEL |
| ------------------------------------------------------------------------------ | |
| S16BD08730 | KLRHLAKLEEMKIRGEQDELEKERDQLQGILASERKMNTLLKKELQADSDAYGDDRRSPL |
| S18BD00684 | KLRHLAKLEEMKIRGEQDELEKERDQLQGILASERKMNTLLKKELQADSDAYGDDRRSPL |
| S18BD03994 | KLRHLAKLEEMKIRGEQDELEKERDQLQGILASERKMNTLLKKELQADSDAYGDDRRSPL |
| S18BD05011 | KLRHLAKLEEMKIRGEQDELEKERDQLQGILASERKMNTLLKKELQADSDAYGDDRRSPL |
| RKI_16-03723 | KLRHLAKLEEMKIRGEQDELEKERDQLQGILASERKMNTLLKKELQADSDAYGDDRRSPL |
| RKI_16-04315 | KLRHLAKLEEMKIRGEQDELEKERDQLQGILASERKMNTLLKKELQADSDAYGDDRRSPL |
| RKI_17-02304 | KLRHLAKLEEMKIRGEQDELEKERDQLQGILASERKMNTLLKKELQADSDAYGDDRRSPL |
| RKI_17-02411 | KLRHLAKLEEMKIRGEQDELEKERDQLQGILASERKMNTLLKKELQADSDAYGDDRRSPL |
| RKI_17-02757 | KLRHLAKLEEMKIRGEQDELEKERDQLQGILASERKMNTLLKKELQADSDAYGDDRRSPL |
| RKI_17-04797 | KLRHLAKLEEMKIRGEQDELEKERDQLQGILASERKMNTLLKKELQADSDAYGDDRRSPL |
| RKI_17-06869 | KLRHLAKLEEMKIRGEQDELEKERDQLQGILASERKMNTLLKKELQADSDAYGDDRRSPL |
| ERR2580277 | KLRHLAKLEEMKIRGEQDELEKERDQLQGILASERKMNTLLKKELQADSDAYGDDRRSPL |
| ERR2580276 | KLRHLAKLEEMKIRGEQDELEKERDQLQGILASERKMNTLLKKELQADSDAYGDDRRSPL |
| ERR2580273 | KLRHLAKLEEMKIRGEQDELEKERDQLQGILASERKMNTLLKKELQADSDAYGDDRRSPL |
| ERR2580274 | KLRHLAKLEEMKIRGEQDELEKERDQLQGILASERKMNTLLKKELQADSDAYGDDRRSPL |
| ERR2173656 | KLRHLAKLEEMKIRGEQDELEKERDQLQGILASERKMNTLLKKELQADSDAYGDDRRSPL |
| 17041676 | KLRHLAKLEEMKIRGEQDELEKERDQLQGILASERKMNTLLKKELQADSDAYGDDRRSPL |
| MT16-000061 | KLRHLAKLEEMKIRGEQDELEKERDQLQGILASERKMNTLLKKELQADSDAYGDDRRSPL |
| MT16-019416 | KLRHLAKLEEMKIRGEQDELEKERDQLQGILASERKMNTLLKKELQADSDAYGDDRRSPL |
| MT16-027865 | KLRHLAKLEEMKIRGEQDELEKERDQLQGILASERKMNTLLKKELQADSDAYGDDRRSPL |
| MT16-031693 | KLRHLAKLEEMKIRGEQDELEKERDQLQGILASERKMNTLLKKELQADSDAYGDDRRSPL |
| MT16-040253 | KLRHLAKLEEMKIRGEQDELEKERDQLQGILASERKMNTLLKKELQADSDAYGDDRRSPL |
| MT16-045379 | KLRHLAKLEEMKIRGEQDELEKERDQLQGILASERKMNTLLKKELQADSDAYGDDRRSPL |
| MT16-442728 | KLRHLAKLEEMKIRGEQDELEKERDQLQGILASERKMNTLLKKELQADSDAYGDDRRSPL |
| MT16-462857 | KLRHLAKLEEMKIRGEQDELEKERDQLQGILASERKMNTLLKKELQADSDAYGDDRRSPL |
| MT16-480196 | KLRHLAKLEEMKIRGEQDELEKERDQLQGILASERKMNTLLKKELQADSDAYGDDRRSPL |
| MT16-861555 | KLRHLAKLEEMKIRGEQDELEKERDQLQGILASERKMNTLLKKELQADSDAYGDDRRSPL |
| MT17-076833 | KLRHLAKLEEMKIRGEQDELEKERDQLQGILASERKMNTLLKKELQADSDAYGDDRRSPL |
| MT17-110677 | KLRHLAKLEEMKIRGEQDELEKERDQLQGILASERKMNTLLKKELQADSDAYGDDRRSPL |
| MT17-131730 | KLRHLAKLEEMKIRGEQDELEKERDQLQGILASERKMNTLLKKELQADSDAYGDDRRSPL |
| MT17-140890 | KLRHLAKLEEMKIRGEQDELEKERDQLQGILASERKMNTLLKKELQADSDAYGDDRRSPL |
| MT17-141840 | KLRHLAKLEEMKIRGEQDELEKERDQLQGILASERKMNTLLKKELQADSDAYGDDRRSPL |
| MT17-152488 | KLRHLAKLEEMKIRGEQDELEKERDQLQGILASERKMNTLLKKELQADSDAYGDDRRSPL |
| MT17-157311 | KLRHLAKLEEMKIRGEQDELEKERDQLQGILASERKMNTLLKKELQADSDAYGDDRRSPL |
| MT17-161645 | KLRHLAKLEEMKIRGEQDELEKERDQLQGILASERKMNTLLKKELQADSDAYGDDRRSPL |
| MT17-167951 | KLRHLAKLEEMKIRGEQDELEKERDQLQGILASERKMNTLLKKELQADSDAYGDDRRSPL |
| MT18-217732 | KLRHLAKLEEMKIRGEQDELEKERDQLQGILASERKMNTLLKKELQADSDAYGDDRRSPL |
| MT18-252580 | KLRHLAKLEEMKIRGEQDELEKERDQLQGILASERKMNTLLKKELQADSDAYGDDRRSPL |
| RIVM_H_2009-01 | KLRHLAKLEEMKIRGEQDELEKERDQLQGILASERKMNTLLKKELQADSDAYGDDRRSPL |
| RIVM_H_2010-01 | KLRHLAKLEEMKIRGEQDELEKERDQLQGILASERKMNTLLKKELQADSDAYGDDRRSPL |
| RIVM_H_2010-02 | KLRHLAKLEEMKIRGEQDELEKERDQLQGILASERKMNTLLKKELQADSDAYGDDRRSPL |
| RIVM_H_2011-01 | KLRHLAKLEEMKIRGEQDELEKERDQLQGILASERKMNTLLKKELQADSDAYGDDRRSPL |
| RIVM_H_2011-02 | KLRHLAKLEEMKIRGEQDELEKERDQLQGILASERKMNTLLKKELQADSDAYGDDRRSPL |
| RIVM_H_2011-03 | KLRHLAKLEEMKIRGEQDELEKERDQLQGILASERKMNTLLKKELQADSDAYGDDRRSPL |
| RIVM_H_2013-01 | KLRHLAKLEEMKIRGEQDELEKERDQLQGILASERKMNTLLKKELQADSDAYGDDRRSPL |
| RIVM_H_2013-02 | KLRHLAKLEEMKIRGEQDELEKERDQLQGILASERKMNTLLKKELQADSDAYGDDRRSPL |
| RIVM_H_2014-01 | KLRHLAKLEEMKIRGEQDELEKERDQLQGILASERKMNTLLKKELQADSDAYGDDRRSPL |
| RIVM_H_2014-02 | KLRHLAKLEEMKIRGEQDELEKERDQLQGILASERKMNTLLKKELQADSDAYGDDRRSPL |
| RIVM_H_2016-01 | KLRHLAKLEEMKIRGEQDELEKERDQLQGILASERKMNTLLKKELQADSDAYGDDRRSPL |
| RIVM_H_2016-02 | KLRHLAKLEEMKIRGEQDELEKERDQLQGILASERKMNTLLKKELQADSDAYGDDRRSPL |
| RIVM_H_2016-03 | KLRHLAKLEEMKIRGEQDELEKERDQLQGILASERKMNTLLKKELQADSDAYGDDRRSPL |
| RIVM_H_2016-04 | KLRHLAKLEEMKIRGEQDELEKERDQLQGILASERKMNTLLKKELQADSDAYGDDRRSPL |
| RIVM_H_2016-05 | KLRHLAKLEEMKIRGEQDELEKERDQLQGILASERKMNTLLKKELQADSDAYGDDRRSPL |
| RIVM_H_2016-06 | KLRHLAKLEEMKIRGEQDELEKERDQLQGILASERKMNTLLKKELQADSDAYGDDRRSPL |
| RIVM_H_2016-07 | KLRHLAKLEEMKIRGEQDELEKERDQLQGILASERKMNTLLKKELQADSDAYGDDRRSPL |
| RIVM_H_2016-08 | KLRHLAKLEEMKIRGEQDELEKERDQLQGILASERKMNTLLKKELQADSDAYGDDRRSPL |
| RIVM_H_2016-09 | KLRHLAKLEEMKIRGEQDELEKERDQLQGILASERKMNTLLKKELQADSDAYGDDRRSPL |
| RIVM_H_2016-10 | KLRHLAKLEEMKIRGEQDELEKERDQLQGILASERKMNTLLKKELQADSDAYGDDRRSPL |
| RIVM_H_2016-11 | KLRHLAKLEEMKIRGEQDELEKERDQLQGILASERKMNTLLKKELQADSDAYGDDRRSPL |
| RIVM_H_2016-12 | KLRHLAKLEEMKIRGEQDELEKERDQLQGILASERKMNTLLKKELQADSDAYGDDRRSPL |
| RIVM_H_2016-13 | KLRHLAKLEEMKIRGEQDELEKERDQLQGILASERKMNTLLKKELQADSDAYGDDRRSPL |
| RIVM_H_2016-14 | KLRHLAKLEEMKIRGEQDELEKERDQLQGILASERKMNTLLKKELQADSDAYGDDRRSPL |
| RIVM_H_2016-15 | KLRHLAKLEEMKIRGEQDELEKERDQLQGILASERKMNTLLKKELQADSDAYGDDRRSPL |
| RIVM_H_2017-01 | KLRHLAKLEEMKIRGEQDELEKERDQLQGILASERKMNTLLKKELQADSDAYGDDRRSPL |
| RIVM_H_2017-02 | KLRHLAKLEEMKIRGEQDELEKERDQLQGILASERKMNTLLKKELQADSDAYGDDRRSPL |
| RIVM_H_2017-03 | KLRHLAKLEEMKIRGEQDELEKERDQLQGILASERKMNTLLKKELQADSDAYGDDRRSPL |
| RIVM_H_2017-04 | KLRHLAKLEEMKIRGEQDELEKERDQLQGILASERKMNTLLKKELQADSDAYGDDRRSPL |
| RIVM_H_2017-05 | KLRHLAKLEEMKIRGEQDELEKERDQLQGILASERKMNTLLKKELQADSDAYGDDRRSPL |
| RIVM_H_2017-06 | KLRHLAKLEEMKIRGEQDELEKERDQLQGILASERKMNTLLKKELQADSDAYGDDRRSPL |
| RIVM_H_2017-07 | KLRHLAKLEEMKIRGEQDELEKERDQLQGILASERKMNTLLKKELQADSDAYGDDRRSPL |
| RIVM_H_2017-08 | KLRHLAKLEEMKIRGEQDELEKERDQLQGILASERKMNTLLKKELQADSDAYGDDRRSPL |
| RIVM_H_2017-09 | KLRHLAKLEEMKIRGEQDELEKERDQLQGILASERKMNTLLKKELQADSDAYGDDRRSPL |
| RIVM_H_2017-10 | KLRHLAKLEEMKIRGEQDELEKERDQLQGILASERKMNTLLKKELQADSDAYGDDRRSPL |
| RIVM_H_2017-11 | KLRHLAKLEEMKIRGEQDELEKERDQLQGILASERKMNTLLKKELQADSDAYGDDRRSPL |
| RIVM_H_2017-12 | KLRHLAKLEEMKIRGEQDELEKERDQLQGILASERKMNTLLKKELQADSDAYGDDRRSPL |
| RIVM_H_2017-13 | KLRHLAKLEEMKIRGEQDELEKERDQLQGILASERKMNTLLKKELQADSDAYGDDRRSPL |
| RIVM_H_2017-14 | KLRHLAKLEEMKIRGEQDELEKERDQLQGILASERKMNTLLKKELQADSDAYGDDRRSPL |
| RIVM_H_2017-15 | KLRHLAKLEEMKIRGEQDELEKERDQLQGILASERKMNTLLKKELQADSDAYGDDRRSPL |
| RIVM_H_2017-16 | KLRHLAKLEEMKIRGEQDELEKERDQLQGILASERKMNTLLKKELQADSDAYGDDRRSPL |
| RIVM_H_2017-17 | KLRHLAKLEEMKIRGEQDELEKERDQLQGILASERKMNTLLKKELQADSDAYGDDRRSPL |
| RIVM_H_2017-18 | KLRHLAKLEEMKIRGEQDELEKERDQLQGILASERKMNTLLKKELQADSDAYGDDRRSPL |
| RIVM_H_2017-19 | KLRHLAKLEEMKIRGEQDELEKERDQLQGILASERKMNTLLKKELQADSDAYGDDRRSPL |
| 15EP001483 | KLRHLAKLEEMKIRGEQDELEKERDQLQGILASERKMNTLLKKELQADSDAYGDDRRSPL |
| 17EP002363 | KLRHLAKLEEMKIRGEQDELEKERDQLQGILASERKMNTLLKKELQADSDAYGDDRRSPL |
| S_0812_17 | KLRHLAKLEEMKIRGEQDELEKERDQLQGILASERKMNTLLKKELQADSDAYGDDRRSPL |
| SRR1957844 | KLRHLAKLEEMKIRGEQDELEKERDQLQGILASERKMNTLLKKELQADSDAYGDDRRSPL |
| SRR1958654 | KLRHLAKLEEMKIRGEQDELEKERDQLQGILASERKMNTLLKKELQADSDAYGDDRRSPL |
| SRR1965077 | KLRHLAKLEEMKIRGEQDELEKERDQLQGILASERKMNTLLKKELQADSDAYGDDRRSPL |
| SRR1966369 | KLRHLAKLEEMKIRGEQDELEKERDQLQGILASERKMNTLLKKELQADSDAYGDDRRSPL |
| SRR1967117 | KLRHLAKLEEMKIRGEQDELEKERDQLQGILASERKMNTLLKKELQADSDAYGDDRRSPL |
| SRR1967922 | KLRHLAKLEEMKIRGEQDELEKERDQLQGILASERKMNTLLKKELQADSDAYGDDRRSPL |
| SRR8704720 | KLRHLAKLEEMKIRGEQDELEKERDQLQGILASERKMNTLLKKELQADSDAYGDDRRSPL |
| SRR7216071 | KLRHLAKLEEMKIRGEQDELEKERDQLQGILASERKMNTLLKKELQADSDAYGDDRRSPL |
| SRR7349175 | KLRHLAKLEEMKIRGEQDELEKERDQLQGILASERKMNTLLKKELQADSDAYGDDRRSPL |
| SRR7523148 | KLRHLAKLEEMKIRGEQDELEKERDQLQGILASERKMNTLLKKELQADSDAYGDDRRSPL |
| SRR7523854 | KLRHLAKLEEMKIRGEQDELEKERDQLQGILASERKMNTLLKKELQADSDAYGDDRRSPL |
| 313865 | KLRHLAKLEEMKIRGEQDELEKERDQLQGILASERKMNTLLKKELQADSDAYGDDRRSPL |
| SRR7277793 | KLRHLAKLEEMKIRGEQDELEKERDQLQGILASERKMNTLLKKELQADSDAYGDDRRSPL |
| SRR7343877 | KLRHLAKLEEMKIRGEQDELEKERDQLQGILASERKMNTLLKKELQADSDAYGDDRRSPL |
| SRR7351477 | KLRHLAKLEEMKIRGEQDELEKERDQLQGILASERKMNTLLKKELQADSDAYGDDRRSPL |
| SRR5583183 | KLRHLAKLEEMKIRGEQDELEKERDQLQGILASERKMNTLLKKELQADSDAYGDDRRSPL |
| SRR5585240 | KLRHLAKLEEMKIRGEQDELEKERDQLQGILASERKMNTLLKKELQADSDAYGDDRRSPL |
| SRR7284317 | KLRHLAKLEEMKIRGEQDELEKERDQLQGILASERKMNTLLKKELQADSDAYGDDRRSPL |
| SRR7299161 | KLRHLAKLEEMKIRGEQDELEKERDQLQGILASERKMNTLLKKELQADSDAYGDDRRSPL |
| SRR7401730 | KLRHLAKLEEMKIRGEQDELEKERDQLQGILASERKMNTLLKKELQADSDAYGDDRRSPL |
| SRR7469092 | KLRHLAKLEEMKIRGEQDELEKERDQLQGILASERKMNTLLKKELQADSDAYGDDRRSPL |
| SRR7879556 | KLRHLAKLEEMKIRGEQDELEKERDQLQGILASERKMNTLLKKELQADSDAYGDDRRSPL |
| SRR8526100 | KLRHLAKLEEMKIRGEQDELEKERDQLQGILASERKMNTLLKKELQADSDAYGDDRRSPL |
| SRR8553991 | KLRHLAKLEEMKIRGEQDELEKERDQLQGILASERKMNTLLKKELQADSDAYGDDRRSPL |
| SRR7842487 | KLRHLAKLEEMKIRGEQDELEKERDQLQGILASERKMNTLLKKELQADSDAYGDDRRSPL |
| SRR8054524 | KLRHLAKLEEMKIRGEQDELEKERDQLQGILASERKMNTLLKKELQADSDAYGDDRRSPL |
| SRR8054525 | KLRHLAKLEEMKIRGEQDELEKERDQLQGILASERKMNTLLKKELQADSDAYGDDRRSPL |
| SRR8524733 | KLRHLAKLEEMKIRGEQDELEKERDQLQGILASERKMNTLLKKELQADSDAYGDDRRSPL |
| SRR4093291 | KLRHLAKLEEMKIRGEQDELEKERDQLQGILASERKMNTLLKKELQADSDAYGDDRRSPL |
| SRR4245549 | KLRHLAKLEEMKIRGEQDELEKERDQLQGILASERKMNTLLKKELQADSDAYGDDRRSPL |
| SRR3057154 | KLRHLAKLEEMKIRGEQDELEKERDQLQGILASERKMNTLLKKELQADSDAYGDDRRSPL |
| SRR1726150 | KLRHLAKLEEMKIRGEQDELEKERDQLQGILASERKMNTLLKKELQADSDAYGDDRRSPL |
| SRR1996141 | KLRHLAKLEEMKIRGEQDELEKERDQLQGILASERKMNTLLKKELQADSDAYGDDRRSPL |
| SRR1107842 | KLRHLAKLEEMKIRGEQDELEKERDQLQGILASERKMNTLLKKELQADSDAYGDDRRSPL |
| SRR1157587 | KLRHLAKLEEMKIRGEQDELEKERDQLQGILASERKMNTLLKKELQADSDAYGDDRRSPL |
| SRR3027706 | KLRHLAKLEEMKIRGEQDELEKERDQLQGILASERKMNTLLKKELQADSDAYGDDRRSPL |
| SRR3027707 | KLRHLAKLEEMKIRGEQDELEKERDQLQGILASERKMNTLLKKELQADSDAYGDDRRSPL |
| SRR3027708 | KLRHLAKLEEMKIRGEQDELEKERDQLQGILASERKMNTLLKKELQADSDAYGDDRRSPL |
| SRR3027710 | KLRHLAKLEEMKIRGEQDELEKERDQLQGILASERKMNTLLKKELQADSDAYGDDRRSPL |
| SRR3027711 | KLRHLAKLEEMKIRGEQDELEKERDQLQGILASERKMNTLLKKELQADSDAYGDDRRSPL |
| SRR3027716 | KLRHLAKLEEMKIRGEQDELEKERDQLQGILASERKMNTLLKKELQADSDAYGDDRRSPL |
| SRR3027717 | KLRHLAKLEEMKIRGEQDELEKERDQLQGILASERKMNTLLKKELQADSDAYGDDRRSPL |
| SRR3027719 | KLRHLAKLEEMKIRGEQDELEKERDQLQGILASERKMNTLLKKELQADSDAYGDDRRSPL |
| SRR3027721 | KLRHLAKLEEMKIRGEQDELEKERDQLQGILASERKMNTLLKKELQADSDAYGDDRRSPL |
| SRR3027723 | KLRHLAKLEEMKIRGEQDELEKERDQLQGILASERKMNTLLKKELQADSDAYGDDRRSPL |
| SRR3115978 | KLRHLAKLEEMKIRGEQDELEKERDQLQGILASERKMNTLLKKELQADSDAYGDDRRSPL |
| SRR2534093 | KLRHLAKLEEMKIRGEQDELEKERDQLQGILASERKMNTLLKKELQADSDAYGDDRRSPL |
| SRR2534094 | KLRHLAKLEEMKIRGEQDELEKERDQLQGILASERKMNTLLKKELQADSDAYGDDRRSPL |
| SRR2534095 | KLRHLAKLEEMKIRGEQDELEKERDQLQGILASERKMNTLLKKELQADSDAYGDDRRSPL |
| SRR2534108 | KLRHLAKLEEMKIRGEQDELEKERDQLQGILASERKMNTLLKKELQADSDAYGDDRRSPL |
| SRR1106464 | KLRHLAKLEEMKIRGEQDELEKERDQLQGILASERKMNTLLKKELQADSDAYGDDRRSPL |
| SRR1106463 | KLRHLAKLEEMKIRGEQDELEKERDQLQGILASERKMNTLLKKELQADSDAYGDDRRSPL |
| SRR6949610 | KLRHLAKLEEMKIRGEQDELEKERDQLQGILASERKMNTLLKKELQADSDAYGDDRRSPL |
| SRR6950452 | KLRHLAKLEEMKIRGEQDELEKERDQLQGILASERKMNTLLKKELQADSDAYGDDRRSPL |
| ERR2019831 | KLRHLAKLEEMKIRGEQDELEKERDQLQGILASERKMNTLLKKELQADSDAYGDDRRSPL |
| SRR2085693 | KLRHLAKLEEMKIRGEQDELEKERDQLQGILASERKMNTLLKKELQADSDAYGDDRRSPL |
| SRR2086898 | KLRHLAKLEEMKIRGEQDELEKERDQLQGILASERKMNTLLKKELQADSDAYGDDRRSPL |
| SRR2175312 | KLRHLAKLEEMKIRGEQDELEKERDQLQGILASERKMNTLLKKELQADSDAYGDDRRSPL |
| SRR2175360 | KLRHLAKLEEMKIRGEQDELEKERDQLQGILASERKMNTLLKKELQADSDAYGDDRRSPL |
| SRR5231997 | KLRHLAKLEEMKIRGEQDELEKERDQLQGILASERKMNTLLKKELQADSDAYGDDRRSPL |
| SRR5232003 | KLRHLAKLEEMKIRGEQDELEKERDQLQGILASERKMNTLLKKELQADSDAYGDDRRSPL |
| SRR5232015 | KLRHLAKLEEMKIRGEQDELEKERDQLQGILASERKMNTLLKKELQADSDAYGDDRRSPL |
| SRR949434 | KLRHLAKLEEMKIRGEQDELEKERDQLQGILASERKMNTLLKKELQADSDAYGDDRRSPL |
| SRR3216575 | KLRHLAKLEEMKIRGEQDELEKERDQLQGILASERKMNTLLKKELQADSDAYGDDRRSPL |
| SRR5205342 | KLRHLAKLEEMKIRGEQDELEKERDQLQGILASERKMNTLLKKELQADSDAYGDDRRSPL |
| SRR1501669 | KLRHLAKLEEMKIRGEQDELEKERDQLQGILASERKMNTLLKKELQADSDAYGDDRRSPL |
| SRR5209740 | KLRHLAKLEEMKIRGEQDELEKERDQLQGILASERKMNTLLKKELQADSDAYGDDRRSPL |
| SRR3240355 | KLRHLAKLEEMKIRGEQDELEKERDQLQGILASERKMNTLLKKELQADSDAYGDDRRSPL |
| SRR3392777 | KLRHLAKLEEMKIRGEQDELEKERDQLQGILASERKMNTLLKKELQADSDAYGDDRRSPL |
| SRR3593671 | KLRHLAKLEEMKIRGEQDELEKERDQLQGILASERKMNTLLKKELQADSDAYGDDRRSPL |
| SRR5413290 | KLRHLAKLEEMKIRGEQDELEKERDQLQGILASERKMNTLLKKELQADSDAYGDDRRSPL |
| SRR5590269 | KLRHLAKLEEMKIRGEQDELEKERDQLQGILASERKMNTLLKKELQADSDAYGDDRRSPL |
| SRR5812103 | KLRHLAKLEEMKIRGEQDELEKERDQLQGILASERKMNTLLKKELQADSDAYGDDRRSPL |
| SRR2830941 | KLRHLAKLEEMKIRGEQDELEKERDQLQGILASERKMNTLLKKELQADSDAYGDDRRSPL |
| SRR2830966 | KLRHLAKLEEMKIRGEQDELEKERDQLQGILASERKMNTLLKKELQADSDAYGDDRRSPL |
| SRR3137270 | KLRHLAKLEEMKIRGEQDELEKERDQLQGILASERKMNTLLKKELQADSDAYGDDRRSPL |
| SRR3137271 | KLRHLAKLEEMKIRGEQDELEKERDQLQGILASERKMNTLLKKELQADSDAYGDDRRSPL |
| ERR526807 | KLRHLAKLEEMKIRGEQDELEKERDQLQGILASERKMNTLLKKELQADSDAYGDDRRSPL |
| ERR2197922 | KLRHLAKLEEMKIRGEQDELEKERDQLQGILASERKMNTLLKKELQADSDAYGDDRRSPL |
| ERR2197923 | KLRHLAKLEEMKIRGEQDELEKERDQLQGILASERKMNTLLKKELQADSDAYGDDRRSPL |
| ERR2197924 | KLRHLAKLEEMKIRGEQDELEKERDQLQGILASERKMNTLLKKELQADSDAYGDDRRSPL |
| ERR2197925 | KLRHLAKLEEMKIRGEQDELEKERDQLQGILASERKMNTLLKKELQADSDAYGDDRRSPL |
| ERR2197927 | KLRHLAKLEEMKIRGEQDELEKERDQLQGILASERKMNTLLKKELQADSDAYGDDRRSPL |
| ERR2197929 | KLRHLAKLEEMKIRGEQDELEKERDQLQGILASERKMNTLLKKELQADSDAYGDDRRSPL |
| SRR1648149 | KLRHLAKLEEMKIRGEQDELEKERDQLQGILASERKMNTLLKKELQADSDAYGDDRRSPL |
| SRR1048299 | KLRHLAKLEEMKIRGEQDELEKERDQLQGILASERKMNTLLKKELQADSDAYGDDRRSPL |
| SRR1300677 | KLRHLAKLEEMKIRGEQDELEKERDQLQGILASERKMNTLLKKELQADSDAYGDDRRSPL |
| SRR1288356 | KLRHLAKLEEMKIRGEQDELEKERDQLQGILASERKMNTLLKKELQADSDAYGDDRRSPL |
| SRR7426190 | KLRHLAKLEEMKIRGEQDELEKERDQLQGILASERKMNTLLKKELQADSDAYGDDRRSPL |
| SRR7426192 | KLRHLAKLEEMKIRGEQDELEKERDQLQGILASERKMNTLLKKELQADSDAYGDDRRSPL |
| SRR7426193 | KLRHLAKLEEMKIRGEQDELEKERDQLQGILASERKMNTLLKKELQADSDAYGDDRRSPL |
| SRR7441832 | KLRHLAKLEEMKIRGEQDELEKERDQLQGILASERKMNTLLKKELQADSDAYGDDRRSPL |
| SRR7426179 | KLRHLAKLEEMKIRGEQDELEKERDQLQGILASERKMNTLLKKELQADSDAYGDDRRSPL |
| SRR7439238 | KLRHLAKLEEMKIRGEQDELEKERDQLQGILASERKMNTLLKKELQADSDAYGDDRRSPL |
| SRR7439244 | KLRHLAKLEEMKIRGEQDELEKERDQLQGILASERKMNTLLKKELQADSDAYGDDRRSPL |
| SRR7439259 | KLRHLAKLEEMKIRGEQDELEKERDQLQGILASERKMNTLLKKELQADSDAYGDDRRSPL |
| SRR7439260 | KLRHLAKLEEMKIRGEQDELEKERDQLQGILASERKMNTLLKKELQADSDAYGDDRRSPL |
| SRR7441786 | KLRHLAKLEEMKIRGEQDELEKERDQLQGILASERKMNTLLKKELQADSDAYGDDRRSPL |
| SRR7441797 | KLRHLAKLEEMKIRGEQDELEKERDQLQGILASERKMNTLLKKELQADSDAYGDDRRSPL |
| ERR1759093 | KLRHLAKLEEMKIRGEQDELEKERDQLQGILASERKMNTLLKKELQADSDAYGDDRRSPL |
| ERR2580275 | KLRHLAKLEEMKIRGEQDELEKERDQLQGILASERKMNTLLKKELQADSDAYGDDRRSPL |
| ERR1759204 | KLRHLAKLEEMKIRGEQDELEKERDQLQGILASERKMNTLLKKELQADSDAYGDDRRSPL |
| SRR1300699 | KLRHLAKLEEMKIRGEQDELEKERDQLQGILASERKMNTLLKKELQADSDAYGDDRRSPL |
| S_0825_17 | KLRHLAKLEEMKIRGEQDELEKERDQLQGILASERKMNTLLKKELQADSDAYGDDRRSPL |
| SRR1958215 | KLRHLAKLEEMKIRGEQDELEKERDQLQGILASERKMNTLLKKELQADSDAYGDDRRSPL |
| SRR1958540 | KLRHLAKLEEMKIRGEQDELEKERDQLQGILASERKMNTLLKKELQADSDAYGDDRRSPL |
| SRR1958636 | KLRHLAKLEEMKIRGEQDELEKERDQLQGILASERKMNTLLKKELQADSDAYGDDRRSPL |
| SRR1959422 | KLRHLAKLEEMKIRGEQDELEKERDQLQGILASERKMNTLLKKELQADSDAYGDDRRSPL |
| SRR1959427 | KLRHLAKLEEMKIRGEQDELEKERDQLQGILASERKMNTLLKKELQADSDAYGDDRRSPL |
| SRR1960226 | KLRHLAKLEEMKIRGEQDELEKERDQLQGILASERKMNTLLKKELQADSDAYGDDRRSPL |
| SRR1963498 | KLRHLAKLEEMKIRGEQDELEKERDQLQGILASERKMNTLLKKELQADSDAYGDDRRSPL |
| SRR1965947 | KLRHLAKLEEMKIRGEQDELEKERDQLQGILASERKMNTLLKKELQADSDAYGDDRRSPL |
| SRR1966125 | KLRHLAKLEEMKIRGEQDELEKERDQLQGILASERKMNTLLKKELQADSDAYGDDRRSPL |
| SRR1966330 | KLRHLAKLEEMKIRGEQDELEKERDQLQGILASERKMNTLLKKELQADSDAYGDDRRSPL |
| SRR1966565 | KLRHLAKLEEMKIRGEQDELEKERDQLQGILASERKMNTLLKKELQADSDAYGDDRRSPL |
| SRR1966864 | KLRHLAKLEEMKIRGEQDELEKERDQLQGILASERKMNTLLKKELQADSDAYGDDRRSPL |
| SRR1966989 | KLRHLAKLEEMKIRGEQDELEKERDQLQGILASERKMNTLLKKELQADSDAYGDDRRSPL |
| SRR1967688 | KLRHLAKLEEMKIRGEQDELEKERDQLQGILASERKMNTLLKKELQADSDAYGDDRRSPL |
| SRR1967733 | KLRHLAKLEEMKIRGEQDELEKERDQLQGILASERKMNTLLKKELQADSDAYGDDRRSPL |
| SRR1967746 | KLRHLAKLEEMKIRGEQDELEKERDQLQGILASERKMNTLLKKELQADSDAYGDDRRSPL |
| SRR1968341 | KLRHLAKLEEMKIRGEQDELEKERDQLQGILASERKMNTLLKKELQADSDAYGDDRRSPL |
| SRR1968456 | KLRHLAKLEEMKIRGEQDELEKERDQLQGILASERKMNTLLKKELQADSDAYGDDRRSPL |
| SRR1968465 | KLRHLAKLEEMKIRGEQDELEKERDQLQGILASERKMNTLLKKELQADSDAYGDDRRSPL |
| SRR1968761 | KLRHLAKLEEMKIRGEQDELEKERDQLQGILASERKMNTLLKKELQADSDAYGDDRRSPL |
| SRR1969047 | KLRHLAKLEEMKIRGEQDELEKERDQLQGILASERKMNTLLKKELQADSDAYGDDRRSPL |
| SRR1969255 | KLRHLAKLEEMKIRGEQDELEKERDQLQGILASERKMNTLLKKELQADSDAYGDDRRSPL |
| SRR1969412 | KLRHLAKLEEMKIRGEQDELEKERDQLQGILASERKMNTLLKKELQADSDAYGDDRRSPL |
| SRR1969524 | KLRHLAKLEEMKIRGEQDELEKERDQLQGILASERKMNTLLKKELQADSDAYGDDRRSPL |
| SRR1969584 | KLRHLAKLEEMKIRGEQDELEKERDQLQGILASERKMNTLLKKELQADSDAYGDDRRSPL |
| SRR1969648 | KLRHLAKLEEMKIRGEQDELEKERDQLQGILASERKMNTLLKKELQADSDAYGDDRRSPL |
| SRR1969804 | KLRHLAKLEEMKIRGEQDELEKERDQLQGILASERKMNTLLKKELQADSDAYGDDRRSPL |
| SRR1970221 | KLRHLAKLEEMKIRGEQDELEKERDQLQGILASERKMNTLLKKELQADSDAYGDDRRSPL |
| SRR1970268 | KLRHLAKLEEMKIRGEQDELEKERDQLQGILASERKMNTLLKKELQADSDAYGDDRRSPL |
| SRR1965862 | KLRHLAKLEEMKIRGEQDELEKERDQLQGILASERKMNTLLKKELQADSDAYGDDRRSPL |
| SRR1967363 | KLRHLAKLEEMKIRGEQDELEKERDQLQGILASERKMNTLLKKELQADSDAYGDDRRSPL |
| SRR1968276 | KLRHLAKLEEMKIRGEQDELEKERDQLQGILASERKMNTLLKKELQADSDAYGDDRRSPL |
| SRR1968967 | KLRHLAKLEEMKIRGEQDELEKERDQLQGILASERKMNTLLKKELQADSDAYGDDRRSPL |
| SRR3321531 | KLRHLAKLEEMKIRGEQDELEKERDQLQGILASERKMNTLLKKELQADSDAYGDDRRSPL |
| SRR3321883 | KLRHLAKLEEMKIRGEQDELEKERDQLQGILASERKMNTLLKKELQADSDAYGDDRRSPL |
| SRR3322413 | KLRHLAKLEEMKIRGEQDELEKERDQLQGILASERKMNTLLKKELQADSDAYGDDRRSPL |
| SRR3323012 | KLRHLAKLEEMKIRGEQDELEKERDQLQGILASERKMNTLLKKELQADSDAYGDDRRSPL |
| SRR5194289 | KLRHLAKLEEMKIRGEQDELEKERDQLQGILASERKMNTLLKKELQADSDAYGDDRRSPL |
| SRR7163798 | KLRHLAKLEEMKIRGEQDELEKERDQLQGILASERKMNTLLKKELQADSDAYGDDRRSPL |
| SRR7172610 | KLRHLAKLEEMKIRGEQDELEKERDQLQGILASERKMNTLLKKELQADSDAYGDDRRSPL |
| SRR7204568 | KLRHLAKLEEMKIRGEQDELEKERDQLQGILASERKMNTLLKKELQADSDAYGDDRRSPL |
| SRR7223230 | KLRHLAKLEEMKIRGEQDELEKERDQLQGILASERKMNTLLKKELQADSDAYGDDRRSPL |
| SRR7230675 | KLRHLAKLEEMKIRGEQDELEKERDQLQGILASERKMNTLLKKELQADSDAYGDDRRSPL |
| SRR7278056 | KLRHLAKLEEMKIRGEQDELEKERDQLQGILASERKMNTLLKKELQADSDAYGDDRRSPL |
| SRR7278086 | KLRHLAKLEEMKIRGEQDELEKERDQLQGILASERKMNTLLKKELQADSDAYGDDRRSPL |
| SRR7285841 | KLRHLAKLEEMKIRGEQDELEKERDQLQGILASERKMNTLLKKELQADSDAYGDDRRSPL |
| SRR7292625 | KLRHLAKLEEMKIRGEQDELEKERDQLQGILASERKMNTLLKKELQADSDAYGDDRRSPL |
| SRR7292665 | KLRHLAKLEEMKIRGEQDELEKERDQLQGILASERKMNTLLKKELQADSDAYGDDRRSPL |
| SRR7297965 | KLRHLAKLEEMKIRGEQDELEKERDQLQGILASERKMNTLLKKELQADSDAYGDDRRSPL |
| SRR7350726 | KLRHLAKLEEMKIRGEQDELEKERDQLQGILASERKMNTLLKKELQADSDAYGDDRRSPL |
| SRR7410328 | KLRHLAKLEEMKIRGEQDELEKERDQLQGILASERKMNTLLKKELQADSDAYGDDRRSPL |
| SRR7474665 | KLRHLAKLEEMKIRGEQDELEKERDQLQGILASERKMNTLLKKELQADSDAYGDDRRSPL |
| SRR7523184 | KLRHLAKLEEMKIRGEQDELEKERDQLQGILASERKMNTLLKKELQADSDAYGDDRRSPL |
| SRR7187264 | KLRHLAKLEEMKIRGEQDELEKERDQLQGILASERKMNTLLKKELQADSDAYGDDRRSPL |
| SRR7204445 | KLRHLAKLEEMKIRGEQDELEKERDQLQGILASERKMNTLLKKELQADSDAYGDDRRSPL |
| SRR7285641 | KLRHLAKLEEMKIRGEQDELEKERDQLQGILASERKMNTLLKKELQADSDAYGDDRRSPL |
| SRR7286695 | KLRHLAKLEEMKIRGEQDELEKERDQLQGILASERKMNTLLKKELQADSDAYGDDRRSPL |
| SRR7286705 | KLRHLAKLEEMKIRGEQDELEKERDQLQGILASERKMNTLLKKELQADSDAYGDDRRSPL |
| SRR7292931 | KLRHLAKLEEMKIRGEQDELEKERDQLQGILASERKMNTLLKKELQADSDAYGDDRRSPL |
| SRR7310349 | KLRHLAKLEEMKIRGEQDELEKERDQLQGILASERKMNTLLKKELQADSDAYGDDRRSPL |
| SRR7351616 | KLRHLAKLEEMKIRGEQDELEKERDQLQGILASERKMNTLLKKELQADSDAYGDDRRSPL |
| SRR7414818 | KLRHLAKLEEMKIRGEQDELEKERDQLQGILASERKMNTLLKKELQADSDAYGDDRRSPL |
| SRR7426480 | KLRHLAKLEEMKIRGEQDELEKERDQLQGILASERKMNTLLKKELQADSDAYGDDRRSPL |
| SRR5584105 | KLRHLAKLEEMKIRGEQDELEKERDQLQGILASERKMNTLLKKELQADSDAYGDDRRSPL |
| SRR5584565 | KLRHLAKLEEMKIRGEQDELEKERDQLQGILASERKMNTLLKKELQADSDAYGDDRRSPL |
| SRR5584614 | KLRHLAKLEEMKIRGEQDELEKERDQLQGILASERKMNTLLKKELQADSDAYGDDRRSPL |
| SRR5631543 | KLRHLAKLEEMKIRGEQDELEKERDQLQGILASERKMNTLLKKELQADSDAYGDDRRSPL |
| SRR5631553 | KLRHLAKLEEMKIRGEQDELEKERDQLQGILASERKMNTLLKKELQADSDAYGDDRRSPL |
| SRR7123196 | KLRHLAKLEEMKIRGEQDELEKERDQLQGILASERKMNTLLKKELQADSDAYGDDRRSPL |
| SRR7163819 | KLRHLAKLEEMKIRGEQDELEKERDQLQGILASERKMNTLLKKELQADSDAYGDDRRSPL |
| SRR7163920 | KLRHLAKLEEMKIRGEQDELEKERDQLQGILASERKMNTLLKKELQADSDAYGDDRRSPL |
| SRR7209528 | KLRHLAKLEEMKIRGEQDELEKERDQLQGILASERKMNTLLKKELQADSDAYGDDRRSPL |
| SRR7249868 | KLRHLAKLEEMKIRGEQDELEKERDQLQGILASERKMNTLLKKELQADSDAYGDDRRSPL |
| SRR7278088 | KLRHLAKLEEMKIRGEQDELEKERDQLQGILASERKMNTLLKKELQADSDAYGDDRRSPL |
| SRR7285788 | KLRHLAKLEEMKIRGEQDELEKERDQLQGILASERKMNTLLKKELQADSDAYGDDRRSPL |
| SRR7286789 | KLRHLAKLEEMKIRGEQDELEKERDQLQGILASERKMNTLLKKELQADSDAYGDDRRSPL |
| SRR7286886 | KLRHLAKLEEMKIRGEQDELEKERDQLQGILASERKMNTLLKKELQADSDAYGDDRRSPL |
| SRR7310632 | KLRHLAKLEEMKIRGEQDELEKERDQLQGILASERKMNTLLKKELQADSDAYGDDRRSPL |
| SRR7350631 | KLRHLAKLEEMKIRGEQDELEKERDQLQGILASERKMNTLLKKELQADSDAYGDDRRSPL |
| SRR7458741 | KLRHLAKLEEMKIRGEQDELEKERDQLQGILASERKMNTLLKKELQADSDAYGDDRRSPL |
| SRR7480280 | KLRHLAKLEEMKIRGEQDELEKERDQLQGILASERKMNTLLKKELQADSDAYGDDRRSPL |
| SRR7523660 | KLRHLAKLEEMKIRGEQDELEKERDQLQGILASERKMNTLLKKELQADSDAYGDDRRSPL |
| SRR7523775 | KLRHLAKLEEMKIRGEQDELEKERDQLQGILASERKMNTLLKKELQADSDAYGDDRRSPL |
| SRR7251101 | KLRHLAKLEEMKIRGEQDELEKERDQLQGILASERKMNTLLKKELQADSDAYGDDRRSPL |
| SRR7284299 | KLRHLAKLEEMKIRGEQDELEKERDQLQGILASERKMNTLLKKELQADSDAYGDDRRSPL |
| SRR7285738 | KLRHLAKLEEMKIRGEQDELEKERDQLQGILASERKMNTLLKKELQADSDAYGDDRRSPL |
| SRR7310640 | KLRHLAKLEEMKIRGEQDELEKERDQLQGILASERKMNTLLKKELQADSDAYGDDRRSPL |
| SRR7349159 | KLRHLAKLEEMKIRGEQDELEKERDQLQGILASERKMNTLLKKELQADSDAYGDDRRSPL |
| SRR7474873 | KLRHLAKLEEMKIRGEQDELEKERDQLQGILASERKMNTLLKKELQADSDAYGDDRRSPL |
| SRR7495689 | KLRHLAKLEEMKIRGEQDELEKERDQLQGILASERKMNTLLKKELQADSDAYGDDRRSPL |
| SRR7495752 | KLRHLAKLEEMKIRGEQDELEKERDQLQGILASERKMNTLLKKELQADSDAYGDDRRSPL |
| ------------------------------------------------------------------------------ | |
| S16BD08730 | REREEAKAMSEHDMLPSEPVTIVLSQMGWVRSAKGHDIDAPGLNYKAGDSFKAAVKGKSN |
| S18BD00684 | REREEAKAMSEHDMLPSEPVTIVLSQMGWVRSAKGHDIDAPGLNYKAGDSFKAAVKGKSN |
| S18BD03994 | REREEAKAMSEHDMLPSEPVTIVLSQMGWVRSAKGHDIDAPGLNYKAGDSFKAAVKGKSN |
| S18BD05011 | REREEAKAMSEHDMLPSEPVTIVLSQMGWVRSAKGHDIDAPGLNYKAGDSFKAAVKGKSN |
| RKI_16-03723 | REREEAKAMSEHDMLPSEPVTIVLSQMGWVRSAKGHDIDAPGLNYKAGDSFKAAVKGKSN |
| RKI_16-04315 | REREEAKAMSEHDMLPSEPVTIVLSQMGWVRSAKGHDIDAPGLNYKAGDSFKAAVKGKSN |
| RKI_17-02304 | REREEAKAMSEHDMLPSEPVTIVLSQMGWVRSAKGHDIDAPGLNYKAGDSFKAAVKGKSN |
| RKI_17-02411 | REREEAKAMSEHDMLPSEPVTIVLSQMGWVRSAKGHDIDAPGLNYKAGDSFKAAVKGKSN |
| RKI_17-02757 | REREEAKAMSEHDMLPSEPVTIVLSQMGWVRSAKGHDIDAPGLNYKAGDSFKAAVKGKSN |
| RKI_17-04797 | REREEAKAMSEHDMLPSEPVTIVLSQMGWVRSAKGHDIDAPGLNYKAGDSFKAAVKGKSN |
| RKI_17-06869 | REREEAKAMSEHDMLPSEPVTIVLSQMGWVRSAKGHDIDAPGLNYKAGDSFKAAVKGKSN |
| ERR2580277 | REREEAKAMSEHDMLPSEPVTIVLSQMGWVRSAKGHDIDAPGLNYKAGDSFKAAVKGKSN |
| ERR2580276 | REREEAKAMSEHDMLPSEPVTIVLSQMGWVRSAKGHDIDAPGLNYKAGDSFKAAVKGKSN |
| ERR2580273 | REREEAKAMSEHDMLPSEPVTIVLSQMGWVRSAKGHDIDAPGLNYKAGDSFKAAVKGKSN |
| ERR2580274 | REREEAKAMSEHDMLPSEPVTIVLSQMGWVRSAKGHDIDAPGLNYKAGDSFKAAVKGKSN |
| ERR2173656 | REREEAKAMSEHDMLPSEPVTIVLSQMGWVRSAKGHDIDAPGLNYKAGDSFKAAVKGKSN |
| 17041676 | REREEAKAMSEHDMLPSEPVTIVLSQMGWVRSAKGHDIDAPGLNYKAGDSFKAAVKGKSN |
| MT16-000061 | REREEAKAMSEHDMLPSEPVTIVLSQMGWVRSAKGHDIDAPGLNYKAGDSFKAAVKGKSN |
| MT16-019416 | REREEAKAMSEHDMLPSEPVTIVLSQMGWVRSAKGHDIDAPGLNYKAGDSFKAAVKGKSN |
| MT16-027865 | REREEAKAMSEHDMLPSEPVTIVLSQMGWVRSAKGHDIDAPGLNYKAGDSFKAAVKGKSN |
| MT16-031693 | REREEAKAMSEHDMLPSEPVTIVLSQMGWVRSAKGHDIDAPGLNYKAGDSFKAAVKGKSN |
| MT16-040253 | REREEAKAMSEHDMLPSEPVTIVLSQMGWVRSAKGHDIDAPGLNYKAGDSFKAAVKGKSN |
| MT16-045379 | REREEAKAMSEHDMLPSEPVTIVLSQMGWVRSAKGHDIDAPGLNYKAGDSFKAAVKGKSN |
| MT16-442728 | REREEAKAMSEHDMLPSEPVTIVLSQMGWVRSAKGHDIDAPGLNYKAGDSFKAAVKGKSN |
| MT16-462857 | REREEAKAMSEHDMLPSEPVTIVLSQMGWVRSAKGHDIDAPGLNYKAGDSFKAAVKGKSN |
| MT16-480196 | REREEAKAMSEHDMLPSEPVTIVLSQMGWVRSAKGHDIDAPGLNYKAGDSFKAAVKGKSN |
| MT16-861555 | REREEAKAMSEHDMLPSEPVTIVLSQMGWVRSAKGHDIDAPGLNYKAGDSFKAAVKGKSN |
| MT17-076833 | REREEAKAMSEHDMLPSEPVTIVLSQMGWVRSAKGHDIDAPGLNYKAGDSFKAAVKGKSN |
| MT17-110677 | REREEAKAMSEHDMLPSEPVTIVLSQMGWVRSAKGHDIDAPGLNYKAGDSFKAAVKGKSN |
| MT17-131730 | REREEAKAMSEHDMLPSEPVTIVLSQMGWVRSAKGHDIDAPGLNYKAGDSFKAAVKGKSN |
| MT17-140890 | REREEAKAMSEHDMLPSEPVTIVLSQMGWVRSAKGHDIDAPGLNYKAGDSFKAAVKGKSN |
| MT17-141840 | REREEAKAMSEHDMLPSEPVTIVLSQMGWVRSAKGHDIDAPGLNYKAGDSFKAAVKGKSN |
| MT17-152488 | REREEAKAMSEHDMLPSEPVTIVLSQMGWVRSAKGHDIDAPGLNYKAGDSFKAAVKGKSN |
| MT17-157311 | REREEAKAMSEHDMLPSEPVTIVLSQMGWVRSAKGHDIDAPGLNYKAGDSFKAAVKGKSN |
| MT17-161645 | REREEAKAMSEHDMLPSEPVTIVLSQMGWVRSAKGHDIDAPGLNYKAGDSFKAAVKGKSN |
| MT17-167951 | REREEAKAMSEHDMLPSEPVTIVLSQMGWVRSAKGHDIDAPGLNYKAGDSFKAAVKGKSN |
| MT18-217732 | REREEAKAMSEHDMLPSEPVTIVLSQMGWVRSAKGHDIDAPGLNYKAGDSFKAAVKGKSN |
| MT18-252580 | REREEAKAMSEHDMLPSEPVTIVLSQMGWVRSAKGHDIDAPGLNYKAGDSFKAAVKGKSN |
| RIVM_H_2009-01 | REREEAKAMSEHDMLPSEPVTIVLSQMGWVRSAKGHDIDAPGLNYKAGDSFKAAVKGKSN |
| RIVM_H_2010-01 | REREEAKAMSEHDMLPSEPVTIVLSQMGWVRSAKGHDIDAPGLNYKAGDSFKAAVKGKSN |
| RIVM_H_2010-02 | REREEAKAMSEHDMLPSEPVTIVLSQMGWVRSAKGHDIDAPGLNYKAGDSFKAAVKGKSN |
| RIVM_H_2011-01 | REREEAKAMSEHDMLPSEPVTIVLSQMGWVRSAKGHDIDAPGLNYKAGDSFKAAVKGKSN |
| RIVM_H_2011-02 | REREEAKAMSEHDMLPSEPVTIVLSQMGWVRSAKGHDIDAPGLNYKAGDSFKAAVKGKSN |
| RIVM_H_2011-03 | REREEAKAMSEHDMLPSEPVTIVLSQMGWVRSAKGHDIDAPGLNYKAGDSFKAAVKGKSN |
| RIVM_H_2013-01 | REREEAKAMSEHDMLPSEPVTIVLSQMGWVRSAKGHDIDAPGLNYKAGDSFKAAVKGKSN |
| RIVM_H_2013-02 | REREEAKAMSEHDMLPSEPVTIVLSQMGWVRSAKGHDIDAPGLNYKAGDSFKAAVKGKSN |
| RIVM_H_2014-01 | REREEAKAMSEHDMLPSEPVTIVLSQMGWVRSAKGHDIDAPGLNYKAGDSFKAAVKGKSN |
| RIVM_H_2014-02 | REREEAKAMSEHDMLPSEPVTIVLSQMGWVRSAKGHDIDAPGLNYKAGDSFKAAVKGKSN |
| RIVM_H_2016-01 | REREEAKAMSEHDMLPSEPVTIVLSQMGWVRSAKGHDIDAPGLNYKAGDSFKAAVKGKSN |
| RIVM_H_2016-02 | REREEAKAMSEHDMLPSEPVTIVLSQMGWVRSAKGHDIDAPGLNYKAGDSFKAAVKGKSN |
| RIVM_H_2016-03 | REREEAKAMSEHDMLPSEPVTIVLSQMGWVRSAKGHDIDAPGLNYKAGDSFKAAVKGKSN |
| RIVM_H_2016-04 | REREEAKAMSEHDMLPSEPVTIVLSQMGWVRSAKGHDIDAPGLNYKAGDSFKAAVKGKSN |
| RIVM_H_2016-05 | REREEAKAMSEHDMLPSEPVTIVLSQMGWVRSAKGHDIDAPGLNYKAGDSFKAAVKGKSN |
| RIVM_H_2016-06 | REREEAKAMSEHDMLPSEPVTIVLSQMGWVRSAKGHDIDAPGLNYKAGDSFKAAVKGKSN |
| RIVM_H_2016-07 | REREEAKAMSEHDMLPSEPVTIVLSQMGWVRSAKGHDIDAPGLNYKAGDSFKAAVKGKSN |
| RIVM_H_2016-08 | REREEAKAMSEHDMLPSEPVTIVLSQMGWVRSAKGHDIDAPGLNYKAGDSFKAAVKGKSN |
| RIVM_H_2016-09 | REREEAKAMSEHDMLPSEPVTIVLSQMGWVRSAKGHDIDAPGLNYKAGDSFKAAVKGKSN |
| RIVM_H_2016-10 | REREEAKAMSEHDMLPSEPVTIVLSQMGWVRSAKGHDIDAPGLNYKAGDSFKAAVKGKSN |
| RIVM_H_2016-11 | REREEAKAMSEHDMLPSEPVTIVLSQMGWVRSAKGHDIDAPGLNYKAGDSFKAAVKGKSN |
| RIVM_H_2016-12 | REREEAKAMSEHDMLPSEPVTIVLSQMGWVRSAKGHDIDAPGLNYKAGDSFKAAVKGKSN |
| RIVM_H_2016-13 | REREEAKAMSEHDMLPSEPVTIVLSQMGWVRSAKGHDIDAPGLNYKAGDSFKAAVKGKSN |
| RIVM_H_2016-14 | REREEAKAMSEHDMLPSEPVTIVLSQMGWVRSAKGHDIDAPGLNYKAGDSFKAAVKGKSN |
| RIVM_H_2016-15 | REREEAKAMSEHDMLPSEPVTIVLSQMGWVRSAKGHDIDAPGLNYKAGDSFKAAVKGKSN |
| RIVM_H_2017-01 | REREEAKAMSEHDMLPSEPVTIVLSQMGWVRSAKGHDIDAPGLNYKAGDSFKAAVKGKSN |
| RIVM_H_2017-02 | REREEAKAMSEHDMLPSEPVTIVLSQMGWVRSAKGHDIDAPGLNYKAGDSFKAAVKGKSN |
| RIVM_H_2017-03 | REREEAKAMSEHDMLPSEPVTIVLSQMGWVRSAKGHDIDAPGLNYKAGDSFKAAVKGKSN |
| RIVM_H_2017-04 | REREEAKAMSEHDMLPSEPVTIVLSQMGWVRSAKGHDIDAPGLNYKAGDSFKAAVKGKSN |
| RIVM_H_2017-05 | REREEAKAMSEHDMLPSEPVTIVLSQMGWVRSAKGHDIDAPGLNYKAGDSFKAAVKGKSN |
| RIVM_H_2017-06 | REREEAKAMSEHDMLPSEPVTIVLSQMGWVRSAKGHDIDAPGLNYKAGDSFKAAVKGKSN |
| RIVM_H_2017-07 | REREEAKAMSEHDMLPSEPVTIVLSQMGWVRSAKGHDIDAPGLNYKAGDSFKAAVKGKSN |
| RIVM_H_2017-08 | REREEAKAMSEHDMLPSEPVTIVLSQMGWVRSAKGHDIDAPGLNYKAGDSFKAAVKGKSN |
| RIVM_H_2017-09 | REREEAKAMSEHDMLPSEPVTIVLSQMGWVRSAKGHDIDAPGLNYKAGDSFKAAVKGKSN |
| RIVM_H_2017-10 | REREEAKAMSEHDMLPSEPVTIVLSQMGWVRSAKGHDIDAPGLNYKAGDSFKAAVKGKSN |
| RIVM_H_2017-11 | REREEAKAMSEHDMLPSEPVTIVLSQMGWVRSAKGHDIDAPGLNYKAGDSFKAAVKGKSN |
| RIVM_H_2017-12 | REREEAKAMSEHDMLPSEPVTIVLSQMGWVRSAKGHDIDAPGLNYKAGDSFKAAVKGKSN |
| RIVM_H_2017-13 | REREEAKAMSEHDMLPSEPVTIVLSQMGWVRSAKGHDIDAPGLNYKAGDSFKAAVKGKSN |
| RIVM_H_2017-14 | REREEAKAMSEHDMLPSEPVTIVLSQMGWVRSAKGHDIDAPGLNYKAGDSFKAAVKGKSN |
| RIVM_H_2017-15 | REREEAKAMSEHDMLPSEPVTIVLSQMGWVRSAKGHDIDAPGLNYKAGDSFKAAVKGKSN |
| RIVM_H_2017-16 | REREEAKAMSEHDMLPSEPVTIVLSQMGWVRSAKGHDIDAPGLNYKAGDSFKAAVKGKSN |
| RIVM_H_2017-17 | REREEAKAMSEHDMLPSEPVTIVLSQMGWVRSAKGHDIDAPGLNYKAGDSFKAAVKGKSN |
| RIVM_H_2017-18 | REREEAKAMSEHDMLPSEPVTIVLSQMGWVRSAKGHDIDAPGLNYKAGDSFKAAVKGKSN |
| RIVM_H_2017-19 | REREEAKAMSEHDMLPSEPVTIVLSQMGWVRSAKGHDIDAPGLNYKAGDSFKAAVKGKSN |
| 15EP001483 | REREEAKAMSEHDMLPSEPVTIVLSQMGWVRSAKGHDIDAPGLNYKAGDSFKAAVKGKSN |
| 17EP002363 | REREEAKAMSEHDMLPSEPVTIVLSQMGWVRSAKGHDIDAPGLNYKAGDSFKAAVKGKSN |
| S_0812_17 | REREEAKAMSEHDMLPSEPVTIVLSQMGWVRSAKGHDIDAPGLNYKAGDSFKAAVKGKSN |
| SRR1957844 | REREEAKAMSEHDMLPSEPVTIVLSQMGWVRSAKGHDIDAPGLNYKAGDSFKAAVKGKSN |
| SRR1958654 | REREEAKAMSEHDMLPSEPVTIVLSQMGWVRSAKGHDIDAPGLNYKAGDSFKAAVKGKSN |
| SRR1965077 | REREEAKAMSEHDMLPSEPVTIVLSQMGWVRSAKGHDIDAPGLNYKAGDSFKAAVKGKSN |
| SRR1966369 | REREEAKAMSEHDMLPSEPVTIVLSQMGWVRSAKGHDIDAPGLNYKAGDSFKAAVKGKSN |
| SRR1967117 | REREEAKAMSEHDMLPSEPVTIVLSQMGWVRSAKGHDIDAPGLNYKAGDSFKAAVKGKSN |
| SRR1967922 | REREEAKAMSEHDMLPSEPVTIVLSQMGWVRSAKGHDIDAPGLNYKAGDSFKAAVKGKSN |
| SRR8704720 | REREEAKAMSEHDMLPSEPVTIVLSQMGWVRSAKGHDIDAPGLNYKAGDSFKAAVKGKSN |
| SRR7216071 | REREEAKAMSEHDMLPSEPVTIVLSQMGWVRSAKGHDIDAPGLNYKAGDSFKAAVKGKSN |
| SRR7349175 | REREEAKAMSEHDMLPSEPVTIVLSQMGWVRSAKGHDIDAPGLNYKAGDSFKAAVKGKSN |
| SRR7523148 | REREEAKAMSEHDMLPSEPVTIVLSQMGWVRSAKGHDIDAPGLNYKAGDSFKAAVKGKSN |
| SRR7523854 | REREEAKAMSEHDMLPSEPVTIVLSQMGWVRSAKGHDIDAPGLNYKAGDSFKAAVKGKSN |
| 313865 | REREEAKAMSEHDMLPSEPVTIVLSQMGWVRSAKGHDIDAPGLNYKAGDSFKAAVKGKSN |
| SRR7277793 | REREEAKAMSEHDMLPSEPVTIVLSQMGWVRSAKGHDIDAPGLNYKAGDSFKAAVKGKSN |
| SRR7343877 | REREEAKAMSEHDMLPSEPVTIVLSQMGWVRSAKGHDIDAPGLNYKAGDSFKAAVKGKSN |
| SRR7351477 | REREEAKAMSEHDMLPSEPVTIVLSQMGWVRSAKGHDIDAPGLNYKAGDSFKAAVKGKSN |
| SRR5583183 | REREEAKAMSEHDMLPSEPVTIVLSQMGWVRSAKGHDIDAPGLNYKAGDSFKAAVKGKSN |
| SRR5585240 | REREEAKAMSEHDMLPSEPVTIVLSQMGWVRSAKGHDIDAPGLNYKAGDSFKAAVKGKSN |
| SRR7284317 | REREEAKAMSEHDMLPSEPVTIVLSQMGWVRSAKGHDIDAPGLNYKAGDSFKAAVKGKSN |
| SRR7299161 | REREEAKAMSEHDMLPSEPVTIVLSQMGWVRSAKGHDIDAPGLNYKAGDSFKAAVKGKSN |
| SRR7401730 | REREEAKAMSEHDMLPSEPVTIVLSQMGWVRSAKGHDIDAPGLNYKAGDSFKAAVKGKSN |
| SRR7469092 | REREEAKAMSEHDMLPSEPVTIVLSQMGWVRSAKGHDIDAPGLNYKAGDSFKAAVKGKSN |
| SRR7879556 | REREEAKAMSEHDMLPSEPVTIVLSQMGWVRSAKGHDIDAPGLNYKAGDSFKAAVKGKSN |
| SRR8526100 | REREEAKAMSEHDMLPSEPVTIVLSQMGWVRSAKGHDIDAPGLNYKAGDSFKAAVKGKSN |
| SRR8553991 | REREEAKAMSEHDMLPSEPVTIVLSQMGWVRSAKGHDIDAPGLNYKAGDSFKAAVKGKSN |
| SRR7842487 | REREEAKAMSEHDMLPSEPVTIVLSQMGWVRSAKGHDIDAPGLNYKAGDSFKAAVKGKSN |
| SRR8054524 | REREEAKAMSEHDMLPSEPVTIVLSQMGWVRSAKGHDIDAPGLNYKAGDSFKAAVKGKSN |
| SRR8054525 | REREEAKAMSEHDMLPSEPVTIVLSQMGWVRSAKGHDIDAPGLNYKAGDSFKAAVKGKSN |
| SRR8524733 | REREEAKAMSEHDMLPSEPVTIVLSQMGWVRSAKGHDIDAPGLNYKAGDSFKAAVKGKSN |
| SRR4093291 | REREEAKAMSEHDMLPSEPVTIVLSQMGWVRSAKGHDIDAPGLNYKAGDSFKAAVKGKSN |
| SRR4245549 | REREEAKAMSEHDMLPSEPVTIVLSQMGWVRSAKGHDIDAPGLNYKAGDSFKAAVKGKSN |
| SRR3057154 | REREEAKAMSEHDMLPSEPVTIVLSQMGWVRSAKGHDIDAPGLNYKAGDSFKAAVKGKSN |
| SRR1726150 | REREEAKAMSEHDMLPSEPVTIVLSQMGWVRSAKGHDIDAPGLNYKAGDSFKAAVKGKSN |
| SRR1996141 | REREEAKAMSEHDMLPSEPVTIVLSQMGWVRSAKGHDIDAPGLNYKAGDSFKAAVKGKSN |
| SRR1107842 | REREEAKAMSEHDMLPSEPVTIVLSQMGWVRSAKGHDIDAPGLNYKAGDSFKAAVKGKSN |
| SRR1157587 | REREEAKAMSEHDMLPSEPVTIVLSQMGWVRSAKGHDIDAPGLNYKAGDSFKAAVKGKSN |
| SRR3027706 | REREEAKAMSEHDMLPSEPVTIVLSQMGWVRSAKGHDIDAPGLNYKAGDSFKAAVKGKSN |
| SRR3027707 | REREEAKAMSEHDMLPSEPVTIVLSQMGWVRSAKGHDIDAPGLNYKAGDSFKAAVKGKSN |
| SRR3027708 | REREEAKAMSEHDMLPSEPVTIVLSQMGWVRSAKGHDIDAPGLNYKAGDSFKAAVKGKSN |
| SRR3027710 | REREEAKAMSEHDMLPSEPVTIVLSQMGWVRSAKGHDIDAPGLNYKAGDSFKAAVKGKSN |
| SRR3027711 | REREEAKAMSEHDMLPSEPVTIVLSQMGWVRSAKGHDIDAPGLNYKAGDSFKAAVKGKSN |
| SRR3027716 | REREEAKAMSEHDMLPSEPVTIVLSQMGWVRSAKGHDIDAPGLNYKAGDSFKAAVKGKSN |
| SRR3027717 | REREEAKAMSEHDMLPSEPVTIVLSQMGWVRSAKGHDIDAPGLNYKAGDSFKAAVKGKSN |
| SRR3027719 | REREEAKAMSEHDMLPSEPVTIVLSQMGWVRSAKGHDIDAPGLNYKAGDSFKAAVKGKSN |
| SRR3027721 | REREEAKAMSEHDMLPSEPVTIVLSQMGWVRSAKGHDIDAPGLNYKAGDSFKAAVKGKSN |
| SRR3027723 | REREEAKAMSEHDMLPSEPVTIVLSQMGWVRSAKGHDIDAPGLNYKAGDSFKAAVKGKSN |
| SRR3115978 | REREEAKAMSEHDMLPSEPVTIVLSQMGWVRSAKGHDIDAPGLNYKAGDSFKAAVKGKSN |
| SRR2534093 | REREEAKAMSEHDMLPSEPVTIVLSQMGWVRSAKGHDIDAPGLNYKAGDSFKAAVKGKSN |
| SRR2534094 | REREEAKAMSEHDMLPSEPVTIVLSQMGWVRSAKGHDIDAPGLNYKAGDSFKAAVKGKSN |
| SRR2534095 | REREEAKAMSEHDMLPSEPVTIVLSQMGWVRSAKGHDIDAPGLNYKAGDSFKAAVKGKSN |
| SRR2534108 | REREEAKAMSEHDMLPSEPVTIVLSQMGWVRSAKGHDIDAPGLNYKAGDSFKAAVKGKSN |
| SRR1106464 | REREEAKAMSEHDMLPSEPVTIVLSQMGWVRSAKGHDIDAPGLNYKAGDSFKAAVKGKSN |
| SRR1106463 | REREEAKAMSEHDMLPSEPVTIVLSQMGWVRSAKGHDIDAPGLNYKAGDSFKAAVKGKSN |
| SRR6949610 | REREEAKAMSEHDMLPSEPVTIVLSQMGWVRSAKGHDIDAPGLNYKAGDSFKAAVKGKSN |
| SRR6950452 | REREEAKAMSEHDMLPSEPVTIVLSQMGWVRSAKGHDIDAPGLNYKAGDSFKAAVKGKSN |
| ERR2019831 | REREEAKAMSEHDMLPSEPVTIVLSQMGWVRSAKGHDIDAPGLNYKAGDSFKAAVKGKSN |
| SRR2085693 | REREEAKAMSEHDMLPSEPVTIVLSQMGWVRSAKGHDIDAPGLNYKAGDSFKAAVKGKSN |
| SRR2086898 | REREEAKAMSEHDMLPSEPVTIVLSQMGWVRSAKGHDIDAPGLNYKAGDSFKAAVKGKSN |
| SRR2175312 | REREEAKAMSEHDMLPSEPVTIVLSQMGWVRSAKGHDIDAPGLNYKAGDSFKAAVKGKSN |
| SRR2175360 | REREEAKAMSEHDMLPSEPVTIVLSQMGWVRSAKGHDIDAPGLNYKAGDSFKAAVKGKSN |
| SRR5231997 | REREEAKAMSEHDMLPSEPVTIVLSQMGWVRSAKGHDIDAPGLNYKAGDSFKAAVKGKSN |
| SRR5232003 | REREEAKAMSEHDMLPSEPVTIVLSQMGWVRSAKGHDIDAPGLNYKAGDSFKAAVKGKSN |
| SRR5232015 | REREEAKAMSEHDMLPSEPVTIVLSQMGWVRSAKGHDIDAPGLNYKAGDSFKAAVKGKSN |
| SRR949434 | REREEAKAMSEHDMLPSEPVTIVLSQMGWVRSAKGHDIDAPGLNYKAGDSFKAAVKGKSN |
| SRR3216575 | REREEAKAMSEHDMLPSEPVTIVLSQMGWVRSAKGHDIDAPGLNYKAGDSFKAAVKGKSN |
| SRR5205342 | REREEAKAMSEHDMLPSEPVTIVLSQMGWVRSAKGHDIDAPGLNYKAGDSFKAAVKGKSN |
| SRR1501669 | REREEAKAMSEHDMLPSEPVTIVLSQMGWVRSAKGHDIDAPGLNYKAGDSFKAAVKGKSN |
| SRR5209740 | REREEAKAMSEHDMLPSEPVTIVLSQMGWVRSAKGHDIDAPGLNYKAGDSFKAAVKGKSN |
| SRR3240355 | REREEAKAMSEHDMLPSEPVTIVLSQMGWVRSAKGHDIDAPGLNYKAGDSFKAAVKGKSN |
| SRR3392777 | REREEAKAMSEHDMLPSEPVTIVLSQMGWVRSAKGHDIDAPGLNYKAGDSFKAAVKGKSN |
| SRR3593671 | REREEAKAMSEHDMLPSEPVTIVLSQMGWVRSAKGHDIDAPGLNYKAGDSFKAAVKGKSN |
| SRR5413290 | REREEAKAMSEHDMLPSEPVTIVLSQMGWVRSAKGHDIDAPGLNYKAGDSFKAAVKGKSN |
| SRR5590269 | REREEAKAMSEHDMLPSEPVTIVLSQMGWVRSAKGHDIDAPGLNYKAGDSFKAAVKGKSN |
| SRR5812103 | REREEAKAMSEHDMLPSEPVTIVLSQMGWVRSAKGHDIDAPGLNYKAGDSFKAAVKGKSN |
| SRR2830941 | REREEAKAMSEHDMLPSEPVTIVLSQMGWVRSAKGHDIDAPGLNYKAGDSFKAAVKGKSN |
| SRR2830966 | REREEAKAMSEHDMLPSEPVTIVLSQMGWVRSAKGHDIDAPGLNYKAGDSFKAAVKGKSN |
| SRR3137270 | REREEAKAMSEHDMLPSEPVTIVLSQMGWVRSAKGHDIDAPGLNYKAGDSFKAAVKGKSN |
| SRR3137271 | REREEAKAMSEHDMLPSEPVTIVLSQMGWVRSAKGHDIDAPGLNYKAGDSFKAAVKGKSN |
| ERR526807 | REREEAKAMSEHDMLPSEPVTIVLSQMGWVRSAKGHDIDAPGLNYKAGDSFKAAVKGKSN |
| ERR2197922 | REREEAKAMSEHDMLPSEPVTIVLSQMGWVRSAKGHDIDAPGLNYKAGDSFKAAVKGKSN |
| ERR2197923 | REREEAKAMSEHDMLPSEPVTIVLSQMGWVRSAKGHDIDAPGLNYKAGDSFKAAVKGKSN |
| ERR2197924 | REREEAKAMSEHDMLPSEPVTIVLSQMGWVRSAKGHDIDAPGLNYKAGDSFKAAVKGKSN |
| ERR2197925 | REREEAKAMSEHDMLPSEPVTIVLSQMGWVRSAKGHDIDAPGLNYKAGDSFKAAVKGKSN |
| ERR2197927 | REREEAKAMSEHDMLPSEPVTIVLSQMGWVRSAKGHDIDAPGLNYKAGDSFKAAVKGKSN |
| ERR2197929 | REREEAKAMSEHDMLPSEPVTIVLSQMGWVRSAKGHDIDAPGLNYKAGDSFKAAVKGKSN |
| SRR1648149 | REREEAKAMSEHDMLPSEPVTIVLSQMGWVRSAKGHDIDAPGLNYKAGDSFKAAVKGKSN |
| SRR1048299 | REREEAKAMSEHDMLPSEPVTIVLSQMGWVRSAKGHDIDAPGLNYKAGDSFKAAVKGKSN |
| SRR1300677 | REREEAKAMSEHDMLPSEPVTIVLSQMGWVRSAKGHDIDAPGLNYKAGDSFKAAVKGKSN |
| SRR1288356 | REREEAKAMSEHDMLPSEPVTIVLSQMGWVRSAKGHDIDAPGLNYKAGDSFKAAVKGKSN |
| SRR7426190 | REREEAKAMSEHDMLPSEPVTIVLSQMGWVRSAKGHDIDAPGLNYKAGDSFKAAVKGKSN |
| SRR7426192 | REREEAKAMSEHDMLPSEPVTIVLSQMGWVRSAKGHDIDAPGLNYKAGDSFKAAVKGKSN |
| SRR7426193 | REREEAKAMSEHDMLPSEPVTIVLSQMGWVRSAKGHDIDAPGLNYKAGDSFKAAVKGKSN |
| SRR7441832 | REREEAKAMSEHDMLPSEPVTIVLSQMGWVRSAKGHDIDAPGLNYKAGDSFKAAVKGKSN |
| SRR7426179 | REREEAKAMSEHDMLPSEPVTIVLSQMGWVRSAKGHDIDAPGLNYKAGDSFKAAVKGKSN |
| SRR7439238 | REREEAKAMSEHDMLPSEPVTIVLSQMGWVRSAKGHDIDAPGLNYKAGDSFKAAVKGKSN |
| SRR7439244 | REREEAKAMSEHDMLPSEPVTIVLSQMGWVRSAKGHDIDAPGLNYKAGDSFKAAVKGKSN |
| SRR7439259 | REREEAKAMSEHDMLPSEPVTIVLSQMGWVRSAKGHDIDAPGLNYKAGDSFKAAVKGKSN |
| SRR7439260 | REREEAKAMSEHDMLPSEPVTIVLSQMGWVRSAKGHDIDAPGLNYKAGDSFKAAVKGKSN |
| SRR7441786 | REREEAKAMSEHDMLPSEPVTIVLSQMGWVRSAKGHDIDAPGLNYKAGDSFKAAVKGKSN |
| SRR7441797 | REREEAKAMSEHDMLPSEPVTIVLSQMGWVRSAKGHDIDAPGLNYKAGDSFKAAVKGKSN |
| ERR1759093 | REREEAKAMSEHDMLPSEPVTIVLSQMGWVRSAKGHDIDAPGLNYKAGDSFKAAVKGKSN |
| ERR2580275 | REREEAKAMSEHDMLPSEPVTIVLSQMGWVRSAKGHDIDAPGLNYKAGDSFKAAVKGKSN |
| ERR1759204 | REREEAKAMSEHDMLPSEPVTIVLSQMGWVRSAKGHDIDAPGLNYKAGDSFKAAVKGKSN |
| SRR1300699 | REREEAKAMSEHDMLPSEPVTIVLSQMGWVRSAKGHDIDAPGLNYKAGDSFKAAVKGKSN |
| S_0825_17 | REREEAKAMSEHDMLPSEPVTIVLSQMGWVRSAKGHDIDAPGLNYKAGDSFKAAVKGKSN |
| SRR1958215 | REREEAKAMSEHDMLPSEPVTIVLSQMGWVRSAKGHDIDAPGLNYKAGDSFKAAVKGKSN |
| SRR1958540 | REREEAKAMSEHDMLPSEPVTIVLSQMGWVRSAKGHDIDAPGLNYKAGDSFKAAVKGKSN |
| SRR1958636 | REREEAKAMSEHDMLPSEPVTIVLSQMGWVRSAKGHDIDAPGLNYKAGDSFKAAVKGKSN |
| SRR1959422 | REREEAKAMSEHDMLPSEPVTIVLSQMGWVRSAKGHDIDAPGLNYKAGDSFKAAVKGKSN |
| SRR1959427 | REREEAKAMSEHDMLPSEPVTIVLSQMGWVRSAKGHDIDAPGLNYKAGDSFKAAVKGKSN |
| SRR1960226 | REREEAKAMSEHDMLPSEPVTIVLSQMGWVRSAKGHDIDAPGLNYKAGDSFKAAVKGKSN |
| SRR1963498 | REREEAKAMSEHDMLPSEPVTIVLSQMGWVRSAKGHDIDAPGLNYKAGDSFKAAVKGKSN |
| SRR1965947 | REREEAKAMSEHDMLPSEPVTIVLSQMGWVRSAKGHDIDAPGLNYKAGDSFKAAVKGKSN |
| SRR1966125 | REREEAKAMSEHDMLPSEPVTIVLSQMGWVRSAKGHDIDAPGLNYKAGDSFKAAVKGKSN |
| SRR1966330 | REREEAKAMSEHDMLPSEPVTIVLSQMGWVRSAKGHDIDAPGLNYKAGDSFKAAVKGKSN |
| SRR1966565 | REREEAKAMSEHDMLPSEPVTIVLSQMGWVRSAKGHDIDAPGLNYKAGDSFKAAVKGKSN |
| SRR1966864 | REREEAKAMSEHDMLPSEPVTIVLSQMGWVRSAKGHDIDAPGLNYKAGDSFKAAVKGKSN |
| SRR1966989 | REREEAKAMSEHDMLPSEPVTIVLSQMGWVRSAKGHDIDAPGLNYKAGDSFKAAVKGKSN |
| SRR1967688 | REREEAKAMSEHDMLPSEPVTIVLSQMGWVRSAKGHDIDAPGLNYKAGDSFKAAVKGKSN |
| SRR1967733 | REREEAKAMSEHDMLPSEPVTIVLSQMGWVRSAKGHDIDAPGLNYKAGDSFKAAVKGKSN |
| SRR1967746 | REREEAKAMSEHDMLPSEPVTIVLSQMGWVRSAKGHDIDAPGLNYKAGDSFKAAVKGKSN |
| SRR1968341 | REREEAKAMSEHDMLPSEPVTIVLSQMGWVRSAKGHDIDAPGLNYKAGDSFKAAVKGKSN |
| SRR1968456 | REREEAKAMSEHDMLPSEPVTIVLSQMGWVRSAKGHDIDAPGLNYKAGDSFKAAVKGKSN |
| SRR1968465 | REREEAKAMSEHDMLPSEPVTIVLSQMGWVRSAKGHDIDAPGLNYKAGDSFKAAVKGKSN |
| SRR1968761 | REREEAKAMSEHDMLPSEPVTIVLSQMGWVRSAKGHDIDAPGLNYKAGDSFKAAVKGKSN |
| SRR1969047 | REREEAKAMSEHDMLPSEPVTIVLSQMGWVRSAKGHDIDAPGLNYKAGDSFKAAVKGKSN |
| SRR1969255 | REREEAKAMSEHDMLPSEPVTIVLSQMGWVRSAKGHDIDAPGLNYKAGDSFKAAVKGKSN |
| SRR1969412 | REREEAKAMSEHDMLPSEPVTIVLSQMGWVRSAKGHDIDAPGLNYKAGDSFKAAVKGKSN |
| SRR1969524 | REREEAKAMSEHDMLPSEPVTIVLSQMGWVRSAKGHDIDAPGLNYKAGDSFKAAVKGKSN |
| SRR1969584 | REREEAKAMSEHDMLPSEPVTIVLSQMGWVRSAKGHDIDAPGLNYKAGDSFKAAVKGKSN |
| SRR1969648 | REREEAKAMSEHDMLPSEPVTIVLSQMGWVRSAKGHDIDAPGLNYKAGDSFKAAVKGKSN |
| SRR1969804 | REREEAKAMSEHDMLPSEPVTIVLSQMGWVRSAKGHDIDAPGLNYKAGDSFKAAVKGKSN |
| SRR1970221 | REREEAKAMSEHDMLPSEPVTIVLSQMGWVRSAKGHDIDAPGLNYKAGDSFKAAVKGKSN |
| SRR1970268 | REREEAKAMSEHDMLPSEPVTIVLSQMGWVRSAKGHDIDAPGLNYKAGDSFKAAVKGKSN |
| SRR1965862 | REREEAKAMSEHDMLPSEPVTIVLSQMGWVRSAKGHDIDAPGLNYKAGDSFKAAVKGKSN |
| SRR1967363 | REREEAKAMSEHDMLPSEPVTIVLSQMGWVRSAKGHDIDAPGLNYKAGDSFKAAVKGKSN |
| SRR1968276 | REREEAKAMSEHDMLPSEPVTIVLSQMGWVRSAKGHDIDAPGLNYKAGDSFKAAVKGKSN |
| SRR1968967 | REREEAKAMSEHDMLPSEPVTIVLSQMGWVRSAKGHDIDAPGLNYKAGDSFKAAVKGKSN |
| SRR3321531 | REREEAKAMSEHDMLPSEPVTIVLSQMGWVRSAKGHDIDAPGLNYKAGDSFKAAVKGKSN |
| SRR3321883 | REREEAKAMSEHDMLPSEPVTIVLSQMGWVRSAKGHDIDAPGLNYKAGDSFKAAVKGKSN |
| SRR3322413 | REREEAKAMSEHDMLPSEPVTIVLSQMGWVRSAKGHDIDAPGLNYKAGDSFKAAVKGKSN |
| SRR3323012 | REREEAKAMSEHDMLPSEPVTIVLSQMGWVRSAKGHDIDAPGLNYKAGDSFKAAVKGKSN |
| SRR5194289 | REREEAKAMSEHDMLPSEPVTIVLSQMGWVRSAKGHDIDAPGLNYKAGDSFKAAVKGKSN |
| SRR7163798 | REREEAKAMSEHDMLPSEPVTIVLSQMGWVRSAKGHDIDAPGLNYKAGDSFKAAVKGKSN |
| SRR7172610 | REREEAKAMSEHDMLPSEPVTIVLSQMGWVRSAKGHDIDAPGLNYKAGDSFKAAVKGKSN |
| SRR7204568 | REREEAKAMSEHDMLPSEPVTIVLSQMGWVRSAKGHDIDAPGLNYKAGDSFKAAVKGKSN |
| SRR7223230 | REREEAKAMSEHDMLPSEPVTIVLSQMGWVRSAKGHDIDAPGLNYKAGDSFKAAVKGKSN |
| SRR7230675 | REREEAKAMSEHDMLPSEPVTIVLSQMGWVRSAKGHDIDAPGLNYKAGDSFKAAVKGKSN |
| SRR7278056 | REREEAKAMSEHDMLPSEPVTIVLSQMGWVRSAKGHDIDAPGLNYKAGDSFKAAVKGKSN |
| SRR7278086 | REREEAKAMSEHDMLPSEPVTIVLSQMGWVRSAKGHDIDAPGLNYKAGDSFKAAVKGKSN |
| SRR7285841 | REREEAKAMSEHDMLPSEPVTIVLSQMGWVRSAKGHDIDAPGLNYKAGDSFKAAVKGKSN |
| SRR7292625 | REREEAKAMSEHDMLPSEPVTIVLSQMGWVRSAKGHDIDAPGLNYKAGDSFKAAVKGKSN |
| SRR7292665 | REREEAKAMSEHDMLPSEPVTIVLSQMGWVRSAKGHDIDAPGLNYKAGDSFKAAVKGKSN |
| SRR7297965 | REREEAKAMSEHDMLPSEPVTIVLSQMGWVRSAKGHDIDAPGLNYKAGDSFKAAVKGKSN |
| SRR7350726 | REREEAKAMSEHDMLPSEPVTIVLSQMGWVRSAKGHDIDAPGLNYKAGDSFKAAVKGKSN |
| SRR7410328 | REREEAKAMSEHDMLPSEPVTIVLSQMGWVRSAKGHDIDAPGLNYKAGDSFKAAVKGKSN |
| SRR7474665 | REREEAKAMSEHDMLPSEPVTIVLSQMGWVRSAKGHDIDAPGLNYKAGDSFKAAVKGKSN |
| SRR7523184 | REREEAKAMSEHDMLPSEPVTIVLSQMGWVRSAKGHDIDAPGLNYKAGDSFKAAVKGKSN |
| SRR7187264 | REREEAKAMSEHDMLPSEPVTIVLSQMGWVRSAKGHDIDAPGLNYKAGDSFKAAVKGKSN |
| SRR7204445 | REREEAKAMSEHDMLPSEPVTIVLSQMGWVRSAKGHDIDAPGLNYKAGDSFKAAVKGKSN |
| SRR7285641 | REREEAKAMSEHDMLPSEPVTIVLSQMGWVRSAKGHDIDAPGLNYKAGDSFKAAVKGKSN |
| SRR7286695 | REREEAKAMSEHDMLPSEPVTIVLSQMGWVRSAKGHDIDAPGLNYKAGDSFKAAVKGKSN |
| SRR7286705 | REREEAKAMSEHDMLPSEPVTIVLSQMGWVRSAKGHDIDAPGLNYKAGDSFKAAVKGKSN |
| SRR7292931 | REREEAKAMSEHDMLPSEPVTIVLSQMGWVRSAKGHDIDAPGLNYKAGDSFKAAVKGKSN |
| SRR7310349 | REREEAKAMSEHDMLPSEPVTIVLSQMGWVRSAKGHDIDAPGLNYKAGDSFKAAVKGKSN |
| SRR7351616 | REREEAKAMSEHDMLPSEPVTIVLSQMGWVRSAKGHDIDAPGLNYKAGDSFKAAVKGKSN |
| SRR7414818 | REREEAKAMSEHDMLPSEPVTIVLSQMGWVRSAKGHDIDAPGLNYKAGDSFKAAVKGKSN |
| SRR7426480 | REREEAKAMSEHDMLPSEPVTIVLSQMGWVRSAKGHDIDAPGLNYKAGDSFKAAVKGKSN |
| SRR5584105 | REREEAKAMSEHDMLPSEPVTIVLSQMGWVRSAKGHDIDAPGLNYKAGDSFKAAVKGKSN |
| SRR5584565 | REREEAKAMSEHDMLPSEPVTIVLSQMGWVRSAKGHDIDAPGLNYKAGDSFKAAVKGKSN |
| SRR5584614 | REREEAKAMSEHDMLPSEPVTIVLSQMGWVRSAKGHDIDAPGLNYKAGDSFKAAVKGKSN |
| SRR5631543 | REREEAKAMSEHDMLPSEPVTIVLSQMGWVRSAKGHDIDAPGLNYKAGDSFKAAVKGKSN |
| SRR5631553 | REREEAKAMSEHDMLPSEPVTIVLSQMGWVRSAKGHDIDAPGLNYKAGDSFKAAVKGKSN |
| SRR7123196 | REREEAKAMSEHDMLPSEPVTIVLSQMGWVRSAKGHDIDAPGLNYKAGDSFKAAVKGKSN |
| SRR7163819 | REREEAKAMSEHDMLPSEPVTIVLSQMGWVRSAKGHDIDAPGLNYKAGDSFKAAVKGKSN |
| SRR7163920 | REREEAKAMSEHDMLPSEPVTIVLSQMGWVRSAKGHDIDAPGLNYKAGDSFKAAVKGKSN |
| SRR7209528 | REREEAKAMSEHDMLPSEPVTIVLSQMGWVRSAKGHDIDAPGLNYKAGDSFKAAVKGKSN |
| SRR7249868 | REREEAKAMSEHDMLPSEPVTIVLSQMGWVRSAKGHDIDAPGLNYKAGDSFKAAVKGKSN |
| SRR7278088 | REREEAKAMSEHDMLPSEPVTIVLSQMGWVRSAKGHDIDAPGLNYKAGDSFKAAVKGKSN |
| SRR7285788 | REREEAKAMSEHDMLPSEPVTIVLSQMGWVRSAKGHDIDAPGLNYKAGDSFKAAVKGKSN |
| SRR7286789 | REREEAKAMSEHDMLPSEPVTIVLSQMGWVRSAKGHDIDAPGLNYKAGDSFKAAVKGKSN |
| SRR7286886 | REREEAKAMSEHDMLPSEPVTIVLSQMGWVRSAKGHDIDAPGLNYKAGDSFKAAVKGKSN |
| SRR7310632 | REREEAKAMSEHDMLPSEPVTIVLSQMGWVRSAKGHDIDAPGLNYKAGDSFKAAVKGKSN |
| SRR7350631 | REREEAKAMSEHDMLPSEPVTIVLSQMGWVRSAKGHDIDAPGLNYKAGDSFKAAVKGKSN |
| SRR7458741 | REREEAKAMSEHDMLPSEPVTIVLSQMGWVRSAKGHDIDAPGLNYKAGDSFKAAVKGKSN |
| SRR7480280 | REREEAKAMSEHDMLPSEPVTIVLSQMGWVRSAKGHDIDAPGLNYKAGDSFKAAVKGKSN |
| SRR7523660 | REREEAKAMSEHDMLPSEPVTIVLSQMGWVRSAKGHDIDAPGLNYKAGDSFKAAVKGKSN |
| SRR7523775 | REREEAKAMSEHDMLPSEPVTIVLSQMGWVRSAKGHDIDAPGLNYKAGDSFKAAVKGKSN |
| SRR7251101 | REREEAKAMSEHDMLPSEPVTIVLSQMGWVRSAKGHDIDAPGLNYKAGDSFKAAVKGKSN |
| SRR7284299 | REREEAKAMSEHDMLPSEPVTIVLSQMGWVRSAKGHDIDAPGLNYKAGDSFKAAVKGKSN |
| SRR7285738 | REREEAKAMSEHDMLPSEPVTIVLSQMGWVRSAKGHDIDAPGLNYKAGDSFKAAVKGKSN |
| SRR7310640 | REREEAKAMSEHDMLPSEPVTIVLSQMGWVRSAKGHDIDAPGLNYKAGDSFKAAVKGKSN |
| SRR7349159 | REREEAKAMSEHDMLPSEPVTIVLSQMGWVRSAKGHDIDAPGLNYKAGDSFKAAVKGKSN |
| SRR7474873 | REREEAKAMSEHDMLPSEPVTIVLSQMGWVRSAKGHDIDAPGLNYKAGDSFKAAVKGKSN |
| SRR7495689 | REREEAKAMSEHDMLPSEPVTIVLSQMGWVRSAKGHDIDAPGLNYKAGDSFKAAVKGKSN |
| SRR7495752 | REREEAKAMSEHDMLPSEPVTIVLSQMGWVRSAKGHDIDAPGLNYKAGDSFKAAVKGKSN |
| ------------------------------------------------------------------------------ | |
| S16BD08730 | QPVVFIDTTGRSYAIDPITLPSARGQGEPLTGKLTLPPGATVEHMLMEGDDQKLLMASDA |
| S18BD00684 | QPVVFIDTTGRSYAIDPITLPSARGQGEPLTGKLTLPPGATVEHMLMEGDDQKLLMASDA |
| S18BD03994 | QPVVFIDTTGRSYAIDPITLPSARGQGEPLTGKLTLPPGATVEHMLMEGDDQKLLMASDA |
| S18BD05011 | QPVVFIDTTGRSYAIDPITLPSARGQGEPLTGKLTLPPGATVEHMLMEGDDQKLLMASDA |
| RKI_16-03723 | QPVVFIDTTGRSYAIDPITLPSARGQGEPLTGKLTLPPGATVEHMLMEGDDQKLLMASDA |
| RKI_16-04315 | QPVVFIDTTGRSYAIDPITLPSARGQGEPLTGKLTLPPGATVEHMLMEGDDQKLLMASDA |
| RKI_17-02304 | QPVVFIDTTGRSYAIDPITLPSARGQGEPLTGKLTLPPGATVEHMLMEGDDQKLLMASDA |
| RKI_17-02411 | QPVVFIDTTGRSYAIDPITLPSARGQGEPLTGKLTLPPGATVEHMLMEGDDQKLLMASDA |
| RKI_17-02757 | QPVVFIDTTGRSYAIDPITLPSARGQGEPLTGKLTLPPGATVEHMLMEGDDQKLLMASDA |
| RKI_17-04797 | QPVVFIDTTGRSYAIDPITLPSARGQGEPLTGKLTLPPGATVEHMLMEGDDQKLLMASDA |
| RKI_17-06869 | QPVVFIDTTGRSYAIDPITLPSARGQGEPLTGKLTLPPGATVEHMLMEGDDQKLLMASDA |
| ERR2580277 | QPVVFIDTTGRSYAIDPITLPSARGQGEPLTGKLTLPPGATVEHMLMEGDDQKLLMASDA |
| ERR2580276 | QPVVFIDTTGRSYAIDPITLPSARGQGEPLTGKLTLPPGATVEHMLMEGDDQKLLMASDA |
| ERR2580273 | QPVVFIDTTGRSYAIDPITLPSARGQGEPLTGKLTLPPGATVEHMLMEGDDQKLLMASDA |
| ERR2580274 | QPVVFIDTTGRSYAIDPITLPSARGQGEPLTGKLTLPPGATVEHMLMEGDDQKLLMASDA |
| ERR2173656 | QPVVFIDTTGRSYAIDPITLPSARGQGEPLTGKLTLPPGATVEHMLMEGDDQKLLMASDA |
| 17041676 | QPVVFIDTTGRSYAIDPITLPSARGQGEPLTGKLTLPPGATVEHMLMEGDDQKLLMASDA |
| MT16-000061 | QPVVFIDTTGRSYAIDPITLPSARGQGEPLTGKLTLPPGATVEHMLMEGDDQKLLMASDA |
| MT16-019416 | QPVVFIDTTGRSYAIDPITLPSARGQGEPLTGKLTLPPGATVEHMLMEGDDQKLLMASDA |
| MT16-027865 | QPVVFIDTTGRSYAIDPITLPSARGQGEPLTGKLTLPPGATVEHMLMEGDDQKLLMASDA |
| MT16-031693 | QPVVFIDTTGRSYAIDPITLPSARGQGEPLTGKLTLPPGATVEHMLMEGDDQKLLMASDA |
| MT16-040253 | QPVVFIDTTGRSYAIDPITLPSARGQGEPLTGKLTLPPGATVEHMLMEGDDQKLLMASDA |
| MT16-045379 | QPVVFIDTTGRSYAIDPITLPSARGQGEPLTGKLTLPPGATVEHMLMEGDDQKLLMASDA |
| MT16-442728 | QPVVFIDTTGRSYAIDPITLPSARGQGEPLTGKLTLPPGATVEHMLMEGDDQKLLMASDA |
| MT16-462857 | QPVVFIDTTGRSYAIDPITLPSARGQGEPLTGKLTLPPGATVEHMLMEGDDQKLLMASDA |
| MT16-480196 | QPVVFIDTTGRSYAIDPITLPSARGQGEPLTGKLTLPPGATVEHMLMEGDDQKLLMASDA |
| MT16-861555 | QPVVFIDTTGRSYAIDPITLPSARGQGEPLTGKLTLPPGATVEHMLMEGDDQKLLMASDA |
| MT17-076833 | QPVVFIDTTGRSYAIDPITLPSARGQGEPLTGKLTLPPGATVEHMLMEGDDQKLLMASDA |
| MT17-110677 | QPVVFIDTTGRSYAIDPITLPSARGQGEPLTGKLTLPPGATVEHMLMEGDDQKLLMASDA |
| MT17-131730 | QPVVFIDTTGRSYAIDPITLPSARGQGEPLTGKLTLPPGATVEHMLMEGDDQKLLMASDA |
| MT17-140890 | QPVVFIDTTGRSYAIDPITLPSARGQGEPLTGKLTLPPGATVEHMLMEGDDQKLLMASDA |
| MT17-141840 | QPVVFIDTTGRSYAIDPITLPSARGQGEPLTGKLTLPPGATVEHMLMEGDDQKLLMASDA |
| MT17-152488 | QPVVFIDTTGRSYAIDPITLPSARGQGEPLTGKLTLPPGATVEHMLMEGDDQKLLMASDA |
| MT17-157311 | QPVVFIDTTGRSYAIDPITLPSARGQGEPLTGKLTLPPGATVEHMLMEGDDQKLLMASDA |
| MT17-161645 | QPVVFIDTTGRSYAIDPITLPSARGQGEPLTGKLTLPPGATVEHMLMEGDDQKLLMASDA |
| MT17-167951 | QPVVFIDTTGRSYAIDPITLPSARGQGEPLTGKLTLPPGATVEHMLMEGDDQKLLMASDA |
| MT18-217732 | QPVVFIDTTGRSYAIDPITLPSARGQGEPLTGKLTLPPGATVEHMLMEGDDQKLLMASDA |
| MT18-252580 | QPVVFIDTTGRSYAIDPITLPSARGQGEPLTGKLTLPPGATVEHMLMEGDDQKLLMASDA |
| RIVM_H_2009-01 | QPVVFIDTTGRSYAIDPITLPSARGQGEPLTGKLTLPPGATVEHMLMEGDDQKLLMASDA |
| RIVM_H_2010-01 | QPVVFIDTTGRSYAIDPITLPSARGQGEPLTGKLTLPPGATVEHMLMEGDDQKLLMASDA |
| RIVM_H_2010-02 | QPVVFIDTTGRSYAIDPITLPSARGQGEPLTGKLTLPPGATVEHMLMEGDDQKLLMASDA |
| RIVM_H_2011-01 | QPVVFIDTTGRSYAIDPITLPSARGQGEPLTGKLTLPPGATVEHMLMEGDDQKLLMASDA |
| RIVM_H_2011-02 | QPVVFIDTTGRSYAIDPITLPSARGQGEPLTGKLTLPPGATVEHMLMEGDDQKLLMASDA |
| RIVM_H_2011-03 | QPVVFIDTTGRSYAIDPITLPSARGQGEPLTGKLTLPPGATVEHMLMEGDDQKLLMASDA |
| RIVM_H_2013-01 | QPVVFIDTTGRSYAIDPITLPSARGQGEPLTGKLTLPPGATVEHMLMEGDDQKLLMASDA |
| RIVM_H_2013-02 | QPVVFIDTTGRSYAIDPITLPSARGQGEPLTGKLTLPPGATVEHMLMEGDDQKLLMASDA |
| RIVM_H_2014-01 | QPVVFIDTTGRSYAIDPITLPSARGQGEPLTGKLTLPPGATVEHMLMEGDDQKLLMASDA |
| RIVM_H_2014-02 | QPVVFIDTTGRSYAIDPITLPSARGQGEPLTGKLTLPPGATVEHMLMEGDDQKLLMASDA |
| RIVM_H_2016-01 | QPVVFIDTTGRSYAIDPITLPSARGQGEPLTGKLTLPPGATVEHMLMEGDDQKLLMASDA |
| RIVM_H_2016-02 | QPVVFIDTTGRSYAIDPITLPSARGQGEPLTGKLTLPPGATVEHMLMEGDDQKLLMASDA |
| RIVM_H_2016-03 | QPVVFIDTTGRSYAIDPITLPSARGQGEPLTGKLTLPPGATVEHMLMEGDDQKLLMASDA |
| RIVM_H_2016-04 | QPVVFIDTTGRSYAIDPITLPSARGQGEPLTGKLTLPPGATVEHMLMEGDDQKLLMASDA |
| RIVM_H_2016-05 | QPVVFIDTTGRSYAIDPITLPSARGQGEPLTGKLTLPPGATVEHMLMEGDDQKLLMASDA |
| RIVM_H_2016-06 | QPVVFIDTTGRSYAIDPITLPSARGQGEPLTGKLTLPPGATVEHMLMEGDDQKLLMASDA |
| RIVM_H_2016-07 | QPVVFIDTTGRSYAIDPITLPSARGQGEPLTGKLTLPPGATVEHMLMEGDDQKLLMASDA |
| RIVM_H_2016-08 | QPVVFIDTTGRSYAIDPITLPSARGQGEPLTGKLTLPPGATVEHMLMEGDDQKLLMASDA |
| RIVM_H_2016-09 | QPVVFIDTTGRSYAIDPITLPSARGQGEPLTGKLTLPPGATVEHMLMEGDDQKLLMASDA |
| RIVM_H_2016-10 | QPVVFIDTTGRSYAIDPITLPSARGQGEPLTGKLTLPPGATVEHMLMEGDDQKLLMASDA |
| RIVM_H_2016-11 | QPVVFIDTTGRSYAIDPITLPSARGQGEPLTGKLTLPPGATVEHMLMEGDDQKLLMASDA |
| RIVM_H_2016-12 | QPVVFIDTTGRSYAIDPITLPSARGQGEPLTGKLTLPPGATVEHMLMEGDDQKLLMASDA |
| RIVM_H_2016-13 | QPVVFIDTTGRSYAIDPITLPSARGQGEPLTGKLTLPPGATVEHMLMEGDDQKLLMASDA |
| RIVM_H_2016-14 | QPVVFIDTTGRSYAIDPITLPSARGQGEPLTGKLTLPPGATVEHMLMEGDDQKLLMASDA |
| RIVM_H_2016-15 | QPVVFIDTTGRSYAIDPITLPSARGQGEPLTGKLTLPPGATVEHMLMEGDDQKLLMASDA |
| RIVM_H_2017-01 | QPVVFIDTTGRSYAIDPITLPSARGQGEPLTGKLTLPPGATVEHMLMEGDDQKLLMASDA |
| RIVM_H_2017-02 | QPVVFIDTTGRSYAIDPITLPSARGQGEPLTGKLTLPPGATVEHMLMEGDDQKLLMASDA |
| RIVM_H_2017-03 | QPVVFIDTTGRSYAIDPITLPSARGQGEPLTGKLTLPPGATVEHMLMEGDDQKLLMASDA |
| RIVM_H_2017-04 | QPVVFIDTTGRSYAIDPITLPSARGQGEPLTGKLTLPPGATVEHMLMEGDDQKLLMASDA |
| RIVM_H_2017-05 | QPVVFIDTTGRSYAIDPITLPSARGQGEPLTGKLTLPPGATVEHMLMEGDDQKLLMASDA |
| RIVM_H_2017-06 | QPVVFIDTTGRSYAIDPITLPSARGQGEPLTGKLTLPPGATVEHMLMEGDDQKLLMASDA |
| RIVM_H_2017-07 | QPVVFIDTTGRSYAIDPITLPSARGQGEPLTGKLTLPPGATVEHMLMEGDDQKLLMASDA |
| RIVM_H_2017-08 | QPVVFIDTTGRSYAIDPITLPSARGQGEPLTGKLTLPPGATVEHMLMEGDDQKLLMASDA |
| RIVM_H_2017-09 | QPVVFIDTTGRSYAIDPITLPSARGQGEPLTGKLTLPPGATVEHMLMEGDDQKLLMASDA |
| RIVM_H_2017-10 | QPVVFIDTTGRSYAIDPITLPSARGQGEPLTGKLTLPPGATVEHMLMEGDDQKLLMASDA |
| RIVM_H_2017-11 | QPVVFIDTTGRSYAIDPITLPSARGQGEPLTGKLTLPPGATVEHMLMEGDDQKLLMASDA |
| RIVM_H_2017-12 | QPVVFIDTTGRSYAIDPITLPSARGQGEPLTGKLTLPPGATVEHMLMEGDDQKLLMASDA |
| RIVM_H_2017-13 | QPVVFIDTTGRSYAIDPITLPSARGQGEPLTGKLTLPPGATVEHMLMEGDDQKLLMASDA |
| RIVM_H_2017-14 | QPVVFIDTTGRSYAIDPITLPSARGQGEPLTGKLTLPPGATVEHMLMEGDDQKLLMASDA |
| RIVM_H_2017-15 | QPVVFIDTTGRSYAIDPITLPSARGQGEPLTGKLTLPPGATVEHMLMEGDDQKLLMASDA |
| RIVM_H_2017-16 | QPVVFIDTTGRSYAIDPITLPSARGQGEPLTGKLTLPPGATVEHMLMEGDDQKLLMASDA |
| RIVM_H_2017-17 | QPVVFIDTTGRSYAIDPITLPSARGQGEPLTGKLTLPPGATVEHMLMEGDDQKLLMASDA |
| RIVM_H_2017-18 | QPVVFIDTTGRSYAIDPITLPSARGQGEPLTGKLTLPPGATVEHMLMEGDDQKLLMASDA |
| RIVM_H_2017-19 | QPVVFIDTTGRSYAIDPITLPSARGQGEPLTGKLTLPPGATVEHMLMEGDDQKLLMASDA |
| 15EP001483 | QPVVFIDTTGRSYAIDPITLPSARGQGEPLTGKLTLPPGATVEHMLMEGDDQKLLMASDA |
| 17EP002363 | QPVVFIDTTGRSYAIDPITLPSARGQGEPLTGKLTLPPGATVEHMLMEGDDQKLLMASDA |
| S_0812_17 | QPVVFIDTTGRSYAIDPITLPSARGQGEPLTGKLTLPPGATVEHMLMEGDDQKLLMASDA |
| SRR1957844 | QPVVFIDTTGRSYAIDPITLPSARGQGEPLTGKLTLPPGATVEHMLMEGDDQKLLMASDA |
| SRR1958654 | QPVVFIDTTGRSYAIDPITLPSARGQGEPLTGKLTLPPGATVEHMLMEGDDQKLLMASDA |
| SRR1965077 | QPVVFIDTTGRSYAIDPITLPSARGQGEPLTGKLTLPPGATVEHMLMEGDDQKLLMASDA |
| SRR1966369 | QPVVFIDTTGRSYAIDPITLPSARGQGEPLTGKLTLPPGATVEHMLMEGDDQKLLMASDA |
| SRR1967117 | QPVVFIDTTGRSYAIDPITLPSARGQGEPLTGKLTLPPGATVEHMLMEGDDQKLLMASDA |
| SRR1967922 | QPVVFIDTTGRSYAIDPITLPSARGQGEPLTGKLTLPPGATVEHMLMEGDDQKLLMASDA |
| SRR8704720 | QPVVFIDTTGRSYAIDPITLPSARGQGEPLTGKLTLPPGATVEHMLMEGDDQKLLMASDA |
| SRR7216071 | QPVVFIDTTGRSYAIDPITLPSARGQGEPLTGKLTLPPGATVEHMLMEGDDQKLLMASDA |
| SRR7349175 | QPVVFIDTTGRSYAIDPITLPSARGQGEPLTGKLTLPPGATVEHMLMEGDDQKLLMASDA |
| SRR7523148 | QPVVFIDTTGRSYAIDPITLPSARGQGEPLTGKLTLPPGATVEHMLMEGDDQKLLMASDA |
| SRR7523854 | QPVVFIDTTGRSYAIDPITLPSARGQGEPLTGKLTLPPGATVEHMLMEGDDQKLLMASDA |
| 313865 | QPVVFIDTTGRSYAIDPITLPSARGQGEPLTGKLTLPPGATVEHMLMEGDDQKLLMASDA |
| SRR7277793 | QPVVFIDTTGRSYAIDPITLPSARGQGEPLTGKLTLPPGATVEHMLMEGDDQKLLMASDA |
| SRR7343877 | QPVVFIDTTGRSYAIDPITLPSARGQGEPLTGKLTLPPGATVEHMLMEGDDQKLLMASDA |
| SRR7351477 | QPVVFIDTTGRSYAIDPITLPSARGQGEPLTGKLTLPPGATVEHMLMEGDDQKLLMASDA |
| SRR5583183 | QPVVFIDTTGRSYAIDPITLPSARGQGEPLTGKLTLPPGATVEHMLMEGDDQKLLMASDA |
| SRR5585240 | QPVVFIDTTGRSYAIDPITLPSARGQGEPLTGKLTLPPGATVEHMLMEGDDQKLLMASDA |
| SRR7284317 | QPVVFIDTTGRSYAIDPITLPSARGQGEPLTGKLTLPPGATVEHMLMEGDDQKLLMASDA |
| SRR7299161 | QPVVFIDTTGRSYAIDPITLPSARGQGEPLTGKLTLPPGATVEHMLMEGDDQKLLMASDA |
| SRR7401730 | QPVVFIDTTGRSYAIDPITLPSARGQGEPLTGKLTLPPGATVEHMLMEGDDQKLLMASDA |
| SRR7469092 | QPVVFIDTTGRSYAIDPITLPSARGQGEPLTGKLTLPPGATVEHMLMEGDDQKLLMASDA |
| SRR7879556 | QPVVFIDTTGRSYAIDPITLPSARGQGEPLTGKLTLPPGATVEHMLMEGDDQKLLMASDA |
| SRR8526100 | QPVVFIDTTGRSYAIDPITLPSARGQGEPLTGKLTLPPGATVEHMLMEGDDQKLLMASDA |
| SRR8553991 | QPVVFIDTTGRSYAIDPITLPSARGQGEPLTGKLTLPPGATVEHMLMEGDDQKLLMASDA |
| SRR7842487 | QPVVFIDTTGRSYAIDPITLPSARGQGEPLTGKLTLPPGATVEHMLMEGDDQKLLMASDA |
| SRR8054524 | QPVVFIDTTGRSYAIDPITLPSARGQGEPLTGKLTLPPGATVEHMLMEGDDQKLLMASDA |
| SRR8054525 | QPVVFIDTTGRSYAIDPITLPSARGQGEPLTGKLTLPPGATVEHMLMEGDDQKLLMASDA |
| SRR8524733 | QPVVFIDTTGRSYAIDPITLPSARGQGEPLTGKLTLPPGATVEHMLMEGDDQKLLMASDA |
| SRR4093291 | QPVVFIDTTGRSYAIDPITLPSARGQGEPLTGKLTLPPGATVEHMLMEGDDQKLLMASDA |
| SRR4245549 | QPVVFIDTTGRSYAIDPITLPSARGQGEPLTGKLTLPPGATVEHMLMEGDDQKLLMASDA |
| SRR3057154 | QPVVFIDTTGRSYAIDPITLPSARGQGEPLTGKLTLPPGATVEHMLMEGDDQKLLMASDA |
| SRR1726150 | QPVVFIDTTGRSYAIDPITLPSARGQGEPLTGKLTLPPGATVEHMLMEGDDQKLLMASDA |
| SRR1996141 | QPVVFIDTTGRSYAIDPITLPSARGQGEPLTGKLTLPPGATVEHMLMEGDDQKLLMASDA |
| SRR1107842 | QPVVFIDTTGRSYAIDPITLPSARGQGEPLTGKLTLPPGATVEHMLMEGDDQKLLMASDA |
| SRR1157587 | QPVVFIDTTGRSYAIDPITLPSARGQGEPLTGKLTLPPGATVEHMLMEGDDQKLLMASDA |
| SRR3027706 | QPVVFIDTTGRSYAIDPITLPSARGQGEPLTGKLTLPPGATVEHMLMEGDDQKLLMASDA |
| SRR3027707 | QPVVFIDTTGRSYAIDPITLPSARGQGEPLTGKLTLPPGATVEHMLMEGDDQKLLMASDA |
| SRR3027708 | QPVVFIDTTGRSYAIDPITLPSARGQGEPLTGKLTLPPGATVEHMLMEGDDQKLLMASDA |
| SRR3027710 | QPVVFIDTTGRSYAIDPITLPSARGQGEPLTGKLTLPPGATVEHMLMEGDDQKLLMASDA |
| SRR3027711 | QPVVFIDTTGRSYAIDPITLPSARGQGEPLTGKLTLPPGATVEHMLMEGDDQKLLMASDA |
| SRR3027716 | QPVVFIDTTGRSYAIDPITLPSARGQGEPLTGKLTLPPGATVEHMLMEGDDQKLLMASDA |
| SRR3027717 | QPVVFIDTTGRSYAIDPITLPSARGQGEPLTGKLTLPPGATVEHMLMEGDDQKLLMASDA |
| SRR3027719 | QPVVFIDTTGRSYAIDPITLPSARGQGEPLTGKLTLPPGATVEHMLMEGDDQKLLMASDA |
| SRR3027721 | QPVVFIDTTGRSYAIDPITLPSARGQGEPLTGKLTLPPGATVEHMLMEGDDQKLLMASDA |
| SRR3027723 | QPVVFIDTTGRSYAIDPITLPSARGQGEPLTGKLTLPPGATVEHMLMEGDDQKLLMASDA |
| SRR3115978 | QPVVFIDTTGRSYAIDPITLPSARGQGEPLTGKLTLPPGATVEHMLMEGDDQKLLMASDA |
| SRR2534093 | QPVVFIDTTGRSYAIDPITLPSARGQGEPLTGKLTLPPGATVEHMLMEGDDQKLLMASDA |
| SRR2534094 | QPVVFIDTTGRSYAIDPITLPSARGQGEPLTGKLTLPPGATVEHMLMEGDDQKLLMASDA |
| SRR2534095 | QPVVFIDTTGRSYAIDPITLPSARGQGEPLTGKLTLPPGATVEHMLMEGDDQKLLMASDA |
| SRR2534108 | QPVVFIDTTGRSYAIDPITLPSARGQGEPLTGKLTLPPGATVEHMLMEGDDQKLLMASDA |
| SRR1106464 | QPVVFIDTTGRSYAIDPITLPSARGQGEPLTGKLTLPPGATVEHMLMEGDDQKLLMASDA |
| SRR1106463 | QPVVFIDTTGRSYAIDPITLPSARGQGEPLTGKLTLPPGATVEHMLMEGDDQKLLMASDA |
| SRR6949610 | QPVVFIDTTGRSYAIDPITLPSARGQGEPLTGKLTLPPGATVEHMLMEGDDQKLLMASDA |
| SRR6950452 | QPVVFIDTTGRSYAIDPITLPSARGQGEPLTGKLTLPPGATVEHMLMEGDDQKLLMASDA |
| ERR2019831 | QPVVFIDTTGRSYAIDPITLPSARGQGEPLTGKLTLPPGATVEHMLMEGDDQKLLMASDA |
| SRR2085693 | QPVVFIDTTGRSYAIDPITLPSARGQGEPLTGKLTLPPGATVEHMLMEGDDQKLLMASDA |
| SRR2086898 | QPVVFIDTTGRSYAIDPITLPSARGQGEPLTGKLTLPPGATVEHMLMEGDDQKLLMASDA |
| SRR2175312 | QPVVFIDTTGRSYAIDPITLPSARGQGEPLTGKLTLPPGATVEHMLMEGDDQKLLMASDA |
| SRR2175360 | QPVVFIDTTGRSYAIDPITLPSARGQGEPLTGKLTLPPGATVEHMLMEGDDQKLLMASDA |
| SRR5231997 | QPVVFIDTTGRSYAIDPITLPSARGQGEPLTGKLTLPPGATVEHMLMEGDDQKLLMASDA |
| SRR5232003 | QPVVFIDTTGRSYAIDPITLPSARGQGEPLTGKLTLPPGATVEHMLMEGDDQKLLMASDA |
| SRR5232015 | QPVVFIDTTGRSYAIDPITLPSARGQGEPLTGKLTLPPGATVEHMLMEGDDQKLLMASDA |
| SRR949434 | QPVVFIDTTGRSYAIDPITLPSARGQGEPLTGKLTLPPGATVEHMLMEGDDQKLLMASDA |
| SRR3216575 | QPVVFIDTTGRSYAIDPITLPSARGQGEPLTGKLTLPPGATVEHMLMEGDDQKLLMASDA |
| SRR5205342 | QPVVFIDTTGRSYAIDPITLPSARGQGEPLTGKLTLPPGATVEHMLMEGDDQKLLMASDA |
| SRR1501669 | QPVVFIDTTGRSYAIDPITLPSARGQGEPLTGKLTLPPGATVEHMLMEGDDQKLLMASDA |
| SRR5209740 | QPVVFIDTTGRSYAIDPITLPSARGQGEPLTGKLTLPPGATVEHMLMEGDDQKLLMASDA |
| SRR3240355 | QPVVFIDTTGRSYAIDPITLPSARGQGEPLTGKLTLPPGATVEHMLMEGDDQKLLMASDA |
| SRR3392777 | QPVVFIDTTGRSYAIDPITLPSARGQGEPLTGKLTLPPGATVEHMLMEGDDQKLLMASDA |
| SRR3593671 | QPVVFIDTTGRSYAIDPITLPSARGQGEPLTGKLTLPPGATVEHMLMEGDDQKLLMASDA |
| SRR5413290 | QPVVFIDTTGRSYAIDPITLPSARGQGEPLTGKLTLPPGATVEHMLMEGDDQKLLMASDA |
| SRR5590269 | QPVVFIDTTGRSYAIDPITLPSARGQGEPLTGKLTLPPGATVEHMLMEGDDQKLLMASDA |
| SRR5812103 | QPVVFIDTTGRSYAIDPITLPSARGQGEPLTGKLTLPPGATVEHMLMEGDDQKLLMASDA |
| SRR2830941 | QPVVFIDTTGRSYAIDPITLPSARGQGEPLTGKLTLPPGATVEHMLMEGDDQKLLMASDA |
| SRR2830966 | QPVVFIDTTGRSYAIDPITLPSARGQGEPLTGKLTLPPGATVEHMLMEGDDQKLLMASDA |
| SRR3137270 | QPVVFIDTTGRSYAIDPITLPSARGQGEPLTGKLTLPPGATVEHMLMEGDDQKLLMASDA |
| SRR3137271 | QPVVFIDTTGRSYAIDPITLPSARGQGEPLTGKLTLPPGATVEHMLMEGDDQKLLMASDA |
| ERR526807 | QPVVFIDTTGRSYAIDPITLPSARGQGEPLTGKLTLPPGATVEHMLMEGDDQKLLMASDA |
| ERR2197922 | QPVVFIDTTGRSYAIDPITLPSARGQGEPLTGKLTLPPGATVEHMLMEGDDQKLLMASDA |
| ERR2197923 | QPVVFIDTTGRSYAIDPITLPSARGQGEPLTGKLTLPPGATVEHMLMEGDDQKLLMASDA |
| ERR2197924 | QPVVFIDTTGRSYAIDPITLPSARGQGEPLTGKLTLPPGATVEHMLMEGDDQKLLMASDA |
| ERR2197925 | QPVVFIDTTGRSYAIDPITLPSARGQGEPLTGKLTLPPGATVEHMLMEGDDQKLLMASDA |
| ERR2197927 | QPVVFIDTTGRSYAIDPITLPSARGQGEPLTGKLTLPPGATVEHMLMEGDDQKLLMASDA |
| ERR2197929 | QPVVFIDTTGRSYAIDPITLPSARGQGEPLTGKLTLPPGATVEHMLMEGDDQKLLMASDA |
| SRR1648149 | QPVVFIDTTGRSYAIDPITLPSARGQGEPLTGKLTLPPGATVEHMLMEGDDQKLLMASDA |
| SRR1048299 | QPVVFIDTTGRSYAIDPITLPSARGQGEPLTGKLTLPPGATVEHMLMEGDDQKLLMASDA |
| SRR1300677 | QPVVFIDTTGRSYAIDPITLPSARGQGEPLTGKLTLPPGATVEHMLMEGDDQKLLMASDA |
| SRR1288356 | QPVVFIDTTGRSYAIDPITLPSARGQGEPLTGKLTLPPGATVEHMLMEGDDQKLLMASDA |
| SRR7426190 | QPVVFIDTTGRSYAIDPITLPSARGQGEPLTGKLTLPPGATVEHMLMEGDDQKLLMASDA |
| SRR7426192 | QPVVFIDTTGRSYAIDPITLPSARGQGEPLTGKLTLPPGATVEHMLMEGDDQKLLMASDA |
| SRR7426193 | QPVVFIDTTGRSYAIDPITLPSARGQGEPLTGKLTLPPGATVEHMLMEGDDQKLLMASDA |
| SRR7441832 | QPVVFIDTTGRSYAIDPITLPSARGQGEPLTGKLTLPPGATVEHMLMEGDDQKLLMASDA |
| SRR7426179 | QPVVFIDTTGRSYAIDPITLPSARGQGEPLTGKLTLPPGATVEHMLMEGDDQKLLMASDA |
| SRR7439238 | QPVVFIDTTGRSYAIDPITLPSARGQGEPLTGKLTLPPGATVEHMLMEGDDQKLLMASDA |
| SRR7439244 | QPVVFIDTTGRSYAIDPITLPSARGQGEPLTGKLTLPPGATVEHMLMEGDDQKLLMASDA |
| SRR7439259 | QPVVFIDTTGRSYAIDPITLPSARGQGEPLTGKLTLPPGATVEHMLMEGDDQKLLMASDA |
| SRR7439260 | QPVVFIDTTGRSYAIDPITLPSARGQGEPLTGKLTLPPGATVEHMLMEGDDQKLLMASDA |
| SRR7441786 | QPVVFIDTTGRSYAIDPITLPSARGQGEPLTGKLTLPPGATVEHMLMEGDDQKLLMASDA |
| SRR7441797 | QPVVFIDTTGRSYAIDPITLPSARGQGEPLTGKLTLPPGATVEHMLMEGDDQKLLMASDA |
| ERR1759093 | QPVVFIDTTGRSYAIDPITLPSARGQGEPLTGKLTLPPGATVEHMLMEGDDQKLLMASDA |
| ERR2580275 | QPVVFIDTTGRSYAIDPITLPSARGQGEPLTGKLTLPPGATVEHMLMEGDDQKLLMASDA |
| ERR1759204 | QPVVFIDTTGRSYAIDPITLPSARGQGEPLTGKLTLPPGATVEHMLMEGDDQKLLMASDA |
| SRR1300699 | QPVVFIDTTGRSYAIDPITLPSARGQGEPLTGKLTLPPGATVEHMLMEGDDQKLLMASDA |
| S_0825_17 | QPVVFIDTTGRSYAIDPITLPSARGQGEPLTGKLTLPPGATVEHMLMEGDDQKLLMASDA |
| SRR1958215 | QPVVFIDTTGRSYAIDPITLPSARGQGEPLTGKLTLPPGATVEHMLMEGDDQKLLMASDA |
| SRR1958540 | QPVVFIDTTGRSYAIDPITLPSARGQGEPLTGKLTLPPGATVEHMLMEGDDQKLLMASDA |
| SRR1958636 | QPVVFIDTTGRSYAIDPITLPSARGQGEPLTGKLTLPPGATVEHMLMEGDDQKLLMASDA |
| SRR1959422 | QPVVFIDTTGRSYAIDPITLPSARGQGEPLTGKLTLPPGATVEHMLMEGDDQKLLMASDA |
| SRR1959427 | QPVVFIDTTGRSYAIDPITLPSARGQGEPLTGKLTLPPGATVEHMLMEGDDQKLLMASDA |
| SRR1960226 | QPVVFIDTTGRSYAIDPITLPSARGQGEPLTGKLTLPPGATVEHMLMEGDDQKLLMASDA |
| SRR1963498 | QPVVFIDTTGRSYAIDPITLPSARGQGEPLTGKLTLPPGATVEHMLMEGDDQKLLMASDA |
| SRR1965947 | QPVVFIDTTGRSYAIDPITLPSARGQGEPLTGKLTLPPGATVEHMLMEGDDQKLLMASDA |
| SRR1966125 | QPVVFIDTTGRSYAIDPITLPSARGQGEPLTGKLTLPPGATVEHMLMEGDDQKLLMASDA |
| SRR1966330 | QPVVFIDTTGRSYAIDPITLPSARGQGEPLTGKLTLPPGATVEHMLMEGDDQKLLMASDA |
| SRR1966565 | QPVVFIDTTGRSYAIDPITLPSARGQGEPLTGKLTLPPGATVEHMLMEGDDQKLLMASDA |
| SRR1966864 | QPVVFIDTTGRSYAIDPITLPSARGQGEPLTGKLTLPPGATVEHMLMEGDDQKLLMASDA |
| SRR1966989 | QPVVFIDTTGRSYAIDPITLPSARGQGEPLTGKLTLPPGATVEHMLMEGDDQKLLMASDA |
| SRR1967688 | QPVVFIDTTGRSYAIDPITLPSARGQGEPLTGKLTLPPGATVEHMLMEGDDQKLLMASDA |
| SRR1967733 | QPVVFIDTTGRSYAIDPITLPSARGQGEPLTGKLTLPPGATVEHMLMEGDDQKLLMASDA |
| SRR1967746 | QPVVFIDTTGRSYAIDPITLPSARGQGEPLTGKLTLPPGATVEHMLMEGDDQKLLMASDA |
| SRR1968341 | QPVVFIDTTGRSYAIDPITLPSARGQGEPLTGKLTLPPGATVEHMLMEGDDQKLLMASDA |
| SRR1968456 | QPVVFIDTTGRSYAIDPITLPSARGQGEPLTGKLTLPPGATVEHMLMEGDDQKLLMASDA |
| SRR1968465 | QPVVFIDTTGRSYAIDPITLPSARGQGEPLTGKLTLPPGATVEHMLMEGDDQKLLMASDA |
| SRR1968761 | QPVVFIDTTGRSYAIDPITLPSARGQGEPLTGKLTLPPGATVEHMLMEGDDQKLLMASDA |
| SRR1969047 | QPVVFIDTTGRSYAIDPITLPSARGQGEPLTGKLTLPPGATVEHMLMEGDDQKLLMASDA |
| SRR1969255 | QPVVFIDTTGRSYAIDPITLPSARGQGEPLTGKLTLPPGATVEHMLMEGDDQKLLMASDA |
| SRR1969412 | QPVVFIDTTGRSYAIDPITLPSARGQGEPLTGKLTLPPGATVEHMLMEGDDQKLLMASDA |
| SRR1969524 | QPVVFIDTTGRSYAIDPITLPSARGQGEPLTGKLTLPPGATVEHMLMEGDDQKLLMASDA |
| SRR1969584 | QPVVFIDTTGRSYAIDPITLPSARGQGEPLTGKLTLPPGATVEHMLMEGDDQKLLMASDA |
| SRR1969648 | QPVVFIDTTGRSYAIDPITLPSARGQGEPLTGKLTLPPGATVEHMLMEGDDQKLLMASDA |
| SRR1969804 | QPVVFIDTTGRSYAIDPITLPSARGQGEPLTGKLTLPPGATVEHMLMEGDDQKLLMASDA |
| SRR1970221 | QPVVFIDTTGRSYAIDPITLPSARGQGEPLTGKLTLPPGATVEHMLMEGDDQKLLMASDA |
| SRR1970268 | QPVVFIDTTGRSYAIDPITLPSARGQGEPLTGKLTLPPGATVEHMLMEGDDQKLLMASDA |
| SRR1965862 | QPVVFIDTTGRSYAIDPITLPSARGQGEPLTGKLTLPPGATVEHMLMEGDDQKLLMASDA |
| SRR1967363 | QPVVFIDTTGRSYAIDPITLPSARGQGEPLTGKLTLPPGATVEHMLMEGDDQKLLMASDA |
| SRR1968276 | QPVVFIDTTGRSYAIDPITLPSARGQGEPLTGKLTLPPGATVEHMLMEGDDQKLLMASDA |
| SRR1968967 | QPVVFIDTTGRSYAIDPITLPSARGQGEPLTGKLTLPPGATVEHMLMEGDDQKLLMASDA |
| SRR3321531 | QPVVFIDTTGRSYAIDPITLPSARGQGEPLTGKLTLPPGATVEHMLMEGDDQKLLMASDA |
| SRR3321883 | QPVVFIDTTGRSYAIDPITLPSARGQGEPLTGKLTLPPGATVEHMLMEGDDQKLLMASDA |
| SRR3322413 | QPVVFIDTTGRSYAIDPITLPSARGQGEPLTGKLTLPPGATVEHMLMEGDDQKLLMASDA |
| SRR3323012 | QPVVFIDTTGRSYAIDPITLPSARGQGEPLTGKLTLPPGATVEHMLMEGDDQKLLMASDA |
| SRR5194289 | QPVVFIDTTGRSYAIDPITLPSARGQGEPLTGKLTLPPGATVEHMLMEGDDQKLLMASDA |
| SRR7163798 | QPVVFIDTTGRSYAIDPITLPSARGQGEPLTGKLTLPPGATVEHMLMEGDDQKLLMASDA |
| SRR7172610 | QPVVFIDTTGRSYAIDPITLPSARGQGEPLTGKLTLPPGATVEHMLMEGDDQKLLMASDA |
| SRR7204568 | QPVVFIDTTGRSYAIDPITLPSARGQGEPLTGKLTLPPGATVEHMLMEGDDQKLLMASDA |
| SRR7223230 | QPVVFIDTTGRSYAIDPITLPSARGQGEPLTGKLTLPPGATVEHMLMEGDDQKLLMASDA |
| SRR7230675 | QPVVFIDTTGRSYAIDPITLPSARGQGEPLTGKLTLPPGATVEHMLMEGDDQKLLMASDA |
| SRR7278056 | QPVVFIDTTGRSYAIDPITLPSARGQGEPLTGKLTLPPGATVEHMLMEGDDQKLLMASDA |
| SRR7278086 | QPVVFIDTTGRSYAIDPITLPSARGQGEPLTGKLTLPPGATVEHMLMEGDDQKLLMASDA |
| SRR7285841 | QPVVFIDTTGRSYAIDPITLPSARGQGEPLTGKLTLPPGATVEHMLMEGDDQKLLMASDA |
| SRR7292625 | QPVVFIDTTGRSYAIDPITLPSARGQGEPLTGKLTLPPGATVEHMLMEGDDQKLLMASDA |
| SRR7292665 | QPVVFIDTTGRSYAIDPITLPSARGQGEPLTGKLTLPPGATVEHMLMEGDDQKLLMASDA |
| SRR7297965 | QPVVFIDTTGRSYAIDPITLPSARGQGEPLTGKLTLPPGATVEHMLMEGDDQKLLMASDA |
| SRR7350726 | QPVVFIDTTGRSYAIDPITLPSARGQGEPLTGKLTLPPGATVEHMLMEGDDQKLLMASDA |
| SRR7410328 | QPVVFIDTTGRSYAIDPITLPSARGQGEPLTGKLTLPPGATVEHMLMEGDDQKLLMASDA |
| SRR7474665 | QPVVFIDTTGRSYAIDPITLPSARGQGEPLTGKLTLPPGATVEHMLMEGDDQKLLMASDA |
| SRR7523184 | QPVVFIDTTGRSYAIDPITLPSARGQGEPLTGKLTLPPGATVEHMLMEGDDQKLLMASDA |
| SRR7187264 | QPVVFIDTTGRSYAIDPITLPSARGQGEPLTGKLTLPPGATVEHMLMEGDDQKLLMASDA |
| SRR7204445 | QPVVFIDTTGRSYAIDPITLPSARGQGEPLTGKLTLPPGATVEHMLMEGDDQKLLMASDA |
| SRR7285641 | QPVVFIDTTGRSYAIDPITLPSARGQGEPLTGKLTLPPGATVEHMLMEGDDQKLLMASDA |
| SRR7286695 | QPVVFIDTTGRSYAIDPITLPSARGQGEPLTGKLTLPPGATVEHMLMEGDDQKLLMASDA |
| SRR7286705 | QPVVFIDTTGRSYAIDPITLPSARGQGEPLTGKLTLPPGATVEHMLMEGDDQKLLMASDA |
| SRR7292931 | QPVVFIDTTGRSYAIDPITLPSARGQGEPLTGKLTLPPGATVEHMLMEGDDQKLLMASDA |
| SRR7310349 | QPVVFIDTTGRSYAIDPITLPSARGQGEPLTGKLTLPPGATVEHMLMEGDDQKLLMASDA |
| SRR7351616 | QPVVFIDTTGRSYAIDPITLPSARGQGEPLTGKLTLPPGATVEHMLMEGDDQKLLMASDA |
| SRR7414818 | QPVVFIDTTGRSYAIDPITLPSARGQGEPLTGKLTLPPGATVEHMLMEGDDQKLLMASDA |
| SRR7426480 | QPVVFIDTTGRSYAIDPITLPSARGQGEPLTGKLTLPPGATVEHMLMEGDDQKLLMASDA |
| SRR5584105 | QPVVFIDTTGRSYAIDPITLPSARGQGEPLTGKLTLPPGATVEHMLMEGDDQKLLMASDA |
| SRR5584565 | QPVVFIDTTGRSYAIDPITLPSARGQGEPLTGKLTLPPGATVEHMLMEGDDQKLLMASDA |
| SRR5584614 | QPVVFIDTTGRSYAIDPITLPSARGQGEPLTGKLTLPPGATVEHMLMEGDDQKLLMASDA |
| SRR5631543 | QPVVFIDTTGRSYAIDPITLPSARGQGEPLTGKLTLPPGATVEHMLMEGDDQKLLMASDA |
| SRR5631553 | QPVVFIDTTGRSYAIDPITLPSARGQGEPLTGKLTLPPGATVEHMLMEGDDQKLLMASDA |
| SRR7123196 | QPVVFIDTTGRSYAIDPITLPSARGQGEPLTGKLTLPPGATVEHMLMEGDDQKLLMASDA |
| SRR7163819 | QPVVFIDTTGRSYAIDPITLPSARGQGEPLTGKLTLPPGATVEHMLMEGDDQKLLMASDA |
| SRR7163920 | QPVVFIDTTGRSYAIDPITLPSARGQGEPLTGKLTLPPGATVEHMLMEGDDQKLLMASDA |
| SRR7209528 | QPVVFIDTTGRSYAIDPITLPSARGQGEPLTGKLTLPPGATVEHMLMEGDDQKLLMASDA |
| SRR7249868 | QPVVFIDTTGRSYAIDPITLPSARGQGEPLTGKLTLPPGATVEHMLMEGDDQKLLMASDA |
| SRR7278088 | QPVVFIDTTGRSYAIDPITLPSARGQGEPLTGKLTLPPGATVEHMLMEGDDQKLLMASDA |
| SRR7285788 | QPVVFIDTTGRSYAIDPITLPSARGQGEPLTGKLTLPPGATVEHMLMEGDDQKLLMASDA |
| SRR7286789 | QPVVFIDTTGRSYAIDPITLPSARGQGEPLTGKLTLPPGATVEHMLMEGDDQKLLMASDA |
| SRR7286886 | QPVVFIDTTGRSYAIDPITLPSARGQGEPLTGKLTLPPGATVEHMLMEGDDQKLLMASDA |
| SRR7310632 | QPVVFIDTTGRSYAIDPITLPSARGQGEPLTGKLTLPPGATVEHMLMEGDDQKLLMASDA |
| SRR7350631 | QPVVFIDTTGRSYAIDPITLPSARGQGEPLTGKLTLPPGATVEHMLMEGDDQKLLMASDA |
| SRR7458741 | QPVVFIDTTGRSYAIDPITLPSARGQGEPLTGKLTLPPGATVEHMLMEGDDQKLLMASDA |
| SRR7480280 | QPVVFIDTTGRSYAIDPITLPSARGQGEPLTGKLTLPPGATVEHMLMEGDDQKLLMASDA |
| SRR7523660 | QPVVFIDTTGRSYAIDPITLPSARGQGEPLTGKLTLPPGATVEHMLMEGDDQKLLMASDA |
| SRR7523775 | QPVVFIDTTGRSYAIDPITLPSARGQGEPLTGKLTLPPGATVEHMLMEGDDQKLLMASDA |
| SRR7251101 | QPVVFIDTTGRSYAIDPITLPSARGQGEPLTGKLTLPPGATVEHMLMEGDDQKLLMASDA |
| SRR7284299 | QPVVFIDTTGRSYAIDPITLPSARGQGEPLTGKLTLPPGATVEHMLMEGDDQKLLMASDA |
| SRR7285738 | QPVVFIDTTGRSYAIDPITLPSARGQGEPLTGKLTLPPGATVEHMLMEGDDQKLLMASDA |
| SRR7310640 | QPVVFIDTTGRSYAIDPITLPSARGQGEPLTGKLTLPPGATVEHMLMEGDDQKLLMASDA |
| SRR7349159 | QPVVFIDTTGRSYAIDPITLPSARGQGEPLTGKLTLPPGATVEHMLMEGDDQKLLMASDA |
| SRR7474873 | QPVVFIDTTGRSYAIDPITLPSARGQGEPLTGKLTLPPGATVEHMLMEGDDQKLLMASDA |
| SRR7495689 | QPVVFIDTTGRSYAIDPITLPSARGQGEPLTGKLTLPPGATVEHMLMEGDDQKLLMASDA |
| SRR7495752 | QPVVFIDTTGRSYAIDPITLPSARGQGEPLTGKLTLPPGATVEHMLMEGDDQKLLMASDA |
| ------------------------------------------------------------------------------ | |
| S16BD08730 | GYGFVCTFNDLVARNRAGKALITLPENAHVMPPLVIEDEHDMLLAITQAGRMLMFPVDSL |
| S18BD00684 | GYGFVCTFNDLVARNRAGKALITLPENAHVMPPLVIEDEHDMLLAITQAGRMLMFPVDSL |
| S18BD03994 | GYGFVCTFNDLVARNRAGKALITLPENAHVMPPLVIEDEHDMLLAITQAGRMLMFPVDSL |
| S18BD05011 | GYGFVCTFNDLVARNRAGKALITLPENAHVMPPLVIEDEHDMLLAITQAGRMLMFPVDSL |
| RKI_16-03723 | GYGFVCTFNDLVARNRAGKALITLPENAHVMPPLVIEDEHDMLLAITQAGRMLMFPVDSL |
| RKI_16-04315 | GYGFVCTFNDLVARNRAGKALITLPENAHVMPPLVIEDEHDMLLAITQAGRMLMFPVDSL |
| RKI_17-02304 | GYGFVCTFNDLVARNRAGKALITLPENAHVMPPLVIEDEHDMLLAITQAGRMLMFPVDSL |
| RKI_17-02411 | GYGFVCTFNDLVARNRAGKALITLPENAHVMPPLVIEDEHDMLLAITQAGRMLMFPVDSL |
| RKI_17-02757 | GYGFVCTFNDLVARNRAGKALITLPENAHVMPPLVIEDEHDMLLAITQAGRMLMFPVDSL |
| RKI_17-04797 | GYGFVCTFNDLVARNRAGKALITLPENAHVMPPLVIEDEHDMLLAITQAGRMLMFPVDSL |
| RKI_17-06869 | GYGFVCTFNDLVARNRAGKALITLPENAHVMPPLVIEDEHDMLLAITQAGRMLMFPVDSL |
| ERR2580277 | GYGFVCTFNDLVARNRAGKALITLPENAHVMPPLVIEDEHDMLLAITQAGRMLMFPVDSL |
| ERR2580276 | GYGFVCTFNDLVARNRAGKALITLPENAHVMPPLVIEDEHDMLLAITQAGRMLMFPVDSL |
| ERR2580273 | GYGFVCTFNDLVARNRAGKALITLPENAHVMPPLVIEDEHDMLLAITQAGRMLMFPVDSL |
| ERR2580274 | GYGFVCTFNDLVARNRAGKALITLPENAHVMPPLVIEDEHDMLLAITQAGRMLMFPVDSL |
| ERR2173656 | GYGFVCTFNDLVARNRAGKALITLPENAHVMPPLVIEDEHDMLLAITQAGRMLMFPVDSL |
| 17041676 | GYGFVCTFNDLVARNRAGKALITLPENAHVMPPLVIEDEHDMLLAITQAGRMLMFPVDSL |
| MT16-000061 | GYGFVCTFNDLVARNRAGKALITLPENAHVMPPLVIEDEHDMLLAITQAGRMLMFPVDSL |
| MT16-019416 | GYGFVCTFNDLVARNRAGKALITLPENAHVMPPLVIEDEHDMLLAITQAGRMLMFPVDSL |
| MT16-027865 | GYGFVCTFNDLVARNRAGKALITLPENAHVMPPLVIEDEHDMLLAITQAGRMLMFPVDSL |
| MT16-031693 | GYGFVCTFNDLVARNRAGKALITLPENAHVMPPLVIEDEHDMLLAITQAGRMLMFPVDSL |
| MT16-040253 | GYGFVCTFNDLVARNRAGKALITLPENAHVMPPLVIEDEHDMLLAITQAGRMLMFPVDSL |
| MT16-045379 | GYGFVCTFNDLVARNRAGKALITLPENAHVMPPLVIEDEHDMLLAITQAGRMLMFPVDSL |
| MT16-442728 | GYGFVCTFNDLVARNRAGKALITLPENAHVMPPLVIEDEHDMLLAITQAGRMLMFPVDSL |
| MT16-462857 | GYGFVCTFNDLVARNRAGKALITLPENAHVMPPLVIEDEHDMLLAITQAGRMLMFPVDSL |
| MT16-480196 | GYGFVCTFNDLVARNRAGKALITLPENAHVMPPLVIEDEHDMLLAITQAGRMLMFPVDSL |
| MT16-861555 | GYGFVCTFNDLVARNRAGKALITLPENAHVMPPLVIEDEHDMLLAITQAGRMLMFPVDSL |
| MT17-076833 | GYGFVCTFNDLVARNRAGKALITLPENAHVMPPLVIEDEHDMLLAITQAGRMLMFPVDSL |
| MT17-110677 | GYGFVCTFNDLVARNRAGKALITLPENAHVMPPLVIEDEHDMLLAITQAGRMLMFPVDSL |
| MT17-131730 | GYGFVCTFNDLVARNRAGKALITLPENAHVMPPLVIEDEHDMLLAITQAGRMLMFPVDSL |
| MT17-140890 | GYGFVCTFNDLVARNRAGKALITLPENAHVMPPLVIEDEHDMLLAITQAGRMLMFPVDSL |
| MT17-141840 | GYGFVCTFNDLVARNRAGKALITLPENAHVMPPLVIEDEHDMLLAITQAGRMLMFPVDSL |
| MT17-152488 | GYGFVCTFNDLVARNRAGKALITLPENAHVMPPLVIEDEHDMLLAITQAGRMLMFPVDSL |
| MT17-157311 | GYGFVCTFNDLVARNRAGKALITLPENAHVMPPLVIEDEHDMLLAITQAGRMLMFPVDSL |
| MT17-161645 | GYGFVCTFNDLVARNRAGKALITLPENAHVMPPLVIEDEHDMLLAITQAGRMLMFPVDSL |
| MT17-167951 | GYGFVCTFNDLVARNRAGKALITLPENAHVMPPLVIEDEHDMLLAITQAGRMLMFPVDSL |
| MT18-217732 | GYGFVCTFNDLVARNRAGKALITLPENAHVMPPLVIEDEHDMLLAITQAGRMLMFPVDSL |
| MT18-252580 | GYGFVCTFNDLVARNRAGKALITLPENAHVMPPLVIEDEHDMLLAITQAGRMLMFPVDSL |
| RIVM_H_2009-01 | GYGFVCTFNDLVARNRAGKALITLPENAHVMPPLVIEDEHDMLLAITQAGRMLMFPVDSL |
| RIVM_H_2010-01 | GYGFVCTFNDLVARNRAGKALITLPENAHVMPPLVIEDEHDMLLAITQAGRMLMFPVDSL |
| RIVM_H_2010-02 | GYGFVCTFNDLVARNRAGKALITLPENAHVMPPLVIEDEHDMLLAITQAGRMLMFPVDSL |
| RIVM_H_2011-01 | GYGFVCTFNDLVARNRAGKALITLPENAHVMPPLVIEDEHDMLLAITQAGRMLMFPVDSL |
| RIVM_H_2011-02 | GYGFVCTFNDLVARNRAGKALITLPENAHVMPPLVIEDEHDMLLAITQAGRMLMFPVDSL |
| RIVM_H_2011-03 | GYGFVCTFNDLVARNRAGKALITLPENAHVMPPLVIEDEHDMLLAITQAGRMLMFPVDSL |
| RIVM_H_2013-01 | GYGFVCTFNDLVARNRAGKALITLPENAHVMPPLVIEDEHDMLLAITQAGRMLMFPVDSL |
| RIVM_H_2013-02 | GYGFVCTFNDLVARNRAGKALITLPENAHVMPPLVIEDEHDMLLAITQAGRMLMFPVDSL |
| RIVM_H_2014-01 | GYGFVCTFNDLVARNRAGKALITLPENAHVMPPLVIEDEHDMLLAITQAGRMLMFPVDSL |
| RIVM_H_2014-02 | GYGFVCTFNDLVARNRAGKALITLPENAHVMPPLVIEDEHDMLLAITQAGRMLMFPVDSL |
| RIVM_H_2016-01 | GYGFVCTFNDLVARNRAGKALITLPENAHVMPPLVIEDEHDMLLAITQAGRMLMFPVDSL |
| RIVM_H_2016-02 | GYGFVCTFNDLVARNRAGKALITLPENAHVMPPLVIEDEHDMLLAITQAGRMLMFPVDSL |
| RIVM_H_2016-03 | GYGFVCTFNDLVARNRAGKALITLPENAHVMPPLVIEDEHDMLLAITQAGRMLMFPVDSL |
| RIVM_H_2016-04 | GYGFVCTFNDLVARNRAGKALITLPENAHVMPPLVIEDEHDMLLAITQAGRMLMFPVDSL |
| RIVM_H_2016-05 | GYGFVCTFNDLVARNRAGKALITLPENAHVMPPLVIEDEHDMLLAITQAGRMLMFPVDSL |
| RIVM_H_2016-06 | GYGFVCTFNDLVARNRAGKALITLPENAHVMPPLVIEDEHDMLLAITQAGRMLMFPVDSL |
| RIVM_H_2016-07 | GYGFVCTFNDLVARNRAGKALITLPENAHVMPPLVIEDEHDMLLAITQAGRMLMFPVDSL |
| RIVM_H_2016-08 | GYGFVCTFNDLVARNRAGKALITLPENAHVMPPLVIEDEHDMLLAITQAGRMLMFPVDSL |
| RIVM_H_2016-09 | GYGFVCTFNDLVARNRAGKALITLPENAHVMPPLVIEDEHDMLLAITQAGRMLMFPVDSL |
| RIVM_H_2016-10 | GYGFVCTFNDLVARNRAGKALITLPENAHVMPPLVIEDEHDMLLAITQAGRMLMFPVDSL |
| RIVM_H_2016-11 | GYGFVCTFNDLVARNRAGKALITLPENAHVMPPLVIEDEHDMLLAITQAGRMLMFPVDSL |
| RIVM_H_2016-12 | GYGFVCTFNDLVARNRAGKALITLPENAHVMPPLVIEDEHDMLLAITQAGRMLMFPVDSL |
| RIVM_H_2016-13 | GYGFVCTFNDLVARNRAGKALITLPENAHVMPPLVIEDEHDMLLAITQAGRMLMFPVDSL |
| RIVM_H_2016-14 | GYGFVCTFNDLVARNRAGKALITLPENAHVMPPLVIEDEHDMLLAITQAGRMLMFPVDSL |
| RIVM_H_2016-15 | GYGFVCTFNDLVARNRAGKALITLPENAHVMPPLVIEDEHDMLLAITQAGRMLMFPVDSL |
| RIVM_H_2017-01 | GYGFVCTFNDLVARNRAGKALITLPENAHVMPPLVIEDEHDMLLAITQAGRMLMFPVDSL |
| RIVM_H_2017-02 | GYGFVCTFNDLVARNRAGKALITLPENAHVMPPLVIEDEHDMLLAITQAGRMLMFPVDSL |
| RIVM_H_2017-03 | GYGFVCTFNDLVARNRAGKALITLPENAHVMPPLVIEDEHDMLLAITQAGRMLMFPVDSL |
| RIVM_H_2017-04 | GYGFVCTFNDLVARNRAGKALITLPENAHVMPPLVIEDEHDMLLAITQAGRMLMFPVDSL |
| RIVM_H_2017-05 | GYGFVCTFNDLVARNRAGKALITLPENAHVMPPLVIEDEHDMLLAITQAGRMLMFPVDSL |
| RIVM_H_2017-06 | GYGFVCTFNDLVARNRAGKALITLPENAHVMPPLVIEDEHDMLLAITQAGRMLMFPVDSL |
| RIVM_H_2017-07 | GYGFVCTFNDLVARNRAGKALITLPENAHVMPPLVIEDEHDMLLAITQAGRMLMFPVDSL |
| RIVM_H_2017-08 | GYGFVCTFNDLVARNRAGKALITLPENAHVMPPLVIEDEHDMLLAITQAGRMLMFPVDSL |
| RIVM_H_2017-09 | GYGFVCTFNDLVARNRAGKALITLPENAHVMPPLVIEDEHDMLLAITQAGRMLMFPVDSL |
| RIVM_H_2017-10 | GYGFVCTFNDLVARNRAGKALITLPENAHVMPPLVIEDEHDMLLAITQAGRMLMFPVDSL |
| RIVM_H_2017-11 | GYGFVCTFNDLVARNRAGKALITLPENAHVMPPLVIEDEHDMLLAITQAGRMLMFPVDSL |
| RIVM_H_2017-12 | GYGFVCTFNDLVARNRAGKALITLPENAHVMPPLVIEDEHDMLLAITQAGRMLMFPVDSL |
| RIVM_H_2017-13 | GYGFVCTFNDLVARNRAGKALITLPENAHVMPPLVIEDEHDMLLAITQAGRMLMFPVDSL |
| RIVM_H_2017-14 | GYGFVCTFNDLVARNRAGKALITLPENAHVMPPLVIEDEHDMLLAITQAGRMLMFPVDSL |
| RIVM_H_2017-15 | GYGFVCTFNDLVARNRAGKALITLPENAHVMPPLVIEDEHDMLLAITQAGRMLMFPVDSL |
| RIVM_H_2017-16 | GYGFVCTFNDLVARNRAGKALITLPENAHVMPPLVIEDEHDMLLAITQAGRMLMFPVDSL |
| RIVM_H_2017-17 | GYGFVCTFNDLVARNRAGKALITLPENAHVMPPLVIEDEHDMLLAITQAGRMLMFPVDSL |
| RIVM_H_2017-18 | GYGFVCTFNDLVARNRAGKALITLPENAHVMPPLVIEDEHDMLLAITQAGRMLMFPVDSL |
| RIVM_H_2017-19 | GYGFVCTFNDLVARNRAGKALITLPENAHVMPPLVIEDEHDMLLAITQAGRMLMFPVDSL |
| 15EP001483 | GYGFVCTFNDLVARNRAGKALITLPENAHVMPPLVIEDEHDMLLAITQAGRMLMFPVDSL |
| 17EP002363 | GYGFVCTFNDLVARNRAGKALITLPENAHVMPPLVIEDEHDMLLAITQAGRMLMFPVDSL |
| S_0812_17 | GYGFVCTFNDLVARNRAGKALITLPENAHVMPPLVIEDEHDMLLAITQAGRMLMFPVDSL |
| SRR1957844 | GYGFVCTFNDLVARNRAGKALITLPENAHVMPPLVIEDEHDMLLAITQAGRMLMFPVDSL |
| SRR1958654 | GYGFVCTFNDLVARNRAGKALITLPENAHVMPPLVIEDEHDMLLAITQAGRMLMFPVDSL |
| SRR1965077 | GYGFVCTFNDLVARNRAGKALITLPENAHVMPPLVIEDEHDMLLAITQAGRMLMFPVDSL |
| SRR1966369 | GYGFVCTFNDLVARNRAGKALITLPENAHVMPPLVIEDEHDMLLAITQAGRMLMFPVDSL |
| SRR1967117 | GYGFVCTFNDLVARNRAGKALITLPENAHVMPPLVIEDEHDMLLAITQAGRMLMFPVDSL |
| SRR1967922 | GYGFVCTFNDLVARNRAGKALITLPENAHVMPPLVIEDEHDMLLAITQAGRMLMFPVDSL |
| SRR8704720 | GYGFVCTFNDLVARNRAGKALITLPENAHVMPPLVIEDEHDMLLAITQAGRMLMFPVDSL |
| SRR7216071 | GYGFVCTFNDLVARNRAGKALITLPENAHVMPPLVIEDEHDMLLAITQAGRMLMFPVDSL |
| SRR7349175 | GYGFVCTFNDLVARNRAGKALITLPENAHVMPPLVIEDEHDMLLAITQAGRMLMFPVDSL |
| SRR7523148 | GYGFVCTFNDLVARNRAGKALITLPENAHVMPPLVIEDEHDMLLAITQAGRMLMFPVDSL |
| SRR7523854 | GYGFVCTFNDLVARNRAGKALITLPENAHVMPPLVIEDEHDMLLAITQAGRMLMFPVDSL |
| 313865 | GYGFVCTFNDLVARNRAGKALITLPENAHVMPPLVIEDEHDMLLAITQAGRMLMFPVDSL |
| SRR7277793 | GYGFVCTFNDLVARNRAGKALITLPENAHVMPPLVIEDEHDMLLAITQAGRMLMFPVDSL |
| SRR7343877 | GYGFVCTFNDLVARNRAGKALITLPENAHVMPPLVIEDEHDMLLAITQAGRMLMFPVDSL |
| SRR7351477 | GYGFVCTFNDLVARNRAGKALITLPENAHVMPPLVIEDEHDMLLAITQAGRMLMFPVDSL |
| SRR5583183 | GYGFVCTFNDLVARNRAGKALITLPENAHVMPPLVIEDEHDMLLAITQAGRMLMFPVDSL |
| SRR5585240 | GYGFVCTFNDLVARNRAGKALITLPENAHVMPPLVIEDEHDMLLAITQAGRMLMFPVDSL |
| SRR7284317 | GYGFVCTFNDLVARNRAGKALITLPENAHVMPPLVIEDEHDMLLAITQAGRMLMFPVDSL |
| SRR7299161 | GYGFVCTFNDLVARNRAGKALITLPENAHVMPPLVIEDEHDMLLAITQAGRMLMFPVDSL |
| SRR7401730 | GYGFVCTFNDLVARNRAGKALITLPENAHVMPPLVIEDEHDMLLAITQAGRMLMFPVDSL |
| SRR7469092 | GYGFVCTFNDLVARNRAGKALITLPENAHVMPPLVIEDEHDMLLAITQAGRMLMFPVDSL |
| SRR7879556 | GYGFVCTFNDLVARNRAGKALITLPENAHVMPPLVIEDEHDMLLAITQAGRMLMFPVDSL |
| SRR8526100 | GYGFVCTFNDLVARNRAGKALITLPENAHVMPPLVIEDEHDMLLAITQAGRMLMFPVDSL |
| SRR8553991 | GYGFVCTFNDLVARNRAGKALITLPENAHVMPPLVIEDEHDMLLAITQAGRMLMFPVDSL |
| SRR7842487 | GYGFVCTFNDLVARNRAGKALITLPENAHVMPPLVIEDEHDMLLAITQAGRMLMFPVDSL |
| SRR8054524 | GYGFVCTFNDLVARNRAGKALITLPENAHVMPPLVIEDEHDMLLAITQAGRMLMFPVDSL |
| SRR8054525 | GYGFVCTFNDLVARNRAGKALITLPENAHVMPPLVIEDEHDMLLAITQAGRMLMFPVDSL |
| SRR8524733 | GYGFVCTFNDLVARNRAGKALITLPENAHVMPPLVIEDEHDMLLAITQAGRMLMFPVDSL |
| SRR4093291 | GYGFVCTFNDLVARNRAGKALITLPENAHVMPPLVIEDEHDMLLAITQAGRMLMFPVDSL |
| SRR4245549 | GYGFVCTFNDLVARNRAGKALITLPENAHVMPPLVIEDEHDMLLAITQAGRMLMFPVDSL |
| SRR3057154 | GYGFVCTFNDLVARNRAGKALITLPENAHVMPPLVIEDEHDMLLAITQAGRMLMFPVDSL |
| SRR1726150 | GYGFVCTFNDLVARNRAGKALITLPENAHVMPPLVIEDEHDMLLAITQAGRMLMFPVDSL |
| SRR1996141 | GYGFVCTFNDLVARNRAGKALITLPENAHVMPPLVIEDEHDMLLAITQAGRMLMFPVDSL |
| SRR1107842 | GYGFVCTFNDLVARNRAGKALITLPENAHVMPPLVIEDEHDMLLAITQAGRMLMFPVDSL |
| SRR1157587 | GYGFVCTFNDLVARNRAGKALITLPENAHVMPPLVIEDEHDMLLAITQAGRMLMFPVDSL |
| SRR3027706 | GYGFVCTFNDLVARNRAGKALITLPENAHVMPPLVIEDEHDMLLAITQAGRMLMFPVDSL |
| SRR3027707 | GYGFVCTFNDLVARNRAGKALITLPENAHVMPPLVIEDEHDMLLAITQAGRMLMFPVDSL |
| SRR3027708 | GYGFVCTFNDLVARNRAGKALITLPENAHVMPPLVIEDEHDMLLAITQAGRMLMFPVDSL |
| SRR3027710 | GYGFVCTFNDLVARNRAGKALITLPENAHVMPPLVIEDEHDMLLAITQAGRMLMFPVDSL |
| SRR3027711 | GYGFVCTFNDLVARNRAGKALITLPENAHVMPPLVIEDEHDMLLAITQAGRMLMFPVDSL |
| SRR3027716 | GYGFVCTFNDLVARNRAGKALITLPENAHVMPPLVIEDEHDMLLAITQAGRMLMFPVDSL |
| SRR3027717 | GYGFVCTFNDLVARNRAGKALITLPENAHVMPPLVIEDEHDMLLAITQAGRMLMFPVDSL |
| SRR3027719 | GYGFVCTFNDLVARNRAGKALITLPENAHVMPPLVIEDEHDMLLAITQAGRMLMFPVDSL |
| SRR3027721 | GYGFVCTFNDLVARNRAGKALITLPENAHVMPPLVIEDEHDMLLAITQAGRMLMFPVDSL |
| SRR3027723 | GYGFVCTFNDLVARNRAGKALITLPENAHVMPPLVIEDEHDMLLAITQAGRMLMFPVDSL |
| SRR3115978 | GYGFVCTFNDLVARNRAGKALITLPENAHVMPPLVIEDEHDMLLAITQAGRMLMFPVDSL |
| SRR2534093 | GYGFVCTFNDLVARNRAGKALITLPENAHVMPPLVIEDEHDMLLAITQAGRMLMFPVDSL |
| SRR2534094 | GYGFVCTFNDLVARNRAGKALITLPENAHVMPPLVIEDEHDMLLAITQAGRMLMFPVDSL |
| SRR2534095 | GYGFVCTFNDLVARNRAGKALITLPENAHVMPPLVIEDEHDMLLAITQAGRMLMFPVDSL |
| SRR2534108 | GYGFVCTFNDLVARNRAGKALITLPENAHVMPPLVIEDEHDMLLAITQAGRMLMFPVDSL |
| SRR1106464 | GYGFVCTFNDLVARNRAGKALITLPENAHVMPPLVIEDEHDMLLAITQAGRMLMFPVDSL |
| SRR1106463 | GYGFVCTFNDLVARNRAGKALITLPENAHVMPPLVIEDEHDMLLAITQAGRMLMFPVDSL |
| SRR6949610 | GYGFVCTFNDLVARNRAGKALITLPENAHVMPPLVIEDEHDMLLAITQAGRMLMFPVDSL |
| SRR6950452 | GYGFVCTFNDLVARNRAGKALITLPENAHVMPPLVIEDEHDMLLAITQAGRMLMFPVDSL |
| ERR2019831 | GYGFVCTFNDLVARNRAGKALITLPENAHVMPPLVIEDEHDMLLAITQAGRMLMFPVDSL |
| SRR2085693 | GYGFVCTFNDLVARNRAGKALITLPENAHVMPPLVIEDEHDMLLAITQAGRMLMFPVDSL |
| SRR2086898 | GYGFVCTFNDLVARNRAGKALITLPENAHVMPPLVIEDEHDMLLAITQAGRMLMFPVDSL |
| SRR2175312 | GYGFVCTFNDLVARNRAGKALITLPENAHVMPPLVIEDEHDMLLAITQAGRMLMFPVDSL |
| SRR2175360 | GYGFVCTFNDLVARNRAGKALITLPENAHVMPPLVIEDEHDMLLAITQAGRMLMFPVDSL |
| SRR5231997 | GYGFVCTFNDLVARNRAGKALITLPENAHVMPPLVIEDEHDMLLAITQAGRMLMFPVDSL |
| SRR5232003 | GYGFVCTFNDLVARNRAGKALITLPENAHVMPPLVIEDEHDMLLAITQAGRMLMFPVDSL |
| SRR5232015 | GYGFVCTFNDLVARNRAGKALITLPENAHVMPPLVIEDEHDMLLAITQAGRMLMFPVDSL |
| SRR949434 | GYGFVCTFNDLVARNRAGKALITLPENAHVMPPLVIEDEHDMLLAITQAGRMLMFPVDSL |
| SRR3216575 | GYGFVCTFNDLVARNRAGKALITLPENAHVMPPLVIEDEHDMLLAITQAGRMLMFPVDSL |
| SRR5205342 | GYGFVCTFNDLVARNRAGKALITLPENAHVMPPLVIEDEHDMLLAITQAGRMLMFPVDSL |
| SRR1501669 | GYGFVCTFNDLVARNRAGKALITLPENAHVMPPLVIEDEHDMLLAITQAGRMLMFPVDSL |
| SRR5209740 | GYGFVCTFNDLVARNRAGKALITLPENAHVMPPLVIEDEHDMLLAITQAGRMLMFPVDSL |
| SRR3240355 | GYGFVCTFNDLVARNRAGKALITLPENAHVMPPLVIEDEHDMLLAITQAGRMLMFPVDSL |
| SRR3392777 | GYGFVCTFNDLVARNRAGKALITLPENAHVMPPLVIEDEHDMLLAITQAGRMLMFPVDSL |
| SRR3593671 | GYGFVCTFNDLVARNRAGKALITLPENAHVMPPLVIEDEHDMLLAITQAGRMLMFPVDSL |
| SRR5413290 | GYGFVCTFNDLVARNRAGKALITLPENAHVMPPLVIEDEHDMLLAITQAGRMLMFPVDSL |
| SRR5590269 | GYGFVCTFNDLVARNRAGKALITLPENAHVMPPLVIEDEHDMLLAITQAGRMLMFPVDSL |
| SRR5812103 | GYGFVCTFNDLVARNRAGKALITLPENAHVMPPLVIEDEHDMLLAITQAGRMLMFPVDSL |
| SRR2830941 | GYGFVCTFNDLVARNRAGKALITLPENAHVMPPLVIEDEHDMLLAITQAGRMLMFPVDSL |
| SRR2830966 | GYGFVCTFNDLVARNRAGKALITLPENAHVMPPLVIEDEHDMLLAITQAGRMLMFPVDSL |
| SRR3137270 | GYGFVCTFNDLVARNRAGKALITLPENAHVMPPLVIEDEHDMLLAITQAGRMLMFPVDSL |
| SRR3137271 | GYGFVCTFNDLVARNRAGKALITLPENAHVMPPLVIEDEHDMLLAITQAGRMLMFPVDSL |
| ERR526807 | GYGFVCTFNDLVARNRAGKALITLPENAHVMPPLVIEDEHDMLLAITQAGRMLMFPVDSL |
| ERR2197922 | GYGFVCTFNDLVARNRAGKALITLPENAHVMPPLVIEDEHDMLLAITQAGRMLMFPVDSL |
| ERR2197923 | GYGFVCTFNDLVARNRAGKALITLPENAHVMPPLVIEDEHDMLLAITQAGRMLMFPVDSL |
| ERR2197924 | GYGFVCTFNDLVARNRAGKALITLPENAHVMPPLVIEDEHDMLLAITQAGRMLMFPVDSL |
| ERR2197925 | GYGFVCTFNDLVARNRAGKALITLPENAHVMPPLVIEDEHDMLLAITQAGRMLMFPVDSL |
| ERR2197927 | GYGFVCTFNDLVARNRAGKALITLPENAHVMPPLVIEDEHDMLLAITQAGRMLMFPVDSL |
| ERR2197929 | GYGFVCTFNDLVARNRAGKALITLPENAHVMPPLVIEDEHDMLLAITQAGRMLMFPVDSL |
| SRR1648149 | GYGFVCTFNDLVARNRAGKALITLPENAHVMPPLVIEDEHDMLLAITQAGRMLMFPVDSL |
| SRR1048299 | GYGFVCTFNDLVARNRAGKALITLPENAHVMPPLVIEDEHDMLLAITQAGRMLMFPVDSL |
| SRR1300677 | GYGFVCTFNDLVARNRAGKALITLPENAHVMPPLVIEDEHDMLLAITQAGRMLMFPVDSL |
| SRR1288356 | GYGFVCTFNDLVARNRAGKALITLPENAHVMPPLVIEDEHDMLLAITQAGRMLMFPVDSL |
| SRR7426190 | GYGFVCTFNDLVARNRAGKALITLPENAHVMPPLVIEDEHDMLLAITQAGRMLMFPVDSL |
| SRR7426192 | GYGFVCTFNDLVARNRAGKALITLPENAHVMPPLVIEDEHDMLLAITQAGRMLMFPVDSL |
| SRR7426193 | GYGFVCTFNDLVARNRAGKALITLPENAHVMPPLVIEDEHDMLLAITQAGRMLMFPVDSL |
| SRR7441832 | GYGFVCTFNDLVARNRAGKALITLPENAHVMPPLVIEDEHDMLLAITQAGRMLMFPVDSL |
| SRR7426179 | GYGFVCTFNDLVARNRAGKALITLPENAHVMPPLVIEDEHDMLLAITQAGRMLMFPVDSL |
| SRR7439238 | GYGFVCTFNDLVARNRAGKALITLPENAHVMPPLVIEDEHDMLLAITQAGRMLMFPVDSL |
| SRR7439244 | GYGFVCTFNDLVARNRAGKALITLPENAHVMPPLVIEDEHDMLLAITQAGRMLMFPVDSL |
| SRR7439259 | GYGFVCTFNDLVARNRAGKALITLPENAHVMPPLVIEDEHDMLLAITQAGRMLMFPVDSL |
| SRR7439260 | GYGFVCTFNDLVARNRAGKALITLPENAHVMPPLVIEDEHDMLLAITQAGRMLMFPVDSL |
| SRR7441786 | GYGFVCTFNDLVARNRAGKALITLPENAHVMPPLVIEDEHDMLLAITQAGRMLMFPVDSL |
| SRR7441797 | GYGFVCTFNDLVARNRAGKALITLPENAHVMPPLVIEDEHDMLLAITQAGRMLMFPVDSL |
| ERR1759093 | GYGFVCTFNDLVARNRAGKALITLPENAHVMPPLVIEDEHDMLLAITQAGRMLMFPVDSL |
| ERR2580275 | GYGFVCTFNDLVARNRAGKALITLPENAHVMPPLVIEDEHDMLLAITQAGRMLMFPVDSL |
| ERR1759204 | GYGFVCTFNDLVARNRAGKALITLPENAHVMPPLVIEDEHDMLLAITQAGRMLMFPVDSL |
| SRR1300699 | GYGFVCTFNDLVARNRAGKALITLPENAHVMPPLVIEDEHDMLLAITQAGRMLMFPVDSL |
| S_0825_17 | GYGFVCTFNDLVARNRAGKALITLPENAHVMPPLVIEDEHDMLLAITQAGRMLMFPVDSL |
| SRR1958215 | GYGFVCTFNDLVARNRAGKALITLPENAHVMPPLVIEDEHDMLLAITQAGRMLMFPVDSL |
| SRR1958540 | GYGFVCTFNDLVARNRAGKALITLPENAHVMPPLVIEDEHDMLLAITQAGRMLMFPVDSL |
| SRR1958636 | GYGFVCTFNDLVARNRAGKALITLPENAHVMPPLVIEDEHDMLLAITQAGRMLMFPVDSL |
| SRR1959422 | GYGFVCTFNDLVARNRAGKALITLPENAHVMPPLVIEDEHDMLLAITQAGRMLMFPVDSL |
| SRR1959427 | GYGFVCTFNDLVARNRAGKALITLPENAHVMPPLVIEDEHDMLLAITQAGRMLMFPVDSL |
| SRR1960226 | GYGFVCTFNDLVARNRAGKALITLPENAHVMPPLVIEDEHDMLLAITQAGRMLMFPVDSL |
| SRR1963498 | GYGFVCTFNDLVARNRAGKALITLPENAHVMPPLVIEDEHDMLLAITQAGRMLMFPVDSL |
| SRR1965947 | GYGFVCTFNDLVARNRAGKALITLPENAHVMPPLVIEDEHDMLLAITQAGRMLMFPVDSL |
| SRR1966125 | GYGFVCTFNDLVARNRAGKALITLPENAHVMPPLVIEDEHDMLLAITQAGRMLMFPVDSL |
| SRR1966330 | GYGFVCTFNDLVARNRAGKALITLPENAHVMPPLVIEDEHDMLLAITQAGRMLMFPVDSL |
| SRR1966565 | GYGFVCTFNDLVARNRAGKALITLPENAHVMPPLVIEDEHDMLLAITQAGRMLMFPVDSL |
| SRR1966864 | GYGFVCTFNDLVARNRAGKALITLPENAHVMPPLVIEDEHDMLLAITQAGRMLMFPVDSL |
| SRR1966989 | GYGFVCTFNDLVARNRAGKALITLPENAHVMPPLVIEDEHDMLLAITQAGRMLMFPVDSL |
| SRR1967688 | GYGFVCTFNDLVARNRAGKALITLPENAHVMPPLVIEDEHDMLLAITQAGRMLMFPVDSL |
| SRR1967733 | GYGFVCTFNDLVARNRAGKALITLPENAHVMPPLVIEDEHDMLLAITQAGRMLMFPVDSL |
| SRR1967746 | GYGFVCTFNDLVARNRAGKALITLPENAHVMPPLVIEDEHDMLLAITQAGRMLMFPVDSL |
| SRR1968341 | GYGFVCTFNDLVARNRAGKALITLPENAHVMPPLVIEDEHDMLLAITQAGRMLMFPVDSL |
| SRR1968456 | GYGFVCTFNDLVARNRAGKALITLPENAHVMPPLVIEDEHDMLLAITQAGRMLMFPVDSL |
| SRR1968465 | GYGFVCTFNDLVARNRAGKALITLPENAHVMPPLVIEDEHDMLLAITQAGRMLMFPVDSL |
| SRR1968761 | GYGFVCTFNDLVARNRAGKALITLPENAHVMPPLVIEDEHDMLLAITQAGRMLMFPVDSL |
| SRR1969047 | GYGFVCTFNDLVARNRAGKALITLPENAHVMPPLVIEDEHDMLLAITQAGRMLMFPVDSL |
| SRR1969255 | GYGFVCTFNDLVARNRAGKALITLPENAHVMPPLVIEDEHDMLLAITQAGRMLMFPVDSL |
| SRR1969412 | GYGFVCTFNDLVARNRAGKALITLPENAHVMPPLVIEDEHDMLLAITQAGRMLMFPVDSL |
| SRR1969524 | GYGFVCTFNDLVARNRAGKALITLPENAHVMPPLVIEDEHDMLLAITQAGRMLMFPVDSL |
| SRR1969584 | GYGFVCTFNDLVARNRAGKALITLPENAHVMPPLVIEDEHDMLLAITQAGRMLMFPVDSL |
| SRR1969648 | GYGFVCTFNDLVARNRAGKALITLPENAHVMPPLVIEDEHDMLLAITQAGRMLMFPVDSL |
| SRR1969804 | GYGFVCTFNDLVARNRAGKALITLPENAHVMPPLVIEDEHDMLLAITQAGRMLMFPVDSL |
| SRR1970221 | GYGFVCTFNDLVARNRAGKALITLPENAHVMPPLVIEDEHDMLLAITQAGRMLMFPVDSL |
| SRR1970268 | GYGFVCTFNDLVARNRAGKALITLPENAHVMPPLVIEDEHDMLLAITQAGRMLMFPVDSL |
| SRR1965862 | GYGFVCTFNDLVARNRAGKALITLPENAHVMPPLVIEDEHDMLLAITQAGRMLMFPVDSL |
| SRR1967363 | GYGFVCTFNDLVARNRAGKALITLPENAHVMPPLVIEDEHDMLLAITQAGRMLMFPVDSL |
| SRR1968276 | GYGFVCTFNDLVARNRAGKALITLPENAHVMPPLVIEDEHDMLLAITQAGRMLMFPVDSL |
| SRR1968967 | GYGFVCTFNDLVARNRAGKALITLPENAHVMPPLVIEDEHDMLLAITQAGRMLMFPVDSL |
| SRR3321531 | GYGFVCTFNDLVARNRAGKALITLPENAHVMPPLVIEDEHDMLLAITQAGRMLMFPVDSL |
| SRR3321883 | GYGFVCTFNDLVARNRAGKALITLPENAHVMPPLVIEDEHDMLLAITQAGRMLMFPVDSL |
| SRR3322413 | GYGFVCTFNDLVARNRAGKALITLPENAHVMPPLVIEDEHDMLLAITQAGRMLMFPVDSL |
| SRR3323012 | GYGFVCTFNDLVARNRAGKALITLPENAHVMPPLVIEDEHDMLLAITQAGRMLMFPVDSL |
| SRR5194289 | GYGFVCTFNDLVARNRAGKALITLPENAHVMPPLVIEDEHDMLLAITQAGRMLMFPVDSL |
| SRR7163798 | GYGFVCTFNDLVARNRAGKALITLPENAHVMPPLVIEDEHDMLLAITQAGRMLMFPVDSL |
| SRR7172610 | GYGFVCTFNDLVARNRAGKALITLPENAHVMPPLVIEDEHDMLLAITQAGRMLMFPVDSL |
| SRR7204568 | GYGFVCTFNDLVARNRAGKALITLPENAHVMPPLVIEDEHDMLLAITQAGRMLMFPVDSL |
| SRR7223230 | GYGFVCTFNDLVARNRAGKALITLPENAHVMPPLVIEDEHDMLLAITQAGRMLMFPVDSL |
| SRR7230675 | GYGFVCTFNDLVARNRAGKALITLPENAHVMPPLVIEDEHDMLLAITQAGRMLMFPVDSL |
| SRR7278056 | GYGFVCTFNDLVARNRAGKALITLPENAHVMPPLVIEDEHDMLLAITQAGRMLMFPVDSL |
| SRR7278086 | GYGFVCTFNDLVARNRAGKALITLPENAHVMPPLVIEDEHDMLLAITQAGRMLMFPVDSL |
| SRR7285841 | GYGFVCTFNDLVARNRAGKALITLPENAHVMPPLVIEDEHDMLLAITQAGRMLMFPVDSL |
| SRR7292625 | GYGFVCTFNDLVARNRAGKALITLPENAHVMPPLVIEDEHDMLLAITQAGRMLMFPVDSL |
| SRR7292665 | GYGFVCTFNDLVARNRAGKALITLPENAHVMPPLVIEDEHDMLLAITQAGRMLMFPVDSL |
| SRR7297965 | GYGFVCTFNDLVARNRAGKALITLPENAHVMPPLVIEDEHDMLLAITQAGRMLMFPVDSL |
| SRR7350726 | GYGFVCTFNDLVARNRAGKALITLPENAHVMPPLVIEDEHDMLLAITQAGRMLMFPVDSL |
| SRR7410328 | GYGFVCTFNDLVARNRAGKALITLPENAHVMPPLVIEDEHDMLLAITQAGRMLMFPVDSL |
| SRR7474665 | GYGFVCTFNDLVARNRAGKALITLPENAHVMPPLVIEDEHDMLLAITQAGRMLMFPVDSL |
| SRR7523184 | GYGFVCTFNDLVARNRAGKALITLPENAHVMPPLVIEDEHDMLLAITQAGRMLMFPVDSL |
| SRR7187264 | GYGFVCTFNDLVARNRAGKALITLPENAHVMPPLVIEDEHDMLLAITQAGRMLMFPVDSL |
| SRR7204445 | GYGFVCTFNDLVARNRAGKALITLPENAHVMPPLVIEDEHDMLLAITQAGRMLMFPVDSL |
| SRR7285641 | GYGFVCTFNDLVARNRAGKALITLPENAHVMPPLVIEDEHDMLLAITQAGRMLMFPVDSL |
| SRR7286695 | GYGFVCTFNDLVARNRAGKALITLPENAHVMPPLVIEDEHDMLLAITQAGRMLMFPVDSL |
| SRR7286705 | GYGFVCTFNDLVARNRAGKALITLPENAHVMPPLVIEDEHDMLLAITQAGRMLMFPVDSL |
| SRR7292931 | GYGFVCTFNDLVARNRAGKALITLPENAHVMPPLVIEDEHDMLLAITQAGRMLMFPVDSL |
| SRR7310349 | GYGFVCTFNDLVARNRAGKALITLPENAHVMPPLVIEDEHDMLLAITQAGRMLMFPVDSL |
| SRR7351616 | GYGFVCTFNDLVARNRAGKALITLPENAHVMPPLVIEDEHDMLLAITQAGRMLMFPVDSL |
| SRR7414818 | GYGFVCTFNDLVARNRAGKALITLPENAHVMPPLVIEDEHDMLLAITQAGRMLMFPVDSL |
| SRR7426480 | GYGFVCTFNDLVARNRAGKALITLPENAHVMPPLVIEDEHDMLLAITQAGRMLMFPVDSL |
| SRR5584105 | GYGFVCTFNDLVARNRAGKALITLPENAHVMPPLVIEDEHDMLLAITQAGRMLMFPVDSL |
| SRR5584565 | GYGFVCTFNDLVARNRAGKALITLPENAHVMPPLVIEDEHDMLLAITQAGRMLMFPVDSL |
| SRR5584614 | GYGFVCTFNDLVARNRAGKALITLPENAHVMPPLVIEDEHDMLLAITQAGRMLMFPVDSL |
| SRR5631543 | GYGFVCTFNDLVARNRAGKALITLPENAHVMPPLVIEDEHDMLLAITQAGRMLMFPVDSL |
| SRR5631553 | GYGFVCTFNDLVARNRAGKALITLPENAHVMPPLVIEDEHDMLLAITQAGRMLMFPVDSL |
| SRR7123196 | GYGFVCTFNDLVARNRAGKALITLPENAHVMPPLVIEDEHDMLLAITQAGRMLMFPVDSL |
| SRR7163819 | GYGFVCTFNDLVARNRAGKALITLPENAHVMPPLVIEDEHDMLLAITQAGRMLMFPVDSL |
| SRR7163920 | GYGFVCTFNDLVARNRAGKALITLPENAHVMPPLVIEDEHDMLLAITQAGRMLMFPVDSL |
| SRR7209528 | GYGFVCTFNDLVARNRAGKALITLPENAHVMPPLVIEDEHDMLLAITQAGRMLMFPVDSL |
| SRR7249868 | GYGFVCTFNDLVARNRAGKALITLPENAHVMPPLVIEDEHDMLLAITQAGRMLMFPVDSL |
| SRR7278088 | GYGFVCTFNDLVARNRAGKALITLPENAHVMPPLVIEDEHDMLLAITQAGRMLMFPVDSL |
| SRR7285788 | GYGFVCTFNDLVARNRAGKALITLPENAHVMPPLVIEDEHDMLLAITQAGRMLMFPVDSL |
| SRR7286789 | GYGFVCTFNDLVARNRAGKALITLPENAHVMPPLVIEDEHDMLLAITQAGRMLMFPVDSL |
| SRR7286886 | GYGFVCTFNDLVARNRAGKALITLPENAHVMPPLVIEDEHDMLLAITQAGRMLMFPVDSL |
| SRR7310632 | GYGFVCTFNDLVARNRAGKALITLPENAHVMPPLVIEDEHDMLLAITQAGRMLMFPVDSL |
| SRR7350631 | GYGFVCTFNDLVARNRAGKALITLPENAHVMPPLVIEDEHDMLLAITQAGRMLMFPVDSL |
| SRR7458741 | GYGFVCTFNDLVARNRAGKALITLPENAHVMPPLVIEDEHDMLLAITQAGRMLMFPVDSL |
| SRR7480280 | GYGFVCTFNDLVARNRAGKALITLPENAHVMPPLVIEDEHDMLLAITQAGRMLMFPVDSL |
| SRR7523660 | GYGFVCTFNDLVARNRAGKALITLPENAHVMPPLVIEDEHDMLLAITQAGRMLMFPVDSL |
| SRR7523775 | GYGFVCTFNDLVARNRAGKALITLPENAHVMPPLVIEDEHDMLLAITQAGRMLMFPVDSL |
| SRR7251101 | GYGFVCTFNDLVARNRAGKALITLPENAHVMPPLVIEDEHDMLLAITQAGRMLMFPVDSL |
| SRR7284299 | GYGFVCTFNDLVARNRAGKALITLPENAHVMPPLVIEDEHDMLLAITQAGRMLMFPVDSL |
| SRR7285738 | GYGFVCTFNDLVARNRAGKALITLPENAHVMPPLVIEDEHDMLLAITQAGRMLMFPVDSL |
| SRR7310640 | GYGFVCTFNDLVARNRAGKALITLPENAHVMPPLVIEDEHDMLLAITQAGRMLMFPVDSL |
| SRR7349159 | GYGFVCTFNDLVARNRAGKALITLPENAHVMPPLVIEDEHDMLLAITQAGRMLMFPVDSL |
| SRR7474873 | GYGFVCTFNDLVARNRAGKALITLPENAHVMPPLVIEDEHDMLLAITQAGRMLMFPVDSL |
| SRR7495689 | GYGFVCTFNDLVARNRAGKALITLPENAHVMPPLVIEDEHDMLLAITQAGRMLMFPVDSL |
| SRR7495752 | GYGFVCTFNDLVARNRAGKALITLPENAHVMPPLVIEDEHDMLLAITQAGRMLMFPVDSL |
| ------------------------------------------------------------------------------ | |
| S16BD08730 | PQLSKGKGNKIINIPSAEAAKGDDGLAHLYVLPPQSTLTIHVGKRKIKLRPEELQKVVGE |
| S18BD00684 | PQLSKGKGNKIINIPSAEAAKGDDGLAHLYVLPPQSTLTIHVGKRKIKLRPEELQKVVGE |
| S18BD03994 | PQLSKGKGNKIINIPSAEAAKGDDGLAHLYVLPPQSTLTIHVGKRKIKLRPEELQKVVGE |
| S18BD05011 | PQLSKGKGNKIINIPSAEAAKGDDGLAHLYVLPPQSTLTIHVGKRKIKLRPEELQKVVGE |
| RKI_16-03723 | PQLSKGKGNKIINIPSAEAAKGDDGLAHLYVLPPQSTLTIHVGKRKIKLRPEELQKVVGE |
| RKI_16-04315 | PQLSKGKGNKIINIPSAEAAKGDDGLAHLYVLPPQSTLTIHVGKRKIKLRPEELQKVVGE |
| RKI_17-02304 | PQLSKGKGNKIINIPSAEAAKGDDGLAHLYVLPPQSTLTIHVGKRKIKLRPEELQKVVGE |
| RKI_17-02411 | PQLSKGKGNKIINIPSAEAAKGDDGLAHLYVLPPQSTLTIHVGKRKIKLRPEELQKVVGE |
| RKI_17-02757 | PQLSKGKGNKIINIPSAEAAKGDDGLAHLYVLPPQSTLTIHVGKRKIKLRPEELQKVVGE |
| RKI_17-04797 | PQLSKGKGNKIINIPSAEAAKGDDGLAHLYVLPPQSTLTIHVGKRKIKLRPEELQKVVGE |
| RKI_17-06869 | PQLSKGKGNKIINIPSAEAAKGDDGLAHLYVLPPQSTLTIHVGKRKIKLRPEELQKVVGE |
| ERR2580277 | PQLSKGKGNKIINIPSAEAAKGDDGLAHLYVLPPQSTLTIHVGKRKIKLRPEELQKVVGE |
| ERR2580276 | PQLSKGKGNKIINIPSAEAAKGDDGLAHLYVLPPQSTLTIHVGKRKIKLRPEELQKVVGE |
| ERR2580273 | PQLSKGKGNKIINIPSAEAAKGDDGLAHLYVLPPQSTLTIHVGKRKIKLRPEELQKVVGE |
| ERR2580274 | PQLSKGKGNKIINIPSAEAAKGDDGLAHLYVLPPQSTLTIHVGKRKIKLRPEELQKVVGE |
| ERR2173656 | PQLSKGKGNKIINIPSAEAAKGDDGLAHLYVLPPQSTLTIHVGKRKIKLRPEELQKVVGE |
| 17041676 | PQLSKGKGNKIINIPSAEAAKGDDGLAHLYVLPPQSTLTIHVGKRKIKLRPEELQKVVGE |
| MT16-000061 | PQLSKGKGNKIINIPSAEAAKGDDGLAHLYVLPPQSTLTIHVGKRKIKLRPEELQKVVGE |
| MT16-019416 | PQLSKGKGNKIINIPSAEAAKGDDGLAHLYVLPPQSTLTIHVGKRKIKLRPEELQKVVGE |
| MT16-027865 | PQLSKGKGNKIINIPSAEAAKGDDGLAHLYVLPPQSTLTIHVGKRKIKLRPEELQKVVGE |
| MT16-031693 | PQLSKGKGNKIINIPSAEAAKGDDGLAHLYVLPPQSTLTIHVGKRKIKLRPEELQKVVGE |
| MT16-040253 | PQLSKGKGNKIINIPSAEAAKGDDGLAHLYVLPPQSTLTIHVGKRKIKLRPEELQKVVGE |
| MT16-045379 | PQLSKGKGNKIINIPSAEAAKGDDGLAHLYVLPPQSTLTIHVGKRKIKLRPEELQKVVGE |
| MT16-442728 | PQLSKGKGNKIINIPSAEAAKGDDGLAHLYVLPPQSTLTIHVGKRKIKLRPEELQKVVGE |
| MT16-462857 | PQLSKGKGNKIINIPSAEAAKGDDGLAHLYVLPPQSTLTIHVGKRKIKLRPEELQKVVGE |
| MT16-480196 | PQLSKGKGNKIINIPSAEAAKGDDGLAHLYVLPPQSTLTIHVGKRKIKLRPEELQKVVGE |
| MT16-861555 | PQLSKGKGNKIINIPSAEAAKGDDGLAHLYVLPPQSTLTIHVGKRKIKLRPEELQKVVGE |
| MT17-076833 | PQLSKGKGNKIINIPSAEAAKGDDGLAHLYVLPPQSTLTIHVGKRKIKLRPEELQKVVGE |
| MT17-110677 | PQLSKGKGNKIINIPSAEAAKGDDGLAHLYVLPPQSTLTIHVGKRKIKLRPEELQKVVGE |
| MT17-131730 | PQLSKGKGNKIINIPSAEAAKGDDGLAHLYVLPPQSTLTIHVGKRKIKLRPEELQKVVGE |
| MT17-140890 | PQLSKGKGNKIINIPSAEAAKGDDGLAHLYVLPPQSTLTIHVGKRKIKLRPEELQKVVGE |
| MT17-141840 | PQLSKGKGNKIINIPSAEAAKGDDGLAHLYVLPPQSTLTIHVGKRKIKLRPEELQKVVGE |
| MT17-152488 | PQLSKGKGNKIINIPSAEAAKGDDGLAHLYVLPPQSTLTIHVGKRKIKLRPEELQKVVGE |
| MT17-157311 | PQLSKGKGNKIINIPSAEAAKGDDGLAHLYVLPPQSTLTIHVGKRKIKLRPEELQKVVGE |
| MT17-161645 | PQLSKGKGNKIINIPSAEAAKGDDGLAHLYVLPPQSTLTIHVGKRKIKLRPEELQKVVGE |
| MT17-167951 | PQLSKGKGNKIINIPSAEAAKGDDGLAHLYVLPPQSTLTIHVGKRKIKLRPEELQKVVGE |
| MT18-217732 | PQLSKGKGNKIINIPSAEAAKGDDGLAHLYVLPPQSTLTIHVGKRKIKLRPEELQKVVGE |
| MT18-252580 | PQLSKGKGNKIINIPSAEAAKGDDGLAHLYVLPPQSTLTIHVGKRKIKLRPEELQKVVGE |
| RIVM_H_2009-01 | PQLSKGKGNKIINIPSAEAAKGDDGLAHLYVLPPQSTLTIHVGKRKIKLRPEELQKVVGE |
| RIVM_H_2010-01 | PQLSKGKGNKIINIPSAEAAKGDDGLAHLYVLPPQSTLTIHVGKRKIKLRPEELQKVVGE |
| RIVM_H_2010-02 | PQLSKGKGNKIINIPSAEAAKGDDGLAHLYVLPPQSTLTIHVGKRKIKLRPEELQKVVGE |
| RIVM_H_2011-01 | PQLSKGKGNKIINIPSAEAAKGDDGLAHLYVLPPQSTLTIHVGKRKIKLRPEELQKVVGE |
| RIVM_H_2011-02 | PQLSKGKGNKIINIPSAEAAKGDDGLAHLYVLPPQSTLTIHVGKRKIKLRPEELQKVVGE |
| RIVM_H_2011-03 | PQLSKGKGNKIINIPSAEAAKGDDGLAHLYVLPPQSTLTIHVGKRKIKLRPEELQKVVGE |
| RIVM_H_2013-01 | PQLSKGKGNKIINIPSAEAAKGDDGLAHLYVLPPQSTLTIHVGKRKIKLRPEELQKVVGE |
| RIVM_H_2013-02 | PQLSKGKGNKIINIPSAEAAKGDDGLAHLYVLPPQSTLTIHVGKRKIKLRPEELQKVVGE |
| RIVM_H_2014-01 | PQLSKGKGNKIINIPSAEAAKGDDGLAHLYVLPPQSTLTIHVGKRKIKLRPEELQKVVGE |
| RIVM_H_2014-02 | PQLSKGKGNKIINIPSAEAAKGDDGLAHLYVLPPQSTLTIHVGKRKIKLRPEELQKVVGE |
| RIVM_H_2016-01 | PQLSKGKGNKIINIPSAEAAKGDDGLAHLYVLPPQSTLTIHVGKRKIKLRPEELQKVVGE |
| RIVM_H_2016-02 | PQLSKGKGNKIINIPSAEAAKGDDGLAHLYVLPPQSTLTIHVGKRKIKLRPEELQKVVGE |
| RIVM_H_2016-03 | PQLSKGKGNKIINIPSAEAAKGDDGLAHLYVLPPQSTLTIHVGKRKIKLRPEELQKVVGE |
| RIVM_H_2016-04 | PQLSKGKGNKIINIPSAEAAKGDDGLAHLYVLPPQSTLTIHVGKRKIKLRPEELQKVVGE |
| RIVM_H_2016-05 | PQLSKGKGNKIINIPSAEAAKGDDGLAHLYVLPPQSTLTIHVGKRKIKLRPEELQKVVGE |
| RIVM_H_2016-06 | PQLSKGKGNKIINIPSAEAAKGDDGLAHLYVLPPQSTLTIHVGKRKIKLRPEELQKVVGE |
| RIVM_H_2016-07 | PQLSKGKGNKIINIPSAEAAKGDDGLAHLYVLPPQSTLTIHVGKRKIKLRPEELQKVVGE |
| RIVM_H_2016-08 | PQLSKGKGNKIINIPSAEAAKGDDGLAHLYVLPPQSTLTIHVGKRKIKLRPEELQKVVGE |
| RIVM_H_2016-09 | PQLSKGKGNKIINIPSAEAAKGDDGLAHLYVLPPQSTLTIHVGKRKIKLRPEELQKVVGE |
| RIVM_H_2016-10 | PQLSKGKGNKIINIPSAEAAKGDDGLAHLYVLPPQSTLTIHVGKRKIKLRPEELQKVVGE |
| RIVM_H_2016-11 | PQLSKGKGNKIINIPSAEAAKGDDGLAHLYVLPPQSTLTIHVGKRKIKLRPEELQKVVGE |
| RIVM_H_2016-12 | PQLSKGKGNKIINIPSAEAAKGDDGLAHLYVLPPQSTLTIHVGKRKIKLRPEELQKVVGE |
| RIVM_H_2016-13 | PQLSKGKGNKIINIPSAEAAKGDDGLAHLYVLPPQSTLTIHVGKRKIKLRPEELQKVVGE |
| RIVM_H_2016-14 | PQLSKGKGNKIINIPSAEAAKGDDGLAHLYVLPPQSTLTIHVGKRKIKLRPEELQKVVGE |
| RIVM_H_2016-15 | PQLSKGKGNKIINIPSAEAAKGDDGLAHLYVLPPQSTLTIHVGKRKIKLRPEELQKVVGE |
| RIVM_H_2017-01 | PQLSKGKGNKIINIPSAEAAKGDDGLAHLYVLPPQSTLTIHVGKRKIKLRPEELQKVVGE |
| RIVM_H_2017-02 | PQLSKGKGNKIINIPSAEAAKGDDGLAHLYVLPPQSTLTIHVGKRKIKLRPEELQKVVGE |
| RIVM_H_2017-03 | PQLSKGKGNKIINIPSAEAAKGDDGLAHLYVLPPQSTLTIHVGKRKIKLRPEELQKVVGE |
| RIVM_H_2017-04 | PQLSKGKGNKIINIPSAEAAKGDDGLAHLYVLPPQSTLTIHVGKRKIKLRPEELQKVVGE |
| RIVM_H_2017-05 | PQLSKGKGNKIINIPSAEAAKGDDGLAHLYVLPPQSTLTIHVGKRKIKLRPEELQKVVGE |
| RIVM_H_2017-06 | PQLSKGKGNKIINIPSAEAAKGDDGLAHLYVLPPQSTLTIHVGKRKIKLRPEELQKVVGE |
| RIVM_H_2017-07 | PQLSKGKGNKIINIPSAEAAKGDDGLAHLYVLPPQSTLTIHVGKRKIKLRPEELQKVVGE |
| RIVM_H_2017-08 | PQLSKGKGNKIINIPSAEAAKGDDGLAHLYVLPPQSTLTIHVGKRKIKLRPEELQKVVGE |
| RIVM_H_2017-09 | PQLSKGKGNKIINIPSAEAAKGDDGLAHLYVLPPQSTLTIHVGKRKIKLRPEELQKVVGE |
| RIVM_H_2017-10 | PQLSKGKGNKIINIPSAEAAKGDDGLAHLYVLPPQSTLTIHVGKRKIKLRPEELQKVVGE |
| RIVM_H_2017-11 | PQLSKGKGNKIINIPSAEAAKGDDGLAHLYVLPPQSTLTIHVGKRKIKLRPEELQKVVGE |
| RIVM_H_2017-12 | PQLSKGKGNKIINIPSAEAAKGDDGLAHLYVLPPQSTLTIHVGKRKIKLRPEELQKVVGE |
| RIVM_H_2017-13 | PQLSKGKGNKIINIPSAEAAKGDDGLAHLYVLPPQSTLTIHVGKRKIKLRPEELQKVVGE |
| RIVM_H_2017-14 | PQLSKGKGNKIINIPSAEAAKGDDGLAHLYVLPPQSTLTIHVGKRKIKLRPEELQKVVGE |
| RIVM_H_2017-15 | PQLSKGKGNKIINIPSAEAAKGDDGLAHLYVLPPQSTLTIHVGKRKIKLRPEELQKVVGE |
| RIVM_H_2017-16 | PQLSKGKGNKIINIPSAEAAKGDDGLAHLYVLPPQSTLTIHVGKRKIKLRPEELQKVVGE |
| RIVM_H_2017-17 | PQLSKGKGNKIINIPSAEAAKGDDGLAHLYVLPPQSTLTIHVGKRKIKLRPEELQKVVGE |
| RIVM_H_2017-18 | PQLSKGKGNKIINIPSAEAAKGDDGLAHLYVLPPQSTLTIHVGKRKIKLRPEELQKVVGE |
| RIVM_H_2017-19 | PQLSKGKGNKIINIPSAEAAKGDDGLAHLYVLPPQSTLTIHVGKRKIKLRPEELQKVVGE |
| 15EP001483 | PQLSKGKGNKIINIPSAEAAKGDDGLAHLYVLPPQSTLTIHVGKRKIKLRPEELQKVVGE |
| 17EP002363 | PQLSKGKGNKIINIPSAEAAKGDDGLAHLYVLPPQSTLTIHVGKRKIKLRPEELQKVVGE |
| S_0812_17 | PQLSKGKGNKIINIPSAEAAKGDDGLAHLYVLPPQSTLTIHVGKRKIKLRPEELQKVVGE |
| SRR1957844 | PQLSKGKGNKIINIPSAEAAKGDDGLAHLYVLPPQSTLTIHVGKRKIKLRPEELQKVVGE |
| SRR1958654 | PQLSKGKGNKIINIPSAEAAKGDDGLAHLYVLPPQSTLTIHVGKRKIKLRPEELQKVVGE |
| SRR1965077 | PQLSKGKGNKIINIPSAEAAKGDDGLAHLYVLPPQSTLTIHVGKRKIKLRPEELQKVVGE |
| SRR1966369 | PQLSKGKGNKIINIPSAEAAKGDDGLAHLYVLPPQSTLTIHVGKRKIKLRPEELQKVVGE |
| SRR1967117 | PQLSKGKGNKIINIPSAEAAKGDDGLAHLYVLPPQSTLTIHVGKRKIKLRPEELQKVVGE |
| SRR1967922 | PQLSKGKGNKIINIPSAEAAKGDDGLAHLYVLPPQSTLTIHVGKRKIKLRPEELQKVVGE |
| SRR8704720 | PQLSKGKGNKIINIPSAEAAKGDDGLAHLYVLPPQSTLTIHVGKRKIKLRPEELQKVVGE |
| SRR7216071 | PQLSKGKGNKIINIPSAEAAKGDDGLAHLYVLPPQSTLTIHVGKRKIKLRPEELQKVVGE |
| SRR7349175 | PQLSKGKGNKIINIPSAEAAKGDDGLAHLYVLPPQSTLTIHVGKRKIKLRPEELQKVVGE |
| SRR7523148 | PQLSKGKGNKIINIPSAEAAKGDDGLAHLYVLPPQSTLTIHVGKRKIKLRPEELQKVVGE |
| SRR7523854 | PQLSKGKGNKIINIPSAEAAKGDDGLAHLYVLPPQSTLTIHVGKRKIKLRPEELQKVVGE |
| 313865 | PQLSKGKGNKIINIPSAEAAKGDDGLAHLYVLPPQSTLTIHVGKRKIKLRPEELQKVVGE |
| SRR7277793 | PQLSKGKGNKIINIPSAEAAKGDDGLAHLYVLPPQSTLTIHVGKRKIKLRPEELQKVVGE |
| SRR7343877 | PQLSKGKGNKIINIPSAEAAKGDDGLAHLYVLPPQSTLTIHVGKRKIKLRPEELQKVVGE |
| SRR7351477 | PQLSKGKGNKIINIPSAEAAKGDDGLAHLYVLPPQSTLTIHVGKRKIKLRPEELQKVVGE |
| SRR5583183 | PQLSKGKGNKIINIPSAEAAKGDDGLAHLYVLPPQSTLTIHVGKRKIKLRPEELQKVVGE |
| SRR5585240 | PQLSKGKGNKIINIPSAEAAKGDDGLAHLYVLPPQSTLTIHVGKRKIKLRPEELQKVVGE |
| SRR7284317 | PQLSKGKGNKIINIPSAEAAKGDDGLAHLYVLPPQSTLTIHVGKRKIKLRPEELQKVVGE |
| SRR7299161 | PQLSKGKGNKIINIPSAEAAKGDDGLAHLYVLPPQSTLTIHVGKRKIKLRPEELQKVVGE |
| SRR7401730 | PQLSKGKGNKIINIPSAEAAKGDDGLAHLYVLPPQSTLTIHVGKRKIKLRPEELQKVVGE |
| SRR7469092 | PQLSKGKGNKIINIPSAEAAKGDDGLAHLYVLPPQSTLTIHVGKRKIKLRPEELQKVVGE |
| SRR7879556 | PQLSKGKGNKIINIPSAEAAKGDDGLAHLYVLPPQSTLTIHVGKRKIKLRPEELQKVVGE |
| SRR8526100 | PQLSKGKGNKIINIPSAEAAKGDDGLAHLYVLPPQSTLTIHVGKRKIKLRPEELQKVVGE |
| SRR8553991 | PQLSKGKGNKIINIPSAEAAKGDDGLAHLYVLPPQSTLTIHVGKRKIKLRPEELQKVVGE |
| SRR7842487 | PQLSKGKGNKIINIPSAEAAKGDDGLAHLYVLPPQSTLTIHVGKRKIKLRPEELQKVVGE |
| SRR8054524 | PQLSKGKGNKIINIPSAEAAKGDDGLAHLYVLPPQSTLTIHVGKRKIKLRPEELQKVVGE |
| SRR8054525 | PQLSKGKGNKIINIPSAEAAKGDDGLAHLYVLPPQSTLTIHVGKRKIKLRPEELQKVVGE |
| SRR8524733 | PQLSKGKGNKIINIPSAEAAKGDDGLAHLYVLPPQSTLTIHVGKRKIKLRPEELQKVVGE |
| SRR4093291 | PQLSKGKGNKIINIPSAEAAKGDDGLAHLYVLPPQSTLTIHVGKRKIKLRPEELQKVVGE |
| SRR4245549 | PQLSKGKGNKIINIPSAEAAKGDDGLAHLYVLPPQSTLTIHVGKRKIKLRPEELQKVVGE |
| SRR3057154 | PQLSKGKGNKIINIPSAEAAKGDDGLAHLYVLPPQSTLTIHVGKRKIKLRPEELQKVVGE |
| SRR1726150 | PQLSKGKGNKIINIPSAEAAKGDDGLAHLYVLPPQSTLTIHVGKRKIKLRPEELQKVVGE |
| SRR1996141 | PQLSKGKGNKIINIPSAEAAKGDDGLAHLYVLPPQSTLTIHVGKRKIKLRPEELQKVVGE |
| SRR1107842 | PQLSKGKGNKIINIPSAEAAKGDDGLAHLYVLPPQSTLTIHVGKRKIKLRPEELQKVVGE |
| SRR1157587 | PQLSKGKGNKIINIPSAEAAKGDDGLAHLYVLPPQSTLTIHVGKRKIKLRPEELQKVVGE |
| SRR3027706 | PQLSKGKGNKIINIPSAEAAKGDDGLAHLYVLPPQSTLTIHVGKRKIKLRPEELQKVVGE |
| SRR3027707 | PQLSKGKGNKIINIPSAEAAKGDDGLAHLYVLPPQSTLTIHVGKRKIKLRPEELQKVVGE |
| SRR3027708 | PQLSKGKGNKIINIPSAEAAKGDDGLAHLYVLPPQSTLTIHVGKRKIKLRPEELQKVVGE |
| SRR3027710 | PQLSKGKGNKIINIPSAEAAKGDDGLAHLYVLPPQSTLTIHVGKRKIKLRPEELQKVVGE |
| SRR3027711 | PQLSKGKGNKIINIPSAEAAKGDDGLAHLYVLPPQSTLTIHVGKRKIKLRPEELQKVVGE |
| SRR3027716 | PQLSKGKGNKIINIPSAEAAKGDDGLAHLYVLPPQSTLTIHVGKRKIKLRPEELQKVVGE |
| SRR3027717 | PQLSKGKGNKIINIPSAEAAKGDDGLAHLYVLPPQSTLTIHVGKRKIKLRPEELQKVVGE |
| SRR3027719 | PQLSKGKGNKIINIPSAEAAKGDDGLAHLYVLPPQSTLTIHVGKRKIKLRPEELQKVVGE |
| SRR3027721 | PQLSKGKGNKIINIPSAEAAKGDDGLAHLYVLPPQSTLTIHVGKRKIKLRPEELQKVVGE |
| SRR3027723 | PQLSKGKGNKIINIPSAEAAKGDDGLAHLYVLPPQSTLTIHVGKRKIKLRPEELQKVVGE |
| SRR3115978 | PQLSKGKGNKIINIPSAEAAKGDDGLAHLYVLPPQSTLTIHVGKRKIKLRPEELQKVVGE |
| SRR2534093 | PQLSKGKGNKIINIPSAEAAKGDDGLAHLYVLPPQSTLTIHVGKRKIKLRPEELQKVVGE |
| SRR2534094 | PQLSKGKGNKIINIPSAEAAKGDDGLAHLYVLPPQSTLTIHVGKRKIKLRPEELQKVVGE |
| SRR2534095 | PQLSKGKGNKIINIPSAEAAKGDDGLAHLYVLPPQSTLTIHVGKRKIKLRPEELQKVVGE |
| SRR2534108 | PQLSKGKGNKIINIPSAEAAKGDDGLAHLYVLPPQSTLTIHVGKRKIKLRPEELQKVVGE |
| SRR1106464 | PQLSKGKGNKIINIPSAEAAKGDDGLAHLYVLPPQSTLTIHVGKRKIKLRPEELQKVVGE |
| SRR1106463 | PQLSKGKGNKIINIPSAEAAKGDDGLAHLYVLPPQSTLTIHVGKRKIKLRPEELQKVVGE |
| SRR6949610 | PQLSKGKGNKIINIPSAEAAKGDDGLAHLYVLPPQSTLTIHVGKRKIKLRPEELQKVVGE |
| SRR6950452 | PQLSKGKGNKIINIPSAEAAKGDDGLAHLYVLPPQSTLTIHVGKRKIKLRPEELQKVVGE |
| ERR2019831 | PQLSKGKGNKIINIPSAEAAKGDDGLAHLYVLPPQSTLTIHVGKRKIKLRPEELQKVVGE |
| SRR2085693 | PQLSKGKGNKIINIPSAEAAKGDDGLAHLYVLPPQSTLTIHVGKRKIKLRPEELQKVVGE |
| SRR2086898 | PQLSKGKGNKIINIPSAEAAKGDDGLAHLYVLPPQSTLTIHVGKRKIKLRPEELQKVVGE |
| SRR2175312 | PQLSKGKGNKIINIPSAEAAKGDDGLAHLYVLPPQSTLTIHVGKRKIKLRPEELQKVVGE |
| SRR2175360 | PQLSKGKGNKIINIPSAEAAKGDDGLAHLYVLPPQSTLTIHVGKRKIKLRPEELQKVVGE |
| SRR5231997 | PQLSKGKGNKIINIPSAEAAKGDDGLAHLYVLPPQSTLTIHVGKRKIKLRPEELQKVVGE |
| SRR5232003 | PQLSKGKGNKIINIPSAEAAKGDDGLAHLYVLPPQSTLTIHVGKRKIKLRPEELQKVVGE |
| SRR5232015 | PQLSKGKGNKIINIPSAEAAKGDDGLAHLYVLPPQSTLTIHVGKRKIKLRPEELQKVVGE |
| SRR949434 | PQLSKGKGNKIINIPSAEAAKGDDGLAHLYVLPPQSTLTIHVGKRKIKLRPEELQKVVGE |
| SRR3216575 | PQLSKGKGNKIINIPSAEAAKGDDGLAHLYVLPPQSTLTIHVGKRKIKLRPEELQKVVGE |
| SRR5205342 | PQLSKGKGNKIINIPSAEAAKGDDGLAHLYVLPPQSTLTIHVGKRKIKLRPEELQKVVGE |
| SRR1501669 | PQLSKGKGNKIINIPSAEAAKGDDGLAHLYVLPPQSTLTIHVGKRKIKLRPEELQKVVGE |
| SRR5209740 | PQLSKGKGNKIINIPSAEAAKGDDGLAHLYVLPPQSTLTIHVGKRKIKLRPEELQKVVGE |
| SRR3240355 | PQLSKGKGNKIINIPSAEAAKGDDGLAHLYVLPPQSTLTIHVGKRKIKLRPEELQKVVGE |
| SRR3392777 | PQLSKGKGNKIINIPSAEAAKGDDGLAHLYVLPPQSTLTIHVGKRKIKLRPEELQKVVGE |
| SRR3593671 | PQLSKGKGNKIINIPSAEAAKGDDGLAHLYVLPPQSTLTIHVGKRKIKLRPEELQKVVGE |
| SRR5413290 | PQLSKGKGNKIINIPSAEAAKGDDGLAHLYVLPPQSTLTIHVGKRKIKLRPEELQKVVGE |
| SRR5590269 | PQLSKGKGNKIINIPSAEAAKGDDGLAHLYVLPPQSTLTIHVGKRKIKLRPEELQKVVGE |
| SRR5812103 | PQLSKGKGNKIINIPSAEAAKGDDGLAHLYVLPPQSTLTIHVGKRKIKLRPEELQKVVGE |
| SRR2830941 | PQLSKGKGNKIINIPSAEAAKGDDGLAHLYVLPPQSTLTIHVGKRKIKLRPEELQKVVGE |
| SRR2830966 | PQLSKGKGNKIINIPSAEAAKGDDGLAHLYVLPPQSTLTIHVGKRKIKLRPEELQKVVGE |
| SRR3137270 | PQLSKGKGNKIINIPSAEAAKGDDGLAHLYVLPPQSTLTIHVGKRKIKLRPEELQKVVGE |
| SRR3137271 | PQLSKGKGNKIINIPSAEAAKGDDGLAHLYVLPPQSTLTIHVGKRKIKLRPEELQKVVGE |
| ERR526807 | PQLSKGKGNKIINIPSAEAAKGDDGLAHLYVLPPQSTLTIHVGKRKIKLRPEELQKVVGE |
| ERR2197922 | PQLSKGKGNKIINIPSAEAAKGDDGLAHLYVLPPQSTLTIHVGKRKIKLRPEELQKVVGE |
| ERR2197923 | PQLSKGKGNKIINIPSAEAAKGDDGLAHLYVLPPQSTLTIHVGKRKIKLRPEELQKVVGE |
| ERR2197924 | PQLSKGKGNKIINIPSAEAAKGDDGLAHLYVLPPQSTLTIHVGKRKIKLRPEELQKVVGE |
| ERR2197925 | PQLSKGKGNKIINIPSAEAAKGDDGLAHLYVLPPQSTLTIHVGKRKIKLRPEELQKVVGE |
| ERR2197927 | PQLSKGKGNKIINIPSAEAAKGDDGLAHLYVLPPQSTLTIHVGKRKIKLRPEELQKVVGE |
| ERR2197929 | PQLSKGKGNKIINIPSAEAAKGDDGLAHLYVLPPQSTLTIHVGKRKIKLRPEELQKVVGE |
| SRR1648149 | PQLSKGKGNKIINIPSAEAAKGDDGLAHLYVLPPQSTLTIHVGKRKIKLRPEELQKVVGE |
| SRR1048299 | PQLSKGKGNKIINIPSAEAAKGDDGLAHLYVLPPQSTLTIHVGKRKIKLRPEELQKVVGE |
| SRR1300677 | PQLSKGKGNKIINIPSAEAAKGDDGLAHLYVLPPQSTLTIHVGKRKIKLRPEELQKVVGE |
| SRR1288356 | PQLSKGKGNKIINIPSAEAAKGDDGLAHLYVLPPQSTLTIHVGKRKIKLRPEELQKVVGE |
| SRR7426190 | PQLSKGKGNKIINIPSAEAAKGDDGLAHLYVLPPQSTLTIHVGKRKIKLRPEELQKVVGE |
| SRR7426192 | PQLSKGKGNKIINIPSAEAAKGDDGLAHLYVLPPQSTLTIHVGKRKIKLRPEELQKVVGE |
| SRR7426193 | PQLSKGKGNKIINIPSAEAAKGDDGLAHLYVLPPQSTLTIHVGKRKIKLRPEELQKVVGE |
| SRR7441832 | PQLSKGKGNKIINIPSAEAAKGDDGLAHLYVLPPQSTLTIHVGKRKIKLRPEELQKVVGE |
| SRR7426179 | PQLSKGKGNKIINIPSAEAAKGDDGLAHLYVLPPQSTLTIHVGKRKIKLRPEELQKVVGE |
| SRR7439238 | PQLSKGKGNKIINIPSAEAAKGDDGLAHLYVLPPQSTLTIHVGKRKIKLRPEELQKVVGE |
| SRR7439244 | PQLSKGKGNKIINIPSAEAAKGDDGLAHLYVLPPQSTLTIHVGKRKIKLRPEELQKVVGE |
| SRR7439259 | PQLSKGKGNKIINIPSAEAAKGDDGLAHLYVLPPQSTLTIHVGKRKIKLRPEELQKVVGE |
| SRR7439260 | PQLSKGKGNKIINIPSAEAAKGDDGLAHLYVLPPQSTLTIHVGKRKIKLRPEELQKVVGE |
| SRR7441786 | PQLSKGKGNKIINIPSAEAAKGDDGLAHLYVLPPQSTLTIHVGKRKIKLRPEELQKVVGE |
| SRR7441797 | PQLSKGKGNKIINIPSAEAAKGDDGLAHLYVLPPQSTLTIHVGKRKIKLRPEELQKVVGE |
| ERR1759093 | PQLSKGKGNKIINIPSAEAAKGDDGLAHLYVLPPQSTLTIHVGKRKIKLRPEELQKVVGE |
| ERR2580275 | PQLSKGKGNKIINIPSAEAAKGDDGLAHLYVLPPQSTLTIHVGKRKIKLRPEELQKVVGE |
| ERR1759204 | PQLSKGKGNKIINIPSAEAAKGDDGLAHLYVLPPQSTLTIHVGKRKIKLRPEELQKVVGE |
| SRR1300699 | PQLSKGKGNKIINIPSAEAAKGDDGLAHLYVLPPQSTLTIHVGKRKIKLRPEELQKVVGE |
| S_0825_17 | PQLSKGKGNKIINIPSAEAAKGDDGLAHLYVLPPQSTLTIHVGKRKIKLRPEELQKVVGE |
| SRR1958215 | PQLSKGKGNKIINIPSAEAAKGDDGLAHLYVLPPQSTLTIHVGKRKIKLRPEELQKVVGE |
| SRR1958540 | PQLSKGKGNKIINIPSAEAAKGDDGLAHLYVLPPQSTLTIHVGKRKIKLRPEELQKVVGE |
| SRR1958636 | PQLSKGKGNKIINIPSAEAAKGDDGLAHLYVLPPQSTLTIHVGKRKIKLRPEELQKVVGE |
| SRR1959422 | PQLSKGKGNKIINIPSAEAAKGDDGLAHLYVLPPQSTLTIHVGKRKIKLRPEELQKVVGE |
| SRR1959427 | PQLSKGKGNKIINIPSAEAAKGDDGLAHLYVLPPQSTLTIHVGKRKIKLRPEELQKVVGE |
| SRR1960226 | PQLSKGKGNKIINIPSAEAAKGDDGLAHLYVLPPQSTLTIHVGKRKIKLRPEELQKVVGE |
| SRR1963498 | PQLSKGKGNKIINIPSAEAAKGDDGLAHLYVLPPQSTLTIHVGKRKIKLRPEELQKVVGE |
| SRR1965947 | PQLSKGKGNKIINIPSAEAAKGDDGLAHLYVLPPQSTLTIHVGKRKIKLRPEELQKVVGE |
| SRR1966125 | PQLSKGKGNKIINIPSAEAAKGDDGLAHLYVLPPQSTLTIHVGKRKIKLRPEELQKVVGE |
| SRR1966330 | PQLSKGKGNKIINIPSAEAAKGDDGLAHLYVLPPQSTLTIHVGKRKIKLRPEELQKVVGE |
| SRR1966565 | PQLSKGKGNKIINIPSAEAAKGDDGLAHLYVLPPQSTLTIHVGKRKIKLRPEELQKVVGE |
| SRR1966864 | PQLSKGKGNKIINIPSAEAAKGDDGLAHLYVLPPQSTLTIHVGKRKIKLRPEELQKVVGE |
| SRR1966989 | PQLSKGKGNKIINIPSAEAAKGDDGLAHLYVLPPQSTLTIHVGKRKIKLRPEELQKVVGE |
| SRR1967688 | PQLSKGKGNKIINIPSAEAAKGDDGLAHLYVLPPQSTLTIHVGKRKIKLRPEELQKVVGE |
| SRR1967733 | PQLSKGKGNKIINIPSAEAAKGDDGLAHLYVLPPQSTLTIHVGKRKIKLRPEELQKVVGE |
| SRR1967746 | PQLSKGKGNKIINIPSAEAAKGDDGLAHLYVLPPQSTLTIHVGKRKIKLRPEELQKVVGE |
| SRR1968341 | PQLSKGKGNKIINIPSAEAAKGDDGLAHLYVLPPQSTLTIHVGKRKIKLRPEELQKVVGE |
| SRR1968456 | PQLSKGKGNKIINIPSAEAAKGDDGLAHLYVLPPQSTLTIHVGKRKIKLRPEELQKVVGE |
| SRR1968465 | PQLSKGKGNKIINIPSAEAAKGDDGLAHLYVLPPQSTLTIHVGKRKIKLRPEELQKVVGE |
| SRR1968761 | PQLSKGKGNKIINIPSAEAAKGDDGLAHLYVLPPQSTLTIHVGKRKIKLRPEELQKVVGE |
| SRR1969047 | PQLSKGKGNKIINIPSAEAAKGDDGLAHLYVLPPQSTLTIHVGKRKIKLRPEELQKVVGE |
| SRR1969255 | PQLSKGKGNKIINIPSAEAAKGDDGLAHLYVLPPQSTLTIHVGKRKIKLRPEELQKVVGE |
| SRR1969412 | PQLSKGKGNKIINIPSAEAAKGDDGLAHLYVLPPQSTLTIHVGKRKIKLRPEELQKVVGE |
| SRR1969524 | PQLSKGKGNKIINIPSAEAAKGDDGLAHLYVLPPQSTLTIHVGKRKIKLRPEELQKVVGE |
| SRR1969584 | PQLSKGKGNKIINIPSAEAAKGDDGLAHLYVLPPQSTLTIHVGKRKIKLRPEELQKVVGE |
| SRR1969648 | PQLSKGKGNKIINIPSAEAAKGDDGLAHLYVLPPQSTLTIHVGKRKIKLRPEELQKVVGE |
| SRR1969804 | PQLSKGKGNKIINIPSAEAAKGDDGLAHLYVLPPQSTLTIHVGKRKIKLRPEELQKVVGE |
| SRR1970221 | PQLSKGKGNKIINIPSAEAAKGDDGLAHLYVLPPQSTLTIHVGKRKIKLRPEELQKVVGE |
| SRR1970268 | PQLSKGKGNKIINIPSAEAAKGDDGLAHLYVLPPQSTLTIHVGKRKIKLRPEELQKVVGE |
| SRR1965862 | PQLSKGKGNKIINIPSAEAAKGDDGLAHLYVLPPQSTLTIHVGKRKIKLRPEELQKVVGE |
| SRR1967363 | PQLSKGKGNKIINIPSAEAAKGDDGLAHLYVLPPQSTLTIHVGKRKIKLRPEELQKVVGE |
| SRR1968276 | PQLSKGKGNKIINIPSAEAAKGDDGLAHLYVLPPQSTLTIHVGKRKIKLRPEELQKVVGE |
| SRR1968967 | PQLSKGKGNKIINIPSAEAAKGDDGLAHLYVLPPQSTLTIHVGKRKIKLRPEELQKVVGE |
| SRR3321531 | PQLSKGKGNKIINIPSAEAAKGDDGLAHLYVLPPQSTLTIHVGKRKIKLRPEELQKVVGE |
| SRR3321883 | PQLSKGKGNKIINIPSAEAAKGDDGLAHLYVLPPQSTLTIHVGKRKIKLRPEELQKVVGE |
| SRR3322413 | PQLSKGKGNKIINIPSAEAAKGDDGLAHLYVLPPQSTLTIHVGKRKIKLRPEELQKVVGE |
| SRR3323012 | PQLSKGKGNKIINIPSAEAAKGDDGLAHLYVLPPQSTLTIHVGKRKIKLRPEELQKVVGE |
| SRR5194289 | PQLSKGKGNKIINIPSAEAAKGDDGLAHLYVLPPQSTLTIHVGKRKIKLRPEELQKVVGE |
| SRR7163798 | PQLSKGKGNKIINIPSAEAAKGDDGLAHLYVLPPQSTLTIHVGKRKIKLRPEELQKVVGE |
| SRR7172610 | PQLSKGKGNKIINIPSAEAAKGDDGLAHLYVLPPQSTLTIHVGKRKIKLRPEELQKVVGE |
| SRR7204568 | PQLSKGKGNKIINIPSAEAAKGDDGLAHLYVLPPQSTLTIHVGKRKIKLRPEELQKVVGE |
| SRR7223230 | PQLSKGKGNKIINIPSAEAAKGDDGLAHLYVLPPQSTLTIHVGKRKIKLRPEELQKVVGE |
| SRR7230675 | PQLSKGKGNKIINIPSAEAAKGDDGLAHLYVLPPQSTLTIHVGKRKIKLRPEELQKVVGE |
| SRR7278056 | PQLSKGKGNKIINIPSAEAAKGDDGLAHLYVLPPQSTLTIHVGKRKIKLRPEELQKVVGE |
| SRR7278086 | PQLSKGKGNKIINIPSAEAAKGDDGLAHLYVLPPQSTLTIHVGKRKIKLRPEELQKVVGE |
| SRR7285841 | PQLSKGKGNKIINIPSAEAAKGDDGLAHLYVLPPQSTLTIHVGKRKIKLRPEELQKVVGE |
| SRR7292625 | PQLSKGKGNKIINIPSAEAAKGDDGLAHLYVLPPQSTLTIHVGKRKIKLRPEELQKVVGE |
| SRR7292665 | PQLSKGKGNKIINIPSAEAAKGDDGLAHLYVLPPQSTLTIHVGKRKIKLRPEELQKVVGE |
| SRR7297965 | PQLSKGKGNKIINIPSAEAAKGDDGLAHLYVLPPQSTLTIHVGKRKIKLRPEELQKVVGE |
| SRR7350726 | PQLSKGKGNKIINIPSAEAAKGDDGLAHLYVLPPQSTLTIHVGKRKIKLRPEELQKVVGE |
| SRR7410328 | PQLSKGKGNKIINIPSAEAAKGDDGLAHLYVLPPQSTLTIHVGKRKIKLRPEELQKVVGE |
| SRR7474665 | PQLSKGKGNKIINIPSAEAAKGDDGLAHLYVLPPQSTLTIHVGKRKIKLRPEELQKVVGE |
| SRR7523184 | PQLSKGKGNKIINIPSAEAAKGDDGLAHLYVLPPQSTLTIHVGKRKIKLRPEELQKVVGE |
| SRR7187264 | PQLSKGKGNKIINIPSAEAAKGDDGLAHLYVLPPQSTLTIHVGKRKIKLRPEELQKVVGE |
| SRR7204445 | PQLSKGKGNKIINIPSAEAAKGDDGLAHLYVLPPQSTLTIHVGKRKIKLRPEELQKVVGE |
| SRR7285641 | PQLSKGKGNKIINIPSAEAAKGDDGLAHLYVLPPQSTLTIHVGKRKIKLRPEELQKVVGE |
| SRR7286695 | PQLSKGKGNKIINIPSAEAAKGDDGLAHLYVLPPQSTLTIHVGKRKIKLRPEELQKVVGE |
| SRR7286705 | PQLSKGKGNKIINIPSAEAAKGDDGLAHLYVLPPQSTLTIHVGKRKIKLRPEELQKVVGE |
| SRR7292931 | PQLSKGKGNKIINIPSAEAAKGDDGLAHLYVLPPQSTLTIHVGKRKIKLRPEELQKVVGE |
| SRR7310349 | PQLSKGKGNKIINIPSAEAAKGDDGLAHLYVLPPQSTLTIHVGKRKIKLRPEELQKVVGE |
| SRR7351616 | PQLSKGKGNKIINIPSAEAAKGDDGLAHLYVLPPQSTLTIHVGKRKIKLRPEELQKVVGE |
| SRR7414818 | PQLSKGKGNKIINIPSAEAAKGDDGLAHLYVLPPQSTLTIHVGKRKIKLRPEELQKVVGE |
| SRR7426480 | PQLSKGKGNKIINIPSAEAAKGDDGLAHLYVLPPQSTLTIHVGKRKIKLRPEELQKVVGE |
| SRR5584105 | PQLSKGKGNKIINIPSAEAAKGDDGLAHLYVLPPQSTLTIHVGKRKIKLRPEELQKVVGE |
| SRR5584565 | PQLSKGKGNKIINIPSAEAAKGDDGLAHLYVLPPQSTLTIHVGKRKIKLRPEELQKVVGE |
| SRR5584614 | PQLSKGKGNKIINIPSAEAAKGDDGLAHLYVLPPQSTLTIHVGKRKIKLRPEELQKVVGE |
| SRR5631543 | PQLSKGKGNKIINIPSAEAAKGDDGLAHLYVLPPQSTLTIHVGKRKIKLRPEELQKVVGE |
| SRR5631553 | PQLSKGKGNKIINIPSAEAAKGDDGLAHLYVLPPQSTLTIHVGKRKIKLRPEELQKVVGE |
| SRR7123196 | PQLSKGKGNKIINIPSAEAAKGDDGLAHLYVLPPQSTLTIHVGKRKIKLRPEELQKVVGE |
| SRR7163819 | PQLSKGKGNKIINIPSAEAAKGDDGLAHLYVLPPQSTLTIHVGKRKIKLRPEELQKVVGE |
| SRR7163920 | PQLSKGKGNKIINIPSAEAAKGDDGLAHLYVLPPQSTLTIHVGKRKIKLRPEELQKVVGE |
| SRR7209528 | PQLSKGKGNKIINIPSAEAAKGDDGLAHLYVLPPQSTLTIHVGKRKIKLRPEELQKVVGE |
| SRR7249868 | PQLSKGKGNKIINIPSAEAAKGDDGLAHLYVLPPQSTLTIHVGKRKIKLRPEELQKVVGE |
| SRR7278088 | PQLSKGKGNKIINIPSAEAAKGDDGLAHLYVLPPQSTLTIHVGKRKIKLRPEELQKVVGE |
| SRR7285788 | PQLSKGKGNKIINIPSAEAAKGDDGLAHLYVLPPQSTLTIHVGKRKIKLRPEELQKVVGE |
| SRR7286789 | PQLSKGKGNKIINIPSAEAAKGDDGLAHLYVLPPQSTLTIHVGKRKIKLRPEELQKVVGE |
| SRR7286886 | PQLSKGKGNKIINIPSAEAAKGDDGLAHLYVLPPQSTLTIHVGKRKIKLRPEELQKVVGE |
| SRR7310632 | PQLSKGKGNKIINIPSAEAAKGDDGLAHLYVLPPQSTLTIHVGKRKIKLRPEELQKVVGE |
| SRR7350631 | PQLSKGKGNKIINIPSAEAAKGDDGLAHLYVLPPQSTLTIHVGKRKIKLRPEELQKVVGE |
| SRR7458741 | PQLSKGKGNKIINIPSAEAAKGDDGLAHLYVLPPQSTLTIHVGKRKIKLRPEELQKVVGE |
| SRR7480280 | PQLSKGKGNKIINIPSAEAAKGDDGLAHLYVLPPQSTLTIHVGKRKIKLRPEELQKVVGE |
| SRR7523660 | PQLSKGKGNKIINIPSAEAAKGDDGLAHLYVLPPQSTLTIHVGKRKIKLRPEELQKVVGE |
| SRR7523775 | PQLSKGKGNKIINIPSAEAAKGDDGLAHLYVLPPQSTLTIHVGKRKIKLRPEELQKVVGE |
| SRR7251101 | PQLSKGKGNKIINIPSAEAAKGDDGLAHLYVLPPQSTLTIHVGKRKIKLRPEELQKVVGE |
| SRR7284299 | PQLSKGKGNKIINIPSAEAAKGDDGLAHLYVLPPQSTLTIHVGKRKIKLRPEELQKVVGE |
| SRR7285738 | PQLSKGKGNKIINIPSAEAAKGDDGLAHLYVLPPQSTLTIHVGKRKIKLRPEELQKVVGE |
| SRR7310640 | PQLSKGKGNKIINIPSAEAAKGDDGLAHLYVLPPQSTLTIHVGKRKIKLRPEELQKVVGE |
| SRR7349159 | PQLSKGKGNKIINIPSAEAAKGDDGLAHLYVLPPQSTLTIHVGKRKIKLRPEELQKVVGE |
| SRR7474873 | PQLSKGKGNKIINIPSAEAAKGDDGLAHLYVLPPQSTLTIHVGKRKIKLRPEELQKVVGE |
| SRR7495689 | PQLSKGKGNKIINIPSAEAAKGDDGLAHLYVLPPQSTLTIHVGKRKIKLRPEELQKVVGE |
| SRR7495752 | PQLSKGKGNKIINIPSAEAAKGDDGLAHLYVLPPQSTLTIHVGKRKIKLRPEELQKVVGE |
| ------------------------------------------------------------------------------ | |
| S16BD08730 | RGRRGTLMRGLQRIDRIEIDSPHRVSHGDSEE* |
| S18BD00684 | RGRRGTLMRGLQRIDRIEIDSPHRVSHGDSEE* |
| S18BD03994 | RGRRGTLMRGLQRIDRIEIDSPHRVSHGDSEE* |
| S18BD05011 | RGRRGTLMRGLQRIDRIEIDSPHRVSHGDSEE* |
| RKI_16-03723 | RGRRGTLMRGLQRIDRIEIDSPHRVSHGDSEE* |
| RKI_16-04315 | RGRRGTLMRGLQRIDRIEIDSPHRVSHGDSEE* |
| RKI_17-02304 | RGRRGTLMRGLQRIDRIEIDSPHRVSHGDSEE* |
| RKI_17-02411 | RGRRGTLMRGLQRIDRIEIDSPHRVSHGDSEE* |
| RKI_17-02757 | RGRRGTLMRGLQRIDRIEIDSPHRVSHGDSEE* |
| RKI_17-04797 | RGRRGTLMRGLQRIDRIEIDSPHRVSHGDSEE* |
| RKI_17-06869 | RGRRGTLMRGLQRIDRIEIDSPHRVSHGDSEE* |
| ERR2580277 | RGRRGTLMRGLQRIDRIEIDSPHRVSHGDSEE* |
| ERR2580276 | RGRRGTLMRGLQRIDRIEIDSPHRVSHGDSEE* |
| ERR2580273 | RGRRGTLMRGLQRIDRIEIDSPHRVSHGDSEE* |
| ERR2580274 | RGRRGTLMRGLQRIDRIEIDSPHRVSHGDSEE* |
| ERR2173656 | RGRRGTLMRGLQRIDRIEIDSPHRVSHGDSEE* |
| 17041676 | RGRRGTLMRGLQRIDRIEIDSPHRVSHGDSEE* |
| MT16-000061 | RGRRGTLMRGLQRIDRIEIDSPHRVSHGDSEE* |
| MT16-019416 | RGRRGTLMRGLQRIDRIEIDSPHRVSHGDSEE* |
| MT16-027865 | RGRRGTLMRGLQRIDRIEIDSPHRVSHGDSEE* |
| MT16-031693 | RGRRGTLMRGLQRIDRIEIDSPHRVSHGDSEE* |
| MT16-040253 | RGRRGTLMRGLQRIDRIEIDSPHRVSHGDSEE* |
| MT16-045379 | RGRRGTLMRGLQRIDRIEIDSPHRVSHGDSEE* |
| MT16-442728 | RGRRGTLMRGLQRIDRIEIDSPHRVSHGDSEE* |
| MT16-462857 | RGRRGTLMRGLQRIDRIEIDSPHRVSHGDSEE* |
| MT16-480196 | RGRRGTLMRGLQRIDRIEIDSPHRVSHGDSEE* |
| MT16-861555 | RGRRGTLMRGLQRIDRIEIDSPHRVSHGDSEE* |
| MT17-076833 | RGRRGTLMRGLQRIDRIEIDSPHRVSHGDSEE* |
| MT17-110677 | RGRRGTLMRGLQRIDRIEIDSPHRVSHGDSEE* |
| MT17-131730 | RGRRGTLMRGLQRIDRIEIDSPHRVSHGDSEE* |
| MT17-140890 | RGRRGTLMRGLQRIDRIEIDSPHRVSHGDSEE* |
| MT17-141840 | RGRRGTLMRGLQRIDRIEIDSPHRVSHGDSEE* |
| MT17-152488 | RGRRGTLMRGLQRIDRIEIDSPHRVSHGDSEE* |
| MT17-157311 | RGRRGTLMRGLQRIDRIEIDSPHRVSHGDSEE* |
| MT17-161645 | RGRRGTLMRGLQRIDRIEIDSPHRVSHGDSEE* |
| MT17-167951 | RGRRGTLMRGLQRIDRIEIDSPHRVSHGDSEE* |
| MT18-217732 | RGRRGTLMRGLQRIDRIEIDSPHRVSHGDSEE* |
| MT18-252580 | RGRRGTLMRGLQRIDRIEIDSPHRVSHGDSEE* |
| RIVM_H_2009-01 | RGRRGTLMRGLQRIDRIEIDSPHRVSHGDSEE* |
| RIVM_H_2010-01 | RGRRGTLMRGLQRIDRIEIDSPHRVSHGDSEE* |
| RIVM_H_2010-02 | RGRRGTLMRGLQRIDRIEIDSPHRVSHGDSEE* |
| RIVM_H_2011-01 | RGRRGTLMRGLQRIDRIEIDSPHRVSHGDSEE* |
| RIVM_H_2011-02 | RGRRGTLMRGLQRIDRIEIDSPHRVSHGDSEE* |
| RIVM_H_2011-03 | RGRRGTLMRGLQRIDRIEIDSPHRVSHGDSEE* |
| RIVM_H_2013-01 | RGRRGTLMRGLQRIDRIEIDSPHRVSHGDSEE* |
| RIVM_H_2013-02 | RGRRGTLMRGLQRIDRIEIDSPHRVSHGDSEE* |
| RIVM_H_2014-01 | RGRRGTLMRGLQRIDRIEIDSPHRVSHGDSEE* |
| RIVM_H_2014-02 | RGRRGTLMRGLQRIDRIEIDSPHRVSHGDSEE* |
| RIVM_H_2016-01 | RGRRGTLMRGLQRIDRIEIDSPHRVSHGDSEE* |
| RIVM_H_2016-02 | RGRRGTLMRGLQRIDRIEIDSPHRVSHGDSEE* |
| RIVM_H_2016-03 | RGRRGTLMRGLQRIDRIEIDSPHRVSHGDSEE* |
| RIVM_H_2016-04 | RGRRGTLMRGLQRIDRIEIDSPHRVSHGDSEE* |
| RIVM_H_2016-05 | RGRRGTLMRGLQRIDRIEIDSPHRVSHGDSEE* |
| RIVM_H_2016-06 | RGRRGTLMRGLQRIDRIEIDSPHRVSHGDSEE* |
| RIVM_H_2016-07 | RGRRGTLMRGLQRIDRIEIDSPHRVSHGDSEE* |
| RIVM_H_2016-08 | RGRRGTLMRGLQRIDRIEIDSPHRVSHGDSEE* |
| RIVM_H_2016-09 | RGRRGTLMRGLQRIDRIEIDSPHRVSHGDSEE* |
| RIVM_H_2016-10 | RGRRGTLMRGLQRIDRIEIDSPHRVSHGDSEE* |
| RIVM_H_2016-11 | RGRRGTLMRGLQRIDRIEIDSPHRVSHGDSEE* |
| RIVM_H_2016-12 | RGRRGTLMRGLQRIDRIEIDSPHRVSHGDSEE* |
| RIVM_H_2016-13 | RGRRGTLMRGLQRIDRIEIDSPHRVSHGDSEE* |
| RIVM_H_2016-14 | RGRRGTLMRGLQRIDRIEIDSPHRVSHGDSEE* |
| RIVM_H_2016-15 | RGRRGTLMRGLQRIDRIEIDSPHRVSHGDSEE* |
| RIVM_H_2017-01 | RGRRGTLMRGLQRIDRIEIDSPHRVSHGDSEE* |
| RIVM_H_2017-02 | RGRRGTLMRGLQRIDRIEIDSPHRVSHGDSEE* |
| RIVM_H_2017-03 | RGRRGTLMRGLQRIDRIEIDSPHRVSHGDSEE* |
| RIVM_H_2017-04 | RGRRGTLMRGLQRIDRIEIDSPHRVSHGDSEE* |
| RIVM_H_2017-05 | RGRRGTLMRGLQRIDRIEIDSPHRVSHGDSEE* |
| RIVM_H_2017-06 | RGRRGTLMRGLQRIDRIEIDSPHRVSHGDSEE* |
| RIVM_H_2017-07 | RGRRGTLMRGLQRIDRIEIDSPHRVSHGDSEE* |
| RIVM_H_2017-08 | RGRRGTLMRGLQRIDRIEIDSPHRVSHGDSEE* |
| RIVM_H_2017-09 | RGRRGTLMRGLQRIDRIEIDSPHRVSHGDSEE* |
| RIVM_H_2017-10 | RGRRGTLMRGLQRIDRIEIDSPHRVSHGDSEE* |
| RIVM_H_2017-11 | RGRRGTLMRGLQRIDRIEIDSPHRVSHGDSEE* |
| RIVM_H_2017-12 | RGRRGTLMRGLQRIDRIEIDSPHRVSHGDSEE* |
| RIVM_H_2017-13 | RGRRGTLMRGLQRIDRIEIDSPHRVSHGDSEE* |
| RIVM_H_2017-14 | RGRRGTLMRGLQRIDRIEIDSPHRVSHGDSEE* |
| RIVM_H_2017-15 | RGRRGTLMRGLQRIDRIEIDSPHRVSHGDSEE* |
| RIVM_H_2017-16 | RGRRGTLMRGLQRIDRIEIDSPHRVSHGDSEE* |
| RIVM_H_2017-17 | RGRRGTLMRGLQRIDRIEIDSPHRVSHGDSEE* |
| RIVM_H_2017-18 | RGRRGTLMRGLQRIDRIEIDSPHRVSHGDSEE* |
| RIVM_H_2017-19 | RGRRGTLMRGLQRIDRIEIDSPHRVSHGDSEE* |
| 15EP001483 | RGRRGTLMRGLQRIDRIEIDSPHRVSHGDSEE* |
| 17EP002363 | RGRRGTLMRGLQRIDRIEIDSPHRVSHGDSEE* |
| S_0812_17 | RGRRGTLMRGLQRIDRIEIDSPHRVSHGDSEE* |
| SRR1957844 | RGRRGTLMRGLQRIDRIEIDSPHRVSHGDSEE* |
| SRR1958654 | RGRRGTLMRGLQRIDRIEIDSPHRVSHGDSEE* |
| SRR1965077 | RGRRGTLMRGLQRIDRIEIDSPHRVSHGDSEE* |
| SRR1966369 | RGRRGTLMRGLQRIDRIEIDSPHRVSHGDSEE* |
| SRR1967117 | RGRRGTLMRGLQRIDRIEIDSPHRVSHGDSEE* |
| SRR1967922 | RGRRGTLMRGLQRIDRIEIDSPHRVSHGDSEE* |
| SRR8704720 | RGRRGTLMRGLQRIDRIEIDSPHRVSHGDSEE* |
| SRR7216071 | RGRRGTLMRGLQRIDRIEIDSPHRVSHGDSEE* |
| SRR7349175 | RGRRGTLMRGLQRIDRIEIDSPHRVSHGDSEE* |
| SRR7523148 | RGRRGTLMRGLQRIDRIEIDSPHRVSHGDSEE* |
| SRR7523854 | RGRRGTLMRGLQRIDRIEIDSPHRVSHGDSEE* |
| 313865 | RGRRGTLMRGLQRIDRIEIDSPHRVSHGDSEE* |
| SRR7277793 | RGRRGTLMRGLQRIDRIEIDSPHRVSHGDSEE* |
| SRR7343877 | RGRRGTLMRGLQRIDRIEIDSPHRVSHGDSEE* |
| SRR7351477 | RGRRGTLMRGLQRIDRIEIDSPHRVSHGDSEE* |
| SRR5583183 | RGRRGTLMRGLQRIDRIEIDSPHRVSHGDSEE* |
| SRR5585240 | RGRRGTLMRGLQRIDRIEIDSPHRVSHGDSEE* |
| SRR7284317 | RGRRGTLMRGLQRIDRIEIDSPHRVSHGDSEE* |
| SRR7299161 | RGRRGTLMRGLQRIDRIEIDSPHRVSHGDSEE* |
| SRR7401730 | RGRRGTLMRGLQRIDRIEIDSPHRVSHGDSEE* |
| SRR7469092 | RGRRGTLMRGLQRIDRIEIDSPHRVSHGDSEE* |
| SRR7879556 | RGRRGTLMRGLQRIDRIEIDSPHRVSHGDSEE* |
| SRR8526100 | RGRRGTLMRGLQRIDRIEIDSPHRVSHGDSEE* |
| SRR8553991 | RGRRGTLMRGLQRIDRIEIDSPHRVSHGDSEE* |
| SRR7842487 | RGRRGTLMRGLQRIDRIEIDSPHRVSHGDSEE* |
| SRR8054524 | RGRRGTLMRGLQRIDRIEIDSPHRVSHGDSEE* |
| SRR8054525 | RGRRGTLMRGLQRIDRIEIDSPHRVSHGDSEE* |
| SRR8524733 | RGRRGTLMRGLQRIDRIEIDSPHRVSHGDSEE* |
| SRR4093291 | RGRRGTLMRGLQRIDRIEIDSPHRVSHGDSEE* |
| SRR4245549 | RGRRGTLMRGLQRIDRIEIDSPHRVSHGDSEE* |
| SRR3057154 | RGRRGTLMRGLQRIDRIEIDSPHRVSHGDSEE* |
| SRR1726150 | RGRRGTLMRGLQRIDRIEIDSPHRVSHGDSEE* |
| SRR1996141 | RGRRGTLMRGLQRIDRIEIDSPHRVSHGDSEE* |
| SRR1107842 | RGRRGTLMRGLQRIDRIEIDSPHRVSHGDSEE* |
| SRR1157587 | RGRRGTLMRGLQRIDRIEIDSPHRVSHGDSEE* |
| SRR3027706 | RGRRGTLMRGLQRIDRIEIDSPHRVSHGDSEE* |
| SRR3027707 | RGRRGTLMRGLQRIDRIEIDSPHRVSHGDSEE* |
| SRR3027708 | RGRRGTLMRGLQRIDRIEIDSPHRVSHGDSEE* |
| SRR3027710 | RGRRGTLMRGLQRIDRIEIDSPHRVSHGDSEE* |
| SRR3027711 | RGRRGTLMRGLQRIDRIEIDSPHRVSHGDSEE* |
| SRR3027716 | RGRRGTLMRGLQRIDRIEIDSPHRVSHGDSEE* |
| SRR3027717 | RGRRGTLMRGLQRIDRIEIDSPHRVSHGDSEE* |
| SRR3027719 | RGRRGTLMRGLQRIDRIEIDSPHRVSHGDSEE* |
| SRR3027721 | RGRRGTLMRGLQRIDRIEIDSPHRVSHGDSEE* |
| SRR3027723 | RGRRGTLMRGLQRIDRIEIDSPHRVSHGDSEE* |
| SRR3115978 | RGRRGTLMRGLQRIDRIEIDSPHRVSHGDSEE* |
| SRR2534093 | RGRRGTLMRGLQRIDRIEIDSPHRVSHGDSEE* |
| SRR2534094 | RGRRGTLMRGLQRIDRIEIDSPHRVSHGDSEE* |
| SRR2534095 | RGRRGTLMRGLQRIDRIEIDSPHRVSHGDSEE* |
| SRR2534108 | RGRRGTLMRGLQRIDRIEIDSPHRVSHGDSEE* |
| SRR1106464 | RGRRGTLMRGLQRIDRIEIDSPHRVSHGDSEE* |
| SRR1106463 | RGRRGTLMRGLQRIDRIEIDSPHRVSHGDSEE* |
| SRR6949610 | RGRRGTLMRGLQRIDRIEIDSPHRVSHGDSEE* |
| SRR6950452 | RGRRGTLMRGLQRIDRIEIDSPHRVSHGDSEE* |
| ERR2019831 | RGRRGTLMRGLQRIDRIEIDSPHRVSHGDSEE* |
| SRR2085693 | RGRRGTLMRGLQRIDRIEIDSPHRVSHGDSEE* |
| SRR2086898 | RGRRGTLMRGLQRIDRIEIDSPHRVSHGDSEE* |
| SRR2175312 | RGRRGTLMRGLQRIDRIEIDSPHRVSHGDSEE* |
| SRR2175360 | RGRRGTLMRGLQRIDRIEIDSPHRVSHGDSEE* |
| SRR5231997 | RGRRGTLMRGLQRIDRIEIDSPHRVSHGDSEE* |
| SRR5232003 | RGRRGTLMRGLQRIDRIEIDSPHRVSHGDSEE* |
| SRR5232015 | RGRRGTLMRGLQRIDRIEIDSPHRVSHGDSEE* |
| SRR949434 | RGRRGTLMRGLQRIDRIEIDSPHRVSHGDSEE* |
| SRR3216575 | RGRRGTLMRGLQRIDRIEIDSPHRVSHGDSEE* |
| SRR5205342 | RGRRGTLMRGLQRIDRIEIDSPHRVSHGDSEE* |
| SRR1501669 | RGRRGTLMRGLQRIDRIEIDSPHRVSHGDSEE* |
| SRR5209740 | RGRRGTLMRGLQRIDRIEIDSPHRVSHGDSEE* |
| SRR3240355 | RGRRGTLMRGLQRIDRIEIDSPHRVSHGDSEE* |
| SRR3392777 | RGRRGTLMRGLQRIDRIEIDSPHRVSHGDSEE* |
| SRR3593671 | RGRRGTLMRGLQRIDRIEIDSPHRVSHGDSEE* |
| SRR5413290 | RGRRGTLMRGLQRIDRIEIDSPHRVSHGDSEE* |
| SRR5590269 | RGRRGTLMRGLQRIDRIEIDSPHRVSHGDSEE* |
| SRR5812103 | RGRRGTLMRGLQRIDRIEIDSPHRVSHGDSEE* |
| SRR2830941 | RGRRGTLMRGLQRIDRIEIDSPHRVSHGDSEE* |
| SRR2830966 | RGRRGTLMRGLQRIDRIEIDSPHRVSHGDSEE* |
| SRR3137270 | RGRRGTLMRGLQRIDRIEIDSPHRVSHGDSEE* |
| SRR3137271 | RGRRGTLMRGLQRIDRIEIDSPHRVSHGDSEE* |
| ERR526807 | RGRRGTLMRGLQRIDRIEIDSPHRVSHGDSEE* |
| ERR2197922 | RGRRGTLMRGLQRIDRIEIDSPHRVSHGDSEE* |
| ERR2197923 | RGRRGTLMRGLQRIDRIEIDSPHRVSHGDSEE* |
| ERR2197924 | RGRRGTLMRGLQRIDRIEIDSPHRVSHGDSEE* |
| ERR2197925 | RGRRGTLMRGLQRIDRIEIDSPHRVSHGDSEE* |
| ERR2197927 | RGRRGTLMRGLQRIDRIEIDSPHRVSHGDSEE* |
| ERR2197929 | RGRRGTLMRGLQRIDRIEIDSPHRVSHGDSEE* |
| SRR1648149 | RGRRGTLMRGLQRIDRIEIDSPHRVSHGDSEE* |
| SRR1048299 | RGRRGTLMRGLQRIDRIEIDSPHRVSHGDSEE* |
| SRR1300677 | RGRRGTLMRGLQRIDRIEIDSPHRVSHGDSEE* |
| SRR1288356 | RGRRGTLMRGLQRIDRIEIDSPHRVSHGDSEE* |
| SRR7426190 | RGRRGTLMRGLQRIDRIEIDSPHRVSHGDSEE* |
| SRR7426192 | RGRRGTLMRGLQRIDRIEIDSPHRVSHGDSEE* |
| SRR7426193 | RGRRGTLMRGLQRIDRIEIDSPHRVSHGDSEE* |
| SRR7441832 | RGRRGTLMRGLQRIDRIEIDSPHRVSHGDSEE* |
| SRR7426179 | RGRRGTLMRGLQRIDRIEIDSPHRVSHGDSEE* |
| SRR7439238 | RGRRGTLMRGLQRIDRIEIDSPHRVSHGDSEE* |
| SRR7439244 | RGRRGTLMRGLQRIDRIEIDSPHRVSHGDSEE* |
| SRR7439259 | RGRRGTLMRGLQRIDRIEIDSPHRVSHGDSEE* |
| SRR7439260 | RGRRGTLMRGLQRIDRIEIDSPHRVSHGDSEE* |
| SRR7441786 | RGRRGTLMRGLQRIDRIEIDSPHRVSHGDSEE* |
| SRR7441797 | RGRRGTLMRGLQRIDRIEIDSPHRVSHGDSEE* |
| ERR1759093 | RGRRGTLMRGLQRIDRIEIDSPHRVSHGDSEE* |
| ERR2580275 | RGRRGTLMRGLQRIDRIEIDSPHRVSHGDSEE* |
| ERR1759204 | RGRRGTLMRGLQRIDRIEIDSPHRVSHGDSEE* |
| SRR1300699 | RGRRGTLMRGLQRIDRIEIDSPHRVSHGDSEE* |
| S_0825_17 | RGRRGTLMRGLQRIDRIEIDSPHRVSHGDSEE* |
| SRR1958215 | RGRRGTLMRGLQRIDRIEIDSPHRVSHGDSEE* |
| SRR1958540 | RGRRGTLMRGLQRIDRIEIDSPHRVSHGDSEE* |
| SRR1958636 | RGRRGTLMRGLQRIDRIEIDSPHRVSHGDSEE* |
| SRR1959422 | RGRRGTLMRGLQRIDRIEIDSPHRVSHGDSEE* |
| SRR1959427 | RGRRGTLMRGLQRIDRIEIDSPHRVSHGDSEE* |
| SRR1960226 | RGRRGTLMRGLQRIDRIEIDSPHRVSHGDSEE* |
| SRR1963498 | RGRRGTLMRGLQRIDRIEIDSPHRVSHGDSEE* |
| SRR1965947 | RGRRGTLMRGLQRIDRIEIDSPHRVSHGDSEE* |
| SRR1966125 | RGRRGTLMRGLQRIDRIEIDSPHRVSHGDSEE* |
| SRR1966330 | RGRRGTLMRGLQRIDRIEIDSPHRVSHGDSEE* |
| SRR1966565 | RGRRGTLMRGLQRIDRIEIDSPHRVSHGDSEE* |
| SRR1966864 | RGRRGTLMRGLQRIDRIEIDSPHRVSHGDSEE* |
| SRR1966989 | RGRRGTLMRGLQRIDRIEIDSPHRVSHGDSEE* |
| SRR1967688 | RGRRGTLMRGLQRIDRIEIDSPHRVSHGDSEE* |
| SRR1967733 | RGRRGTLMRGLQRIDRIEIDSPHRVSHGDSEE* |
| SRR1967746 | RGRRGTLMRGLQRIDRIEIDSPHRVSHGDSEE* |
| SRR1968341 | RGRRGTLMRGLQRIDRIEIDSPHRVSHGDSEE* |
| SRR1968456 | RGRRGTLMRGLQRIDRIEIDSPHRVSHGDSEE* |
| SRR1968465 | RGRRGTLMRGLQRIDRIEIDSPHRVSHGDSEE* |
| SRR1968761 | RGRRGTLMRGLQRIDRIEIDSPHRVSHGDSEE* |
| SRR1969047 | RGRRGTLMRGLQRIDRIEIDSPHRVSHGDSEE* |
| SRR1969255 | RGRRGTLMRGLQRIDRIEIDSPHRVSHGDSEE* |
| SRR1969412 | RGRRGTLMRGLQRIDRIEIDSPHRVSHGDSEE* |
| SRR1969524 | RGRRGTLMRGLQRIDRIEIDSPHRVSHGDSEE* |
| SRR1969584 | RGRRGTLMRGLQRIDRIEIDSPHRVSHGDSEE* |
| SRR1969648 | RGRRGTLMRGLQRIDRIEIDSPHRVSHGDSEE* |
| SRR1969804 | RGRRGTLMRGLQRIDRIEIDSPHRVSHGDSEE* |
| SRR1970221 | RGRRGTLMRGLQRIDRIEIDSPHRVSHGDSEE* |
| SRR1970268 | RGRRGTLMRGLQRIDRIEIDSPHRVSHGDSEE* |
| SRR1965862 | RGRRGTLMRGLQRIDRIEIDSPHRVSHGDSEE* |
| SRR1967363 | RGRRGTLMRGLQRIDRIEIDSPHRVSHGDSEE* |
| SRR1968276 | RGRRGTLMRGLQRIDRIEIDSPHRVSHGDSEE* |
| SRR1968967 | RGRRGTLMRGLQRIDRIEIDSPHRVSHGDSEE* |
| SRR3321531 | RGRRGTLMRGLQRIDRIEIDSPHRVSHGDSEE* |
| SRR3321883 | RGRRGTLMRGLQRIDRIEIDSPHRVSHGDSEE* |
| SRR3322413 | RGRRGTLMRGLQRIDRIEIDSPHRVSHGDSEE* |
| SRR3323012 | RGRRGTLMRGLQRIDRIEIDSPHRVSHGDSEE* |
| SRR5194289 | RGRRGTLMRGLQRIDRIEIDSPHRVSHGDSEE* |
| SRR7163798 | RGRRGTLMRGLQRIDRIEIDSPHRVSHGDSEE* |
| SRR7172610 | RGRRGTLMRGLQRIDRIEIDSPHRVSHGDSEE* |
| SRR7204568 | RGRRGTLMRGLQRIDRIEIDSPHRVSHGDSEE* |
| SRR7223230 | RGRRGTLMRGLQRIDRIEIDSPHRVSHGDSEE* |
| SRR7230675 | RGRRGTLMRGLQRIDRIEIDSPHRVSHGDSEE* |
| SRR7278056 | RGRRGTLMRGLQRIDRIEIDSPHRVSHGDSEE* |
| SRR7278086 | RGRRGTLMRGLQRIDRIEIDSPHRVSHGDSEE* |
| SRR7285841 | RGRRGTLMRGLQRIDRIEIDSPHRVSHGDSEE* |
| SRR7292625 | RGRRGTLMRGLQRIDRIEIDSPHRVSHGDSEE* |
| SRR7292665 | RGRRGTLMRGLQRIDRIEIDSPHRVSHGDSEE* |
| SRR7297965 | RGRRGTLMRGLQRIDRIEIDSPHRVSHGDSEE* |
| SRR7350726 | RGRRGTLMRGLQRIDRIEIDSPHRVSHGDSEE* |
| SRR7410328 | RGRRGTLMRGLQRIDRIEIDSPHRVSHGDSEE* |
| SRR7474665 | RGRRGTLMRGLQRIDRIEIDSPHRVSHGDSEE* |
| SRR7523184 | RGRRGTLMRGLQRIDRIEIDSPHRVSHGDSEE* |
| SRR7187264 | RGRRGTLMRGLQRIDRIEIDSPHRVSHGDSEE* |
| SRR7204445 | RGRRGTLMRGLQRIDRIEIDSPHRVSHGDSEE* |
| SRR7285641 | RGRRGTLMRGLQRIDRIEIDSPHRVSHGDSEE* |
| SRR7286695 | RGRRGTLMRGLQRIDRIEIDSPHRVSHGDSEE* |
| SRR7286705 | RGRRGTLMRGLQRIDRIEIDSPHRVSHGDSEE* |
| SRR7292931 | RGRRGTLMRGLQRIDRIEIDSPHRVSHGDSEE* |
| SRR7310349 | RGRRGTLMRGLQRIDRIEIDSPHRVSHGDSEE* |
| SRR7351616 | RGRRGTLMRGLQRIDRIEIDSPHRVSHGDSEE* |
| SRR7414818 | RGRRGTLMRGLQRIDRIEIDSPHRVSHGDSEE* |
| SRR7426480 | RGRRGTLMRGLQRIDRIEIDSPHRVSHGDSEE* |
| SRR5584105 | RGRRGTLMRGLQRIDRIEIDSPHRVSHGDSEE* |
| SRR5584565 | RGRRGTLMRGLQRIDRIEIDSPHRVSHGDSEE* |
| SRR5584614 | RGRRGTLMRGLQRIDRIEIDSPHRVSHGDSEE* |
| SRR5631543 | RGRRGTLMRGLQRIDRIEIDSPHRVSHGDSEE* |
| SRR5631553 | RGRRGTLMRGLQRIDRIEIDSPHRVSHGDSEE* |
| SRR7123196 | RGRRGTLMRGLQRIDRIEIDSPHRVSHGDSEE* |
| SRR7163819 | RGRRGTLMRGLQRIDRIEIDSPHRVSHGDSEE* |
| SRR7163920 | RGRRGTLMRGLQRIDRIEIDSPHRVSHGDSEE* |
| SRR7209528 | RGRRGTLMRGLQRIDRIEIDSPHRVSHGDSEE* |
| SRR7249868 | RGRRGTLMRGLQRIDRIEIDSPHRVSHGDSEE* |
| SRR7278088 | RGRRGTLMRGLQRIDRIEIDSPHRVSHGDSEE* |
| SRR7285788 | RGRRGTLMRGLQRIDRIEIDSPHRVSHGDSEE* |
| SRR7286789 | RGRRGTLMRGLQRIDRIEIDSPHRVSHGDSEE* |
| SRR7286886 | RGRRGTLMRGLQRIDRIEIDSPHRVSHGDSEE* |
| SRR7310632 | RGRRGTLMRGLQRIDRIEIDSPHRVSHGDSEE* |
| SRR7350631 | RGRRGTLMRGLQRIDRIEIDSPHRVSHGDSEE* |
| SRR7458741 | RGRRGTLMRGLQRIDRIEIDSPHRVSHGDSEE* |
| SRR7480280 | RGRRGTLMRGLQRIDRIEIDSPHRVSHGDSEE* |
| SRR7523660 | RGRRGTLMRGLQRIDRIEIDSPHRVSHGDSEE* |
| SRR7523775 | RGRRGTLMRGLQRIDRIEIDSPHRVSHGDSEE* |
| SRR7251101 | RGRRGTLMRGLQRIDRIEIDSPHRVSHGDSEE* |
| SRR7284299 | RGRRGTLMRGLQRIDRIEIDSPHRVSHGDSEE* |
| SRR7285738 | RGRRGTLMRGLQRIDRIEIDSPHRVSHGDSEE* |
| SRR7310640 | RGRRGTLMRGLQRIDRIEIDSPHRVSHGDSEE* |
| SRR7349159 | RGRRGTLMRGLQRIDRIEIDSPHRVSHGDSEE* |
| SRR7474873 | RGRRGTLMRGLQRIDRIEIDSPHRVSHGDSEE* |
| SRR7495689 | RGRRGTLMRGLQRIDRIEIDSPHRVSHGDSEE* |
| SRR7495752 | RGRRGTLMRGLQRIDRIEIDSPHRVSHGDSEE* |
