# Supplementary material for: Genomic epidemiology of emerging ESBL-producing Salmonella Kentucky blaCTX-M-14b in Europe
Source: Emerg Microbes Infect. 2020 Sep 30;9(1):2124–35. doi: 10.1080/22221751.2020.1821582 (PMC7580578; doi:10.1080/22221751.2020.1821582)
Supplement: Suppelemental_files.zip [file TEMI_A_1821582_SM9137.zip › AlignS2.docx]

| AlignS2 |  |
| --- | --- |
| CLUSTAL W (1.7) multiple sequence alignment | |
|  |  |
|  |  |
| S16BD08730 | MSDLAREITPVNIEEELKSSYLDYAMSVIVGRALPDVRDGLKPVHRRVLYAMNVLGNDWN |
| S18BD00684 | MSDLAREITPVNIEEELKSSYLDYAMSVIVGRALPDVRDGLKPVHRRVLYAMNVLGNDWN |
| S18BD03994 | MSDLAREITPVNIEEELKSSYLDYAMSVIVGRALPDVRDGLKPVHRRVLYAMNVLGNDWN |
| S18BD05011 | MSDLAREITPVNIEEELKSSYLDYAMSVIVGRALPDVRDGLKPVHRRVLYAMNVLGNDWN |
| RKI_16-03723 | MSDLAREITPVNIEEELKSSYLDYAMSVIVGRALPDVRDGLKPVHRRVLYAMNVLGNDWN |
| RKI_16-04315 | MSDLAREITPVNIEEELKSSYLDYAMSVIVGRALPDVRDGLKPVHRRVLYAMNVLGNDWN |
| RKI_17-02304 | MSDLAREITPVNIEEELKSSYLDYAMSVIVGRALPDVRDGLKPVHRRVLYAMNVLGNDWN |
| RKI_17-02411 | MSDLAREITPVNIEEELKSSYLDYAMSVIVGRALPDVRDGLKPVHRRVLYAMNVLGNDWN |
| RKI_17-02757 | MSDLAREITPVNIEEELKSSYLDYAMSVIVGRALPDVRDGLKPVHRRVLYAMNVLGNDWN |
| RKI_17-04797 | MSDLAREITPVNIEEELKSSYLDYAMSVIVGRALPDVRDGLKPVHRRVLYAMNVLGNDWN |
| RKI_17-06869 | MSDLAREITPVNIEEELKSSYLDYAMSVIVGRALPDVRDGLKPVHRRVLYAMNVLGNDWN |
| ERR2580277 | MSDLAREITPVNIEEELKSSYLDYAMSVIVGRALPDVRDGLKPVHRRVLYAMNVLGNDWN |
| ERR2580276 | MSDLAREITPVNIEEELKSSYLDYAMSVIVGRALPDVRDGLKPVHRRVLYAMNVLGNDWN |
| ERR2580273 | MSDLAREITPVNIEEELKSSYLDYAMSVIVGRALPDVRDGLKPVHRRVLYAMNVLGNDWN |
| ERR2580274 | MSDLAREITPVNIEEELKSSYLDYAMSVIVGRALPDVRDGLKPVHRRVLYAMNVLGNDWN |
| ERR2173656 | MSDLAREITPVNIEEELKSSYLDYAMSVIVGRALPDVRDGLKPVHRRVLYAMNVLGNDWN |
| 17041676 | MSDLAREITPVNIEEELKSSYLDYAMSVIVGRALPDVRDGLKPVHRRVLYAMNVLGNDWN |
| MT16-000061 | MSDLAREITPVNIEEELKSSYLDYAMSVIVGRALPDVRDGLKPVHRRVLYAMNVLGNDWN |
| MT16-019416 | MSDLAREITPVNIEEELKSSYLDYAMSVIVGRALPDVRDGLKPVHRRVLYAMNVLGNDWN |
| MT16-027865 | MSDLAREITPVNIEEELKSSYLDYAMSVIVGRALPDVRDGLKPVHRRVLYAMNVLGNDWN |
| MT16-031693 | MSDLAREITPVNIEEELKSSYLDYAMSVIVGRALPDVRDGLKPVHRRVLYAMNVLGNDWN |
| MT16-040253 | MSDLAREITPVNIEEELKSSYLDYAMSVIVGRALPDVRDGLKPVHRRVLYAMNVLGNDWN |
| MT16-045379 | MSDLAREITPVNIEEELKSSYLDYAMSVIVGRALPDVRDGLKPVHRRVLYAMNVLGNDWN |
| MT16-442728 | MSDLAREITPVNIEEELKSSYLDYAMSVIVGRALPDVRDGLKPVHRRVLYAMNVLGNDWN |
| MT16-462857 | MSDLAREITPVNIEEELKSSYLDYAMSVIVGRALPDVRDGLKPVHRRVLYAMNVLGNDWN |
| MT16-480196 | MSDLAREITPVNIEEELKSSYLDYAMSVIVGRALPDVRDGLKPVHRRVLYAMNVLGNDWN |
| MT16-861555 | MSDLAREITPVNIEEELKSSYLDYAMSVIVGRALPDVRDGLKPVHRRVLYAMNVLGNDWN |
| MT17-076833 | MSDLAREITPVNIEEELKSSYLDYAMSVIVGRALPDVRDGLKPVHRRVLYAMNVLGNDWN |
| MT17-110677 | MSDLAREITPVNIEEELKSSYLDYAMSVIVGRALPDVRDGLKPVHRRVLYAMNVLGNDWN |
| MT17-131730 | MSDLAREITPVNIEEELKSSYLDYAMSVIVGRALPDVRDGLKPVHRRVLYAMNVLGNDWN |
| MT17-140890 | MSDLAREITPVNIEEELKSSYLDYAMSVIVGRALPDVRDGLKPVHRRVLYAMNVLGNDWN |
| MT17-141840 | MSDLAREITPVNIEEELKSSYLDYAMSVIVGRALPDVRDGLKPVHRRVLYAMNVLGNDWN |
| MT17-152488 | MSDLAREITPVNIEEELKSSYLDYAMSVIVGRALPDVRDGLKPVHRRVLYAMNVLGNDWN |
| MT17-157311 | MSDLAREITPVNIEEELKSSYLDYAMSVIVGRALPDVRDGLKPVHRRVLYAMNVLGNDWN |
| MT17-161645 | MSDLAREITPVNIEEELKSSYLDYAMSVIVGRALPDVRDGLKPVHRRVLYAMNVLGNDWN |
| MT17-167951 | MSDLAREITPVNIEEELKSSYLDYAMSVIVGRALPDVRDGLKPVHRRVLYAMNVLGNDWN |
| MT18-217732 | MSDLAREITPVNIEEELKSSYLDYAMSVIVGRALPDVRDGLKPVHRRVLYAMNVLGNDWN |
| MT18-252580 | MSDLAREITPVNIEEELKSSYLDYAMSVIVGRALPDVRDGLKPVHRRVLYAMNVLGNDWN |
| RIVM_H_2009-01 | MSDLAREITPVNIEEELKSSYLDYAMSVIVGRALPDVRDGLKPVHRRVLYAMNVLGNDWN |
| RIVM_H_2010-01 | MSDLAREITPVNIEEELKSSYLDYAMSVIVGRALPDVRDGLKPVHRRVLYAMNVLGNDWN |
| RIVM_H_2010-02 | MSDLAREITPVNIEEELKSSYLDYAMSVIVGRALPDVRDGLKPVHRRVLYAMNVLGNDWN |
| RIVM_H_2011-01 | MSDLAREITPVNIEEELKSSYLDYAMSVIVGRALPDVRDGLKPVHRRVLYAMNVLGNDWN |
| RIVM_H_2011-02 | MSDLAREITPVNIEEELKSSYLDYAMSVIVGRALPDVRDGLKPVHRRVLYAMNVLGNDWN |
| RIVM_H_2011-03 | MSDLAREITPVNIEEELKSSYLDYAMSVIVGRALPDVRDGLKPVHRRVLYAMNVLGNDWN |
| RIVM_H_2013-01 | MSDLAREITPVNIEEELKSSYLDYAMSVIVGRALPDVRDGLKPVHRRVLYAMNVLGNDWN |
| RIVM_H_2013-02 | MSDLAREITPVNIEEELKSSYLDYAMSVIVGRALPDVRDGLKPVHRRVLYAMNVLGNDWN |
| RIVM_H_2014-01 | MSDLAREITPVNIEEELKSSYLDYAMSVIVGRALPDVRDGLKPVHRRVLYAMNVLGNDWN |
| RIVM_H_2014-02 | MSDLAREITPVNIEEELKSSYLDYAMSVIVGRALPDVRDGLKPVHRRVLYAMNVLGNDWN |
| RIVM_H_2016-01 | MSDLAREITPVNIEEELKSSYLDYAMSVIVGRALPDVRDGLKPVHRRVLYAMNVLGNDWN |
| RIVM_H_2016-02 | MSDLAREITPVNIEEELKSSYLDYAMSVIVGRALPDVRDGLKPVHRRVLYAMNVLGNDWN |
| RIVM_H_2016-03 | MSDLAREITPVNIEEELKSSYLDYAMSVIVGRALPDVRDGLKPVHRRVLYAMNVLGNDWN |
| RIVM_H_2016-04 | MSDLAREITPVNIEEELKSSYLDYAMSVIVGRALPDVRDGLKPVHRRVLYAMNVLGNDWN |
| RIVM_H_2016-05 | MSDLAREITPVNIEEELKSSYLDYAMSVIVGRALPDVRDGLKPVHRRVLYAMNVLGNDWN |
| RIVM_H_2016-06 | MSDLAREITPVNIEEELKSSYLDYAMSVIVGRALPDVRDGLKPVHRRVLYAMNVLGNDWN |
| RIVM_H_2016-07 | MSDLAREITPVNIEEELKSSYLDYAMSVIVGRALPDVRDGLKPVHRRVLYAMNVLGNDWN |
| RIVM_H_2016-08 | MSDLAREITPVNIEEELKSSYLDYAMSVIVGRALPDVRDGLKPVHRRVLYAMNVLGNDWN |
| RIVM_H_2016-09 | MSDLAREITPVNIEEELKSSYLDYAMSVIVGRALPDVRDGLKPVHRRVLYAMNVLGNDWN |
| RIVM_H_2016-10 | MSDLAREITPVNIEEELKSSYLDYAMSVIVGRALPDVRDGLKPVHRRVLYAMNVLGNDWN |
| RIVM_H_2016-11 | MSDLAREITPVNIEEELKSSYLDYAMSVIVGRALPDVRDGLKPVHRRVLYAMNVLGNDWN |
| RIVM_H_2016-12 | MSDLAREITPVNIEEELKSSYLDYAMSVIVGRALPDVRDGLKPVHRRVLYAMNVLGNDWN |
| RIVM_H_2016-13 | MSDLAREITPVNIEEELKSSYLDYAMSVIVGRALPDVRDGLKPVHRRVLYAMNVLGNDWN |
| RIVM_H_2016-14 | MSDLAREITPVNIEEELKSSYLDYAMSVIVGRALPDVRDGLKPVHRRVLYAMNVLGNDWN |
| RIVM_H_2016-15 | MSDLAREITPVNIEEELKSSYLDYAMSVIVGRALPDVRDGLKPVHRRVLYAMNVLGNDWN |
| RIVM_H_2017-01 | MSDLAREITPVNIEEELKSSYLDYAMSVIVGRALPDVRDGLKPVHRRVLYAMNVLGNDWN |
| RIVM_H_2017-02 | MSDLAREITPVNIEEELKSSYLDYAMSVIVGRALPDVRDGLKPVHRRVLYAMNVLGNDWN |
| RIVM_H_2017-03 | MSDLAREITPVNIEEELKSSYLDYAMSVIVGRALPDVRDGLKPVHRRVLYAMNVLGNDWN |
| RIVM_H_2017-04 | MSDLAREITPVNIEEELKSSYLDYAMSVIVGRALPDVRDGLKPVHRRVLYAMNVLGNDWN |
| RIVM_H_2017-05 | MSDLAREITPVNIEEELKSSYLDYAMSVIVGRALPDVRDGLKPVHRRVLYAMNVLGNDWN |
| RIVM_H_2017-06 | MSDLAREITPVNIEEELKSSYLDYAMSVIVGRALPDVRDGLKPVHRRVLYAMNVLGNDWN |
| RIVM_H_2017-07 | MSDLAREITPVNIEEELKSSYLDYAMSVIVGRALPDVRDGLKPVHRRVLYAMNVLGNDWN |
| RIVM_H_2017-08 | MSDLAREITPVNIEEELKSSYLDYAMSVIVGRALPDVRDGLKPVHRRVLYAMNVLGNDWN |
| RIVM_H_2017-09 | MSDLAREITPVNIEEELKSSYLDYAMSVIVGRALPDVRDGLKPVHRRVLYAMNVLGNDWN |
| RIVM_H_2017-10 | MSDLAREITPVNIEEELKSSYLDYAMSVIVGRALPDVRDGLKPVHRRVLYAMNVLGNDWN |
| RIVM_H_2017-11 | MSDLAREITPVNIEEELKSSYLDYAMSVIVGRALPDVRDGLKPVHRRVLYAMNVLGNDWN |
| RIVM_H_2017-12 | MSDLAREITPVNIEEELKSSYLDYAMSVIVGRALPDVRDGLKPVHRRVLYAMNVLGNDWN |
| RIVM_H_2017-13 | MSDLAREITPVNIEEELKSSYLDYAMSVIVGRALPDVRDGLKPVHRRVLYAMNVLGNDWN |
| RIVM_H_2017-14 | MSDLAREITPVNIEEELKSSYLDYAMSVIVGRALPDVRDGLKPVHRRVLYAMNVLGNDWN |
| RIVM_H_2017-15 | MSDLAREITPVNIEEELKSSYLDYAMSVIVGRALPDVRDGLKPVHRRVLYAMNVLGNDWN |
| RIVM_H_2017-16 | MSDLAREITPVNIEEELKSSYLDYAMSVIVGRALPDVRDGLKPVHRRVLYAMNVLGNDWN |
| RIVM_H_2017-17 | MSDLAREITPVNIEEELKSSYLDYAMSVIVGRALPDVRDGLKPVHRRVLYAMNVLGNDWN |
| RIVM_H_2017-18 | MSDLAREITPVNIEEELKSSYLDYAMSVIVGRALPDVRDGLKPVHRRVLYAMNVLGNDWN |
| RIVM_H_2017-19 | MSDLAREITPVNIEEELKSSYLDYAMSVIVGRALPDVRDGLKPVHRRVLYAMNVLGNDWN |
| 15EP001483 | MSDLAREITPVNIEEELKSSYLDYAMSVIVGRALPDVRDGLKPVHRRVLYAMNVLGNDWN |
| 17EP002363 | MSDLAREITPVNIEEELKSSYLDYAMSVIVGRALPDVRDGLKPVHRRVLYAMNVLGNDWN |
| S_0812_17 | MSDLAREITPVNIEEELKSSYLDYAMSVIVGRALPDVRDGLKPVHRRVLYAMNVLGNDWN |
| SRR1957844 | MSDLAREITPVNIEEELKSSYLDYAMSVIVGRALPDVRDGLKPVHRRVLYAMNVLGNDWN |
| SRR1958654 | MSDLAREITPVNIEEELKSSYLDYAMSVIVGRALPDVRDGLKPVHRRVLYAMNVLGNDWN |
| SRR1965077 | MSDLAREITPVNIEEELKSSYLDYAMSVIVGRALPDVRDGLKPVHRRVLYAMNVLGNDWN |
| SRR1966369 | MSDLAREITPVNIEEELKSSYLDYAMSVIVGRALPDVRDGLKPVHRRVLYAMNVLGNDWN |
| SRR1967117 | MSDLAREITPVNIEEELKSSYLDYAMSVIVGRALPDVRDGLKPVHRRVLYAMNVLGNDWN |
| SRR1967922 | MSDLAREITPVNIEEELKSSYLDYAMSVIVGRALPDVRDGLKPVHRRVLYAMNVLGNDWN |
| SRR8704720 | MSDLAREITPVNIEEELKSSYLDYAMSVIVGRALPDVRDGLKPVHRRVLYAMNVLGNDWN |
| SRR7216071 | MSDLAREITPVNIEEELKSSYLDYAMSVIVGRALPDVRDGLKPVHRRVLYAMNVLGNDWN |
| SRR7349175 | MSDLAREITPVNIEEELKSSYLDYAMSVIVGRALPDVRDGLKPVHRRVLYAMNVLGNDWN |
| SRR7523148 | MSDLAREITPVNIEEELKSSYLDYAMSVIVGRALPDVRDGLKPVHRRVLYAMNVLGNDWN |
| SRR7523854 | MSDLAREITPVNIEEELKSSYLDYAMSVIVGRALPDVRDGLKPVHRRVLYAMNVLGNDWN |
| 313865 | MSDLAREITPVNIEEELKSSYLDYAMSVIVGRALPDVRDGLKPVHRRVLYAMNVLGNDWN |
| SRR7277793 | MSDLAREITPVNIEEELKSSYLDYAMSVIVGRALPDVRDGLKPVHRRVLYAMNVLGNDWN |
| SRR7343877 | MSDLAREITPVNIEEELKSSYLDYAMSVIVGRALPDVRDGLKPVHRRVLYAMNVLGNDWN |
| SRR7351477 | MSDLAREITPVNIEEELKSSYLDYAMSVIVGRALPDVRDGLKPVHRRVLYAMNVLGNDWN |
| SRR5583183 | MSDLAREITPVNIEEELKSSYLDYAMSVIVGRALPDVRDGLKPVHRRVLYAMNVLGNDWN |
| SRR5585240 | MSDLAREITPVNIEEELKSSYLDYAMSVIVGRALPDVRDGLKPVHRRVLYAMNVLGNDWN |
| SRR7284317 | MSDLAREITPVNIEEELKSSYLDYAMSVIVGRALPDVRDGLKPVHRRVLYAMNVLGNDWN |
| SRR7299161 | MSDLAREITPVNIEEELKSSYLDYAMSVIVGRALPDVRDGLKPVHRRVLYAMNVLGNDWN |
| SRR7401730 | MSDLAREITPVNIEEELKSSYLDYAMSVIVGRALPDVRDGLKPVHRRVLYAMNVLGNDWN |
| SRR7469092 | MSDLAREITPVNIEEELKSSYLDYAMSVIVGRALPDVRDGLKPVHRRVLYAMNVLGNDWN |
| SRR7879556 | MSDLAREITPVNIEEELKSSYLDYAMSVIVGRALPDVRDGLKPVHRRVLYAMNVLGNDWN |
| SRR8526100 | MSDLAREITPVNIEEELKSSYLDYAMSVIVGRALPDVRDGLKPVHRRVLYAMNVLGNDWN |
| SRR8553991 | MSDLAREITPVNIEEELKSSYLDYAMSVIVGRALPDVRDGLKPVHRRVLYAMNVLGNDWN |
| SRR7842487 | MSDLAREITPVNIEEELKSSYLDYAMSVIVGRALPDVRDGLKPVHRRVLYAMNVLGNDWN |
| SRR8054524 | MSDLAREITPVNIEEELKSSYLDYAMSVIVGRALPDVRDGLKPVHRRVLYAMNVLGNDWN |
| SRR8054525 | MSDLAREITPVNIEEELKSSYLDYAMSVIVGRALPDVRDGLKPVHRRVLYAMNVLGNDWN |
| SRR8524733 | MSDLAREITPVNIEEELKSSYLDYAMSVIVGRALPDVRDGLKPVHRRVLYAMNVLGNDWN |
| SRR4093291 | MSDLAREITPVNIEEELKSSYLDYAMSVIVGRALPDVRDGLKPVHRRVLYAMNVLGNDWN |
| SRR4245549 | MSDLAREITPVNIEEELKSSYLDYAMSVIVGRALPDVRDGLKPVHRRVLYAMNVLGNDWN |
| SRR3057154 | MSDLAREITPVNIEEELKSSYLDYAMSVIVGRALPDVRDGLKPVHRRVLYAMNVLGNDWN |
| SRR1726150 | MSDLAREITPVNIEEELKSSYLDYAMSVIVGRALPDVRDGLKPVHRRVLYAMNVLGNDWN |
| SRR1996141 | MSDLAREITPVNIEEELKSSYLDYAMSVIVGRALPDVRDGLKPVHRRVLYAMNVLGNDWN |
| SRR1107842 | MSDLAREITPVNIEEELKSSYLDYAMSVIVGRALPDVRDGLKPVHRRVLYAMNVLGNDWN |
| SRR1157587 | MSDLAREITPVNIEEELKSSYLDYAMSVIVGRALPDVRDGLKPVHRRVLYAMNVLGNDWN |
| SRR3027706 | MSDLAREITPVNIEEELKSSYLDYAMSVIVGRALPDVRDGLKPVHRRVLYAMNVLGNDWN |
| SRR3027707 | MSDLAREITPVNIEEELKSSYLDYAMSVIVGRALPDVRDGLKPVHRRVLYAMNVLGNDWN |
| SRR3027708 | MSDLAREITPVNIEEELKSSYLDYAMSVIVGRALPDVRDGLKPVHRRVLYAMNVLGNDWN |
| SRR3027710 | MSDLAREITPVNIEEELKSSYLDYAMSVIVGRALPDVRDGLKPVHRRVLYAMNVLGNDWN |
| SRR3027711 | MSDLAREITPVNIEEELKSSYLDYAMSVIVGRALPDVRDGLKPVHRRVLYAMNVLGNDWN |
| SRR3027716 | MSDLAREITPVNIEEELKSSYLDYAMSVIVGRALPDVRDGLKPVHRRVLYAMNVLGNDWN |
| SRR3027717 | MSDLAREITPVNIEEELKSSYLDYAMSVIVGRALPDVRDGLKPVHRRVLYAMNVLGNDWN |
| SRR3027719 | MSDLAREITPVNIEEELKSSYLDYAMSVIVGRALPDVRDGLKPVHRRVLYAMNVLGNDWN |
| SRR3027721 | MSDLAREITPVNIEEELKSSYLDYAMSVIVGRALPDVRDGLKPVHRRVLYAMNVLGNDWN |
| SRR3027723 | MSDLAREITPVNIEEELKSSYLDYAMSVIVGRALPDVRDGLKPVHRRVLYAMNVLGNDWN |
| SRR3115978 | MSDLAREITPVNIEEELKSSYLDYAMSVIVGRALPDVRDGLKPVHRRVLYAMNVLGNDWN |
| SRR2534093 | MSDLAREITPVNIEEELKSSYLDYAMSVIVGRALPDVRDGLKPVHRRVLYAMNVLGNDWN |
| SRR2534094 | MSDLAREITPVNIEEELKSSYLDYAMSVIVGRALPDVRDGLKPVHRRVLYAMNVLGNDWN |
| SRR2534095 | MSDLAREITPVNIEEELKSSYLDYAMSVIVGRALPDVRDGLKPVHRRVLYAMNVLGNDWN |
| SRR2534108 | MSDLAREITPVNIEEELKSSYLDYAMSVIVGRALPDVRDGLKPVHRRVLYAMNVLGNDWN |
| SRR1106464 | MSDLAREITPVNIEEELKSSYLDYAMSVIVGRALPDVRDGLKPVHRRVLYAMNVLGNDWN |
| SRR1106463 | MSDLAREITPVNIEEELKSSYLDYAMSVIVGRALPDVRDGLKPVHRRVLYAMNVLGNDWN |
| SRR6949610 | MSDLAREITPVNIEEELKSSYLDYAMSVIVGRALPDVRDGLKPVHRRVLYAMNVLGNDWN |
| SRR6950452 | MSDLAREITPVNIEEELKSSYLDYAMSVIVGRALPDVRDGLKPVHRRVLYAMNVLGNDWN |
| ERR2019831 | MSDLAREITPVNIEEELKSSYLDYAMSVIVGRALPDVRDGLKPVHRRVLYAMNVLGNDWN |
| SRR2085693 | MSDLAREITPVNIEEELKSSYLDYAMSVIVGRALPDVRDGLKPVHRRVLYAMNVLGNDWN |
| SRR2086898 | MSDLAREITPVNIEEELKSSYLDYAMSVIVGRALPDVRDGLKPVHRRVLYAMNVLGNDWN |
| SRR2175312 | MSDLAREITPVNIEEELKSSYLDYAMSVIVGRALPDVRDGLKPVHRRVLYAMNVLGNDWN |
| SRR2175360 | MSDLAREITPVNIEEELKSSYLDYAMSVIVGRALPDVRDGLKPVHRRVLYAMNVLGNDWN |
| SRR5231997 | MSDLAREITPVNIEEELKSSYLDYAMSVIVGRALPDVRDGLKPVHRRVLYAMNVLGNDWN |
| SRR5232003 | MSDLAREITPVNIEEELKSSYLDYAMSVIVGRALPDVRDGLKPVHRRVLYAMNVLGNDWN |
| SRR5232015 | MSDLAREITPVNIEEELKSSYLDYAMSVIVGRALPDVRDGLKPVHRRVLYAMNVLGNDWN |
| SRR949434 | MSDLAREITPVNIEEELKSSYLDYAMSVIVGRALPDVRDGLKPVHRRVLYAMNVLGNDWN |
| SRR3216575 | MSDLAREITPVNIEEELKSSYLDYAMSVIVGRALPDVRDGLKPVHRRVLYAMNVLGNDWN |
| SRR5205342 | MSDLAREITPVNIEEELKSSYLDYAMSVIVGRALPDVRDGLKPVHRRVLYAMNVLGNDWN |
| SRR1501669 | MSDLAREITPVNIEEELKSSYLDYAMSVIVGRALPDVRDGLKPVHRRVLYAMNVLGNDWN |
| SRR5209740 | MSDLAREITPVNIEEELKSSYLDYAMSVIVGRALPDVRDGLKPVHRRVLYAMNVLGNDWN |
| SRR3240355 | MSDLAREITPVNIEEELKSSYLDYAMSVIVGRALPDVRDGLKPVHRRVLYAMNVLGNDWN |
| SRR3392777 | MSDLAREITPVNIEEELKSSYLDYAMSVIVGRALPDVRDGLKPVHRRVLYAMNVLGNDWN |
| SRR3593671 | MSDLAREITPVNIEEELKSSYLDYAMSVIVGRALPDVRDGLKPVHRRVLYAMNVLGNDWN |
| SRR5413290 | MSDLAREITPVNIEEELKSSYLDYAMSVIVGRALPDVRDGLKPVHRRVLYAMNVLGNDWN |
| SRR5590269 | MSDLAREITPVNIEEELKSSYLDYAMSVIVGRALPDVRDGLKPVHRRVLYAMNVLGNDWN |
| SRR5812103 | MSDLAREITPVNIEEELKSSYLDYAMSVIVGRALPDVRDGLKPVHRRVLYAMNVLGNDWN |
| SRR2830941 | MSDLAREITPVNIEEELKSSYLDYAMSVIVGRALPDVRDGLKPVHRRVLYAMNVLGNDWN |
| SRR2830966 | MSDLAREITPVNIEEELKSSYLDYAMSVIVGRALPDVRDGLKPVHRRVLYAMNVLGNDWN |
| SRR3137270 | MSDLAREITPVNIEEELKSSYLDYAMSVIVGRALPDVRDGLKPVHRRVLYAMNVLGNDWN |
| SRR3137271 | MSDLAREITPVNIEEELKSSYLDYAMSVIVGRALPDVRDGLKPVHRRVLYAMNVLGNDWN |
| ERR526807 | MSDLAREITPVNIEEELKSSYLDYAMSVIVGRALPDVRDGLKPVHRRVLYAMNVLGNDWN |
| ERR2197922 | MSDLAREITPVNIEEELKSSYLDYAMSVIVGRALPDVRDGLKPVHRRVLYAMNVLGNDWN |
| ERR2197923 | MSDLAREITPVNIEEELKSSYLDYAMSVIVGRALPDVRDGLKPVHRRVLYAMNVLGNDWN |
| ERR2197924 | MSDLAREITPVNIEEELKSSYLDYAMSVIVGRALPDVRDGLKPVHRRVLYAMNVLGNDWN |
| ERR2197925 | MSDLAREITPVNIEEELKSSYLDYAMSVIVGRALPDVRDGLKPVHRRVLYAMNVLGNDWN |
| ERR2197927 | MSDLAREITPVNIEEELKSSYLDYAMSVIVGRALPDVRDGLKPVHRRVLYAMNVLGNDWN |
| ERR2197929 | MSDLAREITPVNIEEELKSSYLDYAMSVIVGRALPDVRDGLKPVHRRVLYAMNVLGNDWN |
| SRR1648149 | MSDLAREITPVNIEEELKSSYLDYAMSVIVGRALPDVRDGLKPVHRRVLYAMNVLGNDWN |
| SRR1048299 | MSDLAREITPVNIEEELKSSYLDYAMSVIVGRALPDVRDGLKPVHRRVLYAMNVLGNDWN |
| SRR1300677 | MSDLAREITPVNIEEELKSSYLDYAMSVIVGRALPDVRDGLKPVHRRVLYAMNVLGNDWN |
| SRR1288356 | MSDLAREITPVNIEEELKSSYLDYAMSVIVGRALPDVRDGLKPVHRRVLYAMNVLGNDWN |
| SRR7426190 | MSDLAREITPVNIEEELKSSYLDYAMSVIVGRALPDVRDGLKPVHRRVLYAMNVLGNDWN |
| SRR7426192 | MSDLAREITPVNIEEELKSSYLDYAMSVIVGRALPDVRDGLKPVHRRVLYAMNVLGNDWN |
| SRR7426193 | MSDLAREITPVNIEEELKSSYLDYAMSVIVGRALPDVRDGLKPVHRRVLYAMNVLGNDWN |
| SRR7441832 | MSDLAREITPVNIEEELKSSYLDYAMSVIVGRALPDVRDGLKPVHRRVLYAMNVLGNDWN |
| SRR7426179 | MSDLAREITPVNIEEELKSSYLDYAMSVIVGRALPDVRDGLKPVHRRVLYAMNVLGNDWN |
| SRR7439238 | MSDLAREITPVNIEEELKSSYLDYAMSVIVGRALPDVRDGLKPVHRRVLYAMNVLGNDWN |
| SRR7439244 | MSDLAREITPVNIEEELKSSYLDYAMSVIVGRALPDVRDGLKPVHRRVLYAMNVLGNDWN |
| SRR7439259 | MSDLAREITPVNIEEELKSSYLDYAMSVIVGRALPDVRDGLKPVHRRVLYAMNVLGNDWN |
| SRR7439260 | MSDLAREITPVNIEEELKSSYLDYAMSVIVGRALPDVRDGLKPVHRRVLYAMNVLGNDWN |
| SRR7441786 | MSDLAREITPVNIEEELKSSYLDYAMSVIVGRALPDVRDGLKPVHRRVLYAMNVLGNDWN |
| SRR7441797 | MSDLAREITPVNIEEELKSSYLDYAMSVIVGRALPDVRDGLKPVHRRVLYAMNVLGNDWN |
| ERR1759093 | MSDLAREITPVNIEEELKSSYLDYAMSVIVGRALPDVRDGLKPVHRRVLYAMNVLGNDWN |
| ERR2580275 | MSDLAREITPVNIEEELKSSYLDYAMSVIVGRALPDVRDGLKPVHRRVLYAMNVLGNDWN |
| ERR1759204 | MSDLAREITPVNIEEELKSSYLDYAMSVIVGRALPDVRDGLKPVHRRVLYAMNVLGNDWN |
| SRR1300699 | MSDLAREITPVNIEEELKSSYLDYAMSVIVGRALPDVRDGLKPVHRRVLYAMNVLGNDWN |
| S_0825_17 | MSDLAREITPVNIEEELKSSYLDYAMSVIVGRALPDVRDGLKPVHRRVLYAMNVLGNDWN |
| SRR1958215 | MSDLAREITPVNIEEELKSSYLDYAMSVIVGRALPDVRDGLKPVHRRVLYAMNVLGNDWN |
| SRR1958540 | MSDLAREITPVNIEEELKSSYLDYAMSVIVGRALPDVRDGLKPVHRRVLYAMNVLGNDWN |
| SRR1958636 | MSDLAREITPVNIEEELKSSYLDYAMSVIVGRALPDVRDGLKPVHRRVLYAMNVLGNDWN |
| SRR1959422 | MSDLAREITPVNIEEELKSSYLDYAMSVIVGRALPDVRDGLKPVHRRVLYAMNVLGNDWN |
| SRR1959427 | MSDLAREITPVNIEEELKSSYLDYAMSVIVGRALPDVRDGLKPVHRRVLYAMNVLGNDWN |
| SRR1960226 | MSDLAREITPVNIEEELKSSYLDYAMSVIVGRALPDVRDGLKPVHRRVLYAMNVLGNDWN |
| SRR1963498 | MSDLAREITPVNIEEELKSSYLDYAMSVIVGRALPDVRDGLKPVHRRVLYAMNVLGNDWN |
| SRR1965947 | MSDLAREITPVNIEEELKSSYLDYAMSVIVGRALPDVRDGLKPVHRRVLYAMNVLGNDWN |
| SRR1966125 | MSDLAREITPVNIEEELKSSYLDYAMSVIVGRALPDVRDGLKPVHRRVLYAMNVLGNDWN |
| SRR1966330 | MSDLAREITPVNIEEELKSSYLDYAMSVIVGRALPDVRDGLKPVHRRVLYAMNVLGNDWN |
| SRR1966565 | MSDLAREITPVNIEEELKSSYLDYAMSVIVGRALPDVRDGLKPVHRRVLYAMNVLGNDWN |
| SRR1966864 | MSDLAREITPVNIEEELKSSYLDYAMSVIVGRALPDVRDGLKPVHRRVLYAMNVLGNDWN |
| SRR1966989 | MSDLAREITPVNIEEELKSSYLDYAMSVIVGRALPDVRDGLKPVHRRVLYAMNVLGNDWN |
| SRR1967688 | MSDLAREITPVNIEEELKSSYLDYAMSVIVGRALPDVRDGLKPVHRRVLYAMNVLGNDWN |
| SRR1967733 | MSDLAREITPVNIEEELKSSYLDYAMSVIVGRALPDVRDGLKPVHRRVLYAMNVLGNDWN |
| SRR1967746 | MSDLAREITPVNIEEELKSSYLDYAMSVIVGRALPDVRDGLKPVHRRVLYAMNVLGNDWN |
| SRR1968341 | MSDLAREITPVNIEEELKSSYLDYAMSVIVGRALPDVRDGLKPVHRRVLYAMNVLGNDWN |
| SRR1968456 | MSDLAREITPVNIEEELKSSYLDYAMSVIVGRALPDVRDGLKPVHRRVLYAMNVLGNDWN |
| SRR1968465 | MSDLAREITPVNIEEELKSSYLDYAMSVIVGRALPDVRDGLKPVHRRVLYAMNVLGNDWN |
| SRR1968761 | MSDLAREITPVNIEEELKSSYLDYAMSVIVGRALPDVRDGLKPVHRRVLYAMNVLGNDWN |
| SRR1969047 | MSDLAREITPVNIEEELKSSYLDYAMSVIVGRALPDVRDGLKPVHRRVLYAMNVLGNDWN |
| SRR1969255 | MSDLAREITPVNIEEELKSSYLDYAMSVIVGRALPDVRDGLKPVHRRVLYAMNVLGNDWN |
| SRR1969412 | MSDLAREITPVNIEEELKSSYLDYAMSVIVGRALPDVRDGLKPVHRRVLYAMNVLGNDWN |
| SRR1969524 | MSDLAREITPVNIEEELKSSYLDYAMSVIVGRALPDVRDGLKPVHRRVLYAMNVLGNDWN |
| SRR1969584 | MSDLAREITPVNIEEELKSSYLDYAMSVIVGRALPDVRDGLKPVHRRVLYAMNVLGNDWN |
| SRR1969648 | MSDLAREITPVNIEEELKSSYLDYAMSVIVGRALPDVRDGLKPVHRRVLYAMNVLGNDWN |
| SRR1969804 | MSDLAREITPVNIEEELKSSYLDYAMSVIVGRALPDVRDGLKPVHRRVLYAMNVLGNDWN |
| SRR1970221 | MSDLAREITPVNIEEELKSSYLDYAMSVIVGRALPDVRDGLKPVHRRVLYAMNVLGNDWN |
| SRR1970268 | MSDLAREITPVNIEEELKSSYLDYAMSVIVGRALPDVRDGLKPVHRRVLYAMNVLGNDWN |
| SRR1965862 | MSDLAREITPVNIEEELKSSYLDYAMSVIVGRALPDVRDGLKPVHRRVLYAMNVLGNDWN |
| SRR1967363 | MSDLAREITPVNIEEELKSSYLDYAMSVIVGRALPDVRDGLKPVHRRVLYAMNVLGNDWN |
| SRR1968276 | MSDLAREITPVNIEEELKSSYLDYAMSVIVGRALPDVRDGLKPVHRRVLYAMNVLGNDWN |
| SRR1968967 | MSDLAREITPVNIEEELKSSYLDYAMSVIVGRALPDVRDGLKPVHRRVLYAMNVLGNDWN |
| SRR3321531 | MSDLAREITPVNIEEELKSSYLDYAMSVIVGRALPDVRDGLKPVHRRVLYAMNVLGNDWN |
| SRR3321883 | MSDLAREITPVNIEEELKSSYLDYAMSVIVGRALPDVRDGLKPVHRRVLYAMNVLGNDWN |
| SRR3322413 | MSDLAREITPVNIEEELKSSYLDYAMSVIVGRALPDVRDGLKPVHRRVLYAMNVLGNDWN |
| SRR3323012 | MSDLAREITPVNIEEELKSSYLDYAMSVIVGRALPDVRDGLKPVHRRVLYAMNVLGNDWN |
| SRR5194289 | MSDLAREITPVNIEEELKSSYLDYAMSVIVGRALPDVRDGLKPVHRRVLYAMNVLGNDWN |
| SRR7163798 | MSDLAREITPVNIEEELKSSYLDYAMSVIVGRALPDVRDGLKPVHRRVLYAMNVLGNDWN |
| SRR7172610 | MSDLAREITPVNIEEELKSSYLDYAMSVIVGRALPDVRDGLKPVHRRVLYAMNVLGNDWN |
| SRR7204568 | MSDLAREITPVNIEEELKSSYLDYAMSVIVGRALPDVRDGLKPVHRRVLYAMNVLGNDWN |
| SRR7223230 | MSDLAREITPVNIEEELKSSYLDYAMSVIVGRALPDVRDGLKPVHRRVLYAMNVLGNDWN |
| SRR7230675 | MSDLAREITPVNIEEELKSSYLDYAMSVIVGRALPDVRDGLKPVHRRVLYAMNVLGNDWN |
| SRR7278056 | MSDLAREITPVNIEEELKSSYLDYAMSVIVGRALPDVRDGLKPVHRRVLYAMNVLGNDWN |
| SRR7278086 | MSDLAREITPVNIEEELKSSYLDYAMSVIVGRALPDVRDGLKPVHRRVLYAMNVLGNDWN |
| SRR7285841 | MSDLAREITPVNIEEELKSSYLDYAMSVIVGRALPDVRDGLKPVHRRVLYAMNVLGNDWN |
| SRR7292625 | MSDLAREITPVNIEEELKSSYLDYAMSVIVGRALPDVRDGLKPVHRRVLYAMNVLGNDWN |
| SRR7292665 | MSDLAREITPVNIEEELKSSYLDYAMSVIVGRALPDVRDGLKPVHRRVLYAMNVLGNDWN |
| SRR7297965 | MSDLAREITPVNIEEELKSSYLDYAMSVIVGRALPDVRDGLKPVHRRVLYAMNVLGNDWN |
| SRR7350726 | MSDLAREITPVNIEEELKSSYLDYAMSVIVGRALPDVRDGLKPVHRRVLYAMNVLGNDWN |
| SRR7410328 | MSDLAREITPVNIEEELKSSYLDYAMSVIVGRALPDVRDGLKPVHRRVLYAMNVLGNDWN |
| SRR7474665 | MSDLAREITPVNIEEELKSSYLDYAMSVIVGRALPDVRDGLKPVHRRVLYAMNVLGNDWN |
| SRR7523184 | MSDLAREITPVNIEEELKSSYLDYAMSVIVGRALPDVRDGLKPVHRRVLYAMNVLGNDWN |
| SRR7187264 | MSDLAREITPVNIEEELKSSYLDYAMSVIVGRALPDVRDGLKPVHRRVLYAMNVLGNDWN |
| SRR7204445 | MSDLAREITPVNIEEELKSSYLDYAMSVIVGRALPDVRDGLKPVHRRVLYAMNVLGNDWN |
| SRR7285641 | MSDLAREITPVNIEEELKSSYLDYAMSVIVGRALPDVRDGLKPVHRRVLYAMNVLGNDWN |
| SRR7286695 | MSDLAREITPVNIEEELKSSYLDYAMSVIVGRALPDVRDGLKPVHRRVLYAMNVLGNDWN |
| SRR7286705 | MSDLAREITPVNIEEELKSSYLDYAMSVIVGRALPDVRDGLKPVHRRVLYAMNVLGNDWN |
| SRR7292931 | MSDLAREITPVNIEEELKSSYLDYAMSVIVGRALPDVRDGLKPVHRRVLYAMNVLGNDWN |
| SRR7310349 | MSDLAREITPVNIEEELKSSYLDYAMSVIVGRALPDVRDGLKPVHRRVLYAMNVLGNDWN |
| SRR7351616 | MSDLAREITPVNIEEELKSSYLDYAMSVIVGRALPDVRDGLKPVHRRVLYAMNVLGNDWN |
| SRR7414818 | MSDLAREITPVNIEEELKSSYLDYAMSVIVGRALPDVRDGLKPVHRRVLYAMNVLGNDWN |
| SRR7426480 | MSDLAREITPVNIEEELKSSYLDYAMSVIVGRALPDVRDGLKPVHRRVLYAMNVLGNDWN |
| SRR5584105 | MSDLAREITPVNIEEELKSSYLDYAMSVIVGRALPDVRDGLKPVHRRVLYAMNVLGNDWN |
| SRR5584565 | MSDLAREITPVNIEEELKSSYLDYAMSVIVGRALPDVRDGLKPVHRRVLYAMNVLGNDWN |
| SRR5584614 | MSDLAREITPVNIEEELKSSYLDYAMSVIVGRALPDVRDGLKPVHRRVLYAMNVLGNDWN |
| SRR5631543 | MSDLAREITPVNIEEELKSSYLDYAMSVIVGRALPDVRDGLKPVHRRVLYAMNVLGNDWN |
| SRR5631553 | MSDLAREITPVNIEEELKSSYLDYAMSVIVGRALPDVRDGLKPVHRRVLYAMNVLGNDWN |
| SRR7123196 | MSDLAREITPVNIEEELKSSYLDYAMSVIVGRALPDVRDGLKPVHRRVLYAMNVLGNDWN |
| SRR7163819 | MSDLAREITPVNIEEELKSSYLDYAMSVIVGRALPDVRDGLKPVHRRVLYAMNVLGNDWN |
| SRR7163920 | MSDLAREITPVNIEEELKSSYLDYAMSVIVGRALPDVRDGLKPVHRRVLYAMNVLGNDWN |
| SRR7209528 | MSDLAREITPVNIEEELKSSYLDYAMSVIVGRALPDVRDGLKPVHRRVLYAMNVLGNDWN |
| SRR7249868 | MSDLAREITPVNIEEELKSSYLDYAMSVIVGRALPDVRDGLKPVHRRVLYAMNVLGNDWN |
| SRR7278088 | MSDLAREITPVNIEEELKSSYLDYAMSVIVGRALPDVRDGLKPVHRRVLYAMNVLGNDWN |
| SRR7285788 | MSDLAREITPVNIEEELKSSYLDYAMSVIVGRALPDVRDGLKPVHRRVLYAMNVLGNDWN |
| SRR7286789 | MSDLAREITPVNIEEELKSSYLDYAMSVIVGRALPDVRDGLKPVHRRVLYAMNVLGNDWN |
| SRR7286886 | MSDLAREITPVNIEEELKSSYLDYAMSVIVGRALPDVRDGLKPVHRRVLYAMNVLGNDWN |
| SRR7310632 | MSDLAREITPVNIEEELKSSYLDYAMSVIVGRALPDVRDGLKPVHRRVLYAMNVLGNDWN |
| SRR7350631 | MSDLAREITPVNIEEELKSSYLDYAMSVIVGRALPDVRDGLKPVHRRVLYAMNVLGNDWN |
| SRR7458741 | MSDLAREITPVNIEEELKSSYLDYAMSVIVGRALPDVRDGLKPVHRRVLYAMNVLGNDWN |
| SRR7480280 | MSDLAREITPVNIEEELKSSYLDYAMSVIVGRALPDVRDGLKPVHRRVLYAMNVLGNDWN |
| SRR7523660 | MSDLAREITPVNIEEELKSSYLDYAMSVIVGRALPDVRDGLKPVHRRVLYAMNVLGNDWN |
| SRR7523775 | MSDLAREITPVNIEEELKSSYLDYAMSVIVGRALPDVRDGLKPVHRRVLYAMNVLGNDWN |
| SRR7251101 | MSDLAREITPVNIEEELKSSYLDYAMSVIVGRALPDVRDGLKPVHRRVLYAMNVLGNDWN |
| SRR7284299 | MSDLAREITPVNIEEELKSSYLDYAMSVIVGRALPDVRDGLKPVHRRVLYAMNVLGNDWN |
| SRR7285738 | MSDLAREITPVNIEEELKSSYLDYAMSVIVGRALPDVRDGLKPVHRRVLYAMNVLGNDWN |
| SRR7310640 | MSDLAREITPVNIEEELKSSYLDYAMSVIVGRALPDVRDGLKPVHRRVLYAMNVLGNDWN |
| SRR7349159 | MSDLAREITPVNIEEELKSSYLDYAMSVIVGRALPDVRDGLKPVHRRVLYAMNVLGNDWN |
| SRR7474873 | MSDLAREITPVNIEEELKSSYLDYAMSVIVGRALPDVRDGLKPVHRRVLYAMNVLGNDWN |
| SRR7495689 | MSDLAREITPVNIEEELKSSYLDYAMSVIVGRALPDVRDGLKPVHRRVLYAMNVLGNDWN |
| SRR7495752 | MSDLAREITPVNIEEELKSSYLDYAMSVIVGRALPDVRDGLKPVHRRVLYAMNVLGNDWN |
| ----------------------------------------------------------------------------- | |
| S16BD08730 | KAYKKSARVVGDVIGKYHPHGDFAVYGTIVRMAQPFSLRYMLVDGQGNFGSIDGDSAAAM |
| S18BD00684 | KAYKKSARVVGDVIGKYHPHGDFAVYYTIVRMAQPFSLRYMLVDGQGNFGSIDGDSAAAM |
| S18BD03994 | KAYKKSARVVGDVIGKYHPHGDFAVYGTIVRMAQPFSLRYMLVDGQGNFGSIDGDSAAAM |
| S18BD05011 | KAYKKSARVVGDVIGKYHPHGDFAVYGTIVRMAQPFSLRYMLVDGQGNFGSIDGDSAAAM |
| RKI_16-03723 | KAYKKSARVVGDVIGKYHPHGDFAVYYTIVRMAQPFSLRYMLVDGQGNFGSIDGDSAAAM |
| RKI_16-04315 | KAYKKSARVVGDVIGKYHPHGDFAVYGTIVRMAQPFSLRYMLVDGQGNFGSIDGDSAAAM |
| RKI_17-02304 | KAYKKSARVVGDVIGKYHPHGDFAVYGTIVRMAQPFSLRYMLVDGQGNFGSIDGDSAAAM |
| RKI_17-02411 | KAYKKSARVVGDVIGKYHPHGDFAVYGTIVRMAQPFSLRYMLVDGQGNFGSIDGDSAAAM |
| RKI_17-02757 | KAYKKSARVVGDVIGKYHPHGDFAVYGTIVRMAQPFSLRYMLVDGQGNFGSIDGDSAAAM |
| RKI_17-04797 | KAYKKSARVVGDVIGKYHPHGDFAVYGTIVRMAQPFSLRYMLVDGQGNFGSIDGDSAAAM |
| RKI_17-06869 | KAYKKSARVVGDVIGKYHPHGDFAVYGTIVRMAQPFSLRYMLVDGQGNFGSIDGDSAAAM |
| ERR2580277 | KAYKKSARVVGDVIGKYHPHGDFAVYGTIVRMAQPFSLRYMLVDGQGNFGSIDGDSAAAM |
| ERR2580276 | KAYKKSARVVGDVIGKYHPHGDFAVYYTIVRMAQPFSLRYMLVDGQGNFGSIDGDSAAAM |
| ERR2580273 | KAYKKSARVVGDVIGKYHPHGDFAVYYTIVRMAQPFSLRYMLVDGQGNFGSIDGDSAAAM |
| ERR2580274 | KAYKKSARVVGDVIGKYHPHGDFAVYGTIVRMAQPFSLRYMLVDGQGNFGSIDGDSAAAM |
| ERR2173656 | KAYKKSARVVGDVIGKYHPHGDFAVYGTIVRMAQPFSLRYMLVDGQGNFGSIDGDSAAAM |
| 17041676 | KAYKKSARVVGDVIGKYHPHGDFAVYNTIVRMAQPFSLRYMLVDGQGNFGSIDGDSAAAM |
| MT16-000061 | KAYKKSARVVGDVIGKYHPHGDFAVYGTIVRMAQPFSLRYMLVDGQGNFGSIDGDSAAAM |
| MT16-019416 | KAYKKSARVVGDVIGKYHPHGDFAVYGTIVRMAQPFSLRYMLVDGQGNFGSIDGDSAAAM |
| MT16-027865 | KAYKKSARVVGDVIGKYHPHGDFAVYGTIVRMAQPFSLRYMLVDGQGNFGSIDGDSAAAM |
| MT16-031693 | KAYKKSARVVGDVIGKYHPHGDFAVYGTIVRMAQPFSLRYMLVDGQGNFGSIDGDSAAAM |
| MT16-040253 | KAYKKSARVVGDVIGKYHPHGDFAVYGTIVRMAQPFSLRYMLVDGQGNFGSIDGDSAAAM |
| MT16-045379 | KAYKKSARVVGDVIGKYHPHGDFAVYGTIVRMAQPFSLRYMLVDGQGNFGSIDGDSAAAM |
| MT16-442728 | KAYKKSARVVGDVIGKYHPHGDFAVYGTIVRMAQPFSLRYMLVDGQGNFGSIDGDSAAAM |
| MT16-462857 | KAYKKSARVVGDVIGKYHPHGDFAVYGTIVRMAQPFSLRYMLVDGQGNFGSIDGDSAAAM |
| MT16-480196 | KAYKKSARVVGDVIGKYHPHGDFAVYGTIVRMAQPFSLRYMLVDGQGNFGSIDGDSAAAM |
| MT16-861555 | KAYKKSARVVGDVIGKYHPHGDFAVYGTIVRMAQPFSLRYMLVDGQGNFGSIDGDSAAAM |
| MT17-076833 | KAYKKSARVVGDVIGKYHPHGDFAVYGTIVRMAQPFSLRYMLVDGQGNFGSIDGDSAAAM |
| MT17-110677 | KAYKKSARVVGDVIGKYHPHGDFAVYGTIVRMAQPFSLRYMLVDGQGNFGSIDGDSAAAM |
| MT17-131730 | KAYKKSARVVGDVIGKYHPHGDFAVYGTIVRMAQPFSLRYMLVDGQGNFGSIDGDSAAAM |
| MT17-140890 | KAYKKSARVVGDVIGKYHPHGDFAVYGTIVRMAQPFSLRYMLVDGQGNFGSIDGDSAAAM |
| MT17-141840 | KAYKKSARVVGDVIGKYHPHGDFAVYGTIVRMAQPFSLRYMLVDGQGNFGSIDGDSAAAM |
| MT17-152488 | KAYKKSARVVGDVIGKYHPHGDFAVYGTIVRMAQPFSLRYMLVDGQGNFGSIDGDSAAAM |
| MT17-157311 | KAYKKSARVVGDVIGKYHPHGDFAVYGTIVRMAQPFSLRYMLVDGQGNFGSIDGDSAAAM |
| MT17-161645 | KAYKKSARVVGDVIGKYHPHGDFAVYGTIVRMAQPFSLRYMLVDGQGNFGSIDGDSAAAM |
| MT17-167951 | KAYKKSARVVGDVIGKYHPHGDFAVYGTIVRMAQPFSLRYMLVDGQGNFGSIDGDSAAAM |
| MT18-217732 | KAYKKSARVVGDVIGKYHPHGDFAVYGTIVRMAQPFSLRYMLVDGQGNFGSIDGDSAAAM |
| MT18-252580 | KAYKKSARVVGDVIGKYHPHGDFAVYGTIVRMAQPFSLRYMLVDGQGNFGSIDGDSAAAM |
| RIVM_H_2009-01 | KAYKKSARVVGDVIGKYHPHGDFAVYGTIVRMAQPFSLRYMLVDGQGNFGSIDGDSAAAM |
| RIVM_H_2010-01 | KAYKKSARVVGDVIGKYHPHGDFAVYYTIVRMAQPFSLRYMLVDGQGNFGSIDGDSAAAM |
| RIVM_H_2010-02 | KAYKKSARVVGDVIGKYHPHGDFAVYNTIVRMAQPFSLRYMLVDGQGNFGSIDGDSAAAM |
| RIVM_H_2011-01 | KAYKKSARVVGDVIGKYHPHGDFAVYNTIVRMAQPFSLRYMLVDGQGNFGSIDGDSAAAM |
| RIVM_H_2011-02 | KAYKKSARVVGDVIGKYHPHGDFAVYNTIVRMAQPFSLRYMLVDGQGNFGSIDGDSAAAM |
| RIVM_H_2011-03 | KAYKKSARVVGDVIGKYHPHGDFAVYNTIVRMAQPFSLRYMLVDGQGNFGSIDGDSAAAM |
| RIVM_H_2013-01 | KAYKKSARVVGDVIGKYHPHGDFAVYNTIVRMAQPFSLRYMLVDGQGNFGSIDGDSAAAM |
| RIVM_H_2013-02 | KAYKKSARVVGDVIGKYHPHGDFAVYNTIVRMAQPFSLRYMLVDGQGNFGSIDGDSAAAM |
| RIVM_H_2014-01 | KAYKKSARVVGDVIGKYHPHGDFAVYNTIVRMAQPFSLRYMLVDGQGNFGSIDGDSAAAM |
| RIVM_H_2014-02 | KAYKKSARVVGDVIGKYHPHGDFAVYNTIVRMAQPFSLRYMLVDGQGNFGSIDGDSAAAM |
| RIVM_H_2016-01 | KAYKKSARVVGDVIGKYHPHGDFAVYYTIVRMAQPFSLRYMLVDGQGNFGSIDGDSAAAM |
| RIVM_H_2016-02 | KAYKKSARVVGDVIGKYHPHGDFAVYNTIVRMAQPFSLRYMLVDGQGNFGSIDGDSAAAM |
| RIVM_H_2016-03 | KAYKKSARVVGDVIGKYHPHGDFAVYNTIVRMAQPFSLRYMLVDGQGNFGSIDGDSAAAM |
| RIVM_H_2016-04 | KAYKKSARVVGDVIGKYHPHGDFAVYGTIVRMAQPFSLRYMLVDGQGNFGSIDGDSAAAM |
| RIVM_H_2016-05 | KAYKKSARVVGDVIGKYHPHGDFAVYNTIVRMAQPFSLRYMLVDGQGNFGSIDGDSAAAM |
| RIVM_H_2016-06 | KAYKKSARVVGDVIGKYHPHGDFAVYNTIVRMAQPFSLRYMLVDGQGNFGSIDGDSAAAM |
| RIVM_H_2016-07 | KAYKKSARVVGDVIGKYHPHGDFAVYGTIVRMAQPFSLRYMLVDGQGNFGSIDGDSAAAM |
| RIVM_H_2016-08 | KAYKKSARVVGDVIGKYHPHGDFAVYGTIVRMAQPFSLRYMLVDGQGNFGSIDGDSAAAM |
| RIVM_H_2016-09 | KAYKKSARVVGDVIGKYHPHGDFAVYGTIVRMAQPFSLRYMLVDGQGNFGSIDGDSAAAM |
| RIVM_H_2016-10 | KAYKKSARVVGDVIGKYHPHGDFAVYNTIVRMAQPFSLRYMLVDGQGNFGSIDGDSAAAM |
| RIVM_H_2016-11 | KAYKKSARVVGDVIGKYHPHGDFAVYGTIVRMAQPFSLRYMLVDGQGNFGSIDGDSAAAM |
| RIVM_H_2016-12 | KAYKKSARVVGDVIGKYHPHGDFAVYGTIVRMAQPFSLRYMLVDGQGNFGSIDGDSAAAM |
| RIVM_H_2016-13 | KAYKKSARVVGDVIGKYHPHGDFAVYGTIVRMAQPFSLRYMLVDGQGNFGSIDGDSAAAM |
| RIVM_H_2016-14 | KAYKKSARVVGDVIGKYHPHGDFAVYGTIVRMAQPFSLRYMLVDGQGNFGSIDGDSAAAM |
| RIVM_H_2016-15 | KAYKKSARVVGDVIGKYHPHGDFAVYGTIVRMAQPFSLRYMLVDGQGNFGSIDGDSAAAM |
| RIVM_H_2017-01 | KAYKKSARVVGDVIGKYHPHGDFAVYNTIVRMAQPFSLRYMLVDGQGNFGSIDGDSAAAM |
| RIVM_H_2017-02 | KAYKKSARVVGDVIGKYHPHGDFAVYGTIVRMAQPFSLRYMLVDGQGNFGSIDGDSAAAM |
| RIVM_H_2017-03 | KAYKKSARVVGDVIGKYHPHGDFAVYGTIVRMAQPFSLRYMLVDGQGNFGSIDGDSAAAM |
| RIVM_H_2017-04 | KAYKKSARVVGDVIGKYHPHGDFAVYNTIVRMAQPFSLRYMLVDGQGNFGSIDGDSAAAM |
| RIVM_H_2017-05 | KAYKKSARVVGDVIGKYHPHGDFAVYGTIVRMAQPFSLRYMLVDGQGNFGSIDGDSAAAM |
| RIVM_H_2017-06 | KAYKKSARVVGDVIGKYHPHGDFAVYYTIVRMAQPFSLRYMLVDGQGNFGSIDGDSAAAM |
| RIVM_H_2017-07 | KAYKKSARVVGDVIGKYHPHGDFAVYGTIVRMAQPFSLRYMLVDGQGNFGSIDGDSAAAM |
| RIVM_H_2017-08 | KAYKKSARVVGDVIGKYHPHGDFAVYGTIVRMAQPFSLRYMLVDGQGNFGSIDGDSAAAM |
| RIVM_H_2017-09 | KAYKKSARVVGDVIGKYHPHGDFAVYGTIVRMAQPFSLRYMLVDGQGNFGSIDGDSAAAM |
| RIVM_H_2017-10 | KAYKKSARVVGDVIGKYHPHGDFAVYGTIVRMAQPFSLRYMLVDGQGNFGSIDGDSAAAM |
| RIVM_H_2017-11 | KAYKKSARVVGDVIGKYHPHGDFAVYGTIVRMAQPFSLRYMLVDGQGNFGSIDGDSAAAM |
| RIVM_H_2017-12 | KAYKKSARVVGDVIGKYHPHGDFAVYGTIVRMAQPFSLRYMLVDGQGNFGSIDGDSAAAM |
| RIVM_H_2017-13 | KAYKKSARVVGDVIGKYHPHGDFAVYGTIVRMAQPFSLRYMLVDGQGNFGSIDGDSAAAM |
| RIVM_H_2017-14 | KAYKKSARVVGDVIGKYHPHGDFAVYGTIVRMAQPFSLRYMLVDGQGNFGSIDGDSAAAM |
| RIVM_H_2017-15 | KAYKKSARVVGDVIGKYHPHGDFAVYGTIVRMAQPFSLRYMLVDGQGNFGSIDGDSAAAM |
| RIVM_H_2017-16 | KAYKKSARVVGDVIGKYHPHGDFAVYGTIVRMAQPFSLRYMLVDGQGNFGSIDGDSAAAM |
| RIVM_H_2017-17 | KAYKKSARVVGDVIGKYHPHGDFAVYNTIVRMAQPFSLRYMLVDGQGNFGSIDGDSAAAM |
| RIVM_H_2017-18 | KAYKKSARVVGDVIGKYHPHGDFAVYNTIVRMAQPFSLRYMLVDGQGNFGSIDGDSAAAM |
| RIVM_H_2017-19 | KAYKKSARVVGDVIGKYHPHGDFAVYGTIVRMAQPFSLRYMLVDGQGNFGSIDGDSAAAM |
| 15EP001483 | KAYKKSARVVGDVIGKYHPHGDFAVYGTIVRMAQPFSLRYMLVDGQGNFGSIDGDSAAAM |
| 17EP002363 | KAYKKSARVVGDVIGKYHPHGDFAVYGTIVRMAQPFSLRYMLVDGQGNFGSIDGDSAAAM |
| S_0812_17 | KAYKKSARVVGDVIGKYHPHGDFAVYYTIVRMAQPFSLRYMLVDGQGNFGSIDGDSAAAM |
| SRR1957844 | KAYKKSARVVGDVIGKYHPHGDFAVYGTIVRMAQPFSLRYMLVDGQGNFGSIDGDSAAAM |
| SRR1958654 | KAYKKSARVVGDVIGKYHPHGDFAVYGTIVRMAQPFSLRYMLVDGQGNFGSIDGDSAAAM |
| SRR1965077 | KAYKKSARVVGDVIGKYHPHGDFAVYGTIVRMAQPFSLRYMLVDGQGNFGSIDGDSAAAM |
| SRR1966369 | KAYKKSARVVGDVIGKYHPHGDFAVYGTIVRMAQPFSLRYMLVDGQGNFGSIDGDSAAAM |
| SRR1967117 | KAYKKSARVVGDVIGKYHPHGDFAVYGTIVRMAQPFSLRYMLVDGQGNFGSIDGDSAAAM |
| SRR1967922 | KAYKKSARVVGDVIGKYHPHGDFAVYGTIVRMAQPFSLRYMLVDGQGNFGSIDGDSAAAM |
| SRR8704720 | KAYKKSARVVGDVIGKYHPHGDFAVYGTIVRMAQPFSLRYMLVDGQGNFGSIDGDSAAAM |
| SRR7216071 | KAYKKSARVVGDVIGKYHPHGDFAVYGTIVRMAQPFSLRYMLVDGQGNFGSIDGDSAAAM |
| SRR7349175 | KAYKKSARVVGDVIGKYHPHGDFAVYNTIVRMAQPFSLRYMLVDGQGNFGSIDGDSAAAM |
| SRR7523148 | KAYKKSARVVGDVIGKYHPHGDFAVYGTIVRMAQPFSLRYMLVDGQGNFGSIDGDSAAAM |
| SRR7523854 | KAYKKSARVVGDVIGKYHPHGDFAVYGTIVRMAQPFSLRYMLVDGQGNFGSIDGDSAAAM |
| 313865 | KAYKKSARVVGDVIGKYHPHGDFAVYGTIVRMAQPFSLRYMLVDGQGNFGSIDGDSAAAM |
| SRR7277793 | KAYKKSARVVGDVIGKYHPHGDFAVYGTIVRMAQPFSLRYMLVDGQGNFGSIDGDSAAAM |
| SRR7343877 | KAYKKSARVVGDVIGKYHPHGDFAVYYTIVRMAQPFSLRYMLVDGQGNFGSIDGDSAAAM |
| SRR7351477 | KAYKKSARVVGDVIGKYHPHGDFAVYGTIVRMAQPFSLRYMLVDGQGNFGSIDGDSAAAM |
| SRR5583183 | KAYKKSARVVGDVIGKYHPHGDFAVYGTIVRMAQPFSLRYMLVDGQGNFGSIDGDSAAAM |
| SRR5585240 | KAYKKSARVVGDVIGKYHPHGDFAVYGTIVRMAQPFSLRYMLVDGQGNFGSIDGDSAAAM |
| SRR7284317 | KAYKKSARVVGDVIGKYHPHGDFAVYGTIVRMAQPFSLRYMLVDGQGNFGSIDGDSAAAM |
| SRR7299161 | KAYKKSARVVGDVIGKYHPHGDFAVYGTIVRMAQPFSLRYMLVDGQGNFGSIDGDSAAAM |
| SRR7401730 | KAYKKSARVVGDVIGKYHPHGDFAVYGTIVRMAQPFSLRYMLVDGQGNFGSIDGDSAAAM |
| SRR7469092 | KAYKKSARVVGDVIGKYHPHGDFAVYGTIVRMAQPFSLRYMLVDGQGNFGSIDGDSAAAM |
| SRR7879556 | KAYKKSARVVGDVIGKYHPHGDFAVYGTIVRMAQPFSLRYMLVDGQGNFGSIDGDSAAAM |
| SRR8526100 | KAYKKSARVVGDVIGKYHPHGDFAVYGTIVRMAQPFSLRYMLVDGQGNFGSIDGDSAAAM |
| SRR8553991 | KAYKKSARVVGDVIGKYHPHGDFAVYGTIVRMAQPFSLRYMLVDGQGNFGSIDGDSAAAM |
| SRR7842487 | KAYKKSARVVGDVIGKYHPHGDFAVYGTIVRMAQPFSLRYMLVDGQGNFGSIDGDSAAAM |
| SRR8054524 | KAYKKSARVVGDVIGKYHPHGDFAVYGTIVRMAQPFSLRYMLVDGQGNFGSIDGDSAAAM |
| SRR8054525 | KAYKKSARVVGDVIGKYHPHGDFAVYGTIVRMAQPFSLRYMLVDGQGNFGSIDGDSAAAM |
| SRR8524733 | KAYKKSARVVGDVIGKYHPHGDFAVYGTIVRMAQPFSLRYMLVDGQGNFGSIDGDSAAAM |
| SRR4093291 | KAYKKSARVVGDVIGKYHPHGDFAVYDTIVRMAQPFSLRYMLVDGQGNFGSIDGDSAAAM |
| SRR4245549 | KAYKKSARVVGDVIGKYHPHGDFAVYDTIVRMAQPFSLRYMLVDGQGNFGSIDGDSAAAM |
| SRR3057154 | KAYKKSARVVGDVIGKYHPHGDFAVYGTIVRMAQPFSLRYMLVDGQGNFGSIDGDSAAAM |
| SRR1726150 | KAYKKSARVVGDVIGKYHPHGDFAVYGTIVRMAQPFSLRYMLVDGQGNFGSIDGDSAAAM |
| SRR1996141 | KAYKKSARVVGDVIGKYHPHGDFAVYGTIVRMAQPFSLRYMLVDGQGNFGSIDGDSAAAM |
| SRR1107842 | KAYKKSARVVGDVIGKYHPHGDFAVYNTIVRMAQPFSLRYMLVDGQGNFGSIDGDSAAAM |
| SRR1157587 | KAYKKSARVVGDVIGKYHPHGDFAVYGTIVRMAQPFSLRYMLVDGQGNFGSIDGDSAAAM |
| SRR3027706 | KAYKKSARVVGDVIGKYHPHGDFAVYGTIVRMAQPFSLRYMLVDGQGNFGSIDGDSAAAM |
| SRR3027707 | KAYKKSARVVGDVIGKYHPHGDFAVYGTIVRMAQPFSLRYMLVDGQGNFGSIDGDSAAAM |
| SRR3027708 | KAYKKSARVVGDVIGKYHPHGDFAVYGTIVRMAQPFSLRYMLVDGQGNFGSIDGDSAAAM |
| SRR3027710 | KAYKKSARVVGDVIGKYHPHGDFAVYGTIVRMAQPFSLRYMLVDGQGNFGSIDGDSAAAM |
| SRR3027711 | KAYKKSARVVGDVIGKYHPHGDFAVYGTIVRMAQPFSLRYMLVDGQGNFGSIDGDSAAAM |
| SRR3027716 | KAYKKSARVVGDVIGKYHPHGDFAVYDTIVRMAQPFSLRYMLVDGQGNFGSIDGDSAAAM |
| SRR3027717 | KAYKKSARVVGDVIGKYHPHGDFAVYGTIVRMAQPFSLRYMLVDGQGNFGSIDGDSAAAM |
| SRR3027719 | KAYKKSARVVGDVIGKYHPHGDFAVYGTIVRMAQPFSLRYMLVDGQGNFGSIDGDSAAAM |
| SRR3027721 | KAYKKSARVVGDVIGKYHPHGDFAVYGTIVRMAQPFSLRYMLVDGQGNFGSIDGDSAAAM |
| SRR3027723 | KAYKKSARVVGDVIGKYHPHGDFAVYGTIVRMAQPFSLRYMLVDGQGNFGSIDGDSAAAM |
| SRR3115978 | KAYKKSARVVGDVIGKYHPHGDFAVYGTIVRMAQPFSLRYMLVDGQGNFGSIDGDSAAAM |
| SRR2534093 | KAYKKSARVVGDVIGKYHPHGDFAVYNTIVRMAQPFSLRYMLVDGQGNFGSIDGDSAAAM |
| SRR2534094 | KAYKKSARVVGDVIGKYHPHGDFAVYNTIVRMAQPFSLRYMLVDGQGNFGSIDGDSAAAM |
| SRR2534095 | KAYKKSARVVGDVIGKYHPHGDFAVYNTIVRMAQPFSLRYMLVDGQGNFGSIDGDSAAAM |
| SRR2534108 | KAYKKSARVVGDVIGKYHPHGDFAVYNTIVRMAQPFSLRYMLVDGQGNFGSIDGDSAAAM |
| SRR1106464 | KAYKKSARVVGDVIGKYHPHGDFAVYGTIVRMAQPFSLRYMLVDGQGNFGSIDGDSAAAM |
| SRR1106463 | KAYKKSARVVGDVIGKYHPHGDFAVYGTIVRMAQPFSLRYMLVDGQGNFGSIDGDSAAAM |
| SRR6949610 | KAYKKSARVVGDVIGKYHPHGDFAVYGTIVRMAQPFSLRYMLVDGQGNFGSIDGDSAAAM |
| SRR6950452 | KAYKKSARVVGDVIGKYHPHGDFAVYGTIVRMAQPFSLRYMLVDGQGNFGSIDGDSAAAM |
| ERR2019831 | KAYKKSARVVGDVIGKYHPHGDFAVYGTIVRMAQPFSLRYMLVDGQGNFGSIDGDSAAAM |
| SRR2085693 | KAYKKSARVVGDVIGKYHPHGDFAVYGTIVRMAQPFSLRYMLVDGQGNFGSIDGDSAAAM |
| SRR2086898 | KAYKKSARVVGDVIGKYHPHGDFAVYGTIVRMAQPFSLRYMLVDGQGNFGSIDGDSAAAM |
| SRR2175312 | KAYKKSARVVGDVIGKYHPHGDFAVYGTIVRMAQPFSLRYMLVDGQGNFGSIDGDSAAAM |
| SRR2175360 | KAYKKSARVVGDVIGKYHPHGDFAVYGTIVRMAQPFSLRYMLVDGQGNFGSIDGDSAAAM |
| SRR5231997 | KAYKKSARVVGDVIGKYHPHGDFAVYNTIVRMAQPFSLRYMLVDGQGNFGSIDGDSAAAM |
| SRR5232003 | KAYKKSARVVGDVIGKYHPHGDFAVYNTIVRMAQPFSLRYMLVDGQGNFGSIDGDSAAAM |
| SRR5232015 | KAYKKSARVVGDVIGKYHPHGDFAVYNTIVRMAQPFSLRYMLVDGQGNFGSIDGDSAAAM |
| SRR949434 | KAYKKSARVVGDVIGKYHPHGDFAVYGTIVRMAQPFSLRYMLVDGQGNFGSIDGDSAAAM |
| SRR3216575 | KAYKKSARVVGDVIGKYHPHGDFAVYYTIVRMAQPFSLRYMLVDGQGNFGSIDGDSAAAM |
| SRR5205342 | KAYKKSARVVGDVIGKYHPHGDFAVYGTIVRMAQPFSLRYMLVDGQGNFGSIDGDSAAAM |
| SRR1501669 | KAYKKSARVVGDVIGKYHPHGDFAVYGTIVRMAQPFSLRYMLVDGQGNFGSIDGDSAAAM |
| SRR5209740 | KAYKKSARVVGDVIGKYHPHGDFAVYNTIVRMAQPFSLRYMLVDGQGNFGSIDGDSAAAM |
| SRR3240355 | KAYKKSARVVGDVIGKYHPHGDFAVYYTIVRMAQPFSLRYMLVDGQGNFGSIDGDSAAAM |
| SRR3392777 | KAYKKSARVVGDVIGKYHPHGDFAVYNTIVRMAQPFSLRYMLVDGQGNFGSIDGDSAAAM |
| SRR3593671 | KAYKKSARVVGDVIGKYHPHGDFAVYNTIVRMAQPFSLRYMLVDGQGNFGSIDGDSAAAM |
| SRR5413290 | KAYKKSARVVGDVIGKYHPHGDFAVYNTIVRMAQPFSLRYMLVDGQGNFGSIDGDSAAAM |
| SRR5590269 | KAYKKSARVVGDVIGKYHPHGDFAVYNTIVRMAQPFSLRYMLVDGQGNFGSIDGDSAAAM |
| SRR5812103 | KAYKKSARVVGDVIGKYHPHGDFAVYGTIVRMAQPFSLRYMLVDGQGNFGSIDGDSAAAM |
| SRR2830941 | KAYKKSARVVGDVIGKYHPHGDFAVYGTIVRMAQPFSLRYMLVDGQGNFGSIDGDSAAAM |
| SRR2830966 | KAYKKSARVVGDVIGKYHPHGDFAVYGTIVRMAQPFSLRYMLVDGQGNFGSIDGDSAAAM |
| SRR3137270 | KAYKKSARVVGDVIGKYHPHGDFAVYGTIVRMAQPFSLRYMLVDGQGNFGSIDGDSAAAM |
| SRR3137271 | KAYKKSARVVGDVIGKYHPHGDFAVYGTIVRMAQPFSLRYMLVDGQGNFGSIDGDSAAAM |
| ERR526807 | KAYKKSARVVGDVIGKYHPHGDFAVYNTIVRMAQPFSLRYMLVDGQGNFGSIDGDSAAAM |
| ERR2197922 | KAYKKSARVVGDVIGKYHPHGDFAVYNTIVRMAQPFSLRYMLVDGQGNFGSIDGDSAAAM |
| ERR2197923 | KAYKKSARVVGDVIGKYHPHGDFAVYNTIVRMAQPFSLRYMLVDGQGNFGSIDGDSAAAM |
| ERR2197924 | KAYKKSARVVGDVIGKYHPHGDFAVYNTIVRMAQPFSLRYMLVDGQGNFGSIDGDSAAAM |
| ERR2197925 | KAYKKSARVVGDVIGKYHPHGDFAVYNTIVRMAQPFSLRYMLVDGQGNFGSIDGDSAAAM |
| ERR2197927 | KAYKKSARVVGDVIGKYHPHGDFAVYNTIVRMAQPFSLRYMLVDGQGNFGSIDGDSAAAM |
| ERR2197929 | KAYKKSARVVGDVIGKYHPHGDFAVYNTIVRMAQPFSLRYMLVDGQGNFGSIDGDSAAAM |
| SRR1648149 | KAYKKSARVVGDVIGKYHPHGDFAVYDTIVRMAQPFSLRYMLVDGQGNFGSIDGDSAAAM |
| SRR1048299 | KAYKKSARVVGDVIGKYHPHGDFAVYNTIVRMAQPFSLRYMLVDGQGNFGSIDGDSAAAM |
| SRR1300677 | KAYKKSARVVGDVIGKYHPHGDFAVYNTIVRMAQPFSLRYMLVDGQGNFGSIDGDSAAAM |
| SRR1288356 | KAYKKSARVVGDVIGKYHPHGDFAVYNTIVRMAQPFSLRYMLVDGQGNFGSIDGDSAAAM |
| SRR7426190 | KAYKKSARVVGDVIGKYHPHGDFAVYGTIVRMAQPFSLRYMLVDGQGNFGSIDGDSAAAM |
| SRR7426192 | KAYKKSARVVGDVIGKYHPHGDFAVYDTIVRMAQPFSLRYMLVDGQGNFGSIDGDSAAAM |
| SRR7426193 | KAYKKSARVVGDVIGKYHPHGDFAVYGTIVRMAQPFSLRYMLVDGQGNFGSIDGDSAAAM |
| SRR7441832 | KAYKKSARVVGDVIGKYHPHGDFAVYGTIVRMAQPFSLRYMLVDGQGNFGSIDGDSAAAM |
| SRR7426179 | KAYKKSARVVGDVIGKYHPHGDFAVYNTIVRMAQPFSLRYMLVDGQGNFGSIDGDSAAAM |
| SRR7439238 | KAYKKSARVVGDVIGKYHPHGDFAVYNTIVRMAQPFSLRYMLVDGQGNFGSIDGDSAAAM |
| SRR7439244 | KAYKKSARVVGDVIGKYHPHGDFAVYNTIVRMAQPFSLRYMLVDGQGNFGSIDGDSAAAM |
| SRR7439259 | KAYKKSARVVGDVIGKYHPHGDFAVYNTIVRMAQPFSLRYMLVDGQGNFGSIDGDSAAAM |
| SRR7439260 | KAYKKSARVVGDVIGKYHPHGDFAVYNTIVRMAQPFSLRYMLVDGQGNFGSIDGDSAAAM |
| SRR7441786 | KAYKKSARVVGDVIGKYHPHGDFAVYGTIVRMAQPFSLRYMLVDGQGNFGSIDGDSAAAM |
| SRR7441797 | KAYKKSARVVGDVIGKYHPHGDFAVYGTIVRMAQPFSLRYMLVDGQGNFGSIDGDSAAAM |
| ERR1759093 | KAYKKSARVVGDVIGKYHPHGDFAVYYTIVRMAQPFSLRYMLVDGQGNFGSIDGDSAAAM |
| ERR2580275 | KAYKKSARVVGDVIGKYHPHGDFAVYGTIVRMAQPFSLRYMLVDGQGNFGSIDGDSAAAM |
| ERR1759204 | KAYKKSARVVGDVIGKYHPHGDFAVYNTIVRMAQPFSLRYMLVDGQGNFGSIDGDSAAAM |
| SRR1300699 | KAYKKSARVVGDVIGKYHPHGDFAVYGTIVRMAQPFSLRYMLVDGQGNFGSIDGDSAAAM |
| S_0825_17 | KAYKKSARVVGDVIGKYHPHGDFAVYYTIVRMAQPFSLRYMLVDGQGNFGSIDGDSAAAM |
| SRR1958215 | KAYKKSARVVGDVIGKYHPHGDFAVYNTIVRMAQPFSLRYMLVDGQGNFGSIDGDSAAAM |
| SRR1958540 | KAYKKSARVVGDVIGKYHPHGDFAVYNTIVRMAQPFSLRYMLVDGQGNFGSIDGDSAAAM |
| SRR1958636 | KAYKKSARVVGDVIGKYHPHGDFAVYNTIVRMAQPFSLRYMLVDGQGNFGSIDGDSAAAM |
| SRR1959422 | KAYKKSARVVGDVIGKYHPHGDFAVYNTIVRMAQPFSLRYMLVDGQGNFGSIDGDSAAAM |
| SRR1959427 | KAYKKSARVVGDVIGKYHPHGDFAVYNTIVRMAQPFSLRYMLVDGQGNFGSIDGDSAAAM |
| SRR1960226 | KAYKKSARVVGDVIGKYHPHGDFAVYYTIVRMAQPFSLRYMLVDGQGNFGSIDGDSAAAM |
| SRR1963498 | KAYKKSARVVGDVIGKYHPHGDFAVYYTIVRMAQPFSLRYMLVDGQGNFGSIDGDSAAAM |
| SRR1965947 | KAYKKSARVVGDVIGKYHPHGDFAVYNTIVRMAQPFSLRYMLVDGQGNFGSIDGDSAAAM |
| SRR1966125 | KAYKKSARVVGDVIGKYHPHGDFAVYYTIVRMAQPFSLRYMLVDGQGNFGSIDGDSAAAM |
| SRR1966330 | KAYKKSARVVGDVIGKYHPHGDFAVYYTIVRMAQPFSLRYMLVDGQGNFGSIDGDSAAAM |
| SRR1966565 | KAYKKSARVVGDVIGKYHPHGDFAVYGTIVRMAQPFSLRYMLVDGQGNFGSIDGDSAAAM |
| SRR1966864 | KAYKKSARVVGDVIGKYHPHGDFAVYGTIVRMAQPFSLRYMLVDGQGNFGSIDGDSAAAM |
| SRR1966989 | KAYKKSARVVGDVIGKYHPHGDFAVYGTIVRMAQPFSLRYMLVDGQGNFGSIDGDSAAAM |
| SRR1967688 | KAYKKSARVVGDVIGKYHPHGDFAVYNTIVRMAQPFSLRYMLVDGQGNFGSIDGDSAAAM |
| SRR1967733 | KAYKKSARVVGDVIGKYHPHGDFAVYYTIVRMAQPFSLRYMLVDGQGNFGSIDGDSAAAM |
| SRR1967746 | KAYKKSARVVGDVIGKYHPHGDFAVYYTIVRMAQPFSLRYMLVDGQGNFGSIDGDSAAAM |
| SRR1968341 | KAYKKSARVVGDVIGKYHPHGDFAVYYTIVRMAQPFSLRYMLVDGQGNFGSIDGDSAAAM |
| SRR1968456 | KAYKKSARVVGDVIGKYHPHGDFAVYGTIVRMAQPFSLRYMLVDGQGNFGSIDGDSAAAM |
| SRR1968465 | KAYKKSARVVGDVIGKYHPHGDFAVYNTIVRMAQPFSLRYMLVDGQGNFGSIDGDSAAAM |
| SRR1968761 | KAYKKSARVVGDVIGKYHPHGDFAVYNTIVRMAQPFSLRYMLVDGQGNFGSIDGDSAAAM |
| SRR1969047 | KAYKKSARVVGDVIGKYHPHGDFAVYYTIVRMAQPFSLRYMLVDGQGNFGSIDGDSAAAM |
| SRR1969255 | KAYKKSARVVGDVIGKYHPHGDFAVYYTIVRMAQPFSLRYMLVDGQGNFGSIDGDSAAAM |
| SRR1969412 | KAYKKSARVVGDVIGKYHPHGDFAVYYTIVRMAQPFSLRYMLVDGQGNFGSIDGDSAAAM |
| SRR1969524 | KAYKKSARVVGDVIGKYHPHGDFAVYGTIVRMAQPFSLRYMLVDGQGNFGSIDGDSAAAM |
| SRR1969584 | KAYKKSARVVGDVIGKYHPHGDFAVYGTIVRMAQPFSLRYMLVDGQGNFGSIDGDSAAAM |
| SRR1969648 | KAYKKSARVVGDVIGKYHPHGDFAVYYTIVRMAQPFSLRYMLVDGQGNFGSIDGDSAAAM |
| SRR1969804 | KAYKKSARVVGDVIGKYHPHGDFAVYYTIVRMAQPFSLRYMLVDGQGNFGSIDGDSAAAM |
| SRR1970221 | KAYKKSARVVGDVIGKYHPHGDFAVYYTIVRMAQPFSLRYMLVDGQGNFGSIDGDSAAAM |
| SRR1970268 | KAYKKSARVVGDVIGKYHPHGDFAVYYTIVRMAQPFSLRYMLVDGQGNFGSIDGDSAAAM |
| SRR1965862 | KAYKKSARVVGDVIGKYHPHGDFAVYNTIVRMAQPFSLRYMLVDGQGNFGSIDGDSAAAM |
| SRR1967363 | KAYKKSARVVGDVIGKYHPHGDFAVYNTIVRMAQPFSLRYMLVDGQGNFGSIDGDSAAAM |
| SRR1968276 | KAYKKSARVVGDVIGKYHPHGDFAVYYTIVRMAQPFSLRYMLVDGQGNFGSIDGDSAAAM |
| SRR1968967 | KAYKKSARVVGDVIGKYHPHGDFAVYYTIVRMAQPFSLRYMLVDGQGNFGSIDGDSAAAM |
| SRR3321531 | KAYKKSARVVGDVIGKYHPHGDFAVYYTIVRMAQPFSLRYMLVDGQGNFGSIDGDSAAAM |
| SRR3321883 | KAYKKSARVVGDVIGKYHPHGDFAVYNTIVRMAQPFSLRYMLVDGQGNFGSIDGDSAAAM |
| SRR3322413 | KAYKKSARVVGDVIGKYHPHGDFAVYNTIVRMAQPFSLRYMLVDGQGNFGSIDGDSAAAM |
| SRR3323012 | KAYKKSARVVGDVIGKYHPHGDFAVYNTIVRMAQPFSLRYMLVDGQGNFGSIDGDSAAAM |
| SRR5194289 | KAYKKSARVVGDVIGKYHPHGDFAVYGTIVRMAQPFSLRYMLVDGQGNFGSIDGDSAAAM |
| SRR7163798 | KAYKKSARVVGDVIGKYHPHGDFAVYYTIVRMAQPFSLRYMLVDGQGNFGSIDGDSAAAM |
| SRR7172610 | KAYKKSARVVGDVIGKYHPHGDFAVYNTIVRMAQPFSLRYMLVDGQGNFGSIDGDSAAAM |
| SRR7204568 | KAYKKSARVVGDVIGKYHPHGDFAVYYTIVRMAQPFSLRYMLVDGQGNFGSIDGDSAAAM |
| SRR7223230 | KAYKKSARVVGDVIGKYHPHGDFAVYYTIVRMAQPFSLRYMLVDGQGNFGSIDGDSAAAM |
| SRR7230675 | KAYKKSARVVGDVIGKYHPHGDFAVYNTIVRMAQPFSLRYMLVDGQGNFGSIDGDSAAAM |
| SRR7278056 | KAYKKSARVVGDVIGKYHPHGDFAVYYTIVRMAQPFSLRYMLVDGQGNFGSIDGDSAAAM |
| SRR7278086 | KAYKKSARVVGDVIGKYHPHGDFAVYNTIVRMAQPFSLRYMLVDGQGNFGSIDGDSAAAM |
| SRR7285841 | KAYKKSARVVGDVIGKYHPHGDFAVYNTIVRMAQPFSLRYMLVDGQGNFGSIDGDSAAAM |
| SRR7292625 | KAYKKSARVVGDVIGKYHPHGDFAVYYTIVRMAQPFSLRYMLVDGQGNFGSIDGDSAAAM |
| SRR7292665 | KAYKKSARVVGDVIGKYHPHGDFAVYYTIVRMAQPFSLRYMLVDGQGNFGSIDGDSAAAM |
| SRR7297965 | KAYKKSARVVGDVIGKYHPHGDFAVYYTIVRMAQPFSLRYMLVDGQGNFGSIDGDSAAAM |
| SRR7350726 | KAYKKSARVVGDVIGKYHPHGDFAVYYTIVRMAQPFSLRYMLVDGQGNFGSIDGDSAAAM |
| SRR7410328 | KAYKKSARVVGDVIGKYHPHGDFAVYGTIVRMAQPFSLRYMLVDGQGNFGSIDGDSAAAM |
| SRR7474665 | KAYKKSARVVGDVIGKYHPHGDFAVYGTIVRMAQPFSLRYMLVDGQGNFGSIDGDSAAAM |
| SRR7523184 | KAYKKSARVVGDVIGKYHPHGDFAVYGTIVRMAQPFSLRYMLVDGQGNFGSIDGDSAAAM |
| SRR7187264 | KAYKKSARVVGDVIGKYHPHGDFAVYGTIVRMAQPFSLRYMLVDGQGNFGSIDGDSAAAM |
| SRR7204445 | KAYKKSARVVGDVIGKYHPHGDFAVYYTIVRMAQPFSLRYMLVDGQGNFGSIDGDSAAAM |
| SRR7285641 | KAYKKSARVVGDVIGKYHPHGDFAVYNTIVRMAQPFSLRYMLVDGQGNFGSIDGDSAAAM |
| SRR7286695 | KAYKKSARVVGDVIGKYHPHGDFAVYYTIVRMAQPFSLRYMLVDGQGNFGSIDGDSAAAM |
| SRR7286705 | KAYKKSARVVGDVIGKYHPHGDFAVYYTIVRMAQPFSLRYMLVDGQGNFGSIDGDSAAAM |
| SRR7292931 | KAYKKSARVVGDVIGKYHPHGDFAVYNTIVRMAQPFSLRYMLVDGQGNFGSIDGDSAAAM |
| SRR7310349 | KAYKKSARVVGDVIGKYHPHGDFAVYYTIVRMAQPFSLRYMLVDGQGNFGSIDGDSAAAM |
| SRR7351616 | KAYKKSARVVGDVIGKYHPHGDFAVYNTIVRMAQPFSLRYMLVDGQGNFGSIDGDSAAAM |
| SRR7414818 | KAYKKSARVVGDVIGKYHPHGDFAVYNTIVRMAQPFSLRYMLVDGQGNFGSIDGDSAAAM |
| SRR7426480 | KAYKKSARVVGDVIGKYHPHGDFAVYNTIVRMAQPFSLRYMLVDGQGNFGSIDGDSAAAM |
| SRR5584105 | KAYKKSARVVGDVIGKYHPHGDFAVYYTIVRMAQPFSLRYMLVDGQGNFGSIDGDSAAAM |
| SRR5584565 | KAYKKSARVVGDVIGKYHPHGDFAVYYTIVRMAQPFSLRYMLVDGQGNFGSIDGDSAAAM |
| SRR5584614 | KAYKKSARVVGDVIGKYHPHGDFAVYNTIVRMAQPFSLRYMLVDGQGNFGSIDGDSAAAM |
| SRR5631543 | KAYKKSARVVGDVIGKYHPHGDFAVYNTIVRMAQPFSLRYMLVDGQGNFGSIDGDSAAAM |
| SRR5631553 | KAYKKSARVVGDVIGKYHPHGDFAVYYTIVRMAQPFSLRYMLVDGQGNFGSIDGDSAAAM |
| SRR7123196 | KAYKKSARVVGDVIGKYHPHGDFAVYNTIVRMAQPFSLRYMLVDGQGNFGSIDGDSAAAM |
| SRR7163819 | KAYKKSARVVGDVIGKYHPHGDFAVYYTIVRMAQPFSLRYMLVDGQGNFGSIDGDSAAAM |
| SRR7163920 | KAYKKSARVVGDVIGKYHPHGDFAVYGTIVRMAQPFSLRYMLVDGQGNFGSIDGDSAAAM |
| SRR7209528 | KAYKKSARVVGDVIGKYHPHGDFAVYNTIVRMAQPFSLRYMLVDGQGNFGSIDGDSAAAM |
| SRR7249868 | KAYKKSARVVGDVIGKYHPHGDFAVYNTIVRMAQPFSLRYMLVDGQGNFGSIDGDSAAAM |
| SRR7278088 | KAYKKSARVVGDVIGKYHPHGDFAVYYTIVRMAQPFSLRYMLVDGQGNFGSIDGDSAAAM |
| SRR7285788 | KAYKKSARVVGDVIGKYHPHGDFAVYYTIVRMAQPFSLRYMLVDGQGNFGSIDGDSAAAM |
| SRR7286789 | KAYKKSARVVGDVIGKYHPHGDFAVYYTIVRMAQPFSLRYMLVDGQGNFGSIDGDSAAAM |
| SRR7286886 | KAYKKSARVVGDVIGKYHPHGDFAVYYTIVRMAQPFSLRYMLVDGQGNFGSIDGDSAAAM |
| SRR7310632 | KAYKKSARVVGDVIGKYHPHGDFAVYGTIVRMAQPFSLRYMLVDGQGNFGSIDGDSAAAM |
| SRR7350631 | KAYKKSARVVGDVIGKYHPHGDFAVYNTIVRMAQPFSLRYMLVDGQGNFGSIDGDSAAAM |
| SRR7458741 | KAYKKSARVVGDVIGKYHPHGDFAVYNTIVRMAQPFSLRYMLVDGQGNFGSIDGDSAAAM |
| SRR7480280 | KAYKKSARVVGDVIGKYHPHGDFAVYGTIVRMAQPFSLRYMLVDGQGNFGSIDGDSAAAM |
| SRR7523660 | KAYKKSARVVGDVIGKYHPHGDFAVYGTIVRMAQPFSLRYMLVDGQGNFGSIDGDSAAAM |
| SRR7523775 | KAYKKSARVVGDVIGKYHPHGDFAVYNTIVRMAQPFSLRYMLVDGQGNFGSIDGDSAAAM |
| SRR7251101 | KAYKKSARVVGDVIGKYHPHGDFAVYNTIVRMAQPFSLRYMLVDGQGNFGSIDGDSAAAM |
| SRR7284299 | KAYKKSARVVGDVIGKYHPHGDFAVYNTIVRMAQPFSLRYMLVDGQGNFGSIDGDSAAAM |
| SRR7285738 | KAYKKSARVVGDVIGKYHPHGDFAVYYTIVRMAQPFSLRYMLVDGQGNFGSIDGDSAAAM |
| SRR7310640 | KAYKKSARVVGDVIGKYHPHGDFAVYYTIVRMAQPFSLRYMLVDGQGNFGSIDGDSAAAM |
| SRR7349159 | KAYKKSARVVGDVIGKYHPHGDFAVYYTIVRMAQPFSLRYMLVDGQGNFGSIDGDSAAAM |
| SRR7474873 | KAYKKSARVVGDVIGKYHPHGDFAVYNTIVRMAQPFSLRYMLVDGQGNFGSIDGDSAAAM |
| SRR7495689 | KAYKKSARVVGDVIGKYHPHGDFAVYNTIVRMAQPFSLRYMLVDGQGNFGSIDGDSAAAM |
| SRR7495752 | KAYKKSARVVGDVIGKYHPHGDFAVYNTIVRMAQPFSLRYMLVDGQGNFGSIDGDSAAAM |
| ----------------------------------------------------------------------------- | |
| S16BD08730 | RYTEIRLAKIAHELMADLEKETVDFVDNYDGTEKIPDVMPTKIPNLLVNGSSGIAVGMAT |
| S18BD00684 | RYTEIRLAKIAHELMADLEKETVDFVDNYDGTEKIPDVMPTKIPNLLVNGSSGIAVGMAT |
| S18BD03994 | RYTEIRLAKIAHELMADLEKETVDFVDNYDGTEKIPDVMPTKIPNLLVNGSSGIAVGMAT |
| S18BD05011 | RYTEIRLAKIAHELMADLEKETVDFVDNYDGTEKIPDVMPTKIPNLLVNGSSGIAVGMAT |
| RKI_16-03723 | RYTEIRLAKIAHELMADLEKETVDFVDNYDGTEKIPDVMPTKIPNLLVNGSSGIAVGMAT |
| RKI_16-04315 | RYTEIRLAKIAHELMADLEKETVDFVDNYDGTEKIPDVMPTKIPNLLVNGSSGIAVGMAT |
| RKI_17-02304 | RYTEIRLAKIAHELMADLEKETVDFVDNYDGTEKIPDVMPTKIPNLLVNGSSGIAVGMAT |
| RKI_17-02411 | RYTEIRLAKIAHELMADLEKETVDFVDNYDGTEKIPDVMPTKIPNLLVNGSSGIAVGMAT |
| RKI_17-02757 | RYTEIRLAKIAHELMADLEKETVDFVDNYDGTEKIPDVMPTKIPNLLVNGSSGIAVGMAT |
| RKI_17-04797 | RYTEIRLAKIAHELMADLEKETVDFVDNYDGTEKIPDVMPTKIPNLLVNGSSGIAVGMAT |
| RKI_17-06869 | RYTEIRLAKIAHELMADLEKETVDFVDNYDGTEKIPDVMPTKIPNLLVNGSSGIAVGMAT |
| ERR2580277 | RYTEIRLAKIAHELMADLEKETVDFVDNYDGTEKIPDVMPTKIPNLLVNGSSGIAVGMAT |
| ERR2580276 | RYTEIRLAKIAHELMADLEKETVDFVDNYDGTEKIPDVMPTKIPNLLVNGSSGIAVGMAT |
| ERR2580273 | RYTEIRLAKIAHELMADLEKETVDFVDNYDGTEKIPDVMPTKIPNLLVNGSSGIAVGMAT |
| ERR2580274 | RYTEIRLAKIAHELMADLEKETVDFVDNYDGTEKIPDVMPTKIPNLLVNGSSGIAVGMAT |
| ERR2173656 | RYTEIRLAKIAHELMADLEKETVDFVDNYDGTEKIPDVMPTKIPNLLVNGSSGIAVGMAT |
| 17041676 | RYTEIRLAKIAHELMADLEKETVDFVDNYDGTEKIPDVMPTKIPNLLVNGSSGIAVGMAT |
| MT16-000061 | RYTEIRLAKIAHELMADLEKETVDFVDNYDGTEKIPDVMPTKIPNLLVNGSSGIAVGMAT |
| MT16-019416 | RYTEIRLAKIAHELMADLEKETVDFVDNYDGTEKIPDVMPTKIPNLLVNGSSGIAVGMAT |
| MT16-027865 | RYTEIRLAKIAHELMADLEKETVDFVDNYDGTEKIPDVMPTKIPNLLVNGSSGIAVGMAT |
| MT16-031693 | RYTEIRLAKIAHELMADLEKETVDFVDNYDGTEKIPDVMPTKIPNLLVNGSSGIAVGMAT |
| MT16-040253 | RYTEIRLAKIAHELMADLEKETVDFVDNYDGTEKIPDVMPTKIPNLLVNGSSGIAVGMAT |
| MT16-045379 | RYTEIRLAKIAHELMADLEKETVDFVDNYDGTEKIPDVMPTKIPNLLVNGSSGIAVGMAT |
| MT16-442728 | RYTEIRLAKIAHELMADLEKETVDFVDNYDGTEKIPDVMPTKIPNLLVNGSSGIAVGMAT |
| MT16-462857 | RYTEIRLAKIAHELMADLEKETVDFVDNYDGTEKIPDVMPTKIPNLLVNGSSGIAVGMAT |
| MT16-480196 | RYTEIRLAKIAHELMADLEKETVDFVDNYDGTEKIPDVMPTKIPNLLVNGSSGIAVGMAT |
| MT16-861555 | RYTEIRLAKIAHELMADLEKETVDFVDNYDGTEKIPDVMPTKIPNLLVNGSSGIAVGMAT |
| MT17-076833 | RYTEIRLAKIAHELMADLEKETVDFVDNYDGTEKIPDVMPTKIPNLLVNGSSGIAVGMAT |
| MT17-110677 | RYTEIRLAKIAHELMADLEKETVDFVDNYDGTEKIPDVMPTKIPNLLVNGSSGIAVGMAT |
| MT17-131730 | RYTEIRLAKIAHELMADLEKETVDFVDNYDGTEKIPDVMPTKIPNLLVNGSSGIAVGMAT |
| MT17-140890 | RYTEIRLAKIAHELMADLEKETVDFVDNYDGTEKIPDVMPTKIPNLLVNGSSGIAVGMAT |
| MT17-141840 | RYTEIRLAKIAHELMADLEKETVDFVDNYDGTEKIPDVMPTKIPNLLVNGSSGIAVGMAT |
| MT17-152488 | RYTEIRLAKIAHELMADLEKETVDFVDNYDGTEKIPDVMPTKIPNLLVNGSSGIAVGMAT |
| MT17-157311 | RYTEIRLAKIAHELMADLEKETVDFVDNYDGTEKIPDVMPTKIPNLLVNGSSGIAVGMAT |
| MT17-161645 | RYTEIRLAKIAHELMADLEKETVDFVDNYDGTEKIPDVMPTKIPNLLVNGSSGIAVGMAT |
| MT17-167951 | RYTEIRLAKIAHELMADLEKETVDFVDNYDGTEKIPDVMPTKIPNLLVNGSSGIAVGMAT |
| MT18-217732 | RYTEIRLAKIAHELMADLEKETVDFVDNYDGTEKIPDVMPTKIPNLLVNGSSGIAVGMAT |
| MT18-252580 | RYTEIRLAKIAHELMADLEKETVDFVDNYDGTEKIPDVMPTKIPNLLVNGSSGIAVGMAT |
| RIVM_H_2009-01 | RYTEIRLAKIAHELMADLEKETVDFVDNYDGTEKIPDVMPTKIPNLLVNGSSGIAVGMAT |
| RIVM_H_2010-01 | RYTEIRLAKIAHELMADLEKETVDFVDNYDGTEKIPDVMPTKIPNLLVNGSSGIAVGMAT |
| RIVM_H_2010-02 | RYTEIRLAKIAHELMADLEKETVDFVDNYDGTEKIPDVMPTKIPNLLVNGSSGIAVGMAT |
| RIVM_H_2011-01 | RYTEIRLAKIAHELMADLEKETVDFVDNYDGTEKIPDVMPTKIPNLLVNGSSGIAVGMAT |
| RIVM_H_2011-02 | RYTEIRLAKIAHELMADLEKETVDFVDNYDGTEKIPDVMPTKIPNLLVNGSSGIAVGMAT |
| RIVM_H_2011-03 | RYTEIRLAKIAHELMADLEKETVDFVDNYDGTEKIPDVMPTKIPNLLVNGSSGIAVGMAT |
| RIVM_H_2013-01 | RYTEIRLAKIAHELMADLEKETVDFVDNYDGTEKIPDVMPTKIPNLLVNGSSGIAVGMAT |
| RIVM_H_2013-02 | RYTEIRLAKIAHELMADLEKETVDFVDNYDGTEKIPDVMPTKIPNLLVNGSSGIAVGMAT |
| RIVM_H_2014-01 | RYTEIRLAKIAHELMADLEKETVDFVDNYDGTEKIPDVMPTKIPNLLVNGSSGIAVGMAT |
| RIVM_H_2014-02 | RYTEIRLAKIAHELMADLEKETVDFVDNYDGTEKIPDVMPTKIPNLLVNGSSGIAVGMAT |
| RIVM_H_2016-01 | RYTEIRLAKIAHELMADLEKETVDFVDNYDGTEKIPDVMPTKIPNLLVNGSSGIAVGMAT |
| RIVM_H_2016-02 | RYTEIRLAKIAHELMADLEKETVDFVDNYDGTEKIPDVMPTKIPNLLVNGSSGIAVGMAT |
| RIVM_H_2016-03 | RYTEIRLAKIAHELMADLEKETVDFVDNYDGTEKIPDVMPTKIPNLLVNGSSGIAVGMAT |
| RIVM_H_2016-04 | RYTEIRLAKIAHELMADLEKETVDFVDNYDGTEKIPDVMPTKIPNLLVNGSSGIAVGMAT |
| RIVM_H_2016-05 | RYTEIRLAKIAHELMADLEKETVDFVDNYDGTEKIPDVMPTKIPNLLVNGSSGIAVGMAT |
| RIVM_H_2016-06 | RYTEIRLAKIAHELMADLEKETVDFVDNYDGTEKIPDVMPTKIPNLLVNGSSGIAVGMAT |
| RIVM_H_2016-07 | RYTEIRLAKIAHELMADLEKETVDFVDNYDGTEKIPDVMPTKIPNLLVNGSSGIAVGMAT |
| RIVM_H_2016-08 | RYTEIRLAKIAHELMADLEKETVDFVDNYDGTEKIPDVMPTKIPNLLVNGSSGIAVGMAT |
| RIVM_H_2016-09 | RYTEIRLAKIAHELMADLEKETVDFVDNYDGTEKIPDVMPTKIPNLLVNGSSGIAVGMAT |
| RIVM_H_2016-10 | RYTEIRLAKIAHELMADLEKETVDFVDNYDGTEKIPDVMPTKIPNLLVNGSSGIAVGMAT |
| RIVM_H_2016-11 | RYTEIRLAKIAHELMADLEKETVDFVDNYDGTEKIPDVMPTKIPNLLVNGSSGIAVGMAT |
| RIVM_H_2016-12 | RYTEIRLAKIAHELMADLEKETVDFVDNYDGTEKIPDVMPTKIPNLLVNGSSGIAVGMAT |
| RIVM_H_2016-13 | RYTEIRLAKIAHELMADLEKETVDFVDNYDGTEKIPDVMPTKIPNLLVNGSSGIAVGMAT |
| RIVM_H_2016-14 | RYTEIRLAKIAHELMADLEKETVDFVDNYDGTEKIPDVMPTKIPNLLVNGSSGIAVGMAT |
| RIVM_H_2016-15 | RYTEIRLAKIAHELMADLEKETVDFVDNYDGTEKIPDVMPTKIPNLLVNGSSGIAVGMAT |
| RIVM_H_2017-01 | RYTEIRLAKIAHELMADLEKETVDFVDNYDGTEKIPDVMPTKIPNLLVNGSSGIAVGMAT |
| RIVM_H_2017-02 | RYTEIRLAKIAHELMADLEKETVDFVDNYDGTEKIPDVMPTKIPNLLVNGSSGIAVGMAT |
| RIVM_H_2017-03 | RYTEIRLAKIAHELMADLEKETVDFVDNYDGTEKIPDVMPTKIPNLLVNGSSGIAVGMAT |
| RIVM_H_2017-04 | RYTEIRLAKIAHELMADLEKETVDFVDNYDGTEKIPDVMPTKIPNLLVNGSSGIAVGMAT |
| RIVM_H_2017-05 | RYTEIRLAKIAHELMADLEKETVDFVDNYDGTEKIPDVMPTKIPNLLVNGSSGIAVGMAT |
| RIVM_H_2017-06 | RYTEIRLAKIAHELMADLEKETVDFVDNYDGTEKIPDVMPTKIPNLLVNGSSGIAVGMAT |
| RIVM_H_2017-07 | RYTEIRLAKIAHELMADLEKETVDFVDNYDGTEKIPDVMPTKIPNLLVNGSSGIAVGMAT |
| RIVM_H_2017-08 | RYTEIRLAKIAHELMADLEKETVDFVDNYDGTEKIPDVMPTKIPNLLVNGSSGIAVGMAT |
| RIVM_H_2017-09 | RYTEIRLAKIAHELMADLEKETVDFVDNYDGTEKIPDVMPTKIPNLLVNGSSGIAVGMAT |
| RIVM_H_2017-10 | RYTEIRLAKIAHELMADLEKETVDFVDNYDGTEKIPDVMPTKIPNLLVNGSSGIAVGMAT |
| RIVM_H_2017-11 | RYTEIRLAKIAHELMADLEKETVDFVDNYDGTEKIPDVMPTKIPNLLVNGSSGIAVGMAT |
| RIVM_H_2017-12 | RYTEIRLAKIAHELMADLEKETVDFVDNYDGTEKIPDVMPTKIPNLLVNGSSGIAVGMAT |
| RIVM_H_2017-13 | RYTEIRLAKIAHELMADLEKETVDFVDNYDGTEKIPDVMPTKIPNLLVNGSSGIAVGMAT |
| RIVM_H_2017-14 | RYTEIRLAKIAHELMADLEKETVDFVDNYDGTEKIPDVMPTKIPNLLVNGSSGIAVGMAT |
| RIVM_H_2017-15 | RYTEIRLAKIAHELMADLEKETVDFVDNYDGTEKIPDVMPTKIPNLLVNGSSGIAVGMAT |
| RIVM_H_2017-16 | RYTEIRLAKIAHELMADLEKETVDFVDNYDGTEKIPDVMPTKIPNLLVNGSSGIAVGMAT |
| RIVM_H_2017-17 | RYTEIRLAKIAHELMADLEKETVDFVDNYDGTEKIPDVMPTKIPNLLVNGSSGIAVGMAT |
| RIVM_H_2017-18 | RYTEIRLAKIAHELMADLEKETVDFVDNYDGTEKIPDVMPTKIPNLLVNGSSGIAVGMAT |
| RIVM_H_2017-19 | RYTEIRLAKIAHELMADLEKETVDFVDNYDGTEKIPDVMPTKIPNLLVNGSSGIAVGMAT |
| 15EP001483 | RYTEIRLAKIAHELMADLEKETVDFVDNYDGTEKIPDVMPTKIPNLLVNGSSGIAVGMAT |
| 17EP002363 | RYTEIRLAKIAHELMADLEKETVDFVDNYDGTEKIPDVMPTKIPNLLVNGSSGIAVGMAT |
| S_0812_17 | RYTEIRLAKIAHELMADLEKETVDFVDNYDGTEKIPDVMPTKIPNLLVNGSSGIAVGMAT |
| SRR1957844 | RYTEIRLAKIAHELMADLEKETVDFVDNYDGTEKIPDVMPTKIPNLLVNGSSGIAVGMAT |
| SRR1958654 | RYTEIRLAKIAHELMADLEKETVDFVDNYDGTEKIPDVMPTKIPNLLVNGSSGIAVGMAT |
| SRR1965077 | RYTEIRLAKIAHELMADLEKETVDFVDNYDGTEKIPDVMPTKIPNLLVNGSSGIAVGMAT |
| SRR1966369 | RYTEIRLAKIAHELMADLEKETVDFVDNYDGTEKIPDVMPTKIPNLLVNGSSGIAVGMAT |
| SRR1967117 | RYTEIRLAKIAHELMADLEKETVDFVDNYDGTEKIPDVMPTKIPNLLVNGSSGIAVGMAT |
| SRR1967922 | RYTEIRLAKIAHELMADLEKETVDFVDNYDGTEKIPDVMPTKIPNLLVNGSSGIAVGMAT |
| SRR8704720 | RYTEIRLAKIAHELMADLEKETVDFVDNYDGTEKIPDVMPTKIPNLLVNGSSGIAVGMAT |
| SRR7216071 | RYTEIRLAKIAHELMADLEKETVDFVDNYDGTEKIPDVMPTKIPNLLVNGSSGIAVGMAT |
| SRR7349175 | RYTEIRLAKIAHELMADLEKETVDFVDNYDGTEKIPDVMPTKIPNLLVNGSSGIAVGMAT |
| SRR7523148 | RYTEIRLAKIAHELMADLEKETVDFVDNYDGTEKIPDVMPTKIPNLLVNGSSGIAVGMAT |
| SRR7523854 | RYTEIRLAKIAHELMADLEKETVDFVDNYDGTEKIPDVMPTKIPNLLVNGSSGIAVGMAT |
| 313865 | RYTEIRLAKIAHELMADLEKETVDFVDNYDGTEKIPDVMPTKIPNLLVNGSSGIAVGMAT |
| SRR7277793 | RYTEIRLAKIAHELMADLEKETVDFVDNYDGTEKIPDVMPTKIPNLLVNGSSGIAVGMAT |
| SRR7343877 | RYTEIRLAKIAHELMADLEKETVDFVDNYDGTEKIPDVMPTKIPNLLVNGSSGIAVGMAT |
| SRR7351477 | RYTEIRLAKIAHELMADLEKETVDFVDNYDGTEKIPDVMPTKIPNLLVNGSSGIAVGMAT |
| SRR5583183 | RYTEIRLAKIAHELMADLEKETVDFVDNYDGTEKIPDVMPTKIPNLLVNGSSGIAVGMAT |
| SRR5585240 | RYTEIRLAKIAHELMADLEKETVDFVDNYDGTEKIPDVMPTKIPNLLVNGSSGIAVGMAT |
| SRR7284317 | RYTEIRLAKIAHELMADLEKETVDFVDNYDGTEKIPDVMPTKIPNLLVNGSSGIAVGMAT |
| SRR7299161 | RYTEIRLAKIAHELMADLEKETVDFVDNYDGTEKIPDVMPTKIPNLLVNGSSGIAVGMAT |
| SRR7401730 | RYTEIRLAKIAHELMADLEKETVDFVDNYDGTEKIPDVMPTKIPNLLVNGSSGIAVGMAT |
| SRR7469092 | RYTEIRLAKIAHELMADLEKETVDFVDNYDGTEKIPDVMPTKIPNLLVNGSSGIAVGMAT |
| SRR7879556 | RYTEIRLAKIAHELMADLEKETVDFVDNYDGTEKIPDVMPTKIPNLLVNGSSGIAVGMAT |
| SRR8526100 | RYTEIRLAKIAHELMADLEKETVDFVDNYDGTEKIPDVMPTKIPNLLVNGSSGIAVGMAT |
| SRR8553991 | RYTEIRLAKIAHELMADLEKETVDFVDNYDGTEKIPDVMPTKIPNLLVNGSSGIAVGMAT |
| SRR7842487 | RYTEIRLAKIAHELMADLEKETVDFVDNYDGTEKIPDVMPTKIPNLLVNGSSGIAVGMAT |
| SRR8054524 | RYTEIRLAKIAHELMADLEKETVDFVDNYDGTEKIPDVMPTKIPNLLVNGSSGIAVGMAT |
| SRR8054525 | RYTEIRLAKIAHELMADLEKETVDFVDNYDGTEKIPDVMPTKIPNLLVNGSSGIAVGMAT |
| SRR8524733 | RYTEIRLAKIAHELMADLEKETVDFVDNYDGTEKIPDVMPTKIPNLLVNGSSGIAVGMAT |
| SRR4093291 | RYTEIRLAKIAHELMADLEKETVDFVDNYDGTEKIPDVMPTKIPNLLVNGSSGIAVGMAT |
| SRR4245549 | RYTEIRLAKIAHELMADLEKETVDFVDNYDGTEKIPDVMPTKIPNLLVNGSSGIAVGMAT |
| SRR3057154 | RYTEIRLAKIAHELMADLEKETVDFVDNYDGTEKIPDVMPTKIPNLLVNGSSGIAVGMAT |
| SRR1726150 | RYTEIRLAKIAHELMADLEKETVDFVDNYDGTEKIPDVMPTKIPNLLVNGSSGIAVGMAT |
| SRR1996141 | RYTEIRLAKIAHELMADLEKETVDFVDNYDGTEKIPDVMPTKIPNLLVNGSSGIAVGMAT |
| SRR1107842 | RYTEIRLAKIAHELMADLEKETVDFVDNYDGTEKIPDVMPTKIPNLLVNGSSGIAVGMAT |
| SRR1157587 | RYTEIRLAKIAHELMADLEKETVDFVDNYDGTEKIPDVMPTKIPNLLVNGSSGIAVGMAT |
| SRR3027706 | RYTEIRLAKIAHELMADLEKETVDFVDNYDGTEKIPDVMPTKIPNLLVNGSSGIAVGMAT |
| SRR3027707 | RYTEIRLAKIAHELMADLEKETVDFVDNYDGTEKIPDVMPTKIPNLLVNGSSGIAVGMAT |
| SRR3027708 | RYTEIRLAKIAHELMADLEKETVDFVDNYDGTEKIPDVMPTKIPNLLVNGSSGIAVGMAT |
| SRR3027710 | RYTEIRLAKIAHELMADLEKETVDFVDNYDGTEKIPDVMPTKIPNLLVNGSSGIAVGMAT |
| SRR3027711 | RYTEIRLAKIAHELMADLEKETVDFVDNYDGTEKIPDVMPTKIPNLLVNGSSGIAVGMAT |
| SRR3027716 | RYTEIRLAKIAHELMADLEKETVDFVDNYDGTEKIPDVMPTKIPNLLVNGSSGIAVGMAT |
| SRR3027717 | RYTEIRLAKIAHELMADLEKETVDFVDNYDGTEKIPDVMPTKIPNLLVNGSSGIAVGMAT |
| SRR3027719 | RYTEIRLAKIAHELMADLEKETVDFVDNYDGTEKIPDVMPTKIPNLLVNGSSGIAVGMAT |
| SRR3027721 | RYTEIRLAKIAHELMADLEKETVDFVDNYDGTEKIPDVMPTKIPNLLVNGSSGIAVGMAT |
| SRR3027723 | RYTEIRLAKIAHELMADLEKETVDFVDNYDGTEKIPDVMPTKIPNLLVNGSSGIAVGMAT |
| SRR3115978 | RYTEIRLAKIAHELMADLEKETVDFVDNYDGTEKIPDVMPTKIPNLLVNGSSGIAVGMAT |
| SRR2534093 | RYTEIRLAKIAHELMADLEKETVDFVDNYDGTEKIPDVMPTKIPNLLVNGSSGIAVGMAT |
| SRR2534094 | RYTEIRLAKIAHELMADLEKETVDFVDNYDGTEKIPDVMPTKIPNLLVNGSSGIAVGMAT |
| SRR2534095 | RYTEIRLAKIAHELMADLEKETVDFVDNYDGTEKIPDVMPTKIPNLLVNGSSGIAVGMAT |
| SRR2534108 | RYTEIRLAKIAHELMADLEKETVDFVDNYDGTEKIPDVMPTKIPNLLVNGSSGIAVGMAT |
| SRR1106464 | RYTEIRLAKIAHELMADLEKETVDFVDNYDGTEKIPDVMPTKIPNLLVNGSSGIAVGMAT |
| SRR1106463 | RYTEIRLAKIAHELMADLEKETVDFVDNYDGTEKIPDVMPTKIPNLLVNGSSGIAVGMAT |
| SRR6949610 | RYTEIRLAKIAHELMADLEKETVDFVDNYDGTEKIPDVMPTKIPNLLVNGSSGIAVGMAT |
| SRR6950452 | RYTEIRLAKIAHELMADLEKETVDFVDNYDGTEKIPDVMPTKIPNLLVNGSSGIAVGMAT |
| ERR2019831 | RYTEIRLAKIAHELMADLEKETVDFVDNYDGTEKIPDVMPTKIPNLLVNGSSGIAVGMAT |
| SRR2085693 | RYTEIRLAKIAHELMADLEKETVDFVDNYDGTEKIPDVMPTKIPNLLVNGSSGIAVGMAT |
| SRR2086898 | RYTEIRLAKIAHELMADLEKETVDFVDNYDGTEKIPDVMPTKIPNLLVNGSSGIAVGMAT |
| SRR2175312 | RYTEIRLAKIAHELMADLEKETVDFVDNYDGTEKIPDVMPTKIPNLLVNGSSGIAVGMAT |
| SRR2175360 | RYTEIRLAKIAHELMADLEKETVDFVDNYDGTEKIPDVMPTKIPNLLVNGSSGIAVGMAT |
| SRR5231997 | RYTEIRLAKIAHELMADLEKETVDFVDNYDGTEKIPDVMPTKIPNLLVNGSSGIAVGMAT |
| SRR5232003 | RYTEIRLAKIAHELMADLEKETVDFVDNYDGTEKIPDVMPTKIPNLLVNGSSGIAVGMAT |
| SRR5232015 | RYTEIRLAKIAHELMADLEKETVDFVDNYDGTEKIPDVMPTKIPNLLVNGSSGIAVGMAT |
| SRR949434 | RYTEIRLAKIAHELMADLEKETVDFVDNYDGTEKIPDVMPTKIPNLLVNGSSGIAVGMAT |
| SRR3216575 | RYTEIRLAKIAHELMADLEKETVDFVDNYDGTEKIPDVMPTKIPNLLVNGSSGIAVGMAT |
| SRR5205342 | RYTEIRLAKIAHELMADLEKETVDFVDNYDGTEKIPDVMPTKIPNLLVNGSSGIAVGMAT |
| SRR1501669 | RYTEIRLAKIAHELMADLEKETVDFVDNYDGTEKIPDVMPTKIPNLLVNGSSGIAVGMAT |
| SRR5209740 | RYTEIRLAKIAHELMADLEKETVDFVDNYDGTEKIPDVMPTKIPNLLVNGSSGIAVGMAT |
| SRR3240355 | RYTEIRLAKIAHELMADLEKETVDFVDNYDGTEKIPDVMPTKIPNLLVNGSSGIAVGMAT |
| SRR3392777 | RYTEIRLAKIAHELMADLEKETVDFVDNYDGTEKIPDVMPTKIPNLLVNGSSGIAVGMAT |
| SRR3593671 | RYTEIRLAKIAHELMADLEKETVDFVDNYDGTEKIPDVMPTKIPNLLVNGSSGIAVGMAT |
| SRR5413290 | RYTEIRLAKIAHELMADLEKETVDFVDNYDGTEKIPDVMPTKIPNLLVNGSSGIAVGMAT |
| SRR5590269 | RYTEIRLAKIAHELMADLEKETVDFVDNYDGTEKIPDVMPTKIPNLLVNGSSGIAVGMAT |
| SRR5812103 | RYTEIRLAKIAHELMADLEKETVDFVDNYDGTEKIPDVMPTKIPNLLVNGSSGIAVGMAT |
| SRR2830941 | RYTEIRLAKIAHELMADLEKETVDFVDNYDGTEKIPDVMPTKIPNLLVNGSSGIAVGMAT |
| SRR2830966 | RYTEIRLAKIAHELMADLEKETVDFVDNYDGTEKIPDVMPTKIPNLLVNGSSGIAVGMAT |
| SRR3137270 | RYTEIRLAKIAHELMADLEKETVDFVDNYDGTEKIPDVMPTKIPNLLVNGSSGIAVGMAT |
| SRR3137271 | RYTEIRLAKIAHELMADLEKETVDFVDNYDGTEKIPDVMPTKIPNLLVNGSSGIAVGMAT |
| ERR526807 | RYTEIRLAKIAHELMADLEKETVDFVDNYDGTEKIPDVMPTKIPNLLVNGSSGIAVGMAT |
| ERR2197922 | RYTEIRLAKIAHELMADLEKETVDFVDNYDGTEKIPDVMPTKIPNLLVNGSSGIAVGMAT |
| ERR2197923 | RYTEIRLAKIAHELMADLEKETVDFVDNYDGTEKIPDVMPTKIPNLLVNGSSGIAVGMAT |
| ERR2197924 | RYTEIRLAKIAHELMADLEKETVDFVDNYDGTEKIPDVMPTKIPNLLVNGSSGIAVGMAT |
| ERR2197925 | RYTEIRLAKIAHELMADLEKETVDFVDNYDGTEKIPDVMPTKIPNLLVNGSSGIAVGMAT |
| ERR2197927 | RYTEIRLAKIAHELMADLEKETVDFVDNYDGTEKIPDVMPTKIPNLLVNGSSGIAVGMAT |
| ERR2197929 | RYTEIRLAKIAHELMADLEKETVDFVDNYDGTEKIPDVMPTKIPNLLVNGSSGIAVGMAT |
| SRR1648149 | RYTEIRLAKIAHELMADLEKETVDFVDNYDGTEKIPDVMPTKIPNLLVNGSSGIAVGMAT |
| SRR1048299 | RYTEIRLAKIAHELMADLEKETVDFVDNYDGTEKIPDVMPTKIPNLLVNGSSGIAVGMAT |
| SRR1300677 | RYTEIRLAKIAHELMADLEKETVDFVDNYDGTEKIPDVMPTKIPNLLVNGSSGIAVGMAT |
| SRR1288356 | RYTEIRLAKIAHELMADLEKETVDFVDNYDGTEKIPDVMPTKIPNLLVNGSSGIAVGMAT |
| SRR7426190 | RYTEIRLAKIAHELMADLEKETVDFVDNYDGTEKIPDVMPTKIPNLLVNGSSGIAVGMAT |
| SRR7426192 | RYTEIRLAKIAHELMADLEKETVDFVDNYDGTEKIPDVMPTKIPNLLVNGSSGIAVGMAT |
| SRR7426193 | RYTEIRLAKIAHELMADLEKETVDFVDNYDGTEKIPDVMPTKIPNLLVNGSSGIAVGMAT |
| SRR7441832 | RYTEIRLAKIAHELMADLEKETVDFVDNYDGTEKIPDVMPTKIPNLLVNGSSGIAVGMAT |
| SRR7426179 | RYTEIRLAKIAHELMADLEKETVDFVDNYDGTEKIPDVMPTKIPNLLVNGSSGIAVGMAT |
| SRR7439238 | RYTEIRLAKIAHELMADLEKETVDFVDNYDGTEKIPDVMPTKIPNLLVNGSSGIAVGMAT |
| SRR7439244 | RYTEIRLAKIAHELMADLEKETVDFVDNYDGTEKIPDVMPTKIPNLLVNGSSGIAVGMAT |
| SRR7439259 | RYTEIRLAKIAHELMADLEKETVDFVDNYDGTEKIPDVMPTKIPNLLVNGSSGIAVGMAT |
| SRR7439260 | RYTEIRLAKIAHELMADLEKETVDFVDNYDGTEKIPDVMPTKIPNLLVNGSSGIAVGMAT |
| SRR7441786 | RYTEIRLAKIAHELMADLEKETVDFVDNYDGTEKIPDVMPTKIPNLLVNGSSGIAVGMAT |
| SRR7441797 | RYTEIRLAKIAHELMADLEKETVDFVDNYDGTEKIPDVMPTKIPNLLVNGSSGIAVGMAT |
| ERR1759093 | RYTEIRLAKIAHELMADLEKETVDFVDNYDGTEKIPDVMPTKIPNLLVNGSSGIAVGMAT |
| ERR2580275 | RYTEIRLAKIAHELMADLEKETVDFVDNYDGTEKIPDVMPTKIPNLLVNGSSGIAVGMAT |
| ERR1759204 | RYTEIRLAKIAHELMADLEKETVDFVDNYDGTEKIPDVMPTKIPNLLVNGSSGIAVGMAT |
| SRR1300699 | RYTEIRLAKIAHELMADLEKETVDFVDNYDGTEKIPDVMPTKIPNLLVNGSSGIAVGMAT |
| S_0825_17 | RYTEIRLAKIAHELMADLEKETVDFVDNYDGTEKIPDVMPTKIPNLLVNGSSGIAVGMAT |
| SRR1958215 | RYTEIRLAKIAHELMADLEKETVDFVDNYDGTEKIPDVMPTKIPNLLVNGSSGIAVGMAT |
| SRR1958540 | RYTEIRLAKIAHELMADLEKETVDFVDNYDGTEKIPDVMPTKIPNLLVNGSSGIAVGMAT |
| SRR1958636 | RYTEIRLAKIAHELMADLEKETVDFVDNYDGTEKIPDVMPTKIPNLLVNGSSGIAVGMAT |
| SRR1959422 | RYTEIRLAKIAHELMADLEKETVDFVDNYDGTEKIPDVMPTKIPNLLVNGSSGIAVGMAT |
| SRR1959427 | RYTEIRLAKIAHELMADLEKETVDFVDNYDGTEKIPDVMPTKIPNLLVNGSSGIAVGMAT |
| SRR1960226 | RYTEIRLAKIAHELMADLEKETVDFVDNYDGTEKIPDVMPTKIPNLLVNGSSGIAVGMAT |
| SRR1963498 | RYTEIRLAKIAHELMADLEKETVDFVDNYDGTEKIPDVMPTKIPNLLVNGSSGIAVGMAT |
| SRR1965947 | RYTEIRLAKIAHELMADLEKETVDFVDNYDGTEKIPDVMPTKIPNLLVNGSSGIAVGMAT |
| SRR1966125 | RYTEIRLAKIAHELMADLEKETVDFVDNYDGTEKIPDVMPTKIPNLLVNGSSGIAVGMAT |
| SRR1966330 | RYTEIRLAKIAHELMADLEKETVDFVDNYDGTEKIPDVMPTKIPNLLVNGSSGIAVGMAT |
| SRR1966565 | RYTEIRLAKIAHELMADLEKETVDFVDNYDGTEKIPDVMPTKIPNLLVNGSSGIAVGMAT |
| SRR1966864 | RYTEIRLAKIAHELMADLEKETVDFVDNYDGTEKIPDVMPTKIPNLLVNGSSGIAVGMAT |
| SRR1966989 | RYTEIRLAKIAHELMADLEKETVDFVDNYDGTEKIPDVMPTKIPNLLVNGSSGIAVGMAT |
| SRR1967688 | RYTEIRLAKIAHELMADLEKETVDFVDNYDGTEKIPDVMPTKIPNLLVNGSSGIAVGMAT |
| SRR1967733 | RYTEIRLAKIAHELMADLEKETVDFVDNYDGTEKIPDVMPTKIPNLLVNGSSGIAVGMAT |
| SRR1967746 | RYTEIRLAKIAHELMADLEKETVDFVDNYDGTEKIPDVMPTKIPNLLVNGSSGIAVGMAT |
| SRR1968341 | RYTEIRLAKIAHELMADLEKETVDFVDNYDGTEKIPDVMPTKIPNLLVNGSSGIAVGMAT |
| SRR1968456 | RYTEIRLAKIAHELMADLEKETVDFVDNYDGTEKIPDVMPTKIPNLLVNGSSGIAVGMAT |
| SRR1968465 | RYTEIRLAKIAHELMADLEKETVDFVDNYDGTEKIPDVMPTKIPNLLVNGSSGIAVGMAT |
| SRR1968761 | RYTEIRLAKIAHELMADLEKETVDFVDNYDGTEKIPDVMPTKIPNLLVNGSSGIAVGMAT |
| SRR1969047 | RYTEIRLAKIAHELMADLEKETVDFVDNYDGTEKIPDVMPTKIPNLLVNGSSGIAVGMAT |
| SRR1969255 | RYTEIRLAKIAHELMADLEKETVDFVDNYDGTEKIPDVMPTKIPNLLVNGSSGIAVGMAT |
| SRR1969412 | RYTEIRLAKIAHELMADLEKETVDFVDNYDGTEKIPDVMPTKIPNLLVNGSSGIAVGMAT |
| SRR1969524 | RYTEIRLAKIAHELMADLEKETVDFVDNYDGTEKIPDVMPTKIPNLLVNGSSGIAVGMAT |
| SRR1969584 | RYTEIRLAKIAHELMADLEKETVDFVDNYDGTEKIPDVMPTKIPNLLVNGSSGIAVGMAT |
| SRR1969648 | RYTEIRLAKIAHELMADLEKETVDFVDNYDGTEKIPDVMPTKIPNLLVNGSSGIAVGMAT |
| SRR1969804 | RYTEIRLAKIAHELMADLEKETVDFVDNYDGTEKIPDVMPTKIPNLLVNGSSGIAVGMAT |
| SRR1970221 | RYTEIRLAKIAHELMADLEKETVDFVDNYDGTEKIPDVMPTKIPNLLVNGSSGIAVGMAT |
| SRR1970268 | RYTEIRLAKIAHELMADLEKETVDFVDNYDGTEKIPDVMPTKIPNLLVNGSSGIAVGMAT |
| SRR1965862 | RYTEIRLAKIAHELMADLEKETVDFVDNYDGTEKIPDVMPTKIPNLLVNGSSGIAVGMAT |
| SRR1967363 | RYTEIRLAKIAHELMADLEKETVDFVDNYDGTEKIPDVMPTKIPNLLVNGSSGIAVGMAT |
| SRR1968276 | RYTEIRLAKIAHELMADLEKETVDFVDNYDGTEKIPDVMPTKIPNLLVNGSSGIAVGMAT |
| SRR1968967 | RYTEIRLAKIAHELMADLEKETVDFVDNYDGTEKIPDVMPTKIPNLLVNGSSGIAVGMAT |
| SRR3321531 | RYTEIRLAKIAHELMADLEKETVDFVDNYDGTEKIPDVMPTKIPNLLVNGSSGIAVGMAT |
| SRR3321883 | RYTEIRLAKIAHELMADLEKETVDFVDNYDGTEKIPDVMPTKIPNLLVNGSSGIAVGMAT |
| SRR3322413 | RYTEIRLAKIAHELMADLEKETVDFVDNYDGTEKIPDVMPTKIPNLLVNGSSGIAVGMAT |
| SRR3323012 | RYTEIRLAKIAHELMADLEKETVDFVDNYDGTEKIPDVMPTKIPNLLVNGSSGIAVGMAT |
| SRR5194289 | RYTEIRLAKIAHELMADLEKETVDFVDNYDGTEKIPDVMPTKIPNLLVNGSSGIAVGMAT |
| SRR7163798 | RYTEIRLAKIAHELMADLEKETVDFVDNYDGTEKIPDVMPTKIPNLLVNGSSGIAVGMAT |
| SRR7172610 | RYTEIRLAKIAHELMADLEKETVDFVDNYDGTEKIPDVMPTKIPNLLVNGSSGIAVGMAT |
| SRR7204568 | RYTEIRLAKIAHELMADLEKETVDFVDNYDGTEKIPDVMPTKIPNLLVNGSSGIAVGMAT |
| SRR7223230 | RYTEIRLAKIAHELMADLEKETVDFVDNYDGTEKIPDVMPTKIPNLLVNGSSGIAVGMAT |
| SRR7230675 | RYTEIRLAKIAHELMADLEKETVDFVDNYDGTEKIPDVMPTKIPNLLVNGSSGIAVGMAT |
| SRR7278056 | RYTEIRLAKIAHELMADLEKETVDFVDNYDGTEKIPDVMPTKIPNLLVNGSSGIAVGMAT |
| SRR7278086 | RYTEIRLAKIAHELMADLEKETVDFVDNYDGTEKIPDVMPTKIPNLLVNGSSGIAVGMAT |
| SRR7285841 | RYTEIRLAKIAHELMADLEKETVDFVDNYDGTEKIPDVMPTKIPNLLVNGSSGIAVGMAT |
| SRR7292625 | RYTEIRLAKIAHELMADLEKETVDFVDNYDGTEKIPDVMPTKIPNLLVNGSSGIAVGMAT |
| SRR7292665 | RYTEIRLAKIAHELMADLEKETVDFVDNYDGTEKIPDVMPTKIPNLLVNGSSGIAVGMAT |
| SRR7297965 | RYTEIRLAKIAHELMADLEKETVDFVDNYDGTEKIPDVMPTKIPNLLVNGSSGIAVGMAT |
| SRR7350726 | RYTEIRLAKIAHELMADLEKETVDFVDNYDGTEKIPDVMPTKIPNLLVNGSSGIAVGMAT |
| SRR7410328 | RYTEIRLAKIAHELMADLEKETVDFVDNYDGTEKIPDVMPTKIPNLLVNGSSGIAVGMAT |
| SRR7474665 | RYTEIRLAKIAHELMADLEKETVDFVDNYDGTEKIPDVMPTKIPNLLVNGSSGIAVGMAT |
| SRR7523184 | RYTEIRLAKIAHELMADLEKETVDFVDNYDGTEKIPDVMPTKIPNLLVNGSSGIAVGMAT |
| SRR7187264 | RYTEIRLAKIAHELMADLEKETVDFVDNYDGTEKIPDVMPTKIPNLLVNGSSGIAVGMAT |
| SRR7204445 | RYTEIRLAKIAHELMADLEKETVDFVDNYDGTEKIPDVMPTKIPNLLVNGSSGIAVGMAT |
| SRR7285641 | RYTEIRLAKIAHELMADLEKETVDFVDNYDGTEKIPDVMPTKIPNLLVNGSSGIAVGMAT |
| SRR7286695 | RYTEIRLAKIAHELMADLEKETVDFVDNYDGTEKIPDVMPTKIPNLLVNGSSGIAVGMAT |
| SRR7286705 | RYTEIRLAKIAHELMADLEKETVDFVDNYDGTEKIPDVMPTKIPNLLVNGSSGIAVGMAT |
| SRR7292931 | RYTEIRLAKIAHELMADLEKETVDFVDNYDGTEKIPDVMPTKIPNLLVNGSSGIAVGMAT |
| SRR7310349 | RYTEIRLAKIAHELMADLEKETVDFVDNYDGTEKIPDVMPTKIPNLLVNGSSGIAVGMAT |
| SRR7351616 | RYTEIRLAKIAHELMADLEKETVDFVDNYDGTEKIPDVMPTKIPNLLVNGSSGIAVGMAT |
| SRR7414818 | RYTEIRLAKIAHELMADLEKETVDFVDNYDGTEKIPDVMPTKIPNLLVNGSSGIAVGMAT |
| SRR7426480 | RYTEIRLAKIAHELMADLEKETVDFVDNYDGTEKIPDVMPTKIPNLLVNGSSGIAVGMAT |
| SRR5584105 | RYTEIRLAKIAHELMADLEKETVDFVDNYDGTEKIPDVMPTKIPNLLVNGSSGIAVGMAT |
| SRR5584565 | RYTEIRLAKIAHELMADLEKETVDFVDNYDGTEKIPDVMPTKIPNLLVNGSSGIAVGMAT |
| SRR5584614 | RYTEIRLAKIAHELMADLEKETVDFVDNYDGTEKIPDVMPTKIPNLLVNGSSGIAVGMAT |
| SRR5631543 | RYTEIRLAKIAHELMADLEKETVDFVDNYDGTEKIPDVMPTKIPNLLVNGSSGIAVGMAT |
| SRR5631553 | RYTEIRLAKIAHELMADLEKETVDFVDNYDGTEKIPDVMPTKIPNLLVNGSSGIAVGMAT |
| SRR7123196 | RYTEIRLAKIAHELMADLEKETVDFVDNYDGTEKIPDVMPTKIPNLLVNGSSGIAVGMAT |
| SRR7163819 | RYTEIRLAKIAHELMADLEKETVDFVDNYDGTEKIPDVMPTKIPNLLVNGSSGIAVGMAT |
| SRR7163920 | RYTEIRLAKIAHELMADLEKETVDFVDNYDGTEKIPDVMPTKIPNLLVNGSSGIAVGMAT |
| SRR7209528 | RYTEIRLAKIAHELMADLEKETVDFVDNYDGTEKIPDVMPTKIPNLLVNGSSGIAVGMAT |
| SRR7249868 | RYTEIRLAKIAHELMADLEKETVDFVDNYDGTEKIPDVMPTKIPNLLVNGSSGIAVGMAT |
| SRR7278088 | RYTEIRLAKIAHELMADLEKETVDFVDNYDGTEKIPDVMPTKIPNLLVNGSSGIAVGMAT |
| SRR7285788 | RYTEIRLAKIAHELMADLEKETVDFVDNYDGTEKIPDVMPTKIPNLLVNGSSGIAVGMAT |
| SRR7286789 | RYTEIRLAKIAHELMADLEKETVDFVDNYDGTEKIPDVMPTKIPNLLVNGSSGIAVGMAT |
| SRR7286886 | RYTEIRLAKIAHELMADLEKETVDFVDNYDGTEKIPDVMPTKIPNLLVNGSSGIAVGMAT |
| SRR7310632 | RYTEIRLAKIAHELMADLEKETVDFVDNYDGTEKIPDVMPTKIPNLLVNGSSGIAVGMAT |
| SRR7350631 | RYTEIRLAKIAHELMADLEKETVDFVDNYDGTEKIPDVMPTKIPNLLVNGSSGIAVGMAT |
| SRR7458741 | RYTEIRLAKIAHELMADLEKETVDFVDNYDGTEKIPDVMPTKIPNLLVNGSSGIAVGMAT |
| SRR7480280 | RYTEIRLAKIAHELMADLEKETVDFVDNYDGTEKIPDVMPTKIPNLLVNGSSGIAVGMAT |
| SRR7523660 | RYTEIRLAKIAHELMADLEKETVDFVDNYDGTEKIPDVMPTKIPNLLVNGSSGIAVGMAT |
| SRR7523775 | RYTEIRLAKIAHELMADLEKETVDFVDNYDGTEKIPDVMPTKIPNLLVNGSSGIAVGMAT |
| SRR7251101 | RYTEIRLAKIAHELMADLEKETVDFVDNYDGTEKIPDVMPTKIPNLLVNGSSGIAVGMAT |
| SRR7284299 | RYTEIRLAKIAHELMADLEKETVDFVDNYDGTEKIPDVMPTKIPNLLVNGSSGIAVGMAT |
| SRR7285738 | RYTEIRLAKIAHELMADLEKETVDFVDNYDGTEKIPDVMPTKIPNLLVNGSSGIAVGMAT |
| SRR7310640 | RYTEIRLAKIAHELMADLEKETVDFVDNYDGTEKIPDVMPTKIPNLLVNGSSGIAVGMAT |
| SRR7349159 | RYTEIRLAKIAHELMADLEKETVDFVDNYDGTEKIPDVMPTKIPNLLVNGSSGIAVGMAT |
| SRR7474873 | RYTEIRLAKIAHELMADLEKETVDFVDNYDGTEKIPDVMPTKIPNLLVNGSSGIAVGMAT |
| SRR7495689 | RYTEIRLAKIAHELMADLEKETVDFVDNYDGTEKIPDVMPTKIPNLLVNGSSGIAVGMAT |
| SRR7495752 | RYTEIRLAKIAHELMADLEKETVDFVDNYDGTEKIPDVMPTKIPNLLVNGSSGIAVGMAT |
| ----------------------------------------------------------------------------- | |
| S16BD08730 | NIPPHNLTEVINGCLAYIDNEDISIEGLMEHIPGPDFPTAAIINGRRGIEEAYRTGRGKV |
| S18BD00684 | NIPPHNLTEVINGCLAYIDNEDISIEGLMEHIPGPDFPTAAIINGRRGIEEAYRTGRGKV |
| S18BD03994 | NIPPHNLTEVINGCLAYIDNEDISIEGLMEHIPGPDFPTAAIINGRRGIEEAYRTGRGKV |
| S18BD05011 | NIPPHNLTEVINGCLAYIDNEDISIEGLMEHIPGPDFPTAAIINGRRGIEEAYRTGRGKV |
| RKI_16-03723 | NIPPHNLTEVINGCLAYIDNEDISIEGLMEHIPGPDFPTAAIINGRRGIEEAYRTGRGKV |
| RKI_16-04315 | NIPPHNLTEVINGCLAYIDNEDISIEGLMEHIPGPDFPTAAIINGRRGIEEAYRTGRGKV |
| RKI_17-02304 | NIPPHNLTEVINGCLAYIDNEDISIEGLMEHIPGPDFPTAAIINGRRGIEEAYRTGRGKV |
| RKI_17-02411 | NIPPHNLTEVINGCLAYIDNEDISIEGLMEHIPGPDFPTAAIINGRRGIEEAYRTGRGKV |
| RKI_17-02757 | NIPPHNLTEVINGCLAYIDNEDISIEGLMEHIPGPDFPTAAIINGRRGIEEAYRTGRGKV |
| RKI_17-04797 | NIPPHNLTEVINGCLAYIDNEDISIEGLMEHIPGPDFPTAAIINGRRGIEEAYRTGRGKV |
| RKI_17-06869 | NIPPHNLTEVINGCLAYIDNEDISIEGLMEHIPGPDFPTAAIINGRRGIEEAYRTGRGKV |
| ERR2580277 | NIPPHNLTEVINGCLAYIDNEDISIEGLMEHIPGPDFPTAAIINGRRGIEEAYRTGRGKV |
| ERR2580276 | NIPPHNLTEVINGCLAYIDNEDISIEGLMEHIPGPDFPTAAIINGRRGIEEAYRTGRGKV |
| ERR2580273 | NIPPHNLTEVINGCLAYIDNEDISIEGLMEHIPGPDFPTAAIINGRRGIEEAYRTGRGKV |
| ERR2580274 | NIPPHNLTEVINGCLAYIDNEDISIEGLMEHIPGPDFPTAAIINGRRGIEEAYRTGRGKV |
| ERR2173656 | NIPPHNLTEVINGCLAYIDNEDISIEGLMEHIPGPDFPTAAIINGRRGIEEAYRTGRGKV |
| 17041676 | NIPPHNLTEVINGCLAYIDNEDISIEGLMEHIPGPDFPTAAIINGRRGIEEAYRTGRGKV |
| MT16-000061 | NIPPHNLTEVINGCLAYIDNEDISIEGLMEHIPGPDFPTAAIINGRRGIEEAYRTGRGKV |
| MT16-019416 | NIPPHNLTEVINGCLAYIDNEDISIEGLMEHIPGPDFPTAAIINGRRGIEEAYRTGRGKV |
| MT16-027865 | NIPPHNLTEVINGCLAYIDNEDISIEGLMEHIPGPDFPTAAIINGRRGIEEAYRTGRGKV |
| MT16-031693 | NIPPHNLTEVINGCLAYIDNEDISIEGLMEHIPGPDFPTAAIINGRRGIEEAYRTGRGKV |
| MT16-040253 | NIPPHNLTEVINGCLAYIDNEDISIEGLMEHIPGPDFPTAAIINGRRGIEEAYRTGRGKV |
| MT16-045379 | NIPPHNLTEVINGCLAYIDNEDISIEGLMEHIPGPDFPTAAIINGRRGIEEAYRTGRGKV |
| MT16-442728 | NIPPHNLTEVINGCLAYIDNEDISIEGLMEHIPGPDFPTAAIINGRRGIEEAYRTGRGKV |
| MT16-462857 | NIPPHNLTEVINGCLAYIDNEDISIEGLMEHIPGPDFPTAAIINGRRGIEEAYRTGRGKV |
| MT16-480196 | NIPPHNLTEVINGCLAYIDNEDISIEGLMEHIPGPDFPTAAIINGRRGIEEAYRTGRGKV |
| MT16-861555 | NIPPHNLTEVINGCLAYIDNEDISIEGLMEHIPGPDFPTAAIINGRRGIEEAYRTGRGKV |
| MT17-076833 | NIPPHNLTEVINGCLAYIDNEDISIEGLMEHIPGPDFPTAAIINGRRGIEEAYRTGRGKV |
| MT17-110677 | NIPPHNLTEVINGCLAYIDNEDISIEGLMEHIPGPDFPTAAIINGRRGIEEAYRTGRGKV |
| MT17-131730 | NIPPHNLTEVINGCLAYIDNEDISIEGLMEHIPGPDFPTAAIINGRRGIEEAYRTGRGKV |
| MT17-140890 | NIPPHNLTEVINGCLAYIDNEDISIEGLMEHIPGPDFPTAAIINGRRGIEEAYRTGRGKV |
| MT17-141840 | NIPPHNLTEVINGCLAYIDNEDISIEGLMEHIPGPDFPTAAIINGRRGIEEAYRTGRGKV |
| MT17-152488 | NIPPHNLTEVINGCLAYIDNEDISIEGLMEHIPGPDFPTAAIINGRRGIEEAYRTGRGKV |
| MT17-157311 | NIPPHNLTEVINGCLAYIDNEDISIEGLMEHIPGPDFPTAAIINGRRGIEEAYRTGRGKV |
| MT17-161645 | NIPPHNLTEVINGCLAYIDNEDISIEGLMEHIPGPDFPTAAIINGRRGIEEAYRTGRGKV |
| MT17-167951 | NIPPHNLTEVINGCLAYIDNEDISIEGLMEHIPGPDFPTAAIINGRRGIEEAYRTGRGKV |
| MT18-217732 | NIPPHNLTEVINGCLAYIDNEDISIEGLMEHIPGPDFPTAAIINGRRGIEEAYRTGRGKV |
| MT18-252580 | NIPPHNLTEVINGCLAYIDNEDISIEGLMEHIPGPDFPTAAIINGRRGIEEAYRTGRGKV |
| RIVM_H_2009-01 | NIPPHNLTEVINGCLAYIDNEDISIEGLMEHIPGPDFPTAAIINGRRGIEEAYRTGRGKV |
| RIVM_H_2010-01 | NIPPHNLTEVINGCLAYIDNEDISIEGLMEHIPGPDFPTAAIINGRRGIEEAYRTGRGKV |
| RIVM_H_2010-02 | NIPPHNLTEVINGCLAYIDNEDISIEGLMEHIPGPDFPTAAIINGRRGIEEAYRTGRGKV |
| RIVM_H_2011-01 | NIPPHNLTEVINGCLAYIDNEDISIEGLMEHIPGPDFPTAAIINGRRGIEEAYRTGRGKV |
| RIVM_H_2011-02 | NIPPHNLTEVINGCLAYIDNEDISIEGLMEHIPGPDFPTAAIINGRRGIEEAYRTGRGKV |
| RIVM_H_2011-03 | NIPPHNLTEVINGCLAYIDNEDISIEGLMEHIPGPDFPTAAIINGRRGIEEAYRTGRGKV |
| RIVM_H_2013-01 | NIPPHNLTEVINGCLAYIDNEDISIEGLMEHIPGPDFPTAAIINGRRGIEEAYRTGRGKV |
| RIVM_H_2013-02 | NIPPHNLTEVINGCLAYIDNEDISIEGLMEHIPGPDFPTAAIINGRRGIEEAYRTGRGKV |
| RIVM_H_2014-01 | NIPPHNLTEVINGCLAYIDNEDISIEGLMEHIPGPDFPTAAIINGRRGIEEAYRTGRGKV |
| RIVM_H_2014-02 | NIPPHNLTEVINGCLAYIDNEDISIEGLMEHIPGPDFPTAAIINGRRGIEEAYRTGRGKV |
| RIVM_H_2016-01 | NIPPHNLTEVINGCLAYIDNEDISIEGLMEHIPGPDFPTAAIINGRRGIEEAYRTGRGKV |
| RIVM_H_2016-02 | NIPPHNLTEVINGCLAYIDNEDISIEGLMEHIPGPDFPTAAIINGRRGIEEAYRTGRGKV |
| RIVM_H_2016-03 | NIPPHNLTEVINGCLAYIDNEDISIEGLMEHIPGPDFPTAAIINGRRGIEEAYRTGRGKV |
| RIVM_H_2016-04 | NIPPHNLTEVINGCLAYIDNEDISIEGLMEHIPGPDFPTAAIINGRRGIEEAYRTGRGKV |
| RIVM_H_2016-05 | NIPPHNLTEVINGCLAYIDNEDISIEGLMEHIPGPDFPTAAIINGRRGIEEAYRTGRGKV |
| RIVM_H_2016-06 | NIPPHNLTEVINGCLAYIDNEDISIEGLMEHIPGPDFPTAAIINGRRGIEEAYRTGRGKV |
| RIVM_H_2016-07 | NIPPHNLTEVINGCLAYIDNEDISIEGLMEHIPGPDFPTAAIINGRRGIEEAYRTGRGKV |
| RIVM_H_2016-08 | NIPPHNLTEVINGCLAYIDNEDISIEGLMEHIPGPDFPTAAIINGRRGIEEAYRTGRGKV |
| RIVM_H_2016-09 | NIPPHNLTEVINGCLAYIDNEDISIEGLMEHIPGPDFPTAAIINGRRGIEEAYRTGRGKV |
| RIVM_H_2016-10 | NIPPHNLTEVINGCLAYIDNEDISIEGLMEHIPGPDFPTAAIINGRRGIEEAYRTGRGKV |
| RIVM_H_2016-11 | NIPPHNLTEVINGCLAYIDNEDISIEGLMEHIPGPDFPTAAIINGRRGIEEAYRTGRGKV |
| RIVM_H_2016-12 | NIPPHNLTEVINGCLAYIDNEDISIEGLMEHIPGPDFPTAAIINGRRGIEEAYRTGRGKV |
| RIVM_H_2016-13 | NIPPHNLTEVINGCLAYIDNEDISIEGLMEHIPGPDFPTAAIINGRRGIEEAYRTGRGKV |
| RIVM_H_2016-14 | NIPPHNLTEVINGCLAYIDNEDISIEGLMEHIPGPDFPTAAIINGRRGIEEAYRTGRGKV |
| RIVM_H_2016-15 | NIPPHNLTEVINGCLAYIDNEDISIEGLMEHIPGPDFPTAAIINGRRGIEEAYRTGRGKV |
| RIVM_H_2017-01 | NIPPHNLTEVINGCLAYIDNEDISIEGLMEHIPGPDFPTAAIINGRRGIEEAYRTGRGKV |
| RIVM_H_2017-02 | NIPPHNLTEVINGCLAYIDNEDISIEGLMEHIPGPDFPTAAIINGRRGIEEAYRTGRGKV |
| RIVM_H_2017-03 | NIPPHNLTEVINGCLAYIDNEDISIEGLMEHIPGPDFPTAAIINGRRGIEEAYRTGRGKV |
| RIVM_H_2017-04 | NIPPHNLTEVINGCLAYIDNEDISIEGLMEHIPGPDFPTAAIINGRRGIEEAYRTGRGKV |
| RIVM_H_2017-05 | NIPPHNLTEVINGCLAYIDNEDISIEGLMEHIPGPDFPTAAIINGRRGIEEAYRTGRGKV |
| RIVM_H_2017-06 | NIPPHNLTEVINGCLAYIDNEDISIEGLMEHIPGPDFPTAAIINGRRGIEEAYRTGRGKV |
| RIVM_H_2017-07 | NIPPHNLTEVINGCLAYIDNEDISIEGLMEHIPGPDFPTAAIINGRRGIEEAYRTGRGKV |
| RIVM_H_2017-08 | NIPPHNLTEVINGCLAYIDNEDISIEGLMEHIPGPDFPTAAIINGRRGIEEAYRTGRGKV |
| RIVM_H_2017-09 | NIPPHNLTEVINGCLAYIDNEDISIEGLMEHIPGPDFPTAAIINGRRGIEEAYRTGRGKV |
| RIVM_H_2017-10 | NIPPHNLTEVINGCLAYIDNEDISIEGLMEHIPGPDFPTAAIINGRRGIEEAYRTGRGKV |
| RIVM_H_2017-11 | NIPPHNLTEVINGCLAYIDNEDISIEGLMEHIPGPDFPTAAIINGRRGIEEAYRTGRGKV |
| RIVM_H_2017-12 | NIPPHNLTEVINGCLAYIDNEDISIEGLMEHIPGPDFPTAAIINGRRGIEEAYRTGRGKV |
| RIVM_H_2017-13 | NIPPHNLTEVINGCLAYIDNEDISIEGLMEHIPGPDFPTAAIINGRRGIEEAYRTGRGKV |
| RIVM_H_2017-14 | NIPPHNLTEVINGCLAYIDNEDISIEGLMEHIPGPDFPTAAIINGRRGIEEAYRTGRGKV |
| RIVM_H_2017-15 | NIPPHNLTEVINGCLAYIDNEDISIEGLMEHIPGPDFPTAAIINGRRGIEEAYRTGRGKV |
| RIVM_H_2017-16 | NIPPHNLTEVINGCLAYIDNEDISIEGLMEHIPGPDFPTAAIINGRRGIEEAYRTGRGKV |
| RIVM_H_2017-17 | NIPPHNLTEVINGCLAYIDNEDISIEGLMEHIPGPDFPTAAIINGRRGIEEAYRTGRGKV |
| RIVM_H_2017-18 | NIPPHNLTEVINGCLAYIDNEDISIEGLMEHIPGPDFPTAAIINGRRGIEEAYRTGRGKV |
| RIVM_H_2017-19 | NIPPHNLTEVINGCLAYIDNEDISIEGLMEHIPGPDFPTAAIINGRRGIEEAYRTGRGKV |
| 15EP001483 | NIPPHNLTEVINGCLAYIDNEDISIEGLMEHIPGPDFPTAAIINGRRGIEEAYRTGRGKV |
| 17EP002363 | NIPPHNLTEVINGCLAYIDNEDISIEGLMEHIPGPDFPTAAIINGRRGIEEAYRTGRGKV |
| S_0812_17 | NIPPHNLTEVINGCLAYIDNEDISIEGLMEHIPGPDFPTAAIINGRRGIEEAYRTGRGKV |
| SRR1957844 | NIPPHNLTEVINGCLAYIDNEDISIEGLMEHIPGPDFPTAAIINGRRGIEEAYRTGRGKV |
| SRR1958654 | NIPPHNLTEVINGCLAYIDNEDISIEGLMEHIPGPDFPTAAIINGRRGIEEAYRTGRGKV |
| SRR1965077 | NIPPHNLTEVINGCLAYIDNEDISIEGLMEHIPGPDFPTAAIINGRRGIEEAYRTGRGKV |
| SRR1966369 | NIPPHNLTEVINGCLAYIDNEDISIEGLMEHIPGPDFPTAAIINGRRGIEEAYRTGRGKV |
| SRR1967117 | NIPPHNLTEVINGCLAYIDNEDISIEGLMEHIPGPDFPTAAIINGRRGIEEAYRTGRGKV |
| SRR1967922 | NIPPHNLTEVINGCLAYIDNEDISIEGLMEHIPGPDFPTAAIINGRRGIEEAYRTGRGKV |
| SRR8704720 | NIPPHNLTEVINGCLAYIDNEDISIEGLMEHIPGPDFPTAAIINGRRGIEEAYRTGRGKV |
| SRR7216071 | NIPPHNLTEVINGCLAYIDNEDISIEGLMEHIPGPDFPTAAIINGRRGIEEAYRTGRGKV |
| SRR7349175 | NIPPHNLTEVINGCLAYIDNEDISIEGLMEHIPGPDFPTAAIINGRRGIEEAYRTGRGKV |
| SRR7523148 | NIPPHNLTEVINGCLAYIDNEDISIEGLMEHIPGPDFPTAAIINGRRGIEEAYRTGRGKV |
| SRR7523854 | NIPPHNLTEVINGCLAYIDNEDISIEGLMEHIPGPDFPTAAIINGRRGIEEAYRTGRGKV |
| 313865 | NIPPHNLTEVINGCLAYIDNEDISIEGLMEHIPGPDFPTAAIINGRRGIEEAYRTGRGKV |
| SRR7277793 | NIPPHNLTEVINGCLAYIDNEDISIEGLMEHIPGPDFPTAAIINGRRGIEEAYRTGRGKV |
| SRR7343877 | NIPPHNLTEVINGCLAYIDNEDISIEGLMEHIPGPDFPTAAIINGRRGIEEAYRTGRGKV |
| SRR7351477 | NIPPHNLTEVINGCLAYIDNEDISIEGLMEHIPGPDFPTAAIINGRRGIEEAYRTGRGKV |
| SRR5583183 | NIPPHNLTEVINGCLAYIDNEDISIEGLMEHIPGPDFPTAAIINGRRGIEEAYRTGRGKV |
| SRR5585240 | NIPPHNLTEVINGCLAYIDNEDISIEGLMEHIPGPDFPTAAIINGRRGIEEAYRTGRGKV |
| SRR7284317 | NIPPHNLTEVINGCLAYIDNEDISIEGLMEHIPGPDFPTAAIINGRRGIEEAYRTGRGKV |
| SRR7299161 | NIPPHNLTEVINGCLAYIDNEDISIEGLMEHIPGPDFPTAAIINGRRGIEEAYRTGRGKV |
| SRR7401730 | NIPPHNLTEVINGCLAYIDNEDISIEGLMEHIPGPDFPTAAIINGRRGIEEAYRTGRGKV |
| SRR7469092 | NIPPHNLTEVINGCLAYIDNEDISIEGLMEHIPGPDFPTAAIINGRRGIEEAYRTGRGKV |
| SRR7879556 | NIPPHNLTEVINGCLAYIDNEDISIEGLMEHIPGPDFPTAAIINGRRGIEEAYRTGRGKV |
| SRR8526100 | NIPPHNLTEVINGCLAYIDNEDISIEGLMEHIPGPDFPTAAIINGRRGIEEAYRTGRGKV |
| SRR8553991 | NIPPHNLTEVINGCLAYIDNEDISIEGLMEHIPGPDFPTAAIINGRRGIEEAYRTGRGKV |
| SRR7842487 | NIPPHNLTEVINGCLAYIDNEDISIEGLMEHIPGPDFPTAAIINGRRGIEEAYRTGRGKV |
| SRR8054524 | NIPPHNLTEVINGCLAYIDNEDISIEGLMEHIPGPDFPTAAIINGRRGIEEAYRTGRGKV |
| SRR8054525 | NIPPHNLTEVINGCLAYIDNEDISIEGLMEHIPGPDFPTAAIINGRRGIEEAYRTGRGKV |
| SRR8524733 | NIPPHNLTEVINGCLAYIDNEDISIEGLMEHIPGPDFPTAAIINGRRGIEEAYRTGRGKV |
| SRR4093291 | NIPPHNLTEVINGCLAYIDNEDISIEGLMEHIPGPDFPTAAIINGRRGIEEAYRTGRGKV |
| SRR4245549 | NIPPHNLTEVINGCLAYIDNEDISIEGLMEHIPGPDFPTAAIINGRRGIEEAYRTGRGKV |
| SRR3057154 | NIPPHNLTEVINGCLAYIDNEDISIEGLMEHIPGPDFPTAAIINGRRGIEEAYRTGRGKV |
| SRR1726150 | NIPPHNLTEVINGCLAYIDNEDISIEGLMEHIPGPDFPTAAIINGRRGIEEAYRTGRGKV |
| SRR1996141 | NIPPHNLTEVINGCLAYIDNEDISIEGLMEHIPGPDFPTAAIINGRRGIEEAYRTGRGKV |
| SRR1107842 | NIPPHNLTEVINGCLAYIDNEDISIEGLMEHIPGPDFPTAAIINGRRGIEEAYRTGRGKV |
| SRR1157587 | NIPPHNLTEVINGCLAYIDNEDISIEGLMEHIPGPDFPTAAIINGRRGIEEAYRTGRGKV |
| SRR3027706 | NIPPHNLTEVINGCLAYIDNEDISIEGLMEHIPGPDFPTAAIINGRRGIEEAYRTGRGKV |
| SRR3027707 | NIPPHNLTEVINGCLAYIDNEDISIEGLMEHIPGPDFPTAAIINGRRGIEEAYRTGRGKV |
| SRR3027708 | NIPPHNLTEVINGCLAYIDNEDISIEGLMEHIPGPDFPTAAIINGRRGIEEAYRTGRGKV |
| SRR3027710 | NIPPHNLTEVINGCLAYIDNEDISIEGLMEHIPGPDFPTAAIINGRRGIEEAYRTGRGKV |
| SRR3027711 | NIPPHNLTEVINGCLAYIDNEDISIEGLMEHIPGPDFPTAAIINGRRGIEEAYRTGRGKV |
| SRR3027716 | NIPPHNLTEVINGCLAYIDNEDISIEGLMEHIPGPDFPTAAIINGRRGIEEAYRTGRGKV |
| SRR3027717 | NIPPHNLTEVINGCLAYIDNEDISIEGLMEHIPGPDFPTAAIINGRRGIEEAYRTGRGKV |
| SRR3027719 | NIPPHNLTEVINGCLAYIDNEDISIEGLMEHIPGPDFPTAAIINGRRGIEEAYRTGRGKV |
| SRR3027721 | NIPPHNLTEVINGCLAYIDNEDISIEGLMEHIPGPDFPTAAIINGRRGIEEAYRTGRGKV |
| SRR3027723 | NIPPHNLTEVINGCLAYIDNEDISIEGLMEHIPGPDFPTAAIINGRRGIEEAYRTGRGKV |
| SRR3115978 | NIPPHNLTEVINGCLAYIDNEDISIEGLMEHIPGPDFPTAAIINGRRGIEEAYRTGRGKV |
| SRR2534093 | NIPPHNLTEVINGCLAYIDNEDISIEGLMEHIPGPDFPTAAIINGRRGIEEAYRTGRGKV |
| SRR2534094 | NIPPHNLTEVINGCLAYIDNEDISIEGLMEHIPGPDFPTAAIINGRRGIEEAYRTGRGKV |
| SRR2534095 | NIPPHNLTEVINGCLAYIDNEDISIEGLMEHIPGPDFPTAAIINGRRGIEEAYRTGRGKV |
| SRR2534108 | NIPPHNLTEVINGCLAYIDNEDISIEGLMEHIPGPDFPTAAIINGRRGIEEAYRTGRGKV |
| SRR1106464 | NIPPHNLTEVINGCLAYIDNEDISIEGLMEHIPGPDFPTAAIINGRRGIEEAYRTGRGKV |
| SRR1106463 | NIPPHNLTEVINGCLAYIDNEDISIEGLMEHIPGPDFPTAAIINGRRGIEEAYRTGRGKV |
| SRR6949610 | NIPPHNLTEVINGCLAYIDNEDISIEGLMEHIPGPDFPTAAIINGRRGIEEAYRTGRGKV |
| SRR6950452 | NIPPHNLTEVINGCLAYIDNEDISIEGLMEHIPGPDFPTAAIINGRRGIEEAYRTGRGKV |
| ERR2019831 | NIPPHNLTEVINGCLAYIDNEDISIEGLMEHIPGPDFPTAAIINGRRGIEEAYRTGRGKV |
| SRR2085693 | NIPPHNLTEVINGCLAYIDNEDISIEGLMEHIPGPDFPTAAIINGRRGIEEAYRTGRGKV |
| SRR2086898 | NIPPHNLTEVINGCLAYIDNEDISIEGLMEHIPGPDFPTAAIINGRRGIEEAYRTGRGKV |
| SRR2175312 | NIPPHNLTEVINGCLAYIDNEDISIEGLMEHIPGPDFPTAAIINGRRGIEEAYRTGRGKV |
| SRR2175360 | NIPPHNLTEVINGCLAYIDNEDISIEGLMEHIPGPDFPTAAIINGRRGIEEAYRTGRGKV |
| SRR5231997 | NIPPHNLTEVINGCLAYIDNEDISIEGLMEHIPGPDFPTAAIINGRRGIEEAYRTGRGKV |
| SRR5232003 | NIPPHNLTEVINGCLAYIDNEDISIEGLMEHIPGPDFPTAAIINGRRGIEEAYRTGRGKV |
| SRR5232015 | NIPPHNLTEVINGCLAYIDNEDISIEGLMEHIPGPDFPTAAIINGRRGIEEAYRTGRGKV |
| SRR949434 | NIPPHNLTEVINGCLAYIDNEDISIEGLMEHIPGPDFPTAAIINGRRGIEEAYRTGRGKV |
| SRR3216575 | NIPPHNLTEVINGCLAYIDNEDISIEGLMEHIPGPDFPTAAIINGRRGIEEAYRTGRGKV |
| SRR5205342 | NIPPHNLTEVINGCLAYIDNEDISIEGLMEHIPGPDFPTAAIINGRRGIEEAYRTGRGKV |
| SRR1501669 | NIPPHNLTEVINGCLAYIDNEDISIEGLMEHIPGPDFPTAAIINGRRGIEEAYRTGRGKV |
| SRR5209740 | NIPPHNLTEVINGCLAYIDNEDISIEGLMEHIPGPDFPTAAIINGRRGIEEAYRTGRGKV |
| SRR3240355 | NIPPHNLTEVINGCLAYIDNEDISIEGLMEHIPGPDFPTAAIINGRRGIEEAYRTGRGKV |
| SRR3392777 | NIPPHNLTEVINGCLAYIDNEDISIEGLMEHIPGPDFPTAAIINGRRGIEEAYRTGRGKV |
| SRR3593671 | NIPPHNLTEVINGCLAYIDNEDISIEGLMEHIPGPDFPTAAIINGRRGIEEAYRTGRGKV |
| SRR5413290 | NIPPHNLTEVINGCLAYIDNEDISIEGLMEHIPGPDFPTAAIINGRRGIEEAYRTGRGKV |
| SRR5590269 | NIPPHNLTEVINGCLAYIDNEDISIEGLMEHIPGPDFPTAAIINGRRGIEEAYRTGRGKV |
| SRR5812103 | NIPPHNLTEVINGCLAYIDNEDISIEGLMEHIPGPDFPTAAIINGRRGIEEAYRTGRGKV |
| SRR2830941 | NIPPHNLTEVINGCLAYIDNEDISIEGLMEHIPGPDFPTAAIINGRRGIEEAYRTGRGKV |
| SRR2830966 | NIPPHNLTEVINGCLAYIDNEDISIEGLMEHIPGPDFPTAAIINGRRGIEEAYRTGRGKV |
| SRR3137270 | NIPPHNLTEVINGCLAYIDNEDISIEGLMEHIPGPDFPTAAIINGRRGIEEAYRTGRGKV |
| SRR3137271 | NIPPHNLTEVINGCLAYIDNEDISIEGLMEHIPGPDFPTAAIINGRRGIEEAYRTGRGKV |
| ERR526807 | NIPPHNLTEVINGCLAYIDNEDISIEGLMEHIPGPDFPTAAIINGRRGIEEAYRTGRGKV |
| ERR2197922 | NIPPHNLTEVINGCLAYIDNEDISIEGLMEHIPGPDFPTAAIINGRRGIEEAYRTGRGKV |
| ERR2197923 | NIPPHNLTEVINGCLAYIDNEDISIEGLMEHIPGPDFPTAAIINGRRGIEEAYRTGRGKV |
| ERR2197924 | NIPPHNLTEVINGCLAYIDNEDISIEGLMEHIPGPDFPTAAIINGRRGIEEAYRTGRGKV |
| ERR2197925 | NIPPHNLTEVINGCLAYIDNEDISIEGLMEHIPGPDFPTAAIINGRRGIEEAYRTGRGKV |
| ERR2197927 | NIPPHNLTEVINGCLAYIDNEDISIEGLMEHIPGPDFPTAAIINGRRGIEEAYRTGRGKV |
| ERR2197929 | NIPPHNLTEVINGCLAYIDNEDISIEGLMEHIPGPDFPTAAIINGRRGIEEAYRTGRGKV |
| SRR1648149 | NIPPHNLTEVINGCLAYIDNEDISIEGLMEHIPGPDFPTAAIINGRRGIEEAYRTGRGKV |
| SRR1048299 | NIPPHNLTEVINGCLAYIDNEDISIEGLMEHIPGPDFPTAAIINGRRGIEEAYRTGRGKV |
| SRR1300677 | NIPPHNLTEVINGCLAYIDNEDISIEGLMEHIPGPDFPTAAIINGRRGIEEAYRTGRGKV |
| SRR1288356 | NIPPHNLTEVINGCLAYIDNEDISIEGLMEHIPGPDFPTAAIINGRRGIEEAYRTGRGKV |
| SRR7426190 | NIPPHNLTEVINGCLAYIDNEDISIEGLMEHIPGPDFPTAAIINGRRGIEEAYRTGRGKV |
| SRR7426192 | NIPPHNLTEVINGCLAYIDNEDISIEGLMEHIPGPDFPTAAIINGRRGIEEAYRTGRGKV |
| SRR7426193 | NIPPHNLTEVINGCLAYIDNEDISIEGLMEHIPGPDFPTAAIINGRRGIEEAYRTGRGKV |
| SRR7441832 | NIPPHNLTEVINGCLAYIDNEDISIEGLMEHIPGPDFPTAAIINGRRGIEEAYRTGRGKV |
| SRR7426179 | NIPPHNLTEVINGCLAYIDNEDISIEGLMEHIPGPDFPTAAIINGRRGIEEAYRTGRGKV |
| SRR7439238 | NIPPHNLTEVINGCLAYIDNEDISIEGLMEHIPGPDFPTAAIINGRRGIEEAYRTGRGKV |
| SRR7439244 | NIPPHNLTEVINGCLAYIDNEDISIEGLMEHIPGPDFPTAAIINGRRGIEEAYRTGRGKV |
| SRR7439259 | NIPPHNLTEVINGCLAYIDNEDISIEGLMEHIPGPDFPTAAIINGRRGIEEAYRTGRGKV |
| SRR7439260 | NIPPHNLTEVINGCLAYIDNEDISIEGLMEHIPGPDFPTAAIINGRRGIEEAYRTGRGKV |
| SRR7441786 | NIPPHNLTEVINGCLAYIDNEDISIEGLMEHIPGPDFPTAAIINGRRGIEEAYRTGRGKV |
| SRR7441797 | NIPPHNLTEVINGCLAYIDNEDISIEGLMEHIPGPDFPTAAIINGRRGIEEAYRTGRGKV |
| ERR1759093 | NIPPHNLTEVINGCLAYIDNEDISIEGLMEHIPGPDFPTAAIINGRRGIEEAYRTGRGKV |
| ERR2580275 | NIPPHNLTEVINGCLAYIDNEDISIEGLMEHIPGPDFPTAAIINGRRGIEEAYRTGRGKV |
| ERR1759204 | NIPPHNLTEVINGCLAYIDNEDISIEGLMEHIPGPDFPTAAIINGRRGIEEAYRTGRGKV |
| SRR1300699 | NIPPHNLTEVINGCLAYIDNEDISIEGLMEHIPGPDFPTAAIINGRRGIEEAYRTGRGKV |
| S_0825_17 | NIPPHNLTEVINGCLAYIDNEDISIEGLMEHIPGPDFPTAAIINGRRGIEEAYRTGRGKV |
| SRR1958215 | NIPPHNLTEVINGCLAYIDNEDISIEGLMEHIPGPDFPTAAIINGRRGIEEAYRTGRGKV |
| SRR1958540 | NIPPHNLTEVINGCLAYIDNEDISIEGLMEHIPGPDFPTAAIINGRRGIEEAYRTGRGKV |
| SRR1958636 | NIPPHNLTEVINGCLAYIDNEDISIEGLMEHIPGPDFPTAAIINGRRGIEEAYRTGRGKV |
| SRR1959422 | NIPPHNLTEVINGCLAYIDNEDISIEGLMEHIPGPDFPTAAIINGRRGIEEAYRTGRGKV |
| SRR1959427 | NIPPHNLTEVINGCLAYIDNEDISIEGLMEHIPGPDFPTAAIINGRRGIEEAYRTGRGKV |
| SRR1960226 | NIPPHNLTEVINGCLAYIDNEDISIEGLMEHIPGPDFPTAAIINGRRGIEEAYRTGRGKV |
| SRR1963498 | NIPPHNLTEVINGCLAYIDNEDISIEGLMEHIPGPDFPTAAIINGRRGIEEAYRTGRGKV |
| SRR1965947 | NIPPHNLTEVINGCLAYIDNEDISIEGLMEHIPGPDFPTAAIINGRRGIEEAYRTGRGKV |
| SRR1966125 | NIPPHNLTEVINGCLAYIDNEDISIEGLMEHIPGPDFPTAAIINGRRGIEEAYRTGRGKV |
| SRR1966330 | NIPPHNLTEVINGCLAYIDNEDISIEGLMEHIPGPDFPTAAIINGRRGIEEAYRTGRGKV |
| SRR1966565 | NIPPHNLTEVINGCLAYIDNEDISIEGLMEHIPGPDFPTAAIINGRRGIEEAYRTGRGKV |
| SRR1966864 | NIPPHNLTEVINGCLAYIDNEDISIEGLMEHIPGPDFPTAAIINGRRGIEEAYRTGRGKV |
| SRR1966989 | NIPPHNLTEVINGCLAYIDNEDISIEGLMEHIPGPDFPTAAIINGRRGIEEAYRTGRGKV |
| SRR1967688 | NIPPHNLTEVINGCLAYIDNEDISIEGLMEHIPGPDFPTAAIINGRRGIEEAYRTGRGKV |
| SRR1967733 | NIPPHNLTEVINGCLAYIDNEDISIEGLMEHIPGPDFPTAAIINGRRGIEEAYRTGRGKV |
| SRR1967746 | NIPPHNLTEVINGCLAYIDNEDISIEGLMEHIPGPDFPTAAIINGRRGIEEAYRTGRGKV |
| SRR1968341 | NIPPHNLTEVINGCLAYIDNEDISIEGLMEHIPGPDFPTAAIINGRRGIEEAYRTGRGKV |
| SRR1968456 | NIPPHNLTEVINGCLAYIDNEDISIEGLMEHIPGPDFPTAAIINGRRGIEEAYRTGRGKV |
| SRR1968465 | NIPPHNLTEVINGCLAYIDNEDISIEGLMEHIPGPDFPTAAIINGRRGIEEAYRTGRGKV |
| SRR1968761 | NIPPHNLTEVINGCLAYIDNEDISIEGLMEHIPGPDFPTAAIINGRRGIEEAYRTGRGKV |
| SRR1969047 | NIPPHNLTEVINGCLAYIDNEDISIEGLMEHIPGPDFPTAAIINGRRGIEEAYRTGRGKV |
| SRR1969255 | NIPPHNLTEVINGCLAYIDNEDISIEGLMEHIPGPDFPTAAIINGRRGIEEAYRTGRGKV |
| SRR1969412 | NIPPHNLTEVINGCLAYIDNEDISIEGLMEHIPGPDFPTAAIINGRRGIEEAYRTGRGKV |
| SRR1969524 | NIPPHNLTEVINGCLAYIDNEDISIEGLMEHIPGPDFPTAAIINGRRGIEEAYRTGRGKV |
| SRR1969584 | NIPPHNLTEVINGCLAYIDNEDISIEGLMEHIPGPDFPTAAIINGRRGIEEAYRTGRGKV |
| SRR1969648 | NIPPHNLTEVINGCLAYIDNEDISIEGLMEHIPGPDFPTAAIINGRRGIEEAYRTGRGKV |
| SRR1969804 | NIPPHNLTEVINGCLAYIDNEDISIEGLMEHIPGPDFPTAAIINGRRGIEEAYRTGRGKV |
| SRR1970221 | NIPPHNLTEVINGCLAYIDNEDISIEGLMEHIPGPDFPTAAIINGRRGIEEAYRTGRGKV |
| SRR1970268 | NIPPHNLTEVINGCLAYIDNEDISIEGLMEHIPGPDFPTAAIINGRRGIEEAYRTGRGKV |
| SRR1965862 | NIPPHNLTEVINGCLAYIDNEDISIEGLMEHIPGPDFPTAAIINGRRGIEEAYRTGRGKV |
| SRR1967363 | NIPPHNLTEVINGCLAYIDNEDISIEGLMEHIPGPDFPTAAIINGRRGIEEAYRTGRGKV |
| SRR1968276 | NIPPHNLTEVINGCLAYIDNEDISIEGLMEHIPGPDFPTAAIINGRRGIEEAYRTGRGKV |
| SRR1968967 | NIPPHNLTEVINGCLAYIDNEDISIEGLMEHIPGPDFPTAAIINGRRGIEEAYRTGRGKV |
| SRR3321531 | NIPPHNLTEVINGCLAYIDNEDISIEGLMEHIPGPDFPTAAIINGRRGIEEAYRTGRGKV |
| SRR3321883 | NIPPHNLTEVINGCLAYIDNEDISIEGLMEHIPGPDFPTAAIINGRRGIEEAYRTGRGKV |
| SRR3322413 | NIPPHNLTEVINGCLAYIDNEDISIEGLMEHIPGPDFPTAAIINGRRGIEEAYRTGRGKV |
| SRR3323012 | NIPPHNLTEVINGCLAYIDNEDISIEGLMEHIPGPDFPTAAIINGRRGIEEAYRTGRGKV |
| SRR5194289 | NIPPHNLTEVINGCLAYIDNEDISIEGLMEHIPGPDFPTAAIINGRRGIEEAYRTGRGKV |
| SRR7163798 | NIPPHNLTEVINGCLAYIDNEDISIEGLMEHIPGPDFPTAAIINGRRGIEEAYRTGRGKV |
| SRR7172610 | NIPPHNLTEVINGCLAYIDNEDISIEGLMEHIPGPDFPTAAIINGRRGIEEAYRTGRGKV |
| SRR7204568 | NIPPHNLTEVINGCLAYIDNEDISIEGLMEHIPGPDFPTAAIINGRRGIEEAYRTGRGKV |
| SRR7223230 | NIPPHNLTEVINGCLAYIDNEDISIEGLMEHIPGPDFPTAAIINGRRGIEEAYRTGRGKV |
| SRR7230675 | NIPPHNLTEVINGCLAYIDNEDISIEGLMEHIPGPDFPTAAIINGRRGIEEAYRTGRGKV |
| SRR7278056 | NIPPHNLTEVINGCLAYIDNEDISIEGLMEHIPGPDFPTAAIINGRRGIEEAYRTGRGKV |
| SRR7278086 | NIPPHNLTEVINGCLAYIDNEDISIEGLMEHIPGPDFPTAAIINGRRGIEEAYRTGRGKV |
| SRR7285841 | NIPPHNLTEVINGCLAYIDNEDISIEGLMEHIPGPDFPTAAIINGRRGIEEAYRTGRGKV |
| SRR7292625 | NIPPHNLTEVINGCLAYIDNEDISIEGLMEHIPGPDFPTAAIINGRRGIEEAYRTGRGKV |
| SRR7292665 | NIPPHNLTEVINGCLAYIDNEDISIEGLMEHIPGPDFPTAAIINGRRGIEEAYRTGRGKV |
| SRR7297965 | NIPPHNLTEVINGCLAYIDNEDISIEGLMEHIPGPDFPTAAIINGRRGIEEAYRTGRGKV |
| SRR7350726 | NIPPHNLTEVINGCLAYIDNEDISIEGLMEHIPGPDFPTAAIINGRRGIEEAYRTGRGKV |
| SRR7410328 | NIPPHNLTEVINGCLAYIDNEDISIEGLMEHIPGPDFPTAAIINGRRGIEEAYRTGRGKV |
| SRR7474665 | NIPPHNLTEVINGCLAYIDNEDISIEGLMEHIPGPDFPTAAIINGRRGIEEAYRTGRGKV |
| SRR7523184 | NIPPHNLTEVINGCLAYIDNEDISIEGLMEHIPGPDFPTAAIINGRRGIEEAYRTGRGKV |
| SRR7187264 | NIPPHNLTEVINGCLAYIDNEDISIEGLMEHIPGPDFPTAAIINGRRGIEEAYRTGRGKV |
| SRR7204445 | NIPPHNLTEVINGCLAYIDNEDISIEGLMEHIPGPDFPTAAIINGRRGIEEAYRTGRGKV |
| SRR7285641 | NIPPHNLTEVINGCLAYIDNEDISIEGLMEHIPGPDFPTAAIINGRRGIEEAYRTGRGKV |
| SRR7286695 | NIPPHNLTEVINGCLAYIDNEDISIEGLMEHIPGPDFPTAAIINGRRGIEEAYRTGRGKV |
| SRR7286705 | NIPPHNLTEVINGCLAYIDNEDISIEGLMEHIPGPDFPTAAIINGRRGIEEAYRTGRGKV |
| SRR7292931 | NIPPHNLTEVINGCLAYIDNEDISIEGLMEHIPGPDFPTAAIINGRRGIEEAYRTGRGKV |
| SRR7310349 | NIPPHNLTEVINGCLAYIDNEDISIEGLMEHIPGPDFPTAAIINGRRGIEEAYRTGRGKV |
| SRR7351616 | NIPPHNLTEVINGCLAYIDNEDISIEGLMEHIPGPDFPTAAIINGRRGIEEAYRTGRGKV |
| SRR7414818 | NIPPHNLTEVINGCLAYIDNEDISIEGLMEHIPGPDFPTAAIINGRRGIEEAYRTGRGKV |
| SRR7426480 | NIPPHNLTEVINGCLAYIDNEDISIEGLMEHIPGPDFPTAAIINGRRGIEEAYRTGRGKV |
| SRR5584105 | NIPPHNLTEVINGCLAYIDNEDISIEGLMEHIPGPDFPTAAIINGRRGIEEAYRTGRGKV |
| SRR5584565 | NIPPHNLTEVINGCLAYIDNEDISIEGLMEHIPGPDFPTAAIINGRRGIEEAYRTGRGKV |
| SRR5584614 | NIPPHNLTEVINGCLAYIDNEDISIEGLMEHIPGPDFPTAAIINGRRGIEEAYRTGRGKV |
| SRR5631543 | NIPPHNLTEVINGCLAYIDNEDISIEGLMEHIPGPDFPTAAIINGRRGIEEAYRTGRGKV |
| SRR5631553 | NIPPHNLTEVINGCLAYIDNEDISIEGLMEHIPGPDFPTAAIINGRRGIEEAYRTGRGKV |
| SRR7123196 | NIPPHNLTEVINGCLAYIDNEDISIEGLMEHIPGPDFPTAAIINGRRGIEEAYRTGRGKV |
| SRR7163819 | NIPPHNLTEVINGCLAYIDNEDISIEGLMEHIPGPDFPTAAIINGRRGIEEAYRTGRGKV |
| SRR7163920 | NIPPHNLTEVINGCLAYIDNEDISIEGLMEHIPGPDFPTAAIINGRRGIEEAYRTGRGKV |
| SRR7209528 | NIPPHNLTEVINGCLAYIDNEDISIEGLMEHIPGPDFPTAAIINGRRGIEEAYRTGRGKV |
| SRR7249868 | NIPPHNLTEVINGCLAYIDNEDISIEGLMEHIPGPDFPTAAIINGRRGIEEAYRTGRGKV |
| SRR7278088 | NIPPHNLTEVINGCLAYIDNEDISIEGLMEHIPGPDFPTAAIINGRRGIEEAYRTGRGKV |
| SRR7285788 | NIPPHNLTEVINGCLAYIDNEDISIEGLMEHIPGPDFPTAAIINGRRGIEEAYRTGRGKV |
| SRR7286789 | NIPPHNLTEVINGCLAYIDNEDISIEGLMEHIPGPDFPTAAIINGRRGIEEAYRTGRGKV |
| SRR7286886 | NIPPHNLTEVINGCLAYIDNEDISIEGLMEHIPGPDFPTAAIINGRRGIEEAYRTGRGKV |
| SRR7310632 | NIPPHNLTEVINGCLAYIDNEDISIEGLMEHIPGPDFPTAAIINGRRGIEEAYRTGRGKV |
| SRR7350631 | NIPPHNLTEVINGCLAYIDNEDISIEGLMEHIPGPDFPTAAIINGRRGIEEAYRTGRGKV |
| SRR7458741 | NIPPHNLTEVINGCLAYIDNEDISIEGLMEHIPGPDFPTAAIINGRRGIEEAYRTGRGKV |
| SRR7480280 | NIPPHNLTEVINGCLAYIDNEDISIEGLMEHIPGPDFPTAAIINGRRGIEEAYRTGRGKV |
| SRR7523660 | NIPPHNLTEVINGCLAYIDNEDISIEGLMEHIPGPDFPTAAIINGRRGIEEAYRTGRGKV |
| SRR7523775 | NIPPHNLTEVINGCLAYIDNEDISIEGLMEHIPGPDFPTAAIINGRRGIEEAYRTGRGKV |
| SRR7251101 | NIPPHNLTEVINGCLAYIDNEDISIEGLMEHIPGPDFPTAAIINGRRGIEEAYRTGRGKV |
| SRR7284299 | NIPPHNLTEVINGCLAYIDNEDISIEGLMEHIPGPDFPTAAIINGRRGIEEAYRTGRGKV |
| SRR7285738 | NIPPHNLTEVINGCLAYIDNEDISIEGLMEHIPGPDFPTAAIINGRRGIEEAYRTGRGKV |
| SRR7310640 | NIPPHNLTEVINGCLAYIDNEDISIEGLMEHIPGPDFPTAAIINGRRGIEEAYRTGRGKV |
| SRR7349159 | NIPPHNLTEVINGCLAYIDNEDISIEGLMEHIPGPDFPTAAIINGRRGIEEAYRTGRGKV |
| SRR7474873 | NIPPHNLTEVINGCLAYIDNEDISIEGLMEHIPGPDFPTAAIINGRRGIEEAYRTGRGKV |
| SRR7495689 | NIPPHNLTEVINGCLAYIDNEDISIEGLMEHIPGPDFPTAAIINGRRGIEEAYRTGRGKV |
| SRR7495752 | NIPPHNLTEVINGCLAYIDNEDISIEGLMEHIPGPDFPTAAIINGRRGIEEAYRTGRGKV |
| ----------------------------------------------------------------------------- | |
| S16BD08730 | YIRARAEVEADAKTGRETIIVHEIPYQVNKARLIEKIAELVKDKRVEGISALRDESDKDG |
| S18BD00684 | YIRARAEVEADAKTGRETIIVHEIPYQVNKARLIEKIAELVKDKRVEGISALRDESDKDG |
| S18BD03994 | YIRARAEVEADAKTGRETIIVHEIPYQVNKARLIEKIAELVKDKRVEGISALRDESDKDG |
| S18BD05011 | YIRARAEVEADAKTGRETIIVHEIPYQVNKARLIEKIAELVKDKRVEGISALRDESDKDG |
| RKI_16-03723 | YIRARAEVEADAKTGRETIIVHEIPYQVNKARLIEKIAELVKDKRVEGISALRDESDKDG |
| RKI_16-04315 | YIRARAEVEADAKTGRETIIVHEIPYQVNKARLIEKIAELVKDKRVEGISALRDESDKDG |
| RKI_17-02304 | YIRARAEVEADAKTGRETIIVHEIPYQVNKARLIEKIAELVKDKRVEGISALRDESDKDG |
| RKI_17-02411 | YIRARAEVEADAKTGRETIIVHEIPYQVNKARLIEKIAELVKDKRVEGISALRDESDKDG |
| RKI_17-02757 | YIRARAEVEADAKTGRETIIVHEIPYQVNKARLIEKIAELVKDKRVEGISALRDESDKDG |
| RKI_17-04797 | YIRARAEVEADAKTGRETIIVHEIPYQVNKARLIEKIAELVKDKRVEGISALRDESDKDG |
| RKI_17-06869 | YIRARAEVEADAKTGRETIIVHEIPYQVNKARLIEKIAELVKDKRVEGISALRDESDKDG |
| ERR2580277 | YIRARAEVEADAKTGRETIIVHEIPYQVNKARLIEKIAELVKDKRVEGISALRDESDKDG |
| ERR2580276 | YIRARAEVEADAKTGRETIIVHEIPYQVNKARLIEKIAELVKDKRVEGISALRDESDKDG |
| ERR2580273 | YIRARAEVEADAKTGRETIIVHEIPYQVNKARLIEKIAELVKDKRVEGISALRDESDKDG |
| ERR2580274 | YIRARAEVEADAKTGRETIIVHEIPYQVNKARLIEKIAELVKDKRVEGISALRDESDKDG |
| ERR2173656 | YIRARAEVEADAKTGRETIIVHEIPYQVNKARLIEKIAELVKDKRVEGISALRDESDKDG |
| 17041676 | YIRARAEVEADAKTGRETIIVHEIPYQVNKARLIEKIAELVKDKRVEGISALRDESDKDG |
| MT16-000061 | YIRARAEVEADAKTGRETIIVHEIPYQVNKARLIEKIAELVKDKRVEGISALRDESDKDG |
| MT16-019416 | YIRARAEVEADAKTGRETIIVHEIPYQVNKARLIEKIAELVKDKRVEGISALRDESDKDG |
| MT16-027865 | YIRARAEVEADAKTGRETIIVHEIPYQVNKARLIEKIAELVKDKRVEGISALRDESDKDG |
| MT16-031693 | YIRARAEVEADAKTGRETIIVHEIPYQVNKARLIEKIAELVKDKRVEGISALRDESDKDG |
| MT16-040253 | YIRARAEVEADAKTGRETIIVHEIPYQVNKARLIEKIAELVKDKRVEGISALRDESDKDG |
| MT16-045379 | YIRARAEVEADAKTGRETIIVHEIPYQVNKARLIEKIAELVKDKRVEGISALRDESDKDG |
| MT16-442728 | YIRARAEVEADAKTGRETIIVHEIPYQVNKARLIEKIAELVKDKRVEGISALRDESDKDG |
| MT16-462857 | YIRARAEVEADAKTGRETIIVHEIPYQVNKARLIEKIAELVKDKRVEGISALRDESDKDG |
| MT16-480196 | YIRARAEVEADAKTGRETIIVHEIPYQVNKARLIEKIAELVKDKRVEGISALRDESDKDG |
| MT16-861555 | YIRARAEVEADAKTGRETIIVHEIPYQVNKARLIEKIAELVKDKRVEGISALRDESDKDG |
| MT17-076833 | YIRARAEVEADAKTGRETIIVHEIPYQVNKARLIEKIAELVKDKRVEGISALRDESDKDG |
| MT17-110677 | YIRARAEVEADAKTGRETIIVHEIPYQVNKARLIEKIAELVKDKRVEGISALRDESDKDG |
| MT17-131730 | YIRARAEVEADAKTGRETIIVHEIPYQVNKARLIEKIAELVKDKRVEGISALRDESDKDG |
| MT17-140890 | YIRARAEVEADAKTGRETIIVHEIPYQVNKARLIEKIAELVKDKRVEGISALRDESDKDG |
| MT17-141840 | YIRARAEVEADAKTGRETIIVHEIPYQVNKARLIEKIAELVKDKRVEGISALRDESDKDG |
| MT17-152488 | YIRARAEVEADAKTGRETIIVHEIPYQVNKARLIEKIAELVKDKRVEGISALRDESDKDG |
| MT17-157311 | YIRARAEVEADAKTGRETIIVHEIPYQVNKARLIEKIAELVKDKRVEGISALRDESDKDG |
| MT17-161645 | YIRARAEVEADAKTGRETIIVHEIPYQVNKARLIEKIAELVKDKRVEGISALRDESDKDG |
| MT17-167951 | YIRARAEVEADAKTGRETIIVHEIPYQVNKARLIEKIAELVKDKRVEGISALRDESDKDG |
| MT18-217732 | YIRARAEVEADAKTGRETIIVHEIPYQVNKARLIEKIAELVKDKRVEGISALRDESDKDG |
| MT18-252580 | YIRARAEVEADAKTGRETIIVHEIPYQVNKARLIEKIAELVKDKRVEGISALRDESDKDG |
| RIVM_H_2009-01 | YIRARAEVEADAKTGRETIIVHEIPYQVNKARLIEKIAELVKDKRVEGISALRDESDKDG |
| RIVM_H_2010-01 | YIRARAEVEADAKTGRETIIVHEIPYQVNKARLIEKIAELVKDKRVEGISALRDESDKDG |
| RIVM_H_2010-02 | YIRARAEVEADAKTGRETIIVHEIPYQVNKARLIEKIAELVKDKRVEGISALRDESDKDG |
| RIVM_H_2011-01 | YIRARAEVEADAKTGRETIIVHEIPYQVNKARLIEKIAELVKDKRVEGISALRDESDKDG |
| RIVM_H_2011-02 | YIRARAEVEADAKTGRETIIVHEIPYQVNKARLIEKIAELVKDKRVEGISALRDESDKDG |
| RIVM_H_2011-03 | YIRARAEVEADAKTGRETIIVHEIPYQVNKARLIEKIAELVKDKRVEGISALRDESDKDG |
| RIVM_H_2013-01 | YIRARAEVEADAKTGRETIIVHEIPYQVNKARLIEKIAELVKDKRVEGISALRDESDKDG |
| RIVM_H_2013-02 | YIRARAEVEADAKTGRETIIVHEIPYQVNKARLIEKIAELVKDKRVEGISALRDESDKDG |
| RIVM_H_2014-01 | YIRARAEVEADAKTGRETIIVHEIPYQVNKARLIEKIAELVKDKRVEGISALRDESDKDG |
| RIVM_H_2014-02 | YIRARAEVEADAKTGRETIIVHEIPYQVNKARLIEKIAELVKDKRVEGISALRDESDKDG |
| RIVM_H_2016-01 | YIRARAEVEADAKTGRETIIVHEIPYQVNKARLIEKIAELVKDKRVEGISALRDESDKDG |
| RIVM_H_2016-02 | YIRARAEVEADAKTGRETIIVHEIPYQVNKARLIEKIAELVKDKRVEGISALRDESDKDG |
| RIVM_H_2016-03 | YIRARAEVEADAKTGRETIIVHEIPYQVNKARLIEKIAELVKDKRVEGISALRDESDKDG |
| RIVM_H_2016-04 | YIRARAEVEADAKTGRETIIVHEIPYQVNKARLIEKIAELVKDKRVEGISALRDESDKDG |
| RIVM_H_2016-05 | YIRARAEVEADAKTGRETIIVHEIPYQVNKARLIEKIAELVKDKRVEGISALRDESDKDG |
| RIVM_H_2016-06 | YIRARAEVEADAKTGRETIIVHEIPYQVNKARLIEKIAELVKDKRVEGISALRDESDKDG |
| RIVM_H_2016-07 | YIRARAEVEADAKTGRETIIVHEIPYQVNKARLIEKIAELVKDKRVEGISALRDESDKDG |
| RIVM_H_2016-08 | YIRARAEVEADAKTGRETIIVHEIPYQVNKARLIEKIAELVKDKRVEGISALRDESDKDG |
| RIVM_H_2016-09 | YIRARAEVEADAKTGRETIIVHEIPYQVNKARLIEKIAELVKDKRVEGISALRDESDKDG |
| RIVM_H_2016-10 | YIRARAEVEADAKTGRETIIVHEIPYQVNKARLIEKIAELVKDKRVEGISALRDESDKDG |
| RIVM_H_2016-11 | YIRARAEVEADAKTGRETIIVHEIPYQVNKARLIEKIAELVKDKRVEGISALRDESDKDG |
| RIVM_H_2016-12 | YIRARAEVEADAKTGRETIIVHEIPYQVNKARLIEKIAELVKDKRVEGISALRDESDKDG |
| RIVM_H_2016-13 | YIRARAEVEADAKTGRETIIVHEIPYQVNKARLIEKIAELVKDKRVEGISALRDESDKDG |
| RIVM_H_2016-14 | YIRARAEVEADAKTGRETIIVHEIPYQVNKARLIEKIAELVKDKRVEGISALRDESDKDG |
| RIVM_H_2016-15 | YIRARAEVEADAKTGRETIIVHEIPYQVNKARLIEKIAELVKDKRVEGISALRDESDKDG |
| RIVM_H_2017-01 | YIRARAEVEADAKTGRETIIVHEIPYQVNKARLIEKIAELVKDKRVEGISALRDESDKDG |
| RIVM_H_2017-02 | YIRARAEVEADAKTGRETIIVHEIPYQVNKARLIEKIAELVKDKRVEGISALRDESDKDG |
| RIVM_H_2017-03 | YIRARAEVEADAKTGRETIIVHEIPYQVNKARLIEKIAELVKDKRVEGISALRDESDKDG |
| RIVM_H_2017-04 | YIRARAEVEADAKTGRETIIVHEIPYQVNKARLIEKIAELVKDKRVEGISALRDESDKDG |
| RIVM_H_2017-05 | YIRARAEVEADAKTGRETIIVHEIPYQVNKARLIEKIAELVKDKRVEGISALRDESDKDG |
| RIVM_H_2017-06 | YIRARAEVEADAKTGRETIIVHEIPYQVNKARLIEKIAELVKDKRVEGISALRDESDKDG |
| RIVM_H_2017-07 | YIRARAEVEADAKTGRETIIVHEIPYQVNKARLIEKIAELVKDKRVEGISALRDESDKDG |
| RIVM_H_2017-08 | YIRARAEVEADAKTGRETIIVHEIPYQVNKARLIEKIAELVKDKRVEGISALRDESDKDG |
| RIVM_H_2017-09 | YIRARAEVEADAKTGRETIIVHEIPYQVNKARLIEKIAELVKDKRVEGISALRDESDKDG |
| RIVM_H_2017-10 | YIRARAEVEADAKTGRETIIVHEIPYQVNKARLIEKIAELVKDKRVEGISALRDESDKDG |
| RIVM_H_2017-11 | YIRARAEVEADAKTGRETIIVHEIPYQVNKARLIEKIAELVKDKRVEGISALRDESDKDG |
| RIVM_H_2017-12 | YIRARAEVEADAKTGRETIIVHEIPYQVNKARLIEKIAELVKDKRVEGISALRDESDKDG |
| RIVM_H_2017-13 | YIRARAEVEADAKTGRETIIVHEIPYQVNKARLIEKIAELVKDKRVEGISALRDESDKDG |
| RIVM_H_2017-14 | YIRARAEVEADAKTGRETIIVHEIPYQVNKARLIEKIAELVKDKRVEGISALRDESDKDG |
| RIVM_H_2017-15 | YIRARAEVEADAKTGRETIIVHEIPYQVNKARLIEKIAELVKDKRVEGISALRDESDKDG |
| RIVM_H_2017-16 | YIRARAEVEADAKTGRETIIVHEIPYQVNKARLIEKIAELVKDKRVEGISALRDESDKDG |
| RIVM_H_2017-17 | YIRARAEVEADAKTGRETIIVHEIPYQVNKARLIEKIAELVKDKRVEGISALRDESDKDG |
| RIVM_H_2017-18 | YIRARAEVEADAKTGRETIIVHEIPYQVNKARLIEKIAELVKDKRVEGISALRDESDKDG |
| RIVM_H_2017-19 | YIRARAEVEADAKTGRETIIVHEIPYQVNKARLIEKIAELVKDKRVEGISALRDESDKDG |
| 15EP001483 | YIRARAEVEADAKTGRETIIVHEIPYQVNKARLIEKIAELVKDKRVEGISALRDESDKDG |
| 17EP002363 | YIRARAEVEADAKTGRETIIVHEIPYQVNKARLIEKIAELVKDKRVEGISALRDESDKDG |
| S_0812_17 | YIRARAEVEADAKTGRETIIVHEIPYQVNKARLIEKIAELVKDKRVEGISALRDESDKDG |
| SRR1957844 | YIRARAEVEADAKTGRETIIVHEIPYQVNKARLIEKIAELVKDKRVEGISALRDESDKDG |
| SRR1958654 | YIRARAEVEADAKTGRETIIVHEIPYQVNKARLIEKIAELVKDKRVEGISALRDESDKDG |
| SRR1965077 | YIRARAEVEADAKTGRETIIVHEIPYQVNKARLIEKIAELVKDKRVEGISALRDESDKDG |
| SRR1966369 | YIRARAEVEADAKTGRETIIVHEIPYQVNKARLIEKIAELVKDKRVEGISALRDESDKDG |
| SRR1967117 | YIRARAEVEADAKTGRETIIVHEIPYQVNKARLIEKIAELVKDKRVEGISALRDESDKDG |
| SRR1967922 | YIRARAEVEADAKTGRETIIVHEIPYQVNKARLIEKIAELVKDKRVEGISALRDESDKDG |
| SRR8704720 | YIRARAEVEADAKTGRETIIVHEIPYQVNKARLIEKIAELVKDKRVEGISALRDESDKDG |
| SRR7216071 | YIRARAEVEADAKTGRETIIVHEIPYQVNKARLIEKIAELVKDKRVEGISALRDESDKDG |
| SRR7349175 | YIRARAEVEADAKTGRETIIVHEIPYQVNKARLIEKIAELVKDKRVEGISALRDESDKDG |
| SRR7523148 | YIRARAEVEADAKTGRETIIVHEIPYQVNKARLIEKIAELVKDKRVEGISALRDESDKDG |
| SRR7523854 | YIRARAEVEADAKTGRETIIVHEIPYQVNKARLIEKIAELVKDKRVEGISALRDESDKDG |
| 313865 | YIRARAEVEADAKTGRETIIVHEIPYQVNKARLIEKIAELVKDKRVEGISALRDESDKDG |
| SRR7277793 | YIRARAEVEADAKTGRETIIVHEIPYQVNKARLIEKIAELVKDKRVEGISALRDESDKDG |
| SRR7343877 | YIRARAEVEADAKTGRETIIVHEIPYQVNKARLIEKIAELVKDKRVEGISALRDESDKDG |
| SRR7351477 | YIRARAEVEADAKTGRETIIVHEIPYQVNKARLIEKIAELVKDKRVEGISALRDESDKDG |
| SRR5583183 | YIRARAEVEADAKTGRETIIVHEIPYQVNKARLIEKIAELVKDKRVEGISALRDESDKDG |
| SRR5585240 | YIRARAEVEADAKTGRETIIVHEIPYQVNKARLIEKIAELVKDKRVEGISALRDESDKDG |
| SRR7284317 | YIRARAEVEADAKTGRETIIVHEIPYQVNKARLIEKIAELVKDKRVEGISALRDESDKDG |
| SRR7299161 | YIRARAEVEADAKTGRETIIVHEIPYQVNKARLIEKIAELVKDKRVEGISALRDESDKDG |
| SRR7401730 | YIRARAEVEADAKTGRETIIVHEIPYQVNKARLIEKIAELVKDKRVEGISALRDESDKDG |
| SRR7469092 | YIRARAEVEADAKTGRETIIVHEIPYQVNKARLIEKIAELVKDKRVEGISALRDESDKDG |
| SRR7879556 | YIRARAEVEADAKTGRETIIVHEIPYQVNKARLIEKIAELVKDKRVEGISALRDESDKDG |
| SRR8526100 | YIRARAEVEADAKTGRETIIVHEIPYQVNKARLIEKIAELVKDKRVEGISALRDESDKDG |
| SRR8553991 | YIRARAEVEADAKTGRETIIVHEIPYQVNKARLIEKIAELVKDKRVEGISALRDESDKDG |
| SRR7842487 | YIRARAEVEADAKTGRETIIVHEIPYQVNKARLIEKIAELVKDKRVEGISALRDESDKDG |
| SRR8054524 | YIRARAEVEADAKTGRETIIVHEIPYQVNKARLIEKIAELVKDKRVEGISALRDESDKDG |
| SRR8054525 | YIRARAEVEADAKTGRETIIVHEIPYQVNKARLIEKIAELVKDKRVEGISALRDESDKDG |
| SRR8524733 | YIRARAEVEADAKTGRETIIVHEIPYQVNKARLIEKIAELVKDKRVEGISALRDESDKDG |
| SRR4093291 | YIRARAEVEADAKTGRETIIVHEIPYQVNKARLIEKIAELVKDKRVEGISALRDESDKDG |
| SRR4245549 | YIRARAEVEADAKTGRETIIVHEIPYQVNKARLIEKIAELVKDKRVEGISALRDESDKDG |
| SRR3057154 | YIRARAEVEADAKTGRETIIVHEIPYQVNKARLIEKIAELVKDKRVEGISALRDESDKDG |
| SRR1726150 | YIRARAEVEADAKTGRETIIVHEIPYQVNKARLIEKIAELVKDKRVEGISALRDESDKDG |
| SRR1996141 | YIRARAEVEADAKTGRETIIVHEIPYQVNKARLIEKIAELVKDKRVEGISALRDESDKDG |
| SRR1107842 | YIRARAEVEADAKTGRETIIVHEIPYQVNKARLIEKIAELVKDKRVEGISALRDESDKDG |
| SRR1157587 | YIRARAEVEADAKTGRETIIVHEIPYQVNKARLIEKIAELVKDKRVEGISALRDESDKDG |
| SRR3027706 | YIRARAEVEADAKTGRETIIVHEIPYQVNKARLIEKIAELVKDKRVEGISALRDESDKDG |
| SRR3027707 | YIRARAEVEADAKTGRETIIVHEIPYQVNKARLIEKIAELVKDKRVEGISALRDESDKDG |
| SRR3027708 | YIRARAEVEADAKTGRETIIVHEIPYQVNKARLIEKIAELVKDKRVEGISALRDESDKDG |
| SRR3027710 | YIRARAEVEADAKTGRETIIVHEIPYQVNKARLIEKIAELVKDKRVEGISALRDESDKDG |
| SRR3027711 | YIRARAEVEADAKTGRETIIVHEIPYQVNKARLIEKIAELVKDKRVEGISALRDESDKDG |
| SRR3027716 | YIRARAEVEADAKTGRETIIVHEIPYQVNKARLIEKIAELVKDKRVEGISALRDESDKDG |
| SRR3027717 | YIRARAEVEADAKTGRETIIVHEIPYQVNKARLIEKIAELVKDKRVEGISALRDESDKDG |
| SRR3027719 | YIRARAEVEADAKTGRETIIVHEIPYQVNKARLIEKIAELVKDKRVEGISALRDESDKDG |
| SRR3027721 | YIRARAEVEADAKTGRETIIVHEIPYQVNKARLIEKIAELVKDKRVEGISALRDESDKDG |
| SRR3027723 | YIRARAEVEADAKTGRETIIVHEIPYQVNKARLIEKIAELVKDKRVEGISALRDESDKDG |
| SRR3115978 | YIRARAEVEADAKTGRETIIVHEIPYQVNKARLIEKIAELVKDKRVEGISALRDESDKDG |
| SRR2534093 | YIRARAEVEADAKTGRETIIVHEIPYQVNKARLIEKIAELVKDKRVEGISALRDESDKDG |
| SRR2534094 | YIRARAEVEADAKTGRETIIVHEIPYQVNKARLIEKIAELVKDKRVEGISALRDESDKDG |
| SRR2534095 | YIRARAEVEADAKTGRETIIVHEIPYQVNKARLIEKIAELVKDKRVEGISALRDESDKDG |
| SRR2534108 | YIRARAEVEADAKTGRETIIVHEIPYQVNKARLIEKIAELVKDKRVEGISALRDESDKDG |
| SRR1106464 | YIRARAEVEADAKTGRETIIVHEIPYQVNKARLIEKIAELVKDKRVEGISALRDESDKDG |
| SRR1106463 | YIRARAEVEADAKTGRETIIVHEIPYQVNKARLIEKIAELVKDKRVEGISALRDESDKDG |
| SRR6949610 | YIRARAEVEADAKTGRETIIVHEIPYQVNKARLIEKIAELVKDKRVEGISALRDESDKDG |
| SRR6950452 | YIRARAEVEADAKTGRETIIVHEIPYQVNKARLIEKIAELVKDKRVEGISALRDESDKDG |
| ERR2019831 | YIRARAEVEADAKTGRETIIVHEIPYQVNKARLIEKIAELVKDKRVEGISALRDESDKDG |
| SRR2085693 | YIRARAEVEADAKTGRETIIVHEIPYQVNKARLIEKIAELVKDKRVEGISALRDESDKDG |
| SRR2086898 | YIRARAEVEADAKTGRETIIVHEIPYQVNKARLIEKIAELVKDKRVEGISALRDESDKDG |
| SRR2175312 | YIRARAEVEADAKTGRETIIVHEIPYQVNKARLIEKIAELVKDKRVEGISALRDESDKDG |
| SRR2175360 | YIRARAEVEADAKTGRETIIVHEIPYQVNKARLIEKIAELVKDKRVEGISALRDESDKDG |
| SRR5231997 | YIRARAEVEADAKTGRETIIVHEIPYQVNKARLIEKIAELVKDKRVEGISALRDESDKDG |
| SRR5232003 | YIRARAEVEADAKTGRETIIVHEIPYQVNKARLIEKIAELVKDKRVEGISALRDESDKDG |
| SRR5232015 | YIRARAEVEADAKTGRETIIVHEIPYQVNKARLIEKIAELVKDKRVEGISALRDESDKDG |
| SRR949434 | YIRARAEVEADAKTGRETIIVHEIPYQVNKARLIEKIAELVKDKRVEGISALRDESDKDG |
| SRR3216575 | YIRARAEVEADAKTGRETIIVHEIPYQVNKARLIEKIAELVKDKRVEGISALRDESDKDG |
| SRR5205342 | YIRARAEVEADAKTGRETIIVHEIPYQVNKARLIEKIAELVKDKRVEGISALRDESDKDG |
| SRR1501669 | YIRARAEVEADAKTGRETIIVHEIPYQVNKARLIEKIAELVKDKRVEGISALRDESDKDG |
| SRR5209740 | YIRARAEVEADAKTGRETIIVHEIPYQVNKARLIEKIAELVKDKRVEGISALRDESDKDG |
| SRR3240355 | YIRARAEVEADAKTGRETIIVHEIPYQVNKARLIEKIAELVKDKRVEGISALRDESDKDG |
| SRR3392777 | YIRARAEVEADAKTGRETIIVHEIPYQVNKARLIEKIAELVKDKRVEGISALRDESDKDG |
| SRR3593671 | YIRARAEVEADAKTGRETIIVHEIPYQVNKARLIEKIAELVKDKRVEGISALRDESDKDG |
| SRR5413290 | YIRARAEVEADAKTGRETIIVHEIPYQVNKARLIEKIAELVKDKRVEGISALRDESDKDG |
| SRR5590269 | YIRARAEVEADAKTGRETIIVHEIPYQVNKARLIEKIAELVKDKRVEGISALRDESDKDG |
| SRR5812103 | YIRARAEVEADAKTGRETIIVHEIPYQVNKARLIEKIAELVKDKRVEGISALRDESDKDG |
| SRR2830941 | YIRARAEVEADAKTGRETIIVHEIPYQVNKARLIEKIAELVKDKRVEGISALRDESDKDG |
| SRR2830966 | YIRARAEVEADAKTGRETIIVHEIPYQVNKARLIEKIAELVKDKRVEGISALRDESDKDG |
| SRR3137270 | YIRARAEVEADAKTGRETIIVHEIPYQVNKARLIEKIAELVKDKRVEGISALRDESDKDG |
| SRR3137271 | YIRARAEVEADAKTGRETIIVHEIPYQVNKARLIEKIAELVKDKRVEGISALRDESDKDG |
| ERR526807 | YIRARAEVEADAKTGRETIIVHEIPYQVNKARLIEKIAELVKDKRVEGISALRDESDKDG |
| ERR2197922 | YIRARAEVEADAKTGRETIIVHEIPYQVNKARLIEKIAELVKDKRVEGISALRDESDKDG |
| ERR2197923 | YIRARAEVEADAKTGRETIIVHEIPYQVNKARLIEKIAELVKDKRVEGISALRDESDKDG |
| ERR2197924 | YIRARAEVEADAKTGRETIIVHEIPYQVNKARLIEKIAELVKDKRVEGISALRDESDKDG |
| ERR2197925 | YIRARAEVEADAKTGRETIIVHEIPYQVNKARLIEKIAELVKDKRVEGISALRDESDKDG |
| ERR2197927 | YIRARAEVEADAKTGRETIIVHEIPYQVNKARLIEKIAELVKDKRVEGISALRDESDKDG |
| ERR2197929 | YIRARAEVEADAKTGRETIIVHEIPYQVNKARLIEKIAELVKDKRVEGISALRDESDKDG |
| SRR1648149 | YIRARAEVEADAKTGRETIIVHEIPYQVNKARLIEKIAELVKDKRVEGISALRDESDKDG |
| SRR1048299 | YIRARAEVEADAKTGRETIIVHEIPYQVNKARLIEKIAELVKDKRVEGISALRDESDKDG |
| SRR1300677 | YIRARAEVEADAKTGRETIIVHEIPYQVNKARLIEKIAELVKDKRVEGISALRDESDKDG |
| SRR1288356 | YIRARAEVEADAKTGRETIIVHEIPYQVNKARLIEKIAELVKDKRVEGISALRDESDKDG |
| SRR7426190 | YIRARAEVEADAKTGRETIIVHEIPYQVNKARLIEKIAELVKDKRVEGISALRDESDKDG |
| SRR7426192 | YIRARAEVEADAKTGRETIIVHEIPYQVNKARLIEKIAELVKDKRVEGISALRDESDKDG |
| SRR7426193 | YIRARAEVEADAKTGRETIIVHEIPYQVNKARLIEKIAELVKDKRVEGISALRDESDKDG |
| SRR7441832 | YIRARAEVEADAKTGRETIIVHEIPYQVNKARLIEKIAELVKDKRVEGISALRDESDKDG |
| SRR7426179 | YIRARAEVEADAKTGRETIIVHEIPYQVNKARLIEKIAELVKDKRVEGISALRDESDKDG |
| SRR7439238 | YIRARAEVEADAKTGRETIIVHEIPYQVNKARLIEKIAELVKDKRVEGISALRDESDKDG |
| SRR7439244 | YIRARAEVEADAKTGRETIIVHEIPYQVNKARLIEKIAELVKDKRVEGISALRDESDKDG |
| SRR7439259 | YIRARAEVEADAKTGRETIIVHEIPYQVNKARLIEKIAELVKDKRVEGISALRDESDKDG |
| SRR7439260 | YIRARAEVEADAKTGRETIIVHEIPYQVNKARLIEKIAELVKDKRVEGISALRDESDKDG |
| SRR7441786 | YIRARAEVEADAKTGRETIIVHEIPYQVNKARLIEKIAELVKDKRVEGISALRDESDKDG |
| SRR7441797 | YIRARAEVEADAKTGRETIIVHEIPYQVNKARLIEKIAELVKDKRVEGISALRDESDKDG |
| ERR1759093 | YIRARAEVEADAKTGRETIIVHEIPYQVNKARLIEKIAELVKDKRVEGISALRDESDKDG |
| ERR2580275 | YIRARAEVEADAKTGRETIIVHEIPYQVNKARLIEKIAELVKDKRVEGISALRDESDKDG |
| ERR1759204 | YIRARAEVEADAKTGRETIIVHEIPYQVNKARLIEKIAELVKDKRVEGISALRDESDKDG |
| SRR1300699 | YIRARAEVEADAKTGRETIIVHEIPYQVNKARLIEKIAELVKDKRVEGISALRDESDKDG |
| S_0825_17 | YIRARAEVEADAKTGRETIIVHEIPYQVNKARLIEKIAELVKDKRVEGISALRDESDKDG |
| SRR1958215 | YIRARAEVEADAKTGRETIIVHEIPYQVNKARLIEKIAELVKDKRVEGISALRDESDKDG |
| SRR1958540 | YIRARAEVEADAKTGRETIIVHEIPYQVNKARLIEKIAELVKDKRVEGISALRDESDKDG |
| SRR1958636 | YIRARAEVEADAKTGRETIIVHEIPYQVNKARLIEKIAELVKDKRVEGISALRDESDKDG |
| SRR1959422 | YIRARAEVEADAKTGRETIIVHEIPYQVNKARLIEKIAELVKDKRVEGISALRDESDKDG |
| SRR1959427 | YIRARAEVEADAKTGRETIIVHEIPYQVNKARLIEKIAELVKDKRVEGISALRDESDKDG |
| SRR1960226 | YIRARAEVEADAKTGRETIIVHEIPYQVNKARLIEKIAELVKDKRVEGISALRDESDKDG |
| SRR1963498 | YIRARAEVEADAKTGRETIIVHEIPYQVNKARLIEKIAELVKDKRVEGISALRDESDKDG |
| SRR1965947 | YIRARAEVEADAKTGRETIIVHEIPYQVNKARLIEKIAELVKDKRVEGISALRDESDKDG |
| SRR1966125 | YIRARAEVEADAKTGRETIIVHEIPYQVNKARLIEKIAELVKDKRVEGISALRDESDKDG |
| SRR1966330 | YIRARAEVEADAKTGRETIIVHEIPYQVNKARLIEKIAELVKDKRVEGISALRDESDKDG |
| SRR1966565 | YIRARAEVEADAKTGRETIIVHEIPYQVNKARLIEKIAELVKDKRVEGISALRDESDKDG |
| SRR1966864 | YIRARAEVEADAKTGRETIIVHEIPYQVNKARLIEKIAELVKDKRVEGISALRDESDKDG |
| SRR1966989 | YIRARAEVEADAKTGRETIIVHEIPYQVNKARLIEKIAELVKDKRVEGISALRDESDKDG |
| SRR1967688 | YIRARAEVEADAKTGRETIIVHEIPYQVNKARLIEKIAELVKDKRVEGISALRDESDKDG |
| SRR1967733 | YIRARAEVEADAKTGRETIIVHEIPYQVNKARLIEKIAELVKDKRVEGISALRDESDKDG |
| SRR1967746 | YIRARAEVEADAKTGRETIIVHEIPYQVNKARLIEKIAELVKDKRVEGISALRDESDKDG |
| SRR1968341 | YIRARAEVEADAKTGRETIIVHEIPYQVNKARLIEKIAELVKDKRVEGISALRDESDKDG |
| SRR1968456 | YIRARAEVEADAKTGRETIIVHEIPYQVNKARLIEKIAELVKDKRVEGISALRDESDKDG |
| SRR1968465 | YIRARAEVEADAKTGRETIIVHEIPYQVNKARLIEKIAELVKDKRVEGISALRDESDKDG |
| SRR1968761 | YIRARAEVEADAKTGRETIIVHEIPYQVNKARLIEKIAELVKDKRVEGISALRDESDKDG |
| SRR1969047 | YIRARAEVEADAKTGRETIIVHEIPYQVNKARLIEKIAELVKDKRVEGISALRDESDKDG |
| SRR1969255 | YIRARAEVEADAKTGRETIIVHEIPYQVNKARLIEKIAELVKDKRVEGISALRDESDKDG |
| SRR1969412 | YIRARAEVEADAKTGRETIIVHEIPYQVNKARLIEKIAELVKDKRVEGISALRDESDKDG |
| SRR1969524 | YIRARAEVEADAKTGRETIIVHEIPYQVNKARLIEKIAELVKDKRVEGISALRDESDKDG |
| SRR1969584 | YIRARAEVEADAKTGRETIIVHEIPYQVNKARLIEKIAELVKDKRVEGISALRDESDKDG |
| SRR1969648 | YIRARAEVEADAKTGRETIIVHEIPYQVNKARLIEKIAELVKDKRVEGISALRDESDKDG |
| SRR1969804 | YIRARAEVEADAKTGRETIIVHEIPYQVNKARLIEKIAELVKDKRVEGISALRDESDKDG |
| SRR1970221 | YIRARAEVEADAKTGRETIIVHEIPYQVNKARLIEKIAELVKDKRVEGISALRDESDKDG |
| SRR1970268 | YIRARAEVEADAKTGRETIIVHEIPYQVNKARLIEKIAELVKDKRVEGISALRDESDKDG |
| SRR1965862 | YIRARAEVEADAKTGRETIIVHEIPYQVNKARLIEKIAELVKDKRVEGISALRDESDKDG |
| SRR1967363 | YIRARAEVEADAKTGRETIIVHEIPYQVNKARLIEKIAELVKDKRVEGISALRDESDKDG |
| SRR1968276 | YIRARAEVEADAKTGRETIIVHEIPYQVNKARLIEKIAELVKDKRVEGISALRDESDKDG |
| SRR1968967 | YIRARAEVEADAKTGRETIIVHEIPYQVNKARLIEKIAELVKDKRVEGISALRDESDKDG |
| SRR3321531 | YIRARAEVEADAKTGRETIIVHEIPYQVNKARLIEKIAELVKDKRVEGISALRDESDKDG |
| SRR3321883 | YIRARAEVEADAKTGRETIIVHEIPYQVNKARLIEKIAELVKDKRVEGISALRDESDKDG |
| SRR3322413 | YIRARAEVEADAKTGRETIIVHEIPYQVNKARLIEKIAELVKDKRVEGISALRDESDKDG |
| SRR3323012 | YIRARAEVEADAKTGRETIIVHEIPYQVNKARLIEKIAELVKDKRVEGISALRDESDKDG |
| SRR5194289 | YIRARAEVEADAKTGRETIIVHEIPYQVNKARLIEKIAELVKDKRVEGISALRDESDKDG |
| SRR7163798 | YIRARAEVEADAKTGRETIIVHEIPYQVNKARLIEKIAELVKDKRVEGISALRDESDKDG |
| SRR7172610 | YIRARAEVEADAKTGRETIIVHEIPYQVNKARLIEKIAELVKDKRVEGISALRDESDKDG |
| SRR7204568 | YIRARAEVEADAKTGRETIIVHEIPYQVNKARLIEKIAELVKDKRVEGISALRDESDKDG |
| SRR7223230 | YIRARAEVEADAKTGRETIIVHEIPYQVNKARLIEKIAELVKDKRVEGISALRDESDKDG |
| SRR7230675 | YIRARAEVEADAKTGRETIIVHEIPYQVNKARLIEKIAELVKDKRVEGISALRDESDKDG |
| SRR7278056 | YIRARAEVEADAKTGRETIIVHEIPYQVNKARLIEKIAELVKDKRVEGISALRDESDKDG |
| SRR7278086 | YIRARAEVEADAKTGRETIIVHEIPYQVNKARLIEKIAELVKDKRVEGISALRDESDKDG |
| SRR7285841 | YIRARAEVEADAKTGRETIIVHEIPYQVNKARLIEKIAELVKDKRVEGISALRDESDKDG |
| SRR7292625 | YIRARAEVEADAKTGRETIIVHEIPYQVNKARLIEKIAELVKDKRVEGISALRDESDKDG |
| SRR7292665 | YIRARAEVEADAKTGRETIIVHEIPYQVNKARLIEKIAELVKDKRVEGISALRDESDKDG |
| SRR7297965 | YIRARAEVEADAKTGRETIIVHEIPYQVNKARLIEKIAELVKDKRVEGISALRDESDKDG |
| SRR7350726 | YIRARAEVEADAKTGRETIIVHEIPYQVNKARLIEKIAELVKDKRVEGISALRDESDKDG |
| SRR7410328 | YIRARAEVEADAKTGRETIIVHEIPYQVNKARLIEKIAELVKDKRVEGISALRDESDKDG |
| SRR7474665 | YIRARAEVEADAKTGRETIIVHEIPYQVNKARLIEKIAELVKDKRVEGISALRDESDKDG |
| SRR7523184 | YIRARAEVEADAKTGRETIIVHEIPYQVNKARLIEKIAELVKDKRVEGISALRDESDKDG |
| SRR7187264 | YIRARAEVEADAKTGRETIIVHEIPYQVNKARLIEKIAELVKDKRVEGISALRDESDKDG |
| SRR7204445 | YIRARAEVEADAKTGRETIIVHEIPYQVNKARLIEKIAELVKDKRVEGISALRDESDKDG |
| SRR7285641 | YIRARAEVEADAKTGRETIIVHEIPYQVNKARLIEKIAELVKDKRVEGISALRDESDKDG |
| SRR7286695 | YIRARAEVEADAKTGRETIIVHEIPYQVNKARLIEKIAELVKDKRVEGISALRDESDKDG |
| SRR7286705 | YIRARAEVEADAKTGRETIIVHEIPYQVNKARLIEKIAELVKDKRVEGISALRDESDKDG |
| SRR7292931 | YIRARAEVEADAKTGRETIIVHEIPYQVNKARLIEKIAELVKDKRVEGISALRDESDKDG |
| SRR7310349 | YIRARAEVEADAKTGRETIIVHEIPYQVNKARLIEKIAELVKDKRVEGISALRDESDKDG |
| SRR7351616 | YIRARAEVEADAKTGRETIIVHEIPYQVNKARLIEKIAELVKDKRVEGISALRDESDKDG |
| SRR7414818 | YIRARAEVEADAKTGRETIIVHEIPYQVNKARLIEKIAELVKDKRVEGISALRDESDKDG |
| SRR7426480 | YIRARAEVEADAKTGRETIIVHEIPYQVNKARLIEKIAELVKDKRVEGISALRDESDKDG |
| SRR5584105 | YIRARAEVEADAKTGRETIIVHEIPYQVNKARLIEKIAELVKDKRVEGISALRDESDKDG |
| SRR5584565 | YIRARAEVEADAKTGRETIIVHEIPYQVNKARLIEKIAELVKDKRVEGISALRDESDKDG |
| SRR5584614 | YIRARAEVEADAKTGRETIIVHEIPYQVNKARLIEKIAELVKDKRVEGISALRDESDKDG |
| SRR5631543 | YIRARAEVEADAKTGRETIIVHEIPYQVNKARLIEKIAELVKDKRVEGISALRDESDKDG |
| SRR5631553 | YIRARAEVEADAKTGRETIIVHEIPYQVNKARLIEKIAELVKDKRVEGISALRDESDKDG |
| SRR7123196 | YIRARAEVEADAKTGRETIIVHEIPYQVNKARLIEKIAELVKDKRVEGISALRDESDKDG |
| SRR7163819 | YIRARAEVEADAKTGRETIIVHEIPYQVNKARLIEKIAELVKDKRVEGISALRDESDKDG |
| SRR7163920 | YIRARAEVEADAKTGRETIIVHEIPYQVNKARLIEKIAELVKDKRVEGISALRDESDKDG |
| SRR7209528 | YIRARAEVEADAKTGRETIIVHEIPYQVNKARLIEKIAELVKDKRVEGISALRDESDKDG |
| SRR7249868 | YIRARAEVEADAKTGRETIIVHEIPYQVNKARLIEKIAELVKDKRVEGISALRDESDKDG |
| SRR7278088 | YIRARAEVEADAKTGRETIIVHEIPYQVNKARLIEKIAELVKDKRVEGISALRDESDKDG |
| SRR7285788 | YIRARAEVEADAKTGRETIIVHEIPYQVNKARLIEKIAELVKDKRVEGISALRDESDKDG |
| SRR7286789 | YIRARAEVEADAKTGRETIIVHEIPYQVNKARLIEKIAELVKDKRVEGISALRDESDKDG |
| SRR7286886 | YIRARAEVEADAKTGRETIIVHEIPYQVNKARLIEKIAELVKDKRVEGISALRDESDKDG |
| SRR7310632 | YIRARAEVEADAKTGRETIIVHEIPYQVNKARLIEKIAELVKDKRVEGISALRDESDKDG |
| SRR7350631 | YIRARAEVEADAKTGRETIIVHEIPYQVNKARLIEKIAELVKDKRVEGISALRDESDKDG |
| SRR7458741 | YIRARAEVEADAKTGRETIIVHEIPYQVNKARLIEKIAELVKDKRVEGISALRDESDKDG |
| SRR7480280 | YIRARAEVEADAKTGRETIIVHEIPYQVNKARLIEKIAELVKDKRVEGISALRDESDKDG |
| SRR7523660 | YIRARAEVEADAKTGRETIIVHEIPYQVNKARLIEKIAELVKDKRVEGISALRDESDKDG |
| SRR7523775 | YIRARAEVEADAKTGRETIIVHEIPYQVNKARLIEKIAELVKDKRVEGISALRDESDKDG |
| SRR7251101 | YIRARAEVEADAKTGRETIIVHEIPYQVNKARLIEKIAELVKDKRVEGISALRDESDKDG |
| SRR7284299 | YIRARAEVEADAKTGRETIIVHEIPYQVNKARLIEKIAELVKDKRVEGISALRDESDKDG |
| SRR7285738 | YIRARAEVEADAKTGRETIIVHEIPYQVNKARLIEKIAELVKDKRVEGISALRDESDKDG |
| SRR7310640 | YIRARAEVEADAKTGRETIIVHEIPYQVNKARLIEKIAELVKDKRVEGISALRDESDKDG |
| SRR7349159 | YIRARAEVEADAKTGRETIIVHEIPYQVNKARLIEKIAELVKDKRVEGISALRDESDKDG |
| SRR7474873 | YIRARAEVEADAKTGRETIIVHEIPYQVNKARLIEKIAELVKDKRVEGISALRDESDKDG |
| SRR7495689 | YIRARAEVEADAKTGRETIIVHEIPYQVNKARLIEKIAELVKDKRVEGISALRDESDKDG |
| SRR7495752 | YIRARAEVEADAKTGRETIIVHEIPYQVNKARLIEKIAELVKDKRVEGISALRDESDKDG |
| ----------------------------------------------------------------------------- | |
| S16BD08730 | MRIVIEVKRDAVGEVVLNNLYSQTQLQVSFGINMVALHHGQPKIMNLKDIISAFVRHRRE |
| S18BD00684 | MRIVIEVKRDAVGEVVLNNLYSQTQLQVSFGINMVALHHGQPKIMNLKDIISAFVRHRRE |
| S18BD03994 | MRIVIEVKRDAVGEVVLNNLYSQTQLQVSFGINMVALHHGQPKIMNLKDIISAFVRHRRE |
| S18BD05011 | MRIVIEVKRDAVGEVVLNNLYSQTQLQVSFGINMVALHHGQPKIMNLKDIISAFVRHRRE |
| RKI_16-03723 | MRIVIEVKRDAVGEVVLNNLYSQTQLQVSFGINMVALHHGQPKIMNLKDIISAFVRHRRE |
| RKI_16-04315 | MRIVIEVKRDAVGEVVLNNLYSQTQLQVSFGINMVALHHGQPKIMNLKDIISAFVRHRRE |
| RKI_17-02304 | MRIVIEVKRDAVGEVVLNNLYSQTQLQVSFGINMVALHHGQPKIMNLKDIISAFVRHRRE |
| RKI_17-02411 | MRIVIEVKRDAVGEVVLNNLYSQTQLQVSFGINMVALHHGQPKIMNLKDIISAFVRHRRE |
| RKI_17-02757 | MRIVIEVKRDAVGEVVLNNLYSQTQLQVSFGINMVALHHGQPKIMNLKDIISAFVRHRRE |
| RKI_17-04797 | MRIVIEVKRDAVGEVVLNNLYSQTQLQVSFGINMVALHHGQPKIMNLKDIISAFVRHRRE |
| RKI_17-06869 | MRIVIEVKRDAVGEVVLNNLYSQTQLQVSFGINMVALHHGQPKIMNLKDIISAFVRHRRE |
| ERR2580277 | MRIVIEVKRDAVGEVVLNNLYSQTQLQVSFGINMVALHHGQPKIMNLKDIISAFVRHRRE |
| ERR2580276 | MRIVIEVKRDAVGEVVLNNLYSQTQLQVSFGINMVALHHGQPKIMNLKDIISAFVRHRRE |
| ERR2580273 | MRIVIEVKRDAVGEVVLNNLYSQTQLQVSFGINMVALHHGQPKIMNLKDIISAFVRHRRE |
| ERR2580274 | MRIVIEVKRDAVGEVVLNNLYSQTQLQVSFGINMVALHHGQPKIMNLKDIISAFVRHRRE |
| ERR2173656 | MRIVIEVKRDAVGEVVLNNLYSQTQLQVSFGINMVALHHGQPKIMNLKDIISAFVRHRRE |
| 17041676 | MRIVIEVKRDAVGEVVLNNLYSQTQLQVSFGINMVALHHGQPKIMNLKDIISAFVRHRRE |
| MT16-000061 | MRIVIEVKRDAVGEVVLNNLYSQTQLQVSFGINMVALHHGQPKIMNLKDIISAFVRHRRE |
| MT16-019416 | MRIVIEVKRDAVGEVVLNNLYSQTQLQVSFGINMVALHHGQPKIMNLKDIISAFVRHRRE |
| MT16-027865 | MRIVIEVKRDAVGEVVLNNLYSQTQLQVSFGINMVALHHGQPKIMNLKDIISAFVRHRRE |
| MT16-031693 | MRIVIEVKRDAVGEVVLNNLYSQTQLQVSFGINMVALHHGQPKIMNLKDIISAFVRHRRE |
| MT16-040253 | MRIVIEVKRDAVGEVVLNNLYSQTQLQVSFGINMVALHHGQPKIMNLKDIISAFVRHRRE |
| MT16-045379 | MRIVIEVKRDAVGEVVLNNLYSQTQLQVSFGINMVALHHGQPKIMNLKDIISAFVRHRRE |
| MT16-442728 | MRIVIEVKRDAVGEVVLNNLYSQTQLQVSFGINMVALHHGQPKIMNLKDIISAFVRHRRE |
| MT16-462857 | MRIVIEVKRDAVGEVVLNNLYSQTQLQVSFGINMVALHHGQPKIMNLKDIISAFVRHRRE |
| MT16-480196 | MRIVIEVKRDAVGEVVLNNLYSQTQLQVSFGINMVALHHGQPKIMNLKDIISAFVRHRRE |
| MT16-861555 | MRIVIEVKRDAVGEVVLNNLYSQTQLQVSFGINMVALHHGQPKIMNLKDIISAFVRHRRE |
| MT17-076833 | MRIVIEVKRDAVGEVVLNNLYSQTQLQVSFGINMVALHHGQPKIMNLKDIISAFVRHRRE |
| MT17-110677 | MRIVIEVKRDAVGEVVLNNLYSQTQLQVSFGINMVALHHGQPKIMNLKDIISAFVRHRRE |
| MT17-131730 | MRIVIEVKRDAVGEVVLNNLYSQTQLQVSFGINMVALHHGQPKIMNLKDIISAFVRHRRE |
| MT17-140890 | MRIVIEVKRDAVGEVVLNNLYSQTQLQVSFGINMVALHHGQPKIMNLKDIISAFVRHRRE |
| MT17-141840 | MRIVIEVKRDAVGEVVLNNLYSQTQLQVSFGINMVALHHGQPKIMNLKDIISAFVRHRRE |
| MT17-152488 | MRIVIEVKRDAVGEVVLNNLYSQTQLQVSFGINMVALHHGQPKIMNLKDIISAFVRHRRE |
| MT17-157311 | MRIVIEVKRDAVGEVVLNNLYSQTQLQVSFGINMVALHHGQPKIMNLKDIISAFVRHRRE |
| MT17-161645 | MRIVIEVKRDAVGEVVLNNLYSQTQLQVSFGINMVALHHGQPKIMNLKDIISAFVRHRRE |
| MT17-167951 | MRIVIEVKRDAVGEVVLNNLYSQTQLQVSFGINMVALHHGQPKIMNLKDIISAFVRHRRE |
| MT18-217732 | MRIVIEVKRDAVGEVVLNNLYSQTQLQVSFGINMVALHHGQPKIMNLKDIISAFVRHRRE |
| MT18-252580 | MRIVIEVKRDAVGEVVLNNLYSQTQLQVSFGINMVALHHGQPKIMNLKDIISAFVRHRRE |
| RIVM_H_2009-01 | MRIVIEVKRDAVGEVVLNNLYSQTQLQVSFGINMVALHHGQPKIMNLKDIISAFVRHRRE |
| RIVM_H_2010-01 | MRIVIEVKRDAVGEVVLNNLYSQTQLQVSFGINMVALHHGQPKIMNLKDIISAFVRHRRE |
| RIVM_H_2010-02 | MRIVIEVKRDAVGEVVLNNLYSQTQLQVSFGINMVALHHGQPKIMNLKDIISAFVRHRRE |
| RIVM_H_2011-01 | MRIVIEVKRDAVGEVVLNNLYSQTQLQVSFGINMVALHHGQPKIMNLKDIISAFVRHRRE |
| RIVM_H_2011-02 | MRIVIEVKRDAVGEVVLNNLYSQTQLQVSFGINMVALHHGQPKIMNLKDIISAFVRHRRE |
| RIVM_H_2011-03 | MRIVIEVKRDAVGEVVLNNLYSQTQLQVSFGINMVALHHGQPKIMNLKDIISAFVRHRRE |
| RIVM_H_2013-01 | MRIVIEVKRDAVGEVVLNNLYSQTQLQVSFGINMVALHHGQPKIMNLKDIISAFVRHRRE |
| RIVM_H_2013-02 | MRIVIEVKRDAVGEVVLNNLYSQTQLQVSFGINMVALHHGQPKIMNLKDIISAFVRHRRE |
| RIVM_H_2014-01 | MRIVIEVKRDAVGEVVLNNLYSQTQLQVSFGINMVALHHGQPKIMNLKDIISAFVRHRRE |
| RIVM_H_2014-02 | MRIVIEVKRDAVGEVVLNNLYSQTQLQVSFGINMVALHHGQPKIMNLKDIISAFVRHRRE |
| RIVM_H_2016-01 | MRIVIEVKRDAVGEVVLNNLYSQTQLQVSFGINMVALHHGQPKIMNLKDIISAFVRHRRE |
| RIVM_H_2016-02 | MRIVIEVKRDAVGEVVLNNLYSQTQLQVSFGINMVALHHGQPKIMNLKDIISAFVRHRRE |
| RIVM_H_2016-03 | MRIVIEVKRDAVGEVVLNNLYSQTQLQVSFGINMVALHHGQPKIMNLKDIISAFVRHRRE |
| RIVM_H_2016-04 | MRIVIEVKRDAVGEVVLNNLYSQTQLQVSFGINMVALHHGQPKIMNLKDIISAFVRHRRE |
| RIVM_H_2016-05 | MRIVIEVKRDAVGEVVLNNLYSQTQLQVSFGINMVALHHGQPKIMNLKDIISAFVRHRRE |
| RIVM_H_2016-06 | MRIVIEVKRDAVGEVVLNNLYSQTQLQVSFGINMVALHHGQPKIMNLKDIISAFVRHRRE |
| RIVM_H_2016-07 | MRIVIEVKRDAVGEVVLNNLYSQTQLQVSFGINMVALHHGQPKIMNLKDIISAFVRHRRE |
| RIVM_H_2016-08 | MRIVIEVKRDAVGEVVLNNLYSQTQLQVSFGINMVALHHGQPKIMNLKDIISAFVRHRRE |
| RIVM_H_2016-09 | MRIVIEVKRDAVGEVVLNNLYSQTQLQVSFGINMVALHHGQPKIMNLKDIISAFVRHRRE |
| RIVM_H_2016-10 | MRIVIEVKRDAVGEVVLNNLYSQTQLQVSFGINMVALHHGQPKIMNLKDIISAFVRHRRE |
| RIVM_H_2016-11 | MRIVIEVKRDAVGEVVLNNLYSQTQLQVSFGINMVALHHGQPKIMNLKDIISAFVRHRRE |
| RIVM_H_2016-12 | MRIVIEVKRDAVGEVVLNNLYSQTQLQVSFGINMVALHHGQPKIMNLKDIISAFVRHRRE |
| RIVM_H_2016-13 | MRIVIEVKRDAVGEVVLNNLYSQTQLQVSFGINMVALHHGQPKIMNLKDIISAFVRHRRE |
| RIVM_H_2016-14 | MRIVIEVKRDAVGEVVLNNLYSQTQLQVSFGINMVALHHGQPKIMNLKDIISAFVRHRRE |
| RIVM_H_2016-15 | MRIVIEVKRDAVGEVVLNNLYSQTQLQVSFGINMVALHHGQPKIMNLKDIISAFVRHRRE |
| RIVM_H_2017-01 | MRIVIEVKRDAVGEVVLNNLYSQTQLQVSFGINMVALHHGQPKIMNLKDIISAFVRHRRE |
| RIVM_H_2017-02 | MRIVIEVKRDAVGEVVLNNLYSQTQLQVSFGINMVALHHGQPKIMNLKDIISAFVRHRRE |
| RIVM_H_2017-03 | MRIVIEVKRDAVGEVVLNNLYSQTQLQVSFGINMVALHHGQPKIMNLKDIISAFVRHRRE |
| RIVM_H_2017-04 | MRIVIEVKRDAVGEVVLNNLYSQTQLQVSFGINMVALHHGQPKIMNLKDIISAFVRHRRE |
| RIVM_H_2017-05 | MRIVIEVKRDAVGEVVLNNLYSQTQLQVSFGINMVALHHGQPKIMNLKDIISAFVRHRRE |
| RIVM_H_2017-06 | MRIVIEVKRDAVGEVVLNNLYSQTQLQVSFGINMVALHHGQPKIMNLKDIISAFVRHRRE |
| RIVM_H_2017-07 | MRIVIEVKRDAVGEVVLNNLYSQTQLQVSFGINMVALHHGQPKIMNLKDIISAFVRHRRE |
| RIVM_H_2017-08 | MRIVIEVKRDAVGEVVLNNLYSQTQLQVSFGINMVALHHGQPKIMNLKDIISAFVRHRRE |
| RIVM_H_2017-09 | MRIVIEVKRDAVGEVVLNNLYSQTQLQVSFGINMVALHHGQPKIMNLKDIISAFVRHRRE |
| RIVM_H_2017-10 | MRIVIEVKRDAVGEVVLNNLYSQTQLQVSFGINMVALHHGQPKIMNLKDIISAFVRHRRE |
| RIVM_H_2017-11 | MRIVIEVKRDAVGEVVLNNLYSQTQLQVSFGINMVALHHGQPKIMNLKDIISAFVRHRRE |
| RIVM_H_2017-12 | MRIVIEVKRDAVGEVVLNNLYSQTQLQVSFGINMVALHHGQPKIMNLKDIISAFVRHRRE |
| RIVM_H_2017-13 | MRIVIEVKRDAVGEVVLNNLYSQTQLQVSFGINMVALHHGQPKIMNLKDIISAFVRHRRE |
| RIVM_H_2017-14 | MRIVIEVKRDAVGEVVLNNLYSQTQLQVSFGINMVALHHGQPKIMNLKDIISAFVRHRRE |
| RIVM_H_2017-15 | MRIVIEVKRDAVGEVVLNNLYSQTQLQVSFGINMVALHHGQPKIMNLKDIISAFVRHRRE |
| RIVM_H_2017-16 | MRIVIEVKRDAVGEVVLNNLYSQTQLQVSFGINMVALHHGQPKIMNLKDIISAFVRHRRE |
| RIVM_H_2017-17 | MRIVIEVKRDAVGEVVLNNLYSQTQLQVSFGINMVALHHGQPKIMNLKDIISAFVRHRRE |
| RIVM_H_2017-18 | MRIVIEVKRDAVGEVVLNNLYSQTQLQVSFGINMVALHHGQPKIMNLKDIISAFVRHRRE |
| RIVM_H_2017-19 | MRIVIEVKRDAVGEVVLNNLYSQTQLQVSFGINMVALHHGQPKIMNLKDIISAFVRHRRE |
| 15EP001483 | MRIVIEVKRDAVGEVVLNNLYSQTQLQVSFGINMVALHHGQPKIMNLKDIISAFVRHRRE |
| 17EP002363 | MRIVIEVKRDAVGEVVLNNLYSQTQLQVSFGINMVALHHGQPKIMNLKDIISAFVRHRRE |
| S_0812_17 | MRIVIEVKRDAVGEVVLNNLYSQTQLQVSFGINMVALHHGQPKIMNLKDIISAFVRHRRE |
| SRR1957844 | MRIVIEVKRDAVGEVVLNNLYSQTQLQVSFGINMVALHHGQPKIMNLKDIISAFVRHRRE |
| SRR1958654 | MRIVIEVKRDAVGEVVLNNLYSQTQLQVSFGINMVALHHGQPKIMNLKDIISAFVRHRRE |
| SRR1965077 | MRIVIEVKRDAVGEVVLNNLYSQTQLQVSFGINMVALHHGQPKIMNLKDIISAFVRHRRE |
| SRR1966369 | MRIVIEVKRDAVGEVVLNNLYSQTQLQVSFGINMVALHHGQPKIMNLKDIISAFVRHRRE |
| SRR1967117 | MRIVIEVKRDAVGEVVLNNLYSQTQLQVSFGINMVALHHGQPKIMNLKDIISAFVRHRRE |
| SRR1967922 | MRIVIEVKRDAVGEVVLNNLYSQTQLQVSFGINMVALHHGQPKIMNLKDIISAFVRHRRE |
| SRR8704720 | MRIVIEVKRDAVGEVVLNNLYSQTQLQVSFGINMVALHHGQPKIMNLKDIISAFVRHRRE |
| SRR7216071 | MRIVIEVKRDAVGEVVLNNLYSQTQLQVSFGINMVALHHGQPKIMNLKDIISAFVRHRRE |
| SRR7349175 | MRIVIEVKRDAVGEVVLNNLYSQTQLQVSFGINMVALHHGQPKIMNLKDIISAFVRHRRE |
| SRR7523148 | MRIVIEVKRDAVGEVVLNNLYSQTQLQVSFGINMVALHHGQPKIMNLKDIISAFVRHRRE |
| SRR7523854 | MRIVIEVKRDAVGEVVLNNLYSQTQLQVSFGINMVALHHGQPKIMNLKDIISAFVRHRRE |
| 313865 | MRIVIEVKRDAVGEVVLNNLYSQTQLQVSFGINMVALHHGQPKIMNLKDIISAFVRHRRE |
| SRR7277793 | MRIVIEVKRDAVGEVVLNNLYSQTQLQVSFGINMVALHHGQPKIMNLKDIISAFVRHRRE |
| SRR7343877 | MRIVIEVKRDAVGEVVLNNLYSQTQLQVSFGINMVALHHGQPKIMNLKDIISAFVRHRRE |
| SRR7351477 | MRIVIEVKRDAVGEVVLNNLYSQTQLQVSFGINMVALHHGQPKIMNLKDIISAFVRHRRE |
| SRR5583183 | MRIVIEVKRDAVGEVVLNNLYSQTQLQVSFGINMVALHHGQPKIMNLKDIISAFVRHRRE |
| SRR5585240 | MRIVIEVKRDAVGEVVLNNLYSQTQLQVSFGINMVALHHGQPKIMNLKDIISAFVRHRRE |
| SRR7284317 | MRIVIEVKRDAVGEVVLNNLYSQTQLQVSFGINMVALHHGQPKIMNLKDIISAFVRHRRE |
| SRR7299161 | MRIVIEVKRDAVGEVVLNNLYSQTQLQVSFGINMVALHHGQPKIMNLKDIISAFVRHRRE |
| SRR7401730 | MRIVIEVKRDAVGEVVLNNLYSQTQLQVSFGINMVALHHGQPKIMNLKDIISAFVRHRRE |
| SRR7469092 | MRIVIEVKRDAVGEVVLNNLYSQTQLQVSFGINMVALHHGQPKIMNLKDIISAFVRHRRE |
| SRR7879556 | MRIVIEVKRDAVGEVVLNNLYSQTQLQVSFGINMVALHHGQPKIMNLKDIISAFVRHRRE |
| SRR8526100 | MRIVIEVKRDAVGEVVLNNLYSQTQLQVSFGINMVALHHGQPKIMNLKDIISAFVRHRRE |
| SRR8553991 | MRIVIEVKRDAVGEVVLNNLYSQTQLQVSFGINMVALHHGQPKIMNLKDIISAFVRHRRE |
| SRR7842487 | MRIVIEVKRDAVGEVVLNNLYSQTQLQVSFGINMVALHHGQPKIMNLKDIISAFVRHRRE |
| SRR8054524 | MRIVIEVKRDAVGEVVLNNLYSQTQLQVSFGINMVALHHGQPKIMNLKDIISAFVRHRRE |
| SRR8054525 | MRIVIEVKRDAVGEVVLNNLYSQTQLQVSFGINMVALHHGQPKIMNLKDIISAFVRHRRE |
| SRR8524733 | MRIVIEVKRDAVGEVVLNNLYSQTQLQVSFGINMVALHHGQPKIMNLKDIISAFVRHRRE |
| SRR4093291 | MRIVIEVKRDAVGEVVLNNLYSQTQLQVSFGINMVALHHGQPKIMNLKDIISAFVRHRRE |
| SRR4245549 | MRIVIEVKRDAVGEVVLNNLYSQTQLQVSFGINMVALHHGQPKIMNLKDIISAFVRHRRE |
| SRR3057154 | MRIVIEVKRDAVGEVVLNNLYSQTQLQVSFGINMVALHHGQPKIMNLKDIISAFVRHRRE |
| SRR1726150 | MRIVIEVKRDAVGEVVLNNLYSQTQLQVSFGINMVALHHGQPKIMNLKDIISAFVRHRRE |
| SRR1996141 | MRIVIEVKRDAVGEVVLNNLYSQTQLQVSFGINMVALHHGQPKIMNLKDIISAFVRHRRE |
| SRR1107842 | MRIVIEVKRDAVGEVVLNNLYSQTQLQVSFGINMVALHHGQPKIMNLKDIISAFVRHRRE |
| SRR1157587 | MRIVIEVKRDAVGEVVLNNLYSQTQLQVSFGINMVALHHGQPKIMNLKDIISAFVRHRRE |
| SRR3027706 | MRIVIEVKRDAVGEVVLNNLYSQTQLQVSFGINMVALHHGQPKIMNLKDIISAFVRHRRE |
| SRR3027707 | MRIVIEVKRDAVGEVVLNNLYSQTQLQVSFGINMVALHHGQPKIMNLKDIISAFVRHRRE |
| SRR3027708 | MRIVIEVKRDAVGEVVLNNLYSQTQLQVSFGINMVALHHGQPKIMNLKDIISAFVRHRRE |
| SRR3027710 | MRIVIEVKRDAVGEVVLNNLYSQTQLQVSFGINMVALHHGQPKIMNLKDIISAFVRHRRE |
| SRR3027711 | MRIVIEVKRDAVGEVVLNNLYSQTQLQVSFGINMVALHHGQPKIMNLKDIISAFVRHRRE |
| SRR3027716 | MRIVIEVKRDAVGEVVLNNLYSQTQLQVSFGINMVALHHGQPKIMNLKDIISAFVRHRRE |
| SRR3027717 | MRIVIEVKRDAVGEVVLNNLYSQTQLQVSFGINMVALHHGQPKIMNLKDIISAFVRHRRE |
| SRR3027719 | MRIVIEVKRDAVGEVVLNNLYSQTQLQVSFGINMVALHHGQPKIMNLKDIISAFVRHRRE |
| SRR3027721 | MRIVIEVKRDAVGEVVLNNLYSQTQLQVSFGINMVALHHGQPKIMNLKDIISAFVRHRRE |
| SRR3027723 | MRIVIEVKRDAVGEVVLNNLYSQTQLQVSFGINMVALHHGQPKIMNLKDIISAFVRHRRE |
| SRR3115978 | MRIVIEVKRDAVGEVVLNNLYSQTQLQVSFGINMVALHHGQPKIMNLKDIISAFVRHRRE |
| SRR2534093 | MRIVIEVKRDAVGEVVLNNLYSQTQLQVSFGINMVALHHGQPKIMNLKDIISAFVRHRRE |
| SRR2534094 | MRIVIEVKRDAVGEVVLNNLYSQTQLQVSFGINMVALHHGQPKIMNLKDIISAFVRHRRE |
| SRR2534095 | MRIVIEVKRDAVGEVVLNNLYSQTQLQVSFGINMVALHHGQPKIMNLKDIISAFVRHRRE |
| SRR2534108 | MRIVIEVKRDAVGEVVLNNLYSQTQLQVSFGINMVALHHGQPKIMNLKDIISAFVRHRRE |
| SRR1106464 | MRIVIEVKRDAVGEVVLNNLYSQTQLQVSFGINMVALHHGQPKIMNLKDIISAFVRHRRE |
| SRR1106463 | MRIVIEVKRDAVGEVVLNNLYSQTQLQVSFGINMVALHHGQPKIMNLKDIISAFVRHRRE |
| SRR6949610 | MRIVIEVKRDAVGEVVLNNLYSQTQLQVSFGINMVALHHGQPKIMNLKDIISAFVRHRRE |
| SRR6950452 | MRIVIEVKRDAVGEVVLNNLYSQTQLQVSFGINMVALHHGQPKIMNLKDIISAFVRHRRE |
| ERR2019831 | MRIVIEVKRDAVGEVVLNNLYSQTQLQVSFGINMVALHHGQPKIMNLKDIISAFVRHRRE |
| SRR2085693 | MRIVIEVKRDAVGEVVLNNLYSQTQLQVSFGINMVALHHGQPKIMNLKDIISAFVRHRRE |
| SRR2086898 | MRIVIEVKRDAVGEVVLNNLYSQTQLQVSFGINMVALHHGQPKIMNLKDIISAFVRHRRE |
| SRR2175312 | MRIVIEVKRDAVGEVVLNNLYSQTQLQVSFGINMVALHHGQPKIMNLKDIISAFVRHRRE |
| SRR2175360 | MRIVIEVKRDAVGEVVLNNLYSQTQLQVSFGINMVALHHGQPKIMNLKDIISAFVRHRRE |
| SRR5231997 | MRIVIEVKRDAVGEVVLNNLYSQTQLQVSFGINMVALHHGQPKIMNLKDIISAFVRHRRE |
| SRR5232003 | MRIVIEVKRDAVGEVVLNNLYSQTQLQVSFGINMVALHHGQPKIMNLKDIISAFVRHRRE |
| SRR5232015 | MRIVIEVKRDAVGEVVLNNLYSQTQLQVSFGINMVALHHGQPKIMNLKDIISAFVRHRRE |
| SRR949434 | MRIVIEVKRDAVGEVVLNNLYSQTQLQVSFGINMVALHHGQPKIMNLKDIISAFVRHRRE |
| SRR3216575 | MRIVIEVKRDAVGEVVLNNLYSQTQLQVSFGINMVALHHGQPKIMNLKDIISAFVRHRRE |
| SRR5205342 | MRIVIEVKRDAVGEVVLNNLYSQTQLQVSFGINMVALHHGQPKIMNLKDIISAFVRHRRE |
| SRR1501669 | MRIVIEVKRDAVGEVVLNNLYSQTQLQVSFGINMVALHHGQPKIMNLKDIISAFVRHRRE |
| SRR5209740 | MRIVIEVKRDAVGEVVLNNLYSQTQLQVSFGINMVALHHGQPKIMNLKDIISAFVRHRRE |
| SRR3240355 | MRIVIEVKRDAVGEVVLNNLYSQTQLQVSFGINMVALHHGQPKIMNLKDIISAFVRHRRE |
| SRR3392777 | MRIVIEVKRDAVGEVVLNNLYSQTQLQVSFGINMVALHHGQPKIMNLKDIISAFVRHRRE |
| SRR3593671 | MRIVIEVKRDAVGEVVLNNLYSQTQLQVSFGINMVALHHGQPKIMNLKDIISAFVRHRRE |
| SRR5413290 | MRIVIEVKRDAVGEVVLNNLYSQTQLQVSFGINMVALHHGQPKIMNLKDIISAFVRHRRE |
| SRR5590269 | MRIVIEVKRDAVGEVVLNNLYSQTQLQVSFGINMVALHHGQPKIMNLKDIISAFVRHRRE |
| SRR5812103 | MRIVIEVKRDAVGEVVLNNLYSQTQLQVSFGINMVALHHGQPKIMNLKDIISAFVRHRRE |
| SRR2830941 | MRIVIEVKRDAVGEVVLNNLYSQTQLQVSFGINMVALHHGQPKIMNLKDIISAFVRHRRE |
| SRR2830966 | MRIVIEVKRDAVGEVVLNNLYSQTQLQVSFGINMVALHHGQPKIMNLKDIISAFVRHRRE |
| SRR3137270 | MRIVIEVKRDAVGEVVLNNLYSQTQLQVSFGINMVALHHGQPKIMNLKDIISAFVRHRRE |
| SRR3137271 | MRIVIEVKRDAVGEVVLNNLYSQTQLQVSFGINMVALHHGQPKIMNLKDIISAFVRHRRE |
| ERR526807 | MRIVIEVKRDAVGEVVLNNLYSQTQLQVSFGINMVALHHGQPKIMNLKDIISAFVRHRRE |
| ERR2197922 | MRIVIEVKRDAVGEVVLNNLYSQTQLQVSFGINMVALHHGQPKIMNLKDIISAFVRHRRE |
| ERR2197923 | MRIVIEVKRDAVGEVVLNNLYSQTQLQVSFGINMVALHHGQPKIMNLKDIISAFVRHRRE |
| ERR2197924 | MRIVIEVKRDAVGEVVLNNLYSQTQLQVSFGINMVALHHGQPKIMNLKDIISAFVRHRRE |
| ERR2197925 | MRIVIEVKRDAVGEVVLNNLYSQTQLQVSFGINMVALHHGQPKIMNLKDIISAFVRHRRE |
| ERR2197927 | MRIVIEVKRDAVGEVVLNNLYSQTQLQVSFGINMVALHHGQPKIMNLKDIISAFVRHRRE |
| ERR2197929 | MRIVIEVKRDAVGEVVLNNLYSQTQLQVSFGINMVALHHGQPKIMNLKDIISAFVRHRRE |
| SRR1648149 | MRIVIEVKRDAVGEVVLNNLYSQTQLQVSFGINMVALHHGQPKIMNLKDIISAFVRHRRE |
| SRR1048299 | MRIVIEVKRDAVGEVVLNNLYSQTQLQVSFGINMVALHHGQPKIMNLKDIISAFVRHRRE |
| SRR1300677 | MRIVIEVKRDAVGEVVLNNLYSQTQLQVSFGINMVALHHGQPKIMNLKDIISAFVRHRRE |
| SRR1288356 | MRIVIEVKRDAVGEVVLNNLYSQTQLQVSFGINMVALHHGQPKIMNLKDIISAFVRHRRE |
| SRR7426190 | MRIVIEVKRDAVGEVVLNNLYSQTQLQVSFGINMVALHHGQPKIMNLKDIISAFVRHRRE |
| SRR7426192 | MRIVIEVKRDAVGEVVLNNLYSQTQLQVSFGINMVALHHGQPKIMNLKDIISAFVRHRRE |
| SRR7426193 | MRIVIEVKRDAVGEVVLNNLYSQTQLQVSFGINMVALHHGQPKIMNLKDIISAFVRHRRE |
| SRR7441832 | MRIVIEVKRDAVGEVVLNNLYSQTQLQVSFGINMVALHHGQPKIMNLKDIISAFVRHRRE |
| SRR7426179 | MRIVIEVKRDAVGEVVLNNLYSQTQLQVSFGINMVALHHGQPKIMNLKDIISAFVRHRRE |
| SRR7439238 | MRIVIEVKRDAVGEVVLNNLYSQTQLQVSFGINMVALHHGQPKIMNLKDIISAFVRHRRE |
| SRR7439244 | MRIVIEVKRDAVGEVVLNNLYSQTQLQVSFGINMVALHHGQPKIMNLKDIISAFVRHRRE |
| SRR7439259 | MRIVIEVKRDAVGEVVLNNLYSQTQLQVSFGINMVALHHGQPKIMNLKDIISAFVRHRRE |
| SRR7439260 | MRIVIEVKRDAVGEVVLNNLYSQTQLQVSFGINMVALHHGQPKIMNLKDIISAFVRHRRE |
| SRR7441786 | MRIVIEVKRDAVGEVVLNNLYSQTQLQVSFGINMVALHHGQPKIMNLKDIISAFVRHRRE |
| SRR7441797 | MRIVIEVKRDAVGEVVLNNLYSQTQLQVSFGINMVALHHGQPKIMNLKDIISAFVRHRRE |
| ERR1759093 | MRIVIEVKRDAVGEVVLNNLYSQTQLQVSFGINMVALHHGQPKIMNLKDIISAFVRHRRE |
| ERR2580275 | MRIVIEVKRDAVGEVVLNNLYSQTQLQVSFGINMVALHHGQPKIMNLKDIISAFVRHRRE |
| ERR1759204 | MRIVIEVKRDAVGEVVLNNLYSQTQLQVSFGINMVALHHGQPKIMNLKDIISAFVRHRRE |
| SRR1300699 | MRIVIEVKRDAVGEVVLNNLYSQTQLQVSFGINMVALHHGQPKIMNLKDIISAFVRHRRE |
| S_0825_17 | MRIVIEVKRDAVGEVVLNNLYSQTQLQVSFGINMVALHHGQPKIMNLKDIISAFVRHRRE |
| SRR1958215 | MRIVIEVKRDAVGEVVLNNLYSQTQLQVSFGINMVALHHGQPKIMNLKDIISAFVRHRRE |
| SRR1958540 | MRIVIEVKRDAVGEVVLNNLYSQTQLQVSFGINMVALHHGQPKIMNLKDIISAFVRHRRE |
| SRR1958636 | MRIVIEVKRDAVGEVVLNNLYSQTQLQVSFGINMVALHHGQPKIMNLKDIISAFVRHRRE |
| SRR1959422 | MRIVIEVKRDAVGEVVLNNLYSQTQLQVSFGINMVALHHGQPKIMNLKDIISAFVRHRRE |
| SRR1959427 | MRIVIEVKRDAVGEVVLNNLYSQTQLQVSFGINMVALHHGQPKIMNLKDIISAFVRHRRE |
| SRR1960226 | MRIVIEVKRDAVGEVVLNNLYSQTQLQVSFGINMVALHHGQPKIMNLKDIISAFVRHRRE |
| SRR1963498 | MRIVIEVKRDAVGEVVLNNLYSQTQLQVSFGINMVALHHGQPKIMNLKDIISAFVRHRRE |
| SRR1965947 | MRIVIEVKRDAVGEVVLNNLYSQTQLQVSFGINMVALHHGQPKIMNLKDIISAFVRHRRE |
| SRR1966125 | MRIVIEVKRDAVGEVVLNNLYSQTQLQVSFGINMVALHHGQPKIMNLKDIISAFVRHRRE |
| SRR1966330 | MRIVIEVKRDAVGEVVLNNLYSQTQLQVSFGINMVALHHGQPKIMNLKDIISAFVRHRRE |
| SRR1966565 | MRIVIEVKRDAVGEVVLNNLYSQTQLQVSFGINMVALHHGQPKIMNLKDIISAFVRHRRE |
| SRR1966864 | MRIVIEVKRDAVGEVVLNNLYSQTQLQVSFGINMVALHHGQPKIMNLKDIISAFVRHRRE |
| SRR1966989 | MRIVIEVKRDAVGEVVLNNLYSQTQLQVSFGINMVALHHGQPKIMNLKDIISAFVRHRRE |
| SRR1967688 | MRIVIEVKRDAVGEVVLNNLYSQTQLQVSFGINMVALHHGQPKIMNLKDIISAFVRHRRE |
| SRR1967733 | MRIVIEVKRDAVGEVVLNNLYSQTQLQVSFGINMVALHHGQPKIMNLKDIISAFVRHRRE |
| SRR1967746 | MRIVIEVKRDAVGEVVLNNLYSQTQLQVSFGINMVALHHGQPKIMNLKDIISAFVRHRRE |
| SRR1968341 | MRIVIEVKRDAVGEVVLNNLYSQTQLQVSFGINMVALHHGQPKIMNLKDIISAFVRHRRE |
| SRR1968456 | MRIVIEVKRDAVGEVVLNNLYSQTQLQVSFGINMVALHHGQPKIMNLKDIISAFVRHRRE |
| SRR1968465 | MRIVIEVKRDAVGEVVLNNLYSQTQLQVSFGINMVALHHGQPKIMNLKDIISAFVRHRRE |
| SRR1968761 | MRIVIEVKRDAVGEVVLNNLYSQTQLQVSFGINMVALHHGQPKIMNLKDIISAFVRHRRE |
| SRR1969047 | MRIVIEVKRDAVGEVVLNNLYSQTQLQVSFGINMVALHHGQPKIMNLKDIISAFVRHRRE |
| SRR1969255 | MRIVIEVKRDAVGEVVLNNLYSQTQLQVSFGINMVALHHGQPKIMNLKDIISAFVRHRRE |
| SRR1969412 | MRIVIEVKRDAVGEVVLNNLYSQTQLQVSFGINMVALHHGQPKIMNLKDIISAFVRHRRE |
| SRR1969524 | MRIVIEVKRDAVGEVVLNNLYSQTQLQVSFGINMVALHHGQPKIMNLKDIISAFVRHRRE |
| SRR1969584 | MRIVIEVKRDAVGEVVLNNLYSQTQLQVSFGINMVALHHGQPKIMNLKDIISAFVRHRRE |
| SRR1969648 | MRIVIEVKRDAVGEVVLNNLYSQTQLQVSFGINMVALHHGQPKIMNLKDIISAFVRHRRE |
| SRR1969804 | MRIVIEVKRDAVGEVVLNNLYSQTQLQVSFGINMVALHHGQPKIMNLKDIISAFVRHRRE |
| SRR1970221 | MRIVIEVKRDAVGEVVLNNLYSQTQLQVSFGINMVALHHGQPKIMNLKDIISAFVRHRRE |
| SRR1970268 | MRIVIEVKRDAVGEVVLNNLYSQTQLQVSFGINMVALHHGQPKIMNLKDIISAFVRHRRE |
| SRR1965862 | MRIVIEVKRDAVGEVVLNNLYSQTQLQVSFGINMVALHHGQPKIMNLKDIISAFVRHRRE |
| SRR1967363 | MRIVIEVKRDAVGEVVLNNLYSQTQLQVSFGINMVALHHGQPKIMNLKDIISAFVRHRRE |
| SRR1968276 | MRIVIEVKRDAVGEVVLNNLYSQTQLQVSFGINMVALHHGQPKIMNLKDIISAFVRHRRE |
| SRR1968967 | MRIVIEVKRDAVGEVVLNNLYSQTQLQVSFGINMVALHHGQPKIMNLKDIISAFVRHRRE |
| SRR3321531 | MRIVIEVKRDAVGEVVLNNLYSQTQLQVSFGINMVALHHGQPKIMNLKDIISAFVRHRRE |
| SRR3321883 | MRIVIEVKRDAVGEVVLNNLYSQTQLQVSFGINMVALHHGQPKIMNLKDIISAFVRHRRE |
| SRR3322413 | MRIVIEVKRDAVGEVVLNNLYSQTQLQVSFGINMVALHHGQPKIMNLKDIISAFVRHRRE |
| SRR3323012 | MRIVIEVKRDAVGEVVLNNLYSQTQLQVSFGINMVALHHGQPKIMNLKDIISAFVRHRRE |
| SRR5194289 | MRIVIEVKRDAVGEVVLNNLYSQTQLQVSFGINMVALHHGQPKIMNLKDIISAFVRHRRE |
| SRR7163798 | MRIVIEVKRDAVGEVVLNNLYSQTQLQVSFGINMVALHHGQPKIMNLKDIISAFVRHRRE |
| SRR7172610 | MRIVIEVKRDAVGEVVLNNLYSQTQLQVSFGINMVALHHGQPKIMNLKDIISAFVRHRRE |
| SRR7204568 | MRIVIEVKRDAVGEVVLNNLYSQTQLQVSFGINMVALHHGQPKIMNLKDIISAFVRHRRE |
| SRR7223230 | MRIVIEVKRDAVGEVVLNNLYSQTQLQVSFGINMVALHHGQPKIMNLKDIISAFVRHRRE |
| SRR7230675 | MRIVIEVKRDAVGEVVLNNLYSQTQLQVSFGINMVALHHGQPKIMNLKDIISAFVRHRRE |
| SRR7278056 | MRIVIEVKRDAVGEVVLNNLYSQTQLQVSFGINMVALHHGQPKIMNLKDIISAFVRHRRE |
| SRR7278086 | MRIVIEVKRDAVGEVVLNNLYSQTQLQVSFGINMVALHHGQPKIMNLKDIISAFVRHRRE |
| SRR7285841 | MRIVIEVKRDAVGEVVLNNLYSQTQLQVSFGINMVALHHGQPKIMNLKDIISAFVRHRRE |
| SRR7292625 | MRIVIEVKRDAVGEVVLNNLYSQTQLQVSFGINMVALHHGQPKIMNLKDIISAFVRHRRE |
| SRR7292665 | MRIVIEVKRDAVGEVVLNNLYSQTQLQVSFGINMVALHHGQPKIMNLKDIISAFVRHRRE |
| SRR7297965 | MRIVIEVKRDAVGEVVLNNLYSQTQLQVSFGINMVALHHGQPKIMNLKDIISAFVRHRRE |
| SRR7350726 | MRIVIEVKRDAVGEVVLNNLYSQTQLQVSFGINMVALHHGQPKIMNLKDIISAFVRHRRE |
| SRR7410328 | MRIVIEVKRDAVGEVVLNNLYSQTQLQVSFGINMVALHHGQPKIMNLKDIISAFVRHRRE |
| SRR7474665 | MRIVIEVKRDAVGEVVLNNLYSQTQLQVSFGINMVALHHGQPKIMNLKDIISAFVRHRRE |
| SRR7523184 | MRIVIEVKRDAVGEVVLNNLYSQTQLQVSFGINMVALHHGQPKIMNLKDIISAFVRHRRE |
| SRR7187264 | MRIVIEVKRDAVGEVVLNNLYSQTQLQVSFGINMVALHHGQPKIMNLKDIISAFVRHRRE |
| SRR7204445 | MRIVIEVKRDAVGEVVLNNLYSQTQLQVSFGINMVALHHGQPKIMNLKDIISAFVRHRRE |
| SRR7285641 | MRIVIEVKRDAVGEVVLNNLYSQTQLQVSFGINMVALHHGQPKIMNLKDIISAFVRHRRE |
| SRR7286695 | MRIVIEVKRDAVGEVVLNNLYSQTQLQVSFGINMVALHHGQPKIMNLKDIISAFVRHRRE |
| SRR7286705 | MRIVIEVKRDAVGEVVLNNLYSQTQLQVSFGINMVALHHGQPKIMNLKDIISAFVRHRRE |
| SRR7292931 | MRIVIEVKRDAVGEVVLNNLYSQTQLQVSFGINMVALHHGQPKIMNLKDIISAFVRHRRE |
| SRR7310349 | MRIVIEVKRDAVGEVVLNNLYSQTQLQVSFGINMVALHHGQPKIMNLKDIISAFVRHRRE |
| SRR7351616 | MRIVIEVKRDAVGEVVLNNLYSQTQLQVSFGINMVALHHGQPKIMNLKDIISAFVRHRRE |
| SRR7414818 | MRIVIEVKRDAVGEVVLNNLYSQTQLQVSFGINMVALHHGQPKIMNLKDIISAFVRHRRE |
| SRR7426480 | MRIVIEVKRDAVGEVVLNNLYSQTQLQVSFGINMVALHHGQPKIMNLKDIISAFVRHRRE |
| SRR5584105 | MRIVIEVKRDAVGEVVLNNLYSQTQLQVSFGINMVALHHGQPKIMNLKDIISAFVRHRRE |
| SRR5584565 | MRIVIEVKRDAVGEVVLNNLYSQTQLQVSFGINMVALHHGQPKIMNLKDIISAFVRHRRE |
| SRR5584614 | MRIVIEVKRDAVGEVVLNNLYSQTQLQVSFGINMVALHHGQPKIMNLKDIISAFVRHRRE |
| SRR5631543 | MRIVIEVKRDAVGEVVLNNLYSQTQLQVSFGINMVALHHGQPKIMNLKDIISAFVRHRRE |
| SRR5631553 | MRIVIEVKRDAVGEVVLNNLYSQTQLQVSFGINMVALHHGQPKIMNLKDIISAFVRHRRE |
| SRR7123196 | MRIVIEVKRDAVGEVVLNNLYSQTQLQVSFGINMVALHHGQPKIMNLKDIISAFVRHRRE |
| SRR7163819 | MRIVIEVKRDAVGEVVLNNLYSQTQLQVSFGINMVALHHGQPKIMNLKDIISAFVRHRRE |
| SRR7163920 | MRIVIEVKRDAVGEVVLNNLYSQTQLQVSFGINMVALHHGQPKIMNLKDIISAFVRHRRE |
| SRR7209528 | MRIVIEVKRDAVGEVVLNNLYSQTQLQVSFGINMVALHHGQPKIMNLKDIISAFVRHRRE |
| SRR7249868 | MRIVIEVKRDAVGEVVLNNLYSQTQLQVSFGINMVALHHGQPKIMNLKDIISAFVRHRRE |
| SRR7278088 | MRIVIEVKRDAVGEVVLNNLYSQTQLQVSFGINMVALHHGQPKIMNLKDIISAFVRHRRE |
| SRR7285788 | MRIVIEVKRDAVGEVVLNNLYSQTQLQVSFGINMVALHHGQPKIMNLKDIISAFVRHRRE |
| SRR7286789 | MRIVIEVKRDAVGEVVLNNLYSQTQLQVSFGINMVALHHGQPKIMNLKDIISAFVRHRRE |
| SRR7286886 | MRIVIEVKRDAVGEVVLNNLYSQTQLQVSFGINMVALHHGQPKIMNLKDIISAFVRHRRE |
| SRR7310632 | MRIVIEVKRDAVGEVVLNNLYSQTQLQVSFGINMVALHHGQPKIMNLKDIISAFVRHRRE |
| SRR7350631 | MRIVIEVKRDAVGEVVLNNLYSQTQLQVSFGINMVALHHGQPKIMNLKDIISAFVRHRRE |
| SRR7458741 | MRIVIEVKRDAVGEVVLNNLYSQTQLQVSFGINMVALHHGQPKIMNLKDIISAFVRHRRE |
| SRR7480280 | MRIVIEVKRDAVGEVVLNNLYSQTQLQVSFGINMVALHHGQPKIMNLKDIISAFVRHRRE |
| SRR7523660 | MRIVIEVKRDAVGEVVLNNLYSQTQLQVSFGINMVALHHGQPKIMNLKDIISAFVRHRRE |
| SRR7523775 | MRIVIEVKRDAVGEVVLNNLYSQTQLQVSFGINMVALHHGQPKIMNLKDIISAFVRHRRE |
| SRR7251101 | MRIVIEVKRDAVGEVVLNNLYSQTQLQVSFGINMVALHHGQPKIMNLKDIISAFVRHRRE |
| SRR7284299 | MRIVIEVKRDAVGEVVLNNLYSQTQLQVSFGINMVALHHGQPKIMNLKDIISAFVRHRRE |
| SRR7285738 | MRIVIEVKRDAVGEVVLNNLYSQTQLQVSFGINMVALHHGQPKIMNLKDIISAFVRHRRE |
| SRR7310640 | MRIVIEVKRDAVGEVVLNNLYSQTQLQVSFGINMVALHHGQPKIMNLKDIISAFVRHRRE |
| SRR7349159 | MRIVIEVKRDAVGEVVLNNLYSQTQLQVSFGINMVALHHGQPKIMNLKDIISAFVRHRRE |
| SRR7474873 | MRIVIEVKRDAVGEVVLNNLYSQTQLQVSFGINMVALHHGQPKIMNLKDIISAFVRHRRE |
| SRR7495689 | MRIVIEVKRDAVGEVVLNNLYSQTQLQVSFGINMVALHHGQPKIMNLKDIISAFVRHRRE |
| SRR7495752 | MRIVIEVKRDAVGEVVLNNLYSQTQLQVSFGINMVALHHGQPKIMNLKDIISAFVRHRRE |
| ----------------------------------------------------------------------------- | |
| S16BD08730 | VVTRRTIFELRKARDRAHILEALAIALANIDPIIELIRRAPTPAEAKAALISRPWDLGNV |
| S18BD00684 | VVTRRTIFELRKARDRAHILEALAIALANIDPIIELIRRAPTPAEAKAALISRPWDLGNV |
| S18BD03994 | VVTRRTIFELRKARDRAHILEALAIALANIDPIIELIRRAPTPAEAKAALISRPWDLGNV |
| S18BD05011 | VVTRRTIFELRKARDRAHILEALAIALANIDPIIELIRRAPTPAEAKAALISRPWDLGNV |
| RKI_16-03723 | VVTRRTIFELRKARDRAHILEALAIALANIDPIIELIRRAPTPAEAKAALISRPWDLGNV |
| RKI_16-04315 | VVTRRTIFELRKARDRAHILEALAIALANIDPIIELIRRAPTPAEAKAALISRPWDLGNV |
| RKI_17-02304 | VVTRRTIFELRKARDRAHILEALAIALANIDPIIELIRRAPTPAEAKAALISRPWDLGNV |
| RKI_17-02411 | VVTRRTIFELRKARDRAHILEALAIALANIDPIIELIRRAPTPAEAKAALISRPWDLGNV |
| RKI_17-02757 | VVTRRTIFELRKARDRAHILEALAIALANIDPIIELIRRAPTPAEAKAALISRPWDLGNV |
| RKI_17-04797 | VVTRRTIFELRKARDRAHILEALAIALANIDPIIELIRRAPTPAEAKAALISRPWDLGNV |
| RKI_17-06869 | VVTRRTIFELRKARDRAHILEALAIALANIDPIIELIRRAPTPAEAKAALISRPWDLGNV |
| ERR2580277 | VVTRRTIFELRKARDRAHILEALAIALANIDPIIELIRRAPTPAEAKAALISRPWDLGNV |
| ERR2580276 | VVTRRTIFELRKARDRAHILEALAIALANIDPIIELIRRAPTPAEAKAALISRPWDLGNV |
| ERR2580273 | VVTRRTIFELRKARDRAHILEALAIALANIDPIIELIRRAPTPAEAKAALISRPWDLGNV |
| ERR2580274 | VVTRRTIFELRKARDRAHILEALAIALANIDPIIELIRRAPTPAEAKAALISRPWDLGNV |
| ERR2173656 | VVTRRTIFELRKARDRAHILEALAIALANIDPIIELIRRAPTPAEAKAALISRPWDLGNV |
| 17041676 | VVTRRTIFELRKARDRAHILEALAIALANIDPIIELIRRAPTPAEAKAALISRPWDLGNV |
| MT16-000061 | VVTRRTIFELRKARDRAHILEALAIALANIDPIIELIRRAPTPAEAKAALISRPWDLGNV |
| MT16-019416 | VVTRRTIFELRKARDRAHILEALAIALANIDPIIELIRRAPTPAEAKAALISRPWDLGNV |
| MT16-027865 | VVTRRTIFELRKARDRAHILEALAIALANIDPIIELIRRAPTPAEAKAALISRPWDLGNV |
| MT16-031693 | VVTRRTIFELRKARDRAHILEALAIALANIDPIIELIRRAPTPAEAKAALISRPWDLGNV |
| MT16-040253 | VVTRRTIFELRKARDRAHILEALAIALANIDPIIELIRRAPTPAEAKAALISRPWDLGNV |
| MT16-045379 | VVTRRTIFELRKARDRAHILEALAIALANIDPIIELIRRAPTPAEAKAALISRPWDLGNV |
| MT16-442728 | VVTRRTIFELRKARDRAHILEALAIALANIDPIIELIRRAPTPAEAKAALISRPWDLGNV |
| MT16-462857 | VVTRRTIFELRKARDRAHILEALAIALANIDPIIELIRRAPTPAEAKAALISRPWDLGNV |
| MT16-480196 | VVTRRTIFELRKARDRAHILEALAIALANIDPIIELIRRAPTPAEAKAALISRPWDLGNV |
| MT16-861555 | VVTRRTIFELRKARDRAHILEALAIALANIDPIIELIRRAPTPAEAKAALISRPWDLGNV |
| MT17-076833 | VVTRRTIFELRKARDRAHILEALAIALANIDPIIELIRRAPTPAEAKAALISRPWDLGNV |
| MT17-110677 | VVTRRTIFELRKARDRAHILEALAIALANIDPIIELIRRAPTPAEAKAALISRPWDLGNV |
| MT17-131730 | VVTRRTIFELRKARDRAHILEALAIALANIDPIIELIRRAPTPAEAKAALISRPWDLGNV |
| MT17-140890 | VVTRRTIFELRKARDRAHILEALAIALANIDPIIELIRRAPTPAEAKAALISRPWDLGNV |
| MT17-141840 | VVTRRTIFELRKARDRAHILEALAIALANIDPIIELIRRAPTPAEAKAALISRPWDLGNV |
| MT17-152488 | VVTRRTIFELRKARDRAHILEALAIALANIDPIIELIRRAPTPAEAKAALISRPWDLGNV |
| MT17-157311 | VVTRRTIFELRKARDRAHILEALAIALANIDPIIELIRRAPTPAEAKAALISRPWDLGNV |
| MT17-161645 | VVTRRTIFELRKARDRAHILEALAIALANIDPIIELIRRAPTPAEAKAALISRPWDLGNV |
| MT17-167951 | VVTRRTIFELRKARDRAHILEALAIALANIDPIIELIRRAPTPAEAKAALISRPWDLGNV |
| MT18-217732 | VVTRRTIFELRKARDRAHILEALAIALANIDPIIELIRRAPTPAEAKAALISRPWDLGNV |
| MT18-252580 | VVTRRTIFELRKARDRAHILEALAIALANIDPIIELIRRAPTPAEAKAALISRPWDLGNV |
| RIVM_H_2009-01 | VVTRRTIFELRKARDRAHILEALAIALANIDPIIELIRRAPTPAEAKAALISRPWDLGNV |
| RIVM_H_2010-01 | VVTRRTIFELRKARDRAHILEALAIALANIDPIIELIRRAPTPAEAKAALISRPWDLGNV |
| RIVM_H_2010-02 | VVTRRTIFELRKARDRAHILEALAIALANIDPIIELIRRAPTPAEAKAALISRPWDLGNV |
| RIVM_H_2011-01 | VVTRRTIFELRKARDRAHILEALAIALANIDPIIELIRRAPTPAEAKAALISRPWDLGNV |
| RIVM_H_2011-02 | VVTRRTIFELRKARDRAHILEALAIALANIDPIIELIRRAPTPAEAKAALISRPWDLGNV |
| RIVM_H_2011-03 | VVTRRTIFELRKARDRAHILEALAIALANIDPIIELIRRAPTPAEAKAALISRPWDLGNV |
| RIVM_H_2013-01 | VVTRRTIFELRKARDRAHILEALAIALANIDPIIELIRRAPTPAEAKAALISRPWDLGNV |
| RIVM_H_2013-02 | VVTRRTIFELRKARDRAHILEALAIALANIDPIIELIRRAPTPAEAKAALISRPWDLGNV |
| RIVM_H_2014-01 | VVTRRTIFELRKARDRAHILEALAIALANIDPIIELIRRAPTPAEAKAALISRPWDLGNV |
| RIVM_H_2014-02 | VVTRRTIFELRKARDRAHILEALAIALANIDPIIELIRRAPTPAEAKAALISRPWDLGNV |
| RIVM_H_2016-01 | VVTRRTIFELRKARDRAHILEALAIALANIDPIIELIRRAPTPAEAKAALISRPWDLGNV |
| RIVM_H_2016-02 | VVTRRTIFELRKARDRAHILEALAIALANIDPIIELIRRAPTPAEAKAALISRPWDLGNV |
| RIVM_H_2016-03 | VVTRRTIFELRKARDRAHILEALAIALANIDPIIELIRRAPTPAEAKAALISRPWDLGNV |
| RIVM_H_2016-04 | VVTRRTIFELRKARDRAHILEALAIALANIDPIIELIRRAPTPAEAKAALISRPWDLGNV |
| RIVM_H_2016-05 | VVTRRTIFELRKARDRAHILEALAIALANIDPIIELIRRAPTPAEAKAALISRPWDLGNV |
| RIVM_H_2016-06 | VVTRRTIFELRKARDRAHILEALAIALANIDPIIELIRRAPTPAEAKAALISRPWDLGNV |
| RIVM_H_2016-07 | VVTRRTIFELRKARDRAHILEALAIALANIDPIIELIRRAPTPAEAKAALISRPWDLGNV |
| RIVM_H_2016-08 | VVTRRTIFELRKARDRAHILEALAIALANIDPIIELIRRAPTPAEAKAALISRPWDLGNV |
| RIVM_H_2016-09 | VVTRRTIFELRKARDRAHILEALAIALANIDPIIELIRRAPTPAEAKAALISRPWDLGNV |
| RIVM_H_2016-10 | VVTRRTIFELRKARDRAHILEALAIALANIDPIIELIRRAPTPAEAKAALISRPWDLGNV |
| RIVM_H_2016-11 | VVTRRTIFELRKARDRAHILEALAIALANIDPIIELIRRAPTPAEAKAALISRPWDLGNV |
| RIVM_H_2016-12 | VVTRRTIFELRKARDRAHILEALAIALANIDPIIELIRRAPTPAEAKAALISRPWDLGNV |
| RIVM_H_2016-13 | VVTRRTIFELRKARDRAHILEALAIALANIDPIIELIRRAPTPAEAKAALISRPWDLGNV |
| RIVM_H_2016-14 | VVTRRTIFELRKARDRAHILEALAIALANIDPIIELIRRAPTPAEAKAALISRPWDLGNV |
| RIVM_H_2016-15 | VVTRRTIFELRKARDRAHILEALAIALANIDPIIELIRRAPTPAEAKAALISRPWDLGNV |
| RIVM_H_2017-01 | VVTRRTIFELRKARDRAHILEALAIALANIDPIIELIRRAPTPAEAKAALISRPWDLGNV |
| RIVM_H_2017-02 | VVTRRTIFELRKARDRAHILEALAIALANIDPIIELIRRAPTPAEAKAALISRPWDLGNV |
| RIVM_H_2017-03 | VVTRRTIFELRKARDRAHILEALAIALANIDPIIELIRRAPTPAEAKAALISRPWDLGNV |
| RIVM_H_2017-04 | VVTRRTIFELRKARDRAHILEALAIALANIDPIIELIRRAPTPAEAKAALISRPWDLGNV |
| RIVM_H_2017-05 | VVTRRTIFELRKARDRAHILEALAIALANIDPIIELIRRAPTPAEAKAALISRPWDLGNV |
| RIVM_H_2017-06 | VVTRRTIFELRKARDRAHILEALAIALANIDPIIELIRRAPTPAEAKAALISRPWDLGNV |
| RIVM_H_2017-07 | VVTRRTIFELRKARDRAHILEALAIALANIDPIIELIRRAPTPAEAKAALISRPWDLGNV |
| RIVM_H_2017-08 | VVTRRTIFELRKARDRAHILEALAIALANIDPIIELIRRAPTPAEAKAALISRPWDLGNV |
| RIVM_H_2017-09 | VVTRRTIFELRKARDRAHILEALAIALANIDPIIELIRRAPTPAEAKAALISRPWDLGNV |
| RIVM_H_2017-10 | VVTRRTIFELRKARDRAHILEALAIALANIDPIIELIRRAPTPAEAKAALISRPWDLGNV |
| RIVM_H_2017-11 | VVTRRTIFELRKARDRAHILEALAIALANIDPIIELIRRAPTPAEAKAALISRPWDLGNV |
| RIVM_H_2017-12 | VVTRRTIFELRKARDRAHILEALAIALANIDPIIELIRRAPTPAEAKAALISRPWDLGNV |
| RIVM_H_2017-13 | VVTRRTIFELRKARDRAHILEALAIALANIDPIIELIRRAPTPAEAKAALISRPWDLGNV |
| RIVM_H_2017-14 | VVTRRTIFELRKARDRAHILEALAIALANIDPIIELIRRAPTPAEAKAALISRPWDLGNV |
| RIVM_H_2017-15 | VVTRRTIFELRKARDRAHILEALAIALANIDPIIELIRRAPTPAEAKAALISRPWDLGNV |
| RIVM_H_2017-16 | VVTRRTIFELRKARDRAHILEALAIALANIDPIIELIRRAPTPAEAKAALISRPWDLGNV |
| RIVM_H_2017-17 | VVTRRTIFELRKARDRAHILEALAIALANIDPIIELIRRAPTPAEAKAALISRPWDLGNV |
| RIVM_H_2017-18 | VVTRRTIFELRKARDRAHILEALAIALANIDPIIELIRRAPTPAEAKAALISRPWDLGNV |
| RIVM_H_2017-19 | VVTRRTIFELRKARDRAHILEALAIALANIDPIIELIRRAPTPAEAKAALISRPWDLGNV |
| 15EP001483 | VVTRRTIFELRKARDRAHILEALAIALANIDPIIELIRRAPTPAEAKAALISRPWDLGNV |
| 17EP002363 | VVTRRTIFELRKARDRAHILEALAIALANIDPIIELIRRAPTPAEAKAALISRPWDLGNV |
| S_0812_17 | VVTRRTIFELRKARDRAHILEALAIALANIDPIIELIRRAPTPAEAKAALISRPWDLGNV |
| SRR1957844 | VVTRRTIFELRKARDRAHILEALAIALANIDPIIELIRRAPTPAEAKAALISRPWDLGNV |
| SRR1958654 | VVTRRTIFELRKARDRAHILEALAIALANIDPIIELIRRAPTPAEAKAALISRPWDLGNV |
| SRR1965077 | VVTRRTIFELRKARDRAHILEALAIALANIDPIIELIRRAPTPAEAKAALISRPWDLGNV |
| SRR1966369 | VVTRRTIFELRKARDRAHILEALAIALANIDPIIELIRRAPTPAEAKAALISRPWDLGNV |
| SRR1967117 | VVTRRTIFELRKARDRAHILEALAIALANIDPIIELIRRAPTPAEAKAALISRPWDLGNV |
| SRR1967922 | VVTRRTIFELRKARDRAHILEALAIALANIDPIIELIRRAPTPAEAKAALISRPWDLGNV |
| SRR8704720 | VVTRRTIFELRKARDRAHILEALAIALANIDPIIELIRRAPTPAEAKAALISRPWDLGNV |
| SRR7216071 | VVTRRTIFELRKARDRAHILEALAIALANIDPIIELIRRAPTPAEAKAALISRPWDLGNV |
| SRR7349175 | VVTRRTIFELRKARDRAHILEALAIALANIDPIIELIRRAPTPAEAKAALISRPWDLGNV |
| SRR7523148 | VVTRRTIFELRKARDRAHILEALAIALANIDPIIELIRRAPTPAEAKAALISRPWDLGNV |
| SRR7523854 | VVTRRTIFELRKARDRAHILEALAIALANIDPIIELIRRAPTPAEAKAALISRPWDLGNV |
| 313865 | VVTRRTIFELRKARDRAHILEALAIALANIDPIIELIRRAPTPAEAKAALISRPWDLGNV |
| SRR7277793 | VVTRRTIFELRKARDRAHILEALAIALANIDPIIELIRRAPTPAEAKAALISRPWDLGNV |
| SRR7343877 | VVTRRTIFELRKARDRAHILEALAIALANIDPIIELIRRAPTPAEAKAALISRPWDLGNV |
| SRR7351477 | VVTRRTIFELRKARDRAHILEALAIALANIDPIIELIRRAPTPAEAKAALISRPWDLGNV |
| SRR5583183 | VVTRRTIFELRKARDRAHILEALAIALANIDPIIELIRRAPTPAEAKAALISRPWDLGNV |
| SRR5585240 | VVTRRTIFELRKARDRAHILEALAIALANIDPIIELIRRAPTPAEAKAALISRPWDLGNV |
| SRR7284317 | VVTRRTIFELRKARDRAHILEALAIALANIDPIIELIRRAPTPAEAKAALISRPWDLGNV |
| SRR7299161 | VVTRRTIFELRKARDRAHILEALAIALANIDPIIELIRRAPTPAEAKAALISRPWDLGNV |
| SRR7401730 | VVTRRTIFELRKARDRAHILEALAIALANIDPIIELIRRAPTPAEAKAALISRPWDLGNV |
| SRR7469092 | VVTRRTIFELRKARDRAHILEALAIALANIDPIIELIRRAPTPAEAKAALISRPWDLGNV |
| SRR7879556 | VVTRRTIFELRKARDRAHILEALAIALANIDPIIELIRRAPTPAEAKAALISRPWDLGNV |
| SRR8526100 | VVTRRTIFELRKARDRAHILEALAIALANIDPIIELIRRAPTPAEAKAALISRPWDLGNV |
| SRR8553991 | VVTRRTIFELRKARDRAHILEALAIALANIDPIIELIRRAPTPAEAKAALISRPWDLGNV |
| SRR7842487 | VVTRRTIFELRKARDRAHILEALAIALANIDPIIELIRRAPTPAEAKAALISRPWDLGNV |
| SRR8054524 | VVTRRTIFELRKARDRAHILEALAIALANIDPIIELIRRAPTPAEAKAALISRPWDLGNV |
| SRR8054525 | VVTRRTIFELRKARDRAHILEALAIALANIDPIIELIRRAPTPAEAKAALISRPWDLGNV |
| SRR8524733 | VVTRRTIFELRKARDRAHILEALAIALANIDPIIELIRRAPTPAEAKAALISRPWDLGNV |
| SRR4093291 | VVTRRTIFELRKARDRAHILEALAIALANIDPIIELIRRAPTPAEAKAALISRPWDLGNV |
| SRR4245549 | VVTRRTIFELRKARDRAHILEALAIALANIDPIIELIRRAPTPAEAKAALISRPWDLGNV |
| SRR3057154 | VVTRRTIFELRKARDRAHILEALAIALANIDPIIELIRRAPTPAEAKAALISRPWDLGNV |
| SRR1726150 | VVTRRTIFELRKARDRAHILEALAIALANIDPIIELIRRAPTPAEAKAALISRPWDLGNV |
| SRR1996141 | VVTRRTIFELRKARDRAHILEALAIALANIDPIIELIRRAPTPAEAKAALISRPWDLGNV |
| SRR1107842 | VVTRRTIFELRKARDRAHILEALAIALANIDPIIELIRRAPTPAEAKAALISRPWDLGNV |
| SRR1157587 | VVTRRTIFELRKARDRAHILEALAIALANIDPIIELIRRAPTPAEAKAALISRPWDLGNV |
| SRR3027706 | VVTRRTIFELRKARDRAHILEALAIALANIDPIIELIRRAPTPAEAKAALISRPWDLGNV |
| SRR3027707 | VVTRRTIFELRKARDRAHILEALAIALANIDPIIELIRRAPTPAEAKAALISRPWDLGNV |
| SRR3027708 | VVTRRTIFELRKARDRAHILEALAIALANIDPIIELIRRAPTPAEAKAALISRPWDLGNV |
| SRR3027710 | VVTRRTIFELRKARDRAHILEALAIALANIDPIIELIRRAPTPAEAKAALISRPWDLGNV |
| SRR3027711 | VVTRRTIFELRKARDRAHILEALAIALANIDPIIELIRRAPTPAEAKAALISRPWDLGNV |
| SRR3027716 | VVTRRTIFELRKARDRAHILEALAIALANIDPIIELIRRAPTPAEAKAALISRPWDLGNV |
| SRR3027717 | VVTRRTIFELRKARDRAHILEALAIALANIDPIIELIRRAPTPAEAKAALISRPWDLGNV |
| SRR3027719 | VVTRRTIFELRKARDRAHILEALAIALANIDPIIELIRRAPTPAEAKAALISRPWDLGNV |
| SRR3027721 | VVTRRTIFELRKARDRAHILEALAIALANIDPIIELIRRAPTPAEAKAALISRPWDLGNV |
| SRR3027723 | VVTRRTIFELRKARDRAHILEALAIALANIDPIIELIRRAPTPAEAKAALISRPWDLGNV |
| SRR3115978 | VVTRRTIFELRKARDRAHILEALAIALANIDPIIELIRRAPTPAEAKAALISRPWDLGNV |
| SRR2534093 | VVTRRTIFELRKARDRAHILEALAIALANIDPIIELIRRAPTPAEAKAALISRPWDLGNV |
| SRR2534094 | VVTRRTIFELRKARDRAHILEALAIALANIDPIIELIRRAPTPAEAKAALISRPWDLGNV |
| SRR2534095 | VVTRRTIFELRKARDRAHILEALAIALANIDPIIELIRRAPTPAEAKAALISRPWDLGNV |
| SRR2534108 | VVTRRTIFELRKARDRAHILEALAIALANIDPIIELIRRAPTPAEAKAALISRPWDLGNV |
| SRR1106464 | VVTRRTIFELRKARDRAHILEALAIALANIDPIIELIRRAPTPAEAKAALISRPWDLGNV |
| SRR1106463 | VVTRRTIFELRKARDRAHILEALAIALANIDPIIELIRRAPTPAEAKAALISRPWDLGNV |
| SRR6949610 | VVTRRTIFELRKARDRAHILEALAIALANIDPIIELIRRAPTPAEAKAALISRPWDLGNV |
| SRR6950452 | VVTRRTIFELRKARDRAHILEALAIALANIDPIIELIRRAPTPAEAKAALISRPWDLGNV |
| ERR2019831 | VVTRRTIFELRKARDRAHILEALAIALANIDPIIELIRRAPTPAEAKAALISRPWDLGNV |
| SRR2085693 | VVTRRTIFELRKARDRAHILEALAIALANIDPIIELIRRAPTPAEAKAALISRPWDLGNV |
| SRR2086898 | VVTRRTIFELRKARDRAHILEALAIALANIDPIIELIRRAPTPAEAKAALISRPWDLGNV |
| SRR2175312 | VVTRRTIFELRKARDRAHILEALAIALANIDPIIELIRRAPTPAEAKAALISRPWDLGNV |
| SRR2175360 | VVTRRTIFELRKARDRAHILEALAIALANIDPIIELIRRAPTPAEAKAALISRPWDLGNV |
| SRR5231997 | VVTRRTIFELRKARDRAHILEALAIALANIDPIIELIRRAPTPAEAKAALISRPWDLGNV |
| SRR5232003 | VVTRRTIFELRKARDRAHILEALAIALANIDPIIELIRRAPTPAEAKAALISRPWDLGNV |
| SRR5232015 | VVTRRTIFELRKARDRAHILEALAIALANIDPIIELIRRAPTPAEAKAALISRPWDLGNV |
| SRR949434 | VVTRRTIFELRKARDRAHILEALAIALANIDPIIELIRRAPTPAEAKAALISRPWDLGNV |
| SRR3216575 | VVTRRTIFELRKARDRAHILEALAIALANIDPIIELIRRAPTPAEAKAALISRPWDLGNV |
| SRR5205342 | VVTRRTIFELRKARDRAHILEALAIALANIDPIIELIRRAPTPAEAKAALISRPWDLGNV |
| SRR1501669 | VVTRRTIFELRKARDRAHILEALAIALANIDPIIELIRRAPTPAEAKAALISRPWDLGNV |
| SRR5209740 | VVTRRTIFELRKARDRAHILEALAIALANIDPIIELIRRAPTPAEAKAALISRPWDLGNV |
| SRR3240355 | VVTRRTIFELRKARDRAHILEALAIALANIDPIIELIRRAPTPAEAKAALISRPWDLGNV |
| SRR3392777 | VVTRRTIFELRKARDRAHILEALAIALANIDPIIELIRRAPTPAEAKAALISRPWDLGNV |
| SRR3593671 | VVTRRTIFELRKARDRAHILEALAIALANIDPIIELIRRAPTPAEAKAALISRPWDLGNV |
| SRR5413290 | VVTRRTIFELRKARDRAHILEALAIALANIDPIIELIRRAPTPAEAKAALISRPWDLGNV |
| SRR5590269 | VVTRRTIFELRKARDRAHILEALAIALANIDPIIELIRRAPTPAEAKAALISRPWDLGNV |
| SRR5812103 | VVTRRTIFELRKARDRAHILEALAIALANIDPIIELIRRAPTPAEAKAALISRPWDLGNV |
| SRR2830941 | VVTRRTIFELRKARDRAHILEALAIALANIDPIIELIRRAPTPAEAKAALISRPWDLGNV |
| SRR2830966 | VVTRRTIFELRKARDRAHILEALAIALANIDPIIELIRRAPTPAEAKAALISRPWDLGNV |
| SRR3137270 | VVTRRTIFELRKARDRAHILEALAIALANIDPIIELIRRAPTPAEAKAALISRPWDLGNV |
| SRR3137271 | VVTRRTIFELRKARDRAHILEALAIALANIDPIIELIRRAPTPAEAKAALISRPWDLGNV |
| ERR526807 | VVTRRTIFELRKARDRAHILEALAIALANIDPIIELIRRAPTPAEAKAALISRPWDLGNV |
| ERR2197922 | VVTRRTIFELRKARDRAHILEALAIALANIDPIIELIRRAPTPAEAKAALISRPWDLGNV |
| ERR2197923 | VVTRRTIFELRKARDRAHILEALAIALANIDPIIELIRRAPTPAEAKAALISRPWDLGNV |
| ERR2197924 | VVTRRTIFELRKARDRAHILEALAIALANIDPIIELIRRAPTPAEAKAALISRPWDLGNV |
| ERR2197925 | VVTRRTIFELRKARDRAHILEALAIALANIDPIIELIRRAPTPAEAKAALISRPWDLGNV |
| ERR2197927 | VVTRRTIFELRKARDRAHILEALAIALANIDPIIELIRRAPTPAEAKAALISRPWDLGNV |
| ERR2197929 | VVTRRTIFELRKARDRAHILEALAIALANIDPIIELIRRAPTPAEAKAALISRPWDLGNV |
| SRR1648149 | VVTRRTIFELRKARDRAHILEALAIALANIDPIIELIRRAPTPAEAKAALISRPWDLGNV |
| SRR1048299 | VVTRRTIFELRKARDRAHILEALAIALANIDPIIELIRRAPTPAEAKAALISRPWDLGNV |
| SRR1300677 | VVTRRTIFELRKARDRAHILEALAIALANIDPIIELIRRAPTPAEAKAALISRPWDLGNV |
| SRR1288356 | VVTRRTIFELRKARDRAHILEALAIALANIDPIIELIRRAPTPAEAKAALISRPWDLGNV |
| SRR7426190 | VVTRRTIFELRKARDRAHILEALAIALANIDPIIELIRRAPTPAEAKAALISRPWDLGNV |
| SRR7426192 | VVTRRTIFELRKARDRAHILEALAIALANIDPIIELIRRAPTPAEAKAALISRPWDLGNV |
| SRR7426193 | VVTRRTIFELRKARDRAHILEALAIALANIDPIIELIRRAPTPAEAKAALISRPWDLGNV |
| SRR7441832 | VVTRRTIFELRKARDRAHILEALAIALANIDPIIELIRRAPTPAEAKAALISRPWDLGNV |
| SRR7426179 | VVTRRTIFELRKARDRAHILEALAIALANIDPIIELIRRAPTPAEAKAALISRPWDLGNV |
| SRR7439238 | VVTRRTIFELRKARDRAHILEALAIALANIDPIIELIRRAPTPAEAKAALISRPWDLGNV |
| SRR7439244 | VVTRRTIFELRKARDRAHILEALAIALANIDPIIELIRRAPTPAEAKAALISRPWDLGNV |
| SRR7439259 | VVTRRTIFELRKARDRAHILEALAIALANIDPIIELIRRAPTPAEAKAALISRPWDLGNV |
| SRR7439260 | VVTRRTIFELRKARDRAHILEALAIALANIDPIIELIRRAPTPAEAKAALISRPWDLGNV |
| SRR7441786 | VVTRRTIFELRKARDRAHILEALAIALANIDPIIELIRRAPTPAEAKAALISRPWDLGNV |
| SRR7441797 | VVTRRTIFELRKARDRAHILEALAIALANIDPIIELIRRAPTPAEAKAALISRPWDLGNV |
| ERR1759093 | VVTRRTIFELRKARDRAHILEALAIALANIDPIIELIRRAPTPAEAKAALISRPWDLGNV |
| ERR2580275 | VVTRRTIFELRKARDRAHILEALAIALANIDPIIELIRRAPTPAEAKAALISRPWDLGNV |
| ERR1759204 | VVTRRTIFELRKARDRAHILEALAIALANIDPIIELIRRAPTPAEAKAALISRPWDLGNV |
| SRR1300699 | VVTRRTIFELRKARDRAHILEALAIALANIDPIIELIRRAPTPAEAKAALISRPWDLGNV |
| S_0825_17 | VVTRRTIFELRKARDRAHILEALAIALANIDPIIELIRRAPTPAEAKAALISRPWDLGNV |
| SRR1958215 | VVTRRTIFELRKARDRAHILEALAIALANIDPIIELIRRAPTPAEAKAALISRPWDLGNV |
| SRR1958540 | VVTRRTIFELRKARDRAHILEALAIALANIDPIIELIRRAPTPAEAKAALISRPWDLGNV |
| SRR1958636 | VVTRRTIFELRKARDRAHILEALAIALANIDPIIELIRRAPTPAEAKAALISRPWDLGNV |
| SRR1959422 | VVTRRTIFELRKARDRAHILEALAIALANIDPIIELIRRAPTPAEAKAALISRPWDLGNV |
| SRR1959427 | VVTRRTIFELRKARDRAHILEALAIALANIDPIIELIRRAPTPAEAKAALISRPWDLGNV |
| SRR1960226 | VVTRRTIFELRKARDRAHILEALAIALANIDPIIELIRRAPTPAEAKAALISRPWDLGNV |
| SRR1963498 | VVTRRTIFELRKARDRAHILEALAIALANIDPIIELIRRAPTPAEAKAALISRPWDLGNV |
| SRR1965947 | VVTRRTIFELRKARDRAHILEALAIALANIDPIIELIRRAPTPAEAKAALISRPWDLGNV |
| SRR1966125 | VVTRRTIFELRKARDRAHILEALAIALANIDPIIELIRRAPTPAEAKAALISRPWDLGNV |
| SRR1966330 | VVTRRTIFELRKARDRAHILEALAIALANIDPIIELIRRAPTPAEAKAALISRPWDLGNV |
| SRR1966565 | VVTRRTIFELRKARDRAHILEALAIALANIDPIIELIRRAPTPAEAKAALISRPWDLGNV |
| SRR1966864 | VVTRRTIFELRKARDRAHILEALAIALTNIDPIIELIRRAPTPAEAKAALISRPWDLGNV |
| SRR1966989 | VVTRRTIFELRKARDRAHILEALAIALANIDPIIELIRRAPTPAEAKAALISRPWDLGNV |
| SRR1967688 | VVTRRTIFELRKARDRAHILEALAIALANIDPIIELIRRAPTPAEAKAALISRPWDLGNV |
| SRR1967733 | VVTRRTIFELRKARDRAHILEALAIALANIDPIIELIRRAPTPAEAKAALISRPWDLGNV |
| SRR1967746 | VVTRRTIFELRKARDRAHILEALAIALANIDPIIELIRRAPTPAEAKAALISRPWDLGNV |
| SRR1968341 | VVTRRTIFELRKARDRAHILEALAIALANIDPIIELIRRAPTPAEAKAALISRPWDLGNV |
| SRR1968456 | VVTRRTIFELRKARDRAHILEALAIALANIDPIIELIRRAPTPAEAKAALISRPWDLGNV |
| SRR1968465 | VVTRRTIFELRKARDRAHILEALAIALANIDPIIELIRRAPTPAEAKAALISRPWDLGNV |
| SRR1968761 | VVTRRTIFELRKARDRAHILEALAIALANIDPIIELIRRAPTPAEAKAALISRPWDLGNV |
| SRR1969047 | VVTRRTIFELRKARDRAHILEALAIALANIDPIIELIRRAPTPAEAKAALISRPWDLGNV |
| SRR1969255 | VVTRRTIFELRKARDRAHILEALAIALANIDPIIELIRRAPTPAEAKAALISRPWDLGNV |
| SRR1969412 | VVTRRTIFELRKARDRAHILEALAIALANIDPIIELIRRAPTPAEAKAALISRPWDLGNV |
| SRR1969524 | VVTRRTIFELRKARDRAHILEALAIALANIDPIIELIRRAPTPAEAKAALISRPWDLGNV |
| SRR1969584 | VVTRRTIFELRKARDRAHILEALAIALANIDPIIELIRRAPTPAEAKAALISRPWDLGNV |
| SRR1969648 | VVTRRTIFELRKARDRAHILEALAIALANIDPIIELIRRAPTPAEAKAALISRPWDLGNV |
| SRR1969804 | VVTRRTIFELRKARDRAHILEALAIALANIDPIIELIRRAPTPAEAKAALISRPWDLGNV |
| SRR1970221 | VVTRRTIFELRKARDRAHILEALAIALANIDPIIELIRRAPTPAEAKAALISRPWDLGNV |
| SRR1970268 | VVTRRTIFELRKARDRAHILEALAIALANIDPIIELIRRAPTPAEAKAALISRPWDLGNV |
| SRR1965862 | VVTRRTIFELRKARDRAHILEALAIALANIDPIIELIRRAPTPAEAKAALISRPWDLGNV |
| SRR1967363 | VVTRRTIFELRKARDRAHILEALAIALANIDPIIELIRRAPTPAEAKAALISRPWDLGNV |
| SRR1968276 | VVTRRTIFELRKARDRAHILEALAIALANIDPIIELIRRAPTPAEAKAALISRPWDLGNV |
| SRR1968967 | VVTRRTIFELRKARDRAHILEALAIALANIDPIIELIRRAPTPAEAKAALISRPWDLGNV |
| SRR3321531 | VVTRRTIFELRKARDRAHILEALAIALANIDPIIELIRRAPTPAEAKAALISRPWDLGNV |
| SRR3321883 | VVTRRTIFELRKARDRAHILEALAIALANIDPIIELIRRAPTPAEAKAALISRPWDLGNV |
| SRR3322413 | VVTRRTIFELRKARDRAHILEALAIALANIDPIIELIRRAPTPAEAKAALISRPWDLGNV |
| SRR3323012 | VVTRRTIFELRKARDRAHILEALAIALANIDPIIELIRRAPTPAEAKAALISRPWDLGNV |
| SRR5194289 | VVTRRTIFELRKARDRAHILEALAIALANIDPIIELIRRAPTPAEAKAALISRPWDLGNV |
| SRR7163798 | VVTRRTIFELRKARDRAHILEALAIALANIDPIIELIRRAPTPAEAKAALISRPWDLGNV |
| SRR7172610 | VVTRRTIFELRKARDRAHILEALAIALANIDPIIELIRRAPTPAEAKAALISRPWDLGNV |
| SRR7204568 | VVTRRTIFELRKARDRAHILEALAIALANIDPIIELIRRAPTPAEAKAALISRPWDLGNV |
| SRR7223230 | VVTRRTIFELRKARDRAHILEALAIALANIDPIIELIRRAPTPAEAKAALISRPWDLGNV |
| SRR7230675 | VVTRRTIFELRKARDRAHILEALAIALANIDPIIELIRRAPTPAEAKAALISRPWDLGNV |
| SRR7278056 | VVTRRTIFELRKARDRAHILEALAIALANIDPIIELIRRAPTPAEAKAALISRPWDLGNV |
| SRR7278086 | VVTRRTIFELRKARDRAHILEALAIALANIDPIIELIRRAPTPAEAKAALISRPWDLGNV |
| SRR7285841 | VVTRRTIFELRKARDRAHILEALAIALANIDPIIELIRRAPTPAEAKAALISRPWDLGNV |
| SRR7292625 | VVTRRTIFELRKARDRAHILEALAIALANIDPIIELIRRAPTPAEAKAALISRPWDLGNV |
| SRR7292665 | VVTRRTIFELRKARDRAHILEALAIALANIDPIIELIRRAPTPAEAKAALISRPWDLGNV |
| SRR7297965 | VVTRRTIFELRKARDRAHILEALAIALANIDPIIELIRRAPTPAEAKAALISRPWDLGNV |
| SRR7350726 | VVTRRTIFELRKARDRAHILEALAIALANIDPIIELIRRAPTPAEAKAALISRPWDLGNV |
| SRR7410328 | VVTRRTIFELRKARDRAHILEALAIALANIDPIIELIRRAPTPAEAKAALISRPWDLGNV |
| SRR7474665 | VVTRRTIFELRKARDRAHILEALAIALANIDPIIELIRRAPTPAEAKAALISRPWDLGNV |
| SRR7523184 | VVTRRTIFELRKARDRAHILEALAIALANIDPIIELIRRAPTPAEAKAALISRPWDLGNV |
| SRR7187264 | VVTRRTIFELRKARDRAHILEALAIALANIDPIIELIRRAPTPAEAKAALISRPWDLGNV |
| SRR7204445 | VVTRRTIFELRKARDRAHILEALAIALANIDPIIELIRRAPTPAEAKAALISRPWDLGNV |
| SRR7285641 | VVTRRTIFELRKARDRAHILEALAIALANIDPIIELIRRAPTPAEAKAALISRPWDLGNV |
| SRR7286695 | VVTRRTIFELRKARDRAHILEALAIALANIDPIIELIRRAPTPAEAKAALISRPWDLGNV |
| SRR7286705 | VVTRRTIFELRKARDRAHILEALAIALANIDPIIELIRRAPTPAEAKAALISRPWDLGNV |
| SRR7292931 | VVTRRTIFELRKARDRAHILEALAIALANIDPIIELIRRAPTPAEAKAALISRPWDLGNV |
| SRR7310349 | VVTRRTIFELRKARDRAHILEALAIALANIDPIIELIRRAPTPAEAKAALISRPWDLGNV |
| SRR7351616 | VVTRRTIFELRKARDRAHILEALAIALANIDPIIELIRRAPTPAEAKAALISRPWDLGNV |
| SRR7414818 | VVTRRTIFELRKARDRAHILEALAIALANIDPIIELIRRAPTPAEAKAALISRPWDLGNV |
| SRR7426480 | VVTRRTIFELRKARDRAHILEALAIALANIDPIIELIRRAPTPAEAKAALISRPWDLGNV |
| SRR5584105 | VVTRRTIFELRKARDRAHILEALAIALANIDPIIELIRRAPTPAEAKAALISRPWDLGNV |
| SRR5584565 | VVTRRTIFELRKARDRAHILEALAIALANIDPIIELIRRAPTPAEAKAALISRPWDLGNV |
| SRR5584614 | VVTRRTIFELRKARDRAHILEALAIALANIDPIIELIRRAPTPAEAKAALISRPWDLGNV |
| SRR5631543 | VVTRRTIFELRKARDRAHILEALAIALANIDPIIELIRRAPTPAEAKAALISRPWDLGNV |
| SRR5631553 | VVTRRTIFELRKARDRAHILEALAIALANIDPIIELIRRAPTPAEAKAALISRPWDLGNV |
| SRR7123196 | VVTRRTIFELRKARDRAHILEALAIALANIDPIIELIRRAPTPAEAKAALISRPWDLGNV |
| SRR7163819 | VVTRRTIFELRKARDRAHILEALAIALANIDPIIELIRRAPTPAEAKAALISRPWDLGNV |
| SRR7163920 | VVTRRTIFELRKARDRAHILEALAIALANIDPIIELIRRAPTPAEAKAALISRPWDLGNV |
| SRR7209528 | VVTRRTIFELRKARDRAHILEALAIALANIDPIIELIRRAPTPAEAKAALISRPWDLGNV |
| SRR7249868 | VVTRRTIFELRKARDRAHILEALAIALANIDPIIELIRRAPTPAEAKAALISRPWDLGNV |
| SRR7278088 | VVTRRTIFELRKARDRAHILEALAIALANIDPIIELIRRAPTPAEAKAALISRPWDLGNV |
| SRR7285788 | VVTRRTIFELRKARDRAHILEALAIALANIDPIIELIRRAPTPAEAKAALISRPWDLGNV |
| SRR7286789 | VVTRRTIFELRKARDRAHILEALAIALANIDPIIELIRRAPTPAEAKAALISRPWDLGNV |
| SRR7286886 | VVTRRTIFELRKARDRAHILEALAIALANIDPIIELIRRAPTPAEAKAALISRPWDLGNV |
| SRR7310632 | VVTRRTIFELRKARDRAHILEALAIALANIDPIIELIRRAPTPAEAKAALISRPWDLGNV |
| SRR7350631 | VVTRRTIFELRKARDRAHILEALAIALANIDPIIELIRRAPTPAEAKAALISRPWDLGNV |
| SRR7458741 | VVTRRTIFELRKARDRAHILEALAIALANIDPIIELIRRAPTPAEAKAALISRPWDLGNV |
| SRR7480280 | VVTRRTIFELRKARDRAHILEALAIALANIDPIIELIRRAPTPAEAKAALISRPWDLGNV |
| SRR7523660 | VVTRRTIFELRKARDRAHILEALAIALANIDPIIELIRRAPTPAEAKAALISRPWDLGNV |
| SRR7523775 | VVTRRTIFELRKARDRAHILEALAIALANIDPIIELIRRAPTPAEAKAALISRPWDLGNV |
| SRR7251101 | VVTRRTIFELRKARDRAHILEALAIALANIDPIIELIRRAPTPAEAKAALISRPWDLGNV |
| SRR7284299 | VVTRRTIFELRKARDRAHILEALAIALANIDPIIELIRRAPTPAEAKAALISRPWDLGNV |
| SRR7285738 | VVTRRTIFELRKARDRAHILEALAIALANIDPIIELIRRAPTPAEAKAALISRPWDLGNV |
| SRR7310640 | VVTRRTIFELRKARDRAHILEALAIALANIDPIIELIRRAPTPAEAKAALISRPWDLGNV |
| SRR7349159 | VVTRRTIFELRKARDRAHILEALAIALANIDPIIELIRRAPTPAEAKAALISRPWDLGNV |
| SRR7474873 | VVTRRTIFELRKARDRAHILEALAIALANIDPIIELIRRAPTPAEAKAALISRPWDLGNV |
| SRR7495689 | VVTRRTIFELRKARDRAHILEALAIALANIDPIIELIRRAPTPAEAKAALISRPWDLGNV |
| SRR7495752 | VVTRRTIFELRKARDRAHILEALAIALANIDPIIELIRRAPTPAEAKAALISRPWDLGNV |
| ----------------------------------------------------------------------------- | |
| S16BD08730 | AAMLERAGDDAARPEWLEPEFGVRDGQYYLTEQQAQAILDLRLQKLTGLEHEKLLDEYKE |
| S18BD00684 | AAMLERAGDDAARPEWLEPEFGVRDGQYYLTEQQAQAILDLRLQKLTGLEHEKLLDEYKE |
| S18BD03994 | AAMLERAGDDAARPEWLEPEFGVRDGQYYLTEQQAQAILDLRLQKLTGLEHEKLLDEYKE |
| S18BD05011 | AAMLERAGDDAARPEWLEPEFGVRDGQYYLTEQQAQAILDLRLQKLTGLEHEKLLDEYKE |
| RKI_16-03723 | AAMLERAGDDAARPEWLEPEFGVRDGQYYLTEQQAQAILDLRLQKLTGLEHEKLLDEYKE |
| RKI_16-04315 | AAMLERAGDDAARPEWLEPEFGVRDGQYYLTEQQAQAILDLRLQKLTGLEHEKLLDEYKE |
| RKI_17-02304 | AAMLERAGDDAARPEWLEPEFGVRDGQYYLTEQQAQAILDLRLQKLTGLEHEKLLDEYKE |
| RKI_17-02411 | AAMLERAGDDAARPEWLEPEFGVRDGQYYLTEQQAQAILDLRLQKLTGLEHEKLLDEYKE |
| RKI_17-02757 | AAMLERAGDDAARPEWLEPEFGVRDGQYYLTEQQAQAILDLRLQKLTGLEHEKLLDEYKE |
| RKI_17-04797 | AAMLERAGDDAARPEWLEPEFGVRDGQYYLTEQQAQAILDLRLQKLTGLEHEKLLDEYKE |
| RKI_17-06869 | AAMLERAGDDAARPEWLEPEFGVRDGQYYLTEQQAQAILDLRLQKLTGLEHEKLLDEYKE |
| ERR2580277 | AAMLERAGDDAARPEWLEPEFGVRDGQYYLTEQQAQAILDLRLQKLTGLEHEKLLDEYKE |
| ERR2580276 | AAMLERAGDDAARPEWLEPEFGVRDGQYYLTEQQAQAILDLRLQKLTGLEHEKLLDEYKE |
| ERR2580273 | AAMLERAGDDAARPEWLEPEFGVRDGQYYLTEQQAQAILDLRLQKLTGLEHEKLLDEYKE |
| ERR2580274 | AAMLERAGDDAARPEWLEPEFGVRDGQYYLTEQQAQAILDLRLQKLTGLEHEKLLDEYKE |
| ERR2173656 | AAMLERAGDDAARPEWLEPEFGVRDGQYYLTEQQAQAILDLRLQKLTGLEHEKLLDEYKE |
| 17041676 | AAMLERAGDDAARPEWLEPEFGVRDGQYYLTEQQAQAILDLRLQKLTGLEHEKLLDEYKE |
| MT16-000061 | AAMLERAGDDAARPEWLEPEFGVRDGQYYLTEQQAQAILDLRLQKLTGLEHEKLLDEYKE |
| MT16-019416 | AAMLERAGDDAARPEWLEPEFGVRDGQYYLTEQQAQAILDLRLQKLTGLEHEKLLDEYKE |
| MT16-027865 | AAMLERAGDDAARPEWLEPEFGVRDGQYYLTEQQAQAILDLRLQKLTGLEHEKLLDEYKE |
| MT16-031693 | AAMLERAGDDAARPEWLEPEFGVRDGQYYLTEQQAQAILDLRLQKLTGLEHEKLLDEYKE |
| MT16-040253 | AAMLERAGDDAARPEWLEPEFGVRDGQYYLTEQQAQAILDLRLQKLTGLEHEKLLDEYKE |
| MT16-045379 | AAMLERAGDDAARPEWLEPEFGVRDGQYYLTEQQAQAILDLRLQKLTGLEHEKLLDEYKE |
| MT16-442728 | AAMLERAGDDAARPEWLEPEFGVRDGQYYLTEQQAQAILDLRLQKLTGLEHEKLLDEYKE |
| MT16-462857 | AAMLERAGDDAARPEWLEPEFGVRDGQYYLTEQQAQAILDLRLQKLTGLEHEKLLDEYKE |
| MT16-480196 | AAMLERAGDDAARPEWLEPEFGVRDGQYYLTEQQAQAILDLRLQKLTGLEHEKLLDEYKE |
| MT16-861555 | AAMLERAGDDAARPEWLEPEFGVRDGQYYLTEQQAQAILDLRLQKLTGLEHEKLLDEYKE |
| MT17-076833 | AAMLERAGDDAARPEWLEPEFGVRDGQYYLTEQQAQAILDLRLQKLTGLEHEKLLDEYKE |
| MT17-110677 | AAMLERAGDDAARPEWLEPEFGVRDGQYYLTEQQAQAILDLRLQKLTGLEHEKLLDEYKE |
| MT17-131730 | AAMLERAGDDAARPEWLEPEFGVRDGQYYLTEQQAQAILDLRLQKLTGLEHEKLLDEYKE |
| MT17-140890 | AAMLERAGDDAARPEWLEPEFGVRDGQYYLTEQQAQAILDLRLQKLTGLEHEKLLDEYKE |
| MT17-141840 | AAMLERAGDDAARPEWLEPEFGVRDGQYYLTEQQAQAILDLRLQKLTGLEHEKLLDEYKE |
| MT17-152488 | AAMLERAGDDAARPEWLEPEFGVRDGQYYLTEQQAQAILDLRLQKLTGLEHEKLLDEYKE |
| MT17-157311 | AAMLERAGDDAARPEWLEPEFGVRDGQYYLTEQQAQAILDLRLQKLTGLEHEKLLDEYKE |
| MT17-161645 | AAMLERAGDDAARPEWLEPEFGVRDGQYYLTEQQAQAILDLRLQKLTGLEHEKLLDEYKE |
| MT17-167951 | AAMLERAGDDAARPEWLEPEFGVRDGQYYLTEQQAQAILDLRLQKLTGLEHEKLLDEYKE |
| MT18-217732 | AAMLERAGDDAARPEWLEPEFGVRDGQYYLTEQQAQAILDLRLQKLTGLEHEKLLDEYKE |
| MT18-252580 | AAMLERAGDDAARPEWLEPEFGVRDGQYYLTEQQAQAILDLRLQKLTGLEHEKLLDEYKE |
| RIVM_H_2009-01 | AAMLERAGDDAARPEWLEPEFGVRDGQYYLTEQQAQAILDLRLQKLTGLEHEKLLDEYKE |
| RIVM_H_2010-01 | AAMLERAGDDAARPEWLEPEFGVRDGQYYLTEQQAQAILDLRLQKLTGLEHEKLLDEYKE |
| RIVM_H_2010-02 | AAMLERAGDDAARPEWLEPEFGVRDGQYYLTEQQAQAILDLRLQKLTGLEHEKLLDEYKE |
| RIVM_H_2011-01 | AAMLERAGDDAARPEWLEPEFGVRDGQYYLTEQQAQAILDLRLQKLTGLEHEKLLDEYKE |
| RIVM_H_2011-02 | AAMLERAGDDAARPEWLEPEFGVRDGQYYLTEQQAQAILDLRLQKLTGLEHEKLLDEYKE |
| RIVM_H_2011-03 | AAMLERAGDDAARPEWLEPEFGVRDGQYYLTEQQAQAILDLRLQKLTGLEHEKLLDEYKE |
| RIVM_H_2013-01 | AAMLERAGDDAARPEWLEPEFGVRDGQYYLTEQQAQAILDLRLQKLTGLEHEKLLDEYKE |
| RIVM_H_2013-02 | AAMLERAGDDAARPEWLEPEFGVRDGQYYLTEQQAQAILDLRLQKLTGLEHEKLLDEYKE |
| RIVM_H_2014-01 | AAMLERAGDDAARPEWLEPEFGVRDGQYYLTEQQAQAILDLRLQKLTGLEHEKLLDEYKE |
| RIVM_H_2014-02 | AAMLERAGDDAARPEWLEPEFGVRDGQYYLTEQQAQAILDLRLQKLTGLEHEKLLDEYKE |
| RIVM_H_2016-01 | AAMLERAGDDAARPEWLEPEFGVRDGQYYLTEQQAQAILDLRLQKLTGLEHEKLLDEYKE |
| RIVM_H_2016-02 | AAMLERAGDDAARPEWLEPEFGVRDGQYYLTEQQAQAILDLRLQKLTGLEHEKLLDEYKE |
| RIVM_H_2016-03 | AAMLERAGDDAARPEWLEPEFGVRDGQYYLTEQQAQAILDLRLQKLTGLEHEKLLDEYKE |
| RIVM_H_2016-04 | AAMLERAGDDAARPEWLEPEFGVRDGQYYLTEQQAQAILDLRLQKLTGLEHEKLLDEYKE |
| RIVM_H_2016-05 | AAMLERAGDDAARPEWLEPEFGVRDGQYYLTEQQAQAILDLRLQKLTGLEHEKLLDEYKE |
| RIVM_H_2016-06 | AAMLERAGDDAARPEWLEPEFGVRDGQYYLTEQQAQAILDLRLQKLTGLEHEKLLDEYKE |
| RIVM_H_2016-07 | AAMLERAGDDAARPEWLEPEFGVRDGQYYLTEQQAQAILDLRLQKLTGLEHEKLLDEYKE |
| RIVM_H_2016-08 | AAMLERAGDDAARPEWLEPEFGVRDGQYYLTEQQAQAILDLRLQKLTGLEHEKLLDEYKE |
| RIVM_H_2016-09 | AAMLERAGDDAARPEWLEPEFGVRDGQYYLTEQQAQAILDLRLQKLTGLEHEKLLDEYKE |
| RIVM_H_2016-10 | AAMLERAGDDAARPEWLEPEFGVRDGQYYLTEQQAQAILDLRLQKLTGLEHEKLLDEYKE |
| RIVM_H_2016-11 | AAMLERAGDDAARPEWLEPEFGVRDGQYYLTEQQAQAILDLRLQKLTGLEHEKLLDEYKE |
| RIVM_H_2016-12 | AAMLERAGDDAARPEWLEPEFGVRDGQYYLTEQQAQAILDLRLQKLTGLEHEKLLDEYKE |
| RIVM_H_2016-13 | AAMLERAGDDAARPEWLEPEFGVRDGQYYLTEQQAQAILDLRLQKLTGLEHEKLLDEYKE |
| RIVM_H_2016-14 | AAMLERAGDDAARPEWLEPEFGVRDGQYYLTEQQAQAILDLRLQKLTGLEHEKLLDEYKE |
| RIVM_H_2016-15 | AAMLERAGDDAARPEWLEPEFGVRDGQYYLTEQQAQAILDLRLQKLTGLEHEKLLDEYKE |
| RIVM_H_2017-01 | AAMLERAGDDAARPEWLEPEFGVRDGQYYLTEQQAQAILDLRLQKLTGLEHEKLLDEYKE |
| RIVM_H_2017-02 | AAMLERAGDDAARPEWLEPEFGVRDGQYYLTEQQAQAILDLRLQKLTGLEHEKLLDEYKE |
| RIVM_H_2017-03 | AAMLERAGDDAARPEWLEPEFGVRDGQYYLTEQQAQAILDLRLQKLTGLEHEKLLDEYKE |
| RIVM_H_2017-04 | AAMLERAGDDAARPEWLEPEFGVRDGQYYLTEQQAQAILDLRLQKLTGLEHEKLLDEYKE |
| RIVM_H_2017-05 | AAMLERAGDDAARPEWLEPEFGVRDGQYYLTEQQAQAILDLRLQKLTGLEHEKLLDEYKE |
| RIVM_H_2017-06 | AAMLERAGDDAARPEWLEPEFGVRDGQYYLTEQQAQAILDLRLQKLTGLEHEKLLDEYKE |
| RIVM_H_2017-07 | AAMLERAGDDAARPEWLEPEFGVRDGQYYLTEQQAQAILDLRLQKLTGLEHEKLLDEYKE |
| RIVM_H_2017-08 | AAMLERAGDDAARPEWLEPEFGVRDGQYYLTEQQAQAILDLRLQKLTGLEHEKLLDEYKE |
| RIVM_H_2017-09 | AAMLERAGDDAARPEWLEPEFGVRDGQYYLTEQQAQAILDLRLQKLTGLEHEKLLDEYKE |
| RIVM_H_2017-10 | AAMLERAGDDAARPEWLEPEFGVRDGQYYLTEQQAQAILDLRLQKLTGLEHEKLLDEYKE |
| RIVM_H_2017-11 | AAMLERAGDDAARPEWLEPEFGVRDGQYYLTEQQAQAILDLRLQKLTGLEHEKLLDEYKE |
| RIVM_H_2017-12 | AAMLERAGDDAARPEWLEPEFGVRDGQYYLTEQQAQAILDLRLQKLTGLEHEKLLDEYKE |
| RIVM_H_2017-13 | AAMLERAGDDAARPEWLEPEFGVRDGQYYLTEQQAQAILDLRLQKLTGLEHEKLLDEYKE |
| RIVM_H_2017-14 | AAMLERAGDDAARPEWLEPEFGVRDGQYYLTEQQAQAILDLRLQKLTGLEHEKLLDEYKE |
| RIVM_H_2017-15 | AAMLERAGDDAARPEWLEPEFGVRDGQYYLTEQQAQAILDLRLQKLTGLEHEKLLDEYKE |
| RIVM_H_2017-16 | AAMLERAGDDAARPEWLEPEFGVRDGQYYLTEQQAQAILDLRLQKLTGLEHEKLLDEYKE |
| RIVM_H_2017-17 | AAMLERAGDDAARPEWLEPEFGVRDGQYYLTEQQAQAILDLRLQKLTGLEHEKLLDEYKE |
| RIVM_H_2017-18 | AAMLERAGDDAARPEWLEPEFGVRDGQYYLTEQQAQAILDLRLQKLTGLEHEKLLDEYKE |
| RIVM_H_2017-19 | AAMLERAGDDAARPEWLEPEFGVRDGQYYLTEQQAQAILDLRLQKLTGLEHEKLLDEYKE |
| 15EP001483 | AAMLERAGDDAARPEWLEPEFGVRDGQYYLTEQQAQAILDLRLQKLTGLEHEKLLDEYKE |
| 17EP002363 | AAMLERAGDDAARPEWLEPEFGVRDGQYYLTEQQAQAILDLRLQKLTGLEHEKLLDEYKE |
| S_0812_17 | AAMLERAGDDAARPEWLEPEFGVRDGQYYLTEQQAQAILDLRLQKLTGLEHEKLLDEYKE |
| SRR1957844 | AAMLERAGDDAARPEWLEPEFGVRDGQYYLTEQQAQAILDLRLQKLTGLEHEKLLDEYKE |
| SRR1958654 | AAMLERAGDDAARPEWLEPEFGVRDGQYYLTEQQAQAILDLRLQKLTGLEHEKLLDEYKE |
| SRR1965077 | AAMLERAGDDAARPEWLEPEFGVRDGQYYLTEQQAQAILDLRLQKLTGLEHEKLLDEYKE |
| SRR1966369 | AAMLERAGDDAARPEWLEPEFGVRDGQYYLTEQQAQAILDLRLQKLTGLEHEKLLDEYKE |
| SRR1967117 | AAMLERAGDDAARPEWLEPEFGVRDGQYYLTEQQAQAILDLRLQKLTGLEHEKLLDEYKE |
| SRR1967922 | AAMLERAGDDAARPEWLEPEFGVRDGQYYLTEQQAQAILDLRLQKLTGLEHEKLLDEYKE |
| SRR8704720 | AAMLERAGDDAARPEWLEPEFGVRDGQYYLTEQQAQAILDLRLQKLTGLEHEKLLDEYKE |
| SRR7216071 | AAMLERAGDDAARPEWLEPEFGVRDGQYYLTEQQAQAILDLRLQKLTGLEHEKLLDEYKE |
| SRR7349175 | AAMLERAGDDAARPEWLEPEFGVRDGQYYLTEQQAQAILDLRLQKLTGLEHEKLLDEYKE |
| SRR7523148 | AAMLERAGDDAARPEWLEPEFGVRDGQYYLTEQQAQAILDLRLQKLTGLEHEKLLDEYKE |
| SRR7523854 | AAMLERAGDDAARPEWLEPEFGVRDGQYYLTEQQAQAILDLRLQKLTGLEHEKLLDEYKE |
| 313865 | AAMLERAGDDAARPEWLEPEFGVRDGQYYLTEQQAQAILDLRLQKLTGLEHEKLLDEYKE |
| SRR7277793 | AAMLERAGDDAARPEWLEPEFGVRDGQYYLTEQQAQAILDLRLQKLTGLEHEKLLDEYKE |
| SRR7343877 | AAMLERAGDDAARPEWLEPEFGVRDGQYYLTEQQAQAILDLRLQKLTGLEHEKLLDEYKE |
| SRR7351477 | AAMLERAGDDAARPEWLEPEFGVRDGQYYLTEQQAQAILDLRLQKLTGLEHEKLLDEYKE |
| SRR5583183 | AAMLERAGDDAARPEWLEPEFGVRDGQYYLTEQQAQAILDLRLQKLTGLEHEKLLDEYKE |
| SRR5585240 | AAMLERAGDDAARPEWLEPEFGVRDGQYYLTEQQAQAILDLRLQKLTGLEHEKLLDEYKE |
| SRR7284317 | AAMLERAGDDAARPEWLEPEFGVRDGQYYLTEQQAQAILDLRLQKLTGLEHEKLLDEYKE |
| SRR7299161 | AAMLERAGDDAARPEWLEPEFGVRDGQYYLTEQQAQAILDLRLQKLTGLEHEKLLDEYKE |
| SRR7401730 | AAMLERAGDDAARPEWLEPEFGVRDGQYYLTEQQAQAILDLRLQKLTGLEHEKLLDEYKE |
| SRR7469092 | AAMLERAGDDAARPEWLEPEFGVRDGQYYLTEQQAQAILDLRLQKLTGLEHEKLLDEYKE |
| SRR7879556 | AAMLERAGDDAARPEWLEPEFGVRDGQYYLTEQQAQAILDLRLQKLTGLEHEKLLDEYKE |
| SRR8526100 | AAMLERAGDDAARPEWLEPEFGVRDGQYYLTEQQAQAILDLRLQKLTGLEHEKLLDEYKE |
| SRR8553991 | AAMLERAGDDAARPEWLEPEFGVRDGQYYLTEQQAQAILDLRLQKLTGLEHEKLLDEYKE |
| SRR7842487 | AAMLERAGDDAARPEWLEPEFGVRDGQYYLTEQQAQAILDLRLQKLTGLEHEKLLDEYKE |
| SRR8054524 | AAMLERAGDDAARPEWLEPEFGVRDGQYYLTEQQAQAILDLRLQKLTGLEHEKLLDEYKE |
| SRR8054525 | AAMLERAGDDAARPEWLEPEFGVRDGQYYLTEQQAQAILDLRLQKLTGLEHEKLLDEYKE |
| SRR8524733 | AAMLERAGDDAARPEWLEPEFGVRDGQYYLTEQQAQAILDLRLQKLTGLEHEKLLDEYKE |
| SRR4093291 | AAMLERAGDDAARPEWLEPEFGVRDGQYYLTEQQAQAILDLRLQKLTGLEHEKLLDEYKE |
| SRR4245549 | AAMLERAGDDAARPEWLEPEFGVRDGQYYLTEQQAQAILDLRLQKLTGLEHEKLLDEYKE |
| SRR3057154 | AAMLERAGDDAARPEWLEPEFGVRDGQYYLTEQQAQAILDLRLQKLTGLEHEKLLDEYKE |
| SRR1726150 | AAMLERAGDDAARPEWLEPEFGVRDGQYYLTEQQAQAILDLRLQKLTGLEHEKLLDEYKE |
| SRR1996141 | AAMLERAGDDAARPEWLEPEFGVRDGQYYLTEQQAQAILDLRLQKLTGLEHEKLLDEYKE |
| SRR1107842 | AAMLERAGDDAARPEWLEPEFGVRDGQYYLTEQQAQAILDLRLQKLTGLEHEKLLDEYKE |
| SRR1157587 | AAMLERAGDDAARPEWLEPEFGVRDGQYYLTEQQAQAILDLRLQKLTGLEHEKLLDEYKE |
| SRR3027706 | AAMLERAGDDAARPEWLEPEFGVRDGQYYLTEQQAQAILDLRLQKLTGLEHEKLLDEYKE |
| SRR3027707 | AAMLERAGDDAARPEWLEPEFGVRDGQYYLTEQQAQAILDLRLQKLTGLEHEKLLDEYKE |
| SRR3027708 | AAMLERAGDDAARPEWLEPEFGVRDGQYYLTEQQAQAILDLRLQKLTGLEHEKLLDEYKE |
| SRR3027710 | AAMLERAGDDAARPEWLEPEFGVRDGQYYLTEQQAQAILDLRLQKLTGLEHEKLLDEYKE |
| SRR3027711 | AAMLERAGDDAARPEWLEPEFGVRDGQYYLTEQQAQAILDLRLQKLTGLEHEKLLDEYKE |
| SRR3027716 | AAMLERAGDDAARPEWLEPEFGVRDGQYYLTEQQAQAILDLRLQKLTGLEHEKLLDEYKE |
| SRR3027717 | AAMLERAGDDAARPEWLEPEFGVRDGQYYLTEQQAQAILDLRLQKLTGLEHEKLLDEYKE |
| SRR3027719 | AAMLERAGDDAARPEWLEPEFGVRDGQYYLTEQQAQAILDLRLQKLTGLEHEKLLDEYKE |
| SRR3027721 | AAMLERAGDDAARPEWLEPEFGVRDGQYYLTEQQAQAILDLRLQKLTGLEHEKLLDEYKE |
| SRR3027723 | AAMLERAGDDAARPEWLEPEFGVRDGQYYLTEQQAQAILDLRLQKLTGLEHEKLLDEYKE |
| SRR3115978 | AAMLERAGDDAARPEWLEPEFGVRDGQYYLTEQQAQAILDLRLQKLTGLEHEKLLDEYKE |
| SRR2534093 | AAMLERAGDDAARPEWLEPEFGVRDGQYYLTEQQAQAILDLRLQKLTGLEHEKLLDEYKE |
| SRR2534094 | AAMLERAGDDAARPEWLEPEFGVRDGQYYLTEQQAQAILDLRLQKLTGLEHEKLLDEYKE |
| SRR2534095 | AAMLERAGDDAARPEWLEPEFGVRDGQYYLTEQQAQAILDLRLQKLTGLEHEKLLDEYKE |
| SRR2534108 | AAMLERAGDDAARPEWLEPEFGVRDGQYYLTEQQAQAILDLRLQKLTGLEHEKLLDEYKE |
| SRR1106464 | AAMLERAGDDAARPEWLEPEFGVRDGQYYLTEQQAQAILDLRLQKLTGLEHEKLLDEYKE |
| SRR1106463 | AAMLERAGDDAARPEWLEPEFGVRDGQYYLTEQQAQAILDLRLQKLTGLEHEKLLDEYKE |
| SRR6949610 | AAMLERAGDDAARPEWLEPEFGVRDGQYYLTEQQAQAILDLRLQKLTGLEHEKLLDEYKE |
| SRR6950452 | AAMLERAGDDAARPEWLEPEFGVRDGQYYLTEQQAQAILDLRLQKLTGLEHEKLLDEYKE |
| ERR2019831 | AAMLERAGDDAARPEWLEPEFGVRDGQYYLTEQQAQAILDLRLQKLTGLEHEKLLDEYKE |
| SRR2085693 | AAMLERAGDDAARPEWLEPEFGVRDGQYYLTEQQAQAILDLRLQKLTGLEHEKLLDEYKE |
| SRR2086898 | AAMLERAGDDAARPEWLEPEFGVRDGQYYLTEQQAQAILDLRLQKLTGLEHEKLLDEYKE |
| SRR2175312 | AAMLERAGDDAARPEWLEPEFGVRDGQYYLTEQQAQAILDLRLQKLTGLEHEKLLDEYKE |
| SRR2175360 | AAMLERAGDDAARPEWLEPEFGVRDGQYYLTEQQAQAILDLRLQKLTGLEHEKLLDEYKE |
| SRR5231997 | AAMLERAGDDAARPEWLEPEFGVRDGQYYLTEQQAQAILDLRLQKLTGLEHEKLLDEYKE |
| SRR5232003 | AAMLERAGDDAARPEWLEPEFGVRDGQYYLTEQQAQAILDLRLQKLTGLEHEKLLDEYKE |
| SRR5232015 | AAMLERAGDDAARPEWLEPEFGVRDGQYYLTEQQAQAILDLRLQKLTGLEHEKLLDEYKE |
| SRR949434 | AAMLERAGDDAARPEWLEPEFGVRDGQYYLTEQQAQAILDLRLQKLTGLEHEKLLDEYKE |
| SRR3216575 | AAMLERAGDDAARPEWLEPEFGVRDGQYYLTEQQAQAILDLRLQKLTGLEHEKLLDEYKE |
| SRR5205342 | AAMLERAGDDAARPEWLEPEFGVRDGQYYLTEQQAQAILDLRLQKLTGLEHEKLLDEYKE |
| SRR1501669 | AAMLERAGDDAARPEWLEPEFGVRDGQYYLTEQQAQAILDLRLQKLTGLEHEKLLDEYKE |
| SRR5209740 | AAMLERAGDDAARPEWLEPEFGVRDGQYYLTEQQAQAILDLRLQKLTGLEHEKLLDEYKE |
| SRR3240355 | AAMLERAGDDAARPEWLEPEFGVRDGQYYLTEQQAQAILDLRLQKLTGLEHEKLLDEYKE |
| SRR3392777 | AAMLERAGDDAARPEWLEPEFGVRDGQYYLTEQQAQAILDLRLQKLTGLEHEKLLDEYKE |
| SRR3593671 | AAMLERAGDDAARPEWLEPEFGVRDGQYYLTEQQAQAILDLRLQKLTGLEHEKLLDEYKE |
| SRR5413290 | AAMLERAGDDAARPEWLEPEFGVRDGQYYLTEQQAQAILDLRLQKLTGLEHEKLLDEYKE |
| SRR5590269 | AAMLERAGDDAARPEWLEPEFGVRDGQYYLTEQQAQAILDLRLQKLTGLEHEKLLDEYKE |
| SRR5812103 | AAMLERAGDDAARPEWLEPEFGVRDGQYYLTEQQAQAILDLRLQKLTGLEHEKLLDEYKE |
| SRR2830941 | AAMLERAGDDAARPEWLEPEFGVRDGQYYLTEQQAQAILDLRLQKLTGLEHEKLLDEYKE |
| SRR2830966 | AAMLERAGDDAARPEWLEPEFGVRDGQYYLTEQQAQAILDLRLQKLTGLEHEKLLDEYKE |
| SRR3137270 | AAMLERAGDDAARPEWLEPEFGVRDGQYYLTEQQAQAILDLRLQKLTGLEHEKLLDEYKE |
| SRR3137271 | AAMLERAGDDAARPEWLEPEFGVRDGQYYLTEQQAQAILDLRLQKLTGLEHEKLLDEYKE |
| ERR526807 | AAMLERAGDDAARPEWLEPEFGVRDGQYYLTEQQAQAILDLRLQKLTGLEHEKLLDEYKE |
| ERR2197922 | AAMLERAGDDAARPEWLEPEFGVRDGQYYLTEQQAQAILDLRLQKLTGLEHEKLLDEYKE |
| ERR2197923 | AAMLERAGDDAARPEWLEPEFGVRDGQYYLTEQQAQAILDLRLQKLTGLEHEKLLDEYKE |
| ERR2197924 | AAMLERAGDDAARPEWLEPEFGVRDGQYYLTEQQAQAILDLRLQKLTGLEHEKLLDEYKE |
| ERR2197925 | AAMLERAGDDAARPEWLEPEFGVRDGQYYLTEQQAQAILDLRLQKLTGLEHEKLLDEYKE |
| ERR2197927 | AAMLERAGDDAARPEWLEPEFGVRDGQYYLTEQQAQAILDLRLQKLTGLEHEKLLDEYKE |
| ERR2197929 | AAMLERAGDDAARPEWLEPEFGVRDGQYYLTEQQAQAILDLRLQKLTGLEHEKLLDEYKE |
| SRR1648149 | AAMLERAGDDAARPEWLEPEFGVRDGQYYLTEQQAQAILDLRLQKLTGLEHEKLLDEYKE |
| SRR1048299 | AAMLERAGDDAARPEWLEPEFGVRDGQYYLTEQQAQAILDLRLQKLTGLEHEKLLDEYKE |
| SRR1300677 | AAMLERAGDDAARPEWLEPEFGVRDGQYYLTEQQAQAILDLRLQKLTGLEHEKLLDEYKE |
| SRR1288356 | AAMLERAGDDAARPEWLEPEFGVRDGQYYLTEQQAQAILDLRLQKLTGLEHEKLLDEYKE |
| SRR7426190 | AAMLERAGDDAARPEWLEPEFGVRDGQYYLTEQQAQAILDLRLQKLTGLEHEKLLDEYKE |
| SRR7426192 | AAMLERAGDDAARPEWLEPEFGVRDGQYYLTEQQAQAILDLRLQKLTGLEHEKLLDEYKE |
| SRR7426193 | AAMLERAGDDAARPEWLEPEFGVRDGQYYLTEQQAQAILDLRLQKLTGLEHEKLLDEYKE |
| SRR7441832 | AAMLERAGDDAARPEWLEPEFGVRDGQYYLTEQQAQAILDLRLQKLTGLEHEKLLDEYKE |
| SRR7426179 | AAMLERAGDDAARPEWLEPEFGVRDGQYYLTEQQAQAILDLRLQKLTGLEHEKLLDEYKE |
| SRR7439238 | AAMLERAGDDAARPEWLEPEFGVRDGQYYLTEQQAQAILDLRLQKLTGLEHEKLLDEYKE |
| SRR7439244 | AAMLERAGDDAARPEWLEPEFGVRDGQYYLTEQQAQAILDLRLQKLTGLEHEKLLDEYKE |
| SRR7439259 | AAMLERAGDDAARPEWLEPEFGVRDGQYYLTEQQAQAILDLRLQKLTGLEHEKLLDEYKE |
| SRR7439260 | AAMLERAGDDAARPEWLEPEFGVRDGQYYLTEQQAQAILDLRLQKLTGLEHEKLLDEYKE |
| SRR7441786 | AAMLERAGDDAARPEWLEPEFGVRDGQYYLTEQQAQAILDLRLQKLTGLEHEKLLDEYKE |
| SRR7441797 | AAMLERAGDDAARPEWLEPEFGVRDGQYYLTEQQAQAILDLRLQKLTGLEHEKLLDEYKE |
| ERR1759093 | AAMLERAGDDAARPEWLEPEFGVRDGQYYLTEQQAQAILDLRLQKLTGLEHEKLLDEYKE |
| ERR2580275 | AAMLERAGDDAARPEWLEPEFGVRDGQYYLTEQQAQAILDLRLQKLTGLEHEKLLDEYKE |
| ERR1759204 | AAMLERAGDDAARPEWLEPEFGVRDGQYYLTEQQAQAILDLRLQKLTGLEHEKLLDEYKE |
| SRR1300699 | AAMLERAGDDAARPEWLEPEFGVRDGQYYLTEQQAQAILDLRLQKLTGLEHEKLLDEYKE |
| S_0825_17 | AAMLERAGDDAARPEWLEPEFGVRDGQYYLTEQQAQAILDLRLQKLTGLEHEKLLDEYKE |
| SRR1958215 | AAMLERAGDDAARPEWLEPEFGVRDGQYYLTEQQAQAILDLRLQKLTGLEHEKLLDEYKE |
| SRR1958540 | AAMLERAGDDAARPEWLEPEFGVRDGQYYLTEQQAQAILDLRLQKLTGLEHEKLLDEYKE |
| SRR1958636 | AAMLERAGDDAARPEWLEPEFGVRDGQYYLTEQQAQAILDLRLQKLTGLEHEKLLDEYKE |
| SRR1959422 | AAMLERAGDDAARPEWLEPEFGVRDGQYYLTEQQAQAILDLRLQKLTGLEHEKLLDEYKE |
| SRR1959427 | AAMLERAGDDAARPEWLEPEFGVRDGQYYLTEQQAQAILDLRLQKLTGLEHEKLLDEYKE |
| SRR1960226 | AAMLERAGDDAARPEWLEPEFGVRDGQYYLTEQQAQAILDLRLQKLTGLEHEKLLDEYKE |
| SRR1963498 | AAMLERAGDDAARPEWLEPEFGVRDGQYYLTEQQAQAILDLRLQKLTGLEHEKLLDEYKE |
| SRR1965947 | AAMLERAGDDAARPEWLEPEFGVRDGQYYLTEQQAQAILDLRLQKLTGLEHEKLLDEYKE |
| SRR1966125 | AAMLERAGDDAARPEWLEPEFGVRDGQYYLTEQQAQAILDLRLQKLTGLEHEKLLDEYKE |
| SRR1966330 | AAMLERAGDDAARPEWLEPEFGVRDGQYYLTEQQAQAILDLRLQKLTGLEHEKLLDEYKE |
| SRR1966565 | AAMLERAGDDAARPEWLEPEFGVRDGQYYLTEQQAQAILDLRLQKLTGLEHEKLLDEYKE |
| SRR1966864 | AAMLERAGDDAARPEWLEPEFGVRDGQYYLTEQQAQAILDLRLQKLTGLEHEKLLDEYKE |
| SRR1966989 | AAMLERAGDDAARPEWLEPEFGVRDGQYYLTEQQAQAILDLRLQKLTGLEHEKLLDEYKE |
| SRR1967688 | AAMLERAGDDAARPEWLEPEFGVRDGQYYLTEQQAQAILDLRLQKLTGLEHEKLLDEYKE |
| SRR1967733 | AAMLERAGDDAARPEWLEPEFGVRDGQYYLTEQQAQAILDLRLQKLTGLEHEKLLDEYKE |
| SRR1967746 | AAMLERAGDDAARPEWLEPEFGVRDGQYYLTEQQAQAILDLRLQKLTGLEHEKLLDEYKE |
| SRR1968341 | AAMLERAGDDAARPEWLEPEFGVRDGQYYLTEQQAQAILDLRLQKLTGLEHEKLLDEYKE |
| SRR1968456 | AAMLERAGDDAARPEWLEPEFGVRDGQYYLTEQQAQAILDLRLQKLTGLEHEKLLDEYKE |
| SRR1968465 | AAMLERAGDDAARPEWLEPEFGVRDGQYYLTEQQAQAILDLRLQKLTGLEHEKLLDEYKE |
| SRR1968761 | AAMLERAGDDAARPEWLEPEFGVRDGQYYLTEQQAQAILDLRLQKLTGLEHEKLLDEYKE |
| SRR1969047 | AAMLERAGDDAARPEWLEPEFGVRDGQYYLTEQQAQAILDLRLQKLTGLEHEKLLDEYKE |
| SRR1969255 | AAMLERAGDDAARPEWLEPEFGVRDGQYYLTEQQAQAILDLRLQKLTGLEHEKLLDEYKE |
| SRR1969412 | AAMLERAGDDAARPEWLEPEFGVRDGQYYLTEQQAQAILDLRLQKLTGLEHEKLLDEYKE |
| SRR1969524 | AAMLERAGDDAARPEWLEPEFGVRDGQYYLTEQQAQAILDLRLQKLTGLEHEKLLDEYKE |
| SRR1969584 | AAMLERAGDDAARPEWLEPEFGVRDGQYYLTEQQAQAILDLRLQKLTGLEHEKLLDEYKE |
| SRR1969648 | AAMLERAGDDAARPEWLEPEFGVRDGQYYLTEQQAQAILDLRLQKLTGLEHEKLLDEYKE |
| SRR1969804 | AAMLERAGDDAARPEWLEPEFGVRDGQYYLTEQQAQAILDLRLQKLTGLEHEKLLDEYKE |
| SRR1970221 | AAMLERAGDDAARPEWLEPEFGVRDGQYYLTEQQAQAILDLRLQKLTGLEHEKLLDEYKE |
| SRR1970268 | AAMLERAGDDAARPEWLEPEFGVRDGQYYLTEQQAQAILDLRLQKLTGLEHEKLLDEYKE |
| SRR1965862 | AAMLERAGDDAARPEWLEPEFGVRDGQYYLTEQQAQAILDLRLQKLTGLEHEKLLDEYKE |
| SRR1967363 | AAMLERAGDDAARPEWLEPEFGVRDGQYYLTEQQAQAILDLRLQKLTGLEHEKLLDEYKE |
| SRR1968276 | AAMLERAGDDAARPEWLEPEFGVRDGQYYLTEQQAQAILDLRLQKLTGLEHEKLLDEYKE |
| SRR1968967 | AAMLERAGDDAARPEWLEPEFGVRDGQYYLTEQQAQAILDLRLQKLTGLEHEKLLDEYKE |
| SRR3321531 | AAMLERAGDDAARPEWLEPEFGVRDGQYYLTEQQAQAILDLRLQKLTGLEHEKLLDEYKE |
| SRR3321883 | AAMLERAGDDAARPEWLEPEFGVRDGQYYLTEQQAQAILDLRLQKLTGLEHEKLLDEYKE |
| SRR3322413 | AAMLERAGDDAARPEWLEPEFGVRDGQYYLTEQQAQAILDLRLQKLTGLEHEKLLDEYKE |
| SRR3323012 | AAMLERAGDDAARPEWLEPEFGVRDGQYYLTEQQAQAILDLRLQKLTGLEHEKLLDEYKE |
| SRR5194289 | AAMLERAGDDAARPEWLEPEFGVRDGQYYLTEQQAQAILDLRLQKLTGLEHEKLLDEYKE |
| SRR7163798 | AAMLERAGDDAARPEWLEPEFGVRDGQYYLTEQQAQAILDLRLQKLTGLEHEKLLDEYKE |
| SRR7172610 | AAMLERAGDDAARPEWLEPEFGVRDGQYYLTEQQAQAILDLRLQKLTGLEHEKLLDEYKE |
| SRR7204568 | AAMLERAGDDAARPEWLEPEFGVRDGQYYLTEQQAQAILDLRLQKLTGLEHEKLLDEYKE |
| SRR7223230 | AAMLERAGDDAARPEWLEPEFGVRDGQYYLTEQQAQAILDLRLQKLTGLEHEKLLDEYKE |
| SRR7230675 | AAMLERAGDDAARPEWLEPEFGVRDGQYYLTEQQAQAILDLRLQKLTGLEHEKLLDEYKE |
| SRR7278056 | AAMLERAGDDAARPEWLEPEFGVRDGQYYLTEQQAQAILDLRLQKLTGLEHEKLLDEYKE |
| SRR7278086 | AAMLERAGDDAARPEWLEPEFGVRDGQYYLTEQQAQAILDLRLQKLTGLEHEKLLDEYKE |
| SRR7285841 | AAMLERAGDDAARPEWLEPEFGVRDGQYYLTEQQAQAILDLRLQKLTGLEHEKLLDEYKE |
| SRR7292625 | AAMLERAGDDAARPEWLEPEFGVRDGQYYLTEQQAQAILDLRLQKLTGLEHEKLLDEYKE |
| SRR7292665 | AAMLERAGDDAARPEWLEPEFGVRDGQYYLTEQQAQAILDLRLQKLTGLEHEKLLDEYKE |
| SRR7297965 | AAMLERAGDDAARPEWLEPEFGVRDGQYYLTEQQAQAILDLRLQKLTGLEHEKLLDEYKE |
| SRR7350726 | AAMLERAGDDAARPEWLEPEFGVRDGQYYLTEQQAQAILDLRLQKLTGLEHEKLLDEYKE |
| SRR7410328 | AAMLERAGDDAARPEWLEPEFGVRDGQYYLTEQQAQAILDLRLQKLTGLEHEKLLDEYKE |
| SRR7474665 | AAMLERAGDDAARPEWLEPEFGVRDGQYYLTEQQAQAILDLRLQKLTGLEHEKLLDEYKE |
| SRR7523184 | AAMLERAGDDAARPEWLEPEFGVRDGQYYLTEQQAQAILDLRLQKLTGLEHEKLLDEYKE |
| SRR7187264 | AAMLERAGDDAARPEWLEPEFGVRDGQYYLTEQQAQAILDLRLQKLTGLEHEKLLDEYKE |
| SRR7204445 | AAMLERAGDDAARPEWLEPEFGVRDGQYYLTEQQAQAILDLRLQKLTGLEHEKLLDEYKE |
| SRR7285641 | AAMLERAGDDAARPEWLEPEFGVRDGQYYLTEQQAQAILDLRLQKLTGLEHEKLLDEYKE |
| SRR7286695 | AAMLERAGDDAARPEWLEPEFGVRDGQYYLTEQQAQAILDLRLQKLTGLEHEKLLDEYKE |
| SRR7286705 | AAMLERAGDDAARPEWLEPEFGVRDGQYYLTEQQAQAILDLRLQKLTGLEHEKLLDEYKE |
| SRR7292931 | AAMLERAGDDAARPEWLEPEFGVRDGQYYLTEQQAQAILDLRLQKLTGLEHEKLLDEYKE |
| SRR7310349 | AAMLERAGDDAARPEWLEPEFGVRDGQYYLTEQQAQAILDLRLQKLTGLEHEKLLDEYKE |
| SRR7351616 | AAMLERAGDDAARPEWLEPEFGVRDGQYYLTEQQAQAILDLRLQKLTGLEHEKLLDEYKE |
| SRR7414818 | AAMLERAGDDAARPEWLEPEFGVRDGQYYLTEQQAQAILDLRLQKLTGLEHEKLLDEYKE |
| SRR7426480 | AAMLERAGDDAARPEWLEPEFGVRDGQYYLTEQQAQAILDLRLQKLTGLEHEKLLDEYKE |
| SRR5584105 | AAMLERAGDDAARPEWLEPEFGVRDGQYYLTEQQAQAILDLRLQKLTGLEHEKLLDEYKE |
| SRR5584565 | AAMLERAGDDAARPEWLEPEFGVRDGQYYLTEQQAQAILDLRLQKLTGLEHEKLLDEYKE |
| SRR5584614 | AAMLERAGDDAARPEWLEPEFGVRDGQYYLTEQQAQAILDLRLQKLTGLEHEKLLDEYKE |
| SRR5631543 | AAMLERAGDDAARPEWLEPEFGVRDGQYYLTEQQAQAILDLRLQKLTGLEHEKLLDEYKE |
| SRR5631553 | AAMLERAGDDAARPEWLEPEFGVRDGQYYLTEQQAQAILDLRLQKLTGLEHEKLLDEYKE |
| SRR7123196 | AAMLERAGDDAARPEWLEPEFGVRDGQYYLTEQQAQAILDLRLQKLTGLEHEKLLDEYKE |
| SRR7163819 | AAMLERAGDDAARPEWLEPEFGVRDGQYYLTEQQAQAILDLRLQKLTGLEHEKLLDEYKE |
| SRR7163920 | AAMLERAGDDAARPEWLEPEFGVRDGQYYLTEQQAQAILDLRLQKLTGLEHEKLLDEYKE |
| SRR7209528 | AAMLERAGDDAARPEWLEPEFGVRDGQYYLTEQQAQAILDLRLQKLTGLEHEKLLDEYKE |
| SRR7249868 | AAMLERAGDDAARPEWLEPEFGVRDGQYYLTEQQAQAILDLRLQKLTGLEHEKLLDEYKE |
| SRR7278088 | AAMLERAGDDAARPEWLEPEFGVRDGQYYLTEQQAQAILDLRLQKLTGLEHEKLLDEYKE |
| SRR7285788 | AAMLERAGDDAARPEWLEPEFGVRDGQYYLTEQQAQAILDLRLQKLTGLEHEKLLDEYKE |
| SRR7286789 | AAMLERAGDDAARPEWLEPEFGVRDGQYYLTEQQAQAILDLRLQKLTGLEHEKLLDEYKE |
| SRR7286886 | AAMLERAGDDAARPEWLEPEFGVRDGQYYLTEQQAQAILDLRLQKLTGLEHEKLLDEYKE |
| SRR7310632 | AAMLERAGDDAARPEWLEPEFGVRDGQYYLTEQQAQAILDLRLQKLTGLEHEKLLDEYKE |
| SRR7350631 | AAMLERAGDDAARPEWLEPEFGVRDGQYYLTEQQAQAILDLRLQKLTGLEHEKLLDEYKE |
| SRR7458741 | AAMLERAGDDAARPEWLEPEFGVRDGQYYLTEQQAQAILDLRLQKLTGLEHEKLLDEYKE |
| SRR7480280 | AAMLERAGDDAARPEWLEPEFGVRDGQYYLTEQQAQAILDLRLQKLTGLEHEKLLDEYKE |
| SRR7523660 | AAMLERAGDDAARPEWLEPEFGVRDGQYYLTEQQAQAILDLRLQKLTGLEHEKLLDEYKE |
| SRR7523775 | AAMLERAGDDAARPEWLEPEFGVRDGQYYLTEQQAQAILDLRLQKLTGLEHEKLLDEYKE |
| SRR7251101 | AAMLERAGDDAARPEWLEPEFGVRDGQYYLTEQQAQAILDLRLQKLTGLEHEKLLDEYKE |
| SRR7284299 | AAMLERAGDDAARPEWLEPEFGVRDGQYYLTEQQAQAILDLRLQKLTGLEHEKLLDEYKE |
| SRR7285738 | AAMLERAGDDAARPEWLEPEFGVRDGQYYLTEQQAQAILDLRLQKLTGLEHEKLLDEYKE |
| SRR7310640 | AAMLERAGDDAARPEWLEPEFGVRDGQYYLTEQQAQAILDLRLQKLTGLEHEKLLDEYKE |
| SRR7349159 | AAMLERAGDDAARPEWLEPEFGVRDGQYYLTEQQAQAILDLRLQKLTGLEHEKLLDEYKE |
| SRR7474873 | AAMLERAGDDAARPEWLEPEFGVRDGQYYLTEQQAQAILDLRLQKLTGLEHEKLLDEYKE |
| SRR7495689 | AAMLERAGDDAARPEWLEPEFGVRDGQYYLTEQQAQAILDLRLQKLTGLEHEKLLDEYKE |
| SRR7495752 | AAMLERAGDDAARPEWLEPEFGVRDGQYYLTEQQAQAILDLRLQKLTGLEHEKLLDEYKE |
| ----------------------------------------------------------------------------- | |
| S16BD08730 | LLEQIAELLHILGSADRLMEVIREEMELIRDQFGDERRTEITANSADINIEDLISQEDVV |
| S18BD00684 | LLEQIAELLHILGSADRLMEVIREEMELIRDQFGDERRTEITANSADINIEDLISQEDVV |
| S18BD03994 | LLEQIAELLHILGSADRLMEVIREEMELIRDQFGDERRTEITANSADINIEDLISQEDVV |
| S18BD05011 | LLEQIAELLHILGSADRLMEVIREEMELIRDQFGDERRTEITANSADINIEDLISQEDVV |
| RKI_16-03723 | LLEQIAELLHILGSADRLMEVIREEMELIRDQFGDERRTEITANSADINIEDLISQEDVV |
| RKI_16-04315 | LLEQIAELLHILGSADRLMEVIREEMELIRDQFGDERRTEITANSADINIEDLISQEDVV |
| RKI_17-02304 | LLEQIAELLHILGSADRLMEVIREEMELIRDQFGDERRTEITANSADINIEDLISQEDVV |
| RKI_17-02411 | LLEQIAELLHILGSADRLMEVIREEMELIRDQFGDERRTEITANSADINIEDLISQEDVV |
| RKI_17-02757 | LLEQIAELLHILGSADRLMEVIREEMELIRDQFGDERRTEITANSADINIEDLISQEDVV |
| RKI_17-04797 | LLEQIAELLHILGSADRLMEVIREEMELIRDQFGDERRTEITANSADINIEDLISQEDVV |
| RKI_17-06869 | LLEQIAELLHILGSADRLMEVIREEMELIRDQFGDERRTEITANSADINIEDLISQEDVV |
| ERR2580277 | LLEQIAELLHILGSADRLMEVIREEMELIRDQFGDERRTEITANSADINIEDLISQEDVV |
| ERR2580276 | LLEQIAELLHILGSADRLMEVIREEMELIRDQFGDERRTEITANSADINIEDLISQEDVV |
| ERR2580273 | LLEQIAELLHILGSADRLMEVIREEMELIRDQFGDERRTEITANSADINIEDLISQEDVV |
| ERR2580274 | LLEQIAELLHILGSADRLMEVIREEMELIRDQFGDERRTEITANSADINIEDLISQEDVV |
| ERR2173656 | LLEQIAELLHILGSADRLMEVIREEMELIRDQFGDERRTEITANSADINIEDLISQEDVV |
| 17041676 | LLEQIAELLHILGSADRLMEVIREEMELIRDQFGDERRTEITANSADINIEDLISQEDVV |
| MT16-000061 | LLEQIAELLHILGSADRLMEVIREEMELIRDQFGDERRTEITANSADINIEDLISQEDVV |
| MT16-019416 | LLEQIAELLHILGSADRLMEVIREEMELIRDQFGDERRTEITANSADINIEDLISQEDVV |
| MT16-027865 | LLEQIAELLHILGSADRLMEVIREEMELIRDQFGDERRTEITANSADINIEDLISQEDVV |
| MT16-031693 | LLEQIAELLHILGSADRLMEVIREEMELIRDQFGDERRTEITANSADINIEDLISQEDVV |
| MT16-040253 | LLEQIAELLHILGSADRLMEVIREEMELIRDQFGDERRTEITANSADINIEDLISQEDVV |
| MT16-045379 | LLEQIAELLHILGSADRLMEVIREEMELIRDQFGDERRTEITANSADINIEDLISQEDVV |
| MT16-442728 | LLEQIAELLHILGSADRLMEVIREEMELIRDQFGDERRTEITANSADINIEDLISQEDVV |
| MT16-462857 | LLEQIAELLHILGSADRLMEVIREEMELIRDQFGDERRTEITANSADINIEDLISQEDVV |
| MT16-480196 | LLEQIAELLHILGSADRLMEVIREEMELIRDQFGDERRTEITANSADINIEDLISQEDVV |
| MT16-861555 | LLEQIAELLHILGSADRLMEVIREEMELIRDQFGDERRTEITANSADINIEDLISQEDVV |
| MT17-076833 | LLEQIAELLHILGSADRLMEVIREEMELIRDQFGDERRTEITANSADINIEDLISQEDVV |
| MT17-110677 | LLEQIAELLHILGSADRLMEVIREEMELIRDQFGDERRTEITANSADINIEDLISQEDVV |
| MT17-131730 | LLEQIAELLHILGSADRLMEVIREEMELIRDQFGDERRTEITANSADINIEDLISQEDVV |
| MT17-140890 | LLEQIAELLHILGSADRLMEVIREEMELIRDQFGDERRTEITANSADINIEDLISQEDVV |
| MT17-141840 | LLEQIAELLHILGSADRLMEVIREEMELIRDQFGDERRTEITANSADINIEDLISQEDVV |
| MT17-152488 | LLEQIAELLHILGSADRLMEVIREEMELIRDQFGDERRTEITANSADINIEDLISQEDVV |
| MT17-157311 | LLEQIAELLHILGSADRLMEVIREEMELIRDQFGDERRTEITANSADINIEDLISQEDVV |
| MT17-161645 | LLEQIAELLHILGSADRLMEVIREEMELIRDQFGDERRTEITANSADINIEDLISQEDVV |
| MT17-167951 | LLEQIAELLHILGSADRLMEVIREEMELIRDQFGDERRTEITANSADINIEDLISQEDVV |
| MT18-217732 | LLEQIAELLHILGSADRLMEVIREEMELIRDQFGDERRTEITANSADINIEDLISQEDVV |
| MT18-252580 | LLEQIAELLHILGSADRLMEVIREEMELIRDQFGDERRTEITANSADINIEDLISQEDVV |
| RIVM_H_2009-01 | LLEQIAELLHILGSADRLMEVIREEMELIRDQFGDERRTEITANSADINIEDLISQEDVV |
| RIVM_H_2010-01 | LLEQIAELLHILGSADRLMEVIREEMELIRDQFGDERRTEITANSADINIEDLISQEDVV |
| RIVM_H_2010-02 | LLEQIAELLHILGSADRLMEVIREEMELIRDQFGDERRTEITANSADINIEDLISQEDVV |
| RIVM_H_2011-01 | LLEQIAELLHILGSADRLMEVIREEMELIRDQFGDERRTEITANSADINIEDLISQEDVV |
| RIVM_H_2011-02 | LLEQIAELLHILGSADRLMEVIREEMELIRDQFGDERRTEITANSADINIEDLISQEDVV |
| RIVM_H_2011-03 | LLEQIAELLHILGSADRLMEVIREEMELIRDQFGDERRTEITANSADINIEDLISQEDVV |
| RIVM_H_2013-01 | LLEQIAELLHILGSADRLMEVIREEMELIRDQFGDERRTEITANSADINIEDLISQEDVV |
| RIVM_H_2013-02 | LLEQIAELLHILGSADRLMEVIREEMELIRDQFGDERRTEITANSADINIEDLISQEDVV |
| RIVM_H_2014-01 | LLEQIAELLHILGSADRLMEVIREEMELIRDQFGDERRTEITANSADINIEDLISQEDVV |
| RIVM_H_2014-02 | LLEQIAELLHILGSADRLMEVIREEMELIRDQFGDERRTEITANSADINIEDLISQEDVV |
| RIVM_H_2016-01 | LLEQIAELLHILGSADRLMEVIREEMELIRDQFGDERRTEITANSADINIEDLISQEDVV |
| RIVM_H_2016-02 | LLEQIAELLHILGSADRLMEVIREEMELIRDQFGDERRTEITANSADINIEDLISQEDVV |
| RIVM_H_2016-03 | LLEQIAELLHILGSADRLMEVIREEMELIRDQFGDERRTEITANSADINIEDLISQEDVV |
| RIVM_H_2016-04 | LLEQIAELLHILGSADRLMEVIREEMELIRDQFGDERRTEITANSADINIEDLISQEDVV |
| RIVM_H_2016-05 | LLEQIAELLHILGSADRLMEVIREEMELIRDQFGDERRTEITANSADINIEDLISQEDVV |
| RIVM_H_2016-06 | LLEQIAELLHILGSADRLMEVIREEMELIRDQFGDERRTEITANSADINIEDLISQEDVV |
| RIVM_H_2016-07 | LLEQIAELLHILGSADRLMEVIREEMELIRDQFGDERRTEITANSADINIEDLISQEDVV |
| RIVM_H_2016-08 | LLEQIAELLHILGSADRLMEVIREEMELIRDQFGDERRTEITANSADINIEDLISQEDVV |
| RIVM_H_2016-09 | LLEQIAELLHILGSADRLMEVIREEMELIRDQFGDERRTEITANSADINIEDLISQEDVV |
| RIVM_H_2016-10 | LLEQIAELLHILGSADRLMEVIREEMELIRDQFGDERRTEITANSADINIEDLISQEDVV |
| RIVM_H_2016-11 | LLEQIAELLHILGSADRLMEVIREEMELIRDQFGDERRTEITANSADINIEDLISQEDVV |
| RIVM_H_2016-12 | LLEQIAELLHILGSADRLMEVIREEMELIRDQFGDERRTEITANSADINIEDLISQEDVV |
| RIVM_H_2016-13 | LLEQIAELLHILGSADRLMEVIREEMELIRDQFGDERRTEITANSADINIEDLISQEDVV |
| RIVM_H_2016-14 | LLEQIAELLHILGSADRLMEVIREEMELIRDQFGDERRTEITANSADINIEDLISQEDVV |
| RIVM_H_2016-15 | LLEQIAELLHILGSADRLMEVIREEMELIRDQFGDERRTEITANSADINIEDLISQEDVV |
| RIVM_H_2017-01 | LLEQIAELLHILGSADRLMEVIREEMELIRDQFGDERRTEITANSADINIEDLISQEDVV |
| RIVM_H_2017-02 | LLEQIAELLHILGSADRLMEVIREEMELIRDQFGDERRTEITANSADINIEDLISQEDVV |
| RIVM_H_2017-03 | LLEQIAELLHILGSADRLMEVIREEMELIRDQFGDERRTEITANSADINIEDLISQEDVV |
| RIVM_H_2017-04 | LLEQIAELLHILGSADRLMEVIREEMELIRDQFGDERRTEITANSADINIEDLISQEDVV |
| RIVM_H_2017-05 | LLEQIAELLHILGSADRLMEVIREEMELIRDQFGDERRTEITANSADINIEDLISQEDVV |
| RIVM_H_2017-06 | LLEQIAELLHILGSADRLMEVIREEMELIRDQFGDERRTEITANSADINIEDLISQEDVV |
| RIVM_H_2017-07 | LLEQIAELLHILGSADRLMEVIREEMELIRDQFGDERRTEITANSADINIEDLISQEDVV |
| RIVM_H_2017-08 | LLEQIAELLHILGSADRLMEVIREEMELIRDQFGDERRTEITANSADINIEDLISQEDVV |
| RIVM_H_2017-09 | LLEQIAELLHILGSADRLMEVIREEMELIRDQFGDERRTEITANSADINIEDLISQEDVV |
| RIVM_H_2017-10 | LLEQIAELLHILGSADRLMEVIREEMELIRDQFGDERRTEITANSADINIEDLISQEDVV |
| RIVM_H_2017-11 | LLEQIAELLHILGSADRLMEVIREEMELIRDQFGDERRTEITANSADINIEDLISQEDVV |
| RIVM_H_2017-12 | LLEQIAELLHILGSADRLMEVIREEMELIRDQFGDERRTEITANSADINIEDLISQEDVV |
| RIVM_H_2017-13 | LLEQIAELLHILGSADRLMEVIREEMELIRDQFGDERRTEITANSADINIEDLISQEDVV |
| RIVM_H_2017-14 | LLEQIAELLHILGSADRLMEVIREEMELIRDQFGDERRTEITANSADINIEDLISQEDVV |
| RIVM_H_2017-15 | LLEQIAELLHILGSADRLMEVIREEMELIRDQFGDERRTEITANSADINIEDLISQEDVV |
| RIVM_H_2017-16 | LLEQIAELLHILGSADRLMEVIREEMELIRDQFGDERRTEITANSADINIEDLISQEDVV |
| RIVM_H_2017-17 | LLEQIAELLHILGSADRLMEVIREEMELIRDQFGDERRTEITANSADINIEDLISQEDVV |
| RIVM_H_2017-18 | LLEQIAELLHILGSADRLMEVIREEMELIRDQFGDERRTEITANSADINIEDLISQEDVV |
| RIVM_H_2017-19 | LLEQIAELLHILGSADRLMEVIREEMELIRDQFGDERRTEITANSADINIEDLISQEDVV |
| 15EP001483 | LLEQIAELLHILGSADRLMEVIREEMELIRDQFGDERRTEITANSADINIEDLISQEDVV |
| 17EP002363 | LLEQIAELLHILGSADRLMEVIREEMELIRDQFGDERRTEITANSADINIEDLISQEDVV |
| S_0812_17 | LLEQIAELLHILGSADRLMEVIREEMELIRDQFGDERRTEITANSADINIEDLISQEDVV |
| SRR1957844 | LLEQIAELLHILGSADRLMEVIREEMELIRDQFGDERRTEITANSADINIEDLISQEDVV |
| SRR1958654 | LLEQIAELLHILGSADRLMEVIREEMELIRDQFGDERRTEITANSADINIEDLISQEDVV |
| SRR1965077 | LLEQIAELLHILGSADRLMEVIREEMELIRDQFGDERRTEITANSADINIEDLISQEDVV |
| SRR1966369 | LLEQIAELLHILGSADRLMEVIREEMELIRDQFGDERRTEITANSADINIEDLISQEDVV |
| SRR1967117 | LLEQIAELLHILGSADRLMEVIREEMELIRDQFGDERRTEITANSADINIEDLISQEDVV |
| SRR1967922 | LLEQIAELLHILGSADRLMEVIREEMELIRDQFGDERRTEITANSADINIEDLISQEDVV |
| SRR8704720 | LLEQIAELLHILGSADRLMEVIREEMELIRDQFGDERRTEITANSADINIEDLISQEDVV |
| SRR7216071 | LLEQIAELLHILGSADRLMEVIREEMELIRDQFGDERRTEITANSADINIEDLISQEDVV |
| SRR7349175 | LLEQIAELLHILGSADRLMEVIREEMELIRDQFGDERRTEITANSADINIEDLISQEDVV |
| SRR7523148 | LLEQIAELLHILGSADRLMEVIREEMELIRDQFGDERRTEITANSADINIEDLISQEDVV |
| SRR7523854 | LLEQIAELLHILGSADRLMEVIREEMELIRDQFGDERRTEITANSADINIEDLISQEDVV |
| 313865 | LLEQIAELLHILGSADRLMEVIREEMELIRDQFGDERRTEITANSADINIEDLISQEDVV |
| SRR7277793 | LLEQIAELLHILGSADRLMEVIREEMELIRDQFGDERRTEITANSADINIEDLISQEDVV |
| SRR7343877 | LLEQIAELLHILGSADRLMEVIREEMELIRDQFGDERRTEITANSADINIEDLISQEDVV |
| SRR7351477 | LLEQIAELLHILGSADRLMEVIREEMELIRDQFGDERRTEITANSADINIEDLISQEDVV |
| SRR5583183 | LLEQIAELLHILGSADRLMEVIREEMELIRDQFGDERRTEITANSADINIEDLISQEDVV |
| SRR5585240 | LLEQIAELLHILGSADRLMEVIREEMELIRDQFGDERRTEITANSADINIEDLISQEDVV |
| SRR7284317 | LLEQIAELLHILGSADRLMEVIREEMELIRDQFGDERRTEITANSADINIEDLISQEDVV |
| SRR7299161 | LLEQIAELLHILGSADRLMEVIREEMELIRDQFGDERRTEITANSADINIEDLISQEDVV |
| SRR7401730 | LLEQIAELLHILGSADRLMEVIREEMELIRDQFGDERRTEITANSADINIEDLISQEDVV |
| SRR7469092 | LLEQIAELLHILGSADRLMEVIREEMELIRDQFGDERRTEITANSADINIEDLISQEDVV |
| SRR7879556 | LLEQIAELLHILGSADRLMEVIREEMELIRDQFGDERRTEITANSADINIEDLISQEDVV |
| SRR8526100 | LLEQIAELLHILGSADRLMEVIREEMELIRDQFGDERRTEITANSADINIEDLISQEDVV |
| SRR8553991 | LLEQIAELLHILGSADRLMEVIREEMELIRDQFGDERRTEITANSADINIEDLISQEDVV |
| SRR7842487 | LLEQIAELLHILGSADRLMEVIREEMELIRDQFGDERRTEITANSADINIEDLISQEDVV |
| SRR8054524 | LLEQIAELLHILGSADRLMEVIREEMELIRDQFGDERRTEITANSADINIEDLISQEDVV |
| SRR8054525 | LLEQIAELLHILGSADRLMEVIREEMELIRDQFGDERRTEITANSADINIEDLISQEDVV |
| SRR8524733 | LLEQIAELLHILGSADRLMEVIREEMELIRDQFGDERRTEITANSADINIEDLISQEDVV |
| SRR4093291 | LLEQIAELLHILGSADRLMEVIREEMELIRDQFGDERRTEITANSADINIEDLISQEDVV |
| SRR4245549 | LLEQIAELLHILGSADRLMEVIREEMELIRDQFGDERRTEITANSADINIEDLISQEDVV |
| SRR3057154 | LLEQIAELLHILGSADRLMEVIREEMELIRDQFGDERRTEITANSADINIEDLISQEDVV |
| SRR1726150 | LLEQIAELLHILGSADRLMEVIREEMELIRDQFGDERRTEITANSADINIEDLISQEDVV |
| SRR1996141 | LLEQIAELLHILGSADRLMEVIREEMELIRDQFGDERRTEITANSADINIEDLISQEDVV |
| SRR1107842 | LLEQIAELLHILGSADRLMEVIREEMELIRDQFGDERRTEITANSADINIEDLISQEDVV |
| SRR1157587 | LLEQIAELLHILGSADRLMEVIREEMELIRDQFGDERRTEITANSADINIEDLISQEDVV |
| SRR3027706 | LLEQIAELLHILGSADRLMEVIREEMELIRDQFGDERRTEITANSADINIEDLISQEDVV |
| SRR3027707 | LLEQIAELLHILGSADRLMEVIREEMELIRDQFGDERRTEITANSADINIEDLISQEDVV |
| SRR3027708 | LLEQIAELLHILGSADRLMEVIREEMELIRDQFGDERRTEITANSADINIEDLISQEDVV |
| SRR3027710 | LLEQIAELLHILGSADRLMEVIREEMELIRDQFGDERRTEITANSADINIEDLISQEDVV |
| SRR3027711 | LLEQIAELLHILGSADRLMEVIREEMELIRDQFGDERRTEITANSADINIEDLISQEDVV |
| SRR3027716 | LLEQIAELLHILGSADRLMEVIREEMELIRDQFGDERRTEITANSADINIEDLISQEDVV |
| SRR3027717 | LLEQIAELLHILGSADRLMEVIREEMELIRDQFGDERRTEITANSADINIEDLISQEDVV |
| SRR3027719 | LLEQIAELLHILGSADRLMEVIREEMELIRDQFGDERRTEITANSADINIEDLISQEDVV |
| SRR3027721 | LLEQIAELLHILGSADRLMEVIREEMELIRDQFGDERRTEITANSADINIEDLISQEDVV |
| SRR3027723 | LLEQIAELLHILGSADRLMEVIREEMELIRDQFGDERRTEITANSADINIEDLISQEDVV |
| SRR3115978 | LLEQIAELLHILGSADRLMEVIREEMELIRDQFGDERRTEITANSADINIEDLISQEDVV |
| SRR2534093 | LLEQIAELLHILGSADRLMEVIREEMELIRDQFGDERRTEITANSADINIEDLISQEDVV |
| SRR2534094 | LLEQIAELLHILGSADRLMEVIREEMELIRDQFGDERRTEITANSADINIEDLISQEDVV |
| SRR2534095 | LLEQIAELLHILGSADRLMEVIREEMELIRDQFGDERRTEITANSADINIEDLISQEDVV |
| SRR2534108 | LLEQIAELLHILGSADRLMEVIREEMELIRDQFGDERRTEITANSADINIEDLISQEDVV |
| SRR1106464 | LLEQIAELLHILGSADRLMEVIREEMELIRDQFGDERRTEITANSADINIEDLISQEDVV |
| SRR1106463 | LLEQIAELLHILGSADRLMEVIREEMELIRDQFGDERRTEITANSADINIEDLISQEDVV |
| SRR6949610 | LLEQIAELLHILGSADRLMEVIREEMELIRDQFGDERRTEITANSADINIEDLISQEDVV |
| SRR6950452 | LLEQIAELLHILGSADRLMEVIREEMELIRDQFGDERRTEITANSADINIEDLISQEDVV |
| ERR2019831 | LLEQIAELLHILGSADRLMEVIREEMELIRDQFGDERRTEITANSADINIEDLISQEDVV |
| SRR2085693 | LLEQIAELLHILGSADRLMEVIREEMELIRDQFGDERRTEITANSADINIEDLISQEDVV |
| SRR2086898 | LLEQIAELLHILGSADRLMEVIREEMELIRDQFGDERRTEITANSADINIEDLISQEDVV |
| SRR2175312 | LLEQIAELLHILGSADRLMEVIREEMELIRDQFGDERRTEITANSADINIEDLISQEDVV |
| SRR2175360 | LLEQIAELLHILGSADRLMEVIREEMELIRDQFGDERRTEITANSADINIEDLISQEDVV |
| SRR5231997 | LLEQIAELLHILGSADRLMEVIREEMELIRDQFGDERRTEITANSADINIEDLISQEDVV |
| SRR5232003 | LLEQIAELLHILGSADRLMEVIREEMELIRDQFGDERRTEITANSADINIEDLISQEDVV |
| SRR5232015 | LLEQIAELLHILGSADRLMEVIREEMELIRDQFGDERRTEITANSADINIEDLISQEDVV |
| SRR949434 | LLEQIAELLHILGSADRLMEVIREEMELIRDQFGDERRTEITANSADINIEDLISQEDVV |
| SRR3216575 | LLEQIAELLHILGSADRLMEVIREEMELIRDQFGDERRTEITANSADINIEDLISQEDVV |
| SRR5205342 | LLEQIAELLHILGSADRLMEVIREEMELIRDQFGDERRTEITANSADINIEDLISQEDVV |
| SRR1501669 | LLEQIAELLHILGSADRLMEVIREEMELIRDQFGDERRTEITANSADINIEDLISQEDVV |
| SRR5209740 | LLEQIAELLHILGSADRLMEVIREEMELIRDQFGDERRTEITANSADINIEDLISQEDVV |
| SRR3240355 | LLEQIAELLHILGSADRLMEVIREEMELIRDQFGDERRTEITANSADINIEDLISQEDVV |
| SRR3392777 | LLEQIAELLHILGSADRLMEVIREEMELIRDQFGDERRTEITANSADINIEDLISQEDVV |
| SRR3593671 | LLEQIAELLHILGSADRLMEVIREEMELIRDQFGDERRTEITANSADINIEDLISQEDVV |
| SRR5413290 | LLEQIAELLHILGSADRLMEVIREEMELIRDQFGDERRTEITANSADINIEDLISQEDVV |
| SRR5590269 | LLEQIAELLHILGSADRLMEVIREEMELIRDQFGDERRTEITANSADINIEDLISQEDVV |
| SRR5812103 | LLEQIAELLHILGSADRLMEVIREEMELIRDQFGDERRTEITANSADINIEDLISQEDVV |
| SRR2830941 | LLEQIAELLHILGSADRLMEVIREEMELIRDQFGDERRTEITANSADINIEDLISQEDVV |
| SRR2830966 | LLEQIAELLHILGSADRLMEVIREEMELIRDQFGDERRTEITANSADINIEDLISQEDVV |
| SRR3137270 | LLEQIAELLHILGSADRLMEVIREEMELIRDQFGDERRTEITANSADINIEDLISQEDVV |
| SRR3137271 | LLEQIAELLHILGSADRLMEVIREEMELIRDQFGDERRTEITANSADINIEDLISQEDVV |
| ERR526807 | LLEQIAELLHILGSADRLMEVIREEMELIRDQFGDERRTEITANSADINIEDLISQEDVV |
| ERR2197922 | LLEQIAELLHILGSADRLMEVIREEMELIRDQFGDERRTEITANSADINIEDLISQEDVV |
| ERR2197923 | LLEQIAELLHILGSADRLMEVIREEMELIRDQFGDERRTEITANSADINIEDLISQEDVV |
| ERR2197924 | LLEQIAELLHILGSADRLMEVIREEMELIRDQFGDERRTEITANSADINIEDLISQEDVV |
| ERR2197925 | LLEQIAELLHILGSADRLMEVIREEMELIRDQFGDERRTEITANSADINIEDLISQEDVV |
| ERR2197927 | LLEQIAELLHILGSADRLMEVIREEMELIRDQFGDERRTEITANSADINIEDLISQEDVV |
| ERR2197929 | LLEQIAELLHILGSADRLMEVIREEMELIRDQFGDERRTEITANSADINIEDLISQEDVV |
| SRR1648149 | LLEQIAELLHILGSADRLMEVIREEMELIRDQFGDERRTEITANSADINIEDLISQEDVV |
| SRR1048299 | LLEQIAELLHILGSADRLMEVIREEMELIRDQFGDERRTEITANSADINIEDLISQEDVV |
| SRR1300677 | LLEQIAELLHILGSADRLMEVIREEMELIRDQFGDERRTEITANSADINIEDLISQEDVV |
| SRR1288356 | LLEQIAELLHILGSADRLMEVIREEMELIRDQFGDERRTEITANSADINIEDLISQEDVV |
| SRR7426190 | LLEQIAELLHILGSADRLMEVIREEMELIRDQFGDERRTEITANSADINIEDLISQEDVV |
| SRR7426192 | LLEQIAELLHILGSADRLMEVIREEMELIRDQFGDERRTEITANSADINIEDLISQEDVV |
| SRR7426193 | LLEQIAELLHILGSADRLMEVIREEMELIRDQFGDERRTEITANSADINIEDLISQEDVV |
| SRR7441832 | LLEQIAELLHILGSADRLMEVIREEMELIRDQFGDERRTEITANSADINIEDLISQEDVV |
| SRR7426179 | LLEQIAELLHILGSADRLMEVIREEMELIRDQFGDERRTEITANSADINIEDLISQEDVV |
| SRR7439238 | LLEQIAELLHILGSADRLMEVIREEMELIRDQFGDERRTEITANSADINIEDLISQEDVV |
| SRR7439244 | LLEQIAELLHILGSADRLMEVIREEMELIRDQFGDERRTEITANSADINIEDLISQEDVV |
| SRR7439259 | LLEQIAELLHILGSADRLMEVIREEMELIRDQFGDERRTEITANSADINIEDLISQEDVV |
| SRR7439260 | LLEQIAELLHILGSADRLMEVIREEMELIRDQFGDERRTEITANSADINIEDLISQEDVV |
| SRR7441786 | LLEQIAELLHILGSADRLMEVIREEMELIRDQFGDERRTEITANSADINIEDLISQEDVV |
| SRR7441797 | LLEQIAELLHILGSADRLMEVIREEMELIRDQFGDERRTEITANSADINIEDLISQEDVV |
| ERR1759093 | LLEQIAELLHILGSADRLMEVIREEMELIRDQFGDERRTEITANSADINIEDLISQEDVV |
| ERR2580275 | LLEQIAELLHILGSADRLMEVIREEMELIRDQFGDERRTEITANSADINIEDLISQEDVV |
| ERR1759204 | LLEQIAELLHILGSADRLMEVIREEMELIRDQFGDERRTEITANSADINIEDLISQEDVV |
| SRR1300699 | LLEQIAELLHILGSADRLMEVIREEMELIRDQFGDERRTEITANSADINIEDLISQEDVV |
| S_0825_17 | LLEQIAELLHILGSADRLMEVIREEMELIRDQFGDERRTEITANSADINIEDLISQEDVV |
| SRR1958215 | LLEQIAELLHILGSADRLMEVIREEMELIRDQFGDERRTEITANSADINIEDLISQEDVV |
| SRR1958540 | LLEQIAELLHILGSADRLMEVIREEMELIRDQFGDERRTEITANSADINIEDLISQEDVV |
| SRR1958636 | LLEQIAELLHILGSADRLMEVIREEMELIRDQFGDERRTEITANSADINIEDLISQEDVV |
| SRR1959422 | LLEQIAELLHILGSADRLMEVIREEMELIRDQFGDERRTEITANSADINIEDLISQEDVV |
| SRR1959427 | LLEQIAELLHILGSADRLMEVIREEMELIRDQFGDERRTEITANSADINIEDLISQEDVV |
| SRR1960226 | LLEQIAELLHILGSADRLMEVIREEMELIRDQFGDERRTEITANSADINIEDLISQEDVV |
| SRR1963498 | LLEQIAELLHILGSADRLMEVIREEMELIRDQFGDERRTEITANSADINIEDLISQEDVV |
| SRR1965947 | LLEQIAELLHILGSADRLMEVIREEMELIRDQFGDERRTEITANSADINIEDLISQEDVV |
| SRR1966125 | LLEQIAELLHILGSADRLMEVIREEMELIRDQFGDERRTEITANSADINIEDLISQEDVV |
| SRR1966330 | LLEQIAELLHILGSADRLMEVIREEMELIRDQFGDERRTEITANSADINIEDLISQEDVV |
| SRR1966565 | LLEQIAELLHILGSADRLMEVIREEMELIRDQFGDERRTEITANSADINIEDLISQEDVV |
| SRR1966864 | LLEQIAELLHILGSADRLMEVIREEMELIRDQFGDERRTEITANSADINIEDLISQEDVV |
| SRR1966989 | LLEQIAELLHILGSADRLMEVIREEMELIRDQFGDERRTEITANSADINIEDLISQEDVV |
| SRR1967688 | LLEQIAELLHILGSADRLMEVIREEMELIRDQFGDERRTEITANSADINIEDLISQEDVV |
| SRR1967733 | LLEQIAELLHILGSADRLMEVIREEMELIRDQFGDERRTEITANSADINIEDLISQEDVV |
| SRR1967746 | LLEQIAELLHILGSADRLMEVIREEMELIRDQFGDERRTEITANSADINIEDLISQEDVV |
| SRR1968341 | LLEQIAELLHILGSADRLMEVIREEMELIRDQFGDERRTEITANSADINIEDLISQEDVV |
| SRR1968456 | LLEQIAELLHILGSADRLMEVIREEMELIRDQFGDERRTEITANSADINIEDLISQEDVV |
| SRR1968465 | LLEQIAELLHILGSADRLMEVIREEMELIRDQFGDERRTEITANSADINIEDLISQEDVV |
| SRR1968761 | LLEQIAELLHILGSADRLMEVIREEMELIRDQFGDERRTEITANSADINIEDLISQEDVV |
| SRR1969047 | LLEQIAELLHILGSADRLMEVIREEMELIRDQFGDERRTEITANSADINIEDLISQEDVV |
| SRR1969255 | LLEQIAELLHILGSADRLMEVIREEMELIRDQFGDERRTEITANSADINIEDLISQEDVV |
| SRR1969412 | LLEQIAELLHILGSADRLMEVIREEMELIRDQFGDERRTEITANSADINIEDLISQEDVV |
| SRR1969524 | LLEQIAELLHILGSADRLMEVIREEMELIRDQFGDERRTEITANSADINIEDLISQEDVV |
| SRR1969584 | LLEQIAELLHILGSADRLMEVIREEMELIRDQFGDERRTEITANSADINIEDLISQEDVV |
| SRR1969648 | LLEQIAELLHILGSADRLMEVIREEMELIRDQFGDERRTEITANSADINIEDLISQEDVV |
| SRR1969804 | LLEQIAELLHILGSADRLMEVIREEMELIRDQFGDERRTEITANSADINIEDLISQEDVV |
| SRR1970221 | LLEQIAELLHILGSADRLMEVIREEMELIRDQFGDERRTEITANSADINIEDLISQEDVV |
| SRR1970268 | LLEQIAELLHILGSADRLMEVIREEMELIRDQFGDERRTEITANSADINIEDLISQEDVV |
| SRR1965862 | LLEQIAELLHILGSADRLMEVIREEMELIRDQFGDERRTEITANSADINIEDLISQEDVV |
| SRR1967363 | LLEQIAELLHILGSADRLMEVIREEMELIRDQFGDERRTEITANSADINIEDLISQEDVV |
| SRR1968276 | LLEQIAELLHILGSADRLMEVIREEMELIRDQFGDERRTEITANSADINIEDLISQEDVV |
| SRR1968967 | LLEQIAELLHILGSADRLMEVIREEMELIRDQFGDERRTEITANSADINIEDLISQEDVV |
| SRR3321531 | LLEQIAELLHILGSADRLMEVIREEMELIRDQFGDERRTEITANSADINIEDLISQEDVV |
| SRR3321883 | LLEQIAELLHILGSADRLMEVIREEMELIRDQFGDERRTEITANSADINIEDLISQEDVV |
| SRR3322413 | LLEQIAELLHILGSADRLMEVIREEMELIRDQFGDERRTEITANSADINIEDLISQEDVV |
| SRR3323012 | LLEQIAELLHILGSADRLMEVIREEMELIRDQFGDERRTEITANSADINIEDLISQEDVV |
| SRR5194289 | LLEQIAELLHILGSADRLMEVIREEMELIRDQFGDERRTEITANSADINIEDLISQEDVV |
| SRR7163798 | LLEQIAELLHILGSADRLMEVIREEMELIRDQFGDERRTEITANSADINIEDLISQEDVV |
| SRR7172610 | LLEQIAELLHILGSADRLMEVIREEMELIRDQFGDERRTEITANSADINIEDLISQEDVV |
| SRR7204568 | LLEQIAELLHILGSADRLMEVIREEMELIRDQFGDERRTEITANSADINIEDLISQEDVV |
| SRR7223230 | LLEQIAELLHILGSADRLMEVIREEMELIRDQFGDERRTEITANSADINIEDLISQEDVV |
| SRR7230675 | LLEQIAELLHILGSADRLMEVIREEMELIRDQFGDERRTEITANSADINIEDLISQEDVV |
| SRR7278056 | LLEQIAELLHILGSADRLMEVIREEMELIRDQFGDERRTEITANSADINIEDLISQEDVV |
| SRR7278086 | LLEQIAELLHILGSADRLMEVIREEMELIRDQFGDERRTEITANSADINIEDLISQEDVV |
| SRR7285841 | LLEQIAELLHILGSADRLMEVIREEMELIRDQFGDERRTEITANSADINIEDLISQEDVV |
| SRR7292625 | LLEQIAELLHILGSADRLMEVIREEMELIRDQFGDERRTEITANSADINIEDLISQEDVV |
| SRR7292665 | LLEQIAELLHILGSADRLMEVIREEMELIRDQFGDERRTEITANSADINIEDLISQEDVV |
| SRR7297965 | LLEQIAELLHILGSADRLMEVIREEMELIRDQFGDERRTEITANSADINIEDLISQEDVV |
| SRR7350726 | LLEQIAELLHILGSADRLMEVIREEMELIRDQFGDERRTEITANSADINIEDLISQEDVV |
| SRR7410328 | LLEQIAELLHILGSADRLMEVIREEMELIRDQFGDERRTEITANSADINIEDLISQEDVV |
| SRR7474665 | LLEQIAELLHILGSADRLMEVIREEMELIRDQFGDERRTEITANSADINIEDLISQEDVV |
| SRR7523184 | LLEQIAELLHILGSADRLMEVIREEMELIRDQFGDERRTEITANSADINIEDLISQEDVV |
| SRR7187264 | LLEQIAELLHILGSADRLMEVIREEMELIRDQFGDERRTEITANSADINIEDLISQEDVV |
| SRR7204445 | LLEQIAELLHILGSADRLMEVIREEMELIRDQFGDERRTEITANSADINIEDLISQEDVV |
| SRR7285641 | LLEQIAELLHILGSADRLMEVIREEMELIRDQFGDERRTEITANSADINIEDLISQEDVV |
| SRR7286695 | LLEQIAELLHILGSADRLMEVIREEMELIRDQFGDERRTEITANSADINIEDLISQEDVV |
| SRR7286705 | LLEQIAELLHILGSADRLMEVIREEMELIRDQFGDERRTEITANSADINIEDLISQEDVV |
| SRR7292931 | LLEQIAELLHILGSADRLMEVIREEMELIRDQFGDERRTEITANSADINIEDLISQEDVV |
| SRR7310349 | LLEQIAELLHILGSADRLMEVIREEMELIRDQFGDERRTEITANSADINIEDLISQEDVV |
| SRR7351616 | LLEQIAELLHILGSADRLMEVIREEMELIRDQFGDERRTEITANSADINIEDLISQEDVV |
| SRR7414818 | LLEQIAELLHILGSADRLMEVIREEMELIRDQFGDERRTEITANSADINIEDLISQEDVV |
| SRR7426480 | LLEQIAELLHILGSADRLMEVIREEMELIRDQFGDERRTEITANSADINIEDLISQEDVV |
| SRR5584105 | LLEQIAELLHILGSADRLMEVIREEMELIRDQFGDERRTEITANSADINIEDLISQEDVV |
| SRR5584565 | LLEQIAELLHILGSADRLMEVIREEMELIRDQFGDERRTEITANSADINIEDLISQEDVV |
| SRR5584614 | LLEQIAELLHILGSADRLMEVIREEMELIRDQFGDERRTEITANSADINIEDLISQEDVV |
| SRR5631543 | LLEQIAELLHILGSADRLMEVIREEMELIRDQFGDERRTEITANSADINIEDLISQEDVV |
| SRR5631553 | LLEQIAELLHILGSADRLMEVIREEMELIRDQFGDERRTEITANSADINIEDLISQEDVV |
| SRR7123196 | LLEQIAELLHILGSADRLMEVIREEMELIRDQFGDERRTEITANSADINIEDLISQEDVV |
| SRR7163819 | LLEQIAELLHILGSADRLMEVIREEMELIRDQFGDERRTEITANSADINIEDLISQEDVV |
| SRR7163920 | LLEQIAELLHILGSADRLMEVIREEMELIRDQFGDERRTEITANSADINIEDLISQEDVV |
| SRR7209528 | LLEQIAELLHILGSADRLMEVIREEMELIRDQFGDERRTEITANSADINIEDLISQEDVV |
| SRR7249868 | LLEQIAELLHILGSADRLMEVIREEMELIRDQFGDERRTEITANSADINIEDLISQEDVV |
| SRR7278088 | LLEQIAELLHILGSADRLMEVIREEMELIRDQFGDERRTEITANSADINIEDLISQEDVV |
| SRR7285788 | LLEQIAELLHILGSADRLMEVIREEMELIRDQFGDERRTEITANSADINIEDLISQEDVV |
| SRR7286789 | LLEQIAELLHILGSADRLMEVIREEMELIRDQFGDERRTEITANSADINIEDLISQEDVV |
| SRR7286886 | LLEQIAELLHILGSADRLMEVIREEMELIRDQFGDERRTEITANSADINIEDLISQEDVV |
| SRR7310632 | LLEQIAELLHILGSADRLMEVIREEMELIRDQFGDERRTEITANSADINIEDLISQEDVV |
| SRR7350631 | LLEQIAELLHILGSADRLMEVIREEMELIRDQFGDERRTEITANSADINIEDLISQEDVV |
| SRR7458741 | LLEQIAELLHILGSADRLMEVIREEMELIRDQFGDERRTEITANSADINIEDLISQEDVV |
| SRR7480280 | LLEQIAELLHILGSADRLMEVIREEMELIRDQFGDERRTEITANSADINIEDLISQEDVV |
| SRR7523660 | LLEQIAELLHILGSADRLMEVIREEMELIRDQFGDERRTEITANSADINIEDLISQEDVV |
| SRR7523775 | LLEQIAELLHILGSADRLMEVIREEMELIRDQFGDERRTEITANSADINIEDLISQEDVV |
| SRR7251101 | LLEQIAELLHILGSADRLMEVIREEMELIRDQFGDERRTEITANSADINIEDLISQEDVV |
| SRR7284299 | LLEQIAELLHILGSADRLMEVIREEMELIRDQFGDERRTEITANSADINIEDLISQEDVV |
| SRR7285738 | LLEQIAELLHILGSADRLMEVIREEMELIRDQFGDERRTEITANSADINIEDLISQEDVV |
| SRR7310640 | LLEQIAELLHILGSADRLMEVIREEMELIRDQFGDERRTEITANSADINIEDLISQEDVV |
| SRR7349159 | LLEQIAELLHILGSADRLMEVIREEMELIRDQFGDERRTEITANSADINIEDLISQEDVV |
| SRR7474873 | LLEQIAELLHILGSADRLMEVIREEMELIRDQFGDERRTEITANSADINIEDLISQEDVV |
| SRR7495689 | LLEQIAELLHILGSADRLMEVIREEMELIRDQFGDERRTEITANSADINIEDLISQEDVV |
| SRR7495752 | LLEQIAELLHILGSADRLMEVIREEMELIRDQFGDERRTEITANSADINIEDLISQEDVV |
| ----------------------------------------------------------------------------- | |
| S16BD08730 | VTLSHQGYVKYQPLTDYEAQRRGGKGKSAARIKEEDFIDRLLVANTHDTILCFSSRGRLY |
| S18BD00684 | VTLSHQGYVKYQPLTDYEAQRRGGKGKSAARIKEEDFIDRLLVANTHDTILCFSSRGRLY |
| S18BD03994 | VTLSHQGYVKYQPLTDYEAQRRGGKGKSAARIKEEDFIDRLLVANTHDTILCFSSRGRLY |
| S18BD05011 | VTLSHQGYVKYQPLTDYEAQRRGGKGKSAARIKEEDFIDRLLVANTHDTILCFSSRGRLY |
| RKI_16-03723 | VTLSHQGYVKYQPLTDYEAQRRGGKGKSAARIKEEDFIDRLLVANTHDTILCFSSRGRLY |
| RKI_16-04315 | VTLSHQGYVKYQPLTDYEAQRRGGKGKSAARIKEEDFIDRLLVANTHDTILCFSSRGRLY |
| RKI_17-02304 | VTLSHQGYVKYQPLTDYEAQRRGGKGKSAARIKEEDFIDRLLVANTHDTILCFSSRGRLY |
| RKI_17-02411 | VTLSHQGYVKYQPLTDYEAQRRGGKGKSAARIKEEDFIDRLLVANTHDTILCFSSRGRLY |
| RKI_17-02757 | VTLSHQGYVKYQPLTDYEAQRRGGKGKSAARIKEEDFIDRLLVANTHDTILCFSSRGRLY |
| RKI_17-04797 | VTLSHQGYVKYQPLTDYEAQRRGGKGKSAARIKEEDFIDRLLVANTHDTILCFSSRGRLY |
| RKI_17-06869 | VTLSHQGYVKYQPLTDYEAQRRGGKGKSAARIKEEDFIDRLLVANTHDTILCFSSRGRLY |
| ERR2580277 | VTLSHQGYVKYQPLTDYEAQRRGGKGKSAARIKEEDFIDRLLVANTHDTILCFSSRGRLY |
| ERR2580276 | VTLSHQGYVKYQPLTDYEAQRRGGKGKSAARIKEEDFIDRLLVANTHDTILCFSSRGRLY |
| ERR2580273 | VTLSHQGYVKYQPLTDYEAQRRGGKGKSAARIKEEDFIDRLLVANTHDTILCFSSRGRLY |
| ERR2580274 | VTLSHQGYVKYQPLTDYEAQRRGGKGKSAARIKEEDFIDRLLVANTHDTILCFSSRGRLY |
| ERR2173656 | VTLSHQGYVKYQPLTDYEAQRRGGKGKSAARIKEEDFIDRLLVANTHDTILCFSSRGRLY |
| 17041676 | VTLSHQGYVKYQPLTDYEAQRRGGKGKSAARIKEEDFIDRLLVANTHDTILCFSSRGRLY |
| MT16-000061 | VTLSHQGYVKYQPLTDYEAQRRGGKGKSAARIKEEDFIDRLLVANTHDTILCFSSRGRLY |
| MT16-019416 | VTLSHQGYVKYQPLTDYEAQRRGGKGKSAARIKEEDFIDRLLVANTHDTILCFSSRGRLY |
| MT16-027865 | VTLSHQGYVKYQPLTDYEAQRRGGKGKSAARIKEEDFIDRLLVANTHDTILCFSSRGRLY |
| MT16-031693 | VTLSHQGYVKYQPLTDYEAQRRGGKGKSAARIKEEDFIDRLLVANTHDTILCFSSRGRLY |
| MT16-040253 | VTLSHQGYVKYQPLTDYEAQRRGGKGKSAARIKEEDFIDRLLVANTHDTILCFSSRGRLY |
| MT16-045379 | VTLSHQGYVKYQPLTDYEAQRRGGKGKSAARIKEEDFIDRLLVANTHDTILCFSSRGRLY |
| MT16-442728 | VTLSHQGYVKYQPLTDYEAQRRGGKGKSAARIKEEDFIDRLLVANTHDTILCFSSRGRLY |
| MT16-462857 | VTLSHQGYVKYQPLTDYEAQRRGGKGKSAARIKEEDFIDRLLVANTHDTILCFSSRGRLY |
| MT16-480196 | VTLSHQGYVKYQPLTDYEAQRRGGKGKSAARIKEEDFIDRLLVANTHDTILCFSSRGRLY |
| MT16-861555 | VTLSHQGYVKYQPLTDYEAQRRGGKGKSAARIKEEDFIDRLLVANTHDTILCFSSRGRLY |
| MT17-076833 | VTLSHQGYVKYQPLTDYEAQRRGGKGKSAARIKEEDFIDRLLVANTHDTILCFSSRGRLY |
| MT17-110677 | VTLSHQGYVKYQPLTDYEAQRRGGKGKSAARIKEEDFIDRLLVANTHDTILCFSSRGRLY |
| MT17-131730 | VTLSHQGYVKYQPLTDYEAQRRGGKGKSAARIKEEDFIDRLLVANTHDTILCFSSRGRLY |
| MT17-140890 | VTLSHQGYVKYQPLTDYEAQRRGGKGKSAARIKEEDFIDRLLVANTHDTILCFSSRGRLY |
| MT17-141840 | VTLSHQGYVKYQPLTDYEAQRRGGKGKSAARIKEEDFIDRLLVANTHDTILCFSSRGRLY |
| MT17-152488 | VTLSHQGYVKYQPLTDYEAQRRGGKGKSAARIKEEDFIDRLLVANTHDTILCFSSRGRLY |
| MT17-157311 | VTLSHQGYVKYQPLTDYEAQRRGGKGKSAARIKEEDFIDRLLVANTHDTILCFSSRGRLY |
| MT17-161645 | VTLSHQGYVKYQPLTDYEAQRRGGKGKSAARIKEEDFIDRLLVANTHDTILCFSSRGRLY |
| MT17-167951 | VTLSHQGYVKYQPLTDYEAQRRGGKGKSAARIKEEDFIDRLLVANTHDTILCFSSRGRLY |
| MT18-217732 | VTLSHQGYVKYQPLTDYEAQRRGGKGKSAARIKEEDFIDRLLVANTHDTILCFSSRGRLY |
| MT18-252580 | VTLSHQGYVKYQPLTDYEAQRRGGKGKSAARIKEEDFIDRLLVANTHDTILCFSSRGRLY |
| RIVM_H_2009-01 | VTLSHQGYVKYQPLTDYEAQRRGGKGKSAARIKEEDFIDRLLVANTHDTILCFSSRGRLY |
| RIVM_H_2010-01 | VTLSHQGYVKYQPLTDYEAQRRGGKGKSAARIKEEDFIDRLLVANTHDTILCFSSRGRLY |
| RIVM_H_2010-02 | VTLSHQGYVKYQPLTDYEAQRRGGKGKSAARIKEEDFIDRLLVANTHDTILCFSSRGRLY |
| RIVM_H_2011-01 | VTLSHQGYVKYQPLTDYEAQRRGGKGKSAARIKEEDFIDRLLVANTHDTILCFSSRGRLY |
| RIVM_H_2011-02 | VTLSHQGYVKYQPLTDYEAQRRGGKGKSAARIKEEDFIDRLLVANTHDTILCFSSRGRLY |
| RIVM_H_2011-03 | VTLSHQGYVKYQPLTDYEAQRRGGKGKSAARIKEEDFIDRLLVANTHDTILCFSSRGRLY |
| RIVM_H_2013-01 | VTLSHQGYVKYQPLTDYEAQRRGGKGKSAARIKEEDFIDRLLVANTHDTILCFSSRGRLY |
| RIVM_H_2013-02 | VTLSHQGYVKYQPLTDYEAQRRGGKGKSAARIKEEDFIDRLLVANTHDTILCFSSRGRLY |
| RIVM_H_2014-01 | VTLSHQGYVKYQPLTDYEAQRRGGKGKSAARIKEEDFIDRLLVANTHDTILCFSSRGRLY |
| RIVM_H_2014-02 | VTLSHQGYVKYQPLTDYEAQRRGGKGKSAARIKEEDFIDRLLVANTHDTILCFSSRGRLY |
| RIVM_H_2016-01 | VTLSHQGYVKYQPLTDYEAQRRGGKGKSAARIKEEDFIDRLLVANTHDTILCFSSRGRLY |
| RIVM_H_2016-02 | VTLSHQGYVKYQPLTDYEAQRRGGKGKSAARIKEEDFIDRLLVANTHDTILCFSSRGRLY |
| RIVM_H_2016-03 | VTLSHQGYVKYQPLTDYEAQRRGGKGKSAARIKEEDFIDRLLVANTHDTILCFSSRGRLY |
| RIVM_H_2016-04 | VTLSHQGYVKYQPLTDYEAQRRGGKGKSAARIKEEDFIDRLLVANTHDTILCFSSRGRLY |
| RIVM_H_2016-05 | VTLSHQGYVKYQPLTDYEAQRRGGKGKSAARIKEEDFIDRLLVANTHDTILCFSSRGRLY |
| RIVM_H_2016-06 | VTLSHQGYVKYQPLTDYEAQRRGGKGKSAARIKEEDFIDRLLVANTHDTILCFSSRGRLY |
| RIVM_H_2016-07 | VTLSHQGYVKYQPLTDYEAQRRGGKGKSAARIKEEDFIDRLLVANTHDTILCFSSRGRLY |
| RIVM_H_2016-08 | VTLSHQGYVKYQPLTDYEAQRRGGKGKSAARIKEEDFIDRLLVANTHDTILCFSSRGRLY |
| RIVM_H_2016-09 | VTLSHQGYVKYQPLTDYEAQRRGGKGKSAARIKEEDFIDRLLVANTHDTILCFSSRGRLY |
| RIVM_H_2016-10 | VTLSHQGYVKYQPLTDYEAQRRGGKGKSAARIKEEDFIDRLLVANTHDTILCFSSRGRLY |
| RIVM_H_2016-11 | VTLSHQGYVKYQPLTDYEAQRRGGKGKSAARIKEEDFIDRLLVANTHDTILCFSSRGRLY |
| RIVM_H_2016-12 | VTLSHQGYVKYQPLTDYEAQRRGGKGKSAARIKEEDFIDRLLVANTHDTILCFSSRGRLY |
| RIVM_H_2016-13 | VTLSHQGYVKYQPLTDYEAQRRGGKGKSAARIKEEDFIDRLLVANTHDTILCFSSRGRLY |
| RIVM_H_2016-14 | VTLSHQGYVKYQPLTDYEAQRRGGKGKSAARIKEEDFIDRLLVANTHDTILCFSSRGRLY |
| RIVM_H_2016-15 | VTLSHQGYVKYQPLTDYEAQRRGGKGKSAARIKEEDFIDRLLVANTHDTILCFSSRGRLY |
| RIVM_H_2017-01 | VTLSHQGYVKYQPLTDYEAQRRGGKGKSAARIKEEDFIDRLLVANTHDTILCFSSRGRLY |
| RIVM_H_2017-02 | VTLSHQGYVKYQPLTDYEAQRRGGKGKSAARIKEEDFIDRLLVANTHDTILCFSSRGRLY |
| RIVM_H_2017-03 | VTLSHQGYVKYQPLTDYEAQRRGGKGKSAARIKEEDFIDRLLVANTHDTILCFSSRGRLY |
| RIVM_H_2017-04 | VTLSHQGYVKYQPLTDYEAQRRGGKGKSAARIKEEDFIDRLLVANTHDTILCFSSRGRLY |
| RIVM_H_2017-05 | VTLSHQGYVKYQPLTDYEAQRRGGKGKSAARIKEEDFIDRLLVANTHDTILCFSSRGRLY |
| RIVM_H_2017-06 | VTLSHQGYVKYQPLTDYEAQRRGGKGKSAARIKEEDFIDRLLVANTHDTILCFSSRGRLY |
| RIVM_H_2017-07 | VTLSHQGYVKYQPLTDYEAQRRGGKGKSAARIKEEDFIDRLLVANTHDTILCFSSRGRLY |
| RIVM_H_2017-08 | VTLSHQGYVKYQPLTDYEAQRRGGKGKSAARIKEEDFIDRLLVANTHDTILCFSSRGRLY |
| RIVM_H_2017-09 | VTLSHQGYVKYQPLTDYEAQRRGGKGKSAARIKEEDFIDRLLVANTHDTILCFSSRGRLY |
| RIVM_H_2017-10 | VTLSHQGYVKYQPLTDYEAQRRGGKGKSAARIKEEDFIDRLLVANTHDTILCFSSRGRLY |
| RIVM_H_2017-11 | VTLSHQGYVKYQPLTDYEAQRRGGKGKSAARIKEEDFIDRLLVANTHDTILCFSSRGRLY |
| RIVM_H_2017-12 | VTLSHQGYVKYQPLTDYEAQRRGGKGKSAARIKEEDFIDRLLVANTHDTILCFSSRGRLY |
| RIVM_H_2017-13 | VTLSHQGYVKYQPLTDYEAQRRGGKGKSAARIKEEDFIDRLLVANTHDTILCFSSRGRLY |
| RIVM_H_2017-14 | VTLSHQGYVKYQPLTDYEAQRRGGKGKSAARIKEEDFIDRLLVANTHDTILCFSSRGRLY |
| RIVM_H_2017-15 | VTLSHQGYVKYQPLTDYEAQRRGGKGKSAARIKEEDFIDRLLVANTHDTILCFSSRGRLY |
| RIVM_H_2017-16 | VTLSHQGYVKYQPLTDYEAQRRGGKGKSAARIKEEDFIDRLLVANTHDTILCFSSRGRLY |
| RIVM_H_2017-17 | VTLSHQGYVKYQPLTDYEAQRRGGKGKSAARIKEEDFIDRLLVANTHDTILCFSSRGRLY |
| RIVM_H_2017-18 | VTLSHQGYVKYQPLTDYEAQRRGGKGKSAARIKEEDFIDRLLVANTHDTILCFSSRGRLY |
| RIVM_H_2017-19 | VTLSHQGYVKYQPLTDYEAQRRGGKGKSAARIKEEDFIDRLLVANTHDTILCFSSRGRLY |
| 15EP001483 | VTLSHQGYVKYQPLTDYEAQRRGGKGKSAARIKEEDFIDRLLVANTHDTILCFSSRGRLY |
| 17EP002363 | VTLSHQGYVKYQPLTDYEAQRRGGKGKSAARIKEEDFIDRLLVANTHDTILCFSSRGRLY |
| S_0812_17 | VTLSHQGYVKYQPLTDYEAQRRGGKGKSAARIKEEDFIDRLLVANTHDTILCFSSRGRLY |
| SRR1957844 | VTLSHQGYVKYQPLTDYEAQRRGGKGKSAARIKEEDFIDRLLVANTHDTILCFSSRGRLY |
| SRR1958654 | VTLSHQGYVKYQPLTDYEAQRRGGKGKSAARIKEEDFIDRLLVANTHDTILCFSSRGRLY |
| SRR1965077 | VTLSHQGYVKYQPLTDYEAQRRGGKGKSAARIKEEDFIDRLLVANTHDTILCFSSRGRLY |
| SRR1966369 | VTLSHQGYVKYQPLTDYEAQRRGGKGKSAARIKEEDFIDRLLVANTHDTILCFSSRGRLY |
| SRR1967117 | VTLSHQGYVKYQPLTDYEAQRRGGKGKSAARIKEEDFIDRLLVANTHDTILCFSSRGRLY |
| SRR1967922 | VTLSHQGYVKYQPLTDYEAQRRGGKGKSAARIKEEDFIDRLLVANTHDTILCFSSRGRLY |
| SRR8704720 | VTLSHQGYVKYQPLTDYEAQRRGGKGKSAARIKEEDFIDRLLVANTHDTILCFSSRGRLY |
| SRR7216071 | VTLSHQGYVKYQPLTDYEAQRRGGKGKSAARIKEEDFIDRLLVANTHDTILCFSSRGRLY |
| SRR7349175 | VTLSHQGYVKYQPLTDYEAQRRGGKGKSAARIKEEDFIDRLLVANTHDTILCFSSRGRLY |
| SRR7523148 | VTLSHQGYVKYQPLTDYEAQRRGGKGKSAARIKEEDFIDRLLVANTHDTILCFSSRGRLY |
| SRR7523854 | VTLSHQGYVKYQPLTDYEAQRRGGKGKSAARIKEEDFIDRLLVANTHDTILCFSSRGRLY |
| 313865 | VTLSHQGYVKYQPLTDYEAQRRGGKGKSAARIKEEDFIDRLLVANTHDTILCFSSRGRLY |
| SRR7277793 | VTLSHQGYVKYQPLTDYEAQRRGGKGKSAARIKEEDFIDRLLVANTHDTILCFSSRGRLY |
| SRR7343877 | VTLSHQGYVKYQPLTDYEAQRRGGKGKSAARIKEEDFIDRLLVANTHDTILCFSSRGRLY |
| SRR7351477 | VTLSHQGYVKYQPLTDYEAQRRGGKGKSAARIKEEDFIDRLLVANTHDTILCFSSRGRLY |
| SRR5583183 | VTLSHQGYVKYQPLTDYEAQRRGGKGKSAARIKEEDFIDRLLVANTHDTILCFSSRGRLY |
| SRR5585240 | VTLSHQGYVKYQPLTDYEAQRRGGKGKSAARIKEEDFIDRLLVANTHDTILCFSSRGRLY |
| SRR7284317 | VTLSHQGYVKYQPLTDYEAQRRGGKGKSAARIKEEDFIDRLLVANTHDTILCFSSRGRLY |
| SRR7299161 | VTLSHQGYVKYQPLTDYEAQRRGGKGKSAARIKEEDFIDRLLVANTHDTILCFSSRGRLY |
| SRR7401730 | VTLSHQGYVKYQPLTDYEAQRRGGKGKSAARIKEEDFIDRLLVANTHDTILCFSSRGRLY |
| SRR7469092 | VTLSHQGYVKYQPLTDYEAQRRGGKGKSAARIKEEDFIDRLLVANTHDTILCFSSRGRLY |
| SRR7879556 | VTLSHQGYVKYQPLTDYEAQRRGGKGKSAARIKEEDFIDRLLVANTHDTILCFSSRGRLY |
| SRR8526100 | VTLSHQGYVKYQPLTDYEAQRRGGKGKSAARIKEEDFIDRLLVANTHDTILCFSSRGRLY |
| SRR8553991 | VTLSHQGYVKYQPLTDYEAQRRGGKGKSAARIKEEDFIDRLLVANTHDTILCFSSRGRLY |
| SRR7842487 | VTLSHQGYVKYQPLTDYEAQRRGGKGKSAARIKEEDFIDRLLVANTHDTILCFSSRGRLY |
| SRR8054524 | VTLSHQGYVKYQPLTDYEAQRRGGKGKSAARIKEEDFIDRLLVANTHDTILCFSSRGRLY |
| SRR8054525 | VTLSHQGYVKYQPLTDYEAQRRGGKGKSAARIKEEDFIDRLLVANTHDTILCFSSRGRLY |
| SRR8524733 | VTLSHQGYVKYQPLTDYEAQRRGGKGKSAARIKEEDFIDRLLVANTHDTILCFSSRGRLY |
| SRR4093291 | VTLSHQGYVKYQPLTDYEAQRRGGKGKSAARIKEEDFIDRLLVANTHDTILCFSSRGRLY |
| SRR4245549 | VTLSHQGYVKYQPLTDYEAQRRGGKGKSAARIKEEDFIDRLLVANTHDTILCFSSRGRLY |
| SRR3057154 | VTLSHQGYVKYQPLTDYEAQRRGGKGKSAARIKEEDFIDRLLVANTHDTILCFSSRGRLY |
| SRR1726150 | VTLSHQGYVKYQPLTDYEAQRRGGKGKSAARIKEEDFIDRLLVANTHDTILCFSSRGRLY |
| SRR1996141 | VTLSHQGYVKYQPLTDYEAQRRGGKGKSAARIKEEDFIDRLLVANTHDTILCFSSRGRLY |
| SRR1107842 | VTLSHQGYVKYQPLTDYEAQRRGGKGKSAARIKEEDFIDRLLVANTHDTILCFSSRGRLY |
| SRR1157587 | VTLSHQGYVKYQPLTDYEAQRRGGKGKSAARIKEEDFIDRLLVANTHDTILCFSSRGRLY |
| SRR3027706 | VTLSHQGYVKYQPLTDYEAQRRGGKGKSAARIKEEDFIDRLLVANTHDTILCFSSRGRLY |
| SRR3027707 | VTLSHQGYVKYQPLTDYEAQRRGGKGKSAARIKEEDFIDRLLVANTHDTILCFSSRGRLY |
| SRR3027708 | VTLSHQGYVKYQPLTDYEAQRRGGKGKSAARIKEEDFIDRLLVANTHDTILCFSSRGRLY |
| SRR3027710 | VTLSHQGYVKYQPLTDYEAQRRGGKGKSAARIKEEDFIDRLLVANTHDTILCFSSRGRLY |
| SRR3027711 | VTLSHQGYVKYQPLTDYEAQRRGGKGKSAARIKEEDFIDRLLVANTHDTILCFSSRGRLY |
| SRR3027716 | VTLSHQGYVKYQPLTDYEAQRRGGKGKSAARIKEEDFIDRLLVANTHDTILCFSSRGRLY |
| SRR3027717 | VTLSHQGYVKYQPLTDYEAQRRGGKGKSAARIKEEDFIDRLLVANTHDTILCFSSRGRLY |
| SRR3027719 | VTLSHQGYVKYQPLTDYEAQRRGGKGKSAARIKEEDFIDRLLVANTHDTILCFSSRGRLY |
| SRR3027721 | VTLSHQGYVKYQPLTDYEAQRRGGKGKSAARIKEEDFIDRLLVANTHDTILCFSSRGRLY |
| SRR3027723 | VTLSHQGYVKYQPLTDYEAQRRGGKGKSAARIKEEDFIDRLLVANTHDTILCFSSRGRLY |
| SRR3115978 | VTLSHQGYVKYQPLTDYEAQRRGGKGKSAARIKEEDFIDRLLVANTHDTILCFSSRGRLY |
| SRR2534093 | VTLSHQGYVKYQPLTDYEAQRRGGKGKSAARIKEEDFIDRLLVANTHDTILCFSSRGRLY |
| SRR2534094 | VTLSHQGYVKYQPLTDYEAQRRGGKGKSAARIKEEDFIDRLLVANTHDTILCFSSRGRLY |
| SRR2534095 | VTLSHQGYVKYQPLTDYEAQRRGGKGKSAARIKEEDFIDRLLVANTHDTILCFSSRGRLY |
| SRR2534108 | VTLSHQGYVKYQPLTDYEAQRRGGKGKSAARIKEEDFIDRLLVANTHDTILCFSSRGRLY |
| SRR1106464 | VTLSHQGYVKYQPLTDYEAQRRGGKGKSAARIKEEDFIDRLLVANTHDTILCFSSRGRLY |
| SRR1106463 | VTLSHQGYVKYQPLTDYEAQRRGGKGKSAARIKEEDFIDRLLVANTHDTILCFSSRGRLY |
| SRR6949610 | VTLSHQGYVKYQPLTDYEAQRRGGKGKSAARIKEEDFIDRLLVANTHDTILCFSSRGRLY |
| SRR6950452 | VTLSHQGYVKYQPLTDYEAQRRGGKGKSAARIKEEDFIDRLLVANTHDTILCFSSRGRLY |
| ERR2019831 | VTLSHQGYVKYQPLTDYEAQRRGGKGKSAARIKEEDFIDRLLVANTHDTILCFSSRGRLY |
| SRR2085693 | VTLSHQGYVKYQPLTDYEAQRRGGKGKSAARIKEEDFIDRLLVANTHDTILCFSSRGRLY |
| SRR2086898 | VTLSHQGYVKYQPLTDYEAQRRGGKGKSAARIKEEDFIDRLLVANTHDTILCFSSRGRLY |
| SRR2175312 | VTLSHQGYVKYQPLTDYEAQRRGGKGKSAARIKEEDFIDRLLVANTHDTILCFSSRGRLY |
| SRR2175360 | VTLSHQGYVKYQPLTDYEAQRRGGKGKSAARIKEEDFIDRLLVANTHDTILCFSSRGRLY |
| SRR5231997 | VTLSHQGYVKYQPLTDYEAQRRGGKGKSAARIKEEDFIDRLLVANTHDTILCFSSRGRLY |
| SRR5232003 | VTLSHQGYVKYQPLTDYEAQRRGGKGKSAARIKEEDFIDRLLVANTHDTILCFSSRGRLY |
| SRR5232015 | VTLSHQGYVKYQPLTDYEAQRRGGKGKSAARIKEEDFIDRLLVANTHDTILCFSSRGRLY |
| SRR949434 | VTLSHQGYVKYQPLTDYEAQRRGGKGKSAARIKEEDFIDRLLVANTHDTILCFSSRGRLY |
| SRR3216575 | VTLSHQGYVKYQPLTDYEAQRRGGKGKSAARIKEEDFIDRLLVANTHDTILCFSSRGRLY |
| SRR5205342 | VTLSHQGYVKYQPLTDYEAQRRGGKGKSAARIKEEDFIDRLLVANTHDTILCFSSRGRLY |
| SRR1501669 | VTLSHQGYVKYQPLTDYEAQRRGGKGKSAARIKEEDFIDRLLVANTHDTILCFSSRGRLY |
| SRR5209740 | VTLSHQGYVKYQPLTDYEAQRRGGKGKSAARIKEEDFIDRLLVANTHDTILCFSSRGRLY |
| SRR3240355 | VTLSHQGYVKYQPLTDYEAQRRGGKGKSAARIKEEDFIDRLLVANTHDTILCFSSRGRLY |
| SRR3392777 | VTLSHQGYVKYQPLTDYEAQRRGGKGKSAARIKEEDFIDRLLVANTHDTILCFSSRGRLY |
| SRR3593671 | VTLSHQGYVKYQPLTDYEAQRRGGKGKSAARIKEEDFIDRLLVANTHDTILCFSSRGRLY |
| SRR5413290 | VTLSHQGYVKYQPLTDYEAQRRGGKGKSAARIKEEDFIDRLLVANTHDTILCFSSRGRLY |
| SRR5590269 | VTLSHQGYVKYQPLTDYEAQRRGGKGKSAARIKEEDFIDRLLVANTHDTILCFSSRGRLY |
| SRR5812103 | VTLSHQGYVKYQPLTDYEAQRRGGKGKSAARIKEEDFIDRLLVANTHDTILCFSSRGRLY |
| SRR2830941 | VTLSHQGYVKYQPLTDYEAQRRGGKGKSAARIKEEDFIDRLLVANTHDTILCFSSRGRLY |
| SRR2830966 | VTLSHQGYVKYQPLTDYEAQRRGGKGKSAARIKEEDFIDRLLVANTHDTILCFSSRGRLY |
| SRR3137270 | VTLSHQGYVKYQPLTDYEAQRRGGKGKSAARIKEEDFIDRLLVANTHDTILCFSSRGRLY |
| SRR3137271 | VTLSHQGYVKYQPLTDYEAQRRGGKGKSAARIKEEDFIDRLLVANTHDTILCFSSRGRLY |
| ERR526807 | VTLSHQGYVKYQPLTDYEAQRRGGKGKSAARIKEEDFIDRLLVANTHDTILCFSSRGRLY |
| ERR2197922 | VTLSHQGYVKYQPLTDYEAQRRGGKGKSAARIKEEDFIDRLLVANTHDTILCFSSRGRLY |
| ERR2197923 | VTLSHQGYVKYQPLTDYEAQRRGGKGKSAARIKEEDFIDRLLVANTHDTILCFSSRGRLY |
| ERR2197924 | VTLSHQGYVKYQPLTDYEAQRRGGKGKSAARIKEEDFIDRLLVANTHDTILCFSSRGRLY |
| ERR2197925 | VTLSHQGYVKYQPLTDYEAQRRGGKGKSAARIKEEDFIDRLLVANTHDTILCFSSRGRLY |
| ERR2197927 | VTLSHQGYVKYQPLTDYEAQRRGGKGKSAARIKEEDFIDRLLVANTHDTILCFSSRGRLY |
| ERR2197929 | VTLSHQGYVKYQPLTDYEAQRRGGKGKSAARIKEEDFIDRLLVANTHDTILCFSSRGRLY |
| SRR1648149 | VTLSHQGYVKYQPLTDYEAQRRGGKGKSAARIKEEDFIDRLLVANTHDTILCFSSRGRLY |
| SRR1048299 | VTLSHQGYVKYQPLTDYEAQRRGGKGKSAARIKEEDFIDRLLVANTHDTILCFSSRGRLY |
| SRR1300677 | VTLSHQGYVKYQPLTDYEAQRRGGKGKSAARIKEEDFIDRLLVANTHDTILCFSSRGRLY |
| SRR1288356 | VTLSHQGYVKYQPLTDYEAQRRGGKGKSAARIKEEDFIDRLLVANTHDTILCFSSRGRLY |
| SRR7426190 | VTLSHQGYVKYQPLTDYEAQRRGGKGKSAARIKEEDFIDRLLVANTHDTILCFSSRGRLY |
| SRR7426192 | VTLSHQGYVKYQPLTDYEAQRRGGKGKSAARIKEEDFIDRLLVANTHDTILCFSSRGRLY |
| SRR7426193 | VTLSHQGYVKYQPLTDYEAQRRGGKGKSAARIKEEDFIDRLLVANTHDTILCFSSRGRLY |
| SRR7441832 | VTLSHQGYVKYQPLTDYEAQRRGGKGKSAARIKEEDFIDRLLVANTHDTILCFSSRGRLY |
| SRR7426179 | VTLSHQGYVKYQPLTDYEAQRRGGKGKSAARIKEEDFIDRLLVANTHDTILCFSSRGRLY |
| SRR7439238 | VTLSHQGYVKYQPLTDYEAQRRGGKGKSAARIKEEDFIDRLLVANTHDTILCFSSRGRLY |
| SRR7439244 | VTLSHQGYVKYQPLTDYEAQRRGGKGKSAARIKEEDFIDRLLVANTHDTILCFSSRGRLY |
| SRR7439259 | VTLSHQGYVKYQPLTDYEAQRRGGKGKSAARIKEEDFIDRLLVANTHDTILCFSSRGRLY |
| SRR7439260 | VTLSHQGYVKYQPLTDYEAQRRGGKGKSAARIKEEDFIDRLLVANTHDTILCFSSRGRLY |
| SRR7441786 | VTLSHQGYVKYQPLTDYEAQRRGGKGKSAARIKEEDFIDRLLVANTHDTILCFSSRGRLY |
| SRR7441797 | VTLSHQGYVKYQPLTDYEAQRRGGKGKSAARIKEEDFIDRLLVANTHDTILCFSSRGRLY |
| ERR1759093 | VTLSHQGYVKYQPLTDYEAQRRGGKGKSAARIKEEDFIDRLLVANTHDTILCFSSRGRLY |
| ERR2580275 | VTLSHQGYVKYQPLTDYEAQRRGGKGKSAARIKEEDFIDRLLVANTHDTILCFSSRGRLY |
| ERR1759204 | VTLSHQGYVKYQPLTDYEAQRRGGKGKSAARIKEEDFIDRLLVANTHDTILCFSSRGRLY |
| SRR1300699 | VTLSHQGYVKYQPLTDYEAQRRGGKGKSAARIKEEDFIDRLLVANTHDTILCFSSRGRLY |
| S_0825_17 | VTLSHQGYVKYQPLTDYEAQRRGGKGKSAARIKEEDFIDRLLVANTHDTILCFSSRGRLY |
| SRR1958215 | VTLSHQGYVKYQPLTDYEAQRRGGKGKSAARIKEEDFIDRLLVANTHDTILCFSSRGRLY |
| SRR1958540 | VTLSHQGYVKYQPLTDYEAQRRGGKGKSAARIKEEDFIDRLLVANTHDTILCFSSRGRLY |
| SRR1958636 | VTLSHQGYVKYQPLTDYEAQRRGGKGKSAARIKEEDFIDRLLVANTHDTILCFSSRGRLY |
| SRR1959422 | VTLSHQGYVKYQPLTDYEAQRRGGKGKSAARIKEEDFIDRLLVANTHDTILCFSSRGRLY |
| SRR1959427 | VTLSHQGYVKYQPLTDYEAQRRGGKGKSAARIKEEDFIDRLLVANTHDTILCFSSRGRLY |
| SRR1960226 | VTLSHQGYVKYQPLTDYEAQRRGGKGKSAARIKEEDFIDRLLVANTHDTILCFSSRGRLY |
| SRR1963498 | VTLSHQGYVKYQPLTDYEAQRRGGKGKSAARIKEEDFIDRLLVANTHDTILCFSSRGRLY |
| SRR1965947 | VTLSHQGYVKYQPLTDYEAQRRGGKGKSAARIKEEDFIDRLLVANTHDTILCFSSRGRLY |
| SRR1966125 | VTLSHQGYVKYQPLTDYEAQRRGGKGKSAARIKEEDFIDRLLVANTHDTILCFSSRGRLY |
| SRR1966330 | VTLSHQGYVKYQPLTDYEAQRRGGKGKSAARIKEEDFIDRLLVANTHDTILCFSSRGRLY |
| SRR1966565 | VTLSHQGYVKYQPLTDYEAQRRGGKGKSAARIKEEDFIDRLLVANTHDTILCFSSRGRLY |
| SRR1966864 | VTLSHQGYVKYQPLTDYEAQRRGGKGKSAARIKEEDFIDRLLVANTHDTILCFSSRGRLY |
| SRR1966989 | VTLSHQGYVKYQPLTDYEAQRRGGKGKSAARIKEEDFIDRLLVANTHDTILCFSSRGRLY |
| SRR1967688 | VTLSHQGYVKYQPLTDYEAQRRGGKGKSAARIKEEDFIDRLLVANTHDTILCFSSRGRLY |
| SRR1967733 | VTLSHQGYVKYQPLTDYEAQRRGGKGKSAARIKEEDFIDRLLVANTHDTILCFSSRGRLY |
| SRR1967746 | VTLSHQGYVKYQPLTDYEAQRRGGKGKSAARIKEEDFIDRLLVANTHDTILCFSSRGRLY |
| SRR1968341 | VTLSHQGYVKYQPLTDYEAQRRGGKGKSAARIKEEDFIDRLLVANTHDTILCFSSRGRLY |
| SRR1968456 | VTLSHQGYVKYQPLTDYEAQRRGGKGKSAARIKEEDFIDRLLVANTHDTILCFSSRGRLY |
| SRR1968465 | VTLSHQGYVKYQPLTDYEAQRRGGKGKSAARIKEEDFIDRLLVANTHDTILCFSSRGRLY |
| SRR1968761 | VTLSHQGYVKYQPLTDYEAQRRGGKGKSAARIKEEDFIDRLLVANTHDTILCFSSRGRLY |
| SRR1969047 | VTLSHQGYVKYQPLTDYEAQRRGGKGKSAARIKEEDFIDRLLVANTHDTILCFSSRGRLY |
| SRR1969255 | VTLSHQGYVKYQPLTDYEAQRRGGKGKSAARIKEEDFIDRLLVANTHDTILCFSSRGRLY |
| SRR1969412 | VTLSHQGYVKYQPLTDYEAQRRGGKGKSAARIKEEDFIDRLLVANTHDTILCFSSRGRLY |
| SRR1969524 | VTLSHQGYVKYQPLTDYEAQRRGGKGKSAARIKEEDFIDRLLVANTHDTILCFSSRGRLY |
| SRR1969584 | VTLSHQGYVKYQPLTDYEAQRRGGKGKSAARIKEEDFIDRLLVANTHDTILCFSSRGRLY |
| SRR1969648 | VTLSHQGYVKYQPLTDYEAQRRGGKGKSAARIKEEDFIDRLLVANTHDTILCFSSRGRLY |
| SRR1969804 | VTLSHQGYVKYQPLTDYEAQRRGGKGKSAARIKEEDFIDRLLVANTHDTILCFSSRGRLY |
| SRR1970221 | VTLSHQGYVKYQPLTDYEAQRRGGKGKSAARIKEEDFIDRLLVANTHDTILCFSSRGRLY |
| SRR1970268 | VTLSHQGYVKYQPLTDYEAQRRGGKGKSAARIKEEDFIDRLLVANTHDTILCFSSRGRLY |
| SRR1965862 | VTLSHQGYVKYQPLTDYEAQRRGGKGKSAARIKEEDFIDRLLVANTHDTILCFSSRGRLY |
| SRR1967363 | VTLSHQGYVKYQPLTDYEAQRRGGKGKSAARIKEEDFIDRLLVANTHDTILCFSSRGRLY |
| SRR1968276 | VTLSHQGYVKYQPLTDYEAQRRGGKGKSAARIKEEDFIDRLLVANTHDTILCFSSRGRLY |
| SRR1968967 | VTLSHQGYVKYQPLTDYEAQRRGGKGKSAARIKEEDFIDRLLVANTHDTILCFSSRGRLY |
| SRR3321531 | VTLSHQGYVKYQPLTDYEAQRRGGKGKSAARIKEEDFIDRLLVANTHDTILCFSSRGRLY |
| SRR3321883 | VTLSHQGYVKYQPLTDYEAQRRGGKGKSAARIKEEDFIDRLLVANTHDTILCFSSRGRLY |
| SRR3322413 | VTLSHQGYVKYQPLTDYEAQRRGGKGKSAARIKEEDFIDRLLVANTHDTILCFSSRGRLY |
| SRR3323012 | VTLSHQGYVKYQPLTDYEAQRRGGKGKSAARIKEEDFIDRLLVANTHDTILCFSSRGRLY |
| SRR5194289 | VTLSHQGYVKYQPLTDYEAQRRGGKGKSAARIKEEDFIDRLLVANTHDTILCFSSRGRLY |
| SRR7163798 | VTLSHQGYVKYQPLTDYEAQRRGGKGKSAARIKEEDFIDRLLVANTHDTILCFSSRGRLY |
| SRR7172610 | VTLSHQGYVKYQPLTDYEAQRRGGKGKSAARIKEEDFIDRLLVANTHDTILCFSSRGRLY |
| SRR7204568 | VTLSHQGYVKYQPLTDYEAQRRGGKGKSAARIKEEDFIDRLLVANTHDTILCFSSRGRLY |
| SRR7223230 | VTLSHQGYVKYQPLTDYEAQRRGGKGKSAARIKEEDFIDRLLVANTHDTILCFSSRGRLY |
| SRR7230675 | VTLSHQGYVKYQPLTDYEAQRRGGKGKSAARIKEEDFIDRLLVANTHDTILCFSSRGRLY |
| SRR7278056 | VTLSHQGYVKYQPLTDYEAQRRGGKGKSAARIKEEDFIDRLLVANTHDTILCFSSRGRLY |
| SRR7278086 | VTLSHQGYVKYQPLTDYEAQRRGGKGKSAARIKEEDFIDRLLVANTHDTILCFSSRGRLY |
| SRR7285841 | VTLSHQGYVKYQPLTDYEAQRRGGKGKSAARIKEEDFIDRLLVANTHDTILCFSSRGRLY |
| SRR7292625 | VTLSHQGYVKYQPLTDYEAQRRGGKGKSAARIKEEDFIDRLLVANTHDTILCFSSRGRLY |
| SRR7292665 | VTLSHQGYVKYQPLTDYEAQRRGGKGKSAARIKEEDFIDRLLVANTHDTILCFSSRGRLY |
| SRR7297965 | VTLSHQGYVKYQPLTDYEAQRRGGKGKSAARIKEEDFIDRLLVANTHDTILCFSSRGRLY |
| SRR7350726 | VTLSHQGYVKYQPLTDYEAQRRGGKGKSAARIKEEDFIDRLLVANTHDTILCFSSRGRLY |
| SRR7410328 | VTLSHQGYVKYQPLTDYEAQRRGGKGKSAARIKEEDFIDRLLVANTHDTILCFSSRGRLY |
| SRR7474665 | VTLSHQGYVKYQPLTDYEAQRRGGKGKSAARIKEEDFIDRLLVANTHDTILCFSSRGRLY |
| SRR7523184 | VTLSHQGYVKYQPLTDYEAQRRGGKGKSAARIKEEDFIDRLLVANTHDTILCFSSRGRLY |
| SRR7187264 | VTLSHQGYVKYQPLTDYEAQRRGGKGKSAARIKEEDFIDRLLVANTHDTILCFSSRGRLY |
| SRR7204445 | VTLSHQGYVKYQPLTDYEAQRRGGKGKSAARIKEEDFIDRLLVANTHDTILCFSSRGRLY |
| SRR7285641 | VTLSHQGYVKYQPLTDYEAQRRGGKGKSAARIKEEDFIDRLLVANTHDTILCFSSRGRLY |
| SRR7286695 | VTLSHQGYVKYQPLTDYEAQRRGGKGKSAARIKEEDFIDRLLVANTHDTILCFSSRGRLY |
| SRR7286705 | VTLSHQGYVKYQPLTDYEAQRRGGKGKSAARIKEEDFIDRLLVANTHDTILCFSSRGRLY |
| SRR7292931 | VTLSHQGYVKYQPLTDYEAQRRGGKGKSAARIKEEDFIDRLLVANTHDTILCFSSRGRLY |
| SRR7310349 | VTLSHQGYVKYQPLTDYEAQRRGGKGKSAARIKEEDFIDRLLVANTHDTILCFSSRGRLY |
| SRR7351616 | VTLSHQGYVKYQPLTDYEAQRRGGKGKSAARIKEEDFIDRLLVANTHDTILCFSSRGRLY |
| SRR7414818 | VTLSHQGYVKYQPLTDYEAQRRGGKGKSAARIKEEDFIDRLLVANTHDTILCFSSRGRLY |
| SRR7426480 | VTLSHQGYVKYQPLTDYEAQRRGGKGKSAARIKEEDFIDRLLVANTHDTILCFSSRGRLY |
| SRR5584105 | VTLSHQGYVKYQPLTDYEAQRRGGKGKSAARIKEEDFIDRLLVANTHDTILCFSSRGRLY |
| SRR5584565 | VTLSHQGYVKYQPLTDYEAQRRGGKGKSAARIKEEDFIDRLLVANTHDTILCFSSRGRLY |
| SRR5584614 | VTLSHQGYVKYQPLTDYEAQRRGGKGKSAARIKEEDFIDRLLVANTHDTILCFSSRGRLY |
| SRR5631543 | VTLSHQGYVKYQPLTDYEAQRRGGKGKSAARIKEEDFIDRLLVANTHDTILCFSSRGRLY |
| SRR5631553 | VTLSHQGYVKYQPLTDYEAQRRGGKGKSAARIKEEDFIDRLLVANTHDTILCFSSRGRLY |
| SRR7123196 | VTLSHQGYVKYQPLTDYEAQRRGGKGKSAARIKEEDFIDRLLVANTHDTILCFSSRGRLY |
| SRR7163819 | VTLSHQGYVKYQPLTDYEAQRRGGKGKSAARIKEEDFIDRLLVANTHDTILCFSSRGRLY |
| SRR7163920 | VTLSHQGYVKYQPLTDYEAQRRGGKGKSAARIKEEDFIDRLLVANTHDTILCFSSRGRLY |
| SRR7209528 | VTLSHQGYVKYQPLTDYEAQRRGGKGKSAARIKEEDFIDRLLVANTHDTILCFSSRGRLY |
| SRR7249868 | VTLSHQGYVKYQPLTDYEAQRRGGKGKSAARIKEEDFIDRLLVANTHDTILCFSSRGRLY |
| SRR7278088 | VTLSHQGYVKYQPLTDYEAQRRGGKGKSAARIKEEDFIDRLLVANTHDTILCFSSRGRLY |
| SRR7285788 | VTLSHQGYVKYQPLTDYEAQRRGGKGKSAARIKEEDFIDRLLVANTHDTILCFSSRGRLY |
| SRR7286789 | VTLSHQGYVKYQPLTDYEAQRRGGKGKSAARIKEEDFIDRLLVANTHDTILCFSSRGRLY |
| SRR7286886 | VTLSHQGYVKYQPLTDYEAQRRGGKGKSAARIKEEDFIDRLLVANTHDTILCFSSRGRLY |
| SRR7310632 | VTLSHQGYVKYQPLTDYEAQRRGGKGKSAARIKEEDFIDRLLVANTHDTILCFSSRGRLY |
| SRR7350631 | VTLSHQGYVKYQPLTDYEAQRRGGKGKSAARIKEEDFIDRLLVANTHDTILCFSSRGRLY |
| SRR7458741 | VTLSHQGYVKYQPLTDYEAQRRGGKGKSAARIKEEDFIDRLLVANTHDTILCFSSRGRLY |
| SRR7480280 | VTLSHQGYVKYQPLTDYEAQRRGGKGKSAARIKEEDFIDRLLVANTHDTILCFSSRGRLY |
| SRR7523660 | VTLSHQGYVKYQPLTDYEAQRRGGKGKSAARIKEEDFIDRLLVANTHDTILCFSSRGRLY |
| SRR7523775 | VTLSHQGYVKYQPLTDYEAQRRGGKGKSAARIKEEDFIDRLLVANTHDTILCFSSRGRLY |
| SRR7251101 | VTLSHQGYVKYQPLTDYEAQRRGGKGKSAARIKEEDFIDRLLVANTHDTILCFSSRGRLY |
| SRR7284299 | VTLSHQGYVKYQPLTDYEAQRRGGKGKSAARIKEEDFIDRLLVANTHDTILCFSSRGRLY |
| SRR7285738 | VTLSHQGYVKYQPLTDYEAQRRGGKGKSAARIKEEDFIDRLLVANTHDTILCFSSRGRLY |
| SRR7310640 | VTLSHQGYVKYQPLTDYEAQRRGGKGKSAARIKEEDFIDRLLVANTHDTILCFSSRGRLY |
| SRR7349159 | VTLSHQGYVKYQPLTDYEAQRRGGKGKSAARIKEEDFIDRLLVANTHDTILCFSSRGRLY |
| SRR7474873 | VTLSHQGYVKYQPLTDYEAQRRGGKGKSAARIKEEDFIDRLLVANTHDTILCFSSRGRLY |
| SRR7495689 | VTLSHQGYVKYQPLTDYEAQRRGGKGKSAARIKEEDFIDRLLVANTHDTILCFSSRGRLY |
| SRR7495752 | VTLSHQGYVKYQPLTDYEAQRRGGKGKSAARIKEEDFIDRLLVANTHDTILCFSSRGRLY |
| ----------------------------------------------------------------------------- | |
| S16BD08730 | WMKVYQLPEASRGARGRPIVNLLPLEANERITAILPVREYEEGVNVFMATASGTVKKTAL |
| S18BD00684 | WMKVYQLPEASRGARGRPIVNLLPLEANERITAILPVREYEEGVNVFMATASGTVKKTAL |
| S18BD03994 | WMKVYQLPEASRGARGRPIVNLLPLEANERITAILPVREYEEGVNVFMATASGTVKKTAL |
| S18BD05011 | WMKVYQLPEASRGARGRPIVNLLPLEANERITAILPVREYEEGVNVFMATASGTVKKTAL |
| RKI_16-03723 | WMKVYQLPEASRGARGRPIVNLLPLEANERITAILPVREYEEGVNVFMATASGTVKKTAL |
| RKI_16-04315 | WMKVYQLPEASRGARGRPIVNLLPLEANERITAILPVREYEEGVNVFMATASGTVKKTAL |
| RKI_17-02304 | WMKVYQLPEASRGARGRPIVNLLPLEANERITAILPVREYEEGVNVFMATASGTVKKTAL |
| RKI_17-02411 | WMKVYQLPEASRGARGRPIVNLLPLEANERITAILPVREYEEGVNVFMATASGTVKKTAL |
| RKI_17-02757 | WMKVYQLPEASRGARGRPIVNLLPLEANERITAILPVREYEEGVNVFMATASGTVKKTAL |
| RKI_17-04797 | WMKVYQLPEASRGARGRPIVNLLPLEANERITAILPVREYEEGVNVFMATASGTVKKTAL |
| RKI_17-06869 | WMKVYQLPEASRGARGRPIVNLLPLEANERITAILPVREYEEGVNVFMATASGTVKKTAL |
| ERR2580277 | WMKVYQLPEASRGARGRPIVNLLPLEANERITAILPVREYEEGVNVFMATASGTVKKTAL |
| ERR2580276 | WMKVYQLPEASRGARGRPIVNLLPLEANERITAILPVREYEEGVNVFMATASGTVKKTAL |
| ERR2580273 | WMKVYQLPEASRGARGRPIVNLLPLEANERITAILPVREYEEGVNVFMATASGTVKKTAL |
| ERR2580274 | WMKVYQLPEASRGARGRPIVNLLPLEANERITAILPVREYEEGVNVFMATASGTVKKTAL |
| ERR2173656 | WMKVYQLPEASRGARGRPIVNLLPLEANERITAILPVREYEEGVNVFMATASGTVKKTAL |
| 17041676 | WMKVYQLPEASRGARGRPIVNLLPLEANERITAILPVREYEEGVNVFMATASGTVKKTAL |
| MT16-000061 | WMKVYQLPEASRGARGRPIVNLLPLEANERITAILPVREYEEGVNVFMATASGTVKKTAL |
| MT16-019416 | WMKVYQLPEASRGARGRPIVNLLPLEANERITAILPVREYEEGVNVFMATASGTVKKTAL |
| MT16-027865 | WMKVYQLPEASRGARGRPIVNLLPLEANERITAILPVREYEEGVNVFMATASGTVKKTAL |
| MT16-031693 | WMKVYQLPEASRGARGRPIVNLLPLEANERITAILPVREYEEGVNVFMATASGTVKKTAL |
| MT16-040253 | WMKVYQLPEASRGARGRPIVNLLPLEANERITAILPVREYEEGVNVFMATASGTVKKTAL |
| MT16-045379 | WMKVYQLPEASRGARGRPIVNLLPLEANERITAILPVREYEEGVNVFMATASGTVKKTAL |
| MT16-442728 | WMKVYQLPEASRGARGRPIVNLLPLEANERITAILPVREYEEGVNVFMATASGTVKKTAL |
| MT16-462857 | WMKVYQLPEASRGARGRPIVNLLPLEANERITAILPVREYEEGVNVFMATASGTVKKTAL |
| MT16-480196 | WMKVYQLPEASRGARGRPIVNLLPLEANERITAILPVREYEEGVNVFMATASGTVKKTAL |
| MT16-861555 | WMKVYQLPEASRGARGRPIVNLLPLEANERITAILPVREYEEGVNVFMATASGTVKKTAL |
| MT17-076833 | WMKVYQLPEASRGARGRPIVNLLPLEANERITAILPVREYEEGVNVFMATASGTVKKTAL |
| MT17-110677 | WMKVYQLPEASRGARGRPIVNLLPLEANERITAILPVREYEEGVNVFMATASGTVKKTAL |
| MT17-131730 | WMKVYQLPEASRGARGRPIVNLLPLEANERITAILPVREYEEGVNVFMATASGTVKKTAL |
| MT17-140890 | WMKVYQLPEASRGARGRPIVNLLPLEANERITAILPVREYEEGVNVFMATASGTVKKTAL |
| MT17-141840 | WMKVYQLPEASRGARGRPIVNLLPLEANERITAILPVREYEEGVNVFMATASGTVKKTAL |
| MT17-152488 | WMKVYQLPEASRGARGRPIVNLLPLEANERITAILPVREYEEGVNVFMATASGTVKKTAL |
| MT17-157311 | WMKVYQLPEASRGARGRPIVNLLPLEANERITAILPVREYEEGVNVFMATASGTVKKTAL |
| MT17-161645 | WMKVYQLPEASRGARGRPIVNLLPLEANERITAILPVREYEEGVNVFMATASGTVKKTAL |
| MT17-167951 | WMKVYQLPEASRGARGRPIVNLLPLEANERITAILPVREYEEGVNVFMATASGTVKKTAL |
| MT18-217732 | WMKVYQLPEASRGARGRPIVNLLPLEANERITAILPVREYEEGVNVFMATASGTVKKTAL |
| MT18-252580 | WMKVYQLPEASRGARGRPIVNLLPLEANERITAILPVREYEEGVNVFMATASGTVKKTAL |
| RIVM_H_2009-01 | WMKVYQLPEASRGARGRPIVNLLPLEANERITAILPVREYEEGVNVFMATASGTVKKTAL |
| RIVM_H_2010-01 | WMKVYQLPEASRGARGRPIVNLLPLEANERITAILPVREYEEGVNVFMATASGTVKKTAL |
| RIVM_H_2010-02 | WMKVYQLPEASRGARGRPIVNLLPLEANERITAILPVREYEEGVNVFMATASGTVKKTAL |
| RIVM_H_2011-01 | WMKVYQLPEASRGARGRPIVNLLPLEANERITAILPVREYEEGVNVFMATASGTVKKTAL |
| RIVM_H_2011-02 | WMKVYQLPEASRGARGRPIVNLLPLEANERITAILPVREYEEGVNVFMATASGTVKKTAL |
| RIVM_H_2011-03 | WMKVYQLPEASRGARGRPIVNLLPLEANERITAILPVREYEEGVNVFMATASGTVKKTAL |
| RIVM_H_2013-01 | WMKVYQLPEASRGARGRPIVNLLPLEANERITAILPVREYEEGVNVFMATASGTVKKTAL |
| RIVM_H_2013-02 | WMKVYQLPEASRGARGRPIVNLLPLEANERITAILPVREYEEGVNVFMATASGTVKKTAL |
| RIVM_H_2014-01 | WMKVYQLPEASRGARGRPIVNLLPLEANERITAILPVREYEEGVNVFMATASGTVKKTAL |
| RIVM_H_2014-02 | WMKVYQLPEASRGARGRPIVNLLPLEANERITAILPVREYEEGVNVFMATASGTVKKTAL |
| RIVM_H_2016-01 | WMKVYQLPEASRGARGRPIVNLLPLEANERITAILPVREYEEGVNVFMATASGTVKKTAL |
| RIVM_H_2016-02 | WMKVYQLPEASRGARGRPIVNLLPLEANERITAILPVREYEEGVNVFMATASGTVKKTAL |
| RIVM_H_2016-03 | WMKVYQLPEASRGARGRPIVNLLPLEANERITAILPVREYEEGVNVFMATASGTVKKTAL |
| RIVM_H_2016-04 | WMKVYQLPEASRGARGRPIVNLLPLEANERITAILPVREYEEGVNVFMATASGTVKKTAL |
| RIVM_H_2016-05 | WMKVYQLPEASRGARGRPIVNLLPLEANERITAILPVREYEEGVNVFMATASGTVKKTAL |
| RIVM_H_2016-06 | WMKVYQLPEASRGARGRPIVNLLPLEANERITAILPVREYEEGVNVFMATASGTVKKTAL |
| RIVM_H_2016-07 | WMKVYQLPEASRGARGRPIVNLLPLEANERITAILPVREYEEGVNVFMATASGTVKKTAL |
| RIVM_H_2016-08 | WMKVYQLPEASRGARGRPIVNLLPLEANERITAILPVREYEEGVNVFMATASGTVKKTAL |
| RIVM_H_2016-09 | WMKVYQLPEASRGARGRPIVNLLPLEANERITAILPVREYEEGVNVFMATASGTVKKTAL |
| RIVM_H_2016-10 | WMKVYQLPEASRGARGRPIVNLLPLEANERITAILPVREYEEGVNVFMATASGTVKKTAL |
| RIVM_H_2016-11 | WMKVYQLPEASRGARGRPIVNLLPLEANERITAILPVREYEEGVNVFMATASGTVKKTAL |
| RIVM_H_2016-12 | WMKVYQLPEASRGARGRPIVNLLPLEANERITAILPVREYEEGVNVFMATASGTVKKTAL |
| RIVM_H_2016-13 | WMKVYQLPEASRGARGRPIVNLLPLEANERITAILPVREYEEGVNVFMATASGTVKKTAL |
| RIVM_H_2016-14 | WMKVYQLPEASRGARGRPIVNLLPLEANERITAILPVREYEEGVNVFMATASGTVKKTAL |
| RIVM_H_2016-15 | WMKVYQLPEASRGARGRPIVNLLPLEANERITAILPVREYEEGVNVFMATASGTVKKTAL |
| RIVM_H_2017-01 | WMKVYQLPEASRGARGRPIVNLLPLEANERITAILPVREYEEGVNVFMATASGTVKKTAL |
| RIVM_H_2017-02 | WMKVYQLPEASRGARGRPIVNLLPLEANERITAILPVREYEEGVNVFMATASGTVKKTAL |
| RIVM_H_2017-03 | WMKVYQLPEASRGARGRPIVNLLPLEANERITAILPVREYEEGVNVFMATASGTVKKTAL |
| RIVM_H_2017-04 | WMKVYQLPEASRGARGRPIVNLLPLEANERITAILPVREYEEGVNVFMATASGTVKKTAL |
| RIVM_H_2017-05 | WMKVYQLPEASRGARGRPIVNLLPLEANERITAILPVREYEEGVNVFMATASGTVKKTAL |
| RIVM_H_2017-06 | WMKVYQLPEASRGARGRPIVNLLPLEANERITAILPVREYEEGVNVFMATASGTVKKTAL |
| RIVM_H_2017-07 | WMKVYQLPEASRGARGRPIVNLLPLEANERITAILPVREYEEGVNVFMATASGTVKKTAL |
| RIVM_H_2017-08 | WMKVYQLPEASRGARGRPIVNLLPLEANERITAILPVREYEEGVNVFMATASGTVKKTAL |
| RIVM_H_2017-09 | WMKVYQLPEASRGARGRPIVNLLPLEANERITAILPVREYEEGVNVFMATASGTVKKTAL |
| RIVM_H_2017-10 | WMKVYQLPEASRGARGRPIVNLLPLEANERITAILPVREYEEGVNVFMATASGTVKKTAL |
| RIVM_H_2017-11 | WMKVYQLPEASRGARGRPIVNLLPLEANERITAILPVREYEEGVNVFMATASGTVKKTAL |
| RIVM_H_2017-12 | WMKVYQLPEASRGARGRPIVNLLPLEANERITAILPVREYEEGVNVFMATASGTVKKTAL |
| RIVM_H_2017-13 | WMKVYQLPEASRGARGRPIVNLLPLEANERITAILPVREYEEGVNVFMATASGTVKKTAL |
| RIVM_H_2017-14 | WMKVYQLPEASRGARGRPIVNLLPLEANERITAILPVREYEEGVNVFMATASGTVKKTAL |
| RIVM_H_2017-15 | WMKVYQLPEASRGARGRPIVNLLPLEANERITAILPVREYEEGVNVFMATASGTVKKTAL |
| RIVM_H_2017-16 | WMKVYQLPEASRGARGRPIVNLLPLEANERITAILPVREYEEGVNVFMATASGTVKKTAL |
| RIVM_H_2017-17 | WMKVYQLPEASRGARGRPIVNLLPLEANERITAILPVREYEEGVNVFMATASGTVKKTAL |
| RIVM_H_2017-18 | WMKVYQLPEASRGARGRPIVNLLPLEANERITAILPVREYEEGVNVFMATASGTVKKTAL |
| RIVM_H_2017-19 | WMKVYQLPEASRGARGRPIVNLLPLEANERITAILPVREYEEGVNVFMATASGTVKKTAL |
| 15EP001483 | WMKVYQLPEASRGARGRPIVNLLPLEANERITAILPVREYEEGVNVFMATASGTVKKTAL |
| 17EP002363 | WMKVYQLPEASRGARGRPIVNLLPLEANERITAILPVREYEEGVNVFMATASGTVKKTAL |
| S_0812_17 | WMKVYQLPEASRGARGRPIVNLLPLEANERITAILPVREYEEGVNVFMATASGTVKKTAL |
| SRR1957844 | WMKVYQLPEASRGARGRPIVNLLPLEANERITAILPVREYEEGVNVFMATASGTVKKTAL |
| SRR1958654 | WMKVYQLPEASRGARGRPIVNLLPLEANERITAILPVREYEEGVNVFMATASGTVKKTAL |
| SRR1965077 | WMKVYQLPEASRGARGRPIVNLLPLEANERITAILPVREYEEGVNVFMATASGTVKKTAL |
| SRR1966369 | WMKVYQLPEASRGARGRPIVNLLPLEANERITAILPVREYEEGVNVFMATASGTVKKTAL |
| SRR1967117 | WMKVYQLPEASRGARGRPIVNLLPLEANERITAILPVREYEEGVNVFMATASGTVKKTAL |
| SRR1967922 | WMKVYQLPEASRGARGRPIVNLLPLEANERITAILPVREYEEGVNVFMATASGTVKKTAL |
| SRR8704720 | WMKVYQLPEASRGARGRPIVNLLPLEANERITAILPVREYEEGVNVFMATASGTVKKTAL |
| SRR7216071 | WMKVYQLPEASRGARGRPIVNLLPLEANERITAILPVREYEEGVNVFMATASGTVKKTAL |
| SRR7349175 | WMKVYQLPEASRGARGRPIVNLLPLEANERITAILPVREYEEGVNVFMATASGTVKKTAL |
| SRR7523148 | WMKVYQLPEASRGARGRPIVNLLPLEANERITAILPVREYEEGVNVFMATASGTVKKTAL |
| SRR7523854 | WMKVYQLPEASRGARGRPIVNLLPLEANERITAILPVREYEEGVNVFMATASGTVKKTAL |
| 313865 | WMKVYQLPEASRGARGRPIVNLLPLEANERITAILPVREYEEGVNVFMATASGTVKKTAL |
| SRR7277793 | WMKVYQLPEASRGARGRPIVNLLPLEANERITAILPVREYEEGVNVFMATASGTVKKTAL |
| SRR7343877 | WMKVYQLPEASRGARGRPIVNLLPLEANERITAILPVREYEEGVNVFMATASGTVKKTAL |
| SRR7351477 | WMKVYQLPEASRGARGRPIVNLLPLEANERITAILPVREYEEGVNVFMATASGTVKKTAL |
| SRR5583183 | WMKVYQLPEASRGARGRPIVNLLPLEANERITAILPVREYEEGVNVFMATASGTVKKTAL |
| SRR5585240 | WMKVYQLPEASRGARGRPIVNLLPLEANERITAILPVREYEEGVNVFMATASGTVKKTAL |
| SRR7284317 | WMKVYQLPEASRGARGRPIVNLLPLEANERITAILPVREYEEGVNVFMATASGTVKKTAL |
| SRR7299161 | WMKVYQLPEASRGARGRPIVNLLPLEANERITAILPVREYEEGVNVFMATASGTVKKTAL |
| SRR7401730 | WMKVYQLPEASRGARGRPIVNLLPLEANERITAILPVREYEEGVNVFMATASGTVKKTAL |
| SRR7469092 | WMKVYQLPEASRGARGRPIVNLLPLEANERITAILPVREYEEGVNVFMATASGTVKKTAL |
| SRR7879556 | WMKVYQLPEASRGARGRPIVNLLPLEANERITAILPVREYEEGVNVFMATASGTVKKTAL |
| SRR8526100 | WMKVYQLPEASRGARGRPIVNLLPLEANERITAILPVREYEEGVNVFMATASGTVKKTAL |
| SRR8553991 | WMKVYQLPEASRGARGRPIVNLLPLEANERITAILPVREYEEGVNVFMATASGTVKKTAL |
| SRR7842487 | WMKVYQLPEASRGARGRPIVNLLPLEANERITAILPVREYEEGVNVFMATASGTVKKTAL |
| SRR8054524 | WMKVYQLPEASRGARGRPIVNLLPLEANERITAILPVREYEEGVNVFMATASGTVKKTAL |
| SRR8054525 | WMKVYQLPEASRGARGRPIVNLLPLEANERITAILPVREYEEGVNVFMATASGTVKKTAL |
| SRR8524733 | WMKVYQLPEASRGARGRPIVNLLPLEANERITAILPVREYEEGVNVFMATASGTVKKTAL |
| SRR4093291 | WMKVYQLPEASRGARGRPIVNLLPLEANERITAILPVREYEEGVNVFMATASGTVKKTAL |
| SRR4245549 | WMKVYQLPEASRGARGRPIVNLLPLEANERITAILPVREYEEGVNVFMATASGTVKKTAL |
| SRR3057154 | WMKVYQLPEASRGARGRPIVNLLPLEANERITAILPVREYEEGVNVFMATASGTVKKTAL |
| SRR1726150 | WMKVYQLPEASRGARGRPIVNLLPLEANERITAILPVREYEEGVNVFMATASGTVKKTAL |
| SRR1996141 | WMKVYQLPEASRGARGRPIVNLLPLEANERITAILPVREYEEGVNVFMATASGTVKKTAL |
| SRR1107842 | WMKVYQLPEASRGARGRPIVNLLPLEANERITAILPVREYEEGVNVFMATASGTVKKTAL |
| SRR1157587 | WMKVYQLPEASRGARGRPIVNLLPLEANERITAILPVREYEEGVNVFMATASGTVKKTAL |
| SRR3027706 | WMKVYQLPEASRGARGRPIVNLLPLEANERITAILPVREYEEGVNVFMATASGTVKKTAL |
| SRR3027707 | WMKVYQLPEASRGARGRPIVNLLPLEANERITAILPVREYEEGVNVFMATASGTVKKTAL |
| SRR3027708 | WMKVYQLPEASRGARGRPIVNLLPLEANERITAILPVREYEEGVNVFMATASGTVKKTAL |
| SRR3027710 | WMKVYQLPEASRGARGRPIVNLLPLEANERITAILPVREYEEGVNVFMATASGTVKKTAL |
| SRR3027711 | WMKVYQLPEASRGARGRPIVNLLPLEANERITAILPVREYEEGVNVFMATASGTVKKTAL |
| SRR3027716 | WMKVYQLPEASRGARGRPIVNLLPLEANERITAILPVREYEEGVNVFMATASGTVKKTAL |
| SRR3027717 | WMKVYQLPEASRGARGRPIVNLLPLEANERITAILPVREYEEGVNVFMATASGTVKKTAL |
| SRR3027719 | WMKVYQLPEASRGARGRPIVNLLPLEANERITAILPVREYEEGVNVFMATASGTVKKTAL |
| SRR3027721 | WMKVYQLPEASRGARGRPIVNLLPLEANERITAILPVREYEEGVNVFMATASGTVKKTAL |
| SRR3027723 | WMKVYQLPEASRGARGRPIVNLLPLEANERITAILPVREYEEGVNVFMATASGTVKKTAL |
| SRR3115978 | WMKVYQLPEASRGARGRPIVNLLPLEANERITAILPVREYEEGVNVFMATASGTVKKTAL |
| SRR2534093 | WMKVYQLPEASRGARGRPIVNLLPLEANERITAILPVREYEEGVNVFMATASGTVKKTAL |
| SRR2534094 | WMKVYQLPEASRGARGRPIVNLLPLEANERITAILPVREYEEGVNVFMATASGTVKKTAL |
| SRR2534095 | WMKVYQLPEASRGARGRPIVNLLPLEANERITAILPVREYEEGVNVFMATASGTVKKTAL |
| SRR2534108 | WMKVYQLPEASRGARGRPIVNLLPLEANERITAILPVREYEEGVNVFMATASGTVKKTAL |
| SRR1106464 | WMKVYQLPEASRGARGRPIVNLLPLEANERITAILPVREYEEGVNVFMATASGTVKKTAL |
| SRR1106463 | WMKVYQLPEASRGARGRPIVNLLPLEANERITAILPVREYEEGVNVFMATASGTVKKTAL |
| SRR6949610 | WMKVYQLPEASRGARGRPIVNLLPLEANERITAILPVREYEEGVNVFMATASGTVKKTAL |
| SRR6950452 | WMKVYQLPEASRGARGRPIVNLLPLEANERITAILPVREYEEGVNVFMATASGTVKKTAL |
| ERR2019831 | WMKVYQLPEASRGARGRPIVNLLPLEANERITAILPVREYEEGVNVFMATASGTVKKTAL |
| SRR2085693 | WMKVYQLPEASRGARGRPIVNLLPLEANERITAILPVREYEEGVNVFMATASGTVKKTAL |
| SRR2086898 | WMKVYQLPEASRGARGRPIVNLLPLEANERITAILPVREYEEGVNVFMATASGTVKKTAL |
| SRR2175312 | WMKVYQLPEASRGARGRPIVNLLPLEANERITAILPVREYEEGVNVFMATASGTVKKTAL |
| SRR2175360 | WMKVYQLPEASRGARGRPIVNLLPLEANERITAILPVREYEEGVNVFMATASGTVKKTAL |
| SRR5231997 | WMKVYQLPEASRGARGRPIVNLLPLEANERITAILPVREYEEGVNVFMATASGTVKKTAL |
| SRR5232003 | WMKVYQLPEASRGARGRPIVNLLPLEANERITAILPVREYEEGVNVFMATASGTVKKTAL |
| SRR5232015 | WMKVYQLPEASRGARGRPIVNLLPLEANERITAILPVREYEEGVNVFMATASGTVKKTAL |
| SRR949434 | WMKVYQLPEASRGARGRPIVNLLPLEANERITAILPVREYEEGVNVFMATASGTVKKTAL |
| SRR3216575 | WMKVYQLPEASRGARGRPIVNLLPLEANERITAILPVREYEEGVNVFMATASGTVKKTAL |
| SRR5205342 | WMKVYQLPEASRGARGRPIVNLLPLEANERITAILPVREYEEGVNVFMATASGTVKKTAL |
| SRR1501669 | WMKVYQLPEASRGARGRPIVNLLPLEANERITAILPVREYEEGVNVFMATASGTVKKTAL |
| SRR5209740 | WMKVYQLPEASRGARGRPIVNLLPLEANERITAILPVREYEEGVNVFMATASGTVKKTAL |
| SRR3240355 | WMKVYQLPEASRGARGRPIVNLLPLEANERITAILPVREYEEGVNVFMATASGTVKKTAL |
| SRR3392777 | WMKVYQLPEASRGARGRPIVNLLPLEANERITAILPVREYEEGVNVFMATASGTVKKTAL |
| SRR3593671 | WMKVYQLPEASRGARGRPIVNLLPLEANERITAILPVREYEEGVNVFMATASGTVKKTAL |
| SRR5413290 | WMKVYQLPEASRGARGRPIVNLLPLEANERITAILPVREYEEGVNVFMATASGTVKKTAL |
| SRR5590269 | WMKVYQLPEASRGARGRPIVNLLPLEANERITAILPVREYEEGVNVFMATASGTVKKTAL |
| SRR5812103 | WMKVYQLPEASRGARGRPIVNLLPLEANERITAILPVREYEEGVNVFMATASGTVKKTAL |
| SRR2830941 | WMKVYQLPEASRGARGRPIVNLLPLEANERITAILPVREYEEGVNVFMATASGTVKKTAL |
| SRR2830966 | WMKVYQLPEASRGARGRPIVNLLPLEANERITAILPVREYEEGVNVFMATASGTVKKTAL |
| SRR3137270 | WMKVYQLPEASRGARGRPIVNLLPLEANERITAILPVREYEEGVNVFMATASGTVKKTAL |
| SRR3137271 | WMKVYQLPEASRGARGRPIVNLLPLEANERITAILPVREYEEGVNVFMATASGTVKKTAL |
| ERR526807 | WMKVYQLPEASRGARGRPIVNLLPLEANERITAILPVREYEEGVNVFMATASGTVKKTAL |
| ERR2197922 | WMKVYQLPEASRGARGRPIVNLLPLEANERITAILPVREYEEGVNVFMATASGTVKKTAL |
| ERR2197923 | WMKVYQLPEASRGARGRPIVNLLPLEANERITAILPVREYEEGVNVFMATASGTVKKTAL |
| ERR2197924 | WMKVYQLPEASRGARGRPIVNLLPLEANERITAILPVREYEEGVNVFMATASGTVKKTAL |
| ERR2197925 | WMKVYQLPEASRGARGRPIVNLLPLEANERITAILPVREYEEGVNVFMATASGTVKKTAL |
| ERR2197927 | WMKVYQLPEASRGARGRPIVNLLPLEANERITAILPVREYEEGVNVFMATASGTVKKTAL |
| ERR2197929 | WMKVYQLPEASRGARGRPIVNLLPLEANERITAILPVREYEEGVNVFMATASGTVKKTAL |
| SRR1648149 | WMKVYQLPEASRGARGRPIVNLLPLEANERITAILPVREYEEGVNVFMATASGTVKKTAL |
| SRR1048299 | WMKVYQLPEASRGARGRPIVNLLPLEANERITAILPVREYEEGVNVFMATASGTVKKTAL |
| SRR1300677 | WMKVYQLPEASRGARGRPIVNLLPLEANERITAILPVREYEEGVNVFMATASGTVKKTAL |
| SRR1288356 | WMKVYQLPEASRGARGRPIVNLLPLEANERITAILPVREYEEGVNVFMATASGTVKKTAL |
| SRR7426190 | WMKVYQLPEASRGARGRPIVNLLPLEANERITAILPVREYEEGVNVFMATASGTVKKTAL |
| SRR7426192 | WMKVYQLPEASRGARGRPIVNLLPLEANERITAILPVREYEEGVNVFMATASGTVKKTAL |
| SRR7426193 | WMKVYQLPEASRGARGRPIVNLLPLEANERITAILPVREYEEGVNVFMATASGTVKKTAL |
| SRR7441832 | WMKVYQLPEASRGARGRPIVNLLPLEANERITAILPVREYEEGVNVFMATASGTVKKTAL |
| SRR7426179 | WMKVYQLPEASRGARGRPIVNLLPLEANERITAILPVREYEEGVNVFMATASGTVKKTAL |
| SRR7439238 | WMKVYQLPEASRGARGRPIVNLLPLEANERITAILPVREYEEGVNVFMATASGTVKKTAL |
| SRR7439244 | WMKVYQLPEASRGARGRPIVNLLPLEANERITAILPVREYEEGVNVFMATASGTVKKTAL |
| SRR7439259 | WMKVYQLPEASRGARGRPIVNLLPLEANERITAILPVREYEEGVNVFMATASGTVKKTAL |
| SRR7439260 | WMKVYQLPEASRGARGRPIVNLLPLEANERITAILPVREYEEGVNVFMATASGTVKKTAL |
| SRR7441786 | WMKVYQLPEASRGARGRPIVNLLPLEANERITAILPVREYEEGVNVFMATASGTVKKTAL |
| SRR7441797 | WMKVYQLPEASRGARGRPIVNLLPLEANERITAILPVREYEEGVNVFMATASGTVKKTAL |
| ERR1759093 | WMKVYQLPEASRGARGRPIVNLLPLEANERITAILPVREYEEGVNVFMATASGTVKKTAL |
| ERR2580275 | WMKVYQLPEASRGARGRPIVNLLPLEANERITAILPVREYEEGVNVFMATASGTVKKTAL |
| ERR1759204 | WMKVYQLPEASRGARGRPIVNLLPLEANERITAILPVREYEEGVNVFMATASGTVKKTAL |
| SRR1300699 | WMKVYQLPEASRGARGRPIVNLLPLEANERITAILPVREYEEGVNVFMATASGTVKKTAL |
| S_0825_17 | WMKVYQLPEASRGARGRPIVNLLPLEANERITAILPVREYEEGVNVFMATASGTVKKTAL |
| SRR1958215 | WMKVYQLPEASRGARGRPIVNLLPLEANERITAILPVREYEEGVNVFMATASGTVKKTAL |
| SRR1958540 | WMKVYQLPEASRGARGRPIVNLLPLEANERITAILPVREYEEGVNVFMATASGTVKKTAL |
| SRR1958636 | WMKVYQLPEASRGARGRPIVNLLPLEANERITAILPVREYEEGVNVFMATASGTVKKTAL |
| SRR1959422 | WMKVYQLPEASRGARGRPIVNLLPLEANERITAILPVREYEEGVNVFMATASGTVKKTAL |
| SRR1959427 | WMKVYQLPEASRGARGRPIVNLLPLEANERITAILPVREYEEGVNVFMATASGTVKKTAL |
| SRR1960226 | WMKVYQLPEASRGARGRPIVNLLPLEANERITAILPVREYEEGVNVFMATASGTVKKTAL |
| SRR1963498 | WMKVYQLPEASRGARGRPIVNLLPLEANERITAILPVREYEEGVNVFMATASGTVKKTAL |
| SRR1965947 | WMKVYQLPEASRGARGRPIVNLLPLEANERITAILPVREYEEGVNVFMATASGTVKKTAL |
| SRR1966125 | WMKVYQLPEASRGARGRPIVNLLPLEANERITAILPVREYEEGVNVFMATASGTVKKTAL |
| SRR1966330 | WMKVYQLPEASRGARGRPIVNLLPLEANERITAILPVREYEEGVNVFMATASGTVKKTAL |
| SRR1966565 | WMKVYQLPEASRGARGRPIVNLLPLEANERITAILPVREYEEGVNVFMATASGTVKKTAL |
| SRR1966864 | WMKVYQLPEASRGARGRPIVNLLPLEANERITAILPVREYEEGVNVFMATASGTVKKTAL |
| SRR1966989 | WMKVYQLPEASRGARGRPIVNLLPLEANERITAILPVREYEEGVNVFMATASGTVKKTAL |
| SRR1967688 | WMKVYQLPEASRGARGRPIVNLLPLEANERITAILPVREYEEGVNVFMATASGTVKKTAL |
| SRR1967733 | WMKVYQLPEASRGARGRPIVNLLPLEANERITAILPVREYEEGVNVFMATASGTVKKTAL |
| SRR1967746 | WMKVYQLPEASRGARGRPIVNLLPLEANERITAILPVREYEEGVNVFMATASGTVKKTAL |
| SRR1968341 | WMKVYQLPEASRGARGRPIVNLLPLEANERITAILPVREYEEGVNVFMATASGTVKKTAL |
| SRR1968456 | WMKVYQLPEASRGARGRPIVNLLPLEANERITAILPVREYEEGVNVFMATASGTVKKTAL |
| SRR1968465 | WMKVYQLPEASRGARGRPIVNLLPLEANERITAILPVREYEEGVNVFMATASGTVKKTAL |
| SRR1968761 | WMKVYQLPEASRGARGRPIVNLLPLEANERITAILPVREYEEGVNVFMATASGTVKKTAL |
| SRR1969047 | WMKVYQLPEASRGARGRPIVNLLPLEANERITAILPVREYEEGVNVFMATASGTVKKTAL |
| SRR1969255 | WMKVYQLPEASRGARGRPIVNLLPLEANERITAILPVREYEEGVNVFMATASGTVKKTAL |
| SRR1969412 | WMKVYQLPEASRGARGRPIVNLLPLEANERITAILPVREYEEGVNVFMATASGTVKKTAL |
| SRR1969524 | WMKVYQLPEASRGARGRPIVNLLPLEANERITAILPVREYEEGVNVFMATASGTVKKTAL |
| SRR1969584 | WMKVYQLPEASRGARGRPIVNLLPLEANERITAILPVREYEEGVNVFMATASGTVKKTAL |
| SRR1969648 | WMKVYQLPEASRGARGRPIVNLLPLEANERITAILPVREYEEGVNVFMATASGTVKKTAL |
| SRR1969804 | WMKVYQLPEASRGARGRPIVNLLPLEANERITAILPVREYEEGVNVFMATASGTVKKTAL |
| SRR1970221 | WMKVYQLPEASRGARGRPIVNLLPLEANERITAILPVREYEEGVNVFMATASGTVKKTAL |
| SRR1970268 | WMKVYQLPEASRGARGRPIVNLLPLEANERITAILPVREYEEGVNVFMATASGTVKKTAL |
| SRR1965862 | WMKVYQLPEASRGARGRPIVNLLPLEANERITAILPVREYEEGVNVFMATASGTVKKTAL |
| SRR1967363 | WMKVYQLPEASRGARGRPIVNLLPLEANERITAILPVREYEEGVNVFMATASGTVKKTAL |
| SRR1968276 | WMKVYQLPEASRGARGRPIVNLLPLEANERITAILPVREYEEGVNVFMATASGTVKKTAL |
| SRR1968967 | WMKVYQLPEASRGARGRPIVNLLPLEANERITAILPVREYEEGVNVFMATASGTVKKTAL |
| SRR3321531 | WMKVYQLPEASRGARGRPIVNLLPLEANERITAILPVREYEEGVNVFMATASGTVKKTAL |
| SRR3321883 | WMKVYQLPEASRGARGRPIVNLLPLEANERITAILPVREYEEGVNVFMATASGTVKKTAL |
| SRR3322413 | WMKVYQLPEASRGARGRPIVNLLPLEANERITAILPVREYEEGVNVFMATASGTVKKTAL |
| SRR3323012 | WMKVYQLPEASRGARGRPIVNLLPLEANERITAILPVREYEEGVNVFMATASGTVKKTAL |
| SRR5194289 | WMKVYQLPEASRGARGRPIVNLLPLEANERITAILPVREYEEGVNVFMATASGTVKKTAL |
| SRR7163798 | WMKVYQLPEASRGARGRPIVNLLPLEANERITAILPVREYEEGVNVFMATASGTVKKTAL |
| SRR7172610 | WMKVYQLPEASRGARGRPIVNLLPLEANERITAILPVREYEEGVNVFMATASGTVKKTAL |
| SRR7204568 | WMKVYQLPEASRGARGRPIVNLLPLEANERITAILPVREYEEGVNVFMATASGTVKKTAL |
| SRR7223230 | WMKVYQLPEASRGARGRPIVNLLPLEANERITAILPVREYEEGVNVFMATASGTVKKTAL |
| SRR7230675 | WMKVYQLPEASRGARGRPIVNLLPLEANERITAILPVREYEEGVNVFMATASGTVKKTAL |
| SRR7278056 | WMKVYQLPEASRGARGRPIVNLLPLEANERITAILPVREYEEGVNVFMATASGTVKKTAL |
| SRR7278086 | WMKVYQLPEASRGARGRPIVNLLPLEANERITAILPVREYEEGVNVFMATASGTVKKTAL |
| SRR7285841 | WMKVYQLPEASRGARGRPIVNLLPLEANERITAILPVREYEEGVNVFMATASGTVKKTAL |
| SRR7292625 | WMKVYQLPEASRGARGRPIVNLLPLEANERITAILPVREYEEGVNVFMATASGTVKKTAL |
| SRR7292665 | WMKVYQLPEASRGARGRPIVNLLPLEANERITAILPVREYEEGVNVFMATASGTVKKTAL |
| SRR7297965 | WMKVYQLPEASRGARGRPIVNLLPLEANERITAILPVREYEEGVNVFMATASGTVKKTAL |
| SRR7350726 | WMKVYQLPEASRGARGRPIVNLLPLEANERITAILPVREYEEGVNVFMATASGTVKKTAL |
| SRR7410328 | WMKVYQLPEASRGARGRPIVNLLPLEANERITAILPVREYEEGVNVFMATASGTVKKTAL |
| SRR7474665 | WMKVYQLPEASRGARGRPIVNLLPLEANERITAILPVREYEEGVNVFMATASGTVKKTAL |
| SRR7523184 | WMKVYQLPEASRGARGRPIVNLLPLEANERITAILPVREYEEGVNVFMATASGTVKKTAL |
| SRR7187264 | WMKVYQLPEASRGARGRPIVNLLPLEANERITAILPVREYEEGVNVFMATASGTVKKTAL |
| SRR7204445 | WMKVYQLPEASRGARGRPIVNLLPLEANERITAILPVREYEEGVNVFMATASGTVKKTAL |
| SRR7285641 | WMKVYQLPEASRGARGRPIVNLLPLEANERITAILPVREYEEGVNVFMATASGTVKKTAL |
| SRR7286695 | WMKVYQLPEASRGARGRPIVNLLPLEANERITAILPVREYEEGVNVFMATASGTVKKTAL |
| SRR7286705 | WMKVYQLPEASRGARGRPIVNLLPLEANERITAILPVREYEEGVNVFMATASGTVKKTAL |
| SRR7292931 | WMKVYQLPEASRGARGRPIVNLLPLEANERITAILPVREYEEGVNVFMATASGTVKKTAL |
| SRR7310349 | WMKVYQLPEASRGARGRPIVNLLPLEANERITAILPVREYEEGVNVFMATASGTVKKTAL |
| SRR7351616 | WMKVYQLPEASRGARGRPIVNLLPLEANERITAILPVREYEEGVNVFMATASGTVKKTAL |
| SRR7414818 | WMKVYQLPEASRGARGRPIVNLLPLEANERITAILPVREYEEGVNVFMATASGTVKKTAL |
| SRR7426480 | WMKVYQLPEASRGARGRPIVNLLPLEANERITAILPVREYEEGVNVFMATASGTVKKTAL |
| SRR5584105 | WMKVYQLPEASRGARGRPIVNLLPLEANERITAILPVREYEEGVNVFMATASGTVKKTAL |
| SRR5584565 | WMKVYQLPEASRGARGRPIVNLLPLEANERITAILPVREYEEGVNVFMATASGTVKKTAL |
| SRR5584614 | WMKVYQLPEASRGARGRPIVNLLPLEANERITAILPVREYEEGVNVFMATASGTVKKTAL |
| SRR5631543 | WMKVYQLPEASRGARGRPIVNLLPLEANERITAILPVREYEEGVNVFMATASGTVKKTAL |
| SRR5631553 | WMKVYQLPEASRGARGRPIVNLLPLEANERITAILPVREYEEGVNVFMATASGTVKKTAL |
| SRR7123196 | WMKVYQLPEASRGARGRPIVNLLPLEANERITAILPVREYEEGVNVFMATASGTVKKTAL |
| SRR7163819 | WMKVYQLPEASRGARGRPIVNLLPLEANERITAILPVREYEEGVNVFMATASGTVKKTAL |
| SRR7163920 | WMKVYQLPEASRGARGRPIVNLLPLEANERITAILPVREYEEGVNVFMATASGTVKKTAL |
| SRR7209528 | WMKVYQLPEASRGARGRPIVNLLPLEANERITAILPVREYEEGVNVFMATASGTVKKTAL |
| SRR7249868 | WMKVYQLPEASRGARGRPIVNLLPLEANERITAILPVREYEEGVNVFMATASGTVKKTAL |
| SRR7278088 | WMKVYQLPEASRGARGRPIVNLLPLEANERITAILPVREYEEGVNVFMATASGTVKKTAL |
| SRR7285788 | WMKVYQLPEASRGARGRPIVNLLPLEANERITAILPVREYEEGVNVFMATASGTVKKTAL |
| SRR7286789 | WMKVYQLPEASRGARGRPIVNLLPLEANERITAILPVREYEEGVNVFMATASGTVKKTAL |
| SRR7286886 | WMKVYQLPEASRGARGRPIVNLLPLEANERITAILPVREYEEGVNVFMATASGTVKKTAL |
| SRR7310632 | WMKVYQLPEASRGARGRPIVNLLPLEANERITAILPVREYEEGVNVFMATASGTVKKTAL |
| SRR7350631 | WMKVYQLPEASRGARGRPIVNLLPLEANERITAILPVREYEEGVNVFMATASGTVKKTAL |
| SRR7458741 | WMKVYQLPEASRGARGRPIVNLLPLEANERITAILPVREYEEGVNVFMATASGTVKKTAL |
| SRR7480280 | WMKVYQLPEASRGARGRPIVNLLPLEANERITAILPVREYEEGVNVFMATASGTVKKTAL |
| SRR7523660 | WMKVYQLPEASRGARGRPIVNLLPLEANERITAILPVREYEEGVNVFMATASGTVKKTAL |
| SRR7523775 | WMKVYQLPEASRGARGRPIVNLLPLEANERITAILPVREYEEGVNVFMATASGTVKKTAL |
| SRR7251101 | WMKVYQLPEASRGARGRPIVNLLPLEANERITAILPVREYEEGVNVFMATASGTVKKTAL |
| SRR7284299 | WMKVYQLPEASRGARGRPIVNLLPLEANERITAILPVREYEEGVNVFMATASGTVKKTAL |
| SRR7285738 | WMKVYQLPEASRGARGRPIVNLLPLEANERITAILPVREYEEGVNVFMATASGTVKKTAL |
| SRR7310640 | WMKVYQLPEASRGARGRPIVNLLPLEANERITAILPVREYEEGVNVFMATASGTVKKTAL |
| SRR7349159 | WMKVYQLPEASRGARGRPIVNLLPLEANERITAILPVREYEEGVNVFMATASGTVKKTAL |
| SRR7474873 | WMKVYQLPEASRGARGRPIVNLLPLEANERITAILPVREYEEGVNVFMATASGTVKKTAL |
| SRR7495689 | WMKVYQLPEASRGARGRPIVNLLPLEANERITAILPVREYEEGVNVFMATASGTVKKTAL |
| SRR7495752 | WMKVYQLPEASRGARGRPIVNLLPLEANERITAILPVREYEEGVNVFMATASGTVKKTAL |
| ----------------------------------------------------------------------------- | |
| S16BD08730 | TEFSRPRSAGIIAVNLNDGDELIGVDLTSGSDEVMLFSAAGKVVRFKEDAVRAMGRTATG |
| S18BD00684 | TEFSRPRSAGIIAVNLNDGDELIGVDLTSGSDEVMLFSAAGKVVRFKEDAVRAMGRTATG |
| S18BD03994 | TEFSRPRSAGIIAVNLNDGDELIGVDLTSGSDEVMLFSAAGKVVRFKEDAVRAMGRTATG |
| S18BD05011 | TEFSRPRSAGIIAVNLNDGDELIGVDLTSGSDEVMLFSAAGKVVRFKEDAVRAMGRTATG |
| RKI_16-03723 | TEFSRPRSAGIIAVNLNDGDELIGVDLTSGSDEVMLFSAAGKVVRFKEDAVRAMGRTATG |
| RKI_16-04315 | TEFSRPRSAGIIAVNLNDGDELIGVDLTSGSDEVMLFSAAGKVVRFKEDAVRAMGRTATG |
| RKI_17-02304 | TEFSRPRSAGIIAVNLNDGDELIGVDLTSGSDEVMLFSAAGKVVRFKEDAVRAMGRTATG |
| RKI_17-02411 | TEFSRPRSAGIIAVNLNDGDELIGVDLTSGSDEVMLFSAAGKVVRFKEDAVRAMGRTATG |
| RKI_17-02757 | TEFSRPRSAGIIAVNLNDGDELIGVDLTSGSDEVMLFSAAGKVVRFKEDAVRAMGRTATG |
| RKI_17-04797 | TEFSRPRSAGIIAVNLNDGDELIGVDLTSGSDEVMLFSAAGKVVRFKEDAVRAMGRTATG |
| RKI_17-06869 | TEFSRPRSAGIIAVNLNDGDELIGVDLTSGSDEVMLFSAAGKVVRFKEDAVRAMGRTATG |
| ERR2580277 | TEFSRPRSAGIIAVNLNDGDELIGVDLTSGSDEVMLFSAAGKVVRFKEDAVRAMGRTATG |
| ERR2580276 | TEFSRPRSAGIIAVNLNDGDELIGVDLTSGSDEVMLFSAAGKVVRFKEDAVRAMGRTATG |
| ERR2580273 | TEFSRPRSAGIIAVNLNDGDELIGVDLTSGSDEVMLFSAAGKVVRFKEDAVRAMGRTATG |
| ERR2580274 | TEFSRPRSAGIIAVNLNDGDELIGVDLTSGSDEVMLFSAAGKVVRFKEDAVRAMGRTATG |
| ERR2173656 | TEFSRPRSAGIIAVNLNDGDELIGVDLTSGSDEVMLFSAAGKVVRFKEDAVRAMGRTATG |
| 17041676 | TEFSRPRSAGIIAVNLNDGDELIGVDLTSGSDEVMLFSAAGKVVRFKEDAVRAMGRTATG |
| MT16-000061 | TEFSRPRSAGIIAVNLNDGDELIGVDLTSGSDEVMLFSAAGKVVRFKEDAVRAMGRTATG |
| MT16-019416 | TEFSRPRSAGIIAVNLNDGDELIGVDLTSGSDEVMLFSAAGKVVRFKEDAVRAMGRTATG |
| MT16-027865 | TEFSRPRSAGIIAVNLNDGDELIGVDLTSGSDEVMLFSAAGKVVRFKEDAVRAMGRTATG |
| MT16-031693 | TEFSRPRSAGIIAVNLNDGDELIGVDLTSGSDEVMLFSAAGKVVRFKEDAVRAMGRTATG |
| MT16-040253 | TEFSRPRSAGIIAVNLNDGDELIGVDLTSGSDEVMLFSAAGKVVRFKEDAVRAMGRTATG |
| MT16-045379 | TEFSRPRSAGIIAVNLNDGDELIGVDLTSGSDEVMLFSAAGKVVRFKEDAVRAMGRTATG |
| MT16-442728 | TEFSRPRSAGIIAVNLNDGDELIGVDLTSGSDEVMLFSAAGKVVRFKEDAVRAMGRTATG |
| MT16-462857 | TEFSRPRSAGIIAVNLNDGDELIGVDLTSGSDEVMLFSAAGKVVRFKEDAVRAMGRTATG |
| MT16-480196 | TEFSRPRSAGIIAVNLNDGDELIGVDLTSGSDEVMLFSAAGKVVRFKEDAVRAMGRTATG |
| MT16-861555 | TEFSRPRSAGIIAVNLNDGDELIGVDLTSGSDEVMLFSAAGKVVRFKEDAVRAMGRTATG |
| MT17-076833 | TEFSRPRSAGIIAVNLNDGDELIGVDLTSGSDEVMLFSAAGKVVRFKEDAVRAMGRTATG |
| MT17-110677 | TEFSRPRSAGIIAVNLNDGDELIGVDLTSGSDEVMLFSAAGKVVRFKEDAVRAMGRTATG |
| MT17-131730 | TEFSRPRSAGIIAVNLNDGDELIGVDLTSGSDEVMLFSAAGKVVRFKEDAVRAMGRTATG |
| MT17-140890 | TEFSRPRSAGIIAVNLNDGDELIGVDLTSGSDEVMLFSAAGKVVRFKEDAVRAMGRTATG |
| MT17-141840 | TEFSRPRSAGIIAVNLNDGDELIGVDLTSGSDEVMLFSAAGKVVRFKEDAVRAMGRTATG |
| MT17-152488 | TEFSRPRSAGIIAVNLNDGDELIGVDLTSGSDEVMLFSAAGKVVRFKEDAVRAMGRTATG |
| MT17-157311 | TEFSRPRSAGIIAVNLNDGDELIGVDLTSGSDEVMLFSAAGKVVRFKEDAVRAMGRTATG |
| MT17-161645 | TEFSRPRSAGIIAVNLNDGDELIGVDLTSGSDEVMLFSAAGKVVRFKEDAVRAMGRTATG |
| MT17-167951 | TEFSRPRSAGIIAVNLNDGDELIGVDLTSGSDEVMLFSAAGKVVRFKEDAVRAMGRTATG |
| MT18-217732 | TEFSRPRSAGIIAVNLNDGDELIGVDLTSGSDEVMLFSAAGKVVRFKEDAVRAMGRTATG |
| MT18-252580 | TEFSRPRSAGIIAVNLNDGDELIGVDLTSGSDEVMLFSAAGKVVRFKEDAVRAMGRTATG |
| RIVM_H_2009-01 | TEFSRPRSAGIIAVNLNDGDELIGVDLTSGSDEVMLFSAAGKVVRFKEDAVRAMGRTATG |
| RIVM_H_2010-01 | TEFSRPRSAGIIAVNLNDGDELIGVDLTSGSDEVMLFSAAGKVVRFKEDAVRAMGRTATG |
| RIVM_H_2010-02 | TEFSRPRSAGIIAVNLNDGDELIGVDLTSGSDEVMLFSAAGKVVRFKEDAVRAMGRTATG |
| RIVM_H_2011-01 | TEFSRPRSAGIIAVNLNDGDELIGVDLTSGSDEVMLFSAAGKVVRFKEDAVRAMGRTATG |
| RIVM_H_2011-02 | TEFSRPRSAGIIAVNLNDGDELIGVDLTSGSDEVMLFSAAGKVVRFKEDAVRAMGRTATG |
| RIVM_H_2011-03 | TEFSRPRSAGIIAVNLNDGDELIGVDLTSGSDEVMLFSAAGKVVRFKEDAVRAMGRTATG |
| RIVM_H_2013-01 | TEFSRPRSAGIIAVNLNDGDELIGVDLTSGSDEVMLFSAAGKVVRFKEDAVRAMGRTATG |
| RIVM_H_2013-02 | TEFSRPRSAGIIAVNLNDGDELIGVDLTSGSDEVMLFSAAGKVVRFKEDAVRAMGRTATG |
| RIVM_H_2014-01 | TEFSRPRSAGIIAVNLNDGDELIGVDLTSGSDEVMLFSAAGKVVRFKEDAVRAMGRTATG |
| RIVM_H_2014-02 | TEFSRPRSAGIIAVNLNDGDELIGVDLTSGSDEVMLFSAAGKVVRFKEDAVRAMGRTATG |
| RIVM_H_2016-01 | TEFSRPRSAGIIAVNLNDGDELIGVDLTSGSDEVMLFSAAGKVVRFKEDAVRAMGRTATG |
| RIVM_H_2016-02 | TEFSRPRSAGIIAVNLNDGDELIGVDLTSGSDEVMLFSAAGKVVRFKEDAVRAMGRTATG |
| RIVM_H_2016-03 | TEFSRPRSAGIIAVNLNDGDELIGVDLTSGSDEVMLFSAAGKVVRFKEDAVRAMGRTATG |
| RIVM_H_2016-04 | TEFSRPRSAGIIAVNLNDGDELIGVDLTSGSDEVMLFSAAGKVVRFKEDAVRAMGRTATG |
| RIVM_H_2016-05 | TEFSRPRSAGIIAVNLNDGDELIGVDLTSGSDEVMLFSAAGKVVRFKEDAVRAMGRTATG |
| RIVM_H_2016-06 | TEFSRPRSAGIIAVNLNDGDELIGVDLTSGSDEVMLFSAAGKVVRFKEDAVRAMGRTATG |
| RIVM_H_2016-07 | TEFSRPRSAGIIAVNLNDGDELIGVDLTSGSDEVMLFSAAGKVVRFKEDAVRAMGRTATG |
| RIVM_H_2016-08 | TEFSRPRSAGIIAVNLNDGDELIGVDLTSGSDEVMLFSAAGKVVRFKEDAVRAMGRTATG |
| RIVM_H_2016-09 | TEFSRPRSAGIIAVNLNDGDELIGVDLTSGSDEVMLFSAAGKVVRFKEDAVRAMGRTATG |
| RIVM_H_2016-10 | TEFSRPRSAGIIAVNLNDGDELIGVDLTSGSDEVMLFSAAGKVVRFKEDAVRAMGRTATG |
| RIVM_H_2016-11 | TEFSRPRSAGIIAVNLNDGDELIGVDLTSGSDEVMLFSAAGKVVRFKEDAVRAMGRTATG |
| RIVM_H_2016-12 | TEFSRPRSAGIIAVNLNDGDELIGVDLTSGSDEVMLFSAAGKVVRFKEDAVRAMGRTATG |
| RIVM_H_2016-13 | TEFSRPRSAGIIAVNLNDGDELIGVDLTSGSDEVMLFSAAGKVVRFKEDAVRAMGRTATG |
| RIVM_H_2016-14 | TEFSRPRSAGIIAVNLNDGDELIGVDLTSGSDEVMLFSAAGKVVRFKEDAVRAMGRTATG |
| RIVM_H_2016-15 | TEFSRPRSAGIIAVNLNDGDELIGVDLTSGSDEVMLFSAAGKVVRFKEDAVRAMGRTATG |
| RIVM_H_2017-01 | TEFSRPRSAGIIAVNLNDGDELIGVDLTSGSDEVMLFSAAGKVVRFKEDAVRAMGRTATG |
| RIVM_H_2017-02 | TEFSRPRSAGIIAVNLNDGDELIGVDLTSGSDEVMLFSAAGKVVRFKEDAVRAMGRTATG |
| RIVM_H_2017-03 | TEFSRPRSAGIIAVNLNDGDELIGVDLTSGSDEVMLFSAAGKVVRFKEDAVRAMGRTATG |
| RIVM_H_2017-04 | TEFSRPRSAGIIAVNLNDGDELIGVDLTSGSDEVMLFSAAGKVVRFKEDAVRAMGRTATG |
| RIVM_H_2017-05 | TEFSRPRSAGIIAVNLNDGDELIGVDLTSGSDEVMLFSAAGKVVRFKEDAVRAMGRTATG |
| RIVM_H_2017-06 | TEFSRPRSAGIIAVNLNDGDELIGVDLTSGSDEVMLFSAAGKVVRFKEDAVRAMGRTATG |
| RIVM_H_2017-07 | TEFSRPRSAGIIAVNLNDGDELIGVDLTSGSDEVMLFSAAGKVVRFKEDAVRAMGRTATG |
| RIVM_H_2017-08 | TEFSRPRSAGIIAVNLNDGDELIGVDLTSGSDEVMLFSAAGKVVRFKEDAVRAMGRTATG |
| RIVM_H_2017-09 | TEFSRPRSAGIIAVNLNDGDELIGVDLTSGSDEVMLFSAAGKVVRFKEDAVRAMGRTATG |
| RIVM_H_2017-10 | TEFSRPRSAGIIAVNLNDGDELIGVDLTSGSDEVMLFSAAGKVVRFKEDAVRAMGRTATG |
| RIVM_H_2017-11 | TEFSRPRSAGIIAVNLNDGDELIGVDLTSGSDEVMLFSAAGKVVRFKEDAVRAMGRTATG |
| RIVM_H_2017-12 | TEFSRPRSAGIIAVNLNDGDELIGVDLTSGSDEVMLFSAAGKVVRFKEDAVRAMGRTATG |
| RIVM_H_2017-13 | TEFSRPRSAGIIAVNLNDGDELIGVDLTSGSDEVMLFSAAGKVVRFKEDAVRAMGRTATG |
| RIVM_H_2017-14 | TEFSRPRSAGIIAVNLNDGDELIGVDLTSGSDEVMLFSAAGKVVRFKEDAVRAMGRTATG |
| RIVM_H_2017-15 | TEFSRPRSAGIIAVNLNDGDELIGVDLTSGSDEVMLFSAAGKVVRFKEDAVRAMGRTATG |
| RIVM_H_2017-16 | TEFSRPRSAGIIAVNLNDGDELIGVDLTSGSDEVMLFSAAGKVVRFKEDAVRAMGRTATG |
| RIVM_H_2017-17 | TEFSRPRSAGIIAVNLNDGDELIGVDLTSGSDEVMLFSAAGKVVRFKEDAVRAMGRTATG |
| RIVM_H_2017-18 | TEFSRPRSAGIIAVNLNDGDELIGVDLTSGSDEVMLFSAAGKVVRFKEDAVRAMGRTATG |
| RIVM_H_2017-19 | TEFSRPRSAGIIAVNLNDGDELIGVDLTSGSDEVMLFSAAGKVVRFKEDAVRAMGRTATG |
| 15EP001483 | TEFSRPRSAGIIAVNLNDGDELIGVDLTSGSDEVMLFSAAGKVVRFKEDAVRAMGRTATG |
| 17EP002363 | TEFSRPRSAGIIAVNLNDGDELIGVDLTSGSDEVMLFSAAGKVVRFKEDAVRAMGRTATG |
| S_0812_17 | TEFSRPRSAGIIAVNLNDGDELIGVDLTSGSDEVMLFSAAGKVVRFKEDAVRAMGRTATG |
| SRR1957844 | TEFSRPRSAGIIAVNLNDGDELIGVDLTSGSDEVMLFSAAGKVVRFKEDAVRAMGRTATG |
| SRR1958654 | TEFSRPRSAGIIAVNLNDGDELIGVDLTSGSDEVMLFSAAGKVVRFKEDAVRAMGRTATG |
| SRR1965077 | TEFSRPRSAGIIAVNLNDGDELIGVDLTSGSDEVMLFSAAGKVVRFKEDAVRAMGRTATG |
| SRR1966369 | TEFSRPRSAGIIAVNLNDGDELIGVDLTSGSDEVMLFSAAGKVVRFKEDAVRAMGRTATG |
| SRR1967117 | TEFSRPRSAGIIAVNLNDGDELIGVDLTSGSDEVMLFSAAGKVVRFKEDAVRAMGRTATG |
| SRR1967922 | TEFSRPRSAGIIAVNLNDGDELIGVDLTSGSDEVMLFSAAGKVVRFKEDAVRAMGRTATG |
| SRR8704720 | TEFSRPRSAGIIAVNLNDGDELIGVDLTSGSDEVMLFSAAGKVVRFKEDAVRAMGRTATG |
| SRR7216071 | TEFSRPRSAGIIAVNLNDGDELIGVDLTSGSDEVMLFSAAGKVVRFKEDAVRAMGRTATG |
| SRR7349175 | TEFSRPRSAGIIAVNLNDGDELIGVDLTSGSDEVMLFSAAGKVVRFKEDAVRAMGRTATG |
| SRR7523148 | TEFSRPRSAGIIAVNLNDGDELIGVDLTSGSDEVMLFSAAGKVVRFKEDAVRAMGRTATG |
| SRR7523854 | TEFSRPRSAGIIAVNLNDGDELIGVDLTSGSDEVMLFSAAGKVVRFKEDAVRAMGRTATG |
| 313865 | TEFSRPRSAGIIAVNLNDGDELIGVDLTSGSDEVMLFSAAGKVVRFKEDAVRAMGRTATG |
| SRR7277793 | TEFSRPRSAGIIAVNLNDGDELIGVDLTSGSDEVMLFSAAGKVVRFKEDAVRAMGRTATG |
| SRR7343877 | TEFSRPRSAGIIAVNLNDGDELIGVDLTSGSDEVMLFSAAGKVVRFKEDAVRAMGRTATG |
| SRR7351477 | TEFSRPRSAGIIAVNLNDGDELIGVDLTSGSDEVMLFSAAGKVVRFKEDAVRAMGRTATG |
| SRR5583183 | TEFSRPRSAGIIAVNLNDGDELIGVDLTSGSDEVMLFSAAGKVVRFKEDAVRAMGRTATG |
| SRR5585240 | TEFSRPRSAGIIAVNLNDGDELIGVDLTSGSDEVMLFSAAGKVVRFKEDAVRAMGRTATG |
| SRR7284317 | TEFSRPRSAGIIAVNLNDGDELIGVDLTSGSDEVMLFSAAGKVVRFKEDAVRAMGRTATG |
| SRR7299161 | TEFSRPRSAGIIAVNLNDGDELIGVDLTSGSDEVMLFSAAGKVVRFKEDAVRAMGRTATG |
| SRR7401730 | TEFSRPRSAGIIAVNLNDGDELIGVDLTSGSDEVMLFSAAGKVVRFKEDAVRAMGRTATG |
| SRR7469092 | TEFSRPRSAGIIAVNLNDGDELIGVDLTSGSDEVMLFSAAGKVVRFKEDAVRAMGRTATG |
| SRR7879556 | TEFSRPRSAGIIAVNLNDGDELIGVDLTSGSDEVMLFSAAGKVVRFKEDAVRAMGRTATG |
| SRR8526100 | TEFSRPRSAGIIAVNLNDGDELIGVDLTSGSDEVMLFSAAGKVVRFKEDAVRAMGRTATG |
| SRR8553991 | TEFSRPRSAGIIAVNLNDGDELIGVDLTSGSDEVMLFSAAGKVVRFKEDAVRAMGRTATG |
| SRR7842487 | TEFSRPRSAGIIAVNLNDGDELIGVDLTSGSDEVMLFSAAGKVVRFKEDAVRAMGRTATG |
| SRR8054524 | TEFSRPRSAGIIAVNLNDGDELIGVDLTSGSDEVMLFSAAGKVVRFKEDAVRAMGRTATG |
| SRR8054525 | TEFSRPRSAGIIAVNLNDGDELIGVDLTSGSDEVMLFSAAGKVVRFKEDAVRAMGRTATG |
| SRR8524733 | TEFSRPRSAGIIAVNLNDGDELIGVDLTSGSDEVMLFSAAGKVVRFKEDAVRAMGRTATG |
| SRR4093291 | TEFSRPRSAGIIAVNLNDGDELIGVDLTSGSDEVMLFSAAGKVVRFKEDAVRAMGRTATG |
| SRR4245549 | TEFSRPRSAGIIAVNLNDGDELIGVDLTSGSDEVMLFSAAGKVVRFKEDAVRAMGRTATG |
| SRR3057154 | TEFSRPRSAGIIAVNLNDGDELIGVDLTSGSDEVMLFSAAGKVVRFKEDAVRAMGRTATG |
| SRR1726150 | TEFSRPRSAGIIAVNLNDGDELIGVDLTSGSDEVMLFSAAGKVVRFKEDAVRAMGRTATG |
| SRR1996141 | TEFSRPRSAGIIAVNLNDGDELIGVDLTSGSDEVMLFSAAGKVVRFKEDAVRAMGRTATG |
| SRR1107842 | TEFSRPRSAGIIAVNLNDGDELIGVDLTSGSDEVMLFSAAGKVVRFKEDAVRAMGRTATG |
| SRR1157587 | TEFSRPRSAGIIAVNLNDGDELIGVDLTSGSDEVMLFSAAGKVVRFKEDAVRAMGRTATG |
| SRR3027706 | TEFSRPRSAGIIAVNLNDGDELIGVDLTSGSDEVMLFSAAGKVVRFKEDAVRAMGRTATG |
| SRR3027707 | TEFSRPRSAGIIAVNLNDGDELIGVDLTSGSDEVMLFSAAGKVVRFKEDAVRAMGRTATG |
| SRR3027708 | TEFSRPRSAGIIAVNLNDGDELIGVDLTSGSDEVMLFSAAGKVVRFKEDAVRAMGRTATG |
| SRR3027710 | TEFSRPRSAGIIAVNLNDGDELIGVDLTSGSDEVMLFSAAGKVVRFKEDAVRAMGRTATG |
| SRR3027711 | TEFSRPRSAGIIAVNLNDGDELIGVDLTSGSDEVMLFSAAGKVVRFKEDAVRAMGRTATG |
| SRR3027716 | TEFSRPRSAGIIAVNLNDGDELIGVDLTSGSDEVMLFSAAGKVVRFKEDAVRAMGRTATG |
| SRR3027717 | TEFSRPRSAGIIAVNLNDGDELIGVDLTSGSDEVMLFSAAGKVVRFKEDAVRAMGRTATG |
| SRR3027719 | TEFSRPRSAGIIAVNLNDGDELIGVDLTSGSDEVMLFSAAGKVVRFKEDAVRAMGRTATG |
| SRR3027721 | TEFSRPRSAGIIAVNLNDGDELIGVDLTSGSDEVMLFSAAGKVVRFKEDAVRAMGRTATG |
| SRR3027723 | TEFSRPRSAGIIAVNLNDGDELIGVDLTSGSDEVMLFSAAGKVVRFKEDAVRAMGRTATG |
| SRR3115978 | TEFSRPRSAGIIAVNLNDGDELIGVDLTSGSDEVMLFSAAGKVVRFKEDAVRAMGRTATG |
| SRR2534093 | TEFSRPRSAGIIAVNLNDGDELIGVDLTSGSDEVMLFSAAGKVVRFKEDAVRAMGRTATG |
| SRR2534094 | TEFSRPRSAGIIAVNLNDGDELIGVDLTSGSDEVMLFSAAGKVVRFKEDAVRAMGRTATG |
| SRR2534095 | TEFSRPRSAGIIAVNLNDGDELIGVDLTSGSDEVMLFSAAGKVVRFKEDAVRAMGRTATG |
| SRR2534108 | TEFSRPRSAGIIAVNLNDGDELIGVDLTSGSDEVMLFSAAGKVVRFKEDAVRAMGRTATG |
| SRR1106464 | TEFSRPRSAGIIAVNLNDGDELIGVDLTSGSDEVMLFSAAGKVVRFKEDAVRAMGRTATG |
| SRR1106463 | TEFSRPRSAGIIAVNLNDGDELIGVDLTSGSDEVMLFSAAGKVVRFKEDAVRAMGRTATG |
| SRR6949610 | TEFSRPRSAGIIAVNLNDGDELIGVDLTSGSDEVMLFSAAGKVVRFKEDAVRAMGRTATG |
| SRR6950452 | TEFSRPRSAGIIAVNLNDGDELIGVDLTSGSDEVMLFSAAGKVVRFKEDAVRAMGRTATG |
| ERR2019831 | TEFSRPRSAGIIAVNLNDGDELIGVDLTSGSDEVMLFSAAGKVVRFKEDAVRAMGRTATG |
| SRR2085693 | TEFSRPRSAGIIAVNLNDGDELIGVDLTSGSDEVMLFSAAGKVVRFKEDAVRAMGRTATG |
| SRR2086898 | TEFSRPRSAGIIAVNLNDGDELIGVDLTSGSDEVMLFSAAGKVVRFKEDAVRAMGRTATG |
| SRR2175312 | TEFSRPRSAGIIAVNLNDGDELIGVDLTSGSDEVMLFSAAGKVVRFKEDAVRAMGRTATG |
| SRR2175360 | TEFSRPRSAGIIAVNLNDGDELIGVDLTSGSDEVMLFSAAGKVVRFKEDAVRAMGRTATG |
| SRR5231997 | TEFSRPRSAGIIAVNLNDGDELIGVDLTSGSDEVMLFSAAGKVVRFKEDAVRAMGRTATG |
| SRR5232003 | TEFSRPRSAGIIAVNLNDGDELIGVDLTSGSDEVMLFSAAGKVVRFKEDAVRAMGRTATG |
| SRR5232015 | TEFSRPRSAGIIAVNLNDGDELIGVDLTSGSDEVMLFSAAGKVVRFKEDAVRAMGRTATG |
| SRR949434 | TEFSRPRSAGIIAVNLNDGDELIGVDLTSGSDEVMLFSAAGKVVRFKEDAVRAMGRTATG |
| SRR3216575 | TEFSRPRSAGIIAVNLNDGDELIGVDLTSGSDEVMLFSAAGKVVRFKEDAVRAMGRTATG |
| SRR5205342 | TEFSRPRSAGIIAVNLNDGDELIGVDLTSGSDEVMLFSAAGKVVRFKEDAVRAMGRTATG |
| SRR1501669 | TEFSRPRSAGIIAVNLNDGDELIGVDLTSGSDEVMLFSAAGKVVRFKEDAVRAMGRTATG |
| SRR5209740 | TEFSRPRSAGIIAVNLNDGDELIGVDLTSGSDEVMLFSAAGKVVRFKEDAVRAMGRTATG |
| SRR3240355 | TEFSRPRSAGIIAVNLNDGDELIGVDLTSGSDEVMLFSAAGKVVRFKEDAVRAMGRTATG |
| SRR3392777 | TEFSRPRSAGIIAVNLNDGDELIGVDLTSGSDEVMLFSAAGKVVRFKEDAVRAMGRTATG |
| SRR3593671 | TEFSRPRSAGIIAVNLNDGDELIGVDLTSGSDEVMLFSAAGKVVRFKEDAVRAMGRTATG |
| SRR5413290 | TEFSRPRSAGIIAVNLNDGDELIGVDLTSGSDEVMLFSAAGKVVRFKEDAVRAMGRTATG |
| SRR5590269 | TEFSRPRSAGIIAVNLNDGDELIGVDLTSGSDEVMLFSAAGKVVRFKEDAVRAMGRTATG |
| SRR5812103 | TEFSRPRSAGIIAVNLNDGDELIGVDLTSGSDEVMLFSAAGKVVRFKEDAVRAMGRTATG |
| SRR2830941 | TEFSRPRSAGIIAVNLNDGDELIGVDLTSGSDEVMLFSAAGKVVRFKEDAVRAMGRTATG |
| SRR2830966 | TEFSRPRSAGIIAVNLNDGDELIGVDLTSGSDEVMLFSAAGKVVRFKEDAVRAMGRTATG |
| SRR3137270 | TEFSRPRSAGIIAVNLNDGDELIGVDLTSGSDEVMLFSAAGKVVRFKEDAVRAMGRTATG |
| SRR3137271 | TEFSRPRSAGIIAVNLNDGDELIGVDLTSGSDEVMLFSAAGKVVRFKEDAVRAMGRTATG |
| ERR526807 | TEFSRPRSAGIIAVNLNDGDELIGVDLTSGSDEVMLFSAAGKVVRFKEDAVRAMGRTATG |
| ERR2197922 | TEFSRPRSAGIIAVNLNDGDELIGVDLTSGSDEVMLFSAAGKVVRFKEDAVRAMGRTATG |
| ERR2197923 | TEFSRPRSAGIIAVNLNDGDELIGVDLTSGSDEVMLFSAAGKVVRFKEDAVRAMGRTATG |
| ERR2197924 | TEFSRPRSAGIIAVNLNDGDELIGVDLTSGSDEVMLFSAAGKVVRFKEDAVRAMGRTATG |
| ERR2197925 | TEFSRPRSAGIIAVNLNDGDELIGVDLTSGSDEVMLFSAAGKVVRFKEDAVRAMGRTATG |
| ERR2197927 | TEFSRPRSAGIIAVNLNDGDELIGVDLTSGSDEVMLFSAAGKVVRFKEDAVRAMGRTATG |
| ERR2197929 | TEFSRPRSAGIIAVNLNDGDELIGVDLTSGSDEVMLFSAAGKVVRFKEDAVRAMGRTATG |
| SRR1648149 | TEFSRPRSAGIIAVNLNDGDELIGVDLTSGSDEVMLFSAAGKVVRFKEDAVRAMGRTATG |
| SRR1048299 | TEFSRPRSAGIIAVNLNDGDELIGVDLTSGSDEVMLFSAAGKVVRFKEDAVRAMGRTATG |
| SRR1300677 | TEFSRPRSAGIIAVNLNDGDELIGVDLTSGSDEVMLFSAAGKVVRFKEDAVRAMGRTATG |
| SRR1288356 | TEFSRPRSAGIIAVNLNDGDELIGVDLTSGSDEVMLFSAAGKVVRFKEDAVRAMGRTATG |
| SRR7426190 | TEFSRPRSAGIIAVNLNDGDELIGVDLTSGSDEVMLFSAAGKVVRFKEDAVRAMGRTATG |
| SRR7426192 | TEFSRPRSAGIIAVNLNDGDELIGVDLTSGSDEVMLFSAAGKVVRFKEDAVRAMGRTATG |
| SRR7426193 | TEFSRPRSAGIIAVNLNDGDELIGVDLTSGSDEVMLFSAAGKVVRFKEDAVRAMGRTATG |
| SRR7441832 | TEFSRPRSAGIIAVNLNDGDELIGVDLTSGSDEVMLFSAAGKVVRFKEDAVRAMGRTATG |
| SRR7426179 | TEFSRPRSAGIIAVNLNDGDELIGVDLTSGSDEVMLFSAAGKVVRFKEDAVRAMGRTATG |
| SRR7439238 | TEFSRPRSAGIIAVNLNDGDELIGVDLTSGSDEVMLFSAAGKVVRFKEDAVRAMGRTATG |
| SRR7439244 | TEFSRPRSAGIIAVNLNDGDELIGVDLTSGSDEVMLFSAAGKVVRFKEDAVRAMGRTATG |
| SRR7439259 | TEFSRPRSAGIIAVNLNDGDELIGVDLTSGSDEVMLFSAAGKVVRFKEDAVRAMGRTATG |
| SRR7439260 | TEFSRPRSAGIIAVNLNDGDELIGVDLTSGSDEVMLFSAAGKVVRFKEDAVRAMGRTATG |
| SRR7441786 | TEFSRPRSAGIIAVNLNDGDELIGVDLTSGSDEVMLFSAAGKVVRFKEDAVRAMGRTATG |
| SRR7441797 | TEFSRPRSAGIIAVNLNDGDELIGVDLTSGSDEVMLFSAAGKVVRFKEDAVRAMGRTATG |
| ERR1759093 | TEFSRPRSAGIIAVNLNDGDELIGVDLTSGSDEVMLFSAAGKVVRFKEDAVRAMGRTATG |
| ERR2580275 | TEFSRPRSAGIIAVNLNDGDELIGVDLTSGSDEVMLFSAAGKVVRFKEDAVRAMGRTATG |
| ERR1759204 | TEFSRPRSAGIIAVNLNDGDELIGVDLTSGSDEVMLFSAAGKVVRFKEDAVRAMGRTATG |
| SRR1300699 | TEFSRPRSAGIIAVNLNDGDELIGVDLTSGSDEVMLFSAAGKVVRFKEDAVRAMGRTATG |
| S_0825_17 | TEFSRPRSAGIIAVNLNDGDELIGVDLTSGSDEVMLFSAAGKVVRFKEDAVRAMGRTATG |
| SRR1958215 | TEFSRPRSAGIIAVNLNDGDELIGVDLTSGSDEVMLFSAAGKVVRFKEDAVRAMGRTATG |
| SRR1958540 | TEFSRPRSAGIIAVNLNDGDELIGVDLTSGSDEVMLFSAAGKVVRFKEDAVRAMGRTATG |
| SRR1958636 | TEFSRPRSAGIIAVNLNDGDELIGVDLTSGSDEVMLFSAAGKVVRFKEDAVRAMGRTATG |
| SRR1959422 | TEFSRPRSAGIIAVNLNDGDELIGVDLTSGSDEVMLFSAAGKVVRFKEDAVRAMGRTATG |
| SRR1959427 | TEFSRPRSAGIIAVNLNDGDELIGVDLTSGSDEVMLFSAAGKVVRFKEDAVRAMGRTATG |
| SRR1960226 | TEFSRPRSAGIIAVNLNDGDELIGVDLTSGSDEVMLFSAAGKVVRFKEDAVRAMGRTATG |
| SRR1963498 | TEFSRPRSAGIIAVNLNDGDELIGVDLTSGSDEVMLFSAAGKVVRFKEDAVRAMGRTATG |
| SRR1965947 | TEFSRPRSAGIIAVNLNDGDELIGVDLTSGSDEVMLFSAAGKVVRFKEDAVRAMGRTATG |
| SRR1966125 | TEFSRPRSAGIIAVNLNDGDELIGVDLTSGSDEVMLFSAAGKVVRFKEDAVRAMGRTATG |
| SRR1966330 | TEFSRPRSAGIIAVNLNDGDELIGVDLTSGSDEVMLFSAAGKVVRFKEDAVRAMGRTATG |
| SRR1966565 | TEFSRPRSAGIIAVNLNDGDELIGVDLTSGSDEVMLFSAAGKVVRFKEDAVRAMGRTATG |
| SRR1966864 | TEFSRPRSAGIIAVNLNDGDELIGVDLTSGSDEVMLFSAAGKVVRFKEDAVRAMGRTATG |
| SRR1966989 | TEFSRPRSAGIIAVNLNDGDELIGVDLTSGSDEVMLFSAAGKVVRFKEDAVRAMGRTATG |
| SRR1967688 | TEFSRPRSAGIIAVNLNDGDELIGVDLTSGSDEVMLFSAAGKVVRFKEDAVRAMGRTATG |
| SRR1967733 | TEFSRPRSAGIIAVNLNDGDELIGVDLTSGSDEVMLFSAAGKVVRFKEDAVRAMGRTATG |
| SRR1967746 | TEFSRPRSAGIIAVNLNDGDELIGVDLTSGSDEVMLFSAAGKVVRFKEDAVRAMGRTATG |
| SRR1968341 | TEFSRPRSAGIIAVNLNDGDELIGVDLTSGSDEVMLFSAAGKVVRFKEDAVRAMGRTATG |
| SRR1968456 | TEFSRPRSAGIIAVNLNDGDELIGVDLTSGSDEVMLFSAAGKVVRFKEDAVRAMGRTATG |
| SRR1968465 | TEFSRPRSAGIIAVNLNDGDELIGVDLTSGSDEVMLFSAAGKVVRFKEDAVRAMGRTATG |
| SRR1968761 | TEFSRPRSAGIIAVNLNDGDELIGVDLTSGSDEVMLFSAAGKVVRFKEDAVRAMGRTATG |
| SRR1969047 | TEFSRPRSAGIIAVNLNDGDELIGVDLTSGSDEVMLFSAAGKVVRFKEDAVRAMGRTATG |
| SRR1969255 | TEFSRPRSAGIIAVNLNDGDELIGVDLTSGSDEVMLFSAAGKVVRFKEDAVRAMGRTATG |
| SRR1969412 | TEFSRPRSAGIIAVNLNDGDELIGVDLTSGSDEVMLFSAAGKVVRFKEDAVRAMGRTATG |
| SRR1969524 | TEFSRPRSAGIIAVNLNDGDELIGVDLTSGSDEVMLFSAAGKVVRFKEDAVRAMGRTATG |
| SRR1969584 | TEFSRPRSAGIIAVNLNDGDELIGVDLTSGSDEVMLFSAAGKVVRFKEDAVRAMGRTATG |
| SRR1969648 | TEFSRPRSAGIIAVNLNDGDELIGVDLTSGSDEVMLFSAAGKVVRFKEDAVRAMGRTATG |
| SRR1969804 | TEFSRPRSAGIIAVNLNDGDELIGVDLTSGSDEVMLFSAAGKVVRFKEDAVRAMGRTATG |
| SRR1970221 | TEFSRPRSAGIIAVNLNDGDELIGVDLTSGSDEVMLFSAAGKVVRFKEDAVRAMGRTATG |
| SRR1970268 | TEFSRPRSAGIIAVNLNDGDELIGVDLTSGSDEVMLFSAAGKVVRFKEDAVRAMGRTATG |
| SRR1965862 | TEFSRPRSAGIIAVNLNDGDELIGVDLTSGSDEVMLFSAAGKVVRFKEDAVRAMGRTATG |
| SRR1967363 | TEFSRPRSAGIIAVNLNDGDELIGVDLTSGSDEVMLFSAAGKVVRFKEDAVRAMGRTATG |
| SRR1968276 | TEFSRPRSAGIIAVNLNDGDELIGVDLTSGSDEVMLFSAAGKVVRFKEDAVRAMGRTATG |
| SRR1968967 | TEFSRPRSAGIIAVNLNDGDELIGVDLTSGSDEVMLFSAAGKVVRFKEDAVRAMGRTATG |
| SRR3321531 | TEFSRPRSAGIIAVNLNDGDELIGVDLTSGSDEVMLFSAAGKVVRFKEDAVRAMGRTATG |
| SRR3321883 | TEFSRPRSAGIIAVNLNDGDELIGVDLTSGSDEVMLFSAAGKVVRFKEDAVRAMGRTATG |
| SRR3322413 | TEFSRPRSAGIIAVNLNDGDELIGVDLTSGSDEVMLFSAAGKVVRFKEDAVRAMGRTATG |
| SRR3323012 | TEFSRPRSAGIIAVNLNDGDELIGVDLTSGSDEVMLFSAAGKVVRFKEDAVRAMGRTATG |
| SRR5194289 | TEFSRPRSAGIIAVNLNDGDELIGVDLTSGSDEVMLFSAAGKVVRFKEDAVRAMGRTATG |
| SRR7163798 | TEFSRPRSAGIIAVNLNDGDELIGVDLTSGSDEVMLFSAAGKVVRFKEDAVRAMGRTATG |
| SRR7172610 | TEFSRPRSAGIIAVNLNDGDELIGVDLTSGSDEVMLFSAAGKVVRFKEDAVRAMGRTATG |
| SRR7204568 | TEFSRPRSAGIIAVNLNDGDELIGVDLTSGSDEVMLFSAAGKVVRFKEDAVRAMGRTATG |
| SRR7223230 | TEFSRPRSAGIIAVNLNDGDELIGVDLTSGSDEVMLFSAAGKVVRFKEDAVRAMGRTATG |
| SRR7230675 | TEFSRPRSAGIIAVNLNDGDELIGVDLTSGSDEVMLFSAAGKVVRFKEDAVRAMGRTATG |
| SRR7278056 | TEFSRPRSAGIIAVNLNDGDELIGVDLTSGSDEVMLFSAAGKVVRFKEDAVRAMGRTATG |
| SRR7278086 | TEFSRPRSAGIIAVNLNDGDELIGVDLTSGSDEVMLFSAAGKVVRFKEDAVRAMGRTATG |
| SRR7285841 | TEFSRPRSAGIIAVNLNDGDELIGVDLTSGSDEVMLFSAAGKVVRFKEDAVRAMGRTATG |
| SRR7292625 | TEFSRPRSAGIIAVNLNDGDELIGVDLTSGSDEVMLFSAAGKVVRFKEDAVRAMGRTATG |
| SRR7292665 | TEFSRPRSAGIIAVNLNDGDELIGVDLTSGSDEVMLFSAAGKVVRFKEDAVRAMGRTATG |
| SRR7297965 | TEFSRPRSAGIIAVNLNDGDELIGVDLTSGSDEVMLFSAAGKVVRFKEDAVRAMGRTATG |
| SRR7350726 | TEFSRPRSAGIIAVNLNDGDELIGVDLTSGSDEVMLFSAAGKVVRFKEDAVRAMGRTATG |
| SRR7410328 | TEFSRPRSAGIIAVNLNDGDELIGVDLTSGSDEVMLFSAAGKVVRFKEDAVRAMGRTATG |
| SRR7474665 | TEFSRPRSAGIIAVNLNDGDELIGVDLTSGSDEVMLFSAAGKVVRFKEDAVRAMGRTATG |
| SRR7523184 | TEFSRPRSAGIIAVNLNDGDELIGVDLTSGSDEVMLFSAAGKVVRFKEDAVRAMGRTATG |
| SRR7187264 | TEFSRPRSAGIIAVNLNDGDELIGVDLTSGSDEVMLFSAAGKVVRFKEDAVRAMGRTATG |
| SRR7204445 | TEFSRPRSAGIIAVNLNDGDELIGVDLTSGSDEVMLFSAAGKVVRFKEDAVRAMGRTATG |
| SRR7285641 | TEFSRPRSAGIIAVNLNDGDELIGVDLTSGSDEVMLFSAAGKVVRFKEDAVRAMGRTATG |
| SRR7286695 | TEFSRPRSAGIIAVNLNDGDELIGVDLTSGSDEVMLFSAAGKVVRFKEDAVRAMGRTATG |
| SRR7286705 | TEFSRPRSAGIIAVNLNDGDELIGVDLTSGSDEVMLFSAAGKVVRFKEDAVRAMGRTATG |
| SRR7292931 | TEFSRPRSAGIIAVNLNDGDELIGVDLTSGSDEVMLFSAAGKVVRFKEDAVRAMGRTATG |
| SRR7310349 | TEFSRPRSAGIIAVNLNDGDELIGVDLTSGSDEVMLFSAAGKVVRFKEDAVRAMGRTATG |
| SRR7351616 | TEFSRPRSAGIIAVNLNDGDELIGVDLTSGSDEVMLFSAAGKVVRFKEDAVRAMGRTATG |
| SRR7414818 | TEFSRPRSAGIIAVNLNDGDELIGVDLTSGSDEVMLFSAAGKVVRFKEDAVRAMGRTATG |
| SRR7426480 | TEFSRPRSAGIIAVNLNDGDELIGVDLTSGSDEVMLFSAAGKVVRFKEDAVRAMGRTATG |
| SRR5584105 | TEFSRPRSAGIIAVNLNDGDELIGVDLTSGSDEVMLFSAAGKVVRFKEDAVRAMGRTATG |
| SRR5584565 | TEFSRPRSAGIIAVNLNDGDELIGVDLTSGSDEVMLFSAAGKVVRFKEDAVRAMGRTATG |
| SRR5584614 | TEFSRPRSAGIIAVNLNDGDELIGVDLTSGSDEVMLFSAAGKVVRFKEDAVRAMGRTATG |
| SRR5631543 | TEFSRPRSAGIIAVNLNDGDELIGVDLTSGSDEVMLFSAAGKVVRFKEDAVRAMGRTATG |
| SRR5631553 | TEFSRPRSAGIIAVNLNDGDELIGVDLTSGSDEVMLFSAAGKVVRFKEDAVRAMGRTATG |
| SRR7123196 | TEFSRPRSAGIIAVNLNDGDELIGVDLTSGSDEVMLFSAAGKVVRFKEDAVRAMGRTATG |
| SRR7163819 | TEFSRPRSAGIIAVNLNDGDELIGVDLTSGSDEVMLFSAAGKVVRFKEDAVRAMGRTATG |
| SRR7163920 | TEFSRPRSAGIIAVNLNDGDELIGVDLTSGSDEVMLFSAAGKVVRFKEDAVRAMGRTATG |
| SRR7209528 | TEFSRPRSAGIIAVNLNDGDELIGVDLTSGSDEVMLFSAAGKVVRFKEDAVRAMGRTATG |
| SRR7249868 | TEFSRPRSAGIIAVNLNDGDELIGVDLTSGSDEVMLFSAAGKVVRFKEDAVRAMGRTATG |
| SRR7278088 | TEFSRPRSAGIIAVNLNDGDELIGVDLTSGSDEVMLFSAAGKVVRFKEDAVRAMGRTATG |
| SRR7285788 | TEFSRPRSAGIIAVNLNDGDELIGVDLTSGSDEVMLFSAAGKVVRFKEDAVRAMGRTATG |
| SRR7286789 | TEFSRPRSAGIIAVNLNDGDELIGVDLTSGSDEVMLFSAAGKVVRFKEDAVRAMGRTATG |
| SRR7286886 | TEFSRPRSAGIIAVNLNDGDELIGVDLTSGSDEVMLFSAAGKVVRFKEDAVRAMGRTATG |
| SRR7310632 | TEFSRPRSAGIIAVNLNDGDELIGVDLTSGSDEVMLFSAAGKVVRFKEDAVRAMGRTATG |
| SRR7350631 | TEFSRPRSAGIIAVNLNDGDELIGVDLTSGSDEVMLFSAAGKVVRFKEDAVRAMGRTATG |
| SRR7458741 | TEFSRPRSAGIIAVNLNDGDELIGVDLTSGSDEVMLFSAAGKVVRFKEDAVRAMGRTATG |
| SRR7480280 | TEFSRPRSAGIIAVNLNDGDELIGVDLTSGSDEVMLFSAAGKVVRFKEDAVRAMGRTATG |
| SRR7523660 | TEFSRPRSAGIIAVNLNDGDELIGVDLTSGSDEVMLFSAAGKVVRFKEDAVRAMGRTATG |
| SRR7523775 | TEFSRPRSAGIIAVNLNDGDELIGVDLTSGSDEVMLFSAAGKVVRFKEDAVRAMGRTATG |
| SRR7251101 | TEFSRPRSAGIIAVNLNDGDELIGVDLTSGSDEVMLFSAAGKVVRFKEDAVRAMGRTATG |
| SRR7284299 | TEFSRPRSAGIIAVNLNDGDELIGVDLTSGSDEVMLFSAAGKVVRFKEDAVRAMGRTATG |
| SRR7285738 | TEFSRPRSAGIIAVNLNDGDELIGVDLTSGSDEVMLFSAAGKVVRFKEDAVRAMGRTATG |
| SRR7310640 | TEFSRPRSAGIIAVNLNDGDELIGVDLTSGSDEVMLFSAAGKVVRFKEDAVRAMGRTATG |
| SRR7349159 | TEFSRPRSAGIIAVNLNDGDELIGVDLTSGSDEVMLFSAAGKVVRFKEDAVRAMGRTATG |
| SRR7474873 | TEFSRPRSAGIIAVNLNDGDELIGVDLTSGSDEVMLFSAAGKVVRFKEDAVRAMGRTATG |
| SRR7495689 | TEFSRPRSAGIIAVNLNDGDELIGVDLTSGSDEVMLFSAAGKVVRFKEDAVRAMGRTATG |
| SRR7495752 | TEFSRPRSAGIIAVNLNDGDELIGVDLTSGSDEVMLFSAAGKVVRFKEDAVRAMGRTATG |
| ----------------------------------------------------------------------------- | |
| S16BD08730 | VRGIKLAGDDKVVSLIIPRGEGAILTVTQNGYGKRTAADEYPTKSRATQGVISIKVTERN |
| S18BD00684 | VRGIKLAGDDKVVSLIIPRGEGAILTVTQNGYGKRTAADEYPTKSRATQGVISIKVTERN |
| S18BD03994 | VRGIKLAGDDKVVSLIIPRGEGAILTVTQNGYGKRTAADEYPTKSRATQGVISIKVTERN |
| S18BD05011 | VRGIKLAGDDKVVSLIIPRGEGAILTVTQNGYGKRTAADEYPTKSRATQGVISIKVTERN |
| RKI_16-03723 | VRGIKLAGDDKVVSLIIPRGEGAILTVTQNGYGKRTAADEYPTKSRATQGVISIKVTERN |
| RKI_16-04315 | VRGIKLAGDDKVVSLIIPRGEGAILTVTQNGYGKRTAADEYPTKSRATQGVISIKVTERN |
| RKI_17-02304 | VRGIKLAGDDKVVSLIIPRGEGAILTVTQNGYGKRTAADEYPTKSRATQGVISIKVTERN |
| RKI_17-02411 | VRGIKLAGDDKVVSLIIPRGEGAILTVTQNGYGKRTAADEYPTKSRATQGVISIKVTERN |
| RKI_17-02757 | VRGIKLAGDDKVVSLIIPRGEGAILTVTQNGYGKRTAADEYPTKSRATQGVISIKVTERN |
| RKI_17-04797 | VRGIKLAGDDKVVSLIIPRGEGAILTVTQNGYGKRTAADEYPTKSRATQGVISIKVTERN |
| RKI_17-06869 | VRGIKLAGDDKVVSLIIPRGEGAILTVTQNGYGKRTAADEYPTKSRATQGVISIKVTERN |
| ERR2580277 | VRGIKLAGDDKVVSLIIPRGEGAILTVTQNGYGKRTAADEYPTKSRATQGVISIKVTERN |
| ERR2580276 | VRGIKLAGDDKVVSLIIPRGEGAILTVTQNGYGKRTAADEYPTKSRATQGVISIKVTERN |
| ERR2580273 | VRGIKLAGDDKVVSLIIPRGEGAILTVTQNGYGKRTAADEYPTKSRATQGVISIKVTERN |
| ERR2580274 | VRGIKLAGDDKVVSLIIPRGEGAILTVTQNGYGKRTAADEYPTKSRATQGVISIKVTERN |
| ERR2173656 | VRGIKLAGDDKVVSLIIPRGEGAILTVTQNGYGKRTAADEYPTKSRATQGVISIKVTERN |
| 17041676 | VRGIKLAGDDKVVSLIIPRGEGAILTVTQNGYGKRTAADEYPTKSRATQGVISIKVTERN |
| MT16-000061 | VRGIKLAGDDKVVSLIIPRGEGAILTVTQNGYGKRTAADEYPTKSRATQGVISIKVTERN |
| MT16-019416 | VRGIKLAGDDKVVSLIIPRGEGAILTVTQNGYGKRTAADEYPTKSRATQGVISIKVTERN |
| MT16-027865 | VRGIKLAGDDKVVSLIIPRGEGAILTVTQNGYGKRTAADEYPTKSRATQGVISIKVTERN |
| MT16-031693 | VRGIKLAGDDKVVSLIIPRGEGAILTVTQNGYGKRTAADEYPTKSRATQGVISIKVTERN |
| MT16-040253 | VRGIKLAGDDKVVSLIIPRGEGAILTVTQNGYGKRTAADEYPTKSRATQGVISIKVTERN |
| MT16-045379 | VRGIKLAGDDKVVSLIIPRGEGAILTVTQNGYGKRTAADEYPTKSRATQGVISIKVTERN |
| MT16-442728 | VRGIKLAGDDKVVSLIIPRGEGAILTVTQNGYGKRTAADEYPTKSRATQGVISIKVTERN |
| MT16-462857 | VRGIKLAGDDKVVSLIIPRGEGAILTVTQNGYGKRTAADEYPTKSRATQGVISIKVTERN |
| MT16-480196 | VRGIKLAGDDKVVSLIIPRGEGAILTVTQNGYGKRTAADEYPTKSRATQGVISIKVTERN |
| MT16-861555 | VRGIKLAGDDKVVSLIIPRGEGAILTVTQNGYGKRTAADEYPTKSRATQGVISIKVTERN |
| MT17-076833 | VRGIKLAGDDKVVSLIIPRGEGAILTVTQNGYGKRTAADEYPTKSRATQGVISIKVTERN |
| MT17-110677 | VRGIKLAGDDKVVSLIIPRGEGAILTVTQNGYGKRTAADEYPTKSRATQGVISIKVTERN |
| MT17-131730 | VRGIKLAGDDKVVSLIIPRGEGAILTVTQNGYGKRTAADEYPTKSRATQGVISIKVTERN |
| MT17-140890 | VRGIKLAGDDKVVSLIIPRGEGAILTVTQNGYGKRTAADEYPTKSRATQGVISIKVTERN |
| MT17-141840 | VRGIKLAGDDKVVSLIIPRGEGAILTVTQNGYGKRTAADEYPTKSRATQGVISIKVTERN |
| MT17-152488 | VRGIKLAGDDKVVSLIIPRGEGAILTVTQNGYGKRTAADEYPTKSRATQGVISIKVTERN |
| MT17-157311 | VRGIKLAGDDKVVSLIIPRGEGAILTVTQNGYGKRTAADEYPTKSRATQGVISIKVTERN |
| MT17-161645 | VRGIKLAGDDKVVSLIIPRGEGAILTVTQNGYGKRTAADEYPTKSRATQGVISIKVTERN |
| MT17-167951 | VRGIKLAGDDKVVSLIIPRGEGAILTVTQNGYGKRTAADEYPTKSRATQGVISIKVTERN |
| MT18-217732 | VRGIKLAGDDKVVSLIIPRGEGAILTVTQNGYGKRTAADEYPTKSRATQGVISIKVTERN |
| MT18-252580 | VRGIKLAGDDKVVSLIIPRGEGAILTVTQNGYGKRTAADEYPTKSRATQGVISIKVTERN |
| RIVM_H_2009-01 | VRGIKLAGDDKVVSLIIPRGEGAILTVTQNGYGKRTAADEYPTKSRATQGVISIKVTERN |
| RIVM_H_2010-01 | VRGIKLAGDDKVVSLIIPRGEGAILTVTQNGYGKRTAADEYPTKSRATQGVISIKVTERN |
| RIVM_H_2010-02 | VRGIKLAGDDKVVSLIIPRGEGAILTVTQNGYGKRTAADEYPTKSRATQGVISIKVTERN |
| RIVM_H_2011-01 | VRGIKLAGDDKVVSLIIPRGEGAILTVTQNGYGKRTAADEYPTKSRATQGVISIKVTERN |
| RIVM_H_2011-02 | VRGIKLAGDDKVVSLIIPRGEGAILTVTQNGYGKRTAADEYPTKSRATQGVISIKVTERN |
| RIVM_H_2011-03 | VRGIKLAGDDKVVSLIIPRGEGAILTVTQNGYGKRTAADEYPTKSRATQGVISIKVTERN |
| RIVM_H_2013-01 | VRGIKLAGDDKVVSLIIPRGEGAILTVTQNGYGKRTAADEYPTKSRATQGVISIKVTERN |
| RIVM_H_2013-02 | VRGIKLAGDDKVVSLIIPRGEGAILTVTQNGYGKRTAADEYPTKSRATQGVISIKVTERN |
| RIVM_H_2014-01 | VRGIKLAGDDKVVSLIIPRGEGAILTVTQNGYGKRTAADEYPTKSRATQGVISIKVTERN |
| RIVM_H_2014-02 | VRGIKLAGDDKVVSLIIPRGEGAILTVTQNGYGKRTAADEYPTKSRATQGVISIKVTERN |
| RIVM_H_2016-01 | VRGIKLAGDDKVVSLIIPRGEGAILTVTQNGYGKRTAADEYPTKSRATQGVISIKVTERN |
| RIVM_H_2016-02 | VRGIKLAGDDKVVSLIIPRGEGAILTVTQNGYGKRTAADEYPTKSRATQGVISIKVTERN |
| RIVM_H_2016-03 | VRGIKLAGDDKVVSLIIPRGEGAILTVTQNGYGKRTAADEYPTKSRATQGVISIKVTERN |
| RIVM_H_2016-04 | VRGIKLAGDDKVVSLIIPRGEGAILTVTQNGYGKRTAADEYPTKSRATQGVISIKVTERN |
| RIVM_H_2016-05 | VRGIKLAGDDKVVSLIIPRGEGAILTVTQNGYGKRTAADEYPTKSRATQGVISIKVTERN |
| RIVM_H_2016-06 | VRGIKLAGDDKVVSLIIPRGEGAILTVTQNGYGKRTAADEYPTKSRATQGVISIKVTERN |
| RIVM_H_2016-07 | VRGIKLAGDDKVVSLIIPRGEGAILTVTQNGYGKRTAADEYPTKSRATQGVISIKVTERN |
| RIVM_H_2016-08 | VRGIKLAGDDKVVSLIIPRGEGAILTVTQNGYGKRTAADEYPTKSRATQGVISIKVTERN |
| RIVM_H_2016-09 | VRGIKLAGDDKVVSLIIPRGEGAILTVTQNGYGKRTAADEYPTKSRATQGVISIKVTERN |
| RIVM_H_2016-10 | VRGIKLAGDDKVVSLIIPRGEGAILTVTQNGYGKRTAADEYPTKSRATQGVISIKVTERN |
| RIVM_H_2016-11 | VRGIKLAGDDKVVSLIIPRGEGAILTVTQNGYGKRTAADEYPTKSRATQGVISIKVTERN |
| RIVM_H_2016-12 | VRGIKLAGDDKVVSLIIPRGEGAILTVTQNGYGKRTAADEYPTKSRATQGVISIKVTERN |
| RIVM_H_2016-13 | VRGIKLAGDDKVVSLIIPRGEGAILTVTQNGYGKRTAADEYPTKSRATQGVISIKVTERN |
| RIVM_H_2016-14 | VRGIKLAGDDKVVSLIIPRGEGAILTVTQNGYGKRTAADEYPTKSRATQGVISIKVTERN |
| RIVM_H_2016-15 | VRGIKLAGDDKVVSLIIPRGEGAILTVTQNGYGKRTAADEYPTKSRATQGVISIKVTERN |
| RIVM_H_2017-01 | VRGIKLAGDDKVVSLIIPRGEGAILTVTQNGYGKRTAADEYPTKSRATQGVISIKVTERN |
| RIVM_H_2017-02 | VRGIKLAGDDKVVSLIIPRGEGAILTVTQNGYGKRTAADEYPTKSRATQGVISIKVTERN |
| RIVM_H_2017-03 | VRGIKLAGDDKVVSLIIPRGEGAILTVTQNGYGKRTAADEYPTKSRATQGVISIKVTERN |
| RIVM_H_2017-04 | VRGIKLAGDDKVVSLIIPRGEGAILTVTQNGYGKRTAADEYPTKSRATQGVISIKVTERN |
| RIVM_H_2017-05 | VRGIKLAGDDKVVSLIIPRGEGAILTVTQNGYGKRTAADEYPTKSRATQGVISIKVTERN |
| RIVM_H_2017-06 | VRGIKLAGDDKVVSLIIPRGEGAILTVTQNGYGKRTAADEYPTKSRATQGVISIKVTERN |
| RIVM_H_2017-07 | VRGIKLAGDDKVVSLIIPRGEGAILTVTQNGYGKRTAADEYPTKSRATQGVISIKVTERN |
| RIVM_H_2017-08 | VRGIKLAGDDKVVSLIIPRGEGAILTVTQNGYGKRTAADEYPTKSRATQGVISIKVTERN |
| RIVM_H_2017-09 | VRGIKLAGDDKVVSLIIPRGEGAILTVTQNGYGKRTAADEYPTKSRATQGVISIKVTERN |
| RIVM_H_2017-10 | VRGIKLAGDDKVVSLIIPRGEGAILTVTQNGYGKRTAADEYPTKSRATQGVISIKVTERN |
| RIVM_H_2017-11 | VRGIKLAGDDKVVSLIIPRGEGAILTVTQNGYGKRTAADEYPTKSRATQGVISIKVTERN |
| RIVM_H_2017-12 | VRGIKLAGDDKVVSLIIPRGEGAILTVTQNGYGKRTAADEYPTKSRATQGVISIKVTERN |
| RIVM_H_2017-13 | VRGIKLAGDDKVVSLIIPRGEGAILTVTQNGYGKRTAADEYPTKSRATQGVISIKVTERN |
| RIVM_H_2017-14 | VRGIKLAGDDKVVSLIIPRGEGAILTVTQNGYGKRTAADEYPTKSRATQGVISIKVTERN |
| RIVM_H_2017-15 | VRGIKLAGDDKVVSLIIPRGEGAILTVTQNGYGKRTAADEYPTKSRATQGVISIKVTERN |
| RIVM_H_2017-16 | VRGIKLAGDDKVVSLIIPRGEGAILTVTQNGYGKRTAADEYPTKSRATQGVISIKVTERN |
| RIVM_H_2017-17 | VRGIKLAGDDKVVSLIIPRGEGAILTVTQNGYGKRTAADEYPTKSRATQGVISIKVTERN |
| RIVM_H_2017-18 | VRGIKLAGDDKVVSLIIPRGEGAILTVTQNGYGKRTAADEYPTKSRATQGVISIKVTERN |
| RIVM_H_2017-19 | VRGIKLAGDDKVVSLIIPRGEGAILTVTQNGYGKRTAADEYPTKSRATQGVISIKVTERN |
| 15EP001483 | VRGIKLAGDDKVVSLIIPRGEGAILTVTQNGYGKRTAADEYPTKSRATQGVISIKVTERN |
| 17EP002363 | VRGIKLAGDDKVVSLIIPRGEGAILTVTQNGYGKRTAADEYPTKSRATQGVISIKVTERN |
| S_0812_17 | VRGIKLAGDDKVVSLIIPRGEGAILTVTQNGYGKRTAADEYPTKSRATQGVISIKVTERN |
| SRR1957844 | VRGIKLAGDDKVVSLIIPRGEGAILTVTQNGYGKRTAADEYPTKSRATQGVISIKVTERN |
| SRR1958654 | VRGIKLAGDDKVVSLIIPRGEGAILTVTQNGYGKRTAADEYPTKSRATQGVISIKVTERN |
| SRR1965077 | VRGIKLAGDDKVVSLIIPRGEGAILTVTQNGYGKRTAADEYPTKSRATQGVISIKVTERN |
| SRR1966369 | VRGIKLAGDDKVVSLIIPRGEGAILTVTQNGYGKRTAADEYPTKSRATQGVISIKVTERN |
| SRR1967117 | VRGIKLAGDDKVVSLIIPRGEGAILTVTQNGYGKRTAADEYPTKSRATQGVISIKVTERN |
| SRR1967922 | VRGIKLAGDDKVVSLIIPRGEGAILTVTQNGYGKRTAADEYPTKSRATQGVISIKVTERN |
| SRR8704720 | VRGIKLAGDDKVVSLIIPRGEGAILTVTQNGYGKRTAADEYPTKSRATQGVISIKVTERN |
| SRR7216071 | VRGIKLAGDDKVVSLIIPRGEGAILTVTQNGYGKRTAADEYPTKSRATQGVISIKVTERN |
| SRR7349175 | VRGIKLAGDDKVVSLIIPRGEGAILTVTQNGYGKRTAADEYPTKSRATQGVISIKVTERN |
| SRR7523148 | VRGIKLAGDDKVVSLIIPRGEGAILTVTQNGYGKRTAADEYPTKSRATQGVISIKVTERN |
| SRR7523854 | VRGIKLAGDDKVVSLIIPRGEGAILTVTQNGYGKRTAADEYPTKSRATQGVISIKVTERN |
| 313865 | VRGIKLAGDDKVVSLIIPRGEGAILTVTQNGYGKRTAADEYPTKSRATQGVISIKVTERN |
| SRR7277793 | VRGIKLAGDDKVVSLIIPRGEGAILTVTQNGYGKRTAADEYPTKSRATQGVISIKVTERN |
| SRR7343877 | VRGIKLAGDDKVVSLIIPRGEGAILTVTQNGYGKRTAADEYPTKSRATQGVISIKVTERN |
| SRR7351477 | VRGIKLAGDDKVVSLIIPRGEGAILTVTQNGYGKRTAADEYPTKSRATQGVISIKVTERN |
| SRR5583183 | VRGIKLAGDDKVVSLIIPRGEGAILTVTQNGYGKRTAADEYPTKSRATQGVISIKVTERN |
| SRR5585240 | VRGIKLAGDDKVVSLIIPRGEGAILTVTQNGYGKRTAADEYPTKSRATQGVISIKVTERN |
| SRR7284317 | VRGIKLAGDDKVVSLIIPRGEGAILTVTQNGYGKRTAADEYPTKSRATQGVISIKVTERN |
| SRR7299161 | VRGIKLAGDDKVVSLIIPRGEGAILTVTQNGYGKRTAADEYPTKSRATQGVISIKVTERN |
| SRR7401730 | VRGIKLAGDDKVVSLIIPRGEGAILTVTQNGYGKRTAADEYPTKSRATQGVISIKVTERN |
| SRR7469092 | VRGIKLAGDDKVVSLIIPRGEGAILTVTQNGYGKRTAADEYPTKSRATQGVISIKVTERN |
| SRR7879556 | VRGIKLAGDDKVVSLIIPRGEGAILTVTQNGYGKRTAADEYPTKSRATQGVISIKVTERN |
| SRR8526100 | VRGIKLAGDDKVVSLIIPRGEGAILTVTQNGYGKRTAADEYPTKSRATQGVISIKVTERN |
| SRR8553991 | VRGIKLAGDDKVVSLIIPRGEGAILTVTQNGYGKRTAADEYPTKSRATQGVISIKVTERN |
| SRR7842487 | VRGIKLAGDDKVVSLIIPRGEGAILTVTQNGYGKRTAADEYPTKSRATQGVISIKVTERN |
| SRR8054524 | VRGIKLAGDDKVVSLIIPRGEGAILTVTQNGYGKRTAADEYPTKSRATQGVISIKVTERN |
| SRR8054525 | VRGIKLAGDDKVVSLIIPRGEGAILTVTQNGYGKRTAADEYPTKSRATQGVISIKVTERN |
| SRR8524733 | VRGIKLAGDDKVVSLIIPRGEGAILTVTQNGYGKRTAADEYPTKSRATQGVISIKVTERN |
| SRR4093291 | VRGIKLAGDDKVVSLIIPRGEGAILTVTQNGYGKRTAADEYPTKSRATQGVISIKVTERN |
| SRR4245549 | VRGIKLAGDDKVVSLIIPRGEGAILTVTQNGYGKRTAADEYPTKSRATQGVISIKVTERN |
| SRR3057154 | VRGIKLAGDDKVVSLIIPRGEGAILTVTQNGYGKRTAADEYPTKSRATQGVISIKVTERN |
| SRR1726150 | VRGIKLAGDDKVVSLIIPRGEGAILTVTQNGYGKRTAADEYPTKSRATQGVISIKVTERN |
| SRR1996141 | VRGIKLAGDDKVVSLIIPRGEGAILTVTQNGYGKRTAADEYPTKSRATQGVISIKVTERN |
| SRR1107842 | VRGIKLAGDDKVVSLIIPRGEGAILTVTQNGYGKRTAADEYPTKSRATQGVISIKVTERN |
| SRR1157587 | VRGIKLAGDDKVVSLIIPRGEGAILTVTQNGYGKRTAADEYPTKSRATQGVISIKVTERN |
| SRR3027706 | VRGIKLAGDDKVVSLIIPRGEGAILTVTQNGYGKRTAADEYPTKSRATQGVISIKVTERN |
| SRR3027707 | VRGIKLAGDDKVVSLIIPRGEGAILTVTQNGYGKRTAADEYPTKSRATQGVISIKVTERN |
| SRR3027708 | VRGIKLAGDDKVVSLIIPRGEGAILTVTQNGYGKRTAADEYPTKSRATQGVISIKVTERN |
| SRR3027710 | VRGIKLAGDDKVVSLIIPRGEGAILTVTQNGYGKRTAADEYPTKSRATQGVISIKVTERN |
| SRR3027711 | VRGIKLAGDDKVVSLIIPRGEGAILTVTQNGYGKRTAADEYPTKSRATQGVISIKVTERN |
| SRR3027716 | VRGIKLAGDDKVVSLIIPRGEGAILTVTQNGYGKRTAADEYPTKSRATQGVISIKVTERN |
| SRR3027717 | VRGIKLAGDDKVVSLIIPRGEGAILTVTQNGYGKRTAADEYPTKSRATQGVISIKVTERN |
| SRR3027719 | VRGIKLAGDDKVVSLIIPRGEGAILTVTQNGYGKRTAADEYPTKSRATQGVISIKVTERN |
| SRR3027721 | VRGIKLAGDDKVVSLIIPRGEGAILTVTQNGYGKRTAADEYPTKSRATQGVISIKVTERN |
| SRR3027723 | VRGIKLAGDDKVVSLIIPRGEGAILTVTQNGYGKRTAADEYPTKSRATQGVISIKVTERN |
| SRR3115978 | VRGIKLAGDDKVVSLIIPRGEGAILTVTQNGYGKRTAADEYPTKSRATQGVISIKVTERN |
| SRR2534093 | VRGIKLAGDDKVVSLIIPRGEGAILTVTQNGYGKRTAADEYPTKSRATQGVISIKVTERN |
| SRR2534094 | VRGIKLAGDDKVVSLIIPRGEGAILTVTQNGYGKRTAADEYPTKSRATQGVISIKVTERN |
| SRR2534095 | VRGIKLAGDDKVVSLIIPRGEGAILTVTQNGYGKRTAADEYPTKSRATQGVISIKVTERN |
| SRR2534108 | VRGIKLAGDDKVVSLIIPRGEGAILTVTQNGYGKRTAADEYPTKSRATQGVISIKVTERN |
| SRR1106464 | VRGIKLAGDDKVVSLIIPRGEGAILTVTQNGYGKRTAADEYPTKSRATQGVISIKVTERN |
| SRR1106463 | VRGIKLAGDDKVVSLIIPRGEGAILTVTQNGYGKRTAADEYPTKSRATQGVISIKVTERN |
| SRR6949610 | VRGIKLAGDDKVVSLIIPRGEGAILTVTQNGYGKRTAADEYPTKSRATQGVISIKVTERN |
| SRR6950452 | VRGIKLAGDDKVVSLIIPRGEGAILTVTQNGYGKRTAADEYPTKSRATQGVISIKVTERN |
| ERR2019831 | VRGIKLAGDDKVVSLIIPRGEGAILTVTQNGYGKRTAADEYPTKSRATQGVISIKVTERN |
| SRR2085693 | VRGIKLAGDDKVVSLIIPRGEGAILTVTQNGYGKRTAADEYPTKSRATQGVISIKVTERN |
| SRR2086898 | VRGIKLAGDDKVVSLIIPRGEGAILTVTQNGYGKRTAADEYPTKSRATQGVISIKVTERN |
| SRR2175312 | VRGIKLAGDDKVVSLIIPRGEGAILTVTQNGYGKRTAADEYPTKSRATQGVISIKVTERN |
| SRR2175360 | VRGIKLAGDDKVVSLIIPRGEGAILTVTQNGYGKRTAADEYPTKSRATQGVISIKVTERN |
| SRR5231997 | VRGIKLAGDDKVVSLIIPRGEGAILTVTQNGYGKRTAADEYPTKSRATQGVISIKVTERN |
| SRR5232003 | VRGIKLAGDDKVVSLIIPRGEGAILTVTQNGYGKRTAADEYPTKSRATQGVISIKVTERN |
| SRR5232015 | VRGIKLAGDDKVVSLIIPRGEGAILTVTQNGYGKRTAADEYPTKSRATQGVISIKVTERN |
| SRR949434 | VRGIKLAGDDKVVSLIIPRGEGAILTVTQNGYGKRTAADEYPTKSRATQGVISIKVTERN |
| SRR3216575 | VRGIKLAGDDKVVSLIIPRGEGAILTVTQNGYGKRTAADEYPTKSRATQGVISIKVTERN |
| SRR5205342 | VRGIKLAGDDKVVSLIIPRGEGAILTVTQNGYGKRTAADEYPTKSRATQGVISIKVTERN |
| SRR1501669 | VRGIKLAGDDKVVSLIIPRGEGAILTVTQNGYGKRTAADEYPTKSRATQGVISIKVTERN |
| SRR5209740 | VRGIKLAGDDKVVSLIIPRGEGAILTVTQNGYGKRTAADEYPTKSRATQGVISIKVTERN |
| SRR3240355 | VRGIKLAGDDKVVSLIIPRGEGAILTVTQNGYGKRTAADEYPTKSRATQGVISIKVTERN |
| SRR3392777 | VRGIKLAGDDKVVSLIIPRGEGAILTVTQNGYGKRTAADEYPTKSRATQGVISIKVTERN |
| SRR3593671 | VRGIKLAGDDKVVSLIIPRGEGAILTVTQNGYGKRTAADEYPTKSRATQGVISIKVTERN |
| SRR5413290 | VRGIKLAGDDKVVSLIIPRGEGAILTVTQNGYGKRTAADEYPTKSRATQGVISIKVTERN |
| SRR5590269 | VRGIKLAGDDKVVSLIIPRGEGAILTVTQNGYGKRTAADEYPTKSRATQGVISIKVTERN |
| SRR5812103 | VRGIKLAGDDKVVSLIIPRGEGAILTVTQNGYGKRTAADEYPTKSRATQGVISIKVTERN |
| SRR2830941 | VRGIKLAGDDKVVSLIIPRGEGAILTVTQNGYGKRTAADEYPTKSRATQGVISIKVTERN |
| SRR2830966 | VRGIKLAGDDKVVSLIIPRGEGAILTVTQNGYGKRTAADEYPTKSRATQGVISIKVTERN |
| SRR3137270 | VRGIKLAGDDKVVSLIIPRGEGAILTVTQNGYGKRTAADEYPTKSRATQGVISIKVTERN |
| SRR3137271 | VRGIKLAGDDKVVSLIIPRGEGAILTVTQNGYGKRTAADEYPTKSRATQGVISIKVTERN |
| ERR526807 | VRGIKLAGDDKVVSLIIPRGEGAILTVTQNGYGKRTAADEYPTKSRATQGVISIKVTERN |
| ERR2197922 | VRGIKLAGDDKVVSLIIPRGEGAILTVTQNGYGKRTAADEYPTKSRATQGVISIKVTERN |
| ERR2197923 | VRGIKLAGDDKVVSLIIPRGEGAILTVTQNGYGKRTAADEYPTKSRATQGVISIKVTERN |
| ERR2197924 | VRGIKLAGDDKVVSLIIPRGEGAILTVTQNGYGKRTAADEYPTKSRATQGVISIKVTERN |
| ERR2197925 | VRGIKLAGDDKVVSLIIPRGEGAILTVTQNGYGKRTAADEYPTKSRATQGVISIKVTERN |
| ERR2197927 | VRGIKLAGDDKVVSLIIPRGEGAILTVTQNGYGKRTAADEYPTKSRATQGVISIKVTERN |
| ERR2197929 | VRGIKLAGDDKVVSLIIPRGEGAILTVTQNGYGKRTAADEYPTKSRATQGVISIKVTERN |
| SRR1648149 | VRGIKLAGDDKVVSLIIPRGEGAILTVTQNGYGKRTAADEYPTKSRATQGVISIKVTERN |
| SRR1048299 | VRGIKLAGDDKVVSLIIPRGEGAILTVTQNGYGKRTAADEYPTKSRATQGVISIKVTERN |
| SRR1300677 | VRGIKLAGDDKVVSLIIPRGEGAILTVTQNGYGKRTAADEYPTKSRATQGVISIKVTERN |
| SRR1288356 | VRGIKLAGDDKVVSLIIPRGEGAILTVTQNGYGKRTAADEYPTKSRATQGVISIKVTERN |
| SRR7426190 | VRGIKLAGDDKVVSLIIPRGEGAILTVTQNGYGKRTAADEYPTKSRATQGVISIKVTERN |
| SRR7426192 | VRGIKLAGDDKVVSLIIPRGEGAILTVTQNGYGKRTAADEYPTKSRATQGVISIKVTERN |
| SRR7426193 | VRGIKLAGDDKVVSLIIPRGEGAILTVTQNGYGKRTAADEYPTKSRATQGVISIKVTERN |
| SRR7441832 | VRGIKLAGDDKVVSLIIPRGEGAILTVTQNGYGKRTAADEYPTKSRATQGVISIKVTERN |
| SRR7426179 | VRGIKLAGDDKVVSLIIPRGEGAILTVTQNGYGKRTAADEYPTKSRATQGVISIKVTERN |
| SRR7439238 | VRGIKLAGDDKVVSLIIPRGEGAILTVTQNGYGKRTAADEYPTKSRATQGVISIKVTERN |
| SRR7439244 | VRGIKLAGDDKVVSLIIPRGEGAILTVTQNGYGKRTAADEYPTKSRATQGVISIKVTERN |
| SRR7439259 | VRGIKLAGDDKVVSLIIPRGEGAILTVTQNGYGKRTAADEYPTKSRATQGVISIKVTERN |
| SRR7439260 | VRGIKLAGDDKVVSLIIPRGEGAILTVTQNGYGKRTAADEYPTKSRATQGVISIKVTERN |
| SRR7441786 | VRGIKLAGDDKVVSLIIPRGEGAILTVTQNGYGKRTAADEYPTKSRATQGVISIKVTERN |
| SRR7441797 | VRGIKLAGDDKVVSLIIPRGEGAILTVTQNGYGKRTAADEYPTKSRATQGVISIKVTERN |
| ERR1759093 | VRGIKLAGDDKVVSLIIPRGEGAILTVTQNGYGKRTAADEYPTKSRATQGVISIKVTERN |
| ERR2580275 | VRGIKLAGDDKVVSLIIPRGEGAILTVTQNGYGKRTAADEYPTKSRATQGVISIKVTERN |
| ERR1759204 | VRGIKLAGDDKVVSLIIPRGEGAILTVTQNGYGKRTAADEYPTKSRATQGVISIKVTERN |
| SRR1300699 | VRGIKLAGDDKVVSLIIPRGEGAILTVTQNGYGKRTAADEYPTKSRATQGVISIKVTERN |
| S_0825_17 | VRGIKLAGDDKVVSLIIPRGEGAILTVTQNGYGKRTAADEYPTKSRATQGVISIKVTERN |
| SRR1958215 | VRGIKLAGDDKVVSLIIPRGEGAILTVTQNGYGKRTAADEYPTKSRATQGVISIKVTERN |
| SRR1958540 | VRGIKLAGDDKVVSLIIPRGEGAILTVTQNGYGKRTAADEYPTKSRATQGVISIKVTERN |
| SRR1958636 | VRGIKLAGDDKVVSLIIPRGEGAILTVTQNGYGKRTAADEYPTKSRATQGVISIKVTERN |
| SRR1959422 | VRGIKLAGDDKVVSLIIPRGEGAILTVTQNGYGKRTAADEYPTKSRATQGVISIKVTERN |
| SRR1959427 | VRGIKLAGDDKVVSLIIPRGEGAILTVTQNGYGKRTAADEYPTKSRATQGVISIKVTERN |
| SRR1960226 | VRGIKLAGDDKVVSLIIPRGEGAILTVTQNGYGKRTAADEYPTKSRATQGVISIKVTERN |
| SRR1963498 | VRGIKLAGDDKVVSLIIPRGEGAILTVTQNGYGKRTAADEYPTKSRATQGVISIKVTERN |
| SRR1965947 | VRGIKLAGDDKVVSLIIPRGEGAILTVTQNGYGKRTAADEYPTKSRATQGVISIKVTERN |
| SRR1966125 | VRGIKLAGDDKVVSLIIPRGEGAILTVTQNGYGKRTAADEYPTKSRATQGVISIKVTERN |
| SRR1966330 | VRGIKLAGDDKVVSLIIPRGEGAILTVTQNGYGKRTAADEYPTKSRATQGVISIKVTERN |
| SRR1966565 | VRGIKLAGDDKVVSLIIPRGEGAILTVTQNGYGKRTAADEYPTKSRATQGVISIKVTERN |
| SRR1966864 | VRGIKLAGDDKVVSLIIPRGEGAILTVTQNGYGKRTAADEYPTKSRATQGVISIKVTERN |
| SRR1966989 | VRGIKLAGDDKVVSLIIPRGEGAILTVTQNGYGKRTAADEYPTKSRATQGVISIKVTERN |
| SRR1967688 | VRGIKLAGDDKVVSLIIPRGEGAILTVTQNGYGKRTAADEYPTKSRATQGVISIKVTERN |
| SRR1967733 | VRGIKLAGDDKVVSLIIPRGEGAILTVTQNGYGKRTAADEYPTKSRATQGVISIKVTERN |
| SRR1967746 | VRGIKLAGDDKVVSLIIPRGEGAILTVTQNGYGKRTAADEYPTKSRATQGVISIKVTERN |
| SRR1968341 | VRGIKLAGDDKVVSLIIPRGEGAILTVTQNGYGKRTAADEYPTKSRATQGVISIKVTERN |
| SRR1968456 | VRGIKLAGDDKVVSLIIPRGEGAILTVTQNGYGKRTAADEYPTKSRATQGVISIKVTERN |
| SRR1968465 | VRGIKLAGDDKVVSLIIPRGEGAILTVTQNGYGKRTAADEYPTKSRATQGVISIKVTERN |
| SRR1968761 | VRGIKLAGDDKVVSLIIPRGEGAILTVTQNGYGKRTAADEYPTKSRATQGVISIKVTERN |
| SRR1969047 | VRGIKLAGDDKVVSLIIPRGEGAILTVTQNGYGKRTAADEYPTKSRATQGVISIKVTERN |
| SRR1969255 | VRGIKLAGDDKVVSLIIPRGEGAILTVTQNGYGKRTAADEYPTKSRATQGVISIKVTERN |
| SRR1969412 | VRGIKLAGDDKVVSLIIPRGEGAILTVTQNGYGKRTAADEYPTKSRATQGVISIKVTERN |
| SRR1969524 | VRGIKLAGDDKVVSLIIPRGEGAILTVTQNGYGKRTAADEYPTKSRATQGVISIKVTERN |
| SRR1969584 | VRGIKLAGDDKVVSLIIPRGEGAILTVTQNGYGKRTAADEYPTKSRATQGVISIKVTERN |
| SRR1969648 | VRGIKLAGDDKVVSLIIPRGEGAILTVTQNGYGKRTAADEYPTKSRATQGVISIKVTERN |
| SRR1969804 | VRGIKLAGDDKVVSLIIPRGEGAILTVTQNGYGKRTAADEYPTKSRATQGVISIKVTERN |
| SRR1970221 | VRGIKLAGDDKVVSLIIPRGEGAILTVTQNGYGKRTAADEYPTKSRATQGVISIKVTERN |
| SRR1970268 | VRGIKLAGDDKVVSLIIPRGEGAILTVTQNGYGKRTAADEYPTKSRATQGVISIKVTERN |
| SRR1965862 | VRGIKLAGDDKVVSLIIPRGEGAILTVTQNGYGKRTAADEYPTKSRATQGVISIKVTERN |
| SRR1967363 | VRGIKLAGDDKVVSLIIPRGEGAILTVTQNGYGKRTAADEYPTKSRATQGVISIKVTERN |
| SRR1968276 | VRGIKLAGDDKVVSLIIPRGEGAILTVTQNGYGKRTAADEYPTKSRATQGVISIKVTERN |
| SRR1968967 | VRGIKLAGDDKVVSLIIPRGEGAILTVTQNGYGKRTAADEYPTKSRATQGVISIKVTERN |
| SRR3321531 | VRGIKLAGDDKVVSLIIPRGEGAILTVTQNGYGKRTAADEYPTKSRATQGVISIKVTERN |
| SRR3321883 | VRGIKLAGDDKVVSLIIPRGEGAILTVTQNGYGKRTAADEYPTKSRATQGVISIKVTERN |
| SRR3322413 | VRGIKLAGDDKVVSLIIPRGEGAILTVTQNGYGKRTAADEYPTKSRATQGVISIKVTERN |
| SRR3323012 | VRGIKLAGDDKVVSLIIPRGEGAILTVTQNGYGKRTAADEYPTKSRATQGVISIKVTERN |
| SRR5194289 | VRGIKLAGDDKVVSLIIPRGEGAILTVTQNGYGKRTAADEYPTKSRATQGVISIKVTERN |
| SRR7163798 | VRGIKLAGDDKVVSLIIPRGEGAILTVTQNGYGKRTAADEYPTKSRATQGVISIKVTERN |
| SRR7172610 | VRGIKLAGDDKVVSLIIPRGEGAILTVTQNGYGKRTAADEYPTKSRATQGVISIKVTERN |
| SRR7204568 | VRGIKLAGDDKVVSLIIPRGEGAILTVTQNGYGKRTAADEYPTKSRATQGVISIKVTERN |
| SRR7223230 | VRGIKLAGDDKVVSLIIPRGEGAILTVTQNGYGKRTAADEYPTKSRATQGVISIKVTERN |
| SRR7230675 | VRGIKLAGDDKVVSLIIPRGEGAILTVTQNGYGKRTAADEYPTKSRATQGVISIKVTERN |
| SRR7278056 | VRGIKLAGDDKVVSLIIPRGEGAILTVTQNGYGKRTAADEYPTKSRATQGVISIKVTERN |
| SRR7278086 | VRGIKLAGDDKVVSLIIPRGEGAILTVTQNGYGKRTAADEYPTKSRATQGVISIKVTERN |
| SRR7285841 | VRGIKLAGDDKVVSLIIPRGEGAILTVTQNGYGKRTAADEYPTKSRATQGVISIKVTERN |
| SRR7292625 | VRGIKLAGDDKVVSLIIPRGEGAILTVTQNGYGKRTAADEYPTKSRATQGVISIKVTERN |
| SRR7292665 | VRGIKLAGDDKVVSLIIPRGEGAILTVTQNGYGKRTAADEYPTKSRATQGVISIKVTERN |
| SRR7297965 | VRGIKLAGDDKVVSLIIPRGEGAILTVTQNGYGKRTAADEYPTKSRATQGVISIKVTERN |
| SRR7350726 | VRGIKLAGDDKVVSLIIPRGEGAILTVTQNGYGKRTAADEYPTKSRATQGVISIKVTERN |
| SRR7410328 | VRGIKLAGDDKVVSLIIPRGEGAILTVTQNGYGKRTAADEYPTKSRATQGVISIKVTERN |
| SRR7474665 | VRGIKLAGDDKVVSLIIPRGEGAILTVTQNGYGKRTAADEYPTKSRATQGVISIKVTERN |
| SRR7523184 | VRGIKLAGDDKVVSLIIPRGEGAILTVTQNGYGKRTAADEYPTKSRATQGVISIKVTERN |
| SRR7187264 | VRGIKLAGDDKVVSLIIPRGEGAILTVTQNGYGKRTAADEYPTKSRATQGVISIKVTERN |
| SRR7204445 | VRGIKLAGDDKVVSLIIPRGEGAILTVTQNGYGKRTAADEYPTKSRATQGVISIKVTERN |
| SRR7285641 | VRGIKLAGDDKVVSLIIPRGEGAILTVTQNGYGKRTAADEYPTKSRATQGVISIKVTERN |
| SRR7286695 | VRGIKLAGDDKVVSLIIPRGEGAILTVTQNGYGKRTAADEYPTKSRATQGVISIKVTERN |
| SRR7286705 | VRGIKLAGDDKVVSLIIPRGEGAILTVTQNGYGKRTAADEYPTKSRATQGVISIKVTERN |
| SRR7292931 | VRGIKLAGDDKVVSLIIPRGEGAILTVTQNGYGKRTAADEYPTKSRATQGVISIKVTERN |
| SRR7310349 | VRGIKLAGDDKVVSLIIPRGEGAILTVTQNGYGKRTAADEYPTKSRATQGVISIKVTERN |
| SRR7351616 | VRGIKLAGDDKVVSLIIPRGEGAILTVTQNGYGKRTAADEYPTKSRATQGVISIKVTERN |
| SRR7414818 | VRGIKLAGDDKVVSLIIPRGEGAILTVTQNGYGKRTAADEYPTKSRATQGVISIKVTERN |
| SRR7426480 | VRGIKLAGDDKVVSLIIPRGEGAILTVTQNGYGKRTAADEYPTKSRATQGVISIKVTERN |
| SRR5584105 | VRGIKLAGDDKVVSLIIPRGEGAILTVTQNGYGKRTAADEYPTKSRATQGVISIKVTERN |
| SRR5584565 | VRGIKLAGDDKVVSLIIPRGEGAILTVTQNGYGKRTAADEYPTKSRATQGVISIKVTERN |
| SRR5584614 | VRGIKLAGDDKVVSLIIPRGEGAILTVTQNGYGKRTAADEYPTKSRATQGVISIKVTERN |
| SRR5631543 | VRGIKLAGDDKVVSLIIPRGEGAILTVTQNGYGKRTAADEYPTKSRATQGVISIKVTERN |
| SRR5631553 | VRGIKLAGDDKVVSLIIPRGEGAILTVTQNGYGKRTAADEYPTKSRATQGVISIKVTERN |
| SRR7123196 | VRGIKLAGDDKVVSLIIPRGEGAILTVTQNGYGKRTAADEYPTKSRATQGVISIKVTERN |
| SRR7163819 | VRGIKLAGDDKVVSLIIPRGEGAILTVTQNGYGKRTAADEYPTKSRATQGVISIKVTERN |
| SRR7163920 | VRGIKLAGDDKVVSLIIPRGEGAILTVTQNGYGKRTAADEYPTKSRATQGVISIKVTERN |
| SRR7209528 | VRGIKLAGDDKVVSLIIPRGEGAILTVTQNGYGKRTAADEYPTKSRATQGVISIKVTERN |
| SRR7249868 | VRGIKLAGDDKVVSLIIPRGEGAILTVTQNGYGKRTAADEYPTKSRATQGVISIKVTERN |
| SRR7278088 | VRGIKLAGDDKVVSLIIPRGEGAILTVTQNGYGKRTAADEYPTKSRATQGVISIKVTERN |
| SRR7285788 | VRGIKLAGDDKVVSLIIPRGEGAILTVTQNGYGKRTAADEYPTKSRATQGVISIKVTERN |
| SRR7286789 | VRGIKLAGDDKVVSLIIPRGEGAILTVTQNGYGKRTAADEYPTKSRATQGVISIKVTERN |
| SRR7286886 | VRGIKLAGDDKVVSLIIPRGEGAILTVTQNGYGKRTAADEYPTKSRATQGVISIKVTERN |
| SRR7310632 | VRGIKLAGDDKVVSLIIPRGEGAILTVTQNGYGKRTAADEYPTKSRATQGVISIKVTERN |
| SRR7350631 | VRGIKLAGDDKVVSLIIPRGEGAILTVTQNGYGKRTAADEYPTKSRATQGVISIKVTERN |
| SRR7458741 | VRGIKLAGDDKVVSLIIPRGEGAILTVTQNGYGKRTAADEYPTKSRATQGVISIKVTERN |
| SRR7480280 | VRGIKLAGDDKVVSLIIPRGEGAILTVTQNGYGKRTAADEYPTKSRATQGVISIKVTERN |
| SRR7523660 | VRGIKLAGDDKVVSLIIPRGEGAILTVTQNGYGKRTAADEYPTKSRATQGVISIKVTERN |
| SRR7523775 | VRGIKLAGDDKVVSLIIPRGEGAILTVTQNGYGKRTAADEYPTKSRATQGVISIKVTERN |
| SRR7251101 | VRGIKLAGDDKVVSLIIPRGEGAILTVTQNGYGKRTAADEYPTKSRATQGVISIKVTERN |
| SRR7284299 | VRGIKLAGDDKVVSLIIPRGEGAILTVTQNGYGKRTAADEYPTKSRATQGVISIKVTERN |
| SRR7285738 | VRGIKLAGDDKVVSLIIPRGEGAILTVTQNGYGKRTAADEYPTKSRATQGVISIKVTERN |
| SRR7310640 | VRGIKLAGDDKVVSLIIPRGEGAILTVTQNGYGKRTAADEYPTKSRATQGVISIKVTERN |
| SRR7349159 | VRGIKLAGDDKVVSLIIPRGEGAILTVTQNGYGKRTAADEYPTKSRATQGVISIKVTERN |
| SRR7474873 | VRGIKLAGDDKVVSLIIPRGEGAILTVTQNGYGKRTAADEYPTKSRATQGVISIKVTERN |
| SRR7495689 | VRGIKLAGDDKVVSLIIPRGEGAILTVTQNGYGKRTAADEYPTKSRATQGVISIKVTERN |
| SRR7495752 | VRGIKLAGDDKVVSLIIPRGEGAILTVTQNGYGKRTAADEYPTKSRATQGVISIKVTERN |
| ----------------------------------------------------------------------------- | |
| S16BD08730 | GSVVGAVQVDDCDQIMMITDAGTLVRTRVSEISVVGRNTQGVILIRTAEDENVVGLQRVA |
| S18BD00684 | GSVVGAVQVDDCDQIMMITDAGTLVRTRVSEISVVGRNTQGVILIRTAEDENVVGLQRVA |
| S18BD03994 | GSVVGAVQVDDCDQIMMITDAGTLVRTRVSEISVVGRNTQGVILIRTAEDENVVGLQRVA |
| S18BD05011 | GSVVGAVQVDDCDQIMMITDAGTLVRTRVSEISVVGRNTQGVILIRTAEDENVVGLQRVA |
| RKI_16-03723 | GSVVGAVQVDDCDQIMMITDAGTLVRTRVSEISVVGRNTQGVILIRTAEDENVVGLQRVA |
| RKI_16-04315 | GSVVGAVQVDDCDQIMMITDAGTLVRTRVSEISVVGRNTQGVILIRTAEDENVVGLQRVA |
| RKI_17-02304 | GSVVGAVQVDDCDQIMMITDAGTLVRTRVSEISVVGRNTQGVILIRTAEDENVVGLQRVA |
| RKI_17-02411 | GSVVGAVQVDDCDQIMMITDAGTLVRTRVSEISVVGRNTQGVILIRTAEDENVVGLQRVA |
| RKI_17-02757 | GSVVGAVQVDDCDQIMMITDAGTLVRTRVSEISVVGRNTQGVILIRTAEDENVVGLQRVA |
| RKI_17-04797 | GSVVGAVQVDDCDQIMMITDAGTLVRTRVSEISVVGRNTQGVILIRTAEDENVVGLQRVA |
| RKI_17-06869 | GSVVGAVQVDDCDQIMMITDAGTLVRTRVSEISVVGRNTQGVILIRTAEDENVVGLQRVA |
| ERR2580277 | GSVVGAVQVDDCDQIMMITDAGTLVRTRVSEISVVGRNTQGVILIRTAEDENVVGLQRVA |
| ERR2580276 | GSVVGAVQVDDCDQIMMITDAGTLVRTRVSEISVVGRNTQGVILIRTAEDENVVGLQRVA |
| ERR2580273 | GSVVGAVQVDDCDQIMMITDAGTLVRTRVSEISVVGRNTQGVILIRTAEDENVVGLQRVA |
| ERR2580274 | GSVVGAVQVDDCDQIMMITDAGTLVRTRVSEISVVGRNTQGVILIRTAEDENVVGLQRVA |
| ERR2173656 | GSVVGAVQVDDCDQIMMITDAGTLVRTRVSEISVVGRNTQGVILIRTAEDENVVGLQRVA |
| 17041676 | GSVVGAVQVDDCDQIMMITDAGTLVRTRVSEISVVGRNTQGVILIRTAEDENVVGLQRVA |
| MT16-000061 | GSVVGAVQVDDCDQIMMITDAGTLVRTRVSEISVVGRNTQGVILIRTAEDENVVGLQRVA |
| MT16-019416 | GSVVGAVQVDDCDQIMMITDAGTLVRTRVSEISVVGRNTQGVILIRTAEDENVVGLQRVA |
| MT16-027865 | GSVVGAVQVDDCDQIMMITDAGTLVRTRVSEISVVGRNTQGVILIRTAEDENVVGLQRVA |
| MT16-031693 | GSVVGAVQVDDCDQIMMITDAGTLVRTRVSEISVVGRNTQGVILIRTAEDENVVGLQRVA |
| MT16-040253 | GSVVGAVQVDDCDQIMMITDAGTLVRTRVSEISVVGRNTQGVILIRTAEDENVVGLQRVA |
| MT16-045379 | GSVVGAVQVDDCDQIMMITDAGTLVRTRVSEISVVGRNTQGVILIRTAEDENVVGLQRVA |
| MT16-442728 | GSVVGAVQVDDCDQIMMITDAGTLVRTRVSEISVVGRNTQGVILIRTAEDENVVGLQRVA |
| MT16-462857 | GSVVGAVQVDDCDQIMMITDAGTLVRTRVSEISVVGRNTQGVILIRTAEDENVVGLQRVA |
| MT16-480196 | GSVVGAVQVDDCDQIMMITDAGTLVRTRVSEISVVGRNTQGVILIRTAEDENVVGLQRVA |
| MT16-861555 | GSVVGAVQVDDCDQIMMITDAGTLVRTRVSEISVVGRNTQGVILIRTAEDENVVGLQRVA |
| MT17-076833 | GSVVGAVQVDDCDQIMMITDAGTLVRTRVSEISVVGRNTQGVILIRTAEDENVVGLQRVA |
| MT17-110677 | GSVVGAVQVDDCDQIMMITDAGTLVRTRVSEISVVGRNTQGVILIRTAEDENVVGLQRVA |
| MT17-131730 | GSVVGAVQVDDCDQIMMITDAGTLVRTRVSEISVVGRNTQGVILIRTAEDENVVGLQRVA |
| MT17-140890 | GSVVGAVQVDDCDQIMMITDAGTLVRTRVSEISVVGRNTQGVILIRTAEDENVVGLQRVA |
| MT17-141840 | GSVVGAVQVDDCDQIMMITDAGTLVRTRVSEISVVGRNTQGVILIRTAEDENVVGLQRVA |
| MT17-152488 | GSVVGAVQVDDCDQIMMITDAGTLVRTRVSEISVVGRNTQGVILIRTAEDENVVGLQRVA |
| MT17-157311 | GSVVGAVQVDDCDQIMMITDAGTLVRTRVSEISVVGRNTQGVILIRTAEDENVVGLQRVA |
| MT17-161645 | GSVVGAVQVDDCDQIMMITDAGTLVRTRVSEISVVGRNTQGVILIRTAEDENVVGLQRVA |
| MT17-167951 | GSVVGAVQVDDCDQIMMITDAGTLVRTRVSEISVVGRNTQGVILIRTAEDENVVGLQRVA |
| MT18-217732 | GSVVGAVQVDDCDQIMMITDAGTLVRTRVSEISVVGRNTQGVILIRTAEDENVVGLQRVA |
| MT18-252580 | GSVVGAVQVDDCDQIMMITDAGTLVRTRVSEISVVGRNTQGVILIRTAEDENVVGLQRVA |
| RIVM_H_2009-01 | GSVVGAVQVDDCDQIMMITDAGTLVRTRVSEISVVGRNTQGVILIRTAEDENVVGLQRVA |
| RIVM_H_2010-01 | GSVVGAVQVDDCDQIMMITDAGTLVRTRVSEISVVGRNTQGVILIRTAEDENVVGLQRVA |
| RIVM_H_2010-02 | GSVVGAVQVDDCDQIMMITDAGTLVRTRVSEISVVGRNTQGVILIRTAEDENVVGLQRVA |
| RIVM_H_2011-01 | GSVVGAVQVDDCDQIMMITDAGTLVRTRVSEISVVGRNTQGVILIRTAEDENVVGLQRVA |
| RIVM_H_2011-02 | GSVVGAVQVDDCDQIMMITDAGTLVRTRVSEISVVGRNTQGVILIRTAEDENVVGLQRVA |
| RIVM_H_2011-03 | GSVVGAVQVDDCDQIMMITDAGTLVRTRVSEISVVGRNTQGVILIRTAEDENVVGLQRVA |
| RIVM_H_2013-01 | GSVVGAVQVDDCDQIMMITDAGTLVRTRVSEISVVGRNTQGVILIRTAEDENVVGLQRVA |
| RIVM_H_2013-02 | GSVVGAVQVDDCDQIMMITDAGTLVRTRVSEISVVGRNTQGVILIRTAEDENVVGLQRVA |
| RIVM_H_2014-01 | GSVVGAVQVDDCDQIMMITDAGTLVRTRVSEISVVGRNTQGVILIRTAEDENVVGLQRVA |
| RIVM_H_2014-02 | GSVVGAVQVDDCDQIMMITDAGTLVRTRVSEISVVGRNTQGVILIRTAEDENVVGLQRVA |
| RIVM_H_2016-01 | GSVVGAVQVDDCDQIMMITDAGTLVRTRVSEISVVGRNTQGVILIRTAEDENVVGLQRVA |
| RIVM_H_2016-02 | GSVVGAVQVDDCDQIMMITDAGTLVRTRVSEISVVGRNTQGVILIRTAEDENVVGLQRVA |
| RIVM_H_2016-03 | GSVVGAVQVDDCDQIMMITDAGTLVRTRVSEISVVGRNTQGVILIRTAEDENVVGLQRVA |
| RIVM_H_2016-04 | GSVVGAVQVDDCDQIMMITDAGTLVRTRVSEISVVGRNTQGVILIRTAEDENVVGLQRVA |
| RIVM_H_2016-05 | GSVVGAVQVDDCDQIMMITDAGTLVRTRVSEISVVGRNTQGVILIRTAEDENVVGLQRVA |
| RIVM_H_2016-06 | GSVVGAVQVDDCDQIMMITDAGTLVRTRVSEISVVGRNTQGVILIRTAEDENVVGLQRVA |
| RIVM_H_2016-07 | GSVVGAVQVDDCDQIMMITDAGTLVRTRVSEISVVGRNTQGVILIRTAEDENVVGLQRVA |
| RIVM_H_2016-08 | GSVVGAVQVDDCDQIMMITDAGTLVRTRVSEISVVGRNTQGVILIRTAEDENVVGLQRVA |
| RIVM_H_2016-09 | GSVVGAVQVDDCDQIMMITDAGTLVRTRVSEISVVGRNTQGVILIRTAEDENVVGLQRVA |
| RIVM_H_2016-10 | GSVVGAVQVDDCDQIMMITDAGTLVRTRVSEISVVGRNTQGVILIRTAEDENVVGLQRVA |
| RIVM_H_2016-11 | GSVVGAVQVDDCDQIMMITDAGTLVRTRVSEISVVGRNTQGVILIRTAEDENVVGLQRVA |
| RIVM_H_2016-12 | GSVVGAVQVDDCDQIMMITDAGTLVRTRVSEISVVGRNTQGVILIRTAEDENVVGLQRVA |
| RIVM_H_2016-13 | GSVVGAVQVDDCDQIMMITDAGTLVRTRVSEISVVGRNTQGVILIRTAEDENVVGLQRVA |
| RIVM_H_2016-14 | GSVVGAVQVDDCDQIMMITDAGTLVRTRVSEISVVGRNTQGVILIRTAEDENVVGLQRVA |
| RIVM_H_2016-15 | GSVVGAVQVDDCDQIMMITDAGTLVRTRVSEISVVGRNTQGVILIRTAEDENVVGLQRVA |
| RIVM_H_2017-01 | GSVVGAVQVDDCDQIMMITDAGTLVRTRVSEISVVGRNTQGVILIRTAEDENVVGLQRVA |
| RIVM_H_2017-02 | GSVVGAVQVDDCDQIMMITDAGTLVRTRVSEISVVGRNTQGVILIRTAEDENVVGLQRVA |
| RIVM_H_2017-03 | GSVVGAVQVDDCDQIMMITDAGTLVRTRVSEISVVGRNTQGVILIRTAEDENVVGLQRVA |
| RIVM_H_2017-04 | GSVVGAVQVDDCDQIMMITDAGTLVRTRVSEISVVGRNTQGVILIRTAEDENVVGLQRVA |
| RIVM_H_2017-05 | GSVVGAVQVDDCDQIMMITDAGTLVRTRVSEISVVGRNTQGVILIRTAEDENVVGLQRVA |
| RIVM_H_2017-06 | GSVVGAVQVDDCDQIMMITDAGTLVRTRVSEISVVGRNTQGVILIRTAEDENVVGLQRVA |
| RIVM_H_2017-07 | GSVVGAVQVDDCDQIMMITDAGTLVRTRVSEISVVGRNTQGVILIRTAEDENVVGLQRVA |
| RIVM_H_2017-08 | GSVVGAVQVDDCDQIMMITDAGTLVRTRVSEISVVGRNTQGVILIRTAEDENVVGLQRVA |
| RIVM_H_2017-09 | GSVVGAVQVDDCDQIMMITDAGTLVRTRVSEISVVGRNTQGVILIRTAEDENVVGLQRVA |
| RIVM_H_2017-10 | GSVVGAVQVDDCDQIMMITDAGTLVRTRVSEISVVGRNTQGVILIRTAEDENVVGLQRVA |
| RIVM_H_2017-11 | GSVVGAVQVDDCDQIMMITDAGTLVRTRVSEISVVGRNTQGVILIRTAEDENVVGLQRVA |
| RIVM_H_2017-12 | GSVVGAVQVDDCDQIMMITDAGTLVRTRVSEISVVGRNTQGVILIRTAEDENVVGLQRVA |
| RIVM_H_2017-13 | GSVVGAVQVDDCDQIMMITDAGTLVRTRVSEISVVGRNTQGVILIRTAEDENVVGLQRVA |
| RIVM_H_2017-14 | GSVVGAVQVDDCDQIMMITDAGTLVRTRVSEISVVGRNTQGVILIRTAEDENVVGLQRVA |
| RIVM_H_2017-15 | GSVVGAVQVDDCDQIMMITDAGTLVRTRVSEISVVGRNTQGVILIRTAEDENVVGLQRVA |
| RIVM_H_2017-16 | GSVVGAVQVDDCDQIMMITDAGTLVRTRVSEISVVGRNTQGVILIRTAEDENVVGLQRVA |
| RIVM_H_2017-17 | GSVVGAVQVDDCDQIMMITDAGTLVRTRVSEISVVGRNTQGVILIRTAEDENVVGLQRVA |
| RIVM_H_2017-18 | GSVVGAVQVDDCDQIMMITDAGTLVRTRVSEISVVGRNTQGVILIRTAEDENVVGLQRVA |
| RIVM_H_2017-19 | GSVVGAVQVDDCDQIMMITDAGTLVRTRVSEISVVGRNTQGVILIRTAEDENVVGLQRVA |
| 15EP001483 | GSVVGAVQVDDCDQIMMITDAGTLVRTRVSEISVVGRNTQGVILIRTAEDENVVGLQRVA |
| 17EP002363 | GSVVGAVQVDDCDQIMMITDAGTLVRTRVSEISVVGRNTQGVILIRTAEDENVVGLQRVA |
| S_0812_17 | GSVVGAVQVDDCDQIMMITDAGTLVRTRVSEISVVGRNTQGVILIRTAEDENVVGLQRVA |
| SRR1957844 | GSVVGAVQVDDCDQIMMITDAGTLVRTRVSEISVVGRNTQGVILIRTAEDENVVGLQRVA |
| SRR1958654 | GSVVGAVQVDDCDQIMMITDAGTLVRTRVSEISVVGRNTQGVILIRTAEDENVVGLQRVA |
| SRR1965077 | GSVVGAVQVDDCDQIMMITDAGTLVRTRVSEISVVGRNTQGVILIRTAEDENVVGLQRVA |
| SRR1966369 | GSVVGAVQVDDCDQIMMITDAGTLVRTRVSEISVVGRNTQGVILIRTAEDENVVGLQRVA |
| SRR1967117 | GSVVGAVQVDDCDQIMMITDAGTLVRTRVSEISVVGRNTQGVILIRTAEDENVVGLQRVA |
| SRR1967922 | GSVVGAVQVDDCDQIMMITDAGTLVRTRVSEISVVGRNTQGVILIRTAEDENVVGLQRVA |
| SRR8704720 | GSVVGAVQVDDCDQIMMITDAGTLVRTRVSEISVVGRNTQGVILIRTAEDENVVGLQRVA |
| SRR7216071 | GSVVGAVQVDDCDQIMMITDAGTLVRTRVSEISVVGRNTQGVILIRTAEDENVVGLQRVA |
| SRR7349175 | GSVVGAVQVDDCDQIMMITDAGTLVRTRVSEISVVGRNTQGVILIRTAEDENVVGLQRVA |
| SRR7523148 | GSVVGAVQVDDCDQIMMITDAGTLVRTRVSEISVVGRNTQGVILIRTAEDENVVGLQRVA |
| SRR7523854 | GSVVGAVQVDDCDQIMMITDAGTLVRTRVSEISVVGRNTQGVILIRTAEDENVVGLQRVA |
| 313865 | GSVVGAVQVDDCDQIMMITDAGTLVRTRVSEISVVGRNTQGVILIRTAEDENVVGLQRVA |
| SRR7277793 | GSVVGAVQVDDCDQIMMITDAGTLVRTRVSEISVVGRNTQGVILIRTAEDENVVGLQRVA |
| SRR7343877 | GSVVGAVQVDDCDQIMMITDAGTLVRTRVSEISVVGRNTQGVILIRTAEDENVVGLQRVA |
| SRR7351477 | GSVVGAVQVDDCDQIMMITDAGTLVRTRVSEISVVGRNTQGVILIRTAEDENVVGLQRVA |
| SRR5583183 | GSVVGAVQVDDCDQIMMITDAGTLVRTRVSEISVVGRNTQGVILIRTAEDENVVGLQRVA |
| SRR5585240 | GSVVGAVQVDDCDQIMMITDAGTLVRTRVSEISVVGRNTQGVILIRTAEDENVVGLQRVA |
| SRR7284317 | GSVVGAVQVDDCDQIMMITDAGTLVRTRVSEISVVGRNTQGVILIRTAEDENVVGLQRVA |
| SRR7299161 | GSVVGAVQVDDCDQIMMITDAGTLVRTRVSEISVVGRNTQGVILIRTAEDENVVGLQRVA |
| SRR7401730 | GSVVGAVQVDDCDQIMMITDAGTLVRTRVSEISVVGRNTQGVILIRTAEDENVVGLQRVA |
| SRR7469092 | GSVVGAVQVDDCDQIMMITDAGTLVRTRVSEISVVGRNTQGVILIRTAEDENVVGLQRVA |
| SRR7879556 | GSVVGAVQVDDCDQIMMITDAGTLVRTRVSEISVVGRNTQGVILIRTAEDENVVGLQRVA |
| SRR8526100 | GSVVGAVQVDDCDQIMMITDAGTLVRTRVSEISVVGRNTQGVILIRTAEDENVVGLQRVA |
| SRR8553991 | GSVVGAVQVDDCDQIMMITDAGTLVRTRVSEISVVGRNTQGVILIRTAEDENVVGLQRVA |
| SRR7842487 | GSVVGAVQVDDCDQIMMITDAGTLVRTRVSEISVVGRNTQGVILIRTAEDENVVGLQRVA |
| SRR8054524 | GSVVGAVQVDDCDQIMMITDAGTLVRTRVSEISVVGRNTQGVILIRTAEDENVVGLQRVA |
| SRR8054525 | GSVVGAVQVDDCDQIMMITDAGTLVRTRVSEISVVGRNTQGVILIRTAEDENVVGLQRVA |
| SRR8524733 | GSVVGAVQVDDCDQIMMITDAGTLVRTRVSEISVVGRNTQGVILIRTAEDENVVGLQRVA |
| SRR4093291 | GSVVGAVQVDDCDQIMMITDAGTLVRTRVSEISVVGRNTQGVILIRTAEDENVVGLQRVA |
| SRR4245549 | GSVVGAVQVDDCDQIMMITDAGTLVRTRVSEISVVGRNTQGVILIRTAEDENVVGLQRVA |
| SRR3057154 | GSVVGAVQVDDCDQIMMITDAGTLVRTRVSEISVVGRNTQGVILIRTAEDENVVGLQRVA |
| SRR1726150 | GSVVGAVQVDDCDQIMMITDAGTLVRTRVSEISVVGRNTQGVILIRTAEDENVVGLQRVA |
| SRR1996141 | GSVVGAVQVDDCDQIMMITDAGTLVRTRVSEISVVGRNTQGVILIRTAEDENVVGLQRVA |
| SRR1107842 | GSVVGAVQVDDCDQIMMITDAGTLVRTRVSEISVVGRNTQGVILIRTAEDENVVGLQRVA |
| SRR1157587 | GSVVGAVQVDDCDQIMMITDAGTLVRTRVSEISVVGRNTQGVILIRTAEDENVVGLQRVA |
| SRR3027706 | GSVVGAVQVDDCDQIMMITDAGTLVRTRVSEISVVGRNTQGVILIRTAEDENVVGLQRVA |
| SRR3027707 | GSVVGAVQVDDCDQIMMITDAGTLVRTRVSEISVVGRNTQGVILIRTAEDENVVGLQRVA |
| SRR3027708 | GSVVGAVQVDDCDQIMMITDAGTLVRTRVSEISVVGRNTQGVILIRTAEDENVVGLQRVA |
| SRR3027710 | GSVVGAVQVDDCDQIMMITDAGTLVRTRVSEISVVGRNTQGVILIRTAEDENVVGLQRVA |
| SRR3027711 | GSVVGAVQVDDCDQIMMITDAGTLVRTRVSEISVVGRNTQGVILIRTAEDENVVGLQRVA |
| SRR3027716 | GSVVGAVQVDDCDQIMMITDAGTLVRTRVSEISVVGRNTQGVILIRTAEDENVVGLQRVA |
| SRR3027717 | GSVVGAVQVDDCDQIMMITDAGTLVRTRVSEISVVGRNTQGVILIRTAEDENVVGLQRVA |
| SRR3027719 | GSVVGAVQVDDCDQIMMITDAGTLVRTRVSEISVVGRNTQGVILIRTAEDENVVGLQRVA |
| SRR3027721 | GSVVGAVQVDDCDQIMMITDAGTLVRTRVSEISVVGRNTQGVILIRTAEDENVVGLQRVA |
| SRR3027723 | GSVVGAVQVDDCDQIMMITDAGTLVRTRVSEISVVGRNTQGVILIRTAEDENVVGLQRVA |
| SRR3115978 | GSVVGAVQVDDCDQIMMITDAGTLVRTRVSEISVVGRNTQGVILIRTAEDENVVGLQRVA |
| SRR2534093 | GSVVGAVQVDDCDQIMMITDAGTLVRTRVSEISVVGRNTQGVILIRTAEDENVVGLQRVA |
| SRR2534094 | GSVVGAVQVDDCDQIMMITDAGTLVRTRVSEISVVGRNTQGVILIRTAEDENVVGLQRVA |
| SRR2534095 | GSVVGAVQVDDCDQIMMITDAGTLVRTRVSEISVVGRNTQGVILIRTAEDENVVGLQRVA |
| SRR2534108 | GSVVGAVQVDDCDQIMMITDAGTLVRTRVSEISVVGRNTQGVILIRTAEDENVVGLQRVA |
| SRR1106464 | GSVVGAVQVDDCDQIMMITDAGTLVRTRVSEISVVGRNTQGVILIRTAEDENVVGLQRVA |
| SRR1106463 | GSVVGAVQVDDCDQIMMITDAGTLVRTRVSEISVVGRNTQGVILIRTAEDENVVGLQRVA |
| SRR6949610 | GSVVGAVQVDDCDQIMMITDAGTLVRTRVSEISVVGRNTQGVILIRTAEDENVVGLQRVA |
| SRR6950452 | GSVVGAVQVDDCDQIMMITDAGTLVRTRVSEISVVGRNTQGVILIRTAEDENVVGLQRVA |
| ERR2019831 | GSVVGAVQVDDCDQIMMITDAGTLVRTRVSEISVVGRNTQGVILIRTAEDENVVGLQRVA |
| SRR2085693 | GSVVGAVQVDDCDQIMMITDAGTLVRTRVSEISVVGRNTQGVILIRTAEDENVVGLQRVA |
| SRR2086898 | GSVVGAVQVDDCDQIMMITDAGTLVRTRVSEISVVGRNTQGVILIRTAEDENVVGLQRVA |
| SRR2175312 | GSVVGAVQVDDCDQIMMITDAGTLVRTRVSEISVVGRNTQGVILIRTAEDENVVGLQRVA |
| SRR2175360 | GSVVGAVQVDDCDQIMMITDAGTLVRTRVSEISVVGRNTQGVILIRTAEDENVVGLQRVA |
| SRR5231997 | GSVVGAVQVDDCDQIMMITDAGTLVRTRVSEISVVGRNTQGVILIRTAEDENVVGLQRVA |
| SRR5232003 | GSVVGAVQVDDCDQIMMITDAGTLVRTRVSEISVVGRNTQGVILIRTAEDENVVGLQRVA |
| SRR5232015 | GSVVGAVQVDDCDQIMMITDAGTLVRTRVSEISVVGRNTQGVILIRTAEDENVVGLQRVA |
| SRR949434 | GSVVGAVQVDDCDQIMMITDAGTLVRTRVSEISVVGRNTQGVILIRTAEDENVVGLQRVA |
| SRR3216575 | GSVVGAVQVDDCDQIMMITDAGTLVRTRVSEISVVGRNTQGVILIRTAEDENVVGLQRVA |
| SRR5205342 | GSVVGAVQVDDCDQIMMITDAGTLVRTRVSEISVVGRNTQGVILIRTAEDENVVGLQRVA |
| SRR1501669 | GSVVGAVQVDDCDQIMMITDAGTLVRTRVSEISVVGRNTQGVILIRTAEDENVVGLQRVA |
| SRR5209740 | GSVVGAVQVDDCDQIMMITDAGTLVRTRVSEISVVGRNTQGVILIRTAEDENVVGLQRVA |
| SRR3240355 | GSVVGAVQVDDCDQIMMITDAGTLVRTRVSEISVVGRNTQGVILIRTAEDENVVGLQRVA |
| SRR3392777 | GSVVGAVQVDDCDQIMMITDAGTLVRTRVSEISVVGRNTQGVILIRTAEDENVVGLQRVA |
| SRR3593671 | GSVVGAVQVDDCDQIMMITDAGTLVRTRVSEISVVGRNTQGVILIRTAEDENVVGLQRVA |
| SRR5413290 | GSVVGAVQVDDCDQIMMITDAGTLVRTRVSEISVVGRNTQGVILIRTAEDENVVGLQRVA |
| SRR5590269 | GSVVGAVQVDDCDQIMMITDAGTLVRTRVSEISVVGRNTQGVILIRTAEDENVVGLQRVA |
| SRR5812103 | GSVVGAVQVDDCDQIMMITDAGTLVRTRVSEISVVGRNTQGVILIRTAEDENVVGLQRVA |
| SRR2830941 | GSVVGAVQVDDCDQIMMITDAGTLVRTRVSEISVVGRNTQGVILIRTAEDENVVGLQRVA |
| SRR2830966 | GSVVGAVQVDDCDQIMMITDAGTLVRTRVSEISVVGRNTQGVILIRTAEDENVVGLQRVA |
| SRR3137270 | GSVVGAVQVDDCDQIMMITDAGTLVRTRVSEISVVGRNTQGVILIRTAEDENVVGLQRVA |
| SRR3137271 | GSVVGAVQVDDCDQIMMITDAGTLVRTRVSEISVVGRNTQGVILIRTAEDENVVGLQRVA |
| ERR526807 | GSVVGAVQVDDCDQIMMITDAGTLVRTRVSEISVVGRNTQGVILIRTAEDENVVGLQRVA |
| ERR2197922 | GSVVGAVQVDDCDQIMMITDAGTLVRTRVSEISVVGRNTQGVILIRTAEDENVVGLQRVA |
| ERR2197923 | GSVVGAVQVDDCDQIMMITDAGTLVRTRVSEISVVGRNTQGVILIRTAEDENVVGLQRVA |
| ERR2197924 | GSVVGAVQVDDCDQIMMITDAGTLVRTRVSEISVVGRNTQGVILIRTAEDENVVGLQRVA |
| ERR2197925 | GSVVGAVQVDDCDQIMMITDAGTLVRTRVSEISVVGRNTQGVILIRTAEDENVVGLQRVA |
| ERR2197927 | GSVVGAVQVDDCDQIMMITDAGTLVRTRVSEISVVGRNTQGVILIRTAEDENVVGLQRVA |
| ERR2197929 | GSVVGAVQVDDCDQIMMITDAGTLVRTRVSEISVVGRNTQGVILIRTAEDENVVGLQRVA |
| SRR1648149 | GSVVGAVQVDDCDQIMMITDAGTLVRTRVSEISVVGRNTQGVILIRTAEDENVVGLQRVA |
| SRR1048299 | GSVVGAVQVDDCDQIMMITDAGTLVRTRVSEISVVGRNTQGVILIRTAEDENVVGLQRVA |
| SRR1300677 | GSVVGAVQVDDCDQIMMITDAGTLVRTRVSEISVVGRNTQGVILIRTAEDENVVGLQRVA |
| SRR1288356 | GSVVGAVQVDDCDQIMMITDAGTLVRTRVSEISVVGRNTQGVILIRTAEDENVVGLQRVA |
| SRR7426190 | GSVVGAVQVDDCDQIMMITDAGTLVRTRVSEISVVGRNTQGVILIRTAEDENVVGLQRVA |
| SRR7426192 | GSVVGAVQVDDCDQIMMITDAGTLVRTRVSEISVVGRNTQGVILIRTAEDENVVGLQRVA |
| SRR7426193 | GSVVGAVQVDDCDQIMMITDAGTLVRTRVSEISVVGRNTQGVILIRTAEDENVVGLQRVA |
| SRR7441832 | GSVVGAVQVDDCDQIMMITDAGTLVRTRVSEISVVGRNTQGVILIRTAEDENVVGLQRVA |
| SRR7426179 | GSVVGAVQVDDCDQIMMITDAGTLVRTRVSEISVVGRNTQGVILIRTAEDENVVGLQRVA |
| SRR7439238 | GSVVGAVQVDDCDQIMMITDAGTLVRTRVSEISVVGRNTQGVILIRTAEDENVVGLQRVA |
| SRR7439244 | GSVVGAVQVDDCDQIMMITDAGTLVRTRVSEISVVGRNTQGVILIRTAEDENVVGLQRVA |
| SRR7439259 | GSVVGAVQVDDCDQIMMITDAGTLVRTRVSEISVVGRNTQGVILIRTAEDENVVGLQRVA |
| SRR7439260 | GSVVGAVQVDDCDQIMMITDAGTLVRTRVSEISVVGRNTQGVILIRTAEDENVVGLQRVA |
| SRR7441786 | GSVVGAVQVDDCDQIMMITDAGTLVRTRVSEISVVGRNTQGVILIRTAEDENVVGLQRVA |
| SRR7441797 | GSVVGAVQVDDCDQIMMITDAGTLVRTRVSEISVVGRNTQGVILIRTAEDENVVGLQRVA |
| ERR1759093 | GSVVGAVQVDDCDQIMMITDAGTLVRTRVSEISVVGRNTQGVILIRTAEDENVVGLQRVA |
| ERR2580275 | GSVVGAVQVDDCDQIMMITDAGTLVRTRVSEISVVGRNTQGVILIRTAEDENVVGLQRVA |
| ERR1759204 | GSVVGAVQVDDCDQIMMITDAGTLVRTRVSEISVVGRNTQGVILIRTAEDENVVGLQRVA |
| SRR1300699 | GSVVGAVQVDDCDQIMMITDAGTLVRTRVSEISVVGRNTQGVILIRTAEDENVVGLQRVA |
| S_0825_17 | GSVVGAVQVDDCDQIMMITDAGTLVRTRVSEISVVGRNTQGVILIRTAEDENVVGLQRVA |
| SRR1958215 | GSVVGAVQVDDCDQIMMITDAGTLVRTRVSEISVVGRNTQGVILIRTAEDENVVGLQRVA |
| SRR1958540 | GSVVGAVQVDDCDQIMMITDAGTLVRTRVSEISVVGRNTQGVILIRTAEDENVVGLQRVA |
| SRR1958636 | GSVVGAVQVDDCDQIMMITDAGTLVRTRVSEISVVGRNTQGVILIRTAEDENVVGLQRVA |
| SRR1959422 | GSVVGAVQVDDCDQIMMITDAGTLVRTRVSEISVVGRNTQGVILIRTAEDENVVGLQRVA |
| SRR1959427 | GSVVGAVQVDDCDQIMMITDAGTLVRTRVSEISVVGRNTQGVILIRTAEDENVVGLQRVA |
| SRR1960226 | GSVVGAVQVDDCDQIMMITDAGTLVRTRVSEISVVGRNTQGVILIRTAEDENVVGLQRVA |
| SRR1963498 | GSVVGAVQVDDCDQIMMITDAGTLVRTRVSEISVVGRNTQGVILIRTAEDENVVGLQRVA |
| SRR1965947 | GSVVGAVQVDDCDQIMMITDAGTLVRTRVSEISVVGRNTQGVILIRTAEDENVVGLQRVA |
| SRR1966125 | GSVVGAVQVDDCDQIMMITDAGTLVRTRVSEISVVGRNTQGVILIRTAEDENVVGLQRVA |
| SRR1966330 | GSVVGAVQVDDCDQIMMITDAGTLVRTRVSEISVVGRNTQGVILIRTAEDENVVGLQRVA |
| SRR1966565 | GSVVGAVQVDDCDQIMMITDAGTLVRTRVSEISVVGRNTQGVILIRTAEDENVVGLQRVA |
| SRR1966864 | GSVVGAVQVDDCDQIMMITDAGTLVRTRVSEISVVGRNTQGVILIRTAEDENVVGLQRVA |
| SRR1966989 | GSVVGAVQVDDCDQIMMITDAGTLVRTRVSEISVVGRNTQGVILIRTAEDENVVGLQRVA |
| SRR1967688 | GSVVGAVQVDDCDQIMMITDAGTLVRTRVSEISVVGRNTQGVILIRTAEDENVVGLQRVA |
| SRR1967733 | GSVVGAVQVDDCDQIMMITDAGTLVRTRVSEISVVGRNTQGVILIRTAEDENVVGLQRVA |
| SRR1967746 | GSVVGAVQVDDCDQIMMITDAGTLVRTRVSEISVVGRNTQGVILIRTAEDENVVGLQRVA |
| SRR1968341 | GSVVGAVQVDDCDQIMMITDAGTLVRTRVSEISVVGRNTQGVILIRTAEDENVVGLQRVA |
| SRR1968456 | GSVVGAVQVDDCDQIMMITDAGTLVRTRVSEISVVGRNTQGVILIRTAEDENVVGLQRVA |
| SRR1968465 | GSVVGAVQVDDCDQIMMITDAGTLVRTRVSEISVVGRNTQGVILIRTAEDENVVGLQRVA |
| SRR1968761 | GSVVGAVQVDDCDQIMMITDAGTLVRTRVSEISVVGRNTQGVILIRTAEDENVVGLQRVA |
| SRR1969047 | GSVVGAVQVDDCDQIMMITDAGTLVRTRVSEISVVGRNTQGVILIRTAEDENVVGLQRVA |
| SRR1969255 | GSVVGAVQVDDCDQIMMITDAGTLVRTRVSEISVVGRNTQGVILIRTAEDENVVGLQRVA |
| SRR1969412 | GSVVGAVQVDDCDQIMMITDAGTLVRTRVSEISVVGRNTQGVILIRTAEDENVVGLQRVA |
| SRR1969524 | GSVVGAVQVDDCDQIMMITDAGTLVRTRVSEISVVGRNTQGVILIRTAEDENVVGLQRVA |
| SRR1969584 | GSVVGAVQVDDCDQIMMITDAGTLVRTRVSEISVVGRNTQGVILIRTAEDENVVGLQRVA |
| SRR1969648 | GSVVGAVQVDDCDQIMMITDAGTLVRTRVSEISVVGRNTQGVILIRTAEDENVVGLQRVA |
| SRR1969804 | GSVVGAVQVDDCDQIMMITDAGTLVRTRVSEISVVGRNTQGVILIRTAEDENVVGLQRVA |
| SRR1970221 | GSVVGAVQVDDCDQIMMITDAGTLVRTRVSEISVVGRNTQGVILIRTAEDENVVGLQRVA |
| SRR1970268 | GSVVGAVQVDDCDQIMMITDAGTLVRTRVSEISVVGRNTQGVILIRTAEDENVVGLQRVA |
| SRR1965862 | GSVVGAVQVDDCDQIMMITDAGTLVRTRVSEISVVGRNTQGVILIRTAEDENVVGLQRVA |
| SRR1967363 | GSVVGAVQVDDCDQIMMITDAGTLVRTRVSEISVVGRNTQGVILIRTAEDENVVGLQRVA |
| SRR1968276 | GSVVGAVQVDDCDQIMMITDAGTLVRTRVSEISVVGRNTQGVILIRTAEDENVVGLQRVA |
| SRR1968967 | GSVVGAVQVDDCDQIMMITDAGTLVRTRVSEISVVGRNTQGVILIRTAEDENVVGLQRVA |
| SRR3321531 | GSVVGAVQVDDCDQIMMITDAGTLVRTRVSEISVVGRNTQGVILIRTAEDENVVGLQRVA |
| SRR3321883 | GSVVGAVQVDDCDQIMMITDAGTLVRTRVSEISVVGRNTQGVILIRTAEDENVVGLQRVA |
| SRR3322413 | GSVVGAVQVDDCDQIMMITDAGTLVRTRVSEISVVGRNTQGVILIRTAEDENVVGLQRVA |
| SRR3323012 | GSVVGAVQVDDCDQIMMITDAGTLVRTRVSEISVVGRNTQGVILIRTAEDENVVGLQRVA |
| SRR5194289 | GSVVGAVQVDDCDQIMMITDAGTLVRTRVSEISVVGRNTQGVILIRTAEDENVVGLQRVA |
| SRR7163798 | GSVVGAVQVDDCDQIMMITDAGTLVRTRVSEISVVGRNTQGVILIRTAEDENVVGLQRVA |
| SRR7172610 | GSVVGAVQVDDCDQIMMITDAGTLVRTRVSEISVVGRNTQGVILIRTAEDENVVGLQRVA |
| SRR7204568 | GSVVGAVQVDDCDQIMMITDAGTLVRTRVSEISVVGRNTQGVILIRTAEDENVVGLQRVA |
| SRR7223230 | GSVVGAVQVDDCDQIMMITDAGTLVRTRVSEISVVGRNTQGVILIRTAEDENVVGLQRVA |
| SRR7230675 | GSVVGAVQVDDCDQIMMITDAGTLVRTRVSEISVVGRNTQGVILIRTAEDENVVGLQRVA |
| SRR7278056 | GSVVGAVQVDDCDQIMMITDAGTLVRTRVSEISVVGRNTQGVILIRTAEDENVVGLQRVA |
| SRR7278086 | GSVVGAVQVDDCDQIMMITDAGTLVRTRVSEISVVGRNTQGVILIRTAEDENVVGLQRVA |
| SRR7285841 | GSVVGAVQVDDCDQIMMITDAGTLVRTRVSEISVVGRNTQGVILIRTAEDENVVGLQRVA |
| SRR7292625 | GSVVGAVQVDDCDQIMMITDAGTLVRTRVSEISVVGRNTQGVILIRTAEDENVVGLQRVA |
| SRR7292665 | GSVVGAVQVDDCDQIMMITDAGTLVRTRVSEISVVGRNTQGVILIRTAEDENVVGLQRVA |
| SRR7297965 | GSVVGAVQVDDCDQIMMITDAGTLVRTRVSEISVVGRNTQGVILIRTAEDENVVGLQRVA |
| SRR7350726 | GSVVGAVQVDDCDQIMMITDAGTLVRTRVSEISVVGRNTQGVILIRTAEDENVVGLQRVA |
| SRR7410328 | GSVVGAVQVDDCDQIMMITDAGTLVRTRVSEISVVGRNTQGVILIRTAEDENVVGLQRVA |
| SRR7474665 | GSVVGAVQVDDCDQIMMITDAGTLVRTRVSEISVVGRNTQGVILIRTAEDENVVGLQRVA |
| SRR7523184 | GSVVGAVQVDDCDQIMMITDAGTLVRTRVSEISVVGRNTQGVILIRTAEDENVVGLQRVA |
| SRR7187264 | GSVVGAVQVDDCDQIMMITDAGTLVRTRVSEISVVGRNTQGVILIRTAEDENVVGLQRVA |
| SRR7204445 | GSVVGAVQVDDCDQIMMITDAGTLVRTRVSEISVVGRNTQGVILIRTAEDENVVGLQRVA |
| SRR7285641 | GSVVGAVQVDDCDQIMMITDAGTLVRTRVSEISVVGRNTQGVILIRTAEDENVVGLQRVA |
| SRR7286695 | GSVVGAVQVDDCDQIMMITDAGTLVRTRVSEISVVGRNTQGVILIRTAEDENVVGLQRVA |
| SRR7286705 | GSVVGAVQVDDCDQIMMITDAGTLVRTRVSEISVVGRNTQGVILIRTAEDENVVGLQRVA |
| SRR7292931 | GSVVGAVQVDDCDQIMMITDAGTLVRTRVSEISVVGRNTQGVILIRTAEDENVVGLQRVA |
| SRR7310349 | GSVVGAVQVDDCDQIMMITDAGTLVRTRVSEISVVGRNTQGVILIRTAEDENVVGLQRVA |
| SRR7351616 | GSVVGAVQVDDCDQIMMITDAGTLVRTRVSEISVVGRNTQGVILIRTAEDENVVGLQRVA |
| SRR7414818 | GSVVGAVQVDDCDQIMMITDAGTLVRTRVSEISVVGRNTQGVILIRTAEDENVVGLQRVA |
| SRR7426480 | GSVVGAVQVDDCDQIMMITDAGTLVRTRVSEISVVGRNTQGVILIRTAEDENVVGLQRVA |
| SRR5584105 | GSVVGAVQVDDCDQIMMITDAGTLVRTRVSEISVVGRNTQGVILIRTAEDENVVGLQRVA |
| SRR5584565 | GSVVGAVQVDDCDQIMMITDAGTLVRTRVSEISVVGRNTQGVILIRTAEDENVVGLQRVA |
| SRR5584614 | GSVVGAVQVDDCDQIMMITDAGTLVRTRVSEISVVGRNTQGVILIRTAEDENVVGLQRVA |
| SRR5631543 | GSVVGAVQVDDCDQIMMITDAGTLVRTRVSEISVVGRNTQGVILIRTAEDENVVGLQRVA |
| SRR5631553 | GSVVGAVQVDDCDQIMMITDAGTLVRTRVSEISVVGRNTQGVILIRTAEDENVVGLQRVA |
| SRR7123196 | GSVVGAVQVDDCDQIMMITDAGTLVRTRVSEISVVGRNTQGVILIRTAEDENVVGLQRVA |
| SRR7163819 | GSVVGAVQVDDCDQIMMITDAGTLVRTRVSEISVVGRNTQGVILIRTAEDENVVGLQRVA |
| SRR7163920 | GSVVGAVQVDDCDQIMMITDAGTLVRTRVSEISVVGRNTQGVILIRTAEDENVVGLQRVA |
| SRR7209528 | GSVVGAVQVDDCDQIMMITDAGTLVRTRVSEISVVGRNTQGVILIRTAEDENVVGLQRVA |
| SRR7249868 | GSVVGAVQVDDCDQIMMITDAGTLVRTRVSEISVVGRNTQGVILIRTAEDENVVGLQRVA |
| SRR7278088 | GSVVGAVQVDDCDQIMMITDAGTLVRTRVSEISVVGRNTQGVILIRTAEDENVVGLQRVA |
| SRR7285788 | GSVVGAVQVDDCDQIMMITDAGTLVRTRVSEISVVGRNTQGVILIRTAEDENVVGLQRVA |
| SRR7286789 | GSVVGAVQVDDCDQIMMITDAGTLVRTRVSEISVVGRNTQGVILIRTAEDENVVGLQRVA |
| SRR7286886 | GSVVGAVQVDDCDQIMMITDAGTLVRTRVSEISVVGRNTQGVILIRTAEDENVVGLQRVA |
| SRR7310632 | GSVVGAVQVDDCDQIMMITDAGTLVRTRVSEISVVGRNTQGVILIRTAEDENVVGLQRVA |
| SRR7350631 | GSVVGAVQVDDCDQIMMITDAGTLVRTRVSEISVVGRNTQGVILIRTAEDENVVGLQRVA |
| SRR7458741 | GSVVGAVQVDDCDQIMMITDAGTLVRTRVSEISVVGRNTQGVILIRTAEDENVVGLQRVA |
| SRR7480280 | GSVVGAVQVDDCDQIMMITDAGTLVRTRVSEISVVGRNTQGVILIRTAEDENVVGLQRVA |
| SRR7523660 | GSVVGAVQVDDCDQIMMITDAGTLVRTRVSEISVVGRNTQGVILIRTAEDENVVGLQRVA |
| SRR7523775 | GSVVGAVQVDDCDQIMMITDAGTLVRTRVSEISVVGRNTQGVILIRTAEDENVVGLQRVA |
| SRR7251101 | GSVVGAVQVDDCDQIMMITDAGTLVRTRVSEISVVGRNTQGVILIRTAEDENVVGLQRVA |
| SRR7284299 | GSVVGAVQVDDCDQIMMITDAGTLVRTRVSEISVVGRNTQGVILIRTAEDENVVGLQRVA |
| SRR7285738 | GSVVGAVQVDDCDQIMMITDAGTLVRTRVSEISVVGRNTQGVILIRTAEDENVVGLQRVA |
| SRR7310640 | GSVVGAVQVDDCDQIMMITDAGTLVRTRVSEISVVGRNTQGVILIRTAEDENVVGLQRVA |
| SRR7349159 | GSVVGAVQVDDCDQIMMITDAGTLVRTRVSEISVVGRNTQGVILIRTAEDENVVGLQRVA |
| SRR7474873 | GSVVGAVQVDDCDQIMMITDAGTLVRTRVSEISVVGRNTQGVILIRTAEDENVVGLQRVA |
| SRR7495689 | GSVVGAVQVDDCDQIMMITDAGTLVRTRVSEISVVGRNTQGVILIRTAEDENVVGLQRVA |
| SRR7495752 | GSVVGAVQVDDCDQIMMITDAGTLVRTRVSEISVVGRNTQGVILIRTAEDENVVGLQRVA |
| ----------------------------------------------------------------------------- | |
| S16BD08730 | EPVDDEELDAIDGSVAEGDEDIAPEAESDDDVADDADE* |
| S18BD00684 | EPVDDEELDAIDGSVAEGDEDIAPEAESDDDVADDADE* |
| S18BD03994 | EPVDDEELDAIDGSVAEGDEDIAPEAESDDDVADDADE* |
| S18BD05011 | EPVDDEELDAIDGSVAEGDEDIAPEAESDDDVADDADE* |
| RKI_16-03723 | EPVDDEELDAIDGSVAEGDEDIAPEAESDDDVADDADE* |
| RKI_16-04315 | EPVDDEELDAIDGSVAEGDEDIAPEAESDDDVADDADE* |
| RKI_17-02304 | EPVDDEELDAIDGSVAEGDEDIAPEAESDDDVADDADE* |
| RKI_17-02411 | EPVDDEELDAIDGSVAEGDEDIAPEAESDDDVADDADE* |
| RKI_17-02757 | EPVDDEELDAIDGSVAEGDEDIAPEAESDDDVADDADE* |
| RKI_17-04797 | EPVDDEELDAIDGSVAEGDEDIAPEAESDDDVADDADE* |
| RKI_17-06869 | EPVDDEELDAIDGSVAEGDEDIAPEAESDDDVADDADE* |
| ERR2580277 | EPVDDEELDAIDGSVAEGDEDIAPEAESDDDVADDADE* |
| ERR2580276 | EPVDDEELDAIDGSVAEGDEDIAPEAESDDDVADDADE* |
| ERR2580273 | EPVDDEELDAIDGSVAEGDEDIAPEAESDDDVADDADE* |
| ERR2580274 | EPVDDEELDAIDGSVAEGDEDIAPEAESDDDVADDADE* |
| ERR2173656 | EPVDDEELDAIDGSVAEGDEDIAPEAESDDDVADDADE* |
| 17041676 | EPVDDEELDAIDGSVAEGDEDIAPEAESDDDVADDADE* |
| MT16-000061 | EPVDDEELDAIDGSVAEGDEDIAPEAESDDDVADDADE* |
| MT16-019416 | EPVDDEELDAIDGSVAEGDEDIAPEAESDDDVADDADE* |
| MT16-027865 | EPVDDEELDAIDGSVAEGDEDIAPEAESDDDVADDADE* |
| MT16-031693 | EPVDDEELDAIDGSVAEGDEDIAPEAESDDDVADDADE* |
| MT16-040253 | EPVDDEELDAIDGSVAEGDEDIAPEAESDDDVADDADE* |
| MT16-045379 | EPVDDEELDAIDGSVAEGDEDIAPEAESDDDVADDADE* |
| MT16-442728 | EPVDDEELDAIDGSVAEGDEDIAPEAESDDDVADDADE* |
| MT16-462857 | EPVDDEELDAIDGSVAEGDEDIAPEAESDDDVADDADE* |
| MT16-480196 | EPVDDEELDAIDGSVAEGDEDIAPEAESDDDVADDADE* |
| MT16-861555 | EPVDDEELDAIDGSVAEGDEDIAPEAESDDDVADDADE* |
| MT17-076833 | EPVDDEELDAIDGSVAEGDEDIAPEAESDDDVADDADE* |
| MT17-110677 | EPVDDEELDAIDGSVAEGDEDIAPEAESDDDVADDADE* |
| MT17-131730 | EPVDDEELDAIDGSVAEGDEDIAPEAESDDDVADDADE* |
| MT17-140890 | EPVDDEELDAIDGSVAEGDEDIAPEAESDDDVADDADE* |
| MT17-141840 | EPVDDEELDAIDGSVAEGDEDIAPEAESDDDVADDADE* |
| MT17-152488 | EPVDDEELDAIDGSVAEGDEDIAPEAESDDDVADDADE* |
| MT17-157311 | EPVDDEELDAIDGSVAEGDEDIAPEAESDDDVADDADE* |
| MT17-161645 | EPVDDEELDAIDGSVAEGDEDIAPEAESDDDVADDADE* |
| MT17-167951 | EPVDDEELDAIDGSVAEGDEDIAPEAESDDDVADDADE* |
| MT18-217732 | EPVDDEELDAIDGSVAEGDEDIAPEAESDDDVADDADE* |
| MT18-252580 | EPVDDEELDAIDGSVAEGDEDIAPEAESDDDVADDADE* |
| RIVM_H_2009-01 | EPVDDEELDAIDGSVAEGDEDIAPEAESDDDVADDADE* |
| RIVM_H_2010-01 | EPVDDEELDAIDGSVAEGDEDIAPEAESDDDVADDADE* |
| RIVM_H_2010-02 | EPVDDEELDAIDGSVAEGDEDIAPEAESDDDVADDADE* |
| RIVM_H_2011-01 | EPVDDEELDAIDGSVAEGDEDIAPEAESDDDVADDADE* |
| RIVM_H_2011-02 | EPVDDEELDAIDGSVAEGDEDIAPEAESDDDVADDADE* |
| RIVM_H_2011-03 | EPVDDEELDAIDGSVAEGDEDIAPEAESDDDVADDADE* |
| RIVM_H_2013-01 | EPVDDEELDAIDGSVAEGDEDIAPEAESDDDVADDADE* |
| RIVM_H_2013-02 | EPVDDEELDAIDGSVAEGDEDIAPEAESDDDVADDADE* |
| RIVM_H_2014-01 | EPVDDEELDAIDGSVAEGDEDIAPEAESDDDVADDADE* |
| RIVM_H_2014-02 | EPVDDEELDAIDGSVAEGDEDIAPEAESDDDVADDADE* |
| RIVM_H_2016-01 | EPVDDEELDAIDGSVAEGDEDIAPEAESDDDVADDADE* |
| RIVM_H_2016-02 | EPVDDEELDAIDGSVAEGDEDIAPEAESDDDVADDADE* |
| RIVM_H_2016-03 | EPVDDEELDAIDGSVAEGDEDIAPEAESDDDVADDADE* |
| RIVM_H_2016-04 | EPVDDEELDAIDGSVAEGDEDIAPEAESDDDVADDADE* |
| RIVM_H_2016-05 | EPVDDEELDAIDGSVAEGDEDIAPEAESDDDVADDADE* |
| RIVM_H_2016-06 | EPVDDEELDAIDGSVAEGDEDIAPEAESDDDVADDADE* |
| RIVM_H_2016-07 | EPVDDEELDAIDGSVAEGDEDIAPEAESDDDVADDADE* |
| RIVM_H_2016-08 | EPVDDEELDAIDGSVAEGDEDIAPEAESDDDVADDADE* |
| RIVM_H_2016-09 | EPVDDEELDAIDGSVAEGDEDIAPEAESDDDVADDADE* |
| RIVM_H_2016-10 | EPVDDEELDAIDGSVAEGDEDIAPEAESDDDVADDADE* |
| RIVM_H_2016-11 | EPVDDEELDAIDGSVAEGDEDIAPEAESDDDVADDADE* |
| RIVM_H_2016-12 | EPVDDEELDAIDGSVAEGDEDIAPEAESDDDVADDADE* |
| RIVM_H_2016-13 | EPVDDEELDAIDGSVAEGDEDIAPEAESDDDVADDADE* |
| RIVM_H_2016-14 | EPVDDEELDAIDGSVAEGDEDIAPEAESDDDVADDADE* |
| RIVM_H_2016-15 | EPVDDEELDAIDGSVAEGDEDIAPEAESDDDVADDADE* |
| RIVM_H_2017-01 | EPVDDEELDAIDGSVAEGDEDIAPEAESDDDVADDADE* |
| RIVM_H_2017-02 | EPVDDEELDAIDGSVAEGDEDIAPEAESDDDVADDADE* |
| RIVM_H_2017-03 | EPVDDEELDAIDGSVAEGDEDIAPEAESDDDVADDADE* |
| RIVM_H_2017-04 | EPVDDEELDAIDGSVAEGDEDIAPEAESDDDVADDADE* |
| RIVM_H_2017-05 | EPVDDEELDAIDGSVAEGDEDIAPEAESDDDVADDADE* |
| RIVM_H_2017-06 | EPVDDEELDAIDGSVAEGDEDIAPEAESDDDVADDADE* |
| RIVM_H_2017-07 | EPVDDEELDAIDGSVAEGDEDIAPEAESDDDVADDADE* |
| RIVM_H_2017-08 | EPVDDEELDAIDGSVAEGDEDIAPEAESDDDVADDADE* |
| RIVM_H_2017-09 | EPVDDEELDAIDGSVAEGDEDIAPEAESDDDVADDADE* |
| RIVM_H_2017-10 | EPVDDEELDAIDGSVAEGDEDIAPEAESDDDVADDADE* |
| RIVM_H_2017-11 | EPVDDEELDAIDGSVAEGDEDIAPEAESDDDVADDADE* |
| RIVM_H_2017-12 | EPVDDEELDAIDGSVAEGDEDIAPEAESDDDVADDADE* |
| RIVM_H_2017-13 | EPVDDEELDAIDGSVAEGDEDIAPEAESDDDVADDADE* |
| RIVM_H_2017-14 | EPVDDEELDAIDGSVAEGDEDIAPEAESDDDVADDADE* |
| RIVM_H_2017-15 | EPVDDEELDAIDGSVAEGDEDIAPEAESDDDVADDADE* |
| RIVM_H_2017-16 | EPVDDEELDAIDGSVAEGDEDIAPEAESDDDVADDADE* |
| RIVM_H_2017-17 | EPVDDEELDAIDGSVAEGDEDIAPEAESDDDVADDADE* |
| RIVM_H_2017-18 | EPVDDEELDAIDGSVAEGDEDIAPEAESDDDVADDADE* |
| RIVM_H_2017-19 | EPVDDEELDAIDGSVAEGDEDIAPEAESDDDVADDADE* |
| 15EP001483 | EPVDDEELDAIDGSVAEGDEDIAPEAESDDDVADDADE* |
| 17EP002363 | EPVDDEELDAIDGSVAEGDEDIAPEAESDDDVADDADE* |
| S_0812_17 | EPVDDEELDAIDGSVAEGDEDIAPEAESDDDVADDADE* |
| SRR1957844 | EPVDDEELDAIDGSVAEGDEDIAPEAESDDDVADDADE* |
| SRR1958654 | EPVDDEELDAIDGSVAEGDEDIAPEAESDDDVADDADE* |
| SRR1965077 | EPVDDEELDAIDGSVAEGDEDIAPEAESDDDVADDADE* |
| SRR1966369 | EPVDDEELDAIDGSVAEGDEDIAPEAESDDDVADDADE* |
| SRR1967117 | EPVDDEELDAIDGSVAEGDEDIAPEAESDDDVADDADE* |
| SRR1967922 | EPVDDEELDAIDGSVAEGDEDIAPEAESDDDVADDADE* |
| SRR8704720 | EPVDDEELDAIDGSVAEGDEDIAPEAESDDDVADDADE* |
| SRR7216071 | EPVDDEELDAIDGSVAEGDEDIAPEAESDDDVADDADE* |
| SRR7349175 | EPVDDEELDAIDGSVAEGDEDIAPEAESDDDVADDADE* |
| SRR7523148 | EPVDDEELDAIDGSVAEGDEDIAPEAESDDDVADDADE* |
| SRR7523854 | EPVDDEELDAIDGSVAEGDEDIAPEAESDDDVADDADE* |
| 313865 | EPVDDEELDAIDGSVAEGDEDIAPEAESDDDVADDADE* |
| SRR7277793 | EPVDDEELDAIDGSVAEGDEDIAPEAESDDDVADDADE* |
| SRR7343877 | EPVDDEELDAIDGSVAEGDEDIAPEAESDDDVADDADE* |
| SRR7351477 | EPVDDEELDAIDGSVAEGDEDIAPEAESDDDVADDADE* |
| SRR5583183 | EPVDDEELDAIDGSVAEGDEDIAPEAESDDDVADDADE* |
| SRR5585240 | EPVDDEELDAIDGSVAEGDEDIAPEAESDDDVADDADE* |
| SRR7284317 | EPVDDEELDAIDGSVAEGDEDIAPEAESDDDVADDADE* |
| SRR7299161 | EPVDDEELDAIDGSVAEGDEDIAPEAESDDDVADDADE* |
| SRR7401730 | EPVDDEELDAIDGSVAEGDEDIAPEAESDDDVADDADE* |
| SRR7469092 | EPVDDEELDAIDGSVAEGDEDIAPEAESDDDVADDADE* |
| SRR7879556 | EPVDDEELDAIDGSVAEGDEDIAPEAESDDDVADDADE* |
| SRR8526100 | EPVDDEELDAIDGSVAEGDEDIAPEAESDDDVADDADE* |
| SRR8553991 | EPVDDEELDAIDGSVAEGDEDIAPEAESDDDVADDADE* |
| SRR7842487 | EPVDDEELDAIDGSVAEGDEDIAPEAESDDDVADDADE* |
| SRR8054524 | EPVDDEELDAIDGSVAEGDEDIAPEAESDDDVADDADE* |
| SRR8054525 | EPVDDEELDAIDGSVAEGDEDIAPEAESDDDVADDADE* |
| SRR8524733 | EPVDDEELDAIDGSVAEGDEDIAPEAESDDDVADDADE* |
| SRR4093291 | EPVDDEELDAIDGSVAEGDEDIAPEAESDDDVADDADE* |
| SRR4245549 | EPVDDEELDAIDGSVAEGDEDIAPEAESDDDVADDADE* |
| SRR3057154 | EPVDDEELDAIDGSVAEGDEDIAPEAESDDDVADDADE* |
| SRR1726150 | EPVDDEELDAIDGSVAEGDEDIAPEAESDDDVADDADE* |
| SRR1996141 | EPVDDEELDAIDGSVAEGDEDIAPEAESDDDVADDADE* |
| SRR1107842 | EPVDDEELDAIDGSVAEGDEDIAPEAESDDDVADDADE* |
| SRR1157587 | EPVDDEELDAIDGSVAEGDEDIAPEAESDDDVADDADE* |
| SRR3027706 | EPVDDEELDAIDGSVAEGDEDIAPEAESDDDVADDADE* |
| SRR3027707 | EPVDDEELDAIDGSVAEGDEDIAPEAESDDDVADDADE* |
| SRR3027708 | EPVDDEELDAIDGSVAEGDEDIAPEAESDDDVADDADE* |
| SRR3027710 | EPVDDEELDAIDGSVAEGDEDIAPEAESDDDVADDADE* |
| SRR3027711 | EPVDDEELDAIDGSVAEGDEDIAPEAESDDDVADDADE* |
| SRR3027716 | EPVDDEELDAIDGSVAEGDEDIAPEAESDDDVADDADE* |
| SRR3027717 | EPVDDEELDAIDGSVAEGDEDIAPEAESDDDVADDADE* |
| SRR3027719 | EPVDDEELDAIDGSVAEGDEDIAPEAESDDDVADDADE* |
| SRR3027721 | EPVDDEELDAIDGSVAEGDEDIAPEAESDDDVADDADE* |
| SRR3027723 | EPVDDEELDAIDGSVAEGDEDIAPEAESDDDVADDADE* |
| SRR3115978 | EPVDDEELDAIDGSVAEGDEDIAPEAESDDDVADDADE* |
| SRR2534093 | EPVDDEELDAIDGSVAEGDEDIAPEAESDDDVADDADE* |
| SRR2534094 | EPVDDEELDAIDGSVAEGDEDIAPEAESDDDVADDADE* |
| SRR2534095 | EPVDDEELDAIDGSVAEGDEDIAPEAESDDDVADDADE* |
| SRR2534108 | EPVDDEELDAIDGSVAEGDEDIAPEAESDDDVADDADE* |
| SRR1106464 | EPVDDEELDAIDGSVAEGDEDIAPEAESDDDVADDADE* |
| SRR1106463 | EPVDDEELDAIDGSVAEGDEDIAPEAESDDDVADDADE* |
| SRR6949610 | EPVDDEELDAIDGSVAEGDEDIAPEAESDDDVADDADE* |
| SRR6950452 | EPVDDEELDAIDGSVAEGDEDIAPEAESDDDVADDADE* |
| ERR2019831 | EPVDDEELDAIDGSVAEGDEDIAPEAESDDDVADDADE* |
| SRR2085693 | EPVDDEELDAIDGSVAEGDEDIAPEAESDDDVADDADE* |
| SRR2086898 | EPVDDEELDAIDGSVAEGDEDIAPEAESDDDVADDADE* |
| SRR2175312 | EPVDDEELDAIDGSVAEGDEDIAPEAESDDDVADDADE* |
| SRR2175360 | EPVDDEELDAIDGSVAEGDEDIAPEAESDDDVADDADE* |
| SRR5231997 | EPVDDEELDAIDGSVAEGDEDIAPEAESDDDVADDADE* |
| SRR5232003 | EPVDDEELDAIDGSVAEGDEDIAPEAESDDDVADDADE* |
| SRR5232015 | EPVDDEELDAIDGSVAEGDEDIAPEAESDDDVADDADE* |
| SRR949434 | EPVDDEELDAIDGSVAEGDEDIAPEAESDDDVADDADE* |
| SRR3216575 | EPVDDEELDAIDGSVAEGDEDIAPEAESDDDVADDADE* |
| SRR5205342 | EPVDDEELDAIDGSVAEGDEDIAPEAESDDDVADDADE* |
| SRR1501669 | EPVDDEELDAIDGSVAEGDEDIAPEAESDDDVADDADE* |
| SRR5209740 | EPVDDEELDAIDGSVAEGDEDIAPEAESDDDVADDADE* |
| SRR3240355 | EPVDDEELDAIDGSVAEGDEDIAPEAESDDDVADDADE* |
| SRR3392777 | EPVDDEELDAIDGSVAEGDEDIAPEAESDDDVADDADE* |
| SRR3593671 | EPVDDEELDAIDGSVAEGDEDIAPEAESDDDVADDADE* |
| SRR5413290 | EPVDDEELDAIDGSVAEGDEDIAPEAESDDDVADDADE* |
| SRR5590269 | EPVDDEELDAIDGSVAEGDEDIAPEAESDDDVADDADE* |
| SRR5812103 | EPVDDEELDAIDGSVAEGDEDIAPEAESDDDVADDADE* |
| SRR2830941 | EPVDDEELDAIDGSVAEGDEDIAPEAESDDDVADDADE* |
| SRR2830966 | EPVDDEELDAIDGSVAEGDEDIAPEAESDDDVADDADE* |
| SRR3137270 | EPVDDEELDAIDGSVAEGDEDIAPEAESDDDVADDADE* |
| SRR3137271 | EPVDDEELDAIDGSVAEGDEDIAPEAESDDDVADDADE* |
| ERR526807 | EPVDDEELDAIDGSVAEGDEDIAPEAESDDDVADDADE* |
| ERR2197922 | EPVDDEELDAIDGSVAEGDEDIAPEAESDDDVADDADE* |
| ERR2197923 | EPVDDEELDAIDGSVAEGDEDIAPEAESDDDVADDADE* |
| ERR2197924 | EPVDDEELDAIDGSVAEGDEDIAPEAESDDDVADDADE* |
| ERR2197925 | EPVDDEELDAIDGSVAEGDEDIAPEAESDDDVADDADE* |
| ERR2197927 | EPVDDEELDAIDGSVAEGDEDIAPEAESDDDVADDADE* |
| ERR2197929 | EPVDDEELDAIDGSVAEGDEDIAPEAESDDDVADDADE* |
| SRR1648149 | EPVDDEELDAIDGSVAEGDEDIAPEAESDDDVADDADE* |
| SRR1048299 | EPVDDEELDAIDGSVAEGDEDIAPEAESDDDVADDADE* |
| SRR1300677 | EPVDDEELDAIDGSVAEGDEDIAPEAESDDDVADDADE* |
| SRR1288356 | EPVDDEELDAIDGSVAEGDEDIAPEAESDDDVADDADE* |
| SRR7426190 | EPVDDEELDAIDGSVAEGDEDIAPEAESDDDVADDADE* |
| SRR7426192 | EPVDDEELDAIDGSVAEGDEDIAPEAESDDDVADDADE* |
| SRR7426193 | EPVDDEELDAIDGSVAEGDEDIAPEAESDDDVADDADE* |
| SRR7441832 | EPVDDEELDAIDGSVAEGDEDIAPEAESDDDVADDADE* |
| SRR7426179 | EPVDDEELDAIDGSVAEGDEDIAPEAESDDDVADDADE* |
| SRR7439238 | EPVDDEELDAIDGSVAEGDEDIAPEAESDDDVADDADE* |
| SRR7439244 | EPVDDEELDAIDGSVAEGDEDIAPEAESDDDVADDADE* |
| SRR7439259 | EPVDDEELDAIDGSVAEGDEDIAPEAESDDDVADDADE* |
| SRR7439260 | EPVDDEELDAIDGSVAEGDEDIAPEAESDDDVADDADE* |
| SRR7441786 | EPVDDEELDAIDGSVAEGDEDIAPEAESDDDVADDADE* |
| SRR7441797 | EPVDDEELDAIDGSVAEGDEDIAPEAESDDDVADDADE* |
| ERR1759093 | EPVDDEELDAIDGSVAEGDEDIAPEAESDDDVADDADE* |
| ERR2580275 | EPVDDEELDAIDGSVAEGDEDIAPEAESDDDVADDADE* |
| ERR1759204 | EPVDDEELDAIDGSVAEGDEDIAPEAESDDDVADDADE* |
| SRR1300699 | EPVDDEELDAIDGSVAEGDEDIAPEAESDDDVADDADE* |
| S_0825_17 | EPVDDEELDAIDGSVAEGDEDIAPEAESDDDVADDADE* |
| SRR1958215 | EPVDDEELDAIDGSVAEGDEDIAPEAESDDDVADDADE* |
| SRR1958540 | EPVDDEELDAIDGSVAEGDEDIAPEAESDDDVADDADE* |
| SRR1958636 | EPVDDEELDAIDGSVAEGDEDIAPEAESDDDVADDADE* |
| SRR1959422 | EPVDDEELDAIDGSVAEGDEDIAPEAESDDDVADDADE* |
| SRR1959427 | EPVDDEELDAIDGSVAEGDEDIAPEAESDDDVADDADE* |
| SRR1960226 | EPVDDEELDAIDGSVAEGDEDIAPEAESDDDVADDADE* |
| SRR1963498 | EPVDDEELDAIDGSVAEGDEDIAPEAESDDDVADDADE* |
| SRR1965947 | EPVDDEELDAIDGSVAEGDEDIAPEAESDDDVADDADE* |
| SRR1966125 | EPVDDEELDAIDGSVAEGDEDIAPEAESDDDVADDADE* |
| SRR1966330 | EPVDDEELDAIDGSVAEGDEDIAPEAESDDDVADDADE* |
| SRR1966565 | EPVDDEELDAIDGSVAEGDEDIAPEAESDDDVADDADE* |
| SRR1966864 | EPVDDEELDAIDGSVAEGDEDIAPEAESDDDVADDADE* |
| SRR1966989 | EPVDDEELDAIDGSVAEGDEDIAPEAESDDDVADDADE* |
| SRR1967688 | EPVDDEELDAIDGSVAEGDEDIAPEAESDDDVADDADE* |
| SRR1967733 | EPVDDEELDAIDGSVAEGDEDIAPEAESDDDVADDADE* |
| SRR1967746 | EPVDDEELDAIDGSVAEGDEDIAPEAESDDDVADDADE* |
| SRR1968341 | EPVDDEELDAIDGSVAEGDEDIAPEAESDDDVADDADE* |
| SRR1968456 | EPVDDEELDAIDGSVAEGDEDIAPEAESDDDVADDADE* |
| SRR1968465 | EPVDDEELDAIDGSVAEGDEDIAPEAESDDDVADDADE* |
| SRR1968761 | EPVDDEELDAIDGSVAEGDEDIAPEAESDDDVADDADE* |
| SRR1969047 | EPVDDEELDAIDGSVAEGDEDIAPEAESDDDVADDADE* |
| SRR1969255 | EPVDDEELDAIDGSVAEGDEDIAPEAESDDDVADDADE* |
| SRR1969412 | EPVDDEELDAIDGSVAEGDEDIAPEAESDDDVADDADE* |
| SRR1969524 | EPVDDEELDAIDGSVAEGDEDIAPEAESDDDVADDADE* |
| SRR1969584 | EPVDDEELDAIDGSVAEGDEDIAPEAESDDDVADDADE* |
| SRR1969648 | EPVDDEELDAIDGSVAEGDEDIAPEAESDDDVADDADE* |
| SRR1969804 | EPVDDEELDAIDGSVAEGDEDIAPEAESDDDVADDADE* |
| SRR1970221 | EPVDDEELDAIDGSVAEGDEDIAPEAESDDDVADDADE* |
| SRR1970268 | EPVDDEELDAIDGSVAEGDEDIAPEAESDDDVADDADE* |
| SRR1965862 | EPVDDEELDAIDGSVAEGDEDIAPEAESDDDVADDADE* |
| SRR1967363 | EPVDDEELDAIDGSVAEGDEDIAPEAESDDDVADDADE* |
| SRR1968276 | EPVDDEELDAIDGSVAEGDEDIAPEAESDDDVADDADE* |
| SRR1968967 | EPVDDEELDAIDGSVAEGDEDIAPEAESDDDVADDADE* |
| SRR3321531 | EPVDDEELDAIDGSVAEGDEDIAPEAESDDDVADDADE* |
| SRR3321883 | EPVDDEELDAIDGSVAEGDEDIAPEAESDDDVADDADE* |
| SRR3322413 | EPVDDEELDAIDGSVAEGDEDIAPEAESDDDVADDADE* |
| SRR3323012 | EPVDDEELDAIDGSVAEGDEDIAPEAESDDDVADDADE* |
| SRR5194289 | EPVDDEELDAIDGSVAEGDEDIAPEAESDDDVADDADE* |
| SRR7163798 | EPVDDEELDAIDGSVAEGDEDIAPEAESDDDVADDADE* |
| SRR7172610 | EPVDDEELDAIDGSVAEGDEDIAPEAESDDDVADDADE* |
| SRR7204568 | EPVDDEELDAIDGSVAEGDEDIAPEAESDDDVADDADE* |
| SRR7223230 | EPVDDEELDAIDGSVAEGDEDIAPEAESDDDVADDADE* |
| SRR7230675 | EPVDDEELDAIDGSVAEGDEDIAPEAESDDDVADDADE* |
| SRR7278056 | EPVDDEELDAIDGSVAEGDEDIAPEAESDDDVADDADE* |
| SRR7278086 | EPVDDEELDAIDGSVAEGDEDIAPEAESDDDVADDADE* |
| SRR7285841 | EPVDDEELDAIDGSVAEGDEDIAPEAESDDDVADDADE* |
| SRR7292625 | EPVDDEELDAIDGSVAEGDEDIAPEAESDDDVADDADE* |
| SRR7292665 | EPVDDEELDAIDGSVAEGDEDIAPEAESDDDVADDADE* |
| SRR7297965 | EPVDDEELDAIDGSVAEGDEDIAPEAESDDDVADDADE* |
| SRR7350726 | EPVDDEELDAIDGSVAEGDEDIAPEAESDDDVADDADE* |
| SRR7410328 | EPVDDEELDAIDGSVAEGDEDIAPEAESDDDVADDADE* |
| SRR7474665 | EPVDDEELDAIDGSVAEGDEDIAPEAESDDDVADDADE* |
| SRR7523184 | EPVDDEELDAIDGSVAEGDEDIAPEAESDDDVADDADE* |
| SRR7187264 | EPVDDEELDAIDGSVAEGDEDIAPEAESDDDVADDADE* |
| SRR7204445 | EPVDDEELDAIDGSVAEGDEDIAPEAESDDDVADDADE* |
| SRR7285641 | EPVDDEELDAIDGSVAEGDEDIAPEAESDDDVADDADE* |
| SRR7286695 | EPVDDEELDAIDGSVAEGDEDIAPEAESDDDVADDADE* |
| SRR7286705 | EPVDDEELDAIDGSVAEGDEDIAPEAESDDDVADDADE* |
| SRR7292931 | EPVDDEELDAIDGSVAEGDEDIAPEAESDDDVADDADE* |
| SRR7310349 | EPVDDEELDAIDGSVAEGDEDIAPEAESDDDVADDADE* |
| SRR7351616 | EPVDDEELDAIDGSVAEGDEDIAPEAESDDDVADDADE* |
| SRR7414818 | EPVDDEELDAIDGSVAEGDEDIAPEAESDDDVADDADE* |
| SRR7426480 | EPVDDEELDAIDGSVAEGDEDIAPEAESDDDVADDADE* |
| SRR5584105 | EPVDDEELDAIDGSVAEGDEDIAPEAESDDDVADDADE* |
| SRR5584565 | EPVDDEELDAIDGSVAEGDEDIAPEAESDDDVADDADE* |
| SRR5584614 | EPVDDEELDAIDGSVAEGDEDIAPEAESDDDVADDADE* |
| SRR5631543 | EPVDDEELDAIDGSVAEGDEDIAPEAESDDDVADDADE* |
| SRR5631553 | EPVDDEELDAIDGSVAEGDEDIAPEAESDDDVADDADE* |
| SRR7123196 | EPVDDEELDAIDGSVAEGDEDIAPEAESDDDVADDADE* |
| SRR7163819 | EPVDDEELDAIDGSVAEGDEDIAPEAESDDDVADDADE* |
| SRR7163920 | EPVDDEELDAIDGSVAEGDEDIAPEAESDDDVADDADE* |
| SRR7209528 | EPVDDEELDAIDGSVAEGDEDIAPEAESDDDVADDADE* |
| SRR7249868 | EPVDDEELDAIDGSVAEGDEDIAPEAESDDDVADDADE* |
| SRR7278088 | EPVDDEELDAIDGSVAEGDEDIAPEAESDDDVADDADE* |
| SRR7285788 | EPVDDEELDAIDGSVAEGDEDIAPEAESDDDVADDADE* |
| SRR7286789 | EPVDDEELDAIDGSVAEGDEDIAPEAESDDDVADDADE* |
| SRR7286886 | EPVDDEELDAIDGSVAEGDEDIAPEAESDDDVADDADE* |
| SRR7310632 | EPVDDEELDAIDGSVAEGDEDIAPEAESDDDVADDADE* |
| SRR7350631 | EPVDDEELDAIDGSVAEGDEDIAPEAESDDDVADDADE* |
| SRR7458741 | EPVDDEELDAIDGSVAEGDEDIAPEAESDDDVADDADE* |
| SRR7480280 | EPVDDEELDAIDGSVAEGDEDIAPEAESDDDVADDADE* |
| SRR7523660 | EPVDDEELDAIDGSVAEGDEDIAPEAESDDDVADDADE* |
| SRR7523775 | EPVDDEELDAIDGSVAEGDEDIAPEAESDDDVADDADE* |
| SRR7251101 | EPVDDEELDAIDGSVAEGDEDIAPEAESDDDVADDADE* |
| SRR7284299 | EPVDDEELDAIDGSVAEGDEDIAPEAESDDDVADDADE* |
| SRR7285738 | EPVDDEELDAIDGSVAEGDEDIAPEAESDDDVADDADE* |
| SRR7310640 | EPVDDEELDAIDGSVAEGDEDIAPEAESDDDVADDADE* |
| SRR7349159 | EPVDDEELDAIDGSVAEGDEDIAPEAESDDDVADDADE* |
| SRR7474873 | EPVDDEELDAIDGSVAEGDEDIAPEAESDDDVADDADE* |
| SRR7495689 | EPVDDEELDAIDGSVAEGDEDIAPEAESDDDVADDADE* |
| SRR7495752 | EPVDDEELDAIDGSVAEGDEDIAPEAESDDDVADDADE* |
