# Supplementary material for: Genomic epidemiology of emerging ESBL-producing Salmonella Kentucky blaCTX-M-14b in Europe
Source: Emerg Microbes Infect. 2020 Sep 30;9(1):2124–35. doi: 10.1080/22221751.2020.1821582 (PMC7580578; doi:10.1080/22221751.2020.1821582)
Supplement: Suppelemental_files.zip [file TEMI_A_1821582_SM9137.zip › AlignS3.docx]

| AlignS3 |  |
| --- | --- |
| CLUSTAL W (1.7) multiple sequence alignment | |
|  |  |
|  |  |
| blaCTX-M-104_1_HQ833652 | ATGGTGACAAAGAGAGTGCAACGGATGATGTTCGCGGCGGCGGCGTGCATTCCGCTGCTG |
| S18BD05011 | ATGGTGACAAAGAGAGTGCAACGGATGATGTTCGCGGCGGCGGCGTGCATTCCGCTGCTG |
| blaCTX-M-14_1_AF252622 | ATGGTGACAAAGAGAGTGCAACGGATGATGTTCGCGGCGGCGGCGTGCATTCCGCTGCTG |
| blaCTX-M-14b_1_DQ359215 | ATGGTGACAAAGAGAGTGCAACGGATGATGTTCGCGGCGGCGGCGTGCATTCCGCTGCTG |
| 15EP001483 | ATGGTGACAAAGAGAGTGCAACGGATGATGTTCGCGGCGGCGGCGTGCATTCCGCTGCTG |
| 17EP002363 | ATGGTGACAAAGAGAGTGCAACGGATGATGTTCGCGGCGGCGGCGTGCATTCCGCTGCTG |
| 313865 | ATGGTGACAAAGAGAGTGCAACGGATGATGTTCGCGGCGGCGGCGTGCATTCCGCTGCTG |
| ERR2173656 | ATGGTGACAAAGAGAGTGCAACGGATGATGTTCGCGGCGGCGGCGTGCATTCCGCTGCTG |
| ERR2580274 | ATGGTGACAAAGAGAGTGCAACGGATGATGTTCGCGGCGGCGGCGTGCATTCCGCTGCTG |
| ERR2580277 | ATGGTGACAAAGAGAGTGCAACGGATGATGTTCGCGGCGGCGGCGTGCATTCCGCTGCTG |
| SRR8704720 | ATGGTGACAAAGAGAGTGCAACGGATGATGTTCGCGGCGGCGGCGTGCATTCCGCTGCTG |
| MT16-000061 | ATGGTGACAAAGAGAGTGCAACGGATGATGTTCGCGGCGGCGGCGTGCATTCCGCTGCTG |
| MT16-019416 | ATGGTGACAAAGAGAGTGCAACGGATGATGTTCGCGGCGGCGGCGTGCATTCCGCTGCTG |
| MT16-027865 | ATGGTGACAAAGAGAGTGCAACGGATGATGTTCGCGGCGGCGGCGTGCATTCCGCTGCTG |
| MT16-031693 | ATGGTGACAAAGAGAGTGCAACGGATGATGTTCGCGGCGGCGGCGTGCATTCCGCTGCTG |
| MT16-040253 | ATGGTGACAAAGAGAGTGCAACGGATGATGTTCGCGGCGGCGGCGTGCATTCCGCTGCTG |
| MT16-045379 | ATGGTGACAAAGAGAGTGCAACGGATGATGTTCGCGGCGGCGGCGTGCATTCCGCTGCTG |
| MT16-442728 | ATGGTGACAAAGAGAGTGCAACGGATGATGTTCGCGGCGGCGGCGTGCATTCCGCTGCTG |
| MT16-462857 | ATGGTGACAAAGAGAGTGCAACGGATGATGTTCGCGGCGGCGGCGTGCATTCCGCTGCTG |
| MT16-480196 | ATGGTGACAAAGAGAGTGCAACGGATGATGTTCGCGGCGGCGGCGTGCATTCCGCTGCTG |
| MT16-861555 | ATGGTGACAAAGAGAGTGCAACGGATGATGTTCGCGGCGGCGGCGTGCATTCCGCTGCTG |
| MT17-076833 | ATGGTGACAAAGAGAGTGCAACGGATGATGTTCGCGGCGGCGGCGTGCATTCCGCTGCTG |
| MT17-110677 | ATGGTGACAAAGAGAGTGCAACGGATGATGTTCGCGGCGGCGGCGTGCATTCCGCTGCTG |
| MT17-131730 | ATGGTGACAAAGAGAGTGCAACGGATGATGTTCGCGGCGGCGGCGTGCATTCCGCTGCTG |
| MT17-140890 | ATGGTGACAAAGAGAGTGCAACGGATGATGTTCGCGGCGGCGGCGTGCATTCCGCTGCTG |
| MT17-141840 | ATGGTGACAAAGAGAGTGCAACGGATGATGTTCGCGGCGGCGGCGTGCATTCCGCTGCTG |
| MT17-152488 | ATGGTGACAAAGAGAGTGCAACGGATGATGTTCGCGGCGGCGGCGTGCATTCCGCTGCTG |
| MT17-157311 | ATGGTGACAAAGAGAGTGCAACGGATGATGTTCGCGGCGGCGGCGTGCATTCCGCTGCTG |
| MT17-161645 | ATGGTGACAAAGAGAGTGCAACGGATGATGTTCGCGGCGGCGGCGTGCATTCCGCTGCTG |
| MT17-167951 | ATGGTGACAAAGAGAGTGCAACGGATGATGTTCGCGGCGGCGGCGTGCATTCCGCTGCTG |
| MT18-217732 | ATGGTGACAAAGAGAGTGCAACGGATGATGTTCGCGGCGGCGGCGTGCATTCCGCTGCTG |
| MT18-252580 | ATGGTGACAAAGAGAGTGCAACGGATGATGTTCGCGGCGGCGGCGTGCATTCCGCTGCTG |
| RIVM_H_2016-04 | ATGGTGACAAAGAGAGTGCAACGGATGATGTTCGCGGCGGCGGCGTGCATTCCGCTGCTG |
| RIVM_H_2016-07 | ATGGTGACAAAGAGAGTGCAACGGATGATGTTCGCGGCGGCGGCGTGCATTCCGCTGCTG |
| RIVM_H_2016-08 | ATGGTGACAAAGAGAGTGCAACGGATGATGTTCGCGGCGGCGGCGTGCATTCCGCTGCTG |
| RIVM_H_2016-09 | ATGGTGACAAAGAGAGTGCAACGGATGATGTTCGCGGCGGCGGCGTGCATTCCGCTGCTG |
| RIVM_H_2016-11 | ATGGTGACAAAGAGAGTGCAACGGATGATGTTCGCGGCGGCGGCGTGCATTCCGCTGCTG |
| RIVM_H_2016-12 | ATGGTGACAAAGAGAGTGCAACGGATGATGTTCGCGGCGGCGGCGTGCATTCCGCTGCTG |
| RIVM_H_2016-13 | ATGGTGACAAAGAGAGTGCAACGGATGATGTTCGCGGCGGCGGCGTGCATTCCGCTGCTG |
| RIVM_H_2016-14 | ATGGTGACAAAGAGAGTGCAACGGATGATGTTCGCGGCGGCGGCGTGCATTCCGCTGCTG |
| RIVM_H_2016-15 | ATGGTGACAAAGAGAGTGCAACGGATGATGTTCGCGGCGGCGGCGTGCATTCCGCTGCTG |
| RIVM_H_2017-02 | ATGGTGACAAAGAGAGTGCAACGGATGATGTTCGCGGCGGCGGCGTGCATTCCGCTGCTG |
| RIVM_H_2017-03 | ATGGTGACAAAGAGAGTGCAACGGATGATGTTCGCGGCGGCGGCGTGCATTCCGCTGCTG |
| RIVM_H_2017-05 | ATGGTGACAAAGAGAGTGCAACGGATGATGTTCGCGGCGGCGGCGTGCATTCCGCTGCTG |
| RIVM_H_2017-07 | ATGGTGACAAAGAGAGTGCAACGGATGATGTTCGCGGCGGCGGCGTGCATTCCGCTGCTG |
| RIVM_H_2017-08 | ATGGTGACAAAGAGAGTGCAACGGATGATGTTCGCGGCGGCGGCGTGCATTCCGCTGCTG |
| RIVM_H_2017-09 | ATGGTGACAAAGAGAGTGCAACGGATGATGTTCGCGGCGGCGGCGTGCATTCCGCTGCTG |
| RIVM_H_2017-10 | ATGGTGACAAAGAGAGTGCAACGGATGATGTTCGCGGCGGCGGCGTGCATTCCGCTGCTG |
| RIVM_H_2017-11 | ATGGTGACAAAGAGAGTGCAACGGATGATGTTCGCGGCGGCGGCGTGCATTCCGCTGCTG |
| RIVM_H_2017-12 | ATGGTGACAAAGAGAGTGCAACGGATGATGTTCGCGGCGGCGGCGTGCATTCCGCTGCTG |
| RIVM_H_2017-13 | ATGGTGACAAAGAGAGTGCAACGGATGATGTTCGCGGCGGCGGCGTGCATTCCGCTGCTG |
| RIVM_H_2017-14 | ATGGTGACAAAGAGAGTGCAACGGATGATGTTCGCGGCGGCGGCGTGCATTCCGCTGCTG |
| RIVM_H_2017-19 | ATGGTGACAAAGAGAGTGCAACGGATGATGTTCGCGGCGGCGGCGTGCATTCCGCTGCTG |
| RKI_16-04315 | ATGGTGACAAAGAGAGTGCAACGGATGATGTTCGCGGCGGCGGCGTGCATTCCGCTGCTG |
| RKI_17-02304 | ATGGTGACAAAGAGAGTGCAACGGATGATGTTCGCGGCGGCGGCGTGCATTCCGCTGCTG |
| RKI_17-02411 | ATGGTGACAAAGAGAGTGCAACGGATGATGTTCGCGGCGGCGGCGTGCATTCCGCTGCTG |
| RKI_17-02757 | ATGGTGACAAAGAGAGTGCAACGGATGATGTTCGCGGCGGCGGCGTGCATTCCGCTGCTG |
| RKI_17-04797 | ATGGTGACAAAGAGAGTGCAACGGATGATGTTCGCGGCGGCGGCGTGCATTCCGCTGCTG |
| S16BD08730 | ATGGTGACAAAGAGAGTGCAACGGATGATGTTCGCGGCGGCGGCGTGCATTCCGCTGCTG |
| S18BD03994 | ATGGTGACAAAGAGAGTGCAACGGATGATGTTCGCGGCGGCGGCGTGCATTCCGCTGCTG |
| SRR1957844 | ATGGTGACAAAGAGAGTGCAACGGATGATGTTCGCGGCGGCGGCGTGCATTCCGCTGCTG |
| SRR1958654 | ATGGTGACAAAGAGAGTGCAACGGATGATGTTCGCGGCGGCGGCGTGCATTCCGCTGCTG |
| SRR1965077 | ATGGTGACAAAGAGAGTGCAACGGATGATGTTCGCGGCGGCGGCGTGCATTCCGCTGCTG |
| SRR1966369 | ATGGTGACAAAGAGAGTGCAACGGATGATGTTCGCGGCGGCGGCGTGCATTCCGCTGCTG |
| SRR1967117 | ATGGTGACAAAGAGAGTGCAACGGATGATGTTCGCGGCGGCGGCGTGCATTCCGCTGCTG |
| SRR1967922 | ATGGTGACAAAGAGAGTGCAACGGATGATGTTCGCGGCGGCGGCGTGCATTCCGCTGCTG |
| SRR5583183 | ATGGTGACAAAGAGAGTGCAACGGATGATGTTCGCGGCGGCGGCGTGCATTCCGCTGCTG |
| SRR5585240 | ATGGTGACAAAGAGAGTGCAACGGATGATGTTCGCGGCGGCGGCGTGCATTCCGCTGCTG |
| SRR7216071 | ATGGTGACAAAGAGAGTGCAACGGATGATGTTCGCGGCGGCGGCGTGCATTCCGCTGCTG |
| SRR7277793 | ATGGTGACAAAGAGAGTGCAACGGATGATGTTCGCGGCGGCGGCGTGCATTCCGCTGCTG |
| SRR7284317 | ATGGTGACAAAGAGAGTGCAACGGATGATGTTCGCGGCGGCGGCGTGCATTCCGCTGCTG |
| SRR7299161 | ATGGTGACAAAGAGAGTGCAACGGATGATGTTCGCGGCGGCGGCGTGCATTCCGCTGCTG |
| SRR7343877 | ATGGTGACAAAGAGAGTGCAACGGATGATGTTCGCGGCGGCGGCGTGCATTCCGCTGCTG |
| SRR7351477 | ATGGTGACAAAGAGAGTGCAACGGATGATGTTCGCGGCGGCGGCGTGCATTCCGCTGCTG |
| SRR7401730 | ATGGTGACAAAGAGAGTGCAACGGATGATGTTCGCGGCGGCGGCGTGCATTCCGCTGCTG |
| SRR7469092 | ATGGTGACAAAGAGAGTGCAACGGATGATGTTCGCGGCGGCGGCGTGCATTCCGCTGCTG |
| SRR7523148 | ATGGTGACAAAGAGAGTGCAACGGATGATGTTCGCGGCGGCGGCGTGCATTCCGCTGCTG |
| SRR7523854 | ATGGTGACAAAGAGAGTGCAACGGATGATGTTCGCGGCGGCGGCGTGCATTCCGCTGCTG |
| SRR7842487 | ATGGTGACAAAGAGAGTGCAACGGATGATGTTCGCGGCGGCGGCGTGCATTCCGCTGCTG |
| SRR7879556 | ATGGTGACAAAGAGAGTGCAACGGATGATGTTCGCGGCGGCGGCGTGCATTCCGCTGCTG |
| SRR8054524 | ATGGTGACAAAGAGAGTGCAACGGATGATGTTCGCGGCGGCGGCGTGCATTCCGCTGCTG |
| SRR8054525 | ATGGTGACAAAGAGAGTGCAACGGATGATGTTCGCGGCGGCGGCGTGCATTCCGCTGCTG |
| SRR8524733 | ATGGTGACAAAGAGAGTGCAACGGATGATGTTCGCGGCGGCGGCGTGCATTCCGCTGCTG |
| SRR8526100 | ATGGTGACAAAGAGAGTGCAACGGATGATGTTCGCGGCGGCGGCGTGCATTCCGCTGCTG |
| SRR8553991 | ATGGTGACAAAGAGAGTGCAACGGATGATGTTCGCGGCGGCGGCGTGCATTCCGCTGCTG |
| blaCTX-M-15_1_AY044436 | ATGGTTAAAAAATCACTGCGCCAGTTCACGCTGATGGCGACGGCAACCGTCACGCTGTTG |
| RIVM_H_2017-15 | ATGGTTAAAAAATCACTGCGCCAGTTCACGCTGATGGCGACGGCAACCGTCACGCTGTTG |
| RKI_17-06869 | ATGGTTAAAAAATCACTGCGCCAGTTCACGCTGATGGCGACGGCAACCGTCACGCTGTTG |
| blaCTX-M-55_1_DQ810789 | ATGGTTAAAAAATCACTGCGCCAGTTCACGCTGATGGCGACGGCAACCGTCACGCTGTTG |
| SRR7349175 | ATGGTTAAAAAATCACTGCGCCAGTTCACGCTGATGGCGACGGCAACCGTCACGCTGTTG |
| ------------------------------------------------------------------------------------ | |
| blaCTX-M-104_1_HQ833652 | CTGGGCAGCGCGCCGCTTTATGCGCAGACGAGTGCGGTGCAGCAAAAGCTGGCGGCGCTG |
| S18BD05011 | CTGGGCAGCGCGCCGCTTTATGCGCAGACGAGTGCGGTGCAGCAAAAGCTGGCGGCGCTG |
| blaCTX-M-14_1_AF252622 | CTGGGCAGCGCGCCGCTTTATGCGCAGACGAGTGCGGTGCAGCAAAAGCTGGCGGCGCTG |
| blaCTX-M-14b_1_DQ359215 | CTGGGCAGCGCGCCGCTTTATGCGCAGACGAGTGCGGTGCAGCAAAAGCTGGCGGCGCTG |
| 15EP001483 | CTGGGCAGCGCGCCGCTTTATGCGCAGACGAGTGCGGTGCAGCAAAAGCTGGCGGCGCTG |
| 17EP002363 | CTGGGCAGCGCGCCGCTTTATGCGCAGACGAGTGCGGTGCAGCAAAAGCTGGCGGCGCTG |
| 313865 | CTGGGCAGCGCGCCGCTTTATGCGCAGACGAGTGCGGTGCAGCAAAAGCTGGCGGCGCTG |
| ERR2173656 | CTGGGCAGCGCGCCGCTTTATGCGCAGACGAGTGCGGTGCAGCAAAAGCTGGCGGCGCTG |
| ERR2580274 | CTGGGCAGCGCGCCGCTTTATGCGCAGACGAGTGCGGTGCAGCAAAAGCTGGCGGCGCTG |
| ERR2580277 | CTGGGCAGCGCGCCGCTTTATGCGCAGACGAGTGCGGTGCAGCAAAAGCTGGCGGCGCTG |
| SRR8704720 | CTGGGCAGCGCGCCGCTTTATGCGCAGACGAGTGCGGTGCAGCAAAAGCTGGCGGCGCTG |
| MT16-000061 | CTGGGCAGCGCGCCGCTTTATGCGCAGACGAGTGCGGTGCAGCAAAAGCTGGCGGCGCTG |
| MT16-019416 | CTGGGCAGCGCGCCGCTTTATGCGCAGACGAGTGCGGTGCAGCAAAAGCTGGCGGCGCTG |
| MT16-027865 | CTGGGCAGCGCGCCGCTTTATGCGCAGACGAGTGCGGTGCAGCAAAAGCTGGCGGCGCTG |
| MT16-031693 | CTGGGCAGCGCGCCGCTTTATGCGCAGACGAGTGCGGTGCAGCAAAAGCTGGCGGCGCTG |
| MT16-040253 | CTGGGCAGCGCGCCGCTTTATGCGCAGACGAGTGCGGTGCAGCAAAAGCTGGCGGCGCTG |
| MT16-045379 | CTGGGCAGCGCGCCGCTTTATGCGCAGACGAGTGCGGTGCAGCAAAAGCTGGCGGCGCTG |
| MT16-442728 | CTGGGCAGCGCGCCGCTTTATGCGCAGACGAGTGCGGTGCAGCAAAAGCTGGCGGCGCTG |
| MT16-462857 | CTGGGCAGCGCGCCGCTTTATGCGCAGACGAGTGCGGTGCAGCAAAAGCTGGCGGCGCTG |
| MT16-480196 | CTGGGCAGCGCGCCGCTTTATGCGCAGACGAGTGCGGTGCAGCAAAAGCTGGCGGCGCTG |
| MT16-861555 | CTGGGCAGCGCGCCGCTTTATGCGCAGACGAGTGCGGTGCAGCAAAAGCTGGCGGCGCTG |
| MT17-076833 | CTGGGCAGCGCGCCGCTTTATGCGCAGACGAGTGCGGTGCAGCAAAAGCTGGCGGCGCTG |
| MT17-110677 | CTGGGCAGCGCGCCGCTTTATGCGCAGACGAGTGCGGTGCAGCAAAAGCTGGCGGCGCTG |
| MT17-131730 | CTGGGCAGCGCGCCGCTTTATGCGCAGACGAGTGCGGTGCAGCAAAAGCTGGCGGCGCTG |
| MT17-140890 | CTGGGCAGCGCGCCGCTTTATGCGCAGACGAGTGCGGTGCAGCAAAAGCTGGCGGCGCTG |
| MT17-141840 | CTGGGCAGCGCGCCGCTTTATGCGCAGACGAGTGCGGTGCAGCAAAAGCTGGCGGCGCTG |
| MT17-152488 | CTGGGCAGCGCGCCGCTTTATGCGCAGACGAGTGCGGTGCAGCAAAAGCTGGCGGCGCTG |
| MT17-157311 | CTGGGCAGCGCGCCGCTTTATGCGCAGACGAGTGCGGTGCAGCAAAAGCTGGCGGCGCTG |
| MT17-161645 | CTGGGCAGCGCGCCGCTTTATGCGCAGACGAGTGCGGTGCAGCAAAAGCTGGCGGCGCTG |
| MT17-167951 | CTGGGCAGCGCGCCGCTTTATGCGCAGACGAGTGCGGTGCAGCAAAAGCTGGCGGCGCTG |
| MT18-217732 | CTGGGCAGCGCGCCGCTTTATGCGCAGACGAGTGCGGTGCAGCAAAAGCTGGCGGCGCTG |
| MT18-252580 | CTGGGCAGCGCGCCGCTTTATGCGCAGACGAGTGCGGTGCAGCAAAAGCTGGCGGCGCTG |
| RIVM_H_2016-04 | CTGGGCAGCGCGCCGCTTTATGCGCAGACGAGTGCGGTGCAGCAAAAGCTGGCGGCGCTG |
| RIVM_H_2016-07 | CTGGGCAGCGCGCCGCTTTATGCGCAGACGAGTGCGGTGCAGCAAAAGCTGGCGGCGCTG |
| RIVM_H_2016-08 | CTGGGCAGCGCGCCGCTTTATGCGCAGACGAGTGCGGTGCAGCAAAAGCTGGCGGCGCTG |
| RIVM_H_2016-09 | CTGGGCAGCGCGCCGCTTTATGCGCAGACGAGTGCGGTGCAGCAAAAGCTGGCGGCGCTG |
| RIVM_H_2016-11 | CTGGGCAGCGCGCCGCTTTATGCGCAGACGAGTGCGGTGCAGCAAAAGCTGGCGGCGCTG |
| RIVM_H_2016-12 | CTGGGCAGCGCGCCGCTTTATGCGCAGACGAGTGCGGTGCAGCAAAAGCTGGCGGCGCTG |
| RIVM_H_2016-13 | CTGGGCAGCGCGCCGCTTTATGCGCAGACGAGTGCGGTGCAGCAAAAGCTGGCGGCGCTG |
| RIVM_H_2016-14 | CTGGGCAGCGCGCCGCTTTATGCGCAGACGAGTGCGGTGCAGCAAAAGCTGGCGGCGCTG |
| RIVM_H_2016-15 | CTGGGCAGCGCGCCGCTTTATGCGCAGACGAGTGCGGTGCAGCAAAAGCTGGCGGCGCTG |
| RIVM_H_2017-02 | CTGGGCAGCGCGCCGCTTTATGCGCAGACGAGTGCGGTGCAGCAAAAGCTGGCGGCGCTG |
| RIVM_H_2017-03 | CTGGGCAGCGCGCCGCTTTATGCGCAGACGAGTGCGGTGCAGCAAAAGCTGGCGGCGCTG |
| RIVM_H_2017-05 | CTGGGCAGCGCGCCGCTTTATGCGCAGACGAGTGCGGTGCAGCAAAAGCTGGCGGCGCTG |
| RIVM_H_2017-07 | CTGGGCAGCGCGCCGCTTTATGCGCAGACGAGTGCGGTGCAGCAAAAGCTGGCGGCGCTG |
| RIVM_H_2017-08 | CTGGGCAGCGCGCCGCTTTATGCGCAGACGAGTGCGGTGCAGCAAAAGCTGGCGGCGCTG |
| RIVM_H_2017-09 | CTGGGCAGCGCGCCGCTTTATGCGCAGACGAGTGCGGTGCAGCAAAAGCTGGCGGCGCTG |
| RIVM_H_2017-10 | CTGGGCAGCGCGCCGCTTTATGCGCAGACGAGTGCGGTGCAGCAAAAGCTGGCGGCGCTG |
| RIVM_H_2017-11 | CTGGGCAGCGCGCCGCTTTATGCGCAGACGAGTGCGGTGCAGCAAAAGCTGGCGGCGCTG |
| RIVM_H_2017-12 | CTGGGCAGCGCGCCGCTTTATGCGCAGACGAGTGCGGTGCAGCAAAAGCTGGCGGCGCTG |
| RIVM_H_2017-13 | CTGGGCAGCGCGCCGCTTTATGCGCAGACGAGTGCGGTGCAGCAAAAGCTGGCGGCGCTG |
| RIVM_H_2017-14 | CTGGGCAGCGCGCCGCTTTATGCGCAGACGAGTGCGGTGCAGCAAAAGCTGGCGGCGCTG |
| RIVM_H_2017-19 | CTGGGCAGCGCGCCGCTTTATGCGCAGACGAGTGCGGTGCAGCAAAAGCTGGCGGCGCTG |
| RKI_16-04315 | CTGGGCAGCGCGCCGCTTTATGCGCAGACGAGTGCGGTGCAGCAAAAGCTGGCGGCGCTG |
| RKI_17-02304 | CTGGGCAGCGCGCCGCTTTATGCGCAGACGAGTGCGGTGCAGCAAAAGCTGGCGGCGCTG |
| RKI_17-02411 | CTGGGCAGCGCGCCGCTTTATGCGCAGACGAGTGCGGTGCAGCAAAAGCTGGCGGCGCTG |
| RKI_17-02757 | CTGGGCAGCGCGCCGCTTTATGCGCAGACGAGTGCGGTGCAGCAAAAGCTGGCGGCGCTG |
| RKI_17-04797 | CTGGGCAGCGCGCCGCTTTATGCGCAGACGAGTGCGGTGCAGCAAAAGCTGGCGGCGCTG |
| S16BD08730 | CTGGGCAGCGCGCCGCTTTATGCGCAGACGAGTGCGGTGCAGCAAAAGCTGGCGGCGCTG |
| S18BD03994 | CTGGGCAGCGCGCCGCTTTATGCGCAGACGAGTGCGGTGCAGCAAAAGCTGGCGGCGCTG |
| SRR1957844 | CTGGGCAGCGCGCCGCTTTATGCGCAGACGAGTGCGGTGCAGCAAAAGCTGGCGGCGCTG |
| SRR1958654 | CTGGGCAGCGCGCCGCTTTATGCGCAGACGAGTGCGGTGCAGCAAAAGCTGGCGGCGCTG |
| SRR1965077 | CTGGGCAGCGCGCCGCTTTATGCGCAGACGAGTGCGGTGCAGCAAAAGCTGGCGGCGCTG |
| SRR1966369 | CTGGGCAGCGCGCCGCTTTATGCGCAGACGAGTGCGGTGCAGCAAAAGCTGGCGGCGCTG |
| SRR1967117 | CTGGGCAGCGCGCCGCTTTATGCGCAGACGAGTGCGGTGCAGCAAAAGCTGGCGGCGCTG |
| SRR1967922 | CTGGGCAGCGCGCCGCTTTATGCGCAGACGAGTGCGGTGCAGCAAAAGCTGGCGGCGCTG |
| SRR5583183 | CTGGGCAGCGCGCCGCTTTATGCGCAGACGAGTGCGGTGCAGCAAAAGCTGGCGGCGCTG |
| SRR5585240 | CTGGGCAGCGCGCCGCTTTATGCGCAGACGAGTGCGGTGCAGCAAAAGCTGGCGGCGCTG |
| SRR7216071 | CTGGGCAGCGCGCCGCTTTATGCGCAGACGAGTGCGGTGCAGCAAAAGCTGGCGGCGCTG |
| SRR7277793 | CTGGGCAGCGCGCCGCTTTATGCGCAGACGAGTGCGGTGCAGCAAAAGCTGGCGGCGCTG |
| SRR7284317 | CTGGGCAGCGCGCCGCTTTATGCGCAGACGAGTGCGGTGCAGCAAAAGCTGGCGGCGCTG |
| SRR7299161 | CTGGGCAGCGCGCCGCTTTATGCGCAGACGAGTGCGGTGCAGCAAAAGCTGGCGGCGCTG |
| SRR7343877 | CTGGGCAGCGCGCCGCTTTATGCGCAGACGAGTGCGGTGCAGCAAAAGCTGGCGGCGCTG |
| SRR7351477 | CTGGGCAGCGCGCCGCTTTATGCGCAGACGAGTGCGGTGCAGCAAAAGCTGGCGGCGCTG |
| SRR7401730 | CTGGGCAGCGCGCCGCTTTATGCGCAGACGAGTGCGGTGCAGCAAAAGCTGGCGGCGCTG |
| SRR7469092 | CTGGGCAGCGCGCCGCTTTATGCGCAGACGAGTGCGGTGCAGCAAAAGCTGGCGGCGCTG |
| SRR7523148 | CTGGGCAGCGCGCCGCTTTATGCGCAGACGAGTGCGGTGCAGCAAAAGCTGGCGGCGCTG |
| SRR7523854 | CTGGGCAGCGCGCCGCTTTATGCGCAGACGAGTGCGGTGCAGCAAAAGCTGGCGGCGCTG |
| SRR7842487 | CTGGGCAGCGCGCCGCTTTATGCGCAGACGAGTGCGGTGCAGCAAAAGCTGGCGGCGCTG |
| SRR7879556 | CTGGGCAGCGCGCCGCTTTATGCGCAGACGAGTGCGGTGCAGCAAAAGCTGGCGGCGCTG |
| SRR8054524 | CTGGGCAGCGCGCCGCTTTATGCGCAGACGAGTGCGGTGCAGCAAAAGCTGGCGGCGCTG |
| SRR8054525 | CTGGGCAGCGCGCCGCTTTATGCGCAGACGAGTGCGGTGCAGCAAAAGCTGGCGGCGCTG |
| SRR8524733 | CTGGGCAGCGCGCCGCTTTATGCGCAGACGAGTGCGGTGCAGCAAAAGCTGGCGGCGCTG |
| SRR8526100 | CTGGGCAGCGCGCCGCTTTATGCGCAGACGAGTGCGGTGCAGCAAAAGCTGGCGGCGCTG |
| SRR8553991 | CTGGGCAGCGCGCCGCTTTATGCGCAGACGAGTGCGGTGCAGCAAAAGCTGGCGGCGCTG |
| blaCTX-M-15_1_AY044436 | TTAGGAAGTGTGCCGCTGTATGCGCAAACGGCGGACGTACAGCAAAAACTTGCCGAATTA |
| RIVM_H_2017-15 | TTAGGAAGTGTGCCGCTGTATGCGCAAACGGCGGACGTACAGCAAAAACTTGCCGAATTA |
| RKI_17-06869 | TTAGGAAGTGTGCCGCTGTATGCGCAAACGGCGGACGTACAGCAAAAACTTGCCGAATTA |
| blaCTX-M-55_1_DQ810789 | TTAGGAAGTGTGCCGCTGTATGCGCAAACGGCGGACGTACAGCAAAAACTTGCCGAATTA |
| SRR7349175 | TTAGGAAGTGTGCCGCTGTATGCGCAAACGGCGGACGTACAGCAAAAACTTGCCGAATTA |
| ------------------------------------------------------------------------------------ | |
| blaCTX-M-104_1_HQ833652 | GAGAAAAGCAGCGGAGGGCGGCTGGGCGTCGCGCTCATCGATACCGCAGATAATACGCAG |
| S18BD05011 | GAGAAAAGCAGCGGAGGGCGGCTGGGCGTCGCGCTCATCGATACCGCAGATAATACGCAG |
| blaCTX-M-14_1_AF252622 | GAGAAAAGCAGCGGAGGGCGGCTGGGCGTCGCGCTCATCGATACCGCAGATAATACGCAG |
| blaCTX-M-14b_1_DQ359215 | GAGAAAAGCAGCGGAGGGCGGCTGGGCGTCGCGCTCATCGATACCGCAGATAATACGCAG |
| 15EP001483 | GAGAAAAGCAGCGGAGGGCGGCTGGGCGTCGCGCTCATCGATACCGCAGATAATACGCAG |
| 17EP002363 | GAGAAAAGCAGCGGAGGGCGGCTGGGCGTCGCGCTCATCGATACCGCAGATAATACGCAG |
| 313865 | GAGAAAAGCAGCGGAGGGCGGCTGGGCGTCGCGCTCATCGATACCGCAGATAATACGCAG |
| ERR2173656 | GAGAAAAGCAGCGGAGGGCGGCTGGGCGTCGCGCTCATCGATACCGCAGATAATACGCAG |
| ERR2580274 | GAGAAAAGCAGCGGAGGGCGGCTGGGCGTCGCGCTCATCGATACCGCAGATAATACGCAG |
| ERR2580277 | GAGAAAAGCAGCGGAGGGCGGCTGGGCGTCGCGCTCATCGATACCGCAGATAATACGCAG |
| SRR8704720 | GAGAAAAGCAGCGGAGGGCGGCTGGGCGTCGCGCTCATCGATACCGCAGATAATACGCAG |
| MT16-000061 | GAGAAAAGCAGCGGAGGGCGGCTGGGCGTCGCGCTCATCGATACCGCAGATAATACGCAG |
| MT16-019416 | GAGAAAAGCAGCGGAGGGCGGCTGGGCGTCGCGCTCATCGATACCGCAGATAATACGCAG |
| MT16-027865 | GAGAAAAGCAGCGGAGGGCGGCTGGGCGTCGCGCTCATCGATACCGCAGATAATACGCAG |
| MT16-031693 | GAGAAAAGCAGCGGAGGGCGGCTGGGCGTCGCGCTCATCGATACCGCAGATAATACGCAG |
| MT16-040253 | GAGAAAAGCAGCGGAGGGCGGCTGGGCGTCGCGCTCATCGATACCGCAGATAATACGCAG |
| MT16-045379 | GAGAAAAGCAGCGGAGGGCGGCTGGGCGTCGCGCTCATCGATACCGCAGATAATACGCAG |
| MT16-442728 | GAGAAAAGCAGCGGAGGGCGGCTGGGCGTCGCGCTCATCGATACCGCAGATAATACGCAG |
| MT16-462857 | GAGAAAAGCAGCGGAGGGCGGCTGGGCGTCGCGCTCATCGATACCGCAGATAATACGCAG |
| MT16-480196 | GAGAAAAGCAGCGGAGGGCGGCTGGGCGTCGCGCTCATCGATACCGCAGATAATACGCAG |
| MT16-861555 | GAGAAAAGCAGCGGAGGGCGGCTGGGCGTCGCGCTCATCGATACCGCAGATAATACGCAG |
| MT17-076833 | GAGAAAAGCAGCGGAGGGCGGCTGGGCGTCGCGCTCATCGATACCGCAGATAATACGCAG |
| MT17-110677 | GAGAAAAGCAGCGGAGGGCGGCTGGGCGTCGCGCTCATCGATACCGCAGATAATACGCAG |
| MT17-131730 | GAGAAAAGCAGCGGAGGGCGGCTGGGCGTCGCGCTCATCGATACCGCAGATAATACGCAG |
| MT17-140890 | GAGAAAAGCAGCGGAGGGCGGCTGGGCGTCGCGCTCATCGATACCGCAGATAATACGCAG |
| MT17-141840 | GAGAAAAGCAGCGGAGGGCGGCTGGGCGTCGCGCTCATCGATACCGCAGATAATACGCAG |
| MT17-152488 | GAGAAAAGCAGCGGAGGGCGGCTGGGCGTCGCGCTCATCGATACCGCAGATAATACGCAG |
| MT17-157311 | GAGAAAAGCAGCGGAGGGCGGCTGGGCGTCGCGCTCATCGATACCGCAGATAATACGCAG |
| MT17-161645 | GAGAAAAGCAGCGGAGGGCGGCTGGGCGTCGCGCTCATCGATACCGCAGATAATACGCAG |
| MT17-167951 | GAGAAAAGCAGCGGAGGGCGGCTGGGCGTCGCGCTCATCGATACCGCAGATAATACGCAG |
| MT18-217732 | GAGAAAAGCAGCGGAGGGCGGCTGGGCGTCGCGCTCATCGATACCGCAGATAATACGCAG |
| MT18-252580 | GAGAAAAGCAGCGGAGGGCGGCTGGGCGTCGCGCTCATCGATACCGCAGATAATACGCAG |
| RIVM_H_2016-04 | GAGAAAAGCAGCGGAGGGCGGCTGGGCGTCGCGCTCATCGATACCGCAGATAATACGCAG |
| RIVM_H_2016-07 | GAGAAAAGCAGCGGAGGGCGGCTGGGCGTCGCGCTCATCGATACCGCAGATAATACGCAG |
| RIVM_H_2016-08 | GAGAAAAGCAGCGGAGGGCGGCTGGGCGTCGCGCTCATCGATACCGCAGATAATACGCAG |
| RIVM_H_2016-09 | GAGAAAAGCAGCGGAGGGCGGCTGGGCGTCGCGCTCATCGATACCGCAGATAATACGCAG |
| RIVM_H_2016-11 | GAGAAAAGCAGCGGAGGGCGGCTGGGCGTCGCGCTCATCGATACCGCAGATAATACGCAG |
| RIVM_H_2016-12 | GAGAAAAGCAGCGGAGGGCGGCTGGGCGTCGCGCTCATCGATACCGCAGATAATACGCAG |
| RIVM_H_2016-13 | GAGAAAAGCAGCGGAGGGCGGCTGGGCGTCGCGCTCATCGATACCGCAGATAATACGCAG |
| RIVM_H_2016-14 | GAGAAAAGCAGCGGAGGGCGGCTGGGCGTCGCGCTCATCGATACCGCAGATAATACGCAG |
| RIVM_H_2016-15 | GAGAAAAGCAGCGGAGGGCGGCTGGGCGTCGCGCTCATCGATACCGCAGATAATACGCAG |
| RIVM_H_2017-02 | GAGAAAAGCAGCGGAGGGCGGCTGGGCGTCGCGCTCATCGATACCGCAGATAATACGCAG |
| RIVM_H_2017-03 | GAGAAAAGCAGCGGAGGGCGGCTGGGCGTCGCGCTCATCGATACCGCAGATAATACGCAG |
| RIVM_H_2017-05 | GAGAAAAGCAGCGGAGGGCGGCTGGGCGTCGCGCTCATCGATACCGCAGATAATACGCAG |
| RIVM_H_2017-07 | GAGAAAAGCAGCGGAGGGCGGCTGGGCGTCGCGCTCATCGATACCGCAGATAATACGCAG |
| RIVM_H_2017-08 | GAGAAAAGCAGCGGAGGGCGGCTGGGCGTCGCGCTCATCGATACCGCAGATAATACGCAG |
| RIVM_H_2017-09 | GAGAAAAGCAGCGGAGGGCGGCTGGGCGTCGCGCTCATCGATACCGCAGATAATACGCAG |
| RIVM_H_2017-10 | GAGAAAAGCAGCGGAGGGCGGCTGGGCGTCGCGCTCATCGATACCGCAGATAATACGCAG |
| RIVM_H_2017-11 | GAGAAAAGCAGCGGAGGGCGGCTGGGCGTCGCGCTCATCGATACCGCAGATAATACGCAG |
| RIVM_H_2017-12 | GAGAAAAGCAGCGGAGGGCGGCTGGGCGTCGCGCTCATCGATACCGCAGATAATACGCAG |
| RIVM_H_2017-13 | GAGAAAAGCAGCGGAGGGCGGCTGGGCGTCGCGCTCATCGATACCGCAGATAATACGCAG |
| RIVM_H_2017-14 | GAGAAAAGCAGCGGAGGGCGGCTGGGCGTCGCGCTCATCGATACCGCAGATAATACGCAG |
| RIVM_H_2017-19 | GAGAAAAGCAGCGGAGGGCGGCTGGGCGTCGCGCTCATCGATACCGCAGATAATACGCAG |
| RKI_16-04315 | GAGAAAAGCAGCGGAGGGCGGCTGGGCGTCGCGCTCATCGATACCGCAGATAATACGCAG |
| RKI_17-02304 | GAGAAAAGCAGCGGAGGGCGGCTGGGCGTCGCGCTCATCGATACCGCAGATAATACGCAG |
| RKI_17-02411 | GAGAAAAGCAGCGGAGGGCGGCTGGGCGTCGCGCTCATCGATACCGCAGATAATACGCAG |
| RKI_17-02757 | GAGAAAAGCAGCGGAGGGCGGCTGGGCGTCGCGCTCATCGATACCGCAGATAATACGCAG |
| RKI_17-04797 | GAGAAAAGCAGCGGAGGGCGGCTGGGCGTCGCGCTCATCGATACCGCAGATAATACGCAG |
| S16BD08730 | GAGAAAAGCAGCGGAGGGCGGCTGGGCGTCGCGCTCATCGATACCGCAGATAATACGCAG |
| S18BD03994 | GAGAAAAGCAGCGGAGGGCGGCTGGGCGTCGCGCTCATCGATACCGCAGATAATACGCAG |
| SRR1957844 | GAGAAAAGCAGCGGAGGGCGGCTGGGCGTCGCGCTCATCGATACCGCAGATAATACGCAG |
| SRR1958654 | GAGAAAAGCAGCGGAGGGCGGCTGGGCGTCGCGCTCATCGATACCGCAGATAATACGCAG |
| SRR1965077 | GAGAAAAGCAGCGGAGGGCGGCTGGGCGTCGCGCTCATCGATACCGCAGATAATACGCAG |
| SRR1966369 | GAGAAAAGCAGCGGAGGGCGGCTGGGCGTCGCGCTCATCGATACCGCAGATAATACGCAG |
| SRR1967117 | GAGAAAAGCAGCGGAGGGCGGCTGGGCGTCGCGCTCATCGATACCGCAGATAATACGCAG |
| SRR1967922 | GAGAAAAGCAGCGGAGGGCGGCTGGGCGTCGCGCTCATCGATACCGCAGATAATACGCAG |
| SRR5583183 | GAGAAAAGCAGCGGAGGGCGGCTGGGCGTCGCGCTCATCGATACCGCAGATAATACGCAG |
| SRR5585240 | GAGAAAAGCAGCGGAGGGCGGCTGGGCGTCGCGCTCATCGATACCGCAGATAATACGCAG |
| SRR7216071 | GAGAAAAGCAGCGGAGGGCGGCTGGGCGTCGCGCTCATCGATACCGCAGATAATACGCAG |
| SRR7277793 | GAGAAAAGCAGCGGAGGGCGGCTGGGCGTCGCGCTCATCGATACCGCAGATAATACGCAG |
| SRR7284317 | GAGAAAAGCAGCGGAGGGCGGCTGGGCGTCGCGCTCATCGATACCGCAGATAATACGCAG |
| SRR7299161 | GAGAAAAGCAGCGGAGGGCGGCTGGGCGTCGCGCTCATCGATACCGCAGATAATACGCAG |
| SRR7343877 | GAGAAAAGCAGCGGAGGGCGGCTGGGCGTCGCGCTCATCGATACCGCAGATAATACGCAG |
| SRR7351477 | GAGAAAAGCAGCGGAGGGCGGCTGGGCGTCGCGCTCATCGATACCGCAGATAATACGCAG |
| SRR7401730 | GAGAAAAGCAGCGGAGGGCGGCTGGGCGTCGCGCTCATCGATACCGCAGATAATACGCAG |
| SRR7469092 | GAGAAAAGCAGCGGAGGGCGGCTGGGCGTCGCGCTCATCGATACCGCAGATAATACGCAG |
| SRR7523148 | GAGAAAAGCAGCGGAGGGCGGCTGGGCGTCGCGCTCATCGATACCGCAGATAATACGCAG |
| SRR7523854 | GAGAAAAGCAGCGGAGGGCGGCTGGGCGTCGCGCTCATCGATACCGCAGATAATACGCAG |
| SRR7842487 | GAGAAAAGCAGCGGAGGGCGGCTGGGCGTCGCGCTCATCGATACCGCAGATAATACGCAG |
| SRR7879556 | GAGAAAAGCAGCGGAGGGCGGCTGGGCGTCGCGCTCATCGATACCGCAGATAATACGCAG |
| SRR8054524 | GAGAAAAGCAGCGGAGGGCGGCTGGGCGTCGCGCTCATCGATACCGCAGATAATACGCAG |
| SRR8054525 | GAGAAAAGCAGCGGAGGGCGGCTGGGCGTCGCGCTCATCGATACCGCAGATAATACGCAG |
| SRR8524733 | GAGAAAAGCAGCGGAGGGCGGCTGGGCGTCGCGCTCATCGATACCGCAGATAATACGCAG |
| SRR8526100 | GAGAAAAGCAGCGGAGGGCGGCTGGGCGTCGCGCTCATCGATACCGCAGATAATACGCAG |
| SRR8553991 | GAGAAAAGCAGCGGAGGGCGGCTGGGCGTCGCGCTCATCGATACCGCAGATAATACGCAG |
| blaCTX-M-15_1_AY044436 | GAGCGGCAGTCGGGAGGCAGACTGGGTGTGGCATTGATTAACACAGCAGATAATTCGCAA |
| RIVM_H_2017-15 | GAGCGGCAGTCGGGAGGCAGACTGGGTGTGGCATTGATTAACACAGCAGATAATTCGCAA |
| RKI_17-06869 | GAGCGGCAGTCGGGAGGCAGACTGGGTGTGGCATTGATTAACACAGCAGATAATTCGCAA |
| blaCTX-M-55_1_DQ810789 | GAGCGGCAGTCGGGAGGCAGACTGGGTGTGGCATTGATTAACACAGCAGATAATTCGCAA |
| SRR7349175 | GAGCGGCAGTCGGGAGGCAGACTGGGTGTGGCATTGATTAACACAGCAGATAATTCGCAA |
| ------------------------------------------------------------------------------------ | |
| blaCTX-M-104_1_HQ833652 | GTGCTTTATCGCGGTGATGAACGCTTTCCAATGTGCAGTACCAGTAAAGTTATGGCGGCC |
| S18BD05011 | GTGCTTTATCGCGGTGATGAACGCTTTCCAATGTGCAGTACCAGTAAAGTTATGGCGGCC |
| blaCTX-M-14_1_AF252622 | GTGCTTTATCGCGGTGATGAACGCTTTCCAATGTGCAGTACCAGTAAAGTTATGGCGGCC |
| blaCTX-M-14b_1_DQ359215 | GTGCTTTATCGCGGTGATGAACGCTTTCCAATGTGCAGTACCAGTAAAGTTATGGCGGCC |
| 15EP001483 | GTGCTTTATCGCGGTGATGAACGCTTTCCAATGTGCAGTACCAGTAAAGTTATGGCGGCC |
| 17EP002363 | GTGCTTTATCGCGGTGATGAACGCTTTCCAATGTGCAGTACCAGTAAAGTTATGGCGGCC |
| 313865 | GTGCTTTATCGCGGTGATGAACGCTTTCCAATGTGCAGTACCAGTAAAGTTATGGCGGCC |
| ERR2173656 | GTGCTTTATCGCGGTGATGAACGCTTTCCAATGTGCAGTACCAGTAAAGTTATGGCGGCC |
| ERR2580274 | GTGCTTTATCGCGGTGATGAACGCTTTCCAATGTGCAGTACCAGTAAAGTTATGGCGGCC |
| ERR2580277 | GTGCTTTATCGCGGTGATGAACGCTTTCCAATGTGCAGTACCAGTAAAGTTATGGCGGCC |
| SRR8704720 | GTGCTTTATCGCGGTGATGAACGCTTTCCAATGTGCAGTACCAGTAAAGTTATGGCGGCC |
| MT16-000061 | GTGCTTTATCGCGGTGATGAACGCTTTCCAATGTGCAGTACCAGTAAAGTTATGGCGGCC |
| MT16-019416 | GTGCTTTATCGCGGTGATGAACGCTTTCCAATGTGCAGTACCAGTAAAGTTATGGCGGCC |
| MT16-027865 | GTGCTTTATCGCGGTGATGAACGCTTTCCAATGTGCAGTACCAGTAAAGTTATGGCGGCC |
| MT16-031693 | GTGCTTTATCGCGGTGATGAACGCTTTCCAATGTGCAGTACCAGTAAAGTTATGGCGGCC |
| MT16-040253 | GTGCTTTATCGCGGTGATGAACGCTTTCCAATGTGCAGTACCAGTAAAGTTATGGCGGCC |
| MT16-045379 | GTGCTTTATCGCGGTGATGAACGCTTTCCAATGTGCAGTACCAGTAAAGTTATGGCGGCC |
| MT16-442728 | GTGCTTTATCGCGGTGATGAACGCTTTCCAATGTGCAGTACCAGTAAAGTTATGGCGGCC |
| MT16-462857 | GTGCTTTATCGCGGTGATGAACGCTTTCCAATGTGCAGTACCAGTAAAGTTATGGCGGCC |
| MT16-480196 | GTGCTTTATCGCGGTGATGAACGCTTTCCAATGTGCAGTACCAGTAAAGTTATGGCGGCC |
| MT16-861555 | GTGCTTTATCGCGGTGATGAACGCTTTCCAATGTGCAGTACCAGTAAAGTTATGGCGGCC |
| MT17-076833 | GTGCTTTATCGCGGTGATGAACGCTTTCCAATGTGCAGTACCAGTAAAGTTATGGCGGCC |
| MT17-110677 | GTGCTTTATCGCGGTGATGAACGCTTTCCAATGTGCAGTACCAGTAAAGTTATGGCGGCC |
| MT17-131730 | GTGCTTTATCGCGGTGATGAACGCTTTCCAATGTGCAGTACCAGTAAAGTTATGGCGGCC |
| MT17-140890 | GTGCTTTATCGCGGTGATGAACGCTTTCCAATGTGCAGTACCAGTAAAGTTATGGCGGCC |
| MT17-141840 | GTGCTTTATCGCGGTGATGAACGCTTTCCAATGTGCAGTACCAGTAAAGTTATGGCGGCC |
| MT17-152488 | GTGCTTTATCGCGGTGATGAACGCTTTCCAATGTGCAGTACCAGTAAAGTTATGGCGGCC |
| MT17-157311 | GTGCTTTATCGCGGTGATGAACGCTTTCCAATGTGCAGTACCAGTAAAGTTATGGCGGCC |
| MT17-161645 | GTGCTTTATCGCGGTGATGAACGCTTTCCAATGTGCAGTACCAGTAAAGTTATGGCGGCC |
| MT17-167951 | GTGCTTTATCGCGGTGATGAACGCTTTCCAATGTGCAGTACCAGTAAAGTTATGGCGGCC |
| MT18-217732 | GTGCTTTATCGCGGTGATGAACGCTTTCCAATGTGCAGTACCAGTAAAGTTATGGCGGCC |
| MT18-252580 | GTGCTTTATCGCGGTGATGAACGCTTTCCAATGTGCAGTACCAGTAAAGTTATGGCGGCC |
| RIVM_H_2016-04 | GTGCTTTATCGCGGTGATGAACGCTTTCCAATGTGCAGTACCAGTAAAGTTATGGCGGCC |
| RIVM_H_2016-07 | GTGCTTTATCGCGGTGATGAACGCTTTCCAATGTGCAGTACCAGTAAAGTTATGGCGGCC |
| RIVM_H_2016-08 | GTGCTTTATCGCGGTGATGAACGCTTTCCAATGTGCAGTACCAGTAAAGTTATGGCGGCC |
| RIVM_H_2016-09 | GTGCTTTATCGCGGTGATGAACGCTTTCCAATGTGCAGTACCAGTAAAGTTATGGCGGCC |
| RIVM_H_2016-11 | GTGCTTTATCGCGGTGATGAACGCTTTCCAATGTGCAGTACCAGTAAAGTTATGGCGGCC |
| RIVM_H_2016-12 | GTGCTTTATCGCGGTGATGAACGCTTTCCAATGTGCAGTACCAGTAAAGTTATGGCGGCC |
| RIVM_H_2016-13 | GTGCTTTATCGCGGTGATGAACGCTTTCCAATGTGCAGTACCAGTAAAGTTATGGCGGCC |
| RIVM_H_2016-14 | GTGCTTTATCGCGGTGATGAACGCTTTCCAATGTGCAGTACCAGTAAAGTTATGGCGGCC |
| RIVM_H_2016-15 | GTGCTTTATCGCGGTGATGAACGCTTTCCAATGTGCAGTACCAGTAAAGTTATGGCGGCC |
| RIVM_H_2017-02 | GTGCTTTATCGCGGTGATGAACGCTTTCCAATGTGCAGTACCAGTAAAGTTATGGCGGCC |
| RIVM_H_2017-03 | GTGCTTTATCGCGGTGATGAACGCTTTCCAATGTGCAGTACCAGTAAAGTTATGGCGGCC |
| RIVM_H_2017-05 | GTGCTTTATCGCGGTGATGAACGCTTTCCAATGTGCAGTACCAGTAAAGTTATGGCGGCC |
| RIVM_H_2017-07 | GTGCTTTATCGCGGTGATGAACGCTTTCCAATGTGCAGTACCAGTAAAGTTATGGCGGCC |
| RIVM_H_2017-08 | GTGCTTTATCGCGGTGATGAACGCTTTCCAATGTGCAGTACCAGTAAAGTTATGGCGGCC |
| RIVM_H_2017-09 | GTGCTTTATCGCGGTGATGAACGCTTTCCAATGTGCAGTACCAGTAAAGTTATGGCGGCC |
| RIVM_H_2017-10 | GTGCTTTATCGCGGTGATGAACGCTTTCCAATGTGCAGTACCAGTAAAGTTATGGCGGCC |
| RIVM_H_2017-11 | GTGCTTTATCGCGGTGATGAACGCTTTCCAATGTGCAGTACCAGTAAAGTTATGGCGGCC |
| RIVM_H_2017-12 | GTGCTTTATCGCGGTGATGAACGCTTTCCAATGTGCAGTACCAGTAAAGTTATGGCGGCC |
| RIVM_H_2017-13 | GTGCTTTATCGCGGTGATGAACGCTTTCCAATGTGCAGTACCAGTAAAGTTATGGCGGCC |
| RIVM_H_2017-14 | GTGCTTTATCGCGGTGATGAACGCTTTCCAATGTGCAGTACCAGTAAAGTTATGGCGGCC |
| RIVM_H_2017-19 | GTGCTTTATCGCGGTGATGAACGCTTTCCAATGTGCAGTACCAGTAAAGTTATGGCGGCC |
| RKI_16-04315 | GTGCTTTATCGCGGTGATGAACGCTTTCCAATGTGCAGTACCAGTAAAGTTATGGCGGCC |
| RKI_17-02304 | GTGCTTTATCGCGGTGATGAACGCTTTCCAATGTGCAGTACCAGTAAAGTTATGGCGGCC |
| RKI_17-02411 | GTGCTTTATCGCGGTGATGAACGCTTTCCAATGTGCAGTACCAGTAAAGTTATGGCGGCC |
| RKI_17-02757 | GTGCTTTATCGCGGTGATGAACGCTTTCCAATGTGCAGTACCAGTAAAGTTATGGCGGCC |
| RKI_17-04797 | GTGCTTTATCGCGGTGATGAACGCTTTCCAATGTGCAGTACCAGTAAAGTTATGGCGGCC |
| S16BD08730 | GTGCTTTATCGCGGTGATGAACGCTTTCCAATGTGCAGTACCAGTAAAGTTATGGCGGCC |
| S18BD03994 | GTGCTTTATCGCGGTGATGAACGCTTTCCAATGTGCAGTACCAGTAAAGTTATGGCGGCC |
| SRR1957844 | GTGCTTTATCGCGGTGATGAACGCTTTCCAATGTGCAGTACCAGTAAAGTTATGGCGGCC |
| SRR1958654 | GTGCTTTATCGCGGTGATGAACGCTTTCCAATGTGCAGTACCAGTAAAGTTATGGCGGCC |
| SRR1965077 | GTGCTTTATCGCGGTGATGAACGCTTTCCAATGTGCAGTACCAGTAAAGTTATGGCGGCC |
| SRR1966369 | GTGCTTTATCGCGGTGATGAACGCTTTCCAATGTGCAGTACCAGTAAAGTTATGGCGGCC |
| SRR1967117 | GTGCTTTATCGCGGTGATGAACGCTTTCCAATGTGCAGTACCAGTAAAGTTATGGCGGCC |
| SRR1967922 | GTGCTTTATCGCGGTGATGAACGCTTTCCAATGTGCAGTACCAGTAAAGTTATGGCGGCC |
| SRR5583183 | GTGCTTTATCGCGGTGATGAACGCTTTCCAATGTGCAGTACCAGTAAAGTTATGGCGGCC |
| SRR5585240 | GTGCTTTATCGCGGTGATGAACGCTTTCCAATGTGCAGTACCAGTAAAGTTATGGCGGCC |
| SRR7216071 | GTGCTTTATCGCGGTGATGAACGCTTTCCAATGTGCAGTACCAGTAAAGTTATGGCGGCC |
| SRR7277793 | GTGCTTTATCGCGGTGATGAACGCTTTCCAATGTGCAGTACCAGTAAAGTTATGGCGGCC |
| SRR7284317 | GTGCTTTATCGCGGTGATGAACGCTTTCCAATGTGCAGTACCAGTAAAGTTATGGCGGCC |
| SRR7299161 | GTGCTTTATCGCGGTGATGAACGCTTTCCAATGTGCAGTACCAGTAAAGTTATGGCGGCC |
| SRR7343877 | GTGCTTTATCGCGGTGATGAACGCTTTCCAATGTGCAGTACCAGTAAAGTTATGGCGGCC |
| SRR7351477 | GTGCTTTATCGCGGTGATGAACGCTTTCCAATGTGCAGTACCAGTAAAGTTATGGCGGCC |
| SRR7401730 | GTGCTTTATCGCGGTGATGAACGCTTTCCAATGTGCAGTACCAGTAAAGTTATGGCGGCC |
| SRR7469092 | GTGCTTTATCGCGGTGATGAACGCTTTCCAATGTGCAGTACCAGTAAAGTTATGGCGGCC |
| SRR7523148 | GTGCTTTATCGCGGTGATGAACGCTTTCCAATGTGCAGTACCAGTAAAGTTATGGCGGCC |
| SRR7523854 | GTGCTTTATCGCGGTGATGAACGCTTTCCAATGTGCAGTACCAGTAAAGTTATGGCGGCC |
| SRR7842487 | GTGCTTTATCGCGGTGATGAACGCTTTCCAATGTGCAGTACCAGTAAAGTTATGGCGGCC |
| SRR7879556 | GTGCTTTATCGCGGTGATGAACGCTTTCCAATGTGCAGTACCAGTAAAGTTATGGCGGCC |
| SRR8054524 | GTGCTTTATCGCGGTGATGAACGCTTTCCAATGTGCAGTACCAGTAAAGTTATGGCGGCC |
| SRR8054525 | GTGCTTTATCGCGGTGATGAACGCTTTCCAATGTGCAGTACCAGTAAAGTTATGGCGGCC |
| SRR8524733 | GTGCTTTATCGCGGTGATGAACGCTTTCCAATGTGCAGTACCAGTAAAGTTATGGCGGCC |
| SRR8526100 | GTGCTTTATCGCGGTGATGAACGCTTTCCAATGTGCAGTACCAGTAAAGTTATGGCGGCC |
| SRR8553991 | GTGCTTTATCGCGGTGATGAACGCTTTCCAATGTGCAGTACCAGTAAAGTTATGGCGGCC |
| blaCTX-M-15_1_AY044436 | ATACTTTATCGTGCTGATGAGCGCTTTGCGATGTGCAGCACCAGTAAAGTGATGGCCGCG |
| RIVM_H_2017-15 | ATACTTTATCGTGCTGATGAGCGCTTTGCGATGTGCAGCACCAGTAAAGTGATGGCCGCG |
| RKI_17-06869 | ATACTTTATCGTGCTGATGAGCGCTTTGCGATGTGCAGCACCAGTAAAGTGATGGCCGCG |
| blaCTX-M-55_1_DQ810789 | ATACTTTATCGTGCTGATGAGCGCTTTGCGATGTGCAGCACCAGTAAAGTGATGGCCGTG |
| SRR7349175 | ATACTTTATCGTGCTGATGAGCGCTTTGCGATGTGCAGCACCAGTAAAGTGATGGCCGTG |
| ------------------------------------------------------------------------------------ | |
| blaCTX-M-104_1_HQ833652 | GCGGCGGTGCTTAAGCAGAGTGAAACGCAAAAGCAGCTGCTTAATCAGCCTGTCGAGATC |
| S18BD05011 | GCGGCGGTGCTTAAGCAGAGTGAAACGCAAAAGCAGCTGCTTAATCAGCCTGTCGAGATC |
| blaCTX-M-14_1_AF252622 | GCGGCGGTGCTTAAGCAGAGTGAAACGCAAAAGCAGCTGCTTAATCAGCCTGTCGAGATC |
| blaCTX-M-14b_1_DQ359215 | GCGGCGGTGCTTAAGCAGAGTGAAACGCAAAAGCAGCTGCTTAATCAGCCTGTCGAGATC |
| 15EP001483 | GCGGCGGTGCTTAAGCAGAGTGAAACGCAAAAGCAGCTGCTTAATCAGCCTGTCGAGATC |
| 17EP002363 | GCGGCGGTGCTTAAGCAGAGTGAAACGCAAAAGCAGCTGCTTAATCAGCCTGTCGAGATC |
| 313865 | GCGGCGGTGCTTAAGCAGAGTGAAACGCAAAAGCAGCTGCTTAATCAGCCTGTCGAGATC |
| ERR2173656 | GCGGCGGTGCTTAAGCAGAGTGAAACGCAAAAGCAGCTGCTTAATCAGCCTGTCGAGATC |
| ERR2580274 | GCGGCGGTGCTTAAGCAGAGTGAAACGCAAAAGCAGCTGCTTAATCAGCCTGTCGAGATC |
| ERR2580277 | GCGGCGGTGCTTAAGCAGAGTGAAACGCAAAAGCAGCTGCTTAATCAGCCTGTCGAGATC |
| SRR8704720 | GCGGCGGTGCTTAAGCAGAGTGAAACGCAAAAGCAGCTGCTTAATCAGCCTGTCGAGATC |
| MT16-000061 | GCGGCGGTGCTTAAGCAGAGTGAAACGCAAAAGCAGCTGCTTAATCAGCCTGTCGAGATC |
| MT16-019416 | GCGGCGGTGCTTAAGCAGAGTGAAACGCAAAAGCAGCTGCTTAATCAGCCTGTCGAGATC |
| MT16-027865 | GCGGCGGTGCTTAAGCAGAGTGAAACGCAAAAGCAGCTGCTTAATCAGCCTGTCGAGATC |
| MT16-031693 | GCGGCGGTGCTTAAGCAGAGTGAAACGCAAAAGCAGCTGCTTAATCAGCCTGTCGAGATC |
| MT16-040253 | GCGGCGGTGCTTAAGCAGAGTGAAACGCAAAAGCAGCTGCTTAATCAGCCTGTCGAGATC |
| MT16-045379 | GCGGCGGTGCTTAAGCAGAGTGAAACGCAAAAGCAGCTGCTTAATCAGCCTGTCGAGATC |
| MT16-442728 | GCGGCGGTGCTTAAGCAGAGTGAAACGCAAAAGCAGCTGCTTAATCAGCCTGTCGAGATC |
| MT16-462857 | GCGGCGGTGCTTAAGCAGAGTGAAACGCAAAAGCAGCTGCTTAATCAGCCTGTCGAGATC |
| MT16-480196 | GCGGCGGTGCTTAAGCAGAGTGAAACGCAAAAGCAGCTGCTTAATCAGCCTGTCGAGATC |
| MT16-861555 | GCGGCGGTGCTTAAGCAGAGTGAAACGCAAAAGCAGCTGCTTAATCAGCCTGTCGAGATC |
| MT17-076833 | GCGGCGGTGCTTAAGCAGAGTGAAACGCAAAAGCAGCTGCTTAATCAGCCTGTCGAGATC |
| MT17-110677 | GCGGCGGTGCTTAAGCAGAGTGAAACGCAAAAGCAGCTGCTTAATCAGCCTGTCGAGATC |
| MT17-131730 | GCGGCGGTGCTTAAGCAGAGTGAAACGCAAAAGCAGCTGCTTAATCAGCCTGTCGAGATC |
| MT17-140890 | GCGGCGGTGCTTAAGCAGAGTGAAACGCAAAAGCAGCTGCTTAATCAGCCTGTCGAGATC |
| MT17-141840 | GCGGCGGTGCTTAAGCAGAGTGAAACGCAAAAGCAGCTGCTTAATCAGCCTGTCGAGATC |
| MT17-152488 | GCGGCGGTGCTTAAGCAGAGTGAAACGCAAAAGCAGCTGCTTAATCAGCCTGTCGAGATC |
| MT17-157311 | GCGGCGGTGCTTAAGCAGAGTGAAACGCAAAAGCAGCTGCTTAATCAGCCTGTCGAGATC |
| MT17-161645 | GCGGCGGTGCTTAAGCAGAGTGAAACGCAAAAGCAGCTGCTTAATCAGCCTGTCGAGATC |
| MT17-167951 | GCGGCGGTGCTTAAGCAGAGTGAAACGCAAAAGCAGCTGCTTAATCAGCCTGTCGAGATC |
| MT18-217732 | GCGGCGGTGCTTAAGCAGAGTGAAACGCAAAAGCAGCTGCTTAATCAGCCTGTCGAGATC |
| MT18-252580 | GCGGCGGTGCTTAAGCAGAGTGAAACGCAAAAGCAGCTGCTTAATCAGCCTGTCGAGATC |
| RIVM_H_2016-04 | GCGGCGGTGCTTAAGCAGAGTGAAACGCAAAAGCAGCTGCTTAATCAGCCTGTCGAGATC |
| RIVM_H_2016-07 | GCGGCGGTGCTTAAGCAGAGTGAAACGCAAAAGCAGCTGCTTAATCAGCCTGTCGAGATC |
| RIVM_H_2016-08 | GCGGCGGTGCTTAAGCAGAGTGAAACGCAAAAGCAGCTGCTTAATCAGCCTGTCGAGATC |
| RIVM_H_2016-09 | GCGGCGGTGCTTAAGCAGAGTGAAACGCAAAAGCAGCTGCTTAATCAGCCTGTCGAGATC |
| RIVM_H_2016-11 | GCGGCGGTGCTTAAGCAGAGTGAAACGCAAAAGCAGCTGCTTAATCAGCCTGTCGAGATC |
| RIVM_H_2016-12 | GCGGCGGTGCTTAAGCAGAGTGAAACGCAAAAGCAGCTGCTTAATCAGCCTGTCGAGATC |
| RIVM_H_2016-13 | GCGGCGGTGCTTAAGCAGAGTGAAACGCAAAAGCAGCTGCTTAATCAGCCTGTCGAGATC |
| RIVM_H_2016-14 | GCGGCGGTGCTTAAGCAGAGTGAAACGCAAAAGCAGCTGCTTAATCAGCCTGTCGAGATC |
| RIVM_H_2016-15 | GCGGCGGTGCTTAAGCAGAGTGAAACGCAAAAGCAGCTGCTTAATCAGCCTGTCGAGATC |
| RIVM_H_2017-02 | GCGGCGGTGCTTAAGCAGAGTGAAACGCAAAAGCAGCTGCTTAATCAGCCTGTCGAGATC |
| RIVM_H_2017-03 | GCGGCGGTGCTTAAGCAGAGTGAAACGCAAAAGCAGCTGCTTAATCAGCCTGTCGAGATC |
| RIVM_H_2017-05 | GCGGCGGTGCTTAAGCAGAGTGAAACGCAAAAGCAGCTGCTTAATCAGCCTGTCGAGATC |
| RIVM_H_2017-07 | GCGGCGGTGCTTAAGCAGAGTGAAACGCAAAAGCAGCTGCTTAATCAGCCTGTCGAGATC |
| RIVM_H_2017-08 | GCGGCGGTGCTTAAGCAGAGTGAAACGCAAAAGCAGCTGCTTAATCAGCCTGTCGAGATC |
| RIVM_H_2017-09 | GCGGCGGTGCTTAAGCAGAGTGAAACGCAAAAGCAGCTGCTTAATCAGCCTGTCGAGATC |
| RIVM_H_2017-10 | GCGGCGGTGCTTAAGCAGAGTGAAACGCAAAAGCAGCTGCTTAATCAGCCTGTCGAGATC |
| RIVM_H_2017-11 | GCGGCGGTGCTTAAGCAGAGTGAAACGCAAAAGCAGCTGCTTAATCAGCCTGTCGAGATC |
| RIVM_H_2017-12 | GCGGCGGTGCTTAAGCAGAGTGAAACGCAAAAGCAGCTGCTTAATCAGCCTGTCGAGATC |
| RIVM_H_2017-13 | GCGGCGGTGCTTAAGCAGAGTGAAACGCAAAAGCAGCTGCTTAATCAGCCTGTCGAGATC |
| RIVM_H_2017-14 | GCGGCGGTGCTTAAGCAGAGTGAAACGCAAAAGCAGCTGCTTAATCAGCCTGTCGAGATC |
| RIVM_H_2017-19 | GCGGCGGTGCTTAAGCAGAGTGAAACGCAAAAGCAGCTGCTTAATCAGCCTGTCGAGATC |
| RKI_16-04315 | GCGGCGGTGCTTAAGCAGAGTGAAACGCAAAAGCAGCTGCTTAATCAGCCTGTCGAGATC |
| RKI_17-02304 | GCGGCGGTGCTTAAGCAGAGTGAAACGCAAAAGCAGCTGCTTAATCAGCCTGTCGAGATC |
| RKI_17-02411 | GCGGCGGTGCTTAAGCAGAGTGAAACGCAAAAGCAGCTGCTTAATCAGCCTGTCGAGATC |
| RKI_17-02757 | GCGGCGGTGCTTAAGCAGAGTGAAACGCAAAAGCAGCTGCTTAATCAGCCTGTCGAGATC |
| RKI_17-04797 | GCGGCGGTGCTTAAGCAGAGTGAAACGCAAAAGCAGCTGCTTAATCAGCCTGTCGAGATC |
| S16BD08730 | GCGGCGGTGCTTAAGCAGAGTGAAACGCAAAAGCAGCTGCTTAATCAGCCTGTCGAGATC |
| S18BD03994 | GCGGCGGTGCTTAAGCAGAGTGAAACGCAAAAGCAGCTGCTTAATCAGCCTGTCGAGATC |
| SRR1957844 | GCGGCGGTGCTTAAGCAGAGTGAAACGCAAAAGCAGCTGCTTAATCAGCCTGTCGAGATC |
| SRR1958654 | GCGGCGGTGCTTAAGCAGAGTGAAACGCAAAAGCAGCTGCTTAATCAGCCTGTCGAGATC |
| SRR1965077 | GCGGCGGTGCTTAAGCAGAGTGAAACGCAAAAGCAGCTGCTTAATCAGCCTGTCGAGATC |
| SRR1966369 | GCGGCGGTGCTTAAGCAGAGTGAAACGCAAAAGCAGCTGCTTAATCAGCCTGTCGAGATC |
| SRR1967117 | GCGGCGGTGCTTAAGCAGAGTGAAACGCAAAAGCAGCTGCTTAATCAGCCTGTCGAGATC |
| SRR1967922 | GCGGCGGTGCTTAAGCAGAGTGAAACGCAAAAGCAGCTGCTTAATCAGCCTGTCGAGATC |
| SRR5583183 | GCGGCGGTGCTTAAGCAGAGTGAAACGCAAAAGCAGCTGCTTAATCAGCCTGTCGAGATC |
| SRR5585240 | GCGGCGGTGCTTAAGCAGAGTGAAACGCAAAAGCAGCTGCTTAATCAGCCTGTCGAGATC |
| SRR7216071 | GCGGCGGTGCTTAAGCAGAGTGAAACGCAAAAGCAGCTGCTTAATCAGCCTGTCGAGATC |
| SRR7277793 | GCGGCGGTGCTTAAGCAGAGTGAAACGCAAAAGCAGCTGCTTAATCAGCCTGTCGAGATC |
| SRR7284317 | GCGGCGGTGCTTAAGCAGAGTGAAACGCAAAAGCAGCTGCTTAATCAGCCTGTCGAGATC |
| SRR7299161 | GCGGCGGTGCTTAAGCAGAGTGAAACGCAAAAGCAGCTGCTTAATCAGCCTGTCGAGATC |
| SRR7343877 | GCGGCGGTGCTTAAGCAGAGTGAAACGCAAAAGCAGCTGCTTAATCAGCCTGTCGAGATC |
| SRR7351477 | GCGGCGGTGCTTAAGCAGAGTGAAACGCAAAAGCAGCTGCTTAATCAGCCTGTCGAGATC |
| SRR7401730 | GCGGCGGTGCTTAAGCAGAGTGAAACGCAAAAGCAGCTGCTTAATCAGCCTGTCGAGATC |
| SRR7469092 | GCGGCGGTGCTTAAGCAGAGTGAAACGCAAAAGCAGCTGCTTAATCAGCCTGTCGAGATC |
| SRR7523148 | GCGGCGGTGCTTAAGCAGAGTGAAACGCAAAAGCAGCTGCTTAATCAGCCTGTCGAGATC |
| SRR7523854 | GCGGCGGTGCTTAAGCAGAGTGAAACGCAAAAGCAGCTGCTTAATCAGCCTGTCGAGATC |
| SRR7842487 | GCGGCGGTGCTTAAGCAGAGTGAAACGCAAAAGCAGCTGCTTAATCAGCCTGTCGAGATC |
| SRR7879556 | GCGGCGGTGCTTAAGCAGAGTGAAACGCAAAAGCAGCTGCTTAATCAGCCTGTCGAGATC |
| SRR8054524 | GCGGCGGTGCTTAAGCAGAGTGAAACGCAAAAGCAGCTGCTTAATCAGCCTGTCGAGATC |
| SRR8054525 | GCGGCGGTGCTTAAGCAGAGTGAAACGCAAAAGCAGCTGCTTAATCAGCCTGTCGAGATC |
| SRR8524733 | GCGGCGGTGCTTAAGCAGAGTGAAACGCAAAAGCAGCTGCTTAATCAGCCTGTCGAGATC |
| SRR8526100 | GCGGCGGTGCTTAAGCAGAGTGAAACGCAAAAGCAGCTGCTTAATCAGCCTGTCGAGATC |
| SRR8553991 | GCGGCGGTGCTTAAGCAGAGTGAAACGCAAAAGCAGCTGCTTAATCAGCCTGTCGAGATC |
| blaCTX-M-15_1_AY044436 | GCCGCGGTGCTGAAGAAAAGTGAAAGCGAACCGAATCTGTTAAATCAGCGAGTTGAGATC |
| RIVM_H_2017-15 | GCCGCGGTGCTGAAGAAAAGTGAAAGCGAACCGAATCTGTTAAATCAGCGAGTTGAGATC |
| RKI_17-06869 | GCCGCGGTGCTGAAGAAAAGTGAAAGCGAACCGAATCTGTTAAATCAGCGAGTTGAGATC |
| blaCTX-M-55_1_DQ810789 | GCCGCGGTGCTGAAGAAAAGTGAAAGCGAACCGAATCTGTTAAATCAGCGAGTTGAGATC |
| SRR7349175 | GCCGCGGTGCTGAAGAAAAGTGAAAGCGAACCGAATCTGTTAAATCAGCGAGTTGAGATC |
| ------------------------------------------------------------------------------------ | |
| blaCTX-M-104_1_HQ833652 | AAGCCTGCCGATCTGGTTAACTACAATCCGATTGCCGAAAAACACGTCAACGGCACAATG |
| S18BD05011 | AAGCCTGCCGATCTGGTTAACTACAATCCGATTGCCGAAAAACACGTCAACGGCACAATG |
| blaCTX-M-14_1_AF252622 | AAGCCTGCCGATCTGGTTAACTACAATCCGATTGCCGAAAAACACGTCAACGGCACAATG |
| blaCTX-M-14b_1_DQ359215 | AAGCCTGCCGATCTGGTTAACTACAATCCGATTGCCGAAAAACACGTCAACGGCACAATG |
| 15EP001483 | AAGCCTGCCGATCTGGTTAACTACAATCCGATTGCCGAAAAACACGTCAACGGCACAATG |
| 17EP002363 | AAGCCTGCCGATCTGGTTAACTACAATCCGATTGCCGAAAAACACGTCAACGGCACAATG |
| 313865 | AAGCCTGCCGATCTGGTTAACTACAATCCGATTGCCGAAAAACACGTCAACGGCACAATG |
| ERR2173656 | AAGCCTGCCGATCTGGTTAACTACAATCCGATTGCCGAAAAACACGTCAACGGCACAATG |
| ERR2580274 | AAGCCTGCCGATCTGGTTAACTACAATCCGATTGCCGAAAAACACGTCAACGGCACAATG |
| ERR2580277 | AAGCCTGCCGATCTGGTTAACTACAATCCGATTGCCGAAAAACACGTCAACGGCACAATG |
| SRR8704720 | AAGCCTGCCGATCTGGTTAACTACAATCCGATTGCCGAAAAACACGTCAACGGCACAATG |
| MT16-000061 | AAGCCTGCCGATCTGGTTAACTACAATCCGATTGCCGAAAAACACGTCAACGGCACAATG |
| MT16-019416 | AAGCCTGCCGATCTGGTTAACTACAATCCGATTGCCGAAAAACACGTCAACGGCACAATG |
| MT16-027865 | AAGCCTGCCGATCTGGTTAACTACAATCCGATTGCCGAAAAACACGTCAACGGCACAATG |
| MT16-031693 | AAGCCTGCCGATCTGGTTAACTACAATCCGATTGCCGAAAAACACGTCAACGGCACAATG |
| MT16-040253 | AAGCCTGCCGATCTGGTTAACTACAATCCGATTGCCGAAAAACACGTCAACGGCACAATG |
| MT16-045379 | AAGCCTGCCGATCTGGTTAACTACAATCCGATTGCCGAAAAACACGTCAACGGCACAATG |
| MT16-442728 | AAGCCTGCCGATCTGGTTAACTACAATCCGATTGCCGAAAAACACGTCAACGGCACAATG |
| MT16-462857 | AAGCCTGCCGATCTGGTTAACTACAATCCGATTGCCGAAAAACACGTCAACGGCACAATG |
| MT16-480196 | AAGCCTGCCGATCTGGTTAACTACAATCCGATTGCCGAAAAACACGTCAACGGCACAATG |
| MT16-861555 | AAGCCTGCCGATCTGGTTAACTACAATCCGATTGCCGAAAAACACGTCAACGGCACAATG |
| MT17-076833 | AAGCCTGCCGATCTGGTTAACTACAATCCGATTGCCGAAAAACACGTCAACGGCACAATG |
| MT17-110677 | AAGCCTGCCGATCTGGTTAACTACAATCCGATTGCCGAAAAACACGTCAACGGCACAATG |
| MT17-131730 | AAGCCTGCCGATCTGGTTAACTACAATCCGATTGCCGAAAAACACGTCAACGGCACAATG |
| MT17-140890 | AAGCCTGCCGATCTGGTTAACTACAATCCGATTGCCGAAAAACACGTCAACGGCACAATG |
| MT17-141840 | AAGCCTGCCGATCTGGTTAACTACAATCCGATTGCCGAAAAACACGTCAACGGCACAATG |
| MT17-152488 | AAGCCTGCCGATCTGGTTAACTACAATCCGATTGCCGAAAAACACGTCAACGGCACAATG |
| MT17-157311 | AAGCCTGCCGATCTGGTTAACTACAATCCGATTGCCGAAAAACACGTCAACGGCACAATG |
| MT17-161645 | AAGCCTGCCGATCTGGTTAACTACAATCCGATTGCCGAAAAACACGTCAACGGCACAATG |
| MT17-167951 | AAGCCTGCCGATCTGGTTAACTACAATCCGATTGCCGAAAAACACGTCAACGGCACAATG |
| MT18-217732 | AAGCCTGCCGATCTGGTTAACTACAATCCGATTGCCGAAAAACACGTCAACGGCACAATG |
| MT18-252580 | AAGCCTGCCGATCTGGTTAACTACAATCCGATTGCCGAAAAACACGTCAACGGCACAATG |
| RIVM_H_2016-04 | AAGCCTGCCGATCTGGTTAACTACAATCCGATTGCCGAAAAACACGTCAACGGCACAATG |
| RIVM_H_2016-07 | AAGCCTGCCGATCTGGTTAACTACAATCCGATTGCCGAAAAACACGTCAACGGCACAATG |
| RIVM_H_2016-08 | AAGCCTGCCGATCTGGTTAACTACAATCCGATTGCCGAAAAACACGTCAACGGCACAATG |
| RIVM_H_2016-09 | AAGCCTGCCGATCTGGTTAACTACAATCCGATTGCCGAAAAACACGTCAACGGCACAATG |
| RIVM_H_2016-11 | AAGCCTGCCGATCTGGTTAACTACAATCCGATTGCCGAAAAACACGTCAACGGCACAATG |
| RIVM_H_2016-12 | AAGCCTGCCGATCTGGTTAACTACAATCCGATTGCCGAAAAACACGTCAACGGCACAATG |
| RIVM_H_2016-13 | AAGCCTGCCGATCTGGTTAACTACAATCCGATTGCCGAAAAACACGTCAACGGCACAATG |
| RIVM_H_2016-14 | AAGCCTGCCGATCTGGTTAACTACAATCCGATTGCCGAAAAACACGTCAACGGCACAATG |
| RIVM_H_2016-15 | AAGCCTGCCGATCTGGTTAACTACAATCCGATTGCCGAAAAACACGTCAACGGCACAATG |
| RIVM_H_2017-02 | AAGCCTGCCGATCTGGTTAACTACAATCCGATTGCCGAAAAACACGTCAACGGCACAATG |
| RIVM_H_2017-03 | AAGCCTGCCGATCTGGTTAACTACAATCCGATTGCCGAAAAACACGTCAACGGCACAATG |
| RIVM_H_2017-05 | AAGCCTGCCGATCTGGTTAACTACAATCCGATTGCCGAAAAACACGTCAACGGCACAATG |
| RIVM_H_2017-07 | AAGCCTGCCGATCTGGTTAACTACAATCCGATTGCCGAAAAACACGTCAACGGCACAATG |
| RIVM_H_2017-08 | AAGCCTGCCGATCTGGTTAACTACAATCCGATTGCCGAAAAACACGTCAACGGCACAATG |
| RIVM_H_2017-09 | AAGCCTGCCGATCTGGTTAACTACAATCCGATTGCCGAAAAACACGTCAACGGCACAATG |
| RIVM_H_2017-10 | AAGCCTGCCGATCTGGTTAACTACAATCCGATTGCCGAAAAACACGTCAACGGCACAATG |
| RIVM_H_2017-11 | AAGCCTGCCGATCTGGTTAACTACAATCCGATTGCCGAAAAACACGTCAACGGCACAATG |
| RIVM_H_2017-12 | AAGCCTGCCGATCTGGTTAACTACAATCCGATTGCCGAAAAACACGTCAACGGCACAATG |
| RIVM_H_2017-13 | AAGCCTGCCGATCTGGTTAACTACAATCCGATTGCCGAAAAACACGTCAACGGCACAATG |
| RIVM_H_2017-14 | AAGCCTGCCGATCTGGTTAACTACAATCCGATTGCCGAAAAACACGTCAACGGCACAATG |
| RIVM_H_2017-19 | AAGCCTGCCGATCTGGTTAACTACAATCCGATTGCCGAAAAACACGTCAACGGCACAATG |
| RKI_16-04315 | AAGCCTGCCGATCTGGTTAACTACAATCCGATTGCCGAAAAACACGTCAACGGCACAATG |
| RKI_17-02304 | AAGCCTGCCGATCTGGTTAACTACAATCCGATTGCCGAAAAACACGTCAACGGCACAATG |
| RKI_17-02411 | AAGCCTGCCGATCTGGTTAACTACAATCCGATTGCCGAAAAACACGTCAACGGCACAATG |
| RKI_17-02757 | AAGCCTGCCGATCTGGTTAACTACAATCCGATTGCCGAAAAACACGTCAACGGCACAATG |
| RKI_17-04797 | AAGCCTGCCGATCTGGTTAACTACAATCCGATTGCCGAAAAACACGTCAACGGCACAATG |
| S16BD08730 | AAGCCTGCCGATCTGGTTAACTACAATCCGATTGCCGAAAAACACGTCAACGGCACAATG |
| S18BD03994 | AAGCCTGCCGATCTGGTTAACTACAATCCGATTGCCGAAAAACACGTCAACGGCACAATG |
| SRR1957844 | AAGCCTGCCGATCTGGTTAACTACAATCCGATTGCCGAAAAACACGTCAACGGCACAATG |
| SRR1958654 | AAGCCTGCCGATCTGGTTAACTACAATCCGATTGCCGAAAAACACGTCAACGGCACAATG |
| SRR1965077 | AAGCCTGCCGATCTGGTTAACTACAATCCGATTGCCGAAAAACACGTCAACGGCACAATG |
| SRR1966369 | AAGCCTGCCGATCTGGTTAACTACAATCCGATTGCCGAAAAACACGTCAACGGCACAATG |
| SRR1967117 | AAGCCTGCCGATCTGGTTAACTACAATCCGATTGCCGAAAAACACGTCAACGGCACAATG |
| SRR1967922 | AAGCCTGCCGATCTGGTTAACTACAATCCGATTGCCGAAAAACACGTCAACGGCACAATG |
| SRR5583183 | AAGCCTGCCGATCTGGTTAACTACAATCCGATTGCCGAAAAACACGTCAACGGCACAATG |
| SRR5585240 | AAGCCTGCCGATCTGGTTAACTACAATCCGATTGCCGAAAAACACGTCAACGGCACAATG |
| SRR7216071 | AAGCCTGCCGATCTGGTTAACTACAATCCGATTGCCGAAAAACACGTCAACGGCACAATG |
| SRR7277793 | AAGCCTGCCGATCTGGTTAACTACAATCCGATTGCCGAAAAACACGTCAACGGCACAATG |
| SRR7284317 | AAGCCTGCCGATCTGGTTAACTACAATCCGATTGCCGAAAAACACGTCAACGGCACAATG |
| SRR7299161 | AAGCCTGCCGATCTGGTTAACTACAATCCGATTGCCGAAAAACACGTCAACGGCACAATG |
| SRR7343877 | AAGCCTGCCGATCTGGTTAACTACAATCCGATTGCCGAAAAACACGTCAACGGCACAATG |
| SRR7351477 | AAGCCTGCCGATCTGGTTAACTACAATCCGATTGCCGAAAAACACGTCAACGGCACAATG |
| SRR7401730 | AAGCCTGCCGATCTGGTTAACTACAATCCGATTGCCGAAAAACACGTCAACGGCACAATG |
| SRR7469092 | AAGCCTGCCGATCTGGTTAACTACAATCCGATTGCCGAAAAACACGTCAACGGCACAATG |
| SRR7523148 | AAGCCTGCCGATCTGGTTAACTACAATCCGATTGCCGAAAAACACGTCAACGGCACAATG |
| SRR7523854 | AAGCCTGCCGATCTGGTTAACTACAATCCGATTGCCGAAAAACACGTCAACGGCACAATG |
| SRR7842487 | AAGCCTGCCGATCTGGTTAACTACAATCCGATTGCCGAAAAACACGTCAACGGCACAATG |
| SRR7879556 | AAGCCTGCCGATCTGGTTAACTACAATCCGATTGCCGAAAAACACGTCAACGGCACAATG |
| SRR8054524 | AAGCCTGCCGATCTGGTTAACTACAATCCGATTGCCGAAAAACACGTCAACGGCACAATG |
| SRR8054525 | AAGCCTGCCGATCTGGTTAACTACAATCCGATTGCCGAAAAACACGTCAACGGCACAATG |
| SRR8524733 | AAGCCTGCCGATCTGGTTAACTACAATCCGATTGCCGAAAAACACGTCAACGGCACAATG |
| SRR8526100 | AAGCCTGCCGATCTGGTTAACTACAATCCGATTGCCGAAAAACACGTCAACGGCACAATG |
| SRR8553991 | AAGCCTGCCGATCTGGTTAACTACAATCCGATTGCCGAAAAACACGTCAACGGCACAATG |
| blaCTX-M-15_1_AY044436 | AAAAAATCTGACCTTGTTAACTATAATCCGATTGCGGAAAAGCACGTCAATGGGACGATG |
| RIVM_H_2017-15 | AAAAAATCTGACCTTGTTAACTATAATCCGATTGCGGAAAAGCACGTCAATGGGACGATG |
| RKI_17-06869 | AAAAAATCTGACCTTGTTAACTATAATCCGATTGCGGAAAAGCACGTCAATGGGACGATG |
| blaCTX-M-55_1_DQ810789 | AAAAAATCTGACCTTGTTAACTATAATCCGATTGCGGAAAAGCACGTCAATGGGACGATG |
| SRR7349175 | AAAAAATCTGACCTTGTTAACTATAATCCGATTGCGGAAAAGCACGTCAATGGGACGATG |
| ------------------------------------------------------------------------------------ | |
| blaCTX-M-104_1_HQ833652 | ACGCTGGCAGAACTGAGCGCGGCCGCGTTGCAGTACAGCGACAATACCGCCATGAACAAA |
| S18BD05011 | ACGCTGGCAGAGCTGAGCGCGGCCGCGTTGCAGTACAGCGACAATACCGCCATGAACAAA |
| blaCTX-M-14_1_AF252622 | ACGCTGGCAGAACTGAGCGCGGCCGCGTTGCAGTACAGCGACAATACCGCCATGAACAAA |
| blaCTX-M-14b_1_DQ359215 | ACGCTGGCAGAGCTGAGCGCGGCCGCGTTGCAGTACAGCGACAATACCGCCATGAACAAA |
| 15EP001483 | ACGCTGGCAGAGCTGAGCGCGGCCGCGTTGCAGTACAGCGACAATACCGCCATGAACAAA |
| 17EP002363 | ACGCTGGCAGAGCTGAGCGCGGCCGCGTTGCAGTACAGCGACAATACCGCCATGAACAAA |
| 313865 | ACGCTGGCAGAGCTGAGCGCGGCCGCGTTGCAGTACAGCGACAATACCGCCATGAACAAA |
| ERR2173656 | ACGCTGGCAGAGCTGAGCGCGGCCGCGTTGCAGTACAGCGACAATACCGCCATGAACAAA |
| ERR2580274 | ACGCTGGCAGAGCTGAGCGCGGCCGCGTTGCAGTACAGCGACAATACCGCCATGAACAAA |
| ERR2580277 | ACGCTGGCAGAGCTGAGCGCGGCCGCGTTGCAGTACAGCGACAATACCGCCATGAACAAA |
| SRR8704720 | ACGCTGGCAGAGCTGAGCGCGGCCGCGTTGCAGTACAGCGACAATACCGCCATGAACAAA |
| MT16-000061 | ACGCTGGCAGAGCTGAGCGCGGCCGCGTTGCAGTACAGCGACAATACCGCCATGAACAAA |
| MT16-019416 | ACGCTGGCAGAGCTGAGCGCGGCCGCGTTGCAGTACAGCGACAATACCGCCATGAACAAA |
| MT16-027865 | ACGCTGGCAGAGCTGAGCGCGGCCGCGTTGCAGTACAGCGACAATACCGCCATGAACAAA |
| MT16-031693 | ACGCTGGCAGAGCTGAGCGCGGCCGCGTTGCAGTACAGCGACAATACCGCCATGAACAAA |
| MT16-040253 | ACGCTGGCAGAGCTGAGCGCGGCCGCGTTGCAGTACAGCGACAATACCGCCATGAACAAA |
| MT16-045379 | ACGCTGGCAGAGCTGAGCGCGGCCGCGTTGCAGTACAGCGACAATACCGCCATGAACAAA |
| MT16-442728 | ACGCTGGCAGAGCTGAGCGCGGCCGCGTTGCAGTACAGCGACAATACCGCCATGAACAAA |
| MT16-462857 | ACGCTGGCAGAGCTGAGCGCGGCCGCGTTGCAGTACAGCGACAATACCGCCATGAACAAA |
| MT16-480196 | ACGCTGGCAGAGCTGAGCGCGGCCGCGTTGCAGTACAGCGACAATACCGCCATGAACAAA |
| MT16-861555 | ACGCTGGCAGAGCTGAGCGCGGCCGCGTTGCAGTACAGCGACAATACCGCCATGAACAAA |
| MT17-076833 | ACGCTGGCAGAGCTGAGCGCGGCCGCGTTGCAGTACAGCGACAATACCGCCATGAACAAA |
| MT17-110677 | ACGCTGGCAGAGCTGAGCGCGGCCGCGTTGCAGTACAGCGACAATACCGCCATGAACAAA |
| MT17-131730 | ACGCTGGCAGAGCTGAGCGCGGCCGCGTTGCAGTACAGCGACAATACCGCCATGAACAAA |
| MT17-140890 | ACGCTGGCAGAGCTGAGCGCGGCCGCGTTGCAGTACAGCGACAATACCGCCATGAACAAA |
| MT17-141840 | ACGCTGGCAGAGCTGAGCGCGGCCGCGTTGCAGTACAGCGACAATACCGCCATGAACAAA |
| MT17-152488 | ACGCTGGCAGAGCTGAGCGCGGCCGCGTTGCAGTACAGCGACAATACCGCCATGAACAAA |
| MT17-157311 | ACGCTGGCAGAGCTGAGCGCGGCCGCGTTGCAGTACAGCGACAATACCGCCATGAACAAA |
| MT17-161645 | ACGCTGGCAGAGCTGAGCGCGGCCGCGTTGCAGTACAGCGACAATACCGCCATGAACAAA |
| MT17-167951 | ACGCTGGCAGAGCTGAGCGCGGCCGCGTTGCAGTACAGCGACAATACCGCCATGAACAAA |
| MT18-217732 | ACGCTGGCAGAGCTGAGCGCGGCCGCGTTGCAGTACAGCGACAATACCGCCATGAACAAA |
| MT18-252580 | ACGCTGGCAGAGCTGAGCGCGGCCGCGTTGCAGTACAGCGACAATACCGCCATGAACAAA |
| RIVM_H_2016-04 | ACGCTGGCAGAGCTGAGCGCGGCCGCGTTGCAGTACAGCGACAATACCGCCATGAACAAA |
| RIVM_H_2016-07 | ACGCTGGCAGAGCTGAGCGCGGCCGCGTTGCAGTACAGCGACAATACCGCCATGAACAAA |
| RIVM_H_2016-08 | ACGCTGGCAGAGCTGAGCGCGGCCGCGTTGCAGTACAGCGACAATACCGCCATGAACAAA |
| RIVM_H_2016-09 | ACGCTGGCAGAGCTGAGCGCGGCCGCGTTGCAGTACAGCGACAATACCGCCATGAACAAA |
| RIVM_H_2016-11 | ACGCTGGCAGAGCTGAGCGCGGCCGCGTTGCAGTACAGCGACAATACCGCCATGAACAAA |
| RIVM_H_2016-12 | ACGCTGGCAGAGCTGAGCGCGGCCGCGTTGCAGTACAGCGACAATACCGCCATGAACAAA |
| RIVM_H_2016-13 | ACGCTGGCAGAGCTGAGCGCGGCCGCGTTGCAGTACAGCGACAATACCGCCATGAACAAA |
| RIVM_H_2016-14 | ACGCTGGCAGAGCTGAGCGCGGCCGCGTTGCAGTACAGCGACAATACCGCCATGAACAAA |
| RIVM_H_2016-15 | ACGCTGGCAGAGCTGAGCGCGGCCGCGTTGCAGTACAGCGACAATACCGCCATGAACAAA |
| RIVM_H_2017-02 | ACGCTGGCAGAGCTGAGCGCGGCCGCGTTGCAGTACAGCGACAATACCGCCATGAACAAA |
| RIVM_H_2017-03 | ACGCTGGCAGAGCTGAGCGCGGCCGCGTTGCAGTACAGCGACAATACCGCCATGAACAAA |
| RIVM_H_2017-05 | ACGCTGGCAGAGCTGAGCGCGGCCGCGTTGCAGTACAGCGACAATACCGCCATGAACAAA |
| RIVM_H_2017-07 | ACGCTGGCAGAGCTGAGCGCGGCCGCGTTGCAGTACAGCGACAATACCGCCATGAACAAA |
| RIVM_H_2017-08 | ACGCTGGCAGAGCTGAGCGCGGCCGCGTTGCAGTACAGCGACAATACCGCCATGAACAAA |
| RIVM_H_2017-09 | ACGCTGGCAGAGCTGAGCGCGGCCGCGTTGCAGTACAGCGACAATACCGCCATGAACAAA |
| RIVM_H_2017-10 | ACGCTGGCAGAGCTGAGCGCGGCCGCGTTGCAGTACAGCGACAATACCGCCATGAACAAA |
| RIVM_H_2017-11 | ACGCTGGCAGAGCTGAGCGCGGCCGCGTTGCAGTACAGCGACAATACCGCCATGAACAAA |
| RIVM_H_2017-12 | ACGCTGGCAGAGCTGAGCGCGGCCGCGTTGCAGTACAGCGACAATACCGCCATGAACAAA |
| RIVM_H_2017-13 | ACGCTGGCAGAGCTGAGCGCGGCCGCGTTGCAGTACAGCGACAATACCGCCATGAACAAA |
| RIVM_H_2017-14 | ACGCTGGCAGAGCTGAGCGCGGCCGCGTTGCAGTACAGCGACAATACCGCCATGAACAAA |
| RIVM_H_2017-19 | ACGCTGGCAGAGCTGAGCGCGGCCGCGTTGCAGTACAGCGACAATACCGCCATGAACAAA |
| RKI_16-04315 | ACGCTGGCAGAGCTGAGCGCGGCCGCGTTGCAGTACAGCGACAATACCGCCATGAACAAA |
| RKI_17-02304 | ACGCTGGCAGAGCTGAGCGCGGCCGCGTTGCAGTACAGCGACAATACCGCCATGAACAAA |
| RKI_17-02411 | ACGCTGGCAGAGCTGAGCGCGGCCGCGTTGCAGTACAGCGACAATACCGCCATGAACAAA |
| RKI_17-02757 | ACGCTGGCAGAGCTGAGCGCGGCCGCGTTGCAGTACAGCGACAATACCGCCATGAACAAA |
| RKI_17-04797 | ACGCTGGCAGAGCTGAGCGCGGCCGCGTTGCAGTACAGCGACAATACCGCCATGAACAAA |
| S16BD08730 | ACGCTGGCAGAGCTGAGCGCGGCCGCGTTGCAGTACAGCGACAATACCGCCATGAACAAA |
| S18BD03994 | ACGCTGGCAGAGCTGAGCGCGGCCGCGTTGCAGTACAGCGACAATACCGCCATGAACAAA |
| SRR1957844 | ACGCTGGCAGAGCTGAGCGCGGCCGCGTTGCAGTACAGCGACAATACCGCCATGAACAAA |
| SRR1958654 | ACGCTGGCAGAGCTGAGCGCGGCCGCGTTGCAGTACAGCGACAATACCGCCATGAACAAA |
| SRR1965077 | ACGCTGGCAGAGCTGAGCGCGGCCGCGTTGCAGTACAGCGACAATACCGCCATGAACAAA |
| SRR1966369 | ACGCTGGCAGAGCTGAGCGCGGCCGCGTTGCAGTACAGCGACAATACCGCCATGAACAAA |
| SRR1967117 | ACGCTGGCAGAGCTGAGCGCGGCCGCGTTGCAGTACAGCGACAATACCGCCATGAACAAA |
| SRR1967922 | ACGCTGGCAGAGCTGAGCGCGGCCGCGTTGCAGTACAGCGACAATACCGCCATGAACAAA |
| SRR5583183 | ACGCTGGCAGAGCTGAGCGCGGCCGCGTTGCAGTACAGCGACAATACCGCCATGAACAAA |
| SRR5585240 | ACGCTGGCAGAGCTGAGCGCGGCCGCGTTGCAGTACAGCGACAATACCGCCATGAACAAA |
| SRR7216071 | ACGCTGGCAGAGCTGAGCGCGGCCGCGTTGCAGTACAGCGACAATACCGCCATGAACAAA |
| SRR7277793 | ACGCTGGCAGAGCTGAGCGCGGCCGCGTTGCAGTACAGCGACAATACCGCCATGAACAAA |
| SRR7284317 | ACGCTGGCAGAGCTGAGCGCGGCCGCGTTGCAGTACAGCGACAATACCGCCATGAACAAA |
| SRR7299161 | ACGCTGGCAGAGCTGAGCGCGGCCGCGTTGCAGTACAGCGACAATACCGCCATGAACAAA |
| SRR7343877 | ACGCTGGCAGAGCTGAGCGCGGCCGCGTTGCAGTACAGCGACAATACCGCCATGAACAAA |
| SRR7351477 | ACGCTGGCAGAGCTGAGCGCGGCCGCGTTGCAGTACAGCGACAATACCGCCATGAACAAA |
| SRR7401730 | ACGCTGGCAGAGCTGAGCGCGGCCGCGTTGCAGTACAGCGACAATACCGCCATGAACAAA |
| SRR7469092 | ACGCTGGCAGAGCTGAGCGCGGCCGCGTTGCAGTACAGCGACAATACCGCCATGAACAAA |
| SRR7523148 | ACGCTGGCAGAGCTGAGCGCGGCCGCGTTGCAGTACAGCGACAATACCGCCATGAACAAA |
| SRR7523854 | ACGCTGGCAGAGCTGAGCGCGGCCGCGTTGCAGTACAGCGACAATACCGCCATGAACAAA |
| SRR7842487 | ACGCTGGCAGAGCTGAGCGCGGCCGCGTTGCAGTACAGCGACAATACCGCCATGAACAAA |
| SRR7879556 | ACGCTGGCAGAGCTGAGCGCGGCCGCGTTGCAGTACAGCGACAATACCGCCATGAACAAA |
| SRR8054524 | ACGCTGGCAGAGCTGAGCGCGGCCGCGTTGCAGTACAGCGACAATACCGCCATGAACAAA |
| SRR8054525 | ACGCTGGCAGAGCTGAGCGCGGCCGCGTTGCAGTACAGCGACAATACCGCCATGAACAAA |
| SRR8524733 | ACGCTGGCAGAGCTGAGCGCGGCCGCGTTGCAGTACAGCGACAATACCGCCATGAACAAA |
| SRR8526100 | ACGCTGGCAGAGCTGAGCGCGGCCGCGTTGCAGTACAGCGACAATACCGCCATGAACAAA |
| SRR8553991 | ACGCTGGCAGAGCTGAGCGCGGCCGCGTTGCAGTACAGCGACAATACCGCCATGAACAAA |
| blaCTX-M-15_1_AY044436 | TCACTGGCTGAGCTTAGCGCGGCCGCGCTACAGTACAGCGATAACGTGGCGATGAATAAG |
| RIVM_H_2017-15 | TCACTGGCTGAGCTTAGCGCGGCCGCGCTACAGTACAGCGATAACGTGGCGATGAATAAG |
| RKI_17-06869 | TCACTGGCTGAGCTTAGCGCGGCCGCGCTACAGTACAGCGATAACGTGGCGATGAATAAG |
| blaCTX-M-55_1_DQ810789 | TCACTGGCTGAGCTTAGCGCGGCCGCGCTACAGTACAGCGATAACGTGGCGATGAATAAG |
| SRR7349175 | TCACTGGCTGAGCTTAGCGCGGCCGCGCTACAGTACAGCGATAACGTGGCGATGAATAAG |
| ------------------------------------------------------------------------------------ | |
| blaCTX-M-104_1_HQ833652 | TTGATTGCCCAGCTCGGTGGCCCGGGAGGCGTGACGGCTTTTGCCCGCGCGATCGGCGAT |
| S18BD05011 | TTGATTGCCCAGCTCGGTGGCCCGGGAGGCGTGACGGCTTTTGCCCGCGCGATCGGCGAT |
| blaCTX-M-14_1_AF252622 | TTGATTGCCCAGCTCGGTGGCCCGGGAGGCGTGACGGCTTTTGCCCGCGCGATCGGCGAT |
| blaCTX-M-14b_1_DQ359215 | TTGATTGCCCAGCTCGGTGGCCCGGGAGGCGTGACGGCTTTTGCCCGCGCGATCGGCGAT |
| 15EP001483 | TTGATTGCCCAGCTCGGTGGCCCGGGAGGCGTGACGGCTTTTGCCCGCGCGATCGGCGAT |
| 17EP002363 | TTGATTGCCCAGCTCGGTGGCCCGGGAGGCGTGACGGCTTTTGCCCGCGCGATCGGCGAT |
| 313865 | TTGATTGCCCAGCTCGGTGGCCCGGGAGGCGTGACGGCTTTTGCCCGCGCGATCGGCGAT |
| ERR2173656 | TTGATTGCCCAGCTCGGTGGCCCGGGAGGCGTGACGGCTTTTGCCCGCGCGATCGGCGAT |
| ERR2580274 | TTGATTGCCCAGCTCGGTGGCCCGGGAGGCGTGACGGCTTTTGCCCGCGCGATCGGCGAT |
| ERR2580277 | TTGATTGCCCAGCTCGGTGGCCCGGGAGGCGTGACGGCTTTTGCCCGCGCGATCGGCGAT |
| SRR8704720 | TTGATTGCCCAGCTCGGTGGCCCGGGAGGCGTGACGGCTTTTGCCCGCGCGATCGGCGAT |
| MT16-000061 | TTGATTGCCCAGCTCGGTGGCCCGGGAGGCGTGACGGCTTTTGCCCGCGCGATCGGCGAT |
| MT16-019416 | TTGATTGCCCAGCTCGGTGGCCCGGGAGGCGTGACGGCTTTTGCCCGCGCGATCGGCGAT |
| MT16-027865 | TTGATTGCCCAGCTCGGTGGCCCGGGAGGCGTGACGGCTTTTGCCCGCGCGATCGGCGAT |
| MT16-031693 | TTGATTGCCCAGCTCGGTGGCCCGGGAGGCGTGACGGCTTTTGCCCGCGCGATCGGCGAT |
| MT16-040253 | TTGATTGCCCAGCTCGGTGGCCCGGGAGGCGTGACGGCTTTTGCCCGCGCGATCGGCGAT |
| MT16-045379 | TTGATTGCCCAGCTCGGTGGCCCGGGAGGCGTGACGGCTTTTGCCCGCGCGATCGGCGAT |
| MT16-442728 | TTGATTGCCCAGCTCGGTGGCCCGGGAGGCGTGACGGCTTTTGCCCGCGCGATCGGCGAT |
| MT16-462857 | TTGATTGCCCAGCTCGGTGGCCCGGGAGGCGTGACGGCTTTTGCCCGCGCGATCGGCGAT |
| MT16-480196 | TTGATTGCCCAGCTCGGTGGCCCGGGAGGCGTGACGGCTTTTGCCCGCGCGATCGGCGAT |
| MT16-861555 | TTGATTGCCCAGCTCGGTGGCCCGGGAGGCGTGACGGCTTTTGCCCGCGCGATCGGCGAT |
| MT17-076833 | TTGATTGCCCAGCTCGGTGGCCCGGGAGGCGTGACGGCTTTTGCCCGCGCGATCGGCGAT |
| MT17-110677 | TTGATTGCCCAGCTCGGTGGCCCGGGAGGCGTGACGGCTTTTGCCCGCGCGATCGGCGAT |
| MT17-131730 | TTGATTGCCCAGCTCGGTGGCCCGGGAGGCGTGACGGCTTTTGCCCGCGCGATCGGCGAT |
| MT17-140890 | TTGATTGCCCAGCTCGGTGGCCCGGGAGGCGTGACGGCTTTTGCCCGCGCGATCGGCGAT |
| MT17-141840 | TTGATTGCCCAGCTCGGTGGCCCGGGAGGCGTGACGGCTTTTGCCCGCGCGATCGGCGAT |
| MT17-152488 | TTGATTGCCCAGCTCGGTGGCCCGGGAGGCGTGACGGCTTTTGCCCGCGCGATCGGCGAT |
| MT17-157311 | TTGATTGCCCAGCTCGGTGGCCCGGGAGGCGTGACGGCTTTTGCCCGCGCGATCGGCGAT |
| MT17-161645 | TTGATTGCCCAGCTCGGTGGCCCGGGAGGCGTGACGGCTTTTGCCCGCGCGATCGGCGAT |
| MT17-167951 | TTGATTGCCCAGCTCGGTGGCCCGGGAGGCGTGACGGCTTTTGCCCGCGCGATCGGCGAT |
| MT18-217732 | TTGATTGCCCAGCTCGGTGGCCCGGGAGGCGTGACGGCTTTTGCCCGCGCGATCGGCGAT |
| MT18-252580 | TTGATTGCCCAGCTCGGTGGCCCGGGAGGCGTGACGGCTTTTGCCCGCGCGATCGGCGAT |
| RIVM_H_2016-04 | TTGATTGCCCAGCTCGGTGGCCCGGGAGGCGTGACGGCTTTTGCCCGCGCGATCGGCGAT |
| RIVM_H_2016-07 | TTGATTGCCCAGCTCGGTGGCCCGGGAGGCGTGACGGCTTTTGCCCGCGCGATCGGCGAT |
| RIVM_H_2016-08 | TTGATTGCCCAGCTCGGTGGCCCGGGAGGCGTGACGGCTTTTGCCCGCGCGATCGGCGAT |
| RIVM_H_2016-09 | TTGATTGCCCAGCTCGGTGGCCCGGGAGGCGTGACGGCTTTTGCCCGCGCGATCGGCGAT |
| RIVM_H_2016-11 | TTGATTGCCCAGCTCGGTGGCCCGGGAGGCGTGACGGCTTTTGCCCGCGCGATCGGCGAT |
| RIVM_H_2016-12 | TTGATTGCCCAGCTCGGTGGCCCGGGAGGCGTGACGGCTTTTGCCCGCGCGATCGGCGAT |
| RIVM_H_2016-13 | TTGATTGCCCAGCTCGGTGGCCCGGGAGGCGTGACGGCTTTTGCCCGCGCGATCGGCGAT |
| RIVM_H_2016-14 | TTGATTGCCCAGCTCGGTGGCCCGGGAGGCGTGACGGCTTTTGCCCGCGCGATCGGCGAT |
| RIVM_H_2016-15 | TTGATTGCCCAGCTCGGTGGCCCGGGAGGCGTGACGGCTTTTGCCCGCGCGATCGGCGAT |
| RIVM_H_2017-02 | TTGATTGCCCAGCTCGGTGGCCCGGGAGGCGTGACGGCTTTTGCCCGCGCGATCGGCGAT |
| RIVM_H_2017-03 | TTGATTGCCCAGCTCGGTGGCCCGGGAGGCGTGACGGCTTTTGCCCGCGCGATCGGCGAT |
| RIVM_H_2017-05 | TTGATTGCCCAGCTCGGTGGCCCGGGAGGCGTGACGGCTTTTGCCCGCGCGATCGGCGAT |
| RIVM_H_2017-07 | TTGATTGCCCAGCTCGGTGGCCCGGGAGGCGTGACGGCTTTTGCCCGCGCGATCGGCGAT |
| RIVM_H_2017-08 | TTGATTGCCCAGCTCGGTGGCCCGGGAGGCGTGACGGCTTTTGCCCGCGCGATCGGCGAT |
| RIVM_H_2017-09 | TTGATTGCCCAGCTCGGTGGCCCGGGAGGCGTGACGGCTTTTGCCCGCGCGATCGGCGAT |
| RIVM_H_2017-10 | TTGATTGCCCAGCTCGGTGGCCCGGGAGGCGTGACGGCTTTTGCCCGCGCGATCGGCGAT |
| RIVM_H_2017-11 | TTGATTGCCCAGCTCGGTGGCCCGGGAGGCGTGACGGCTTTTGCCCGCGCGATCGGCGAT |
| RIVM_H_2017-12 | TTGATTGCCCAGCTCGGTGGCCCGGGAGGCGTGACGGCTTTTGCCCGCGCGATCGGCGAT |
| RIVM_H_2017-13 | TTGATTGCCCAGCTCGGTGGCCCGGGAGGCGTGACGGCTTTTGCCCGCGCGATCGGCGAT |
| RIVM_H_2017-14 | TTGATTGCCCAGCTCGGTGGCCCGGGAGGCGTGACGGCTTTTGCCCGCGCGATCGGCGAT |
| RIVM_H_2017-19 | TTGATTGCCCAGCTCGGTGGCCCGGGAGGCGTGACGGCTTTTGCCCGCGCGATCGGCGAT |
| RKI_16-04315 | TTGATTGCCCAGCTCGGTGGCCCGGGAGGCGTGACGGCTTTTGCCCGCGCGATCGGCGAT |
| RKI_17-02304 | TTGATTGCCCAGCTCGGTGGCCCGGGAGGCGTGACGGCTTTTGCCCGCGCGATCGGCGAT |
| RKI_17-02411 | TTGATTGCCCAGCTCGGTGGCCCGGGAGGCGTGACGGCTTTTGCCCGCGCGATCGGCGAT |
| RKI_17-02757 | TTGATTGCCCAGCTCGGTGGCCCGGGAGGCGTGACGGCTTTTGCCCGCGCGATCGGCGAT |
| RKI_17-04797 | TTGATTGCCCAGCTCGGTGGCCCGGGAGGCGTGACGGCTTTTGCCCGCGCGATCGGCGAT |
| S16BD08730 | TTGATTGCCCAGCTCGGTGGCCCGGGAGGCGTGACGGCTTTTGCCCGCGCGATCGGCGAT |
| S18BD03994 | TTGATTGCCCAGCTCGGTGGCCCGGGAGGCGTGACGGCTTTTGCCCGCGCGATCGGCGAT |
| SRR1957844 | TTGATTGCCCAGCTCGGTGGCCCGGGAGGCGTGACGGCTTTTGCCCGCGCGATCGGCGAT |
| SRR1958654 | TTGATTGCCCAGCTCGGTGGCCCGGGAGGCGTGACGGCTTTTGCCCGCGCGATCGGCGAT |
| SRR1965077 | TTGATTGCCCAGCTCGGTGGCCCGGGAGGCGTGACGGCTTTTGCCCGCGCGATCGGCGAT |
| SRR1966369 | TTGATTGCCCAGCTCGGTGGCCCGGGAGGCGTGACGGCTTTTGCCCGCGCGATCGGCGAT |
| SRR1967117 | TTGATTGCCCAGCTCGGTGGCCCGGGAGGCGTGACGGCTTTTGCCCGCGCGATCGGCGAT |
| SRR1967922 | TTGATTGCCCAGCTCGGTGGCCCGGGAGGCGTGACGGCTTTTGCCCGCGCGATCGGCGAT |
| SRR5583183 | TTGATTGCCCAGCTCGGTGGCCCGGGAGGCGTGACGGCTTTTGCCCGCGCGATCGGCGAT |
| SRR5585240 | TTGATTGCCCAGCTCGGTGGCCCGGGAGGCGTGACGGCTTTTGCCCGCGCGATCGGCGAT |
| SRR7216071 | TTGATTGCCCAGCTCGGTGGCCCGGGAGGCGTGACGGCTTTTGCCCGCGCGATCGGCGAT |
| SRR7277793 | TTGATTGCCCAGCTCGGTGGCCCGGGAGGCGTGACGGCTTTTGCCCGCGCGATCGGCGAT |
| SRR7284317 | TTGATTGCCCAGCTCGGTGGCCCGGGAGGCGTGACGGCTTTTGCCCGCGCGATCGGCGAT |
| SRR7299161 | TTGATTGCCCAGCTCGGTGGCCCGGGAGGCGTGACGGCTTTTGCCCGCGCGATCGGCGAT |
| SRR7343877 | TTGATTGCCCAGCTCGGTGGCCCGGGAGGCGTGACGGCTTTTGCCCGCGCGATCGGCGAT |
| SRR7351477 | TTGATTGCCCAGCTCGGTGGCCCGGGAGGCGTGACGGCTTTTGCCCGCGCGATCGGCGAT |
| SRR7401730 | TTGATTGCCCAGCTCGGTGGCCCGGGAGGCGTGACGGCTTTTGCCCGCGCGATCGGCGAT |
| SRR7469092 | TTGATTGCCCAGCTCGGTGGCCCGGGAGGCGTGACGGCTTTTGCCCGCGCGATCGGCGAT |
| SRR7523148 | TTGATTGCCCAGCTCGGTGGCCCGGGAGGCGTGACGGCTTTTGCCCGCGCGATCGGCGAT |
| SRR7523854 | TTGATTGCCCAGCTCGGTGGCCCGGGAGGCGTGACGGCTTTTGCCCGCGCGATCGGCGAT |
| SRR7842487 | TTGATTGCCCAGCTCGGTGGCCCGGGAGGCGTGACGGCTTTTGCCCGCGCGATCGGCGAT |
| SRR7879556 | TTGATTGCCCAGCTCGGTGGCCCGGGAGGCGTGACGGCTTTTGCCCGCGCGATCGGCGAT |
| SRR8054524 | TTGATTGCCCAGCTCGGTGGCCCGGGAGGCGTGACGGCTTTTGCCCGCGCGATCGGCGAT |
| SRR8054525 | TTGATTGCCCAGCTCGGTGGCCCGGGAGGCGTGACGGCTTTTGCCCGCGCGATCGGCGAT |
| SRR8524733 | TTGATTGCCCAGCTCGGTGGCCCGGGAGGCGTGACGGCTTTTGCCCGCGCGATCGGCGAT |
| SRR8526100 | TTGATTGCCCAGCTCGGTGGCCCGGGAGGCGTGACGGCTTTTGCCCGCGCGATCGGCGAT |
| SRR8553991 | TTGATTGCCCAGCTCGGTGGCCCGGGAGGCGTGACGGCTTTTGCCCGCGCGATCGGCGAT |
| blaCTX-M-15_1_AY044436 | CTGATTGCTCACGTTGGCGGCCCGGCTAGCGTCACCGCGTTCGCCCGACAGCTGGGAGAC |
| RIVM_H_2017-15 | CTGATTGCTCACGTTGGCGGCCCGGCTAGCGTCACCGCGTTCGCCCGACAGCTGGGAGAC |
| RKI_17-06869 | CTGATTGCTCACGTTGGCGGCCCGGCTAGCGTCACCGCGTTCGCCCGACAGCTGGGAGAC |
| blaCTX-M-55_1_DQ810789 | CTGATTGCTCACGTTGGCGGCCCGGCTAGCGTCACCGCGTTCGCCCGACAGCTGGGAGAC |
| SRR7349175 | CTGATTGCTCACGTTGGCGGCCCGGCTAGCGTCACCGCGTTCGCCCGACAGCTGGGAGAC |
| ------------------------------------------------------------------------------------ | |
| blaCTX-M-104_1_HQ833652 | GAGACGTTTCGTCTGGATCGCACTGAACCTACGCTGAATACCGCCATTCCCGGCGACCCG |
| S18BD05011 | GAGACGTTTCGTCTGGATCGCACTGAACCTACGCTGAATACCGCCATTCCCGGCGACCCG |
| blaCTX-M-14_1_AF252622 | GAGACGTTTCGTCTGGATCGCACTGAACCTACGCTGAATACCGCCATTCCCGGCGACCCG |
| blaCTX-M-14b_1_DQ359215 | GAGACGTTTCGTCTGGATCGCACTGAACCTACGCTGAATACCGCCATTCCCGGCGACCCG |
| 15EP001483 | GAGACGTTTCGTCTGGATCGCACTGAACCTACGCTGAATACCGCCATTCCCGGCGACCCG |
| 17EP002363 | GAGACGTTTCGTCTGGATCGCACTGAACCTACGCTGAATACCGCCATTCCCGGCGACCCG |
| 313865 | GAGACGTTTCGTCTGGATCGCACTGAACCTACGCTGAATACCGCCATTCCCGGCGACCCG |
| ERR2173656 | GAGACGTTTCGTCTGGATCGCACTGAACCTACGCTGAATACCGCCATTCCCGGCGACCCG |
| ERR2580274 | GAGACGTTTCGTCTGGATCGCACTGAACCTACGCTGAATACCGCCATTCCCGGCGACCCG |
| ERR2580277 | GAGACGTTTCGTCTGGATCGCACTGAACCTACGCTGAATACCGCCATTCCCGGCGACCCG |
| SRR8704720 | GAGACGTTTCGTCTGGATCGCACTGAACCTACGCTGAATACCGCCATTCCCGGCGACCCG |
| MT16-000061 | GAGACGTTTCGTCTGGATCGCACTGAACCTACGCTGAATACCGCCATTCCCGGCGACCCG |
| MT16-019416 | GAGACGTTTCGTCTGGATCGCACTGAACCTACGCTGAATACCGCCATTCCCGGCGACCCG |
| MT16-027865 | GAGACGTTTCGTCTGGATCGCACTGAACCTACGCTGAATACCGCCATTCCCGGCGACCCG |
| MT16-031693 | GAGACGTTTCGTCTGGATCGCACTGAACCTACGCTGAATACCGCCATTCCCGGCGACCCG |
| MT16-040253 | GAGACGTTTCGTCTGGATCGCACTGAACCTACGCTGAATACCGCCATTCCCGGCGACCCG |
| MT16-045379 | GAGACGTTTCGTCTGGATCGCACTGAACCTACGCTGAATACCGCCATTCCCGGCGACCCG |
| MT16-442728 | GAGACGTTTCGTCTGGATCGCACTGAACCTACGCTGAATACCGCCATTCCCGGCGACCCG |
| MT16-462857 | GAGACGTTTCGTCTGGATCGCACTGAACCTACGCTGAATACCGCCATTCCCGGCGACCCG |
| MT16-480196 | GAGACGTTTCGTCTGGATCGCACTGAACCTACGCTGAATACCGCCATTCCCGGCGACCCG |
| MT16-861555 | GAGACGTTTCGTCTGGATCGCACTGAACCTACGCTGAATACCGCCATTCCCGGCGACCCG |
| MT17-076833 | GAGACGTTTCGTCTGGATCGCACTGAACCTACGCTGAATACCGCCATTCCCGGCGACCCG |
| MT17-110677 | GAGACGTTTCGTCTGGATCGCACTGAACCTACGCTGAATACCGCCATTCCCGGCGACCCG |
| MT17-131730 | GAGACGTTTCGTCTGGATCGCACTGAACCTACGCTGAATACCGCCATTCCCGGCGACCCG |
| MT17-140890 | GAGACGTTTCGTCTGGATCGCACTGAACCTACGCTGAATACCGCCATTCCCGGCGACCCG |
| MT17-141840 | GAGACGTTTCGTCTGGATCGCACTGAACCTACGCTGAATACCGCCATTCCCGGCGACCCG |
| MT17-152488 | GAGACGTTTCGTCTGGATCGCACTGAACCTACGCTGAATACCGCCATTCCCGGCGACCCG |
| MT17-157311 | GAGACGTTTCGTCTGGATCGCACTGAACCTACGCTGAATACCGCCATTCCCGGCGACCCG |
| MT17-161645 | GAGACGTTTCGTCTGGATCGCACTGAACCTACGCTGAATACCGCCATTCCCGGCGACCCG |
| MT17-167951 | GAGACGTTTCGTCTGGATCGCACTGAACCTACGCTGAATACCGCCATTCCCGGCGACCCG |
| MT18-217732 | GAGACGTTTCGTCTGGATCGCACTGAACCTACGCTGAATACCGCCATTCCCGGCGACCCG |
| MT18-252580 | GAGACGTTTCGTCTGGATCGCACTGAACCTACGCTGAATACCGCCATTCCCGGCGACCCG |
| RIVM_H_2016-04 | GAGACGTTTCGTCTGGATCGCACTGAACCTACGCTGAATACCGCCATTCCCGGCGACCCG |
| RIVM_H_2016-07 | GAGACGTTTCGTCTGGATCGCACTGAACCTACGCTGAATACCGCCATTCCCGGCGACCCG |
| RIVM_H_2016-08 | GAGACGTTTCGTCTGGATCGCACTGAACCTACGCTGAATACCGCCATTCCCGGCGACCCG |
| RIVM_H_2016-09 | GAGACGTTTCGTCTGGATCGCACTGAACCTACGCTGAATACCGCCATTCCCGGCGACCCG |
| RIVM_H_2016-11 | GAGACGTTTCGTCTGGATCGCACTGAACCTACGCTGAATACCGCCATTCCCGGCGACCCG |
| RIVM_H_2016-12 | GAGACGTTTCGTCTGGATCGCACTGAACCTACGCTGAATACCGCCATTCCCGGCGACCCG |
| RIVM_H_2016-13 | GAGACGTTTCGTCTGGATCGCACTGAACCTACGCTGAATACCGCCATTCCCGGCGACCCG |
| RIVM_H_2016-14 | GAGACGTTTCGTCTGGATCGCACTGAACCTACGCTGAATACCGCCATTCCCGGCGACCCG |
| RIVM_H_2016-15 | GAGACGTTTCGTCTGGATCGCACTGAACCTACGCTGAATACCGCCATTCCCGGCGACCCG |
| RIVM_H_2017-02 | GAGACGTTTCGTCTGGATCGCACTGAACCTACGCTGAATACCGCCATTCCCGGCGACCCG |
| RIVM_H_2017-03 | GAGACGTTTCGTCTGGATCGCACTGAACCTACGCTGAATACCGCCATTCCCGGCGACCCG |
| RIVM_H_2017-05 | GAGACGTTTCGTCTGGATCGCACTGAACCTACGCTGAATACCGCCATTCCCGGCGACCCG |
| RIVM_H_2017-07 | GAGACGTTTCGTCTGGATCGCACTGAACCTACGCTGAATACCGCCATTCCCGGCGACCCG |
| RIVM_H_2017-08 | GAGACGTTTCGTCTGGATCGCACTGAACCTACGCTGAATACCGCCATTCCCGGCGACCCG |
| RIVM_H_2017-09 | GAGACGTTTCGTCTGGATCGCACTGAACCTACGCTGAATACCGCCATTCCCGGCGACCCG |
| RIVM_H_2017-10 | GAGACGTTTCGTCTGGATCGCACTGAACCTACGCTGAATACCGCCATTCCCGGCGACCCG |
| RIVM_H_2017-11 | GAGACGTTTCGTCTGGATCGCACTGAACCTACGCTGAATACCGCCATTCCCGGCGACCCG |
| RIVM_H_2017-12 | GAGACGTTTCGTCTGGATCGCACTGAACCTACGCTGAATACCGCCATTCCCGGCGACCCG |
| RIVM_H_2017-13 | GAGACGTTTCGTCTGGATCGCACTGAACCTACGCTGAATACCGCCATTCCCGGCGACCCG |
| RIVM_H_2017-14 | GAGACGTTTCGTCTGGATCGCACTGAACCTACGCTGAATACCGCCATTCCCGGCGACCCG |
| RIVM_H_2017-19 | GAGACGTTTCGTCTGGATCGCACTGAACCTACGCTGAATACCGCCATTCCCGGCGACCCG |
| RKI_16-04315 | GAGACGTTTCGTCTGGATCGCACTGAACCTACGCTGAATACCGCCATTCCCGGCGACCCG |
| RKI_17-02304 | GAGACGTTTCGTCTGGATCGCACTGAACCTACGCTGAATACCGCCATTCCCGGCGACCCG |
| RKI_17-02411 | GAGACGTTTCGTCTGGATCGCACTGAACCTACGCTGAATACCGCCATTCCCGGCGACCCG |
| RKI_17-02757 | GAGACGTTTCGTCTGGATCGCACTGAACCTACGCTGAATACCGCCATTCCCGGCGACCCG |
| RKI_17-04797 | GAGACGTTTCGTCTGGATCGCACTGAACCTACGCTGAATACCGCCATTCCCGGCGACCCG |
| S16BD08730 | GAGACGTTTCGTCTGGATCGCACTGAACCTACGCTGAATACCGCCATTCCCGGCGACCCG |
| S18BD03994 | GAGACGTTTCGTCTGGATCGCACTGAACCTACGCTGAATACCGCCATTCCCGGCGACCCG |
| SRR1957844 | GAGACGTTTCGTCTGGATCGCACTGAACCTACGCTGAATACCGCCATTCCCGGCGACCCG |
| SRR1958654 | GAGACGTTTCGTCTGGATCGCACTGAACCTACGCTGAATACCGCCATTCCCGGCGACCCG |
| SRR1965077 | GAGACGTTTCGTCTGGATCGCACTGAACCTACGCTGAATACCGCCATTCCCGGCGACCCG |
| SRR1966369 | GAGACGTTTCGTCTGGATCGCACTGAACCTACGCTGAATACCGCCATTCCCGGCGACCCG |
| SRR1967117 | GAGACGTTTCGTCTGGATCGCACTGAACCTACGCTGAATACCGCCATTCCCGGCGACCCG |
| SRR1967922 | GAGACGTTTCGTCTGGATCGCACTGAACCTACGCTGAATACCGCCATTCCCGGCGACCCG |
| SRR5583183 | GAGACGTTTCGTCTGGATCGCACTGAACCTACGCTGAATACCGCCATTCCCGGCGACCCG |
| SRR5585240 | GAGACGTTTCGTCTGGATCGCACTGAACCTACGCTGAATACCGCCATTCCCGGCGACCCG |
| SRR7216071 | GAGACGTTTCGTCTGGATCGCACTGAACCTACGCTGAATACCGCCATTCCCGGCGACCCG |
| SRR7277793 | GAGACGTTTCGTCTGGATCGCACTGAACCTACGCTGAATACCGCCATTCCCGGCGACCCG |
| SRR7284317 | GAGACGTTTCGTCTGGATCGCACTGAACCTACGCTGAATACCGCCATTCCCGGCGACCCG |
| SRR7299161 | GAGACGTTTCGTCTGGATCGCACTGAACCTACGCTGAATACCGCCATTCCCGGCGACCCG |
| SRR7343877 | GAGACGTTTCGTCTGGATCGCACTGAACCTACGCTGAATACCGCCATTCCCGGCGACCCG |
| SRR7351477 | GAGACGTTTCGTCTGGATCGCACTGAACCTACGCTGAATACCGCCATTCCCGGCGACCCG |
| SRR7401730 | GAGACGTTTCGTCTGGATCGCACTGAACCTACGCTGAATACCGCCATTCCCGGCGACCCG |
| SRR7469092 | GAGACGTTTCGTCTGGATCGCACTGAACCTACGCTGAATACCGCCATTCCCGGCGACCCG |
| SRR7523148 | GAGACGTTTCGTCTGGATCGCACTGAACCTACGCTGAATACCGCCATTCCCGGCGACCCG |
| SRR7523854 | GAGACGTTTCGTCTGGATCGCACTGAACCTACGCTGAATACCGCCATTCCCGGCGACCCG |
| SRR7842487 | GAGACGTTTCGTCTGGATCGCACTGAACCTACGCTGAATACCGCCATTCCCGGCGACCCG |
| SRR7879556 | GAGACGTTTCGTCTGGATCGCACTGAACCTACGCTGAATACCGCCATTCCCGGCGACCCG |
| SRR8054524 | GAGACGTTTCGTCTGGATCGCACTGAACCTACGCTGAATACCGCCATTCCCGGCGACCCG |
| SRR8054525 | GAGACGTTTCGTCTGGATCGCACTGAACCTACGCTGAATACCGCCATTCCCGGCGACCCG |
| SRR8524733 | GAGACGTTTCGTCTGGATCGCACTGAACCTACGCTGAATACCGCCATTCCCGGCGACCCG |
| SRR8526100 | GAGACGTTTCGTCTGGATCGCACTGAACCTACGCTGAATACCGCCATTCCCGGCGACCCG |
| SRR8553991 | GAGACGTTTCGTCTGGATCGCACTGAACCTACGCTGAATACCGCCATTCCCGGCGACCCG |
| blaCTX-M-15_1_AY044436 | GAAACGTTCCGTCTCGACCGTACCGAGCCGACGTTAAACACCGCCATTCCGGGCGATCCG |
| RIVM_H_2017-15 | GAAACGTTCCGTCTCGACCGTACCGAGCCGACGTTAAACACCGCCATTCCGGGCGATCCG |
| RKI_17-06869 | GAAACGTTCCGTCTCGACCGTACCGAGCCGACGTTAAACACCGCCATTCCGGGCGATCCG |
| blaCTX-M-55_1_DQ810789 | GAAACGTTCCGTCTCGACCGTACCGAGCCGACGTTAAACACCGCCATTCCGGGCGATCCG |
| SRR7349175 | GAAACGTTCCGTCTCGACCGTACCGAGCCGACGTTAAACACCGCCATTCCGGGCGATCCG |
| ------------------------------------------------------------------------------------ | |
| blaCTX-M-104_1_HQ833652 | AGAGACACCACCACGCCGCGGGCGATGGCGCAGACGTTGCGTCAGCTTACGCTGGGTCAT |
| S18BD05011 | AGAGACACCACCACGCCGCGGGCGATGGCACAGACGTTGCGTCAGCTTACGCTGGGTCAT |
| blaCTX-M-14_1_AF252622 | AGAGACACCACCACGCCGCGGGCGATGGCGCAGACGTTGCGTCAGCTTACGCTGGGTCAT |
| blaCTX-M-14b_1_DQ359215 | AGAGACACCACCACGCCGCGGGCGATGGCACAGACGTTGCGTCAGCTTACGCTGGGTCAT |
| 15EP001483 | AGAGACACCACCACGCCGCGGGCGATGGCACAGACGTTGCGTCAGCTTACGCTGGGTCAT |
| 17EP002363 | AGAGACACCACCACGCCGCGGGCGATGGCACAGACGTTGCGTCAGCTTACGCTGGGTCAT |
| 313865 | AGAGACACCACCACGCCGCGGGCGATGGCACAGACGTTGCGTCAGCTTACGCTGGGTCAT |
| ERR2173656 | AGAGACACCACCACGCCGCGGGCGATGGCACAGACGTTGCGTCAGCTTACGCTGGGTCAT |
| ERR2580274 | AGAGACACCACCACGCCGCGGGCGATGGCACAGACGTTGCGTCAGCTTACGCTGGGTCAT |
| ERR2580277 | AGAGACACCACCACGCCGCGGGCGATGGCACAGACGTTGCGTCAGCTTACGCTGGGTCAT |
| SRR8704720 | AGAGACACCACCACGCCGCGGGCGATGGCACAGACGTTGCGTCAGCTTACGCTGGGTCAT |
| MT16-000061 | AGAGACACCACCACGCCGCGGGCGATGGCACAGACGTTGCGTCAGCTTACGCTGGGTCAT |
| MT16-019416 | AGAGACACCACCACGCCGCGGGCGATGGCACAGACGTTGCGTCAGCTTACGCTGGGTCAT |
| MT16-027865 | AGAGACACCACCACGCCGCGGGCGATGGCACAGACGTTGCGTCAGCTTACGCTGGGTCAT |
| MT16-031693 | AGAGACACCACCACGCCGCGGGCGATGGCACAGACGTTGCGTCAGCTTACGCTGGGTCAT |
| MT16-040253 | AGAGACACCACCACGCCGCGGGCGATGGCACAGACGTTGCGTCAGCTTACGCTGGGTCAT |
| MT16-045379 | AGAGACACCACCACGCCGCGGGCGATGGCACAGACGTTGCGTCAGCTTACGCTGGGTCAT |
| MT16-442728 | AGAGACACCACCACGCCGCGGGCGATGGCACAGACGTTGCGTCAGCTTACGCTGGGTCAT |
| MT16-462857 | AGAGACACCACCACGCCGCGGGCGATGGCACAGACGTTGCGTCAGCTTACGCTGGGTCAT |
| MT16-480196 | AGAGACACCACCACGCCGCGGGCGATGGCACAGACGTTGCGTCAGCTTACGCTGGGTCAT |
| MT16-861555 | AGAGACACCACCACGCCGCGGGCGATGGCACAGACGTTGCGTCAGCTTACGCTGGGTCAT |
| MT17-076833 | AGAGACACCACCACGCCGCGGGCGATGGCACAGACGTTGCGTCAGCTTACGCTGGGTCAT |
| MT17-110677 | AGAGACACCACCACGCCGCGGGCGATGGCACAGACGTTGCGTCAGCTTACGCTGGGTCAT |
| MT17-131730 | AGAGACACCACCACGCCGCGGGCGATGGCACAGACGTTGCGTCAGCTTACGCTGGGTCAT |
| MT17-140890 | AGAGACACCACCACGCCGCGGGCGATGGCACAGACGTTGCGTCAGCTTACGCTGGGTCAT |
| MT17-141840 | AGAGACACCACCACGCCGCGGGCGATGGCACAGACGTTGCGTCAGCTTACGCTGGGTCAT |
| MT17-152488 | AGAGACACCACCACGCCGCGGGCGATGGCACAGACGTTGCGTCAGCTTACGCTGGGTCAT |
| MT17-157311 | AGAGACACCACCACGCCGCGGGCGATGGCACAGACGTTGCGTCAGCTTACGCTGGGTCAT |
| MT17-161645 | AGAGACACCACCACGCCGCGGGCGATGGCACAGACGTTGCGTCAGCTTACGCTGGGTCAT |
| MT17-167951 | AGAGACACCACCACGCCGCGGGCGATGGCACAGACGTTGCGTCAGCTTACGCTGGGTCAT |
| MT18-217732 | AGAGACACCACCACGCCGCGGGCGATGGCACAGACGTTGCGTCAGCTTACGCTGGGTCAT |
| MT18-252580 | AGAGACACCACCACGCCGCGGGCGATGGCACAGACGTTGCGTCAGCTTACGCTGGGTCAT |
| RIVM_H_2016-04 | AGAGACACCACCACGCCGCGGGCGATGGCACAGACGTTGCGTCAGCTTACGCTGGGTCAT |
| RIVM_H_2016-07 | AGAGACACCACCACGCCGCGGGCGATGGCACAGACGTTGCGTCAGCTTACGCTGGGTCAT |
| RIVM_H_2016-08 | AGAGACACCACCACGCCGCGGGCGATGGCACAGACGTTGCGTCAGCTTACGCTGGGTCAT |
| RIVM_H_2016-09 | AGAGACACCACCACGCCGCGGGCGATGGCACAGACGTTGCGTCAGCTTACGCTGGGTCAT |
| RIVM_H_2016-11 | AGAGACACCACCACGCCGCGGGCGATGGCACAGACGTTGCGTCAGCTTACGCTGGGTCAT |
| RIVM_H_2016-12 | AGAGACACCACCACGCCGCGGGCGATGGCACAGACGTTGCGTCAGCTTACGCTGGGTCAT |
| RIVM_H_2016-13 | AGAGACACCACCACGCCGCGGGCGATGGCACAGACGTTGCGTCAGCTTACGCTGGGTCAT |
| RIVM_H_2016-14 | AGAGACACCACCACGCCGCGGGCGATGGCACAGACGTTGCGTCAGCTTACGCTGGGTCAT |
| RIVM_H_2016-15 | AGAGACACCACCACGCCGCGGGCGATGGCACAGACGTTGCGTCAGCTTACGCTGGGTCAT |
| RIVM_H_2017-02 | AGAGACACCACCACGCCGCGGGCGATGGCACAGACGTTGCGTCAGCTTACGCTGGGTCAT |
| RIVM_H_2017-03 | AGAGACACCACCACGCCGCGGGCGATGGCACAGACGTTGCGTCAGCTTACGCTGGGTCAT |
| RIVM_H_2017-05 | AGAGACACCACCACGCCGCGGGCGATGGCACAGACGTTGCGTCAGCTTACGCTGGGTCAT |
| RIVM_H_2017-07 | AGAGACACCACCACGCCGCGGGCGATGGCACAGACGTTGCGTCAGCTTACGCTGGGTCAT |
| RIVM_H_2017-08 | AGAGACACCACCACGCCGCGGGCGATGGCACAGACGTTGCGTCAGCTTACGCTGGGTCAT |
| RIVM_H_2017-09 | AGAGACACCACCACGCCGCGGGCGATGGCACAGACGTTGCGTCAGCTTACGCTGGGTCAT |
| RIVM_H_2017-10 | AGAGACACCACCACGCCGCGGGCGATGGCACAGACGTTGCGTCAGCTTACGCTGGGTCAT |
| RIVM_H_2017-11 | AGAGACACCACCACGCCGCGGGCGATGGCACAGACGTTGCGTCAGCTTACGCTGGGTCAT |
| RIVM_H_2017-12 | AGAGACACCACCACGCCGCGGGCGATGGCACAGACGTTGCGTCAGCTTACGCTGGGTCAT |
| RIVM_H_2017-13 | AGAGACACCACCACGCCGCGGGCGATGGCACAGACGTTGCGTCAGCTTACGCTGGGTCAT |
| RIVM_H_2017-14 | AGAGACACCACCACGCCGCGGGCGATGGCACAGACGTTGCGTCAGCTTACGCTGGGTCAT |
| RIVM_H_2017-19 | AGAGACACCACCACGCCGCGGGCGATGGCACAGACGTTGCGTCAGCTTACGCTGGGTCAT |
| RKI_16-04315 | AGAGACACCACCACGCCGCGGGCGATGGCACAGACGTTGCGTCAGCTTACGCTGGGTCAT |
| RKI_17-02304 | AGAGACACCACCACGCCGCGGGCGATGGCACAGACGTTGCGTCAGCTTACGCTGGGTCAT |
| RKI_17-02411 | AGAGACACCACCACGCCGCGGGCGATGGCACAGACGTTGCGTCAGCTTACGCTGGGTCAT |
| RKI_17-02757 | AGAGACACCACCACGCCGCGGGCGATGGCACAGACGTTGCGTCAGCTTACGCTGGGTCAT |
| RKI_17-04797 | AGAGACACCACCACGCCGCGGGCGATGGCACAGACGTTGCGTCAGCTTACGCTGGGTCAT |
| S16BD08730 | AGAGACACCACCACGCCGCGGGCGATGGCACAGACGTTGCGTCAGCTTACGCTGGGTCAT |
| S18BD03994 | AGAGACACCACCACGCCGCGGGCGATGGCACAGACGTTGCGTCAGCTTACGCTGGGTCAT |
| SRR1957844 | AGAGACACCACCACGCCGCGGGCGATGGCACAGACGTTGCGTCAGCTTACGCTGGGTCAT |
| SRR1958654 | AGAGACACCACCACGCCGCGGGCGATGGCACAGACGTTGCGTCAGCTTACGCTGGGTCAT |
| SRR1965077 | AGAGACACCACCACGCCGCGGGCGATGGCACAGACGTTGCGTCAGCTTACGCTGGGTCAT |
| SRR1966369 | AGAGACACCACCACGCCGCGGGCGATGGCACAGACGTTGCGTCAGCTTACGCTGGGTCAT |
| SRR1967117 | AGAGACACCACCACGCCGCGGGCGATGGCACAGACGTTGCGTCAGCTTACGCTGGGTCAT |
| SRR1967922 | AGAGACACCACCACGCCGCGGGCGATGGCACAGACGTTGCGTCAGCTTACGCTGGGTCAT |
| SRR5583183 | AGAGACACCACCACGCCGCGGGCGATGGCACAGACGTTGCGTCAGCTTACGCTGGGTCAT |
| SRR5585240 | AGAGACACCACCACGCCGCGGGCGATGGCACAGACGTTGCGTCAGCTTACGCTGGGTCAT |
| SRR7216071 | AGAGACACCACCACGCCGCGGGCGATGGCACAGACGTTGCGTCAGCTTACGCTGGGTCAT |
| SRR7277793 | AGAGACACCACCACGCCGCGGGCGATGGCACAGACGTTGCGTCAGCTTACGCTGGGTCAT |
| SRR7284317 | AGAGACACCACCACGCCGCGGGCGATGGCACAGACGTTGCGTCAGCTTACGCTGGGTCAT |
| SRR7299161 | AGAGACACCACCACGCCGCGGGCGATGGCACAGACGTTGCGTCAGCTTACGCTGGGTCAT |
| SRR7343877 | AGAGACACCACCACGCCGCGGGCGATGGCACAGACGTTGCGTCAGCTTACGCTGGGTCAT |
| SRR7351477 | AGAGACACCACCACGCCGCGGGCGATGGCACAGACGTTGCGTCAGCTTACGCTGGGTCAT |
| SRR7401730 | AGAGACACCACCACGCCGCGGGCGATGGCACAGACGTTGCGTCAGCTTACGCTGGGTCAT |
| SRR7469092 | AGAGACACCACCACGCCGCGGGCGATGGCACAGACGTTGCGTCAGCTTACGCTGGGTCAT |
| SRR7523148 | AGAGACACCACCACGCCGCGGGCGATGGCACAGACGTTGCGTCAGCTTACGCTGGGTCAT |
| SRR7523854 | AGAGACACCACCACGCCGCGGGCGATGGCACAGACGTTGCGTCAGCTTACGCTGGGTCAT |
| SRR7842487 | AGAGACACCACCACGCCGCGGGCGATGGCACAGACGTTGCGTCAGCTTACGCTGGGTCAT |
| SRR7879556 | AGAGACACCACCACGCCGCGGGCGATGGCACAGACGTTGCGTCAGCTTACGCTGGGTCAT |
| SRR8054524 | AGAGACACCACCACGCCGCGGGCGATGGCACAGACGTTGCGTCAGCTTACGCTGGGTCAT |
| SRR8054525 | AGAGACACCACCACGCCGCGGGCGATGGCACAGACGTTGCGTCAGCTTACGCTGGGTCAT |
| SRR8524733 | AGAGACACCACCACGCCGCGGGCGATGGCACAGACGTTGCGTCAGCTTACGCTGGGTCAT |
| SRR8526100 | AGAGACACCACCACGCCGCGGGCGATGGCACAGACGTTGCGTCAGCTTACGCTGGGTCAT |
| SRR8553991 | AGAGACACCACCACGCCGCGGGCGATGGCACAGACGTTGCGTCAGCTTACGCTGGGTCAT |
| blaCTX-M-15_1_AY044436 | CGTGATACCACTTCACCTCGGGCAATGGCGCAAACTCTGCGGAATCTGACGCTGGGTAAA |
| RIVM_H_2017-15 | CGTGATACCACTTCACCTCGGGCAATGGCGCAAACTCTGCGGAATCTGACGCTGGGTAAA |
| RKI_17-06869 | CGTGATACCACTTCACCTCGGGCAATGGCGCAAACTCTGCGGAATCTGACGCTGGGTAAA |
| blaCTX-M-55_1_DQ810789 | CGTGATACCACTTCACCTCGGGCAATGGCGCAAACTCTGCGGAATCTGACGCTGGGTAAA |
| SRR7349175 | CGTGATACCACTTCACCTCGGGCAATGGCGCAAACTCTGCGGAATCTGACGCTGGGTAAA |
| ------------------------------------------------------------------------------------ | |
| blaCTX-M-104_1_HQ833652 | GCGCTGGGCGAAACCCAGCGGGCGCAGTTGGTGACGTGGCTCAAAGGCAATACGACCGGC |
| S18BD05011 | GCGCTGGGCGAAACCCAGCGGGCGCAGTTGGTGACGTGGCTCAAAGGCAATACGACCGGC |
| blaCTX-M-14_1_AF252622 | GCGCTGGGCGAAACCCAGCGGGCGCAGTTGGTGACGTGGCTCAAAGGCAATACGACCGGC |
| blaCTX-M-14b_1_DQ359215 | GCGCTGGGCGAAACCCAGCGGGCGCAGTTGGTGACGTGGCTCAAAGGCAATACGACCGGC |
| 15EP001483 | GCGCTGGGCGAAACCCAGCGGGCGCAGTTGGTGACGTGGCTCAAAGGCAATACGACCGGC |
| 17EP002363 | GCGCTGGGCGAAACCCAGCGGGCGCAGTTGGTGACGTGGCTCAAAGGCAATACGACCGGC |
| 313865 | GCGCTGGGCGAAACCCAGCGGGCGCAGTTGGTGACGTGGCTCAAAGGCAATACGACCGGC |
| ERR2173656 | GCGCTGGGCGAAACCCAGCGGGCGCAGTTGGTGACGTGGCTCAAAGGCAATACGACCGGC |
| ERR2580274 | GCGCTGGGCGAAACCCAGCGGGCGCAGTTGGTGACGTGGCTCAAAGGCAATACGACCGGC |
| ERR2580277 | GCGCTGGGCGAAACCCAGCGGGCGCAGTTGGTGACGTGGCTCAAAGGCAATACGACCGGC |
| SRR8704720 | GCGCTGGGCGAAACCCAGCGGGCGCAGTTGGTGACGTGGCTCAAAGGCAATACGACCGGC |
| MT16-000061 | GCGCTGGGCGAAACCCAGCGGGCGCAGTTGGTGACGTGGCTCAAAGGCAATACGACCGGC |
| MT16-019416 | GCGCTGGGCGAAACCCAGCGGGCGCAGTTGGTGACGTGGCTCAAAGGCAATACGACCGGC |
| MT16-027865 | GCGCTGGGCGAAACCCAGCGGGCGCAGTTGGTGACGTGGCTCAAAGGCAATACGACCGGC |
| MT16-031693 | GCGCTGGGCGAAACCCAGCGGGCGCAGTTGGTGACGTGGCTCAAAGGCAATACGACCGGC |
| MT16-040253 | GCGCTGGGCGAAACCCAGCGGGCGCAGTTGGTGACGTGGCTCAAAGGCAATACGACCGGC |
| MT16-045379 | GCGCTGGGCGAAACCCAGCGGGCGCAGTTGGTGACGTGGCTCAAAGGCAATACGACCGGC |
| MT16-442728 | GCGCTGGGCGAAACCCAGCGGGCGCAGTTGGTGACGTGGCTCAAAGGCAATACGACCGGC |
| MT16-462857 | GCGCTGGGCGAAACCCAGCGGGCGCAGTTGGTGACGTGGCTCAAAGGCAATACGACCGGC |
| MT16-480196 | GCGCTGGGCGAAACCCAGCGGGCGCAGTTGGTGACGTGGCTCAAAGGCAATACGACCGGC |
| MT16-861555 | GCGCTGGGCGAAACCCAGCGGGCGCAGTTGGTGACGTGGCTCAAAGGCAATACGACCGGC |
| MT17-076833 | GCGCTGGGCGAAACCCAGCGGGCGCAGTTGGTGACGTGGCTCAAAGGCAATACGACCGGC |
| MT17-110677 | GCGCTGGGCGAAACCCAGCGGGCGCAGTTGGTGACGTGGCTCAAAGGCAATACGACCGGC |
| MT17-131730 | GCGCTGGGCGAAACCCAGCGGGCGCAGTTGGTGACGTGGCTCAAAGGCAATACGACCGGC |
| MT17-140890 | GCGCTGGGCGAAACCCAGCGGGCGCAGTTGGTGACGTGGCTCAAAGGCAATACGACCGGC |
| MT17-141840 | GCGCTGGGCGAAACCCAGCGGGCGCAGTTGGTGACGTGGCTCAAAGGCAATACGACCGGC |
| MT17-152488 | GCGCTGGGCGAAACCCAGCGGGCGCAGTTGGTGACGTGGCTCAAAGGCAATACGACCGGC |
| MT17-157311 | GCGCTGGGCGAAACCCAGCGGGCGCAGTTGGTGACGTGGCTCAAAGGCAATACGACCGGC |
| MT17-161645 | GCGCTGGGCGAAACCCAGCGGGCGCAGTTGGTGACGTGGCTCAAAGGCAATACGACCGGC |
| MT17-167951 | GCGCTGGGCGAAACCCAGCGGGCGCAGTTGGTGACGTGGCTCAAAGGCAATACGACCGGC |
| MT18-217732 | GCGCTGGGCGAAACCCAGCGGGCGCAGTTGGTGACGTGGCTCAAAGGCAATACGACCGGC |
| MT18-252580 | GCGCTGGGCGAAACCCAGCGGGCGCAGTTGGTGACGTGGCTCAAAGGCAATACGACCGGC |
| RIVM_H_2016-04 | GCGCTGGGCGAAACCCAGCGGGCGCAGTTGGTGACGTGGCTCAAAGGCAATACGACCGGC |
| RIVM_H_2016-07 | GCGCTGGGCGAAACCCAGCGGGCGCAGTTGGTGACGTGGCTCAAAGGCAATACGACCGGC |
| RIVM_H_2016-08 | GCGCTGGGCGAAACCCAGCGGGCGCAGTTGGTGACGTGGCTCAAAGGCAATACGACCGGC |
| RIVM_H_2016-09 | GCGCTGGGCGAAACCCAGCGGGCGCAGTTGGTGACGTGGCTCAAAGGCAATACGACCGGC |
| RIVM_H_2016-11 | GCGCTGGGCGAAACCCAGCGGGCGCAGTTGGTGACGTGGCTCAAAGGCAATACGACCGGC |
| RIVM_H_2016-12 | GCGCTGGGCGAAACCCAGCGGGCGCAGTTGGTGACGTGGCTCAAAGGCAATACGACCGGC |
| RIVM_H_2016-13 | GCGCTGGGCGAAACCCAGCGGGCGCAGTTGGTGACGTGGCTCAAAGGCAATACGACCGGC |
| RIVM_H_2016-14 | GCGCTGGGCGAAACCCAGCGGGCGCAGTTGGTGACGTGGCTCAAAGGCAATACGACCGGC |
| RIVM_H_2016-15 | GCGCTGGGCGAAACCCAGCGGGCGCAGTTGGTGACGTGGCTCAAAGGCAATACGACCGGC |
| RIVM_H_2017-02 | GCGCTGGGCGAAACCCAGCGGGCGCAGTTGGTGACGTGGCTCAAAGGCAATACGACCGGC |
| RIVM_H_2017-03 | GCGCTGGGCGAAACCCAGCGGGCGCAGTTGGTGACGTGGCTCAAAGGCAATACGACCGGC |
| RIVM_H_2017-05 | GCGCTGGGCGAAACCCAGCGGGCGCAGTTGGTGACGTGGCTCAAAGGCAATACGACCGGC |
| RIVM_H_2017-07 | GCGCTGGGCGAAACCCAGCGGGCGCAGTTGGTGACGTGGCTCAAAGGCAATACGACCGGC |
| RIVM_H_2017-08 | GCGCTGGGCGAAACCCAGCGGGCGCAGTTGGTGACGTGGCTCAAAGGCAATACGACCGGC |
| RIVM_H_2017-09 | GCGCTGGGCGAAACCCAGCGGGCGCAGTTGGTGACGTGGCTCAAAGGCAATACGACCGGC |
| RIVM_H_2017-10 | GCGCTGGGCGAAACCCAGCGGGCGCAGTTGGTGACGTGGCTCAAAGGCAATACGACCGGC |
| RIVM_H_2017-11 | GCGCTGGGCGAAACCCAGCGGGCGCAGTTGGTGACGTGGCTCAAAGGCAATACGACCGGC |
| RIVM_H_2017-12 | GCGCTGGGCGAAACCCAGCGGGCGCAGTTGGTGACGTGGCTCAAAGGCAATACGACCGGC |
| RIVM_H_2017-13 | GCGCTGGGCGAAACCCAGCGGGCGCAGTTGGTGACGTGGCTCAAAGGCAATACGACCGGC |
| RIVM_H_2017-14 | GCGCTGGGCGAAACCCAGCGGGCGCAGTTGGTGACGTGGCTCAAAGGCAATACGACCGGC |
| RIVM_H_2017-19 | GCGCTGGGCGAAACCCAGCGGGCGCAGTTGGTGACGTGGCTCAAAGGCAATACGACCGGC |
| RKI_16-04315 | GCGCTGGGCGAAACCCAGCGGGCGCAGTTGGTGACGTGGCTCAAAGGCAATACGACCGGC |
| RKI_17-02304 | GCGCTGGGCGAAACCCAGCGGGCGCAGTTGGTGACGTGGCTCAAAGGCAATACGACCGGC |
| RKI_17-02411 | GCGCTGGGCGAAACCCAGCGGGCGCAGTTGGTGACGTGGCTCAAAGGCAATACGACCGGC |
| RKI_17-02757 | GCGCTGGGCGAAACCCAGCGGGCGCAGTTGGTGACGTGGCTCAAAGGCAATACGACCGGC |
| RKI_17-04797 | GCGCTGGGCGAAACCCAGCGGGCGCAGTTGGTGACGTGGCTCAAAGGCAATACGACCGGC |
| S16BD08730 | GCGCTGGGCGAAACCCAGCGGGCGCAGTTGGTGACGTGGCTCAAAGGCAATACGACCGGC |
| S18BD03994 | GCGCTGGGCGAAACCCAGCGGGCGCAGTTGGTGACGTGGCTCAAAGGCAATACGACCGGC |
| SRR1957844 | GCGCTGGGCGAAACCCAGCGGGCGCAGTTGGTGACGTGGCTCAAAGGCAATACGACCGGC |
| SRR1958654 | GCGCTGGGCGAAACCCAGCGGGCGCAGTTGGTGACGTGGCTCAAAGGCAATACGACCGGC |
| SRR1965077 | GCGCTGGGCGAAACCCAGCGGGCGCAGTTGGTGACGTGGCTCAAAGGCAATACGACCGGC |
| SRR1966369 | GCGCTGGGCGAAACCCAGCGGGCGCAGTTGGTGACGTGGCTCAAAGGCAATACGACCGGC |
| SRR1967117 | GCGCTGGGCGAAACCCAGCGGGCGCAGTTGGTGACGTGGCTCAAAGGCAATACGACCGGC |
| SRR1967922 | GCGCTGGGCGAAACCCAGCGGGCGCAGTTGGTGACGTGGCTCAAAGGCAATACGACCGGC |
| SRR5583183 | GCGCTGGGCGAAACCCAGCGGGCGCAGTTGGTGACGTGGCTCAAAGGCAATACGACCGGC |
| SRR5585240 | GCGCTGGGCGAAACCCAGCGGGCGCAGTTGGTGACGTGGCTCAAAGGCAATACGACCGGC |
| SRR7216071 | GCGCTGGGCGAAACCCAGCGGGCGCAGTTGGTGACGTGGCTCAAAGGCAATACGACCGGC |
| SRR7277793 | GCGCTGGGCGAAACCCAGCGGGCGCAGTTGGTGACGTGGCTCAAAGGCAATACGACCGGC |
| SRR7284317 | GCGCTGGGCGAAACCCAGCGGGCGCAGTTGGTGACGTGGCTCAAAGGCAATACGACCGGC |
| SRR7299161 | GCGCTGGGCGAAACCCAGCGGGCGCAGTTGGTGACGTGGCTCAAAGGCAATACGACCGGC |
| SRR7343877 | GCGCTGGGCGAAACCCAGCGGGCGCAGTTGGTGACGTGGCTCAAAGGCAATACGACCGGC |
| SRR7351477 | GCGCTGGGCGAAACCCAGCGGGCGCAGTTGGTGACGTGGCTCAAAGGCAATACGACCGGC |
| SRR7401730 | GCGCTGGGCGAAACCCAGCGGGCGCAGTTGGTGACGTGGCTCAAAGGCAATACGACCGGC |
| SRR7469092 | GCGCTGGGCGAAACCCAGCGGGCGCAGTTGGTGACGTGGCTCAAAGGCAATACGACCGGC |
| SRR7523148 | GCGCTGGGCGAAACCCAGCGGGCGCAGTTGGTGACGTGGCTCAAAGGCAATACGACCGGC |
| SRR7523854 | GCGCTGGGCGAAACCCAGCGGGCGCAGTTGGTGACGTGGCTCAAAGGCAATACGACCGGC |
| SRR7842487 | GCGCTGGGCGAAACCCAGCGGGCGCAGTTGGTGACGTGGCTCAAAGGCAATACGACCGGC |
| SRR7879556 | GCGCTGGGCGAAACCCAGCGGGCGCAGTTGGTGACGTGGCTCAAAGGCAATACGACCGGC |
| SRR8054524 | GCGCTGGGCGAAACCCAGCGGGCGCAGTTGGTGACGTGGCTCAAAGGCAATACGACCGGC |
| SRR8054525 | GCGCTGGGCGAAACCCAGCGGGCGCAGTTGGTGACGTGGCTCAAAGGCAATACGACCGGC |
| SRR8524733 | GCGCTGGGCGAAACCCAGCGGGCGCAGTTGGTGACGTGGCTCAAAGGCAATACGACCGGC |
| SRR8526100 | GCGCTGGGCGAAACCCAGCGGGCGCAGTTGGTGACGTGGCTCAAAGGCAATACGACCGGC |
| SRR8553991 | GCGCTGGGCGAAACCCAGCGGGCGCAGTTGGTGACGTGGCTCAAAGGCAATACGACCGGC |
| blaCTX-M-15_1_AY044436 | GCATTGGGCGACAGCCAACGGGCGCAGCTGGTGACATGGATGAAAGGCAATACCACCGGT |
| RIVM_H_2017-15 | GCATTGGGCGACAGCCAACGGGCGCAGCTGGTGACATGGATGAAAGGCAATACCACCGGT |
| RKI_17-06869 | GCATTGGGCGACAGCCAACGGGCGCAGCTGGTGACATGGATGAAAGGCAATACCACCGGT |
| blaCTX-M-55_1_DQ810789 | GCATTGGGCGACAGCCAACGGGCGCAGCTGGTGACATGGATGAAAGGCAATACCACCGGT |
| SRR7349175 | GCATTGGGCGACAGCCAACGGGCGCAGCTGGTGACATGGATGAAAGGCAATACCACCGGT |
| ------------------------------------------------------------------------------------ | |
| blaCTX-M-104_1_HQ833652 | GCAGCCAGCATTCGGGCCGGCTTACCGACGTCGTGGACTGTGGGTGATAAGACCGGCAGC |
| S18BD05011 | GCAGCCAGCATTCGGGCCGGCTTACCGACGTCGTGGACTGTAGGTGATAAGACCGGCAGC |
| blaCTX-M-14_1_AF252622 | GCAGCCAGCATTCGGGCCGGCTTACCGACGTCGTGGACTGTGGGTGATAAGACCGGCAGC |
| blaCTX-M-14b_1_DQ359215 | GCAGCCAGCATTCGGGCCGGCTTACCGACGTCGTGGACTGTAGGTGATAAGACCGGCAGC |
| 15EP001483 | GCAGCCAGCATTCGGGCCGGCTTACCGACGTCGTGGACTGTAGGTGATAAGACCGGCAGC |
| 17EP002363 | GCAGCCAGCATTCGGGCCGGCTTACCGACGTCGTGGACTGTAGGTGATAAGACCGGCAGC |
| 313865 | GCAGCCAGCATTCGGGCCGGCTTACCGACGTCGTGGACTGTAGGTGATAAGACCGGCAGC |
| ERR2173656 | GCAGCCAGCATTCGGGCCGGCTTACCGACGTCGTGGACTGTAGGTGATAAGACCGGCAGC |
| ERR2580274 | GCAGCCAGCATTCGGGCCGGCTTACCGACGTCGTGGACTGTAGGTGATAAGACCGGCAGC |
| ERR2580277 | GCAGCCAGCATTCGGGCCGGCTTACCGACGTCGTGGACTGTAGGTGATAAGACCGGCAGC |
| SRR8704720 | GCAGCCAGCATTCGGGCCGGCTTACCGACGTCGTGGACTGTAGGTGATAAGACCGGCAGC |
| MT16-000061 | GCAGCCAGCATTCGGGCCGGCTTACCGACGTCGTGGACTGTAGGTGATAAGACCGGCAGC |
| MT16-019416 | GCAGCCAGCATTCGGGCCGGCTTACCGACGTCGTGGACTGTAGGTGATAAGACCGGCAGC |
| MT16-027865 | GCAGCCAGCATTCGGGCCGGCTTACCGACGTCGTGGACTGTAGGTGATAAGACCGGCAGC |
| MT16-031693 | GCAGCCAGCATTCGGGCCGGCTTACCGACGTCGTGGACTGTAGGTGATAAGACCGGCAGC |
| MT16-040253 | GCAGCCAGCATTCGGGCCGGCTTACCGACGTCGTGGACTGTAGGTGATAAGACCGGCAGC |
| MT16-045379 | GCAGCCAGCATTCGGGCCGGCTTACCGACGTCGTGGACTGTAGGTGATAAGACCGGCAGC |
| MT16-442728 | GCAGCCAGCATTCGGGCCGGCTTACCGACGTCGTGGACTGTAGGTGATAAGACCGGCAGC |
| MT16-462857 | GCAGCCAGCATTCGGGCCGGCTTACCGACGTCGTGGACTGTAGGTGATAAGACCGGCAGC |
| MT16-480196 | GCAGCCAGCATTCGGGCCGGCTTACCGACGTCGTGGACTGTAGGTGATAAGACCGGCAGC |
| MT16-861555 | GCAGCCAGCATTCGGGCCGGCTTACCGACGTCGTGGACTGTAGGTGATAAGACCGGCAGC |
| MT17-076833 | GCAGCCAGCATTCGGGCCGGCTTACCGACGTCGTGGACTGTAGGTGATAAGACCGGCAGC |
| MT17-110677 | GCAGCCAGCATTCGGGCCGGCTTACCGACGTCGTGGACTGTAGGTGATAAGACCGGCAGC |
| MT17-131730 | GCAGCCAGCATTCGGGCCGGCTTACCGACGTCGTGGACTGTAGGTGATAAGACCGGCAGC |
| MT17-140890 | GCAGCCAGCATTCGGGCCGGCTTACCGACGTCGTGGACTGTAGGTGATAAGACCGGCAGC |
| MT17-141840 | GCAGCCAGCATTCGGGCCGGCTTACCGACGTCGTGGACTGTAGGTGATAAGACCGGCAGC |
| MT17-152488 | GCAGCCAGCATTCGGGCCGGCTTACCGACGTCGTGGACTGTAGGTGATAAGACCGGCAGC |
| MT17-157311 | GCAGCCAGCATTCGGGCCGGCTTACCGACGTCGTGGACTGTAGGTGATAAGACCGGCAGC |
| MT17-161645 | GCAGCCAGCATTCGGGCCGGCTTACCGACGTCGTGGACTGTAGGTGATAAGACCGGCAGC |
| MT17-167951 | GCAGCCAGCATTCGGGCCGGCTTACCGACGTCGTGGACTGTAGGTGATAAGACCGGCAGC |
| MT18-217732 | GCAGCCAGCATTCGGGCCGGCTTACCGACGTCGTGGACTGTAGGTGATAAGACCGGCAGC |
| MT18-252580 | GCAGCCAGCATTCGGGCCGGCTTACCGACGTCGTGGACTGTAGGTGATAAGACCGGCAGC |
| RIVM_H_2016-04 | GCAGCCAGCATTCGGGCCGGCTTACCGACGTCGTGGACTGTAGGTGATAAGACCGGCAGC |
| RIVM_H_2016-07 | GCAGCCAGCATTCGGGCCGGCTTACCGACGTCGTGGACTGTAGGTGATAAGACCGGCAGC |
| RIVM_H_2016-08 | GCAGCCAGCATTCGGGCCGGCTTACCGACGTCGTGGACTGTAGGTGATAAGACCGGCAGC |
| RIVM_H_2016-09 | GCAGCCAGCATTCGGGCCGGCTTACCGACGTCGTGGACTGTAGGTGATAAGACCGGCAGC |
| RIVM_H_2016-11 | GCAGCCAGCATTCGGGCCGGCTTACCGACGTCGTGGACTGTAGGTGATAAGACCGGCAGC |
| RIVM_H_2016-12 | GCAGCCAGCATTCGGGCCGGCTTACCGACGTCGTGGACTGTAGGTGATAAGACCGGCAGC |
| RIVM_H_2016-13 | GCAGCCAGCATTCGGGCCGGCTTACCGACGTCGTGGACTGTAGGTGATAAGACCGGCAGC |
| RIVM_H_2016-14 | GCAGCCAGCATTCGGGCCGGCTTACCGACGTCGTGGACTGTAGGTGATAAGACCGGCAGC |
| RIVM_H_2016-15 | GCAGCCAGCATTCGGGCCGGCTTACCGACGTCGTGGACTGTAGGTGATAAGACCGGCAGC |
| RIVM_H_2017-02 | GCAGCCAGCATTCGGGCCGGCTTACCGACGTCGTGGACTGTAGGTGATAAGACCGGCAGC |
| RIVM_H_2017-03 | GCAGCCAGCATTCGGGCCGGCTTACCGACGTCGTGGACTGTAGGTGATAAGACCGGCAGC |
| RIVM_H_2017-05 | GCAGCCAGCATTCGGGCCGGCTTACCGACGTCGTGGACTGTAGGTGATAAGACCGGCAGC |
| RIVM_H_2017-07 | GCAGCCAGCATTCGGGCCGGCTTACCGACGTCGTGGACTGTAGGTGATAAGACCGGCAGC |
| RIVM_H_2017-08 | GCAGCCAGCATTCGGGCCGGCTTACCGACGTCGTGGACTGTAGGTGATAAGACCGGCAGC |
| RIVM_H_2017-09 | GCAGCCAGCATTCGGGCCGGCTTACCGACGTCGTGGACTGTAGGTGATAAGACCGGCAGC |
| RIVM_H_2017-10 | GCAGCCAGCATTCGGGCCGGCTTACCGACGTCGTGGACTGTAGGTGATAAGACCGGCAGC |
| RIVM_H_2017-11 | GCAGCCAGCATTCGGGCCGGCTTACCGACGTCGTGGACTGTAGGTGATAAGACCGGCAGC |
| RIVM_H_2017-12 | GCAGCCAGCATTCGGGCCGGCTTACCGACGTCGTGGACTGTAGGTGATAAGACCGGCAGC |
| RIVM_H_2017-13 | GCAGCCAGCATTCGGGCCGGCTTACCGACGTCGTGGACTGTAGGTGATAAGACCGGCAGC |
| RIVM_H_2017-14 | GCAGCCAGCATTCGGGCCGGCTTACCGACGTCGTGGACTGTAGGTGATAAGACCGGCAGC |
| RIVM_H_2017-19 | GCAGCCAGCATTCGGGCCGGCTTACCGACGTCGTGGACTGTAGGTGATAAGACCGGCAGC |
| RKI_16-04315 | GCAGCCAGCATTCGGGCCGGCTTACCGACGTCGTGGACTGTAGGTGATAAGACCGGCAGC |
| RKI_17-02304 | GCAGCCAGCATTCGGGCCGGCTTACCGACGTCGTGGACTGTAGGTGATAAGACCGGCAGC |
| RKI_17-02411 | GCAGCCAGCATTCGGGCCGGCTTACCGACGTCGTGGACTGTAGGTGATAAGACCGGCAGC |
| RKI_17-02757 | GCAGCCAGCATTCGGGCCGGCTTACCGACGTCGTGGACTGTAGGTGATAAGACCGGCAGC |
| RKI_17-04797 | GCAGCCAGCATTCGGGCCGGCTTACCGACGTCGTGGACTGTAGGTGATAAGACCGGCAGC |
| S16BD08730 | GCAGCCAGCATTCGGGCCGGCTTACCGACGTCGTGGACTGTAGGTGATAAGACCGGCAGC |
| S18BD03994 | GCAGCCAGCATTCGGGCCGGCTTACCGACGTCGTGGACTGTAGGTGATAAGACCGGCAGC |
| SRR1957844 | GCAGCCAGCATTCGGGCCGGCTTACCGACGTCGTGGACTGTAGGTGATAAGACCGGCAGC |
| SRR1958654 | GCAGCCAGCATTCGGGCCGGCTTACCGACGTCGTGGACTGTAGGTGATAAGACCGGCAGC |
| SRR1965077 | GCAGCCAGCATTCGGGCCGGCTTACCGACGTCGTGGACTGTAGGTGATAAGACCGGCAGC |
| SRR1966369 | GCAGCCAGCATTCGGGCCGGCTTACCGACGTCGTGGACTGTAGGTGATAAGACCGGCAGC |
| SRR1967117 | GCAGCCAGCATTCGGGCCGGCTTACCGACGTCGTGGACTGTAGGTGATAAGACCGGCAGC |
| SRR1967922 | GCAGCCAGCATTCGGGCCGGCTTACCGACGTCGTGGACTGTAGGTGATAAGACCGGCAGC |
| SRR5583183 | GCAGCCAGCATTCGGGCCGGCTTACCGACGTCGTGGACTGTAGGTGATAAGACCGGCAGC |
| SRR5585240 | GCAGCCAGCATTCGGGCCGGCTTACCGACGTCGTGGACTGTAGGTGATAAGACCGGCAGC |
| SRR7216071 | GCAGCCAGCATTCGGGCCGGCTTACCGACGTCGTGGACTGTAGGTGATAAGACCGGCAGC |
| SRR7277793 | GCAGCCAGCATTCGGGCCGGCTTACCGACGTCGTGGACTGTAGGTGATAAGACCGGCAGC |
| SRR7284317 | GCAGCCAGCATTCGGGCCGGCTTACCGACGTCGTGGACTGTAGGTGATAAGACCGGCAGC |
| SRR7299161 | GCAGCCAGCATTCGGGCCGGCTTACCGACGTCGTGGACTGTAGGTGATAAGACCGGCAGC |
| SRR7343877 | GCAGCCAGCATTCGGGCCGGCTTACCGACGTCGTGGACTGTAGGTGATAAGACCGGCAGC |
| SRR7351477 | GCAGCCAGCATTCGGGCCGGCTTACCGACGTCGTGGACTGTAGGTGATAAGACCGGCAGC |
| SRR7401730 | GCAGCCAGCATTCGGGCCGGCTTACCGACGTCGTGGACTGTAGGTGATAAGACCGGCAGC |
| SRR7469092 | GCAGCCAGCATTCGGGCCGGCTTACCGACGTCGTGGACTGTAGGTGATAAGACCGGCAGC |
| SRR7523148 | GCAGCCAGCATTCGGGCCGGCTTACCGACGTCGTGGACTGTAGGTGATAAGACCGGCAGC |
| SRR7523854 | GCAGCCAGCATTCGGGCCGGCTTACCGACGTCGTGGACTGTAGGTGATAAGACCGGCAGC |
| SRR7842487 | GCAGCCAGCATTCGGGCCGGCTTACCGACGTCGTGGACTGTAGGTGATAAGACCGGCAGC |
| SRR7879556 | GCAGCCAGCATTCGGGCCGGCTTACCGACGTCGTGGACTGTAGGTGATAAGACCGGCAGC |
| SRR8054524 | GCAGCCAGCATTCGGGCCGGCTTACCGACGTCGTGGACTGTAGGTGATAAGACCGGCAGC |
| SRR8054525 | GCAGCCAGCATTCGGGCCGGCTTACCGACGTCGTGGACTGTAGGTGATAAGACCGGCAGC |
| SRR8524733 | GCAGCCAGCATTCGGGCCGGCTTACCGACGTCGTGGACTGTAGGTGATAAGACCGGCAGC |
| SRR8526100 | GCAGCCAGCATTCGGGCCGGCTTACCGACGTCGTGGACTGTAGGTGATAAGACCGGCAGC |
| SRR8553991 | GCAGCCAGCATTCGGGCCGGCTTACCGACGTCGTGGACTGTAGGTGATAAGACCGGCAGC |
| blaCTX-M-15_1_AY044436 | GCAGCGAGCATTCAGGCTGGACTGCCTGCTTCCTGGGTTGTGGGGGATAAAACCGGCAGC |
| RIVM_H_2017-15 | GCAGCGAGCATTCAGGCTGGACTGCCTGCTTCCTGGGTTGTGGGGGATAAAACCGGCAGC |
| RKI_17-06869 | GCAGCGAGCATTCAGGCTGGACTGCCTGCTTCCTGGGTTGTGGGGGATAAAACCGGCAGC |
| blaCTX-M-55_1_DQ810789 | GCAGCGAGCATTCAGGCTGGACTGCCTGCTTCCTGGGTTGTGGGGGATAAAACCGGCAGC |
| SRR7349175 | GCAGCGAGCATTCAGGCTGGACTGCCTGCTTCCTGGGTTGTGGGGGATAAAACCGGCAGC |
| ------------------------------------------------------------------------------------ | |
| blaCTX-M-104_1_HQ833652 | GGCGACTACGGCACCACCAATGATATTGCGGTGATCTGGCCGCAGGGTCGTGCGCCGCTG |
| S18BD05011 | GGCGACTACGGCACCACCAATGATATTGCGGTGATCTGGCCGCAGGGTCGTGCGCCGCTG |
| blaCTX-M-14_1_AF252622 | GGCGACTACGGCACCACCAATGATATTGCGGTGATCTGGCCGCAGGGTCGTGCGCCGCTG |
| blaCTX-M-14b_1_DQ359215 | GGCGACTACGGCACCACCAATGATATTGCGGTGATCTGGCCGCAGGGTCGTGCGCCGCTG |
| 15EP001483 | GGCGACTACGGCACCACCAATGATATTGCGGTGATCTGGCCGCAGGGTCGTGCGCCGCTG |
| 17EP002363 | GGCGACTACGGCACCACCAATGATATTGCGGTGATCTGGCCGCAGGGTCGTGCGCCGCTG |
| 313865 | GGCGACTACGGCACCACCAATGATATTGCGGTGATCTGGCCGCAGGGTCGTGCGCCGCTG |
| ERR2173656 | GGCGACTACGGCACCACCAATGATATTGCGGTGATCTGGCCGCAGGGTCGTGCGCCGCTG |
| ERR2580274 | GGCGACTACGGCACCACCAATGATATTGCGGTGATCTGGCCGCAGGGTCGTGCGCCGCTG |
| ERR2580277 | GGCGACTACGGCACCACCAATGATATTGCGGTGATCTGGCCGCAGGGTCGTGCGCCGCTG |
| SRR8704720 | GGCGACTACGGCACCACCAATGATATTGCGGTGATCTGGCCGCAGGGTCGTGCGCCGCTG |
| MT16-000061 | GGCGACTACGGCACCACCAATGATATTGCGGTGATCTGGCCGCAGGGTCGTGCGCCGCTG |
| MT16-019416 | GGCGACTACGGCACCACCAATGATATTGCGGTGATCTGGCCGCAGGGTCGTGCGCCGCTG |
| MT16-027865 | GGCGACTACGGCACCACCAATGATATTGCGGTGATCTGGCCGCAGGGTCGTGCGCCGCTG |
| MT16-031693 | GGCGACTACGGCACCACCAATGATATTGCGGTGATCTGGCCGCAGGGTCGTGCGCCGCTG |
| MT16-040253 | GGCGACTACGGCACCACCAATGATATTGCGGTGATCTGGCCGCAGGGTCGTGCGCCGCTG |
| MT16-045379 | GGCGACTACGGCACCACCAATGATATTGCGGTGATCTGGCCGCAGGGTCGTGCGCCGCTG |
| MT16-442728 | GGCGACTACGGCACCACCAATGATATTGCGGTGATCTGGCCGCAGGGTCGTGCGCCGCTG |
| MT16-462857 | GGCGACTACGGCACCACCAATGATATTGCGGTGATCTGGCCGCAGGGTCGTGCGCCGCTG |
| MT16-480196 | GGCGACTACGGCACCACCAATGATATTGCGGTGATCTGGCCGCAGGGTCGTGCGCCGCTG |
| MT16-861555 | GGCGACTACGGCACCACCAATGATATTGCGGTGATCTGGCCGCAGGGTCGTGCGCCGCTG |
| MT17-076833 | GGCGACTACGGCACCACCAATGATATTGCGGTGATCTGGCCGCAGGGTCGTGCGCCGCTG |
| MT17-110677 | GGCGACTACGGCACCACCAATGATATTGCGGTGATCTGGCCGCAGGGTCGTGCGCCGCTG |
| MT17-131730 | GGCGACTACGGCACCACCAATGATATTGCGGTGATCTGGCCGCAGGGTCGTGCGCCGCTG |
| MT17-140890 | GGCGACTACGGCACCACCAATGATATTGCGGTGATCTGGCCGCAGGGTCGTGCGCCGCTG |
| MT17-141840 | GGCGACTACGGCACCACCAATGATATTGCGGTGATCTGGCCGCAGGGTCGTGCGCCGCTG |
| MT17-152488 | GGCGACTACGGCACCACCAATGATATTGCGGTGATCTGGCCGCAGGGTCGTGCGCCGCTG |
| MT17-157311 | GGCGACTACGGCACCACCAATGATATTGCGGTGATCTGGCCGCAGGGTCGTGCGCCGCTG |
| MT17-161645 | GGCGACTACGGCACCACCAATGATATTGCGGTGATCTGGCCGCAGGGTCGTGCGCCGCTG |
| MT17-167951 | GGCGACTACGGCACCACCAATGATATTGCGGTGATCTGGCCGCAGGGTCGTGCGCCGCTG |
| MT18-217732 | GGCGACTACGGCACCACCAATGATATTGCGGTGATCTGGCCGCAGGGTCGTGCGCCGCTG |
| MT18-252580 | GGCGACTACGGCACCACCAATGATATTGCGGTGATCTGGCCGCAGGGTCGTGCGCCGCTG |
| RIVM_H_2016-04 | GGCGACTACGGCACCACCAATGATATTGCGGTGATCTGGCCGCAGGGTCGTGCGCCGCTG |
| RIVM_H_2016-07 | GGCGACTACGGCACCACCAATGATATTGCGGTGATCTGGCCGCAGGGTCGTGCGCCGCTG |
| RIVM_H_2016-08 | GGCGACTACGGCACCACCAATGATATTGCGGTGATCTGGCCGCAGGGTCGTGCGCCGCTG |
| RIVM_H_2016-09 | GGCGACTACGGCACCACCAATGATATTGCGGTGATCTGGCCGCAGGGTCGTGCGCCGCTG |
| RIVM_H_2016-11 | GGCGACTACGGCACCACCAATGATATTGCGGTGATCTGGCCGCAGGGTCGTGCGCCGCTG |
| RIVM_H_2016-12 | GGCGACTACGGCACCACCAATGATATTGCGGTGATCTGGCCGCAGGGTCGTGCGCCGCTG |
| RIVM_H_2016-13 | GGCGACTACGGCACCACCAATGATATTGCGGTGATCTGGCCGCAGGGTCGTGCGCCGCTG |
| RIVM_H_2016-14 | GGCGACTACGGCACCACCAATGATATTGCGGTGATCTGGCCGCAGGGTCGTGCGCCGCTG |
| RIVM_H_2016-15 | GGCGACTACGGCACCACCAATGATATTGCGGTGATCTGGCCGCAGGGTCGTGCGCCGCTG |
| RIVM_H_2017-02 | GGCGACTACGGCACCACCAATGATATTGCGGTGATCTGGCCGCAGGGTCGTGCGCCGCTG |
| RIVM_H_2017-03 | GGCGACTACGGCACCACCAATGATATTGCGGTGATCTGGCCGCAGGGTCGTGCGCCGCTG |
| RIVM_H_2017-05 | GGCGACTACGGCACCACCAATGATATTGCGGTGATCTGGCCGCAGGGTCGTGCGCCGCTG |
| RIVM_H_2017-07 | GGCGACTACGGCACCACCAATGATATTGCGGTGATCTGGCCGCAGGGTCGTGCGCCGCTG |
| RIVM_H_2017-08 | GGCGACTACGGCACCACCAATGATATTGCGGTGATCTGGCCGCAGGGTCGTGCGCCGCTG |
| RIVM_H_2017-09 | GGCGACTACGGCACCACCAATGATATTGCGGTGATCTGGCCGCAGGGTCGTGCGCCGCTG |
| RIVM_H_2017-10 | GGCGACTACGGCACCACCAATGATATTGCGGTGATCTGGCCGCAGGGTCGTGCGCCGCTG |
| RIVM_H_2017-11 | GGCGACTACGGCACCACCAATGATATTGCGGTGATCTGGCCGCAGGGTCGTGCGCCGCTG |
| RIVM_H_2017-12 | GGCGACTACGGCACCACCAATGATATTGCGGTGATCTGGCCGCAGGGTCGTGCGCCGCTG |
| RIVM_H_2017-13 | GGCGACTACGGCACCACCAATGATATTGCGGTGATCTGGCCGCAGGGTCGTGCGCCGCTG |
| RIVM_H_2017-14 | GGCGACTACGGCACCACCAATGATATTGCGGTGATCTGGCCGCAGGGTCGTGCGCCGCTG |
| RIVM_H_2017-19 | GGCGACTACGGCACCACCAATGATATTGCGGTGATCTGGCCGCAGGGTCGTGCGCCGCTG |
| RKI_16-04315 | GGCGACTACGGCACCACCAATGATATTGCGGTGATCTGGCCGCAGGGTCGTGCGCCGCTG |
| RKI_17-02304 | GGCGACTACGGCACCACCAATGATATTGCGGTGATCTGGCCGCAGGGTCGTGCGCCGCTG |
| RKI_17-02411 | GGCGACTACGGCACCACCAATGATATTGCGGTGATCTGGCCGCAGGGTCGTGCGCCGCTG |
| RKI_17-02757 | GGCGACTACGGCACCACCAATGATATTGCGGTGATCTGGCCGCAGGGTCGTGCGCCGCTG |
| RKI_17-04797 | GGCGACTACGGCACCACCAATGATATTGCGGTGATCTGGCCGCAGGGTCGTGCGCCGCTG |
| S16BD08730 | GGCGACTACGGCACCACCAATGATATTGCGGTGATCTGGCCGCAGGGTCGTGCGCCGCTG |
| S18BD03994 | GGCGACTACGGCACCACCAATGATATTGCGGTGATCTGGCCGCAGGGTCGTGCGCCGCTG |
| SRR1957844 | GGCGACTACGGCACCACCAATGATATTGCGGTGATCTGGCCGCAGGGTCGTGCGCCGCTG |
| SRR1958654 | GGCGACTACGGCACCACCAATGATATTGCGGTGATCTGGCCGCAGGGTCGTGCGCCGCTG |
| SRR1965077 | GGCGACTACGGCACCACCAATGATATTGCGGTGATCTGGCCGCAGGGTCGTGCGCCGCTG |
| SRR1966369 | GGCGACTACGGCACCACCAATGATATTGCGGTGATCTGGCCGCAGGGTCGTGCGCCGCTG |
| SRR1967117 | GGCGACTACGGCACCACCAATGATATTGCGGTGATCTGGCCGCAGGGTCGTGCGCCGCTG |
| SRR1967922 | GGCGACTACGGCACCACCAATGATATTGCGGTGATCTGGCCGCAGGGTCGTGCGCCGCTG |
| SRR5583183 | GGCGACTACGGCACCACCAATGATATTGCGGTGATCTGGCCGCAGGGTCGTGCGCCGCTG |
| SRR5585240 | GGCGACTACGGCACCACCAATGATATTGCGGTGATCTGGCCGCAGGGTCGTGCGCCGCTG |
| SRR7216071 | GGCGACTACGGCACCACCAATGATATTGCGGTGATCTGGCCGCAGGGTCGTGCGCCGCTG |
| SRR7277793 | GGCGACTACGGCACCACCAATGATATTGCGGTGATCTGGCCGCAGGGTCGTGCGCCGCTG |
| SRR7284317 | GGCGACTACGGCACCACCAATGATATTGCGGTGATCTGGCCGCAGGGTCGTGCGCCGCTG |
| SRR7299161 | GGCGACTACGGCACCACCAATGATATTGCGGTGATCTGGCCGCAGGGTCGTGCGCCGCTG |
| SRR7343877 | GGCGACTACGGCACCACCAATGATATTGCGGTGATCTGGCCGCAGGGTCGTGCGCCGCTG |
| SRR7351477 | GGCGACTACGGCACCACCAATGATATTGCGGTGATCTGGCCGCAGGGTCGTGCGCCGCTG |
| SRR7401730 | GGCGACTACGGCACCACCAATGATATTGCGGTGATCTGGCCGCAGGGTCGTGCGCCGCTG |
| SRR7469092 | GGCGACTACGGCACCACCAATGATATTGCGGTGATCTGGCCGCAGGGTCGTGCGCCGCTG |
| SRR7523148 | GGCGACTACGGCACCACCAATGATATTGCGGTGATCTGGCCGCAGGGTCGTGCGCCGCTG |
| SRR7523854 | GGCGACTACGGCACCACCAATGATATTGCGGTGATCTGGCCGCAGGGTCGTGCGCCGCTG |
| SRR7842487 | GGCGACTACGGCACCACCAATGATATTGCGGTGATCTGGCCGCAGGGTCGTGCGCCGCTG |
| SRR7879556 | GGCGACTACGGCACCACCAATGATATTGCGGTGATCTGGCCGCAGGGTCGTGCGCCGCTG |
| SRR8054524 | GGCGACTACGGCACCACCAATGATATTGCGGTGATCTGGCCGCAGGGTCGTGCGCCGCTG |
| SRR8054525 | GGCGACTACGGCACCACCAATGATATTGCGGTGATCTGGCCGCAGGGTCGTGCGCCGCTG |
| SRR8524733 | GGCGACTACGGCACCACCAATGATATTGCGGTGATCTGGCCGCAGGGTCGTGCGCCGCTG |
| SRR8526100 | GGCGACTACGGCACCACCAATGATATTGCGGTGATCTGGCCGCAGGGTCGTGCGCCGCTG |
| SRR8553991 | GGCGACTACGGCACCACCAATGATATTGCGGTGATCTGGCCGCAGGGTCGTGCGCCGCTG |
| blaCTX-M-15_1_AY044436 | GGTGGCTATGGCACCACCAACGATATCGCGGTGATCTGGCCAAAAGATCGTGCGCCGCTG |
| RIVM_H_2017-15 | GGTGGCTATGGCACCACCAACGATATCGCGGTGATCTGGCCAAAAGATCGTGCGCCGCTG |
| RKI_17-06869 | GGTGGCTATGGCACCACCAACGATATCGCGGTGATCTGGCCAAAAGATCGTGCGCCGCTG |
| blaCTX-M-55_1_DQ810789 | GGTGGCTATGGCACCACCAACGATATCGCGGTGATCTGGCCAAAAGATCGTGCGCCGCTG |
| SRR7349175 | GGTGGCTATGGCACCACCAACGATATCGCGGTGATCTGGCCAAAAGATCGTGCGCCGCTG |
| ------------------------------------------------------------------------------------ | |
| blaCTX-M-104_1_HQ833652 | GTTCTGGTGACCTATTTTACCCAGCCGCAACAGAACGCAGAGAACCGCCGCGATGTGCTG |
| S18BD05011 | GTTCTGGTGACCTATTTTACCCAGCCGCAACAGAACGCAGAGAACCGCCGCGATGTGCTG |
| blaCTX-M-14_1_AF252622 | GTTCTGGTGACCTATTTTACCCAGCCGCAACAGAACGCAGAGAGCCGCCGCGATGTGCTG |
| blaCTX-M-14b_1_DQ359215 | GTTCTGGTGACCTATTTTACCCAGCCGCAACAGAACGCAGAGAGCCGCCGCGATGTGCTG |
| 15EP001483 | GTTCTGGTGACCTATTTTACCCAGCCGCAACAGAACGCAGAGAGCCGCCGCGATGTGCTG |
| 17EP002363 | GTTCTGGTGACCTATTTTACCCAGCCGCAACAGAACGCAGAGAGCCGCCGCGATGTGCTG |
| 313865 | GTTCTGGTGACCTATTTTACCCAGCCGCAACAGAACGCAGAGAGCCGCCGCGATGTGCTG |
| ERR2173656 | GTTCTGGTGACCTATTTTACCCAGCCGCAACAGAACGCAGAGAGCCGCCGCGATGTGCTG |
| ERR2580274 | GTTCTGGTGACCTATTTTACCCAGCCGCAACAGAACGCAGAGAGCCGCCGCGATGTGCTG |
| ERR2580277 | GTTCTGGTGACCTATTTTACCCAGCCGCAACAGAACGCAGAGAGCCGCCGCGATGTGCTG |
| SRR8704720 | GTTCTGGTGACCTATTTTACCCAGCCGCAACAGAACGCAGAGAGCCGCCGCGATGTGCTG |
| MT16-000061 | GTTCTGGTGACCTATTTTACCCAGCCGCAACAGAACGCAGAGAGCCGCCGCGATGTGCTG |
| MT16-019416 | GTTCTGGTGACCTATTTTACCCAGCCGCAACAGAACGCAGAGAGCCGCCGCGATGTGCTG |
| MT16-027865 | GTTCTGGTGACCTATTTTACCCAGCCGCAACAGAACGCAGAGAGCCGCCGCGATGTGCTG |
| MT16-031693 | GTTCTGGTGACCTATTTTACCCAGCCGCAACAGAACGCAGAGAGCCGCCGCGATGTGCTG |
| MT16-040253 | GTTCTGGTGACCTATTTTACCCAGCCGCAACAGAACGCAGAGAGCCGCCGCGATGTGCTG |
| MT16-045379 | GTTCTGGTGACCTATTTTACCCAGCCGCAACAGAACGCAGAGAGCCGCCGCGATGTGCTG |
| MT16-442728 | GTTCTGGTGACCTATTTTACCCAGCCGCAACAGAACGCAGAGAGCCGCCGCGATGTGCTG |
| MT16-462857 | GTTCTGGTGACCTATTTTACCCAGCCGCAACAGAACGCAGAGAGCCGCCGCGATGTGCTG |
| MT16-480196 | GTTCTGGTGACCTATTTTACCCAGCCGCAACAGAACGCAGAGAGCCGCCGCGATGTGCTG |
| MT16-861555 | GTTCTGGTGACCTATTTTACCCAGCCGCAACAGAACGCAGAGAGCCGCCGCGATGTGCTG |
| MT17-076833 | GTTCTGGTGACCTATTTTACCCAGCCGCAACAGAACGCAGAGAGCCGCCGCGATGTGCTG |
| MT17-110677 | GTTCTGGTGACCTATTTTACCCAGCCGCAACAGAACGCAGAGAGCCGCCGCGATGTGCTG |
| MT17-131730 | GTTCTGGTGACCTATTTTACCCAGCCGCAACAGAACGCAGAGAGCCGCCGCGATGTGCTG |
| MT17-140890 | GTTCTGGTGACCTATTTTACCCAGCCGCAACAGAACGCAGAGAGCCGCCGCGATGTGCTG |
| MT17-141840 | GTTCTGGTGACCTATTTTACCCAGCCGCAACAGAACGCAGAGAGCCGCCGCGATGTGCTG |
| MT17-152488 | GTTCTGGTGACCTATTTTACCCAGCCGCAACAGAACGCAGAGAGCCGCCGCGATGTGCTG |
| MT17-157311 | GTTCTGGTGACCTATTTTACCCAGCCGCAACAGAACGCAGAGAGCCGCCGCGATGTGCTG |
| MT17-161645 | GTTCTGGTGACCTATTTTACCCAGCCGCAACAGAACGCAGAGAGCCGCCGCGATGTGCTG |
| MT17-167951 | GTTCTGGTGACCTATTTTACCCAGCCGCAACAGAACGCAGAGAGCCGCCGCGATGTGCTG |
| MT18-217732 | GTTCTGGTGACCTATTTTACCCAGCCGCAACAGAACGCAGAGAGCCGCCGCGATGTGCTG |
| MT18-252580 | GTTCTGGTGACCTATTTTACCCAGCCGCAACAGAACGCAGAGAGCCGCCGCGATGTGCTG |
| RIVM_H_2016-04 | GTTCTGGTGACCTATTTTACCCAGCCGCAACAGAACGCAGAGAGCCGCCGCGATGTGCTG |
| RIVM_H_2016-07 | GTTCTGGTGACCTATTTTACCCAGCCGCAACAGAACGCAGAGAGCCGCCGCGATGTGCTG |
| RIVM_H_2016-08 | GTTCTGGTGACCTATTTTACCCAGCCGCAACAGAACGCAGAGAGCCGCCGCGATGTGCTG |
| RIVM_H_2016-09 | GTTCTGGTGACCTATTTTACCCAGCCGCAACAGAACGCAGAGAGCCGCCGCGATGTGCTG |
| RIVM_H_2016-11 | GTTCTGGTGACCTATTTTACCCAGCCGCAACAGAACGCAGAGAGCCGCCGCGATGTGCTG |
| RIVM_H_2016-12 | GTTCTGGTGACCTATTTTACCCAGCCGCAACAGAACGCAGAGAGCCGCCGCGATGTGCTG |
| RIVM_H_2016-13 | GTTCTGGTGACCTATTTTACCCAGCCGCAACAGAACGCAGAGAGCCGCCGCGATGTGCTG |
| RIVM_H_2016-14 | GTTCTGGTGACCTATTTTACCCAGCCGCAACAGAACGCAGAGAGCCGCCGCGATGTGCTG |
| RIVM_H_2016-15 | GTTCTGGTGACCTATTTTACCCAGCCGCAACAGAACGCAGAGAGCCGCCGCGATGTGCTG |
| RIVM_H_2017-02 | GTTCTGGTGACCTATTTTACCCAGCCGCAACAGAACGCAGAGAGCCGCCGCGATGTGCTG |
| RIVM_H_2017-03 | GTTCTGGTGACCTATTTTACCCAGCCGCAACAGAACGCAGAGAGCCGCCGCGATGTGCTG |
| RIVM_H_2017-05 | GTTCTGGTGACCTATTTTACCCAGCCGCAACAGAACGCAGAGAGCCGCCGCGATGTGCTG |
| RIVM_H_2017-07 | GTTCTGGTGACCTATTTTACCCAGCCGCAACAGAACGCAGAGAGCCGCCGCGATGTGCTG |
| RIVM_H_2017-08 | GTTCTGGTGACCTATTTTACCCAGCCGCAACAGAACGCAGAGAGCCGCCGCGATGTGCTG |
| RIVM_H_2017-09 | GTTCTGGTGACCTATTTTACCCAGCCGCAACAGAACGCAGAGAGCCGCCGCGATGTGCTG |
| RIVM_H_2017-10 | GTTCTGGTGACCTATTTTACCCAGCCGCAACAGAACGCAGAGAGCCGCCGCGATGTGCTG |
| RIVM_H_2017-11 | GTTCTGGTGACCTATTTTACCCAGCCGCAACAGAACGCAGAGAGCCGCCGCGATGTGCTG |
| RIVM_H_2017-12 | GTTCTGGTGACCTATTTTACCCAGCCGCAACAGAACGCAGAGAGCCGCCGCGATGTGCTG |
| RIVM_H_2017-13 | GTTCTGGTGACCTATTTTACCCAGCCGCAACAGAACGCAGAGAGCCGCCGCGATGTGCTG |
| RIVM_H_2017-14 | GTTCTGGTGACCTATTTTACCCAGCCGCAACAGAACGCAGAGAGCCGCCGCGATGTGCTG |
| RIVM_H_2017-19 | GTTCTGGTGACCTATTTTACCCAGCCGCAACAGAACGCAGAGAGCCGCCGCGATGTGCTG |
| RKI_16-04315 | GTTCTGGTGACCTATTTTACCCAGCCGCAACAGAACGCAGAGAGCCGCCGCGATGTGCTG |
| RKI_17-02304 | GTTCTGGTGACCTATTTTACCCAGCCGCAACAGAACGCAGAGAGCCGCCGCGATGTGCTG |
| RKI_17-02411 | GTTCTGGTGACCTATTTTACCCAGCCGCAACAGAACGCAGAGAGCCGCCGCGATGTGCTG |
| RKI_17-02757 | GTTCTGGTGACCTATTTTACCCAGCCGCAACAGAACGCAGAGAGCCGCCGCGATGTGCTG |
| RKI_17-04797 | GTTCTGGTGACCTATTTTACCCAGCCGCAACAGAACGCAGAGAGCCGCCGCGATGTGCTG |
| S16BD08730 | GTTCTGGTGACCTATTTTACCCAGCCGCAACAGAACGCAGAGAGCCGCCGCGATGTGCTG |
| S18BD03994 | GTTCTGGTGACCTATTTTACCCAGCCGCAACAGAACGCAGAGAGCCGCCGCGATGTGCTG |
| SRR1957844 | GTTCTGGTGACCTATTTTACCCAGCCGCAACAGAACGCAGAGAGCCGCCGCGATGTGCTG |
| SRR1958654 | GTTCTGGTGACCTATTTTACCCAGCCGCAACAGAACGCAGAGAGCCGCCGCGATGTGCTG |
| SRR1965077 | GTTCTGGTGACCTATTTTACCCAGCCGCAACAGAACGCAGAGAGCCGCCGCGATGTGCTG |
| SRR1966369 | GTTCTGGTGACCTATTTTACCCAGCCGCAACAGAACGCAGAGAGCCGCCGCGATGTGCTG |
| SRR1967117 | GTTCTGGTGACCTATTTTACCCAGCCGCAACAGAACGCAGAGAGCCGCCGCGATGTGCTG |
| SRR1967922 | GTTCTGGTGACCTATTTTACCCAGCCGCAACAGAACGCAGAGAGCCGCCGCGATGTGCTG |
| SRR5583183 | GTTCTGGTGACCTATTTTACCCAGCCGCAACAGAACGCAGAGAGCCGCCGCGATGTGCTG |
| SRR5585240 | GTTCTGGTGACCTATTTTACCCAGCCGCAACAGAACGCAGAGAGCCGCCGCGATGTGCTG |
| SRR7216071 | GTTCTGGTGACCTATTTTACCCAGCCGCAACAGAACGCAGAGAGCCGCCGCGATGTGCTG |
| SRR7277793 | GTTCTGGTGACCTATTTTACCCAGCCGCAACAGAACGCAGAGAGCCGCCGCGATGTGCTG |
| SRR7284317 | GTTCTGGTGACCTATTTTACCCAGCCGCAACAGAACGCAGAGAGCCGCCGCGATGTGCTG |
| SRR7299161 | GTTCTGGTGACCTATTTTACCCAGCCGCAACAGAACGCAGAGAGCCGCCGCGATGTGCTG |
| SRR7343877 | GTTCTGGTGACCTATTTTACCCAGCCGCAACAGAACGCAGAGAACCGCCGCGATGTGCTG |
| SRR7351477 | GTTCTGGTGACCTATTTTACCCAGCCGCAACAGAACGCAGAGAGCCGCCGCGATGTGCTG |
| SRR7401730 | GTTCTGGTGACCTATTTTACCCAGCCGCAACAGAACGCAGAGAGCCGCCGCGATGTGCTG |
| SRR7469092 | GTTCTGGTGACCTATTTTACCCAGCCGCAACAGAACGCAGAGAGCCGCCGCGATGTGCTG |
| SRR7523148 | GTTCTGGTGACCTATTTTACCCAGCCGCAACAGAACGCAGAGAACCGCCGCGATGTGCTG |
| SRR7523854 | GTTCTGGTGACCTATTTTACCCAGCCGCAACAGAACGCAGAGAGCCGCCGCGATGTGCTG |
| SRR7842487 | GTTCTGGTGACCTATTTTACCCAGCCGCAACAGAACGCAGAGAGCCGCCGCGATGTGCTG |
| SRR7879556 | GTTCTGGTGACCTATTTTACCCAGCCGCAACAGAACGCAGAGAGCCGCCGCGATGTGCTG |
| SRR8054524 | GTTCTGGTGACCTATTTTACCCAGCCGCAACAGAACGCAGAGAGCCGCCGCGATGTGCTG |
| SRR8054525 | GTTCTGGTGACCTATTTTACCCAGCCGCAACAGAACGCAGAGAGCCGCCGCGATGTGCTG |
| SRR8524733 | GTTCTGGTGACCTATTTTACCCAGCCGCAACAGAACGCAGAGAGCCGCCGCGATGTGCTG |
| SRR8526100 | GTTCTGGTGACCTATTTTACCCAGCCGCAACAGAACGCAGAGAGCCGCCGCGATGTGCTG |
| SRR8553991 | GTTCTGGTGACCTATTTTACCCAGCCGCAACAGAACGCAGAGAGCCGCCGCGATGTGCTG |
| blaCTX-M-15_1_AY044436 | ATTCTGGTCACTTACTTCACCCAGCCTCAACCTAAGGCAGAAAGCCGTCGCGATGTATTA |
| RIVM_H_2017-15 | ATTCTGGTCACTTACTTCACCCAGCCTCAACCTAAGGCAGAAAGCCGTCGCGATGTATTA |
| RKI_17-06869 | ATTCTGGTCACTTACTTCACCCAGCCTCAACCTAAGGCAGAAAGCCGTCGCGATGTATTA |
| blaCTX-M-55_1_DQ810789 | ATTCTGGTCACTTACTTCACCCAGCCTCAACCTAAGGCAGAAAGCCGTCGCGATGTATTA |
| SRR7349175 | ATTCTGGTCACTTACTTCACCCAGCCTCAACCTAAGGCAGAAAGCCGTCGCGATGTATTA |
| ------------------------------------------------------------------------------------ | |
| blaCTX-M-104_1_HQ833652 | GCTTCAGCGGCGAGAATCATCGCCGAAGGGCTGTAA |
| S18BD05011 | GCTTCAGCGGCGAGAATCATCGCCGAAGGGCTGTAA |
| blaCTX-M-14_1_AF252622 | GCTTCAGCGGCGAGAATCATCGCCGAAGGGCTGTAA |
| blaCTX-M-14b_1_DQ359215 | GCTTCAGCGGCGAGAATCATCGCCGAAGGGCTGTAA |
| 15EP001483 | GCTTCAGCGGCGAGAATCATCGCCGAAGGGCTGTAA |
| 17EP002363 | GCTTCAGCGGCGAGAATCATCGCCGAAGGGCTGTAA |
| 313865 | GCTTCAGCGGCGAGAATCATCGCCGAAGGGCTGTAA |
| ERR2173656 | GCTTCAGCGGCGAGAATCATCGCCGAAGGGCTGTAA |
| ERR2580274 | GCTTCAGCGGCGAGAATCATCGCCGAAGGGCTGTAA |
| ERR2580277 | GCTTCAGCGGCGAGAATCATCGCCGAAGGGCTGTAA |
| SRR8704720 | GCTTCAGCGGCGAGAATCATCGCCGAAGGGCTGTAA |
| MT16-000061 | GCTTCAGCGGCGAGAATCATCGCCGAAGGGCTGTAA |
| MT16-019416 | GCTTCAGCGGCGAGAATCATCGCCGAAGGGCTGTAA |
| MT16-027865 | GCTTCAGCGGCGAGAATCATCGCCGAAGGGCTGTAA |
| MT16-031693 | GCTTCAGCGGCGAGAATCATCGCCGAAGGGCTGTAA |
| MT16-040253 | GCTTCAGCGGCGAGAATCATCGCCGAAGGGCTGTAA |
| MT16-045379 | GCTTCAGCGGCGAGAATCATCGCCGAAGGGCTGTAA |
| MT16-442728 | GCTTCAGCGGCGAGAATCATCGCCGAAGGGCTGTAA |
| MT16-462857 | GCTTCAGCGGCGAGAATCATCGCCGAAGGGCTGTAA |
| MT16-480196 | GCTTCAGCGGCGAGAATCATCGCCGAAGGGCTGTAA |
| MT16-861555 | GCTTCAGCGGCGAGAATCATCGCCGAAGGGCTGTAA |
| MT17-076833 | GCTTCAGCGGCGAGAATCATCGCCGAAGGGCTGTAA |
| MT17-110677 | GCTTCAGCGGCGAGAATCATCGCCGAAGGGCTGTAA |
| MT17-131730 | GCTTCAGCGGCGAGAATCATCGCCGAAGGGCTGTAA |
| MT17-140890 | GCTTCAGCGGCGAGAATCATCGCCGAAGGGCTGTAA |
| MT17-141840 | GCTTCAGCGGCGAGAATCATCGCCGAAGGGCTGTAA |
| MT17-152488 | GCTTCAGCGGCGAGAATCATCGCCGAAGGGCTGTAA |
| MT17-157311 | GCTTCAGCGGCGAGAATCATCGCCGAAGGGCTGTAA |
| MT17-161645 | GCTTCAGCGGCGAGAATCATCGCCGAAGGGCTGTAA |
| MT17-167951 | GCTTCAGCGGCGAGAATCATCGCCGAAGGGCTGTAA |
| MT18-217732 | GCTTCAGCGGCGAGAATCATCGCCGAAGGGCTGTAA |
| MT18-252580 | GCTTCAGCGGCGAGAATCATCGCCGAAGGGCTGTAA |
| RIVM_H_2016-04 | GCTTCAGCGGCGAGAATCATCGCCGAAGGGCTGTAA |
| RIVM_H_2016-07 | GCTTCAGCGGCGAGAATCATCGCCGAAGGGCTGTAA |
| RIVM_H_2016-08 | GCTTCAGCGGCGAGAATCATCGCCGAAGGGCTGTAA |
| RIVM_H_2016-09 | GCTTCAGCGGCGAGAATCATCGCCGAAGGGCTGTAA |
| RIVM_H_2016-11 | GCTTCAGCGGCGAGAATCATCGCCGAAGGGCTGTAA |
| RIVM_H_2016-12 | GCTTCAGCGGCGAGAATCATCGCCGAAGGGCTGTAA |
| RIVM_H_2016-13 | GCTTCAGCGGCGAGAATCATCGCCGAAGGGCTGTAA |
| RIVM_H_2016-14 | GCTTCAGCGGCGAGAATCATCGCCGAAGGGCTGTAA |
| RIVM_H_2016-15 | GCTTCAGCGGCGAGAATCATCGCCGAAGGGCTGTAA |
| RIVM_H_2017-02 | GCTTCAGCGGCGAGAATCATCGCCGAAGGGCTGTAA |
| RIVM_H_2017-03 | GCTTCAGCGGCGAGAATCATCGCCGAAGGGCTGTAA |
| RIVM_H_2017-05 | GCTTCAGCGGCGAGAATCATCGCCGAAGGGCTGTAA |
| RIVM_H_2017-07 | GCTTCAGCGGCGAGAATCATCGCCGAAGGGCTGTAA |
| RIVM_H_2017-08 | GCTTCAGCGGCGAGAATCATCGCCGAAGGGCTGTAA |
| RIVM_H_2017-09 | GCTTCAGCGGCGAGAATCATCGCCGAAGGGCTGTAA |
| RIVM_H_2017-10 | GCTTCAGCGGCGAGAATCATCGCCGAAGGGCTGTAA |
| RIVM_H_2017-11 | GCTTCAGCGGCGAGAATCATCGCCGAAGGGCTGTAA |
| RIVM_H_2017-12 | GCTTCAGCGGCGAGAATCATCGCCGAAGGGCTGTAA |
| RIVM_H_2017-13 | GCTTCAGCGGCGAGAATCATCGCCGAAGGGCTGTAA |
| RIVM_H_2017-14 | GCTTCAGCGGCGAGAATCATCGCCGAAGGGCTGTAA |
| RIVM_H_2017-19 | GCTTCAGCGGCGAGAATCATCGCCGAAGGGCTGTAA |
| RKI_16-04315 | GCTTCAGCGGCGAGAATCATCGCCGAAGGGCTGTAA |
| RKI_17-02304 | GCTTCAGCGGCGAGAATCATCGCCGAAGGGCTGTAA |
| RKI_17-02411 | GCTTCAGCGGCGAGAATCATCGCCGAAGGGCTGTAA |
| RKI_17-02757 | GCTTCAGCGGCGAGAATCATCGCCGAAGGGCTGTAA |
| RKI_17-04797 | GCTTCAGCGGCGAGAATCATCGCCGAAGGGCTGTAA |
| S16BD08730 | GCTTCAGCGGCGAGAATCATCGCCGAAGGGCTGTAA |
| S18BD03994 | GCTTCAGCGGCGAGAATCATCGCCGAAGGGCTGTAA |
| SRR1957844 | GCTTCAGCGGCGAGAATCATCGCCGAAGGGCTGTAA |
| SRR1958654 | GCTTCAGCGGCGAGAATCATCGCCGAAGGGCTGTAA |
| SRR1965077 | GCTTCAGCGGCGAGAATCATCGCCGAAGGGCTGTAA |
| SRR1966369 | GCTTCAGCGGCGAGAATCATCGCCGAAGGGCTGTAA |
| SRR1967117 | GCTTCAGCGGCGAGAATCATCGCCGAAGGGCTGTAA |
| SRR1967922 | GCTTCAGCGGCGAGAATCATCGCCGAAGGGCTGTAA |
| SRR5583183 | GCTTCAGCGGCGAGAATCATCGCCGAAGGGCTGTAA |
| SRR5585240 | GCTTCAGCGGCGAGAATCATCGCCGAAGGGCTGTAA |
| SRR7216071 | GCTTCAGCGGCGAGAATCATCGCCGAAGGGCTGTAA |
| SRR7277793 | GCTTCAGCGGCGAGAATCATCGCCGAAGGGCTGTAA |
| SRR7284317 | GCTTCAGCGGCGAGAATCATCGCCGAAGGGCTGTAA |
| SRR7299161 | GCTTCAGCGGCGAGAATCATCGCCGAAGGGCTGTAA |
| SRR7343877 | GCTTCAGCGGCGAGAATCATCGCCGAAGGGCTGTAA |
| SRR7351477 | GCTTCAGCGGCGAGAATCATCGCCGAAGGGCTGTAA |
| SRR7401730 | GCTTCAGCGGCGAGAATCATCGCCGAAGGGCTGTAA |
| SRR7469092 | GCTTCAGCGGCGAGAATCATCGCCGAAGGGCTGTAA |
| SRR7523148 | GCTTCAGCGGCGAGAATCATCGCCGAAGGGCTGTAA |
| SRR7523854 | GCTTCAGCGGCGAGAATCATCGCCGAAGGGCTGTAA |
| SRR7842487 | GCTTCAGCGGCGAGAATCATCGCCGAAGGGCTGTAA |
| SRR7879556 | GCTTCAGCGGCGAGAATCATCGCCGAAGGGCTGTAA |
| SRR8054524 | GCTTCAGCGGCGAGAATCATCGCCGAAGGGCTGTAA |
| SRR8054525 | GCTTCAGCGGCGAGAATCATCGCCGAAGGGCTGTAA |
| SRR8524733 | GCTTCAGCGGCGAGAATCATCGCCGAAGGGCTGTAA |
| SRR8526100 | GCTTCAGCGGCGAGAATCATCGCCGAAGGGCTGTAA |
| SRR8553991 | GCTTCAGCGGCGAGAATCATCGCCGAAGGGCTGTAA |
| blaCTX-M-15_1_AY044436 | GCGTCGGCGGCTAAAATCGTCACCGACGGTTTGTAA |
| RIVM_H_2017-15 | GCGTCGGCGGCTAAAATCGTCACCGACGGTTTGTAA |
| RKI_17-06869 | GCGTCGGCGGCTAAAATCGTCACCGACGGTTTGTAA |
| blaCTX-M-55_1_DQ810789 | GCGTCGGCGGCTAAAATCGTCACCGACGGTTTGTAA |
| SRR7349175 | GCGTCGGCGGCTAAAATCGTCACCGACGGTTTGTAA |
