# Supplementary material for: Genomic epidemiology of emerging ESBL-producing Salmonella Kentucky blaCTX-M-14b in Europe
Source: Emerg Microbes Infect. 2020 Sep 30;9(1):2124–35. doi: 10.1080/22221751.2020.1821582 (PMC7580578; doi:10.1080/22221751.2020.1821582)
Supplement: Suppelemental_files.zip [file TEMI_A_1821582_SM9137.zip › AlignS4.docx]

| AlignS4 |  |
| --- | --- |
| CLUSTAL W (1.7) multiple sequence alignment | |
|  |  |
|  |  |
| blaCTX-M-104_1_HQ833652 | MVTKRVQRMMFAAAACIPLLLGSAPLYAQTSAVQQKLAALEKSSGGRLGVALIDTADNTQ |
| S18BD05011 | MVTKRVQRMMFAAAACIPLLLGSAPLYAQTSAVQQKLAALEKSSGGRLGVALIDTADNTQ |
| blaCTX-M-14_1_AF252622 | MVTKRVQRMMFAAAACIPLLLGSAPLYAQTSAVQQKLAALEKSSGGRLGVALIDTADNTQ |
| blaCTX-M-14b_1_DQ359215 | MVTKRVQRMMFAAAACIPLLLGSAPLYAQTSAVQQKLAALEKSSGGRLGVALIDTADNTQ |
| 15EP001483 | MVTKRVQRMMFAAAACIPLLLGSAPLYAQTSAVQQKLAALEKSSGGRLGVALIDTADNTQ |
| 17EP002363 | MVTKRVQRMMFAAAACIPLLLGSAPLYAQTSAVQQKLAALEKSSGGRLGVALIDTADNTQ |
| 313865 | MVTKRVQRMMFAAAACIPLLLGSAPLYAQTSAVQQKLAALEKSSGGRLGVALIDTADNTQ |
| ERR2173656 | MVTKRVQRMMFAAAACIPLLLGSAPLYAQTSAVQQKLAALEKSSGGRLGVALIDTADNTQ |
| ERR2580274 | MVTKRVQRMMFAAAACIPLLLGSAPLYAQTSAVQQKLAALEKSSGGRLGVALIDTADNTQ |
| ERR2580277 | MVTKRVQRMMFAAAACIPLLLGSAPLYAQTSAVQQKLAALEKSSGGRLGVALIDTADNTQ |
| SRR8704720 | MVTKRVQRMMFAAAACIPLLLGSAPLYAQTSAVQQKLAALEKSSGGRLGVALIDTADNTQ |
| MT16-000061 | MVTKRVQRMMFAAAACIPLLLGSAPLYAQTSAVQQKLAALEKSSGGRLGVALIDTADNTQ |
| MT16-019416 | MVTKRVQRMMFAAAACIPLLLGSAPLYAQTSAVQQKLAALEKSSGGRLGVALIDTADNTQ |
| MT16-027865 | MVTKRVQRMMFAAAACIPLLLGSAPLYAQTSAVQQKLAALEKSSGGRLGVALIDTADNTQ |
| MT16-031693 | MVTKRVQRMMFAAAACIPLLLGSAPLYAQTSAVQQKLAALEKSSGGRLGVALIDTADNTQ |
| MT16-040253 | MVTKRVQRMMFAAAACIPLLLGSAPLYAQTSAVQQKLAALEKSSGGRLGVALIDTADNTQ |
| MT16-045379 | MVTKRVQRMMFAAAACIPLLLGSAPLYAQTSAVQQKLAALEKSSGGRLGVALIDTADNTQ |
| MT16-442728 | MVTKRVQRMMFAAAACIPLLLGSAPLYAQTSAVQQKLAALEKSSGGRLGVALIDTADNTQ |
| MT16-462857 | MVTKRVQRMMFAAAACIPLLLGSAPLYAQTSAVQQKLAALEKSSGGRLGVALIDTADNTQ |
| MT16-480196 | MVTKRVQRMMFAAAACIPLLLGSAPLYAQTSAVQQKLAALEKSSGGRLGVALIDTADNTQ |
| MT16-861555 | MVTKRVQRMMFAAAACIPLLLGSAPLYAQTSAVQQKLAALEKSSGGRLGVALIDTADNTQ |
| MT17-076833 | MVTKRVQRMMFAAAACIPLLLGSAPLYAQTSAVQQKLAALEKSSGGRLGVALIDTADNTQ |
| MT17-110677 | MVTKRVQRMMFAAAACIPLLLGSAPLYAQTSAVQQKLAALEKSSGGRLGVALIDTADNTQ |
| MT17-131730 | MVTKRVQRMMFAAAACIPLLLGSAPLYAQTSAVQQKLAALEKSSGGRLGVALIDTADNTQ |
| MT17-140890 | MVTKRVQRMMFAAAACIPLLLGSAPLYAQTSAVQQKLAALEKSSGGRLGVALIDTADNTQ |
| MT17-141840 | MVTKRVQRMMFAAAACIPLLLGSAPLYAQTSAVQQKLAALEKSSGGRLGVALIDTADNTQ |
| MT17-152488 | MVTKRVQRMMFAAAACIPLLLGSAPLYAQTSAVQQKLAALEKSSGGRLGVALIDTADNTQ |
| MT17-157311 | MVTKRVQRMMFAAAACIPLLLGSAPLYAQTSAVQQKLAALEKSSGGRLGVALIDTADNTQ |
| MT17-161645 | MVTKRVQRMMFAAAACIPLLLGSAPLYAQTSAVQQKLAALEKSSGGRLGVALIDTADNTQ |
| MT17-167951 | MVTKRVQRMMFAAAACIPLLLGSAPLYAQTSAVQQKLAALEKSSGGRLGVALIDTADNTQ |
| MT18-217732 | MVTKRVQRMMFAAAACIPLLLGSAPLYAQTSAVQQKLAALEKSSGGRLGVALIDTADNTQ |
| MT18-252580 | MVTKRVQRMMFAAAACIPLLLGSAPLYAQTSAVQQKLAALEKSSGGRLGVALIDTADNTQ |
| RIVM_H_2016-04 | MVTKRVQRMMFAAAACIPLLLGSAPLYAQTSAVQQKLAALEKSSGGRLGVALIDTADNTQ |
| RIVM_H_2016-07 | MVTKRVQRMMFAAAACIPLLLGSAPLYAQTSAVQQKLAALEKSSGGRLGVALIDTADNTQ |
| RIVM_H_2016-08 | MVTKRVQRMMFAAAACIPLLLGSAPLYAQTSAVQQKLAALEKSSGGRLGVALIDTADNTQ |
| RIVM_H_2016-09 | MVTKRVQRMMFAAAACIPLLLGSAPLYAQTSAVQQKLAALEKSSGGRLGVALIDTADNTQ |
| RIVM_H_2016-11 | MVTKRVQRMMFAAAACIPLLLGSAPLYAQTSAVQQKLAALEKSSGGRLGVALIDTADNTQ |
| RIVM_H_2016-12 | MVTKRVQRMMFAAAACIPLLLGSAPLYAQTSAVQQKLAALEKSSGGRLGVALIDTADNTQ |
| RIVM_H_2016-13 | MVTKRVQRMMFAAAACIPLLLGSAPLYAQTSAVQQKLAALEKSSGGRLGVALIDTADNTQ |
| RIVM_H_2016-14 | MVTKRVQRMMFAAAACIPLLLGSAPLYAQTSAVQQKLAALEKSSGGRLGVALIDTADNTQ |
| RIVM_H_2016-15 | MVTKRVQRMMFAAAACIPLLLGSAPLYAQTSAVQQKLAALEKSSGGRLGVALIDTADNTQ |
| RIVM_H_2017-02 | MVTKRVQRMMFAAAACIPLLLGSAPLYAQTSAVQQKLAALEKSSGGRLGVALIDTADNTQ |
| RIVM_H_2017-03 | MVTKRVQRMMFAAAACIPLLLGSAPLYAQTSAVQQKLAALEKSSGGRLGVALIDTADNTQ |
| RIVM_H_2017-05 | MVTKRVQRMMFAAAACIPLLLGSAPLYAQTSAVQQKLAALEKSSGGRLGVALIDTADNTQ |
| RIVM_H_2017-07 | MVTKRVQRMMFAAAACIPLLLGSAPLYAQTSAVQQKLAALEKSSGGRLGVALIDTADNTQ |
| RIVM_H_2017-08 | MVTKRVQRMMFAAAACIPLLLGSAPLYAQTSAVQQKLAALEKSSGGRLGVALIDTADNTQ |
| RIVM_H_2017-09 | MVTKRVQRMMFAAAACIPLLLGSAPLYAQTSAVQQKLAALEKSSGGRLGVALIDTADNTQ |
| RIVM_H_2017-10 | MVTKRVQRMMFAAAACIPLLLGSAPLYAQTSAVQQKLAALEKSSGGRLGVALIDTADNTQ |
| RIVM_H_2017-11 | MVTKRVQRMMFAAAACIPLLLGSAPLYAQTSAVQQKLAALEKSSGGRLGVALIDTADNTQ |
| RIVM_H_2017-12 | MVTKRVQRMMFAAAACIPLLLGSAPLYAQTSAVQQKLAALEKSSGGRLGVALIDTADNTQ |
| RIVM_H_2017-13 | MVTKRVQRMMFAAAACIPLLLGSAPLYAQTSAVQQKLAALEKSSGGRLGVALIDTADNTQ |
| RIVM_H_2017-14 | MVTKRVQRMMFAAAACIPLLLGSAPLYAQTSAVQQKLAALEKSSGGRLGVALIDTADNTQ |
| RIVM_H_2017-19 | MVTKRVQRMMFAAAACIPLLLGSAPLYAQTSAVQQKLAALEKSSGGRLGVALIDTADNTQ |
| RKI_16-04315 | MVTKRVQRMMFAAAACIPLLLGSAPLYAQTSAVQQKLAALEKSSGGRLGVALIDTADNTQ |
| RKI_17-02304 | MVTKRVQRMMFAAAACIPLLLGSAPLYAQTSAVQQKLAALEKSSGGRLGVALIDTADNTQ |
| RKI_17-02411 | MVTKRVQRMMFAAAACIPLLLGSAPLYAQTSAVQQKLAALEKSSGGRLGVALIDTADNTQ |
| RKI_17-02757 | MVTKRVQRMMFAAAACIPLLLGSAPLYAQTSAVQQKLAALEKSSGGRLGVALIDTADNTQ |
| RKI_17-04797 | MVTKRVQRMMFAAAACIPLLLGSAPLYAQTSAVQQKLAALEKSSGGRLGVALIDTADNTQ |
| S16BD08730 | MVTKRVQRMMFAAAACIPLLLGSAPLYAQTSAVQQKLAALEKSSGGRLGVALIDTADNTQ |
| S18BD03994 | MVTKRVQRMMFAAAACIPLLLGSAPLYAQTSAVQQKLAALEKSSGGRLGVALIDTADNTQ |
| SRR1957844 | MVTKRVQRMMFAAAACIPLLLGSAPLYAQTSAVQQKLAALEKSSGGRLGVALIDTADNTQ |
| SRR1958654 | MVTKRVQRMMFAAAACIPLLLGSAPLYAQTSAVQQKLAALEKSSGGRLGVALIDTADNTQ |
| SRR1965077 | MVTKRVQRMMFAAAACIPLLLGSAPLYAQTSAVQQKLAALEKSSGGRLGVALIDTADNTQ |
| SRR1966369 | MVTKRVQRMMFAAAACIPLLLGSAPLYAQTSAVQQKLAALEKSSGGRLGVALIDTADNTQ |
| SRR1967117 | MVTKRVQRMMFAAAACIPLLLGSAPLYAQTSAVQQKLAALEKSSGGRLGVALIDTADNTQ |
| SRR1967922 | MVTKRVQRMMFAAAACIPLLLGSAPLYAQTSAVQQKLAALEKSSGGRLGVALIDTADNTQ |
| SRR5583183 | MVTKRVQRMMFAAAACIPLLLGSAPLYAQTSAVQQKLAALEKSSGGRLGVALIDTADNTQ |
| SRR5585240 | MVTKRVQRMMFAAAACIPLLLGSAPLYAQTSAVQQKLAALEKSSGGRLGVALIDTADNTQ |
| SRR7216071 | MVTKRVQRMMFAAAACIPLLLGSAPLYAQTSAVQQKLAALEKSSGGRLGVALIDTADNTQ |
| SRR7277793 | MVTKRVQRMMFAAAACIPLLLGSAPLYAQTSAVQQKLAALEKSSGGRLGVALIDTADNTQ |
| SRR7284317 | MVTKRVQRMMFAAAACIPLLLGSAPLYAQTSAVQQKLAALEKSSGGRLGVALIDTADNTQ |
| SRR7299161 | MVTKRVQRMMFAAAACIPLLLGSAPLYAQTSAVQQKLAALEKSSGGRLGVALIDTADNTQ |
| SRR7343877 | MVTKRVQRMMFAAAACIPLLLGSAPLYAQTSAVQQKLAALEKSSGGRLGVALIDTADNTQ |
| SRR7351477 | MVTKRVQRMMFAAAACIPLLLGSAPLYAQTSAVQQKLAALEKSSGGRLGVALIDTADNTQ |
| SRR7401730 | MVTKRVQRMMFAAAACIPLLLGSAPLYAQTSAVQQKLAALEKSSGGRLGVALIDTADNTQ |
| SRR7469092 | MVTKRVQRMMFAAAACIPLLLGSAPLYAQTSAVQQKLAALEKSSGGRLGVALIDTADNTQ |
| SRR7523148 | MVTKRVQRMMFAAAACIPLLLGSAPLYAQTSAVQQKLAALEKSSGGRLGVALIDTADNTQ |
| SRR7523854 | MVTKRVQRMMFAAAACIPLLLGSAPLYAQTSAVQQKLAALEKSSGGRLGVALIDTADNTQ |
| SRR7842487 | MVTKRVQRMMFAAAACIPLLLGSAPLYAQTSAVQQKLAALEKSSGGRLGVALIDTADNTQ |
| SRR7879556 | MVTKRVQRMMFAAAACIPLLLGSAPLYAQTSAVQQKLAALEKSSGGRLGVALIDTADNTQ |
| SRR8054524 | MVTKRVQRMMFAAAACIPLLLGSAPLYAQTSAVQQKLAALEKSSGGRLGVALIDTADNTQ |
| SRR8054525 | MVTKRVQRMMFAAAACIPLLLGSAPLYAQTSAVQQKLAALEKSSGGRLGVALIDTADNTQ |
| SRR8524733 | MVTKRVQRMMFAAAACIPLLLGSAPLYAQTSAVQQKLAALEKSSGGRLGVALIDTADNTQ |
| SRR8526100 | MVTKRVQRMMFAAAACIPLLLGSAPLYAQTSAVQQKLAALEKSSGGRLGVALIDTADNTQ |
| SRR8553991 | MVTKRVQRMMFAAAACIPLLLGSAPLYAQTSAVQQKLAALEKSSGGRLGVALIDTADNTQ |
| blaCTX-M-15_1_AY044436 | MVKKSLRQFTLMATATVTLLLGSVPLYAQTADVQQKLAELERQSGGRLGVALINTADNSQ |
| RIVM_H_2017-15 | MVKKSLRQFTLMATATVTLLLGSVPLYAQTADVQQKLAELERQSGGRLGVALINTADNSQ |
| RKI_17-06869 | MVKKSLRQFTLMATATVTLLLGSVPLYAQTADVQQKLAELERQSGGRLGVALINTADNSQ |
| blaCTX-M-55_1_DQ810789 | MVKKSLRQFTLMATATVTLLLGSVPLYAQTADVQQKLAELERQSGGRLGVALINTADNSQ |
| SRR7349175 | MVKKSLRQFTLMATATVTLLLGSVPLYAQTADVQQKLAELERQSGGRLGVALINTADNSQ |
| ------------------------------------------------------------------------------------ | |
| blaCTX-M-104_1_HQ833652 | VLYRGDERFPMCSTSKVMAAAAVLKQSETQKQLLNQPVEIKPADLVNYNPIAEKHVNGTM |
| S18BD05011 | VLYRGDERFPMCSTSKVMAAAAVLKQSETQKQLLNQPVEIKPADLVNYNPIAEKHVNGTM |
| blaCTX-M-14_1_AF252622 | VLYRGDERFPMCSTSKVMAAAAVLKQSETQKQLLNQPVEIKPADLVNYNPIAEKHVNGTM |
| blaCTX-M-14b_1_DQ359215 | VLYRGDERFPMCSTSKVMAAAAVLKQSETQKQLLNQPVEIKPADLVNYNPIAEKHVNGTM |
| 15EP001483 | VLYRGDERFPMCSTSKVMAAAAVLKQSETQKQLLNQPVEIKPADLVNYNPIAEKHVNGTM |
| 17EP002363 | VLYRGDERFPMCSTSKVMAAAAVLKQSETQKQLLNQPVEIKPADLVNYNPIAEKHVNGTM |
| 313865 | VLYRGDERFPMCSTSKVMAAAAVLKQSETQKQLLNQPVEIKPADLVNYNPIAEKHVNGTM |
| ERR2173656 | VLYRGDERFPMCSTSKVMAAAAVLKQSETQKQLLNQPVEIKPADLVNYNPIAEKHVNGTM |
| ERR2580274 | VLYRGDERFPMCSTSKVMAAAAVLKQSETQKQLLNQPVEIKPADLVNYNPIAEKHVNGTM |
| ERR2580277 | VLYRGDERFPMCSTSKVMAAAAVLKQSETQKQLLNQPVEIKPADLVNYNPIAEKHVNGTM |
| SRR8704720 | VLYRGDERFPMCSTSKVMAAAAVLKQSETQKQLLNQPVEIKPADLVNYNPIAEKHVNGTM |
| MT16-000061 | VLYRGDERFPMCSTSKVMAAAAVLKQSETQKQLLNQPVEIKPADLVNYNPIAEKHVNGTM |
| MT16-019416 | VLYRGDERFPMCSTSKVMAAAAVLKQSETQKQLLNQPVEIKPADLVNYNPIAEKHVNGTM |
| MT16-027865 | VLYRGDERFPMCSTSKVMAAAAVLKQSETQKQLLNQPVEIKPADLVNYNPIAEKHVNGTM |
| MT16-031693 | VLYRGDERFPMCSTSKVMAAAAVLKQSETQKQLLNQPVEIKPADLVNYNPIAEKHVNGTM |
| MT16-040253 | VLYRGDERFPMCSTSKVMAAAAVLKQSETQKQLLNQPVEIKPADLVNYNPIAEKHVNGTM |
| MT16-045379 | VLYRGDERFPMCSTSKVMAAAAVLKQSETQKQLLNQPVEIKPADLVNYNPIAEKHVNGTM |
| MT16-442728 | VLYRGDERFPMCSTSKVMAAAAVLKQSETQKQLLNQPVEIKPADLVNYNPIAEKHVNGTM |
| MT16-462857 | VLYRGDERFPMCSTSKVMAAAAVLKQSETQKQLLNQPVEIKPADLVNYNPIAEKHVNGTM |
| MT16-480196 | VLYRGDERFPMCSTSKVMAAAAVLKQSETQKQLLNQPVEIKPADLVNYNPIAEKHVNGTM |
| MT16-861555 | VLYRGDERFPMCSTSKVMAAAAVLKQSETQKQLLNQPVEIKPADLVNYNPIAEKHVNGTM |
| MT17-076833 | VLYRGDERFPMCSTSKVMAAAAVLKQSETQKQLLNQPVEIKPADLVNYNPIAEKHVNGTM |
| MT17-110677 | VLYRGDERFPMCSTSKVMAAAAVLKQSETQKQLLNQPVEIKPADLVNYNPIAEKHVNGTM |
| MT17-131730 | VLYRGDERFPMCSTSKVMAAAAVLKQSETQKQLLNQPVEIKPADLVNYNPIAEKHVNGTM |
| MT17-140890 | VLYRGDERFPMCSTSKVMAAAAVLKQSETQKQLLNQPVEIKPADLVNYNPIAEKHVNGTM |
| MT17-141840 | VLYRGDERFPMCSTSKVMAAAAVLKQSETQKQLLNQPVEIKPADLVNYNPIAEKHVNGTM |
| MT17-152488 | VLYRGDERFPMCSTSKVMAAAAVLKQSETQKQLLNQPVEIKPADLVNYNPIAEKHVNGTM |
| MT17-157311 | VLYRGDERFPMCSTSKVMAAAAVLKQSETQKQLLNQPVEIKPADLVNYNPIAEKHVNGTM |
| MT17-161645 | VLYRGDERFPMCSTSKVMAAAAVLKQSETQKQLLNQPVEIKPADLVNYNPIAEKHVNGTM |
| MT17-167951 | VLYRGDERFPMCSTSKVMAAAAVLKQSETQKQLLNQPVEIKPADLVNYNPIAEKHVNGTM |
| MT18-217732 | VLYRGDERFPMCSTSKVMAAAAVLKQSETQKQLLNQPVEIKPADLVNYNPIAEKHVNGTM |
| MT18-252580 | VLYRGDERFPMCSTSKVMAAAAVLKQSETQKQLLNQPVEIKPADLVNYNPIAEKHVNGTM |
| RIVM_H_2016-04 | VLYRGDERFPMCSTSKVMAAAAVLKQSETQKQLLNQPVEIKPADLVNYNPIAEKHVNGTM |
| RIVM_H_2016-07 | VLYRGDERFPMCSTSKVMAAAAVLKQSETQKQLLNQPVEIKPADLVNYNPIAEKHVNGTM |
| RIVM_H_2016-08 | VLYRGDERFPMCSTSKVMAAAAVLKQSETQKQLLNQPVEIKPADLVNYNPIAEKHVNGTM |
| RIVM_H_2016-09 | VLYRGDERFPMCSTSKVMAAAAVLKQSETQKQLLNQPVEIKPADLVNYNPIAEKHVNGTM |
| RIVM_H_2016-11 | VLYRGDERFPMCSTSKVMAAAAVLKQSETQKQLLNQPVEIKPADLVNYNPIAEKHVNGTM |
| RIVM_H_2016-12 | VLYRGDERFPMCSTSKVMAAAAVLKQSETQKQLLNQPVEIKPADLVNYNPIAEKHVNGTM |
| RIVM_H_2016-13 | VLYRGDERFPMCSTSKVMAAAAVLKQSETQKQLLNQPVEIKPADLVNYNPIAEKHVNGTM |
| RIVM_H_2016-14 | VLYRGDERFPMCSTSKVMAAAAVLKQSETQKQLLNQPVEIKPADLVNYNPIAEKHVNGTM |
| RIVM_H_2016-15 | VLYRGDERFPMCSTSKVMAAAAVLKQSETQKQLLNQPVEIKPADLVNYNPIAEKHVNGTM |
| RIVM_H_2017-02 | VLYRGDERFPMCSTSKVMAAAAVLKQSETQKQLLNQPVEIKPADLVNYNPIAEKHVNGTM |
| RIVM_H_2017-03 | VLYRGDERFPMCSTSKVMAAAAVLKQSETQKQLLNQPVEIKPADLVNYNPIAEKHVNGTM |
| RIVM_H_2017-05 | VLYRGDERFPMCSTSKVMAAAAVLKQSETQKQLLNQPVEIKPADLVNYNPIAEKHVNGTM |
| RIVM_H_2017-07 | VLYRGDERFPMCSTSKVMAAAAVLKQSETQKQLLNQPVEIKPADLVNYNPIAEKHVNGTM |
| RIVM_H_2017-08 | VLYRGDERFPMCSTSKVMAAAAVLKQSETQKQLLNQPVEIKPADLVNYNPIAEKHVNGTM |
| RIVM_H_2017-09 | VLYRGDERFPMCSTSKVMAAAAVLKQSETQKQLLNQPVEIKPADLVNYNPIAEKHVNGTM |
| RIVM_H_2017-10 | VLYRGDERFPMCSTSKVMAAAAVLKQSETQKQLLNQPVEIKPADLVNYNPIAEKHVNGTM |
| RIVM_H_2017-11 | VLYRGDERFPMCSTSKVMAAAAVLKQSETQKQLLNQPVEIKPADLVNYNPIAEKHVNGTM |
| RIVM_H_2017-12 | VLYRGDERFPMCSTSKVMAAAAVLKQSETQKQLLNQPVEIKPADLVNYNPIAEKHVNGTM |
| RIVM_H_2017-13 | VLYRGDERFPMCSTSKVMAAAAVLKQSETQKQLLNQPVEIKPADLVNYNPIAEKHVNGTM |
| RIVM_H_2017-14 | VLYRGDERFPMCSTSKVMAAAAVLKQSETQKQLLNQPVEIKPADLVNYNPIAEKHVNGTM |
| RIVM_H_2017-19 | VLYRGDERFPMCSTSKVMAAAAVLKQSETQKQLLNQPVEIKPADLVNYNPIAEKHVNGTM |
| RKI_16-04315 | VLYRGDERFPMCSTSKVMAAAAVLKQSETQKQLLNQPVEIKPADLVNYNPIAEKHVNGTM |
| RKI_17-02304 | VLYRGDERFPMCSTSKVMAAAAVLKQSETQKQLLNQPVEIKPADLVNYNPIAEKHVNGTM |
| RKI_17-02411 | VLYRGDERFPMCSTSKVMAAAAVLKQSETQKQLLNQPVEIKPADLVNYNPIAEKHVNGTM |
| RKI_17-02757 | VLYRGDERFPMCSTSKVMAAAAVLKQSETQKQLLNQPVEIKPADLVNYNPIAEKHVNGTM |
| RKI_17-04797 | VLYRGDERFPMCSTSKVMAAAAVLKQSETQKQLLNQPVEIKPADLVNYNPIAEKHVNGTM |
| S16BD08730 | VLYRGDERFPMCSTSKVMAAAAVLKQSETQKQLLNQPVEIKPADLVNYNPIAEKHVNGTM |
| S18BD03994 | VLYRGDERFPMCSTSKVMAAAAVLKQSETQKQLLNQPVEIKPADLVNYNPIAEKHVNGTM |
| SRR1957844 | VLYRGDERFPMCSTSKVMAAAAVLKQSETQKQLLNQPVEIKPADLVNYNPIAEKHVNGTM |
| SRR1958654 | VLYRGDERFPMCSTSKVMAAAAVLKQSETQKQLLNQPVEIKPADLVNYNPIAEKHVNGTM |
| SRR1965077 | VLYRGDERFPMCSTSKVMAAAAVLKQSETQKQLLNQPVEIKPADLVNYNPIAEKHVNGTM |
| SRR1966369 | VLYRGDERFPMCSTSKVMAAAAVLKQSETQKQLLNQPVEIKPADLVNYNPIAEKHVNGTM |
| SRR1967117 | VLYRGDERFPMCSTSKVMAAAAVLKQSETQKQLLNQPVEIKPADLVNYNPIAEKHVNGTM |
| SRR1967922 | VLYRGDERFPMCSTSKVMAAAAVLKQSETQKQLLNQPVEIKPADLVNYNPIAEKHVNGTM |
| SRR5583183 | VLYRGDERFPMCSTSKVMAAAAVLKQSETQKQLLNQPVEIKPADLVNYNPIAEKHVNGTM |
| SRR5585240 | VLYRGDERFPMCSTSKVMAAAAVLKQSETQKQLLNQPVEIKPADLVNYNPIAEKHVNGTM |
| SRR7216071 | VLYRGDERFPMCSTSKVMAAAAVLKQSETQKQLLNQPVEIKPADLVNYNPIAEKHVNGTM |
| SRR7277793 | VLYRGDERFPMCSTSKVMAAAAVLKQSETQKQLLNQPVEIKPADLVNYNPIAEKHVNGTM |
| SRR7284317 | VLYRGDERFPMCSTSKVMAAAAVLKQSETQKQLLNQPVEIKPADLVNYNPIAEKHVNGTM |
| SRR7299161 | VLYRGDERFPMCSTSKVMAAAAVLKQSETQKQLLNQPVEIKPADLVNYNPIAEKHVNGTM |
| SRR7343877 | VLYRGDERFPMCSTSKVMAAAAVLKQSETQKQLLNQPVEIKPADLVNYNPIAEKHVNGTM |
| SRR7351477 | VLYRGDERFPMCSTSKVMAAAAVLKQSETQKQLLNQPVEIKPADLVNYNPIAEKHVNGTM |
| SRR7401730 | VLYRGDERFPMCSTSKVMAAAAVLKQSETQKQLLNQPVEIKPADLVNYNPIAEKHVNGTM |
| SRR7469092 | VLYRGDERFPMCSTSKVMAAAAVLKQSETQKQLLNQPVEIKPADLVNYNPIAEKHVNGTM |
| SRR7523148 | VLYRGDERFPMCSTSKVMAAAAVLKQSETQKQLLNQPVEIKPADLVNYNPIAEKHVNGTM |
| SRR7523854 | VLYRGDERFPMCSTSKVMAAAAVLKQSETQKQLLNQPVEIKPADLVNYNPIAEKHVNGTM |
| SRR7842487 | VLYRGDERFPMCSTSKVMAAAAVLKQSETQKQLLNQPVEIKPADLVNYNPIAEKHVNGTM |
| SRR7879556 | VLYRGDERFPMCSTSKVMAAAAVLKQSETQKQLLNQPVEIKPADLVNYNPIAEKHVNGTM |
| SRR8054524 | VLYRGDERFPMCSTSKVMAAAAVLKQSETQKQLLNQPVEIKPADLVNYNPIAEKHVNGTM |
| SRR8054525 | VLYRGDERFPMCSTSKVMAAAAVLKQSETQKQLLNQPVEIKPADLVNYNPIAEKHVNGTM |
| SRR8524733 | VLYRGDERFPMCSTSKVMAAAAVLKQSETQKQLLNQPVEIKPADLVNYNPIAEKHVNGTM |
| SRR8526100 | VLYRGDERFPMCSTSKVMAAAAVLKQSETQKQLLNQPVEIKPADLVNYNPIAEKHVNGTM |
| SRR8553991 | VLYRGDERFPMCSTSKVMAAAAVLKQSETQKQLLNQPVEIKPADLVNYNPIAEKHVNGTM |
| blaCTX-M-15_1_AY044436 | ILYRADERFAMCSTSKVMAAAAVLKKSESEPNLLNQRVEIKKSDLVNYNPIAEKHVNGTM |
| RIVM_H_2017-15 | ILYRADERFAMCSTSKVMAAAAVLKKSESEPNLLNQRVEIKKSDLVNYNPIAEKHVNGTM |
| RKI_17-06869 | ILYRADERFAMCSTSKVMAAAAVLKKSESEPNLLNQRVEIKKSDLVNYNPIAEKHVNGTM |
| blaCTX-M-55_1_DQ810789 | ILYRADERFAMCSTSKVMAVAAVLKKSESEPNLLNQRVEIKKSDLVNYNPIAEKHVNGTM |
| SRR7349175 | ILYRADERFAMCSTSKVMAVAAVLKKSESEPNLLNQRVEIKKSDLVNYNPIAEKHVNGTM |
| ------------------------------------------------------------------------------------ | |
| blaCTX-M-104_1_HQ833652 | TLAELSAAALQYSDNTAMNKLIAQLGGPGGVTAFARAIGDETFRLDRTEPTLNTAIPGDP |
| S18BD05011 | TLAELSAAALQYSDNTAMNKLIAQLGGPGGVTAFARAIGDETFRLDRTEPTLNTAIPGDP |
| blaCTX-M-14_1_AF252622 | TLAELSAAALQYSDNTAMNKLIAQLGGPGGVTAFARAIGDETFRLDRTEPTLNTAIPGDP |
| blaCTX-M-14b_1_DQ359215 | TLAELSAAALQYSDNTAMNKLIAQLGGPGGVTAFARAIGDETFRLDRTEPTLNTAIPGDP |
| 15EP001483 | TLAELSAAALQYSDNTAMNKLIAQLGGPGGVTAFARAIGDETFRLDRTEPTLNTAIPGDP |
| 17EP002363 | TLAELSAAALQYSDNTAMNKLIAQLGGPGGVTAFARAIGDETFRLDRTEPTLNTAIPGDP |
| 313865 | TLAELSAAALQYSDNTAMNKLIAQLGGPGGVTAFARAIGDETFRLDRTEPTLNTAIPGDP |
| ERR2173656 | TLAELSAAALQYSDNTAMNKLIAQLGGPGGVTAFARAIGDETFRLDRTEPTLNTAIPGDP |
| ERR2580274 | TLAELSAAALQYSDNTAMNKLIAQLGGPGGVTAFARAIGDETFRLDRTEPTLNTAIPGDP |
| ERR2580277 | TLAELSAAALQYSDNTAMNKLIAQLGGPGGVTAFARAIGDETFRLDRTEPTLNTAIPGDP |
| SRR8704720 | TLAELSAAALQYSDNTAMNKLIAQLGGPGGVTAFARAIGDETFRLDRTEPTLNTAIPGDP |
| MT16-000061 | TLAELSAAALQYSDNTAMNKLIAQLGGPGGVTAFARAIGDETFRLDRTEPTLNTAIPGDP |
| MT16-019416 | TLAELSAAALQYSDNTAMNKLIAQLGGPGGVTAFARAIGDETFRLDRTEPTLNTAIPGDP |
| MT16-027865 | TLAELSAAALQYSDNTAMNKLIAQLGGPGGVTAFARAIGDETFRLDRTEPTLNTAIPGDP |
| MT16-031693 | TLAELSAAALQYSDNTAMNKLIAQLGGPGGVTAFARAIGDETFRLDRTEPTLNTAIPGDP |
| MT16-040253 | TLAELSAAALQYSDNTAMNKLIAQLGGPGGVTAFARAIGDETFRLDRTEPTLNTAIPGDP |
| MT16-045379 | TLAELSAAALQYSDNTAMNKLIAQLGGPGGVTAFARAIGDETFRLDRTEPTLNTAIPGDP |
| MT16-442728 | TLAELSAAALQYSDNTAMNKLIAQLGGPGGVTAFARAIGDETFRLDRTEPTLNTAIPGDP |
| MT16-462857 | TLAELSAAALQYSDNTAMNKLIAQLGGPGGVTAFARAIGDETFRLDRTEPTLNTAIPGDP |
| MT16-480196 | TLAELSAAALQYSDNTAMNKLIAQLGGPGGVTAFARAIGDETFRLDRTEPTLNTAIPGDP |
| MT16-861555 | TLAELSAAALQYSDNTAMNKLIAQLGGPGGVTAFARAIGDETFRLDRTEPTLNTAIPGDP |
| MT17-076833 | TLAELSAAALQYSDNTAMNKLIAQLGGPGGVTAFARAIGDETFRLDRTEPTLNTAIPGDP |
| MT17-110677 | TLAELSAAALQYSDNTAMNKLIAQLGGPGGVTAFARAIGDETFRLDRTEPTLNTAIPGDP |
| MT17-131730 | TLAELSAAALQYSDNTAMNKLIAQLGGPGGVTAFARAIGDETFRLDRTEPTLNTAIPGDP |
| MT17-140890 | TLAELSAAALQYSDNTAMNKLIAQLGGPGGVTAFARAIGDETFRLDRTEPTLNTAIPGDP |
| MT17-141840 | TLAELSAAALQYSDNTAMNKLIAQLGGPGGVTAFARAIGDETFRLDRTEPTLNTAIPGDP |
| MT17-152488 | TLAELSAAALQYSDNTAMNKLIAQLGGPGGVTAFARAIGDETFRLDRTEPTLNTAIPGDP |
| MT17-157311 | TLAELSAAALQYSDNTAMNKLIAQLGGPGGVTAFARAIGDETFRLDRTEPTLNTAIPGDP |
| MT17-161645 | TLAELSAAALQYSDNTAMNKLIAQLGGPGGVTAFARAIGDETFRLDRTEPTLNTAIPGDP |
| MT17-167951 | TLAELSAAALQYSDNTAMNKLIAQLGGPGGVTAFARAIGDETFRLDRTEPTLNTAIPGDP |
| MT18-217732 | TLAELSAAALQYSDNTAMNKLIAQLGGPGGVTAFARAIGDETFRLDRTEPTLNTAIPGDP |
| MT18-252580 | TLAELSAAALQYSDNTAMNKLIAQLGGPGGVTAFARAIGDETFRLDRTEPTLNTAIPGDP |
| RIVM_H_2016-04 | TLAELSAAALQYSDNTAMNKLIAQLGGPGGVTAFARAIGDETFRLDRTEPTLNTAIPGDP |
| RIVM_H_2016-07 | TLAELSAAALQYSDNTAMNKLIAQLGGPGGVTAFARAIGDETFRLDRTEPTLNTAIPGDP |
| RIVM_H_2016-08 | TLAELSAAALQYSDNTAMNKLIAQLGGPGGVTAFARAIGDETFRLDRTEPTLNTAIPGDP |
| RIVM_H_2016-09 | TLAELSAAALQYSDNTAMNKLIAQLGGPGGVTAFARAIGDETFRLDRTEPTLNTAIPGDP |
| RIVM_H_2016-11 | TLAELSAAALQYSDNTAMNKLIAQLGGPGGVTAFARAIGDETFRLDRTEPTLNTAIPGDP |
| RIVM_H_2016-12 | TLAELSAAALQYSDNTAMNKLIAQLGGPGGVTAFARAIGDETFRLDRTEPTLNTAIPGDP |
| RIVM_H_2016-13 | TLAELSAAALQYSDNTAMNKLIAQLGGPGGVTAFARAIGDETFRLDRTEPTLNTAIPGDP |
| RIVM_H_2016-14 | TLAELSAAALQYSDNTAMNKLIAQLGGPGGVTAFARAIGDETFRLDRTEPTLNTAIPGDP |
| RIVM_H_2016-15 | TLAELSAAALQYSDNTAMNKLIAQLGGPGGVTAFARAIGDETFRLDRTEPTLNTAIPGDP |
| RIVM_H_2017-02 | TLAELSAAALQYSDNTAMNKLIAQLGGPGGVTAFARAIGDETFRLDRTEPTLNTAIPGDP |
| RIVM_H_2017-03 | TLAELSAAALQYSDNTAMNKLIAQLGGPGGVTAFARAIGDETFRLDRTEPTLNTAIPGDP |
| RIVM_H_2017-05 | TLAELSAAALQYSDNTAMNKLIAQLGGPGGVTAFARAIGDETFRLDRTEPTLNTAIPGDP |
| RIVM_H_2017-07 | TLAELSAAALQYSDNTAMNKLIAQLGGPGGVTAFARAIGDETFRLDRTEPTLNTAIPGDP |
| RIVM_H_2017-08 | TLAELSAAALQYSDNTAMNKLIAQLGGPGGVTAFARAIGDETFRLDRTEPTLNTAIPGDP |
| RIVM_H_2017-09 | TLAELSAAALQYSDNTAMNKLIAQLGGPGGVTAFARAIGDETFRLDRTEPTLNTAIPGDP |
| RIVM_H_2017-10 | TLAELSAAALQYSDNTAMNKLIAQLGGPGGVTAFARAIGDETFRLDRTEPTLNTAIPGDP |
| RIVM_H_2017-11 | TLAELSAAALQYSDNTAMNKLIAQLGGPGGVTAFARAIGDETFRLDRTEPTLNTAIPGDP |
| RIVM_H_2017-12 | TLAELSAAALQYSDNTAMNKLIAQLGGPGGVTAFARAIGDETFRLDRTEPTLNTAIPGDP |
| RIVM_H_2017-13 | TLAELSAAALQYSDNTAMNKLIAQLGGPGGVTAFARAIGDETFRLDRTEPTLNTAIPGDP |
| RIVM_H_2017-14 | TLAELSAAALQYSDNTAMNKLIAQLGGPGGVTAFARAIGDETFRLDRTEPTLNTAIPGDP |
| RIVM_H_2017-19 | TLAELSAAALQYSDNTAMNKLIAQLGGPGGVTAFARAIGDETFRLDRTEPTLNTAIPGDP |
| RKI_16-04315 | TLAELSAAALQYSDNTAMNKLIAQLGGPGGVTAFARAIGDETFRLDRTEPTLNTAIPGDP |
| RKI_17-02304 | TLAELSAAALQYSDNTAMNKLIAQLGGPGGVTAFARAIGDETFRLDRTEPTLNTAIPGDP |
| RKI_17-02411 | TLAELSAAALQYSDNTAMNKLIAQLGGPGGVTAFARAIGDETFRLDRTEPTLNTAIPGDP |
| RKI_17-02757 | TLAELSAAALQYSDNTAMNKLIAQLGGPGGVTAFARAIGDETFRLDRTEPTLNTAIPGDP |
| RKI_17-04797 | TLAELSAAALQYSDNTAMNKLIAQLGGPGGVTAFARAIGDETFRLDRTEPTLNTAIPGDP |
| S16BD08730 | TLAELSAAALQYSDNTAMNKLIAQLGGPGGVTAFARAIGDETFRLDRTEPTLNTAIPGDP |
| S18BD03994 | TLAELSAAALQYSDNTAMNKLIAQLGGPGGVTAFARAIGDETFRLDRTEPTLNTAIPGDP |
| SRR1957844 | TLAELSAAALQYSDNTAMNKLIAQLGGPGGVTAFARAIGDETFRLDRTEPTLNTAIPGDP |
| SRR1958654 | TLAELSAAALQYSDNTAMNKLIAQLGGPGGVTAFARAIGDETFRLDRTEPTLNTAIPGDP |
| SRR1965077 | TLAELSAAALQYSDNTAMNKLIAQLGGPGGVTAFARAIGDETFRLDRTEPTLNTAIPGDP |
| SRR1966369 | TLAELSAAALQYSDNTAMNKLIAQLGGPGGVTAFARAIGDETFRLDRTEPTLNTAIPGDP |
| SRR1967117 | TLAELSAAALQYSDNTAMNKLIAQLGGPGGVTAFARAIGDETFRLDRTEPTLNTAIPGDP |
| SRR1967922 | TLAELSAAALQYSDNTAMNKLIAQLGGPGGVTAFARAIGDETFRLDRTEPTLNTAIPGDP |
| SRR5583183 | TLAELSAAALQYSDNTAMNKLIAQLGGPGGVTAFARAIGDETFRLDRTEPTLNTAIPGDP |
| SRR5585240 | TLAELSAAALQYSDNTAMNKLIAQLGGPGGVTAFARAIGDETFRLDRTEPTLNTAIPGDP |
| SRR7216071 | TLAELSAAALQYSDNTAMNKLIAQLGGPGGVTAFARAIGDETFRLDRTEPTLNTAIPGDP |
| SRR7277793 | TLAELSAAALQYSDNTAMNKLIAQLGGPGGVTAFARAIGDETFRLDRTEPTLNTAIPGDP |
| SRR7284317 | TLAELSAAALQYSDNTAMNKLIAQLGGPGGVTAFARAIGDETFRLDRTEPTLNTAIPGDP |
| SRR7299161 | TLAELSAAALQYSDNTAMNKLIAQLGGPGGVTAFARAIGDETFRLDRTEPTLNTAIPGDP |
| SRR7343877 | TLAELSAAALQYSDNTAMNKLIAQLGGPGGVTAFARAIGDETFRLDRTEPTLNTAIPGDP |
| SRR7351477 | TLAELSAAALQYSDNTAMNKLIAQLGGPGGVTAFARAIGDETFRLDRTEPTLNTAIPGDP |
| SRR7401730 | TLAELSAAALQYSDNTAMNKLIAQLGGPGGVTAFARAIGDETFRLDRTEPTLNTAIPGDP |
| SRR7469092 | TLAELSAAALQYSDNTAMNKLIAQLGGPGGVTAFARAIGDETFRLDRTEPTLNTAIPGDP |
| SRR7523148 | TLAELSAAALQYSDNTAMNKLIAQLGGPGGVTAFARAIGDETFRLDRTEPTLNTAIPGDP |
| SRR7523854 | TLAELSAAALQYSDNTAMNKLIAQLGGPGGVTAFARAIGDETFRLDRTEPTLNTAIPGDP |
| SRR7842487 | TLAELSAAALQYSDNTAMNKLIAQLGGPGGVTAFARAIGDETFRLDRTEPTLNTAIPGDP |
| SRR7879556 | TLAELSAAALQYSDNTAMNKLIAQLGGPGGVTAFARAIGDETFRLDRTEPTLNTAIPGDP |
| SRR8054524 | TLAELSAAALQYSDNTAMNKLIAQLGGPGGVTAFARAIGDETFRLDRTEPTLNTAIPGDP |
| SRR8054525 | TLAELSAAALQYSDNTAMNKLIAQLGGPGGVTAFARAIGDETFRLDRTEPTLNTAIPGDP |
| SRR8524733 | TLAELSAAALQYSDNTAMNKLIAQLGGPGGVTAFARAIGDETFRLDRTEPTLNTAIPGDP |
| SRR8526100 | TLAELSAAALQYSDNTAMNKLIAQLGGPGGVTAFARAIGDETFRLDRTEPTLNTAIPGDP |
| SRR8553991 | TLAELSAAALQYSDNTAMNKLIAQLGGPGGVTAFARAIGDETFRLDRTEPTLNTAIPGDP |
| blaCTX-M-15_1_AY044436 | SLAELSAAALQYSDNVAMNKLIAHVGGPASVTAFARQLGDETFRLDRTEPTLNTAIPGDP |
| RIVM_H_2017-15 | SLAELSAAALQYSDNVAMNKLIAHVGGPASVTAFARQLGDETFRLDRTEPTLNTAIPGDP |
| RKI_17-06869 | SLAELSAAALQYSDNVAMNKLIAHVGGPASVTAFARQLGDETFRLDRTEPTLNTAIPGDP |
| blaCTX-M-55_1_DQ810789 | SLAELSAAALQYSDNVAMNKLIAHVGGPASVTAFARQLGDETFRLDRTEPTLNTAIPGDP |
| SRR7349175 | SLAELSAAALQYSDNVAMNKLIAHVGGPASVTAFARQLGDETFRLDRTEPTLNTAIPGDP |
| ------------------------------------------------------------------------------------ | |
| blaCTX-M-104_1_HQ833652 | RDTTTPRAMAQTLRQLTLGHALGETQRAQLVTWLKGNTTGAASIRAGLPTSWTVGDKTGS |
| S18BD05011 | RDTTTPRAMAQTLRQLTLGHALGETQRAQLVTWLKGNTTGAASIRAGLPTSWTVGDKTGS |
| blaCTX-M-14_1_AF252622 | RDTTTPRAMAQTLRQLTLGHALGETQRAQLVTWLKGNTTGAASIRAGLPTSWTVGDKTGS |
| blaCTX-M-14b_1_DQ359215 | RDTTTPRAMAQTLRQLTLGHALGETQRAQLVTWLKGNTTGAASIRAGLPTSWTVGDKTGS |
| 15EP001483 | RDTTTPRAMAQTLRQLTLGHALGETQRAQLVTWLKGNTTGAASIRAGLPTSWTVGDKTGS |
| 17EP002363 | RDTTTPRAMAQTLRQLTLGHALGETQRAQLVTWLKGNTTGAASIRAGLPTSWTVGDKTGS |
| 313865 | RDTTTPRAMAQTLRQLTLGHALGETQRAQLVTWLKGNTTGAASIRAGLPTSWTVGDKTGS |
| ERR2173656 | RDTTTPRAMAQTLRQLTLGHALGETQRAQLVTWLKGNTTGAASIRAGLPTSWTVGDKTGS |
| ERR2580274 | RDTTTPRAMAQTLRQLTLGHALGETQRAQLVTWLKGNTTGAASIRAGLPTSWTVGDKTGS |
| ERR2580277 | RDTTTPRAMAQTLRQLTLGHALGETQRAQLVTWLKGNTTGAASIRAGLPTSWTVGDKTGS |
| SRR8704720 | RDTTTPRAMAQTLRQLTLGHALGETQRAQLVTWLKGNTTGAASIRAGLPTSWTVGDKTGS |
| MT16-000061 | RDTTTPRAMAQTLRQLTLGHALGETQRAQLVTWLKGNTTGAASIRAGLPTSWTVGDKTGS |
| MT16-019416 | RDTTTPRAMAQTLRQLTLGHALGETQRAQLVTWLKGNTTGAASIRAGLPTSWTVGDKTGS |
| MT16-027865 | RDTTTPRAMAQTLRQLTLGHALGETQRAQLVTWLKGNTTGAASIRAGLPTSWTVGDKTGS |
| MT16-031693 | RDTTTPRAMAQTLRQLTLGHALGETQRAQLVTWLKGNTTGAASIRAGLPTSWTVGDKTGS |
| MT16-040253 | RDTTTPRAMAQTLRQLTLGHALGETQRAQLVTWLKGNTTGAASIRAGLPTSWTVGDKTGS |
| MT16-045379 | RDTTTPRAMAQTLRQLTLGHALGETQRAQLVTWLKGNTTGAASIRAGLPTSWTVGDKTGS |
| MT16-442728 | RDTTTPRAMAQTLRQLTLGHALGETQRAQLVTWLKGNTTGAASIRAGLPTSWTVGDKTGS |
| MT16-462857 | RDTTTPRAMAQTLRQLTLGHALGETQRAQLVTWLKGNTTGAASIRAGLPTSWTVGDKTGS |
| MT16-480196 | RDTTTPRAMAQTLRQLTLGHALGETQRAQLVTWLKGNTTGAASIRAGLPTSWTVGDKTGS |
| MT16-861555 | RDTTTPRAMAQTLRQLTLGHALGETQRAQLVTWLKGNTTGAASIRAGLPTSWTVGDKTGS |
| MT17-076833 | RDTTTPRAMAQTLRQLTLGHALGETQRAQLVTWLKGNTTGAASIRAGLPTSWTVGDKTGS |
| MT17-110677 | RDTTTPRAMAQTLRQLTLGHALGETQRAQLVTWLKGNTTGAASIRAGLPTSWTVGDKTGS |
| MT17-131730 | RDTTTPRAMAQTLRQLTLGHALGETQRAQLVTWLKGNTTGAASIRAGLPTSWTVGDKTGS |
| MT17-140890 | RDTTTPRAMAQTLRQLTLGHALGETQRAQLVTWLKGNTTGAASIRAGLPTSWTVGDKTGS |
| MT17-141840 | RDTTTPRAMAQTLRQLTLGHALGETQRAQLVTWLKGNTTGAASIRAGLPTSWTVGDKTGS |
| MT17-152488 | RDTTTPRAMAQTLRQLTLGHALGETQRAQLVTWLKGNTTGAASIRAGLPTSWTVGDKTGS |
| MT17-157311 | RDTTTPRAMAQTLRQLTLGHALGETQRAQLVTWLKGNTTGAASIRAGLPTSWTVGDKTGS |
| MT17-161645 | RDTTTPRAMAQTLRQLTLGHALGETQRAQLVTWLKGNTTGAASIRAGLPTSWTVGDKTGS |
| MT17-167951 | RDTTTPRAMAQTLRQLTLGHALGETQRAQLVTWLKGNTTGAASIRAGLPTSWTVGDKTGS |
| MT18-217732 | RDTTTPRAMAQTLRQLTLGHALGETQRAQLVTWLKGNTTGAASIRAGLPTSWTVGDKTGS |
| MT18-252580 | RDTTTPRAMAQTLRQLTLGHALGETQRAQLVTWLKGNTTGAASIRAGLPTSWTVGDKTGS |
| RIVM_H_2016-04 | RDTTTPRAMAQTLRQLTLGHALGETQRAQLVTWLKGNTTGAASIRAGLPTSWTVGDKTGS |
| RIVM_H_2016-07 | RDTTTPRAMAQTLRQLTLGHALGETQRAQLVTWLKGNTTGAASIRAGLPTSWTVGDKTGS |
| RIVM_H_2016-08 | RDTTTPRAMAQTLRQLTLGHALGETQRAQLVTWLKGNTTGAASIRAGLPTSWTVGDKTGS |
| RIVM_H_2016-09 | RDTTTPRAMAQTLRQLTLGHALGETQRAQLVTWLKGNTTGAASIRAGLPTSWTVGDKTGS |
| RIVM_H_2016-11 | RDTTTPRAMAQTLRQLTLGHALGETQRAQLVTWLKGNTTGAASIRAGLPTSWTVGDKTGS |
| RIVM_H_2016-12 | RDTTTPRAMAQTLRQLTLGHALGETQRAQLVTWLKGNTTGAASIRAGLPTSWTVGDKTGS |
| RIVM_H_2016-13 | RDTTTPRAMAQTLRQLTLGHALGETQRAQLVTWLKGNTTGAASIRAGLPTSWTVGDKTGS |
| RIVM_H_2016-14 | RDTTTPRAMAQTLRQLTLGHALGETQRAQLVTWLKGNTTGAASIRAGLPTSWTVGDKTGS |
| RIVM_H_2016-15 | RDTTTPRAMAQTLRQLTLGHALGETQRAQLVTWLKGNTTGAASIRAGLPTSWTVGDKTGS |
| RIVM_H_2017-02 | RDTTTPRAMAQTLRQLTLGHALGETQRAQLVTWLKGNTTGAASIRAGLPTSWTVGDKTGS |
| RIVM_H_2017-03 | RDTTTPRAMAQTLRQLTLGHALGETQRAQLVTWLKGNTTGAASIRAGLPTSWTVGDKTGS |
| RIVM_H_2017-05 | RDTTTPRAMAQTLRQLTLGHALGETQRAQLVTWLKGNTTGAASIRAGLPTSWTVGDKTGS |
| RIVM_H_2017-07 | RDTTTPRAMAQTLRQLTLGHALGETQRAQLVTWLKGNTTGAASIRAGLPTSWTVGDKTGS |
| RIVM_H_2017-08 | RDTTTPRAMAQTLRQLTLGHALGETQRAQLVTWLKGNTTGAASIRAGLPTSWTVGDKTGS |
| RIVM_H_2017-09 | RDTTTPRAMAQTLRQLTLGHALGETQRAQLVTWLKGNTTGAASIRAGLPTSWTVGDKTGS |
| RIVM_H_2017-10 | RDTTTPRAMAQTLRQLTLGHALGETQRAQLVTWLKGNTTGAASIRAGLPTSWTVGDKTGS |
| RIVM_H_2017-11 | RDTTTPRAMAQTLRQLTLGHALGETQRAQLVTWLKGNTTGAASIRAGLPTSWTVGDKTGS |
| RIVM_H_2017-12 | RDTTTPRAMAQTLRQLTLGHALGETQRAQLVTWLKGNTTGAASIRAGLPTSWTVGDKTGS |
| RIVM_H_2017-13 | RDTTTPRAMAQTLRQLTLGHALGETQRAQLVTWLKGNTTGAASIRAGLPTSWTVGDKTGS |
| RIVM_H_2017-14 | RDTTTPRAMAQTLRQLTLGHALGETQRAQLVTWLKGNTTGAASIRAGLPTSWTVGDKTGS |
| RIVM_H_2017-19 | RDTTTPRAMAQTLRQLTLGHALGETQRAQLVTWLKGNTTGAASIRAGLPTSWTVGDKTGS |
| RKI_16-04315 | RDTTTPRAMAQTLRQLTLGHALGETQRAQLVTWLKGNTTGAASIRAGLPTSWTVGDKTGS |
| RKI_17-02304 | RDTTTPRAMAQTLRQLTLGHALGETQRAQLVTWLKGNTTGAASIRAGLPTSWTVGDKTGS |
| RKI_17-02411 | RDTTTPRAMAQTLRQLTLGHALGETQRAQLVTWLKGNTTGAASIRAGLPTSWTVGDKTGS |
| RKI_17-02757 | RDTTTPRAMAQTLRQLTLGHALGETQRAQLVTWLKGNTTGAASIRAGLPTSWTVGDKTGS |
| RKI_17-04797 | RDTTTPRAMAQTLRQLTLGHALGETQRAQLVTWLKGNTTGAASIRAGLPTSWTVGDKTGS |
| S16BD08730 | RDTTTPRAMAQTLRQLTLGHALGETQRAQLVTWLKGNTTGAASIRAGLPTSWTVGDKTGS |
| S18BD03994 | RDTTTPRAMAQTLRQLTLGHALGETQRAQLVTWLKGNTTGAASIRAGLPTSWTVGDKTGS |
| SRR1957844 | RDTTTPRAMAQTLRQLTLGHALGETQRAQLVTWLKGNTTGAASIRAGLPTSWTVGDKTGS |
| SRR1958654 | RDTTTPRAMAQTLRQLTLGHALGETQRAQLVTWLKGNTTGAASIRAGLPTSWTVGDKTGS |
| SRR1965077 | RDTTTPRAMAQTLRQLTLGHALGETQRAQLVTWLKGNTTGAASIRAGLPTSWTVGDKTGS |
| SRR1966369 | RDTTTPRAMAQTLRQLTLGHALGETQRAQLVTWLKGNTTGAASIRAGLPTSWTVGDKTGS |
| SRR1967117 | RDTTTPRAMAQTLRQLTLGHALGETQRAQLVTWLKGNTTGAASIRAGLPTSWTVGDKTGS |
| SRR1967922 | RDTTTPRAMAQTLRQLTLGHALGETQRAQLVTWLKGNTTGAASIRAGLPTSWTVGDKTGS |
| SRR5583183 | RDTTTPRAMAQTLRQLTLGHALGETQRAQLVTWLKGNTTGAASIRAGLPTSWTVGDKTGS |
| SRR5585240 | RDTTTPRAMAQTLRQLTLGHALGETQRAQLVTWLKGNTTGAASIRAGLPTSWTVGDKTGS |
| SRR7216071 | RDTTTPRAMAQTLRQLTLGHALGETQRAQLVTWLKGNTTGAASIRAGLPTSWTVGDKTGS |
| SRR7277793 | RDTTTPRAMAQTLRQLTLGHALGETQRAQLVTWLKGNTTGAASIRAGLPTSWTVGDKTGS |
| SRR7284317 | RDTTTPRAMAQTLRQLTLGHALGETQRAQLVTWLKGNTTGAASIRAGLPTSWTVGDKTGS |
| SRR7299161 | RDTTTPRAMAQTLRQLTLGHALGETQRAQLVTWLKGNTTGAASIRAGLPTSWTVGDKTGS |
| SRR7343877 | RDTTTPRAMAQTLRQLTLGHALGETQRAQLVTWLKGNTTGAASIRAGLPTSWTVGDKTGS |
| SRR7351477 | RDTTTPRAMAQTLRQLTLGHALGETQRAQLVTWLKGNTTGAASIRAGLPTSWTVGDKTGS |
| SRR7401730 | RDTTTPRAMAQTLRQLTLGHALGETQRAQLVTWLKGNTTGAASIRAGLPTSWTVGDKTGS |
| SRR7469092 | RDTTTPRAMAQTLRQLTLGHALGETQRAQLVTWLKGNTTGAASIRAGLPTSWTVGDKTGS |
| SRR7523148 | RDTTTPRAMAQTLRQLTLGHALGETQRAQLVTWLKGNTTGAASIRAGLPTSWTVGDKTGS |
| SRR7523854 | RDTTTPRAMAQTLRQLTLGHALGETQRAQLVTWLKGNTTGAASIRAGLPTSWTVGDKTGS |
| SRR7842487 | RDTTTPRAMAQTLRQLTLGHALGETQRAQLVTWLKGNTTGAASIRAGLPTSWTVGDKTGS |
| SRR7879556 | RDTTTPRAMAQTLRQLTLGHALGETQRAQLVTWLKGNTTGAASIRAGLPTSWTVGDKTGS |
| SRR8054524 | RDTTTPRAMAQTLRQLTLGHALGETQRAQLVTWLKGNTTGAASIRAGLPTSWTVGDKTGS |
| SRR8054525 | RDTTTPRAMAQTLRQLTLGHALGETQRAQLVTWLKGNTTGAASIRAGLPTSWTVGDKTGS |
| SRR8524733 | RDTTTPRAMAQTLRQLTLGHALGETQRAQLVTWLKGNTTGAASIRAGLPTSWTVGDKTGS |
| SRR8526100 | RDTTTPRAMAQTLRQLTLGHALGETQRAQLVTWLKGNTTGAASIRAGLPTSWTVGDKTGS |
| SRR8553991 | RDTTTPRAMAQTLRQLTLGHALGETQRAQLVTWLKGNTTGAASIRAGLPTSWTVGDKTGS |
| blaCTX-M-15_1_AY044436 | RDTTSPRAMAQTLRNLTLGKALGDSQRAQLVTWMKGNTTGAASIQAGLPASWVVGDKTGS |
| RIVM_H_2017-15 | RDTTSPRAMAQTLRNLTLGKALGDSQRAQLVTWMKGNTTGAASIQAGLPASWVVGDKTGS |
| RKI_17-06869 | RDTTSPRAMAQTLRNLTLGKALGDSQRAQLVTWMKGNTTGAASIQAGLPASWVVGDKTGS |
| blaCTX-M-55_1_DQ810789 | RDTTSPRAMAQTLRNLTLGKALGDSQRAQLVTWMKGNTTGAASIQAGLPASWVVGDKTGS |
| SRR7349175 | RDTTSPRAMAQTLRNLTLGKALGDSQRAQLVTWMKGNTTGAASIQAGLPASWVVGDKTGS |
| ------------------------------------------------------------------------------------ | |
| blaCTX-M-104_1_HQ833652 | GDYGTTNDIAVIWPQGRAPLVLVTYFTQPQQNAENRRDVLASAARIIAEGL |
| S18BD05011 | GDYGTTNDIAVIWPQGRAPLVLVTYFTQPQQNAENRRDVLASAARIIAEGL |
| blaCTX-M-14_1_AF252622 | GDYGTTNDIAVIWPQGRAPLVLVTYFTQPQQNAESRRDVLASAARIIAEGL |
| blaCTX-M-14b_1_DQ359215 | GDYGTTNDIAVIWPQGRAPLVLVTYFTQPQQNAESRRDVLASAARIIAEGL |
| 15EP001483 | GDYGTTNDIAVIWPQGRAPLVLVTYFTQPQQNAESRRDVLASAARIIAEGL |
| 17EP002363 | GDYGTTNDIAVIWPQGRAPLVLVTYFTQPQQNAESRRDVLASAARIIAEGL |
| 313865 | GDYGTTNDIAVIWPQGRAPLVLVTYFTQPQQNAESRRDVLASAARIIAEGL |
| ERR2173656 | GDYGTTNDIAVIWPQGRAPLVLVTYFTQPQQNAESRRDVLASAARIIAEGL |
| ERR2580274 | GDYGTTNDIAVIWPQGRAPLVLVTYFTQPQQNAESRRDVLASAARIIAEGL |
| ERR2580277 | GDYGTTNDIAVIWPQGRAPLVLVTYFTQPQQNAESRRDVLASAARIIAEGL |
| SRR8704720 | GDYGTTNDIAVIWPQGRAPLVLVTYFTQPQQNAESRRDVLASAARIIAEGL |
| MT16-000061 | GDYGTTNDIAVIWPQGRAPLVLVTYFTQPQQNAESRRDVLASAARIIAEGL |
| MT16-019416 | GDYGTTNDIAVIWPQGRAPLVLVTYFTQPQQNAESRRDVLASAARIIAEGL |
| MT16-027865 | GDYGTTNDIAVIWPQGRAPLVLVTYFTQPQQNAESRRDVLASAARIIAEGL |
| MT16-031693 | GDYGTTNDIAVIWPQGRAPLVLVTYFTQPQQNAESRRDVLASAARIIAEGL |
| MT16-040253 | GDYGTTNDIAVIWPQGRAPLVLVTYFTQPQQNAESRRDVLASAARIIAEGL |
| MT16-045379 | GDYGTTNDIAVIWPQGRAPLVLVTYFTQPQQNAESRRDVLASAARIIAEGL |
| MT16-442728 | GDYGTTNDIAVIWPQGRAPLVLVTYFTQPQQNAESRRDVLASAARIIAEGL |
| MT16-462857 | GDYGTTNDIAVIWPQGRAPLVLVTYFTQPQQNAESRRDVLASAARIIAEGL |
| MT16-480196 | GDYGTTNDIAVIWPQGRAPLVLVTYFTQPQQNAESRRDVLASAARIIAEGL |
| MT16-861555 | GDYGTTNDIAVIWPQGRAPLVLVTYFTQPQQNAESRRDVLASAARIIAEGL |
| MT17-076833 | GDYGTTNDIAVIWPQGRAPLVLVTYFTQPQQNAESRRDVLASAARIIAEGL |
| MT17-110677 | GDYGTTNDIAVIWPQGRAPLVLVTYFTQPQQNAESRRDVLASAARIIAEGL |
| MT17-131730 | GDYGTTNDIAVIWPQGRAPLVLVTYFTQPQQNAESRRDVLASAARIIAEGL |
| MT17-140890 | GDYGTTNDIAVIWPQGRAPLVLVTYFTQPQQNAESRRDVLASAARIIAEGL |
| MT17-141840 | GDYGTTNDIAVIWPQGRAPLVLVTYFTQPQQNAESRRDVLASAARIIAEGL |
| MT17-152488 | GDYGTTNDIAVIWPQGRAPLVLVTYFTQPQQNAESRRDVLASAARIIAEGL |
| MT17-157311 | GDYGTTNDIAVIWPQGRAPLVLVTYFTQPQQNAESRRDVLASAARIIAEGL |
| MT17-161645 | GDYGTTNDIAVIWPQGRAPLVLVTYFTQPQQNAESRRDVLASAARIIAEGL |
| MT17-167951 | GDYGTTNDIAVIWPQGRAPLVLVTYFTQPQQNAESRRDVLASAARIIAEGL |
| MT18-217732 | GDYGTTNDIAVIWPQGRAPLVLVTYFTQPQQNAESRRDVLASAARIIAEGL |
| MT18-252580 | GDYGTTNDIAVIWPQGRAPLVLVTYFTQPQQNAESRRDVLASAARIIAEGL |
| RIVM_H_2016-04 | GDYGTTNDIAVIWPQGRAPLVLVTYFTQPQQNAESRRDVLASAARIIAEGL |
| RIVM_H_2016-07 | GDYGTTNDIAVIWPQGRAPLVLVTYFTQPQQNAESRRDVLASAARIIAEGL |
| RIVM_H_2016-08 | GDYGTTNDIAVIWPQGRAPLVLVTYFTQPQQNAESRRDVLASAARIIAEGL |
| RIVM_H_2016-09 | GDYGTTNDIAVIWPQGRAPLVLVTYFTQPQQNAESRRDVLASAARIIAEGL |
| RIVM_H_2016-11 | GDYGTTNDIAVIWPQGRAPLVLVTYFTQPQQNAESRRDVLASAARIIAEGL |
| RIVM_H_2016-12 | GDYGTTNDIAVIWPQGRAPLVLVTYFTQPQQNAESRRDVLASAARIIAEGL |
| RIVM_H_2016-13 | GDYGTTNDIAVIWPQGRAPLVLVTYFTQPQQNAESRRDVLASAARIIAEGL |
| RIVM_H_2016-14 | GDYGTTNDIAVIWPQGRAPLVLVTYFTQPQQNAESRRDVLASAARIIAEGL |
| RIVM_H_2016-15 | GDYGTTNDIAVIWPQGRAPLVLVTYFTQPQQNAESRRDVLASAARIIAEGL |
| RIVM_H_2017-02 | GDYGTTNDIAVIWPQGRAPLVLVTYFTQPQQNAESRRDVLASAARIIAEGL |
| RIVM_H_2017-03 | GDYGTTNDIAVIWPQGRAPLVLVTYFTQPQQNAESRRDVLASAARIIAEGL |
| RIVM_H_2017-05 | GDYGTTNDIAVIWPQGRAPLVLVTYFTQPQQNAESRRDVLASAARIIAEGL |
| RIVM_H_2017-07 | GDYGTTNDIAVIWPQGRAPLVLVTYFTQPQQNAESRRDVLASAARIIAEGL |
| RIVM_H_2017-08 | GDYGTTNDIAVIWPQGRAPLVLVTYFTQPQQNAESRRDVLASAARIIAEGL |
| RIVM_H_2017-09 | GDYGTTNDIAVIWPQGRAPLVLVTYFTQPQQNAESRRDVLASAARIIAEGL |
| RIVM_H_2017-10 | GDYGTTNDIAVIWPQGRAPLVLVTYFTQPQQNAESRRDVLASAARIIAEGL |
| RIVM_H_2017-11 | GDYGTTNDIAVIWPQGRAPLVLVTYFTQPQQNAESRRDVLASAARIIAEGL |
| RIVM_H_2017-12 | GDYGTTNDIAVIWPQGRAPLVLVTYFTQPQQNAESRRDVLASAARIIAEGL |
| RIVM_H_2017-13 | GDYGTTNDIAVIWPQGRAPLVLVTYFTQPQQNAESRRDVLASAARIIAEGL |
| RIVM_H_2017-14 | GDYGTTNDIAVIWPQGRAPLVLVTYFTQPQQNAESRRDVLASAARIIAEGL |
| RIVM_H_2017-19 | GDYGTTNDIAVIWPQGRAPLVLVTYFTQPQQNAESRRDVLASAARIIAEGL |
| RKI_16-04315 | GDYGTTNDIAVIWPQGRAPLVLVTYFTQPQQNAESRRDVLASAARIIAEGL |
| RKI_17-02304 | GDYGTTNDIAVIWPQGRAPLVLVTYFTQPQQNAESRRDVLASAARIIAEGL |
| RKI_17-02411 | GDYGTTNDIAVIWPQGRAPLVLVTYFTQPQQNAESRRDVLASAARIIAEGL |
| RKI_17-02757 | GDYGTTNDIAVIWPQGRAPLVLVTYFTQPQQNAESRRDVLASAARIIAEGL |
| RKI_17-04797 | GDYGTTNDIAVIWPQGRAPLVLVTYFTQPQQNAESRRDVLASAARIIAEGL |
| S16BD08730 | GDYGTTNDIAVIWPQGRAPLVLVTYFTQPQQNAESRRDVLASAARIIAEGL |
| S18BD03994 | GDYGTTNDIAVIWPQGRAPLVLVTYFTQPQQNAESRRDVLASAARIIAEGL |
| SRR1957844 | GDYGTTNDIAVIWPQGRAPLVLVTYFTQPQQNAESRRDVLASAARIIAEGL |
| SRR1958654 | GDYGTTNDIAVIWPQGRAPLVLVTYFTQPQQNAESRRDVLASAARIIAEGL |
| SRR1965077 | GDYGTTNDIAVIWPQGRAPLVLVTYFTQPQQNAESRRDVLASAARIIAEGL |
| SRR1966369 | GDYGTTNDIAVIWPQGRAPLVLVTYFTQPQQNAESRRDVLASAARIIAEGL |
| SRR1967117 | GDYGTTNDIAVIWPQGRAPLVLVTYFTQPQQNAESRRDVLASAARIIAEGL |
| SRR1967922 | GDYGTTNDIAVIWPQGRAPLVLVTYFTQPQQNAESRRDVLASAARIIAEGL |
| SRR5583183 | GDYGTTNDIAVIWPQGRAPLVLVTYFTQPQQNAESRRDVLASAARIIAEGL |
| SRR5585240 | GDYGTTNDIAVIWPQGRAPLVLVTYFTQPQQNAESRRDVLASAARIIAEGL |
| SRR7216071 | GDYGTTNDIAVIWPQGRAPLVLVTYFTQPQQNAESRRDVLASAARIIAEGL |
| SRR7277793 | GDYGTTNDIAVIWPQGRAPLVLVTYFTQPQQNAESRRDVLASAARIIAEGL |
| SRR7284317 | GDYGTTNDIAVIWPQGRAPLVLVTYFTQPQQNAESRRDVLASAARIIAEGL |
| SRR7299161 | GDYGTTNDIAVIWPQGRAPLVLVTYFTQPQQNAESRRDVLASAARIIAEGL |
| SRR7343877 | GDYGTTNDIAVIWPQGRAPLVLVTYFTQPQQNAENRRDVLASAARIIAEGL |
| SRR7351477 | GDYGTTNDIAVIWPQGRAPLVLVTYFTQPQQNAESRRDVLASAARIIAEGL |
| SRR7401730 | GDYGTTNDIAVIWPQGRAPLVLVTYFTQPQQNAESRRDVLASAARIIAEGL |
| SRR7469092 | GDYGTTNDIAVIWPQGRAPLVLVTYFTQPQQNAESRRDVLASAARIIAEGL |
| SRR7523148 | GDYGTTNDIAVIWPQGRAPLVLVTYFTQPQQNAENRRDVLASAARIIAEGL |
| SRR7523854 | GDYGTTNDIAVIWPQGRAPLVLVTYFTQPQQNAESRRDVLASAARIIAEGL |
| SRR7842487 | GDYGTTNDIAVIWPQGRAPLVLVTYFTQPQQNAESRRDVLASAARIIAEGL |
| SRR7879556 | GDYGTTNDIAVIWPQGRAPLVLVTYFTQPQQNAESRRDVLASAARIIAEGL |
| SRR8054524 | GDYGTTNDIAVIWPQGRAPLVLVTYFTQPQQNAESRRDVLASAARIIAEGL |
| SRR8054525 | GDYGTTNDIAVIWPQGRAPLVLVTYFTQPQQNAESRRDVLASAARIIAEGL |
| SRR8524733 | GDYGTTNDIAVIWPQGRAPLVLVTYFTQPQQNAESRRDVLASAARIIAEGL |
| SRR8526100 | GDYGTTNDIAVIWPQGRAPLVLVTYFTQPQQNAESRRDVLASAARIIAEGL |
| SRR8553991 | GDYGTTNDIAVIWPQGRAPLVLVTYFTQPQQNAESRRDVLASAARIIAEGL |
| blaCTX-M-15_1_AY044436 | GGYGTTNDIAVIWPKDRAPLILVTYFTQPQPKAESRRDVLASAAKIVTDGL |
| RIVM_H_2017-15 | GGYGTTNDIAVIWPKDRAPLILVTYFTQPQPKAESRRDVLASAAKIVTDGL |
| RKI_17-06869 | GGYGTTNDIAVIWPKDRAPLILVTYFTQPQPKAESRRDVLASAAKIVTDGL |
| blaCTX-M-55_1_DQ810789 | GGYGTTNDIAVIWPKDRAPLILVTYFTQPQPKAESRRDVLASAAKIVTDGL |
| SRR7349175 | GGYGTTNDIAVIWPKDRAPLILVTYFTQPQPKAESRRDVLASAAKIVTDGL |
